# A Comprehensive Description and Evolutionary Analysis of 22 Grouper (Perciformes, Epinephelidae) Mitochondrial Genomes with Emphasis on Two Novel Genome Organizations

**DOI:** 10.1371/journal.pone.0073561

**Published:** 2013-08-09

**Authors:** Xuan Zhuang, Meng Qu, Xiang Zhang, Shaoxiong Ding

**Affiliations:** 1 State Key Laboratory of Marine Environmental Science, Xiamen University, Xiamen, China; 2 The Laboratory of Marine Biodiversity and Global Change, Xiamen University, Xiamen, China; 3 Department of Animal Biology, University of Illinois at Urbana-Champaign, Champaign, Illinois, United States of America; University of Cyprus, Cyprus

## Abstract

Groupers of the family Epinephelidae are a diverse and economically valuable group of reef fishes. To investigate the evolution of their mitochondrial genomes we characterized and compared these genomes among 22 species, 17 newly sequenced. Among these fishes we identified three distinct genome organizations, two of them never previously reported in vertebrates. In 19 of these species, mitochondrial genomes followed the typical vertebrate canonical organization with 13 protein-coding genes, 22 *tRNAs*, two *rRNAs*, and a non-coding control region. Differing from this, members of genus *Variola* have an extra *tRNA-Ile* between *tRNA-Val* and *16S rRNA*. Evidence suggests that this evolved from *tRNA-Val* via a duplication event due to slipped strand mispairing during replication. Additionally, *Cephalopholis* *argus* has an extra *tRNA-Asp* in the midst of the control region, likely resulting from long-range duplication of the canonical *tRNA-Asp* through illicit priming of mitochondrial replication by tRNAs. Along with their gene contents, we characterized the regulatory elements of these mitochondrial genomes’ control regions, including putative termination-associated sequences and conserved sequence blocks. Looking at the mitochondrial genomic constituents, *rRNA* and *tRNA* are the most conserved, followed by protein-coding genes, and non-coding regions are the most divergent. Divergence rates vary among the protein-coding genes, and the three cytochrome oxidase subunits (*COI, II, III*) are the most conserved, while NADH dehydrogenase subunit 6 (*ND6*) and the ATP synthase subunit 8 (ATP8) are the most divergent. We then tested the phylogenetic utility of this new mt genome data using 12 protein-coding genes of 48 species from the suborder Percoidei. From this, we provide further support for the elevation of the subfamily Epinephelinae to family Epinephelidae, the resurrection of the genus *Hyporthodus*, and the combination of the monotypic genera *Anyperodon* and *Cromileptes* to genus *Epinephelus* , and *Aethaloperca* to genus *Cephalopholis* *.*

## Introduction

The Perciform family Epinephelidae (previously subfamily Epinephelinae in family Serranidae), commonly known as groupers, is an assemblage of reef fishes comprising more than 160 species in 16 genera [1]. These commercially important fishes inhabit coastal areas of the tropics and subtropics, with species frequently sharing small geographic ranges. Their evolutionary biology has been of continuing interest due in part to their biological diversity and apparent rapid sympatric speciation [1–3]. While many areas of their evolutionary biology are under active investigation, current understanding of the structure and evolution of the groupers’ mitochondrial (mt) genome is limited as few have been sequenced [4,5] and none have yet to be comprehensively described. This is of particular interest as the mt genome presents a simpler system than the nuclear genome for studying the molecular dynamics and mechanisms of rearrangements that underlie variations in the genome.

While organization of the fish mt genome was long thought to be highly conserved, there is increasing appreciation that it may be considerably more plastic than originally believed. The canonical fish mt genome contains 13 protein-coding genes (*ATP8*, *ATP6*, *COI-III*, Cyt *b*, *ND1-6* and *ND4L*), two ribosomal RNA genes (*12S rRNA* and *16S rRNA*), and 22 transfer RNA genes (*tRNAs*). Additionally, there are two major non-coding regions involved in mtDNA replication and transcription, the light-strand (L-strand) origin of replication (O_L_) and the control region (CR) [6]. However, recent work has shown that the fish mt genomes can differ from this canonical organization in both gene content and order, with several recognized hotspots of reorganization. These include the *tRNA-WANCY* cluster (W, Trp; A, Ala; N, Asn; C, Cys; Y, Tyr) located between *ND2* and *COI* [7,8], the *tRNA-IQM* cluster (I, Ile; Q, Gln; M, Met) located between *ND1* and *ND2* [9,10], and the CR, usually involved in the translocation of *ND6* and *tRNA-Glu* to CR [11,12]. In addition to these hotspots, large-scale reorganizations of the mt genome have also been observed in some fishes [13,14]. Our interest in this study is first in comparing the mt genome arrangement and sequence among the groupers for insight into the evolutionary dynamics and molecular mechanisms that have shaped them. Beyond this though, these fully sequenced mt genomes provide a broadly useful molecular resource for these fishes.

Among its other uses, several features of the mtDNA make it particularly attractive as a tool for studying evolutionary biology [15]. These include its small size, cellular abundance, maternal inheritance, compact gene arrangement, and high rate of evolution [16]. Thus, mtDNA is generally considered a good molecular marker for phylogenetic analyses among fish taxa. However, short mt gene fragments exhibit limitations in resolving complicated phylogenetic relationships in many fish lineages [17]. The additional informative sites from longer DNA sequences (e.g. mt genomes) allow these deeper branches and higher-level relationships to be more fully resolved [18]. Hence, the mt genomes provided in this study may help resolve the evolutionary relationships among the groupers [1,19,20] where recent studies have led to suggested taxonomic revisions.

Additionally, the mt genomes from these fishes can be applied towards species identification and in aid of conservation efforts for this commercially important group. mtDNA has been used for DNA barcoding allowing for identification on the level of species or population. This is particularly useful for groupers where similarities in color patterns, a lack of morphological variation, and ontogenetic changes combine to cause taxonomic confusion [21]. Such improved means of identification are important for future efforts to manage grouper fisheries with 33 grouper species already assessed as ‘endangered’, ‘vulnerable’, or ‘near threatened’ on the ICUN (International Union for Conservation of Nature and Natural Resources) Red List [22].

To better understand the structure of the grouper mt genome and its evolution we took a detailed view of its organization, gene content, and functional regions in 22 species, including representatives from nine of the 16 genera in family Epinephelidae (Table 1). Not only does this provide new insight into novel mt genome organization among these fishes, but the additional informative sites available from these fully sequenced mt genomes allows us to test recently proposed taxonomic revisions. Finally, this work also provides important new molecular resources for the species identification, fishery management, and conservation biology of groupers.

**Table 1:** Genome sizes and nucleotide compositions for the mitochondrial genomes of 22 grouper species analyzed in this study and sampling locations of the 17 newly sequenced ones.

Species			Sampling location			Genome size (bp)			Base compositions (%)								AT-skew		GC-skew	
									A		T		G		C					
*Aethaloperca* *rogaa*			HongKong, China			16538			29.4		27.3		15.9		27.4		0.04		-0.27	
*Anyperodon* *leucogrammicus*			—			16616			28.7		26.9		15.7		28.7		0.03		-0.29	
*Cephalopholis* *argus*			Taiwan			16767			29.2		27.7		16.2		26.9		0.03		-0.25	
*Cephalopholis* *sonnerati*			HongKong, China			16587			29.6		26.2		15.9		28.3		0.06		-0.28	
*Cromileptes* *altivelis*			Hainan, China			16504			29.1		26.3		15.7		29.0		0.05		-0.30	
*Epinephelus* *akaara*			Fujian, China			16795			28.7		27.3		16.2		27.8		0.03		-0.26	
*Epinephelus* *areolatus*			Hainan, China			16965			28.7		27.1		16.3		28.0		0.03		-0.26	
*Epinephelus* *awoara*			Fujian, China			16802			28.5		27.3		16.5		27.7		0.02		-0.25	
*Epinephelus* *bruneus*			—			16692			28.5		26.6		16.2		28.7		0.03		-0.28	
*Epinephelus* *coioides*			Fujian, China			16418			28.7		26.5		15.8		28.9		0.04		-0.29	
*Epinephelus* *epistictus*			Hainan, China			16927			29.2		27.0		15.7		28.1		0.04		-0.28	
*Epinephelus* *fuscoguttatus*			Hainan, China			16648			29.2		26.9		15.6		28.3		0.04		-0.29	
*Epinephelus* *lanceolatus*			—			16714			29.5		26.5		15.1		28.8		0.05		-0.31	
*Epinephelus* *moara*			—			16696			28.6		26.5		16.1		28.9		0.04		-0.28	
*Epinephelus* *trimaculatus*			Hainan, China			16777			29.0		27.1		15.9		28.0		0.03		-0.27	
*Hyporthodus* *octofasciatus*			Fujian, China			16545			28.6		27.4		16.3		27.8		0.02		-0.26	
*Hyporthodusseptemfasciatus*			—			16558			28.6		26.8		16.3		28.3		0.03		-0.27	
*Plectropomus* *areolatus*			HongKong, China			16770			28.8		27.6		16.3		27.2		0.02		-0.25	
*Plectropomus* *leopardus*			Fujian, China			16753			29.1		28.0		16.2		26.7		0.02		-0.24	
*Triso* *dermopterus*			Hainan, China			16605			28.2		25.7		16.7		29.4		0.05		-0.28	
*Variola* *albimarginata*			Taiwan			16768			28.4		27.7		16.4		27.6		0.01		-0.26	
*Variola* *louti*			Hainan, China			16770			28.4		27.8		16.3		27.5		0.01		-0.26	

The species with listed sampling locations were sequenced in this study. These sampling locations are for fish markets where specimens were purchased. In China fresh groupers are usually only available in fish markets that are supplied by local fishermen, making these representative of the local fish fauna.

## Results and Discussion

### Canonical genome organization and composition

**Figure 1:**
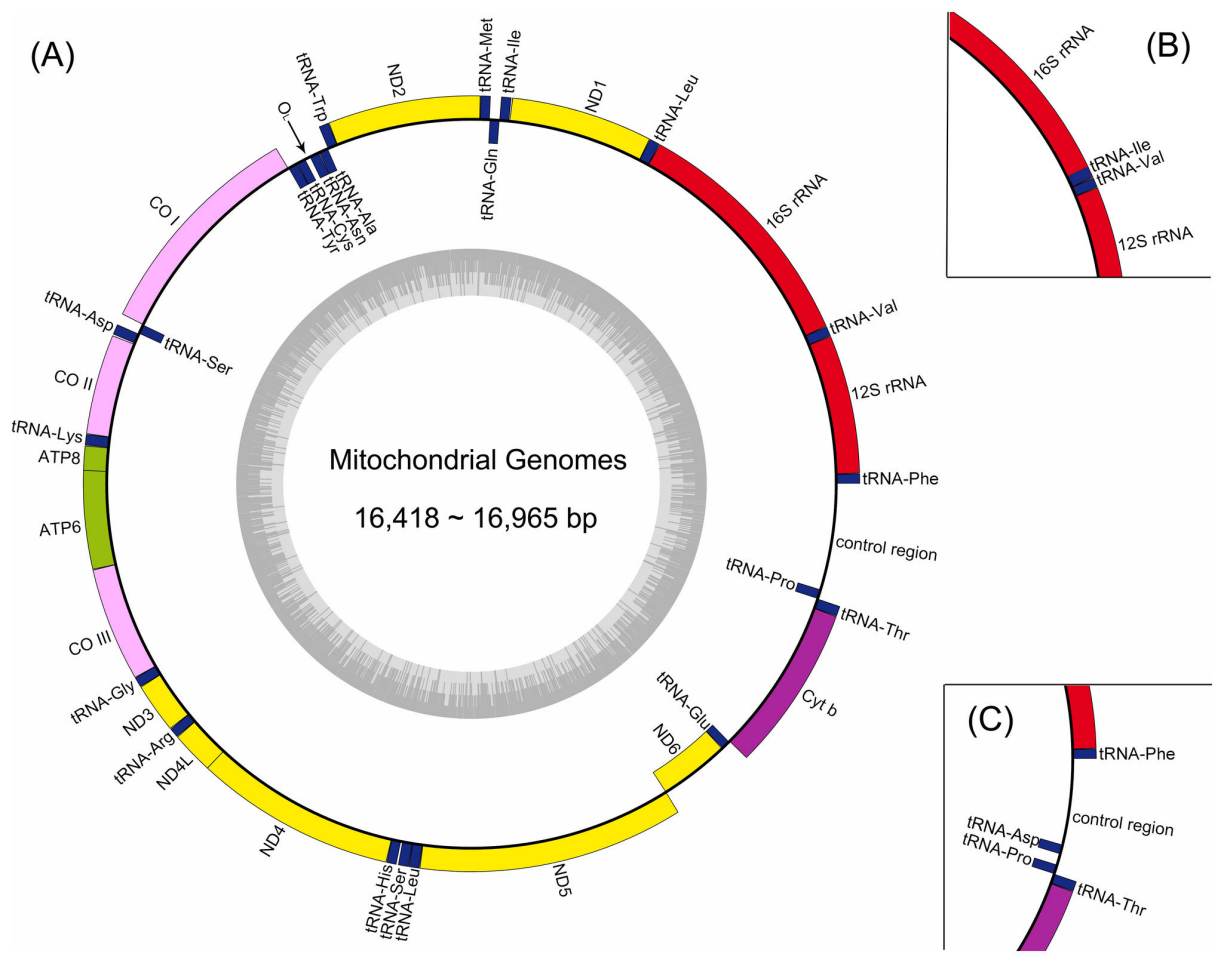
Gene maps for mitochondrial genomes. Genes encoded on the heavy and light strand are shown outside and inside the circle, respectively. The inner grey ring indicates the GC content. This genome map was constructed via OrganellarGenomeDRAW [81] with manual modifications. (A) The mt gemone organization of *Aethaloperca rogaa*, *Anyperodon leucogrammicus*, *Cephalopholis sonnerati*, *Cromileptes altivelis*, *Epinephelus akaara*, *E*. *areolatus*, *E*. *awoara*, *E*. *bruneus*, *E*. *coioides*, *E*. *epistictus*, *E*. *fuscoguttatus*, *E*. *lanceolatus*, *E*. *moara*, *E*. *trimaculatus*, *Hyporthodus octofasciatus*, *H*. *septemfasciatus*, *Plectropomus areolatus*, *P*. *leopardus*, and *Triso dermopterus*. (B) The mt gemone organization of genera *Variola, V*. *albimarginata* and *V*. *louti*. (C) The mt gemone organization of *Cephalopholis argus*.

The mt genomes of the 22 analyzed groupers range in size from 16,418 to 16,965 bp and show three distinct organizations. While *Variola* *albimarginata* , *V* *. louti* , and *Cephalopholis* *argus* have novel mt genome organization, the 19 remaining species share the canonical mt gene content and arrangement (Figure 1A and Table 2). The canonical mt genomes of the 19 species contain 37 genes encoding 13 proteins, 22 tRNAs, and two rRNAs. As in many teleosts, most of the mt genes are located on the heavy-strand (H-strand) with the exceptions of *ND6* and eight *tRNA* (*tRNA-Gln*, *tRNA-Ala*, *tRNA-Asn*, *tRNA-Cys*, *tRNA-Tyr*, *tRNA-Ser*(*UCN*), *tRNA-Glu*, *tRNA-Pro*), which are encoded on the L-strand. The overall genome nucleotide identity between these 19 mt genomes which show identical synteny is 83.9%.

**Table 2:** Gene content of the mitochondrial genomes of 19 grouper species with canonical genome organization (genome structure shown in Figure 1A).

Gene or region	Size		Codon		Intergenic nucleotides*	Strand
	Nucleotide (bp)	Amino acid	Start	Stop		
tRNA-Phe	69 to 71				0	H
12S rRNA	952 to 961				0	H
tRNA-Val	70 to 73				-1 to 2	H
16S rRNA	1695 to 1722				0 to 20	H
tRNA-Leu(UUR)	73 to 76				0	H
ND1	975	324	ATG	TAA/TAG	0	H
tRNA-Ile	69 to 70				4 to 6	H
tRNA-Gln	71				-2 to 0	L
tRNA-Met	69 to 70				-1 to 0	H
ND2	1045 to 1048	348 to 349	ATG	T/TA/TAA/TAG	0	H
tRNA-Trp	70 to 72				-2 to 4	H
tRNA-Ala	69				1 to 2	L
tRNA-Asn	73 to 75				-2 to 1	L
O_L_	33 to 43				-1 to 0	-
tRNA-Cys	66 to 68				0 to 27	L
tRNA-Tyr	70 to 71				0 to 1	L
COI	1551	516	GTG	TAA/TAG	1	H
tRNA-Ser(UCN)	71				0 to 3	L
tRNA-Asp	72 to 73				2 to 8	H
COII	691	230	ATG	T	0 to 8	H
tRNA-Lys	72 to 74				0 to 1	H
ATPase8	168	55	ATG	TAA	0 to 1	H
ATPase6	684	227	ATG/CTG/TTG	TAA	-10	H
COIII	785 to 786	261	ATG	TA/TAA	-1	H
tRNA-Gly	70 to 73				-1 to 0	H
ND3	349	116	ATG	T	-2 to 0	H
tRNA-Arg	69				0	H
ND4L	297	98	ATG	TAA	0	H
ND4	1381	460	ATG	T	-7	H
tRNA-His	69 to 73				0	H
tRNA-Ser(AGY)	70 to 82				0 to 10	H
tRNA-Leu(CUN)	73 to 74				0 to 10	H
ND5	1836 to 1839	611 to 612	ATG	TAA/TAG	-2 to 0	H
ND6	522	173	ATG	TAA/TAG	-4	L
tRNA-Glu	69 to 70				0	L
Cytb	1141 to 1144	380	ATG	T	2 to 9	H
tRNA-Thr	72 to 74				0 to 1	H
tRNA-Pro	70 to 71				-1 to 4	L
Control Region	721 to 1264				0	-

* Negative numbers indicate overlapping nucleotides between adjacent genes.

The nucleotide compositions of the 22 mt genomes are shown in Table 1. Strand asymmetry of nucleotide composition is usually described by AT and GC skews. The mt genome AT-skews of these groupers are barely above zero, while the GC-skews are all negative. These results indicate that the A content is only slightly higher than T, whereas C is considerably more prevalent than G. Such skews towards a particular nucleotide are attributed to differential mutational pressures imposed on the L- and H-strands [23], resulting from the asymmetric replication of mtDNA [24,25].

### Novel genome organizations and their plausible evolutionary mechanisms

Two novel types of mt genome organizations were found among the groupers, each containing an additional *tRNA*. Both species of genus *Variola* have an additional intergenic spacer of around 100 bp between *tRNA-Val* and *16S rRNA*. This spacer can fold into the conventional cloverleaf structure of tRNA and includes an anticodon of *tRNA-Ile*. This additional *tRNA-Ile* shares no meaningful sequence similarity with the canonical *tRNA-Ile* between *ND1* and *tRNA-Gln*. Furthermore, it has an anticodon AAU different from the GAU of canonical *tRNA-Ile*. However, the additional *tRNA-Ile* shares a high sequence identity with the adjacent *tRNA-Val* (83% in *V* *. albimarginata* and 76% in *V* . *louti* ), and in both species it is encoded by the H-strand. Thus, we speculate the new *tRNA-Ile* may have evolved from *tRNA-Val* via a duplication event due to slipped strand mispairing during mtDNA replication. Nucleotide mutations of the anticodon from TAC to AAT then switched the redundant *tRNA-Val* (anticodon UAC) to a new *tRNA-Ile* (anticodon AAU). This novel mt genome organization is a genus-specific characteristic of *Variola* and distinct from other grouper genera (Figure 1B).

Similar intergenic sequences between *tRNA-Val* and *16S rRNA* have also been observed in another clade of Serranidae fishes, fairy basslets in subfamily Anthiinae [26]. Although the intergenic sequences of Anthiinae do not reveal cloverleaf secondary structure of tRNA, they still can be folded into stem-loop structures and were also thought to have been retained from the ancestral *tRNA-Val* [26]. Therefore, this can be regarded as a case of convergent evolution in which similar mt genome rearrangements have occurred independently in two separate lineages of Percoidei fishes.

The other novel mt genome organization was observed in *C* *. argus* . This species has an additional *tRNA-Asp* on the L-strand inserted in the middle of the CR (Figure 1C). This tRNA gene shares 97% sequence identity and the same anticodon GUC with the canonical H-strand coded *tRNA-Asp* found between *tRNA-Ser* (*UCN*) and *COII*. This additional copy is therefore likely the result of a long-range duplication event, which could be explained by illicit priming of mitochondrial replication by tRNAs and the resultant integration of tRNA genes in the CR [27,28]. Although the canonical *tRNA-Asp* is distant from the H-strand replication origin (O_H_) in the CR, at the start of replication the tRNA encoded by this gene could illegitimately prime DNA synthesis of the H-strand which would lead to an extra copy of the tRNA gene within the CR on the L-strand.

The gene contents of fish mt genomes are generally stable with variation typically the result of gene duplications [29,30]. What mt genome rearrangements have been seen in fishes are typically gene translocations [13,31,32], resulting from tandem duplication of gene regions. These are then often followed by deletions of the functionally redundant duplicated gene; however, there are examples of *tRNAs* retained in the mt genome after duplication. Beyond the additional *tRNA-Ile* and *tRNA-Asp* described here, the non-canonical *tRNAs* observed thus far in fish mt genomes include the extra *tRNA-Ser* downstream of *ND5* in seabass *Morone* *saxatilis* [33], the extra *tRNA-Met* in the *tRNA-IQM* cluster in *Pampus* species [10] and the pseudo *tRNA-Met* in the same position in parrotfish *Chlorurus* *sordidus* [9], the additional *tRNA-Asn* and pseudo *tRNA-Ala* in the *tRNA-WANCY* cluster in polar cod *Boreogadus* *saida* [7], the duplicated *tRNA-Thr* and *tRNA-Pro* in the CR of Antarctic notothenioids [12]. While new *tRNAs* appear to be scattered through different mt regions, most in fact remain located at the rearrangement hotspots (*tRNA* clusters and CR) in the fish mt genome.

### Protein-coding genes

The cumulative lengths of the groupers’ mt protein coding genes range from 11,408 to 11,417 bp. This accounts for 69.5% to 67.3% of the mt genome’s total length. Animal mtDNA is generally very compact and contains some overlapping regions between the adjacent protein-coding genes. Among the 13 protein-coding genes in these groupers, we found a 10 bp overlap between *ATP8* and *ATP6*, 7 bp between *ND4L* and *ND4*, 4 bp between *ND5* and *ND6*, and 1 bp between *ATP6* and *COIII* (Table 2).

**Figure 2:**
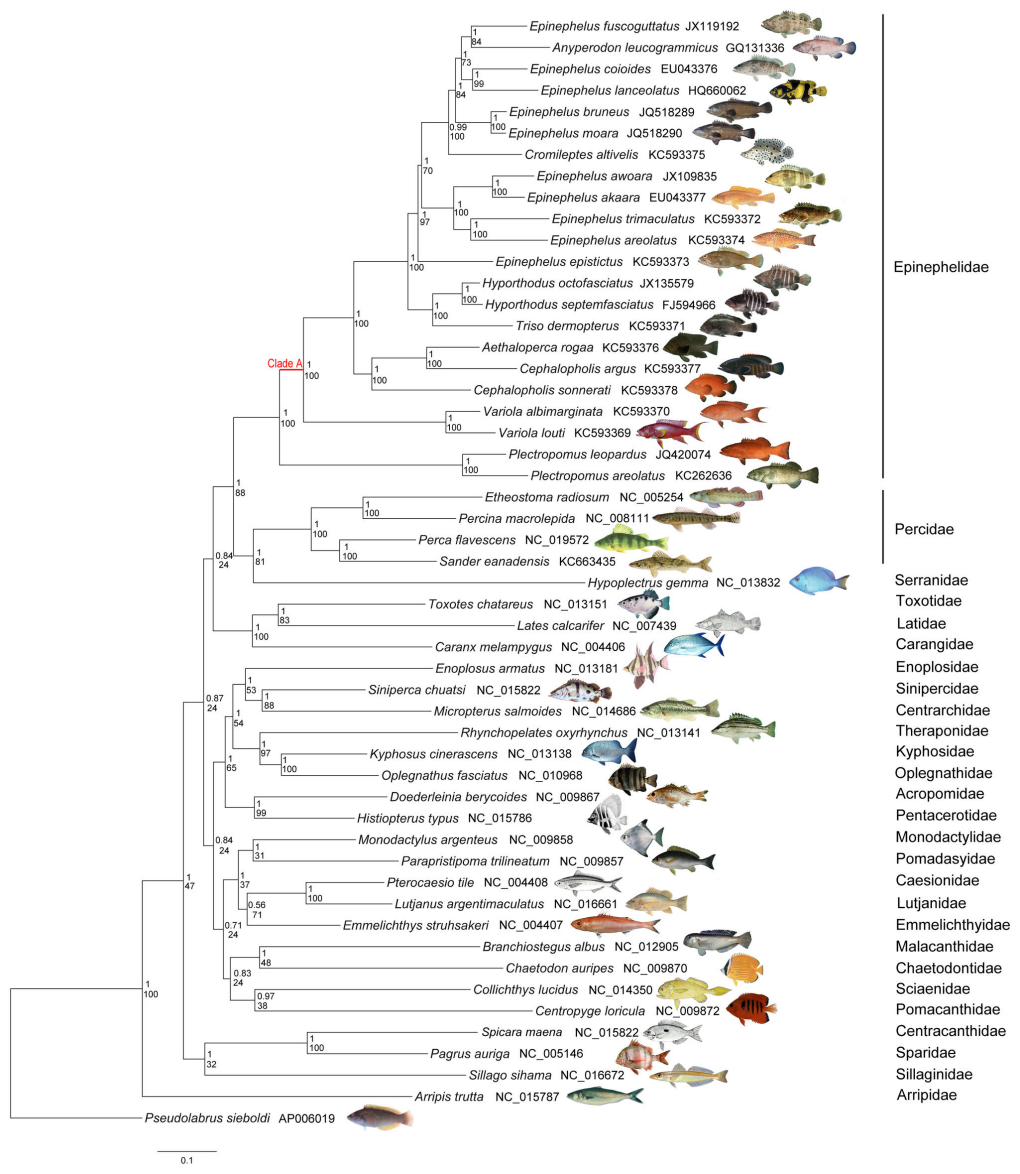
Phylogenetic tree of 22 groupers in family Epinephelidae and 29 representatives from other families in suborder Percoidei. A species from the suborder Labrodei family Labridae, *Pseudolabrus sieboldi*, was selected as outgroup. Congruent tree topology was inferred from partitioned Maximum likelihood and Bayesian analyses using the concatenated nucleotide sequences of 12 mitochondrial protein-coding genes (excluding *ND6*). The Bayesian posterior probability values (top) and bootstrap values (bottom) are labeled at branch nodes. Branch length information from the Bayesian tree is shown. NCBI RefSeq or GenBank accession number of each species was listed on the right of the species name. Clade A indicates the derived epinephelid clade whose *ATP6* start codon is not ATG but CTG or TTG, different from most other teleosts and basal groupers.

Most of the grouper mt protein-coding genes begin with the typical start codon ATG. As in many other metazoans [16], the grouper *COI* gene differs in that it begins with GTG. Additionally, while most teleost fishes retain ATG as the *ATP6* start codon, this is CTG or TTG in groupers outside of the basal genus *Plectropomus* [19,34,35]. This latter difference is not common among teleosts, and could be considered a linage-specific characteristic of the more derived epinephelid clade (clade A in Figure 2) with the exceptions of *E* *. bruneus* , *E* *. moara* , and *H* *. septemfasciatus* *.*

Unlike the start codon, variation is more common in the termination codons of mt genes [16]. Among the groupers this was seen in the protein-coding genes *COII*, *ND2*, *ND3*, *ND4*, and Cyt *b* all of which terminate with the incomplete stop codon T or TA (Table 2). These stop codons are completed with the addition of 3' adenine residues to the mRNA by post-transcriptional polyadenylation [36,37].

Calculating the nucleotide base frequency at each codon position across all 13 mt protein-coding genes (Table 3), T was found over-represented at the second codon position (P < 0.01). Since the triplet codons with T at the second positions all code for hydrophobic residues this observed bias is explained by the high proportion of hydrophobic residues among mt transmembrane proteins. The 12 protein genes encoded on H-strand share a strong anti-G bias (lower than 8%, P < 0.01) at the third codon positions typical in vertebrates [38]. However, *ND6* encoded on the L-strand exhibits the opposite trend with a strong anti-C bias (lower than 10%, P < 0.01) at the third codon positions. This may suggest that the well-known mitochondrial anti-G bias is actually strand-speciﬁc rather than a common feature for all mt protein genes. Moreover, among groupers this bias is primarily at the third codon positions, likely due to the less stringent selective pressure on synonymous mutations at this position.

**Table 3:** Nucleotide compositions of different regions in grouper mitochondrial genomes.

Strand	Gene/Region	Position	Nucleotide composition (%)				
			A	T	G	C	A+T
H	Protein-coding gene	1^st^ codon	26.3±0.4	21.0±0.4	24.8±0.4	27.9±0.5	47.3±0.8
		2^nd^ codon	18.3±0.1	40.5±0.2	13.2±0.1	28.0±0.2	58.8±0.3
		3^rd^ codon	35.5±1.3	24.4±2.5	6.1±1.1	34.3±2.4	59.9±3.8
	tRNA gene		30.6±0.6	25.6±0.4	21.1±0.6	22.7±0.6	56.2±1.0
	rRNA gene		32.2±0.5	21.5±0.5	20.9±0.3	25.4±0.7	53.7±1.0
	Control region		34.5±1.7	32.9±2.0	13.8±1.1	18.8±2.5	67.4±3.7
L	Protein-coding gene (ND6)	1^st^ codon	15.1±1.3	30.5±1.5	42.9±1.8	11.5±1.2	45.6±2.8
		2^nd^ codon	11.8±0.7	42.4±1.6	23.3±1.1	22.5±1.7	54.2±2.2
		3^rd^ codon	21.8±4.9	41.8±3.8	27.8±3.0	8.6±2.8	63.6±8.7
	tRNA gene		25.6±0.6	31.9±0.6	25.7±0.7	16.9±0.4	57.5±1.2

Numbers in the table are means and standard deviations of 22 grouper species.

### Ribosomal and transfer RNA genes

Comparing *rRNA* among groupers, *12S rRNAs* range in length from 952 to 961 bp, and *16S rRNAs* from 1,695 to 1,722 bp. The two *rRNAs*, both encoded by the H-strand, indicate moderate nucleotide compositional bias with A% > C% > T% > G%(P < 0.01) (Table 3). This compositional bias is similar to, but not as strong as, that observed in H-strand coded protein-coding genes. Thus, this confirms that the biased nucleotide composition is a strand-specific characteristic of mtDNA. Similar phenomena have been found in the mt genomes of other fishes [39] and mammals [40], which is thought to be correlated with asymmetric replication of the L- and H-strand [40]. The reduced compositional bias of *rRNA* compared to protein-coding genes is likely due to its structural constraints, particularly in stem regions [41].

Except for the two *Variola* species and *C* *. argus* , the mt genomes of the remaining 19 grouper species all contain 22 *tRNAs*, 14 of which are encoded by the H-strand and eight encoded by L-strand. Among the 22 *tRNAs*, two forms of *tRNA-Leu* (*UUR and CUN*) and *tRNA-Ser* (*UCN and AGY*) were observed in all groupers (Table 2). These *tRNAs* range in size from 66 to 76 bp, and in some their end sequences overlap with neighboring *tRNA* or *rRNA*. All *tRNAs* except *tRNA-Ser* (*AGY*) could be folded into the typical clover-leaf secondary structure as determined by tRNAscan-SE. In all groupers *tRNA-Ser* (*AGY*) was found to lack the entire dihydrouridine arm, reducing its secondary structure to a 'truncated cloverleaf', resembling that of most metazoans [42].

### Non-coding regions

**Figure 3:**
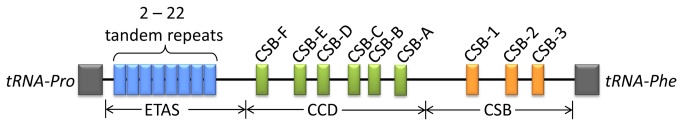
Schematic structures of mitochondrial control regions. ETAS, extended termination associated sequences; CCD, central conserved domain; CBSs, conserved sequence blocks. This figure does not show the structure for the CR of *Cephalopholis argus*.

The non-coding regions in the grouper mtDNA include the CR, the O_L_, and several short intergenic spacers that range in size from 1 to 27 bp. Located between *tRNA-Pro* and *tRNA-Phe*, CR is the largest of these non-coding regions. It is AT rich (average 67.4%) and ranges in size among groupers from 721 to 1264 bp. The grouper CR consists of several termination-associated sequences (TAS) comprising the extended termination-associated sequences (ETAS) region. This is followed by six conserved sequence blocks (CSB-F to CSB-A) making up the central conserved domain (CCD), then three additional conserved sequence blocks (CSB-1 to CSB-3) located downstream from the central conserved domain (Figure 3). However, none of these sequence elements are found in the CR of *C* *. argus* which shares no significant sequence similarity with other grouper species and contains an extra *tRNA-Asp* insertion in the middle of the region.

The repeat-rich ETAS region is the most variable part of the CR, with 2 to 22 tandem repeats depending on the species. This region also includes repetitive elements that range in length from 15 to 134 bp, each containing a conserved TAS motif TACAT and the reversed complement cTAS motif ATGTA. The TAS motif can pair with the cTAS motif, leading to formation of stable hairpin loops which presumably function as sequence-specific signals for termination of mtDNA replication [43]. Repeats in the ETAS region occur in diverse fish taxa [44–46], and the distribution of number of repeated copies suggests that they are randomly added and deleted. The variation in tandem repeats observed in the ETAS region likely evolved via the process of illegitimate elongation [46] or imprecise termination during the replication of the circular mtDNA, and are probably mediated by the mechanism of slipped-strand mispairing [47].

We aligned the conserved regions of CRs in the 21 grouper species, and identified six conserved sequence blocks (CSB-F to CSB-A) with high sequence similarity to the CSBs of other fishes [48]. While five conserved sequence blocks (CSB-F to CSB-B) in the central conserved domain have been reported in mammalian mt CRs [49], only three (CSB-F to CSB-D) are typically found in teleosts [5,50,51]. Looking at these blocks in more detail, first CSB-F demarcates the boundary of ETAS region and central conserved domain. Although this has little sequence similarity with the mammalian CSB-F, both are equivalently positioned within the mt genome CR. Next, the grouper CSB-E has the identifying GTGGG-box of teleost CSB-E [50]. Both CSB-F and CSB-E share 85% sequence identity among these groupers. The CSB-D has a critical role in maintaining the proper regulatory functions of CR and is recognized as the most universally conserved CR segment among teleost fishes [50], which was also seen among the groupers (95% sequence identity). The remaining three CSBs, CSB-C, CSB-B, and CSB-A, are the least conserved (81%–84%), although they can still be identified by the consensus sequences.

We determined the consensus sequences of the six CSBs in the central conserved domain of grouper CR (Figure S1), which are:

CSB-F, GCACAGTAAGAACCTACCAA;
CSB-E, GACAATAATTGTGGGGGT;
CSB-D, TATTCCTGGCATTTGGTTCCTACTTCAGGGCCA;
CSB-C, CTTTCATTGACGCTTGCATAAGTTAATG;
CSB-B, CATTCGACTCGTTACCCA;
CSB-A, TCCAGAGGGTAGGGGGTT.

In addition, we also identified three conserved sequence blocks downstream from the central conserved domain of grouper CR (Figure S1 and Table 4). Comparing these to available CSB sequences from representative teleost fishes (Table 4) we identified the following key features: CSB-1 has an A/T rich region followed by a conserved GATACA motif. Unlike those of higher vertebrates, teleost CSB-2 is the most conserved among the three downstream CSBs and is characterized by a poly-C stretch separated by TA or TTA. CSB-3 contains a sequence of three or four As followed by a poly-C stretch. These CSBs are thought to be involved in the formation of proper RNA primer for mtDNA replication and play a role in the switch from RNA to DNA synthesis that commences at O_H_ [52,53].

**Table 4:** Conserved sequence blocks in the mitochondrial control region of some teleost representatives.

Species	CSB-1	CSB-2	CSB-3	reference
three *Branchiostegus* species	TT-CTTAATGCATACTCTTATTGA-GGTG	A-AAACCCCCCTACCCCC	GAAAACCCCCC-G-AAACA	[68]
*Chanos* *chanos*	NA	AAACCCCCCCCTCCCCCCA	TGTTAAACCCCCAAAACCA	[69]
*Collichthysniveatus*	AT-TACTGTATTTTAGTGCATAA	TAGACCCCCCTACCCCCC	T-AA-C-CCTAAAAACA	[70]
*Crossostoma* *lacustre*	CTATGTATGTAGAATGAGCATAA	ACAAACCCCCCTACCCCCCT	TGCTCAAACCCCGAAACCA	[71]
Cyprinids (different ploidy level)	ATT-AATTAATG-T-GCAGGACA-TA	CAAA-CCCCCCTACCCCC	TGTCAAACCCGAAACCA	[51]
*Cyprinella* *spiloptera*	AGGTTAATGATTATAAGACATAA	CAAACCCCCTTACCCCC	TGTCAAACCCCGAAAGCA	[72]
*Dascyllus* *trimaculatus*	CACTACTGTCTTCCCGGACATACA	TAAAACCCCCCTACCCCCCT	TGAAACCCCCCGAAAACA	[73]
*Dicentrarchus* *labrax*	TTTATCGTAAGTGACATAGGTAA	AATTTCCCCCCCTACCCCCCC	TGCTCAAAATAAATCCCCCTAAGAAAAAGC	[74]
*Larimichthys* *crocea* *, * *L* *. polyactis*	ATTT-AAGTATTC-AGTGCATTA	TAGACCCCCCTACCCCCC	TAA--CCC-TAAAAACA	[75]
*Oryzias latipes*	ATACAGACCTTGTTGACAAG	CAAACCCCCCTACCCCC	TGCAAACCCCCCGGAAACA	[76]
*Pampus sp., * *Trichiurus* *japonicas*	ATAACTGATATCAAGAGCATAA	TA--CCCCCCTACCCCCC	T--AAACCC-----AAACA	[77]
*Pleuragramma* *antarcticum*	TTCTGGGGCATAA	AACCCCCCCCCACCCCC	NA	[12]
*Rastrelliger* *brachysoma*	ATATAAGGATATCATGAGCATAA	TAAACCCCCCTACCCCC	TGCAAACCCCCCGGAAA	[78]
*Rivulus* *marmoratus*	ATAACTGATATCACGGGCATATC	TAGACCCCCCTACCCCCCT	CATTACAAACTTAAACCA	[79]
*Scomber* *colias* , *S.* japonicas	ACATTTTCCTCGCATAA	GTCAAACCCCCCCACCCCCC	CTGCAAACCCCCCGGGAAACA	[80]
22 groupers in family Epinephelidae	CATAACTGATTTCAAGAACATAA	TAAACCCCCCTACCCCCC	TGTAAACCCCCCGGAAACA	this study

Dashed lines indicate the unconserved nucleotides, i.e. substitutions, insertions, or deletions.

The O_L_ non-coding region in groupers is formed on the H-strand located in a cluster of five tRNA genes (Trp, W; Ala, A; Asn, N; Cys, C; Tyr, Y), the so called WANCY region. The O_L_ region region is a short stretch of 33 to 43 nucleotides with a predicted stable stem-loop secondary structure featuring a GC-rich stem and T-rich loop. It is priming of this latter poly-T site that is thought to initiate L-strand synthesis [54]. Groupers share two common features of the vertebrate O_L_, a pyrimidine-rich 5’ flanking region of the stem, and a conserved motif (5’-GCCGG-3’) found at the base of the stem within the *tRNA-Cys*. Both may be associated with accuracy and efficiency of DNA replication at O_L_ as suggested by study of the human mt genome [54].

### Sequence variation and evolutionary rates of different grouper mt genome regions

While the mt genome as a whole generally evolves faster than the nuclear genome, the extent of interspecific sequence variation differs among mt genes. To compare the sequence variability of different regions in grouper mt genomes we calculated the mean of the pairwise sequence identities for each mt gene type and non-coding region among the 22 species. Overall, the most conserved gene types are *rRNA* (89.2±4.2%) and *tRNA* (88.4±3.8%), protein-coding genes present moderate variation (82.6±3.7%), and as expected, the non-coding CR (59.1±9.1%) are the most divergent (P < 0.01).

**Figure 4:**
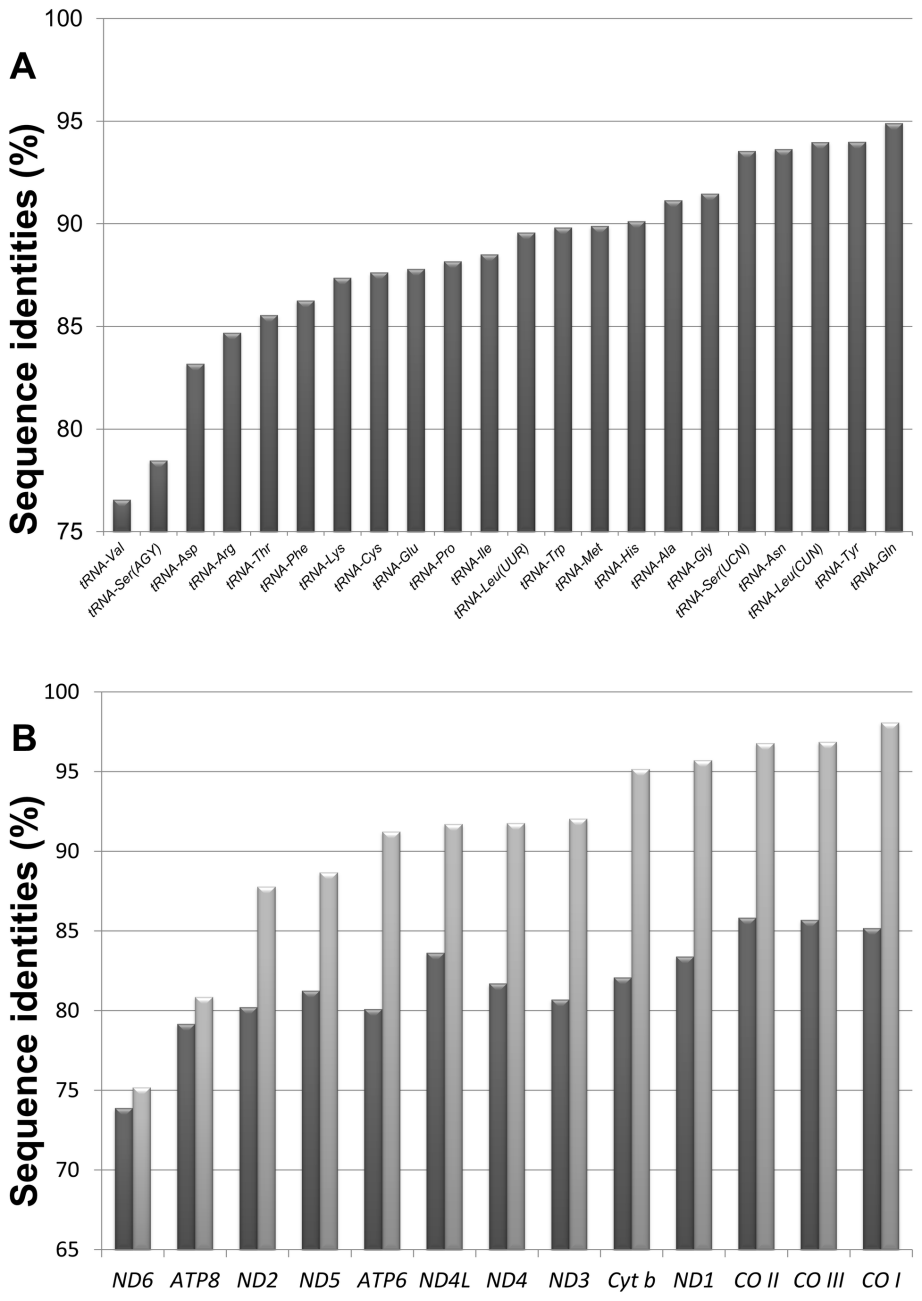
Sequence variations among mitochondrial genes. Genes were ranked by their sequence identity percentages from low to high (left to right). (A) Sequence identities of 22 tRNA genes. (B) NT% (dark grey) and deduced AA% (light grey) of 13 protein-coding genes. Genes were ranked by the AA%.

Except for *tRNA-Val* and *tRNA-Ser* (*AGY*)*, rRNAs* and the remaining *tRNAs* show high levels of sequence similarity (sequence identity > 83%) among the investigated species (Figure 4A). The conservation of rRNAs and tRNAs is presumably required for the formation of their secondary structures and tertiary interactions. This functional constraint would explain their high sequence identities despite the high mutation rate in mtDNA. The metazoan *tRNA-Ser* (*AGY*) generally exhibits considerable sequence variability [42], which may be explained by taking into account that its dihydrouridine arm is replaced by a variable loop, likely weakening structural constraints.

Sequence identity comparisons of both nucleotides (NT %) and their deduced amino acids (AA %) (Figure 4B) from the 13 protein-coding genes show these to be less strictly conserved than the *rRNA* and *tRNA* sequences. Divergence varied among these genes, with the three cytochrome oxidase subunits, COI, II, and III, being the most conserved (AA > 96%, NT > 85%), while NADH dehydrogenase subunit 6 (ND6) of complex I and ATP synthase subunit 8 (ATP8) are the most divergent (AA< 81%, NT < 80%). The difference in divergence among mt protein-coding genes can be explained by the presence of all the functional groups of complex IV in the mt electron transport chain, including COI, II, and III, are mitochondrially encoded, whereas most of the complex I and ATP synthase subunits are instead encoded by nuclear genes [27,55].

Looking at the divergence rates of these genes allows us to compare their utility for DNA barcoding. Though *COI* is commonly used for DNA barcoding [56], the low divergence rate seen in this gene among groupers suggests it may not be able to clearly distinguish between closely related species. As the optimal mt genes for DNA barcoding can vary between taxonomic groups, our analysis of pairwise sequence identities of the 13 protein-coding grouper mt genes leads us to suggest *ND2* as a better candidate for barcoding identification in groupers. *ND2* has a higher percentage of variable sites (52.2% among 22 grouper species) than *COI* (36.9%) and can therefore better discriminate between newly derived species. Additionally, the smaller size of *ND2* (1046 bp) compared to *COI* (1551 bp) makes it easier to completely sequence using only a pair of primers. Thus, a complete gene instead of a partial gene can be used for barcoding.

**Figure 5:**
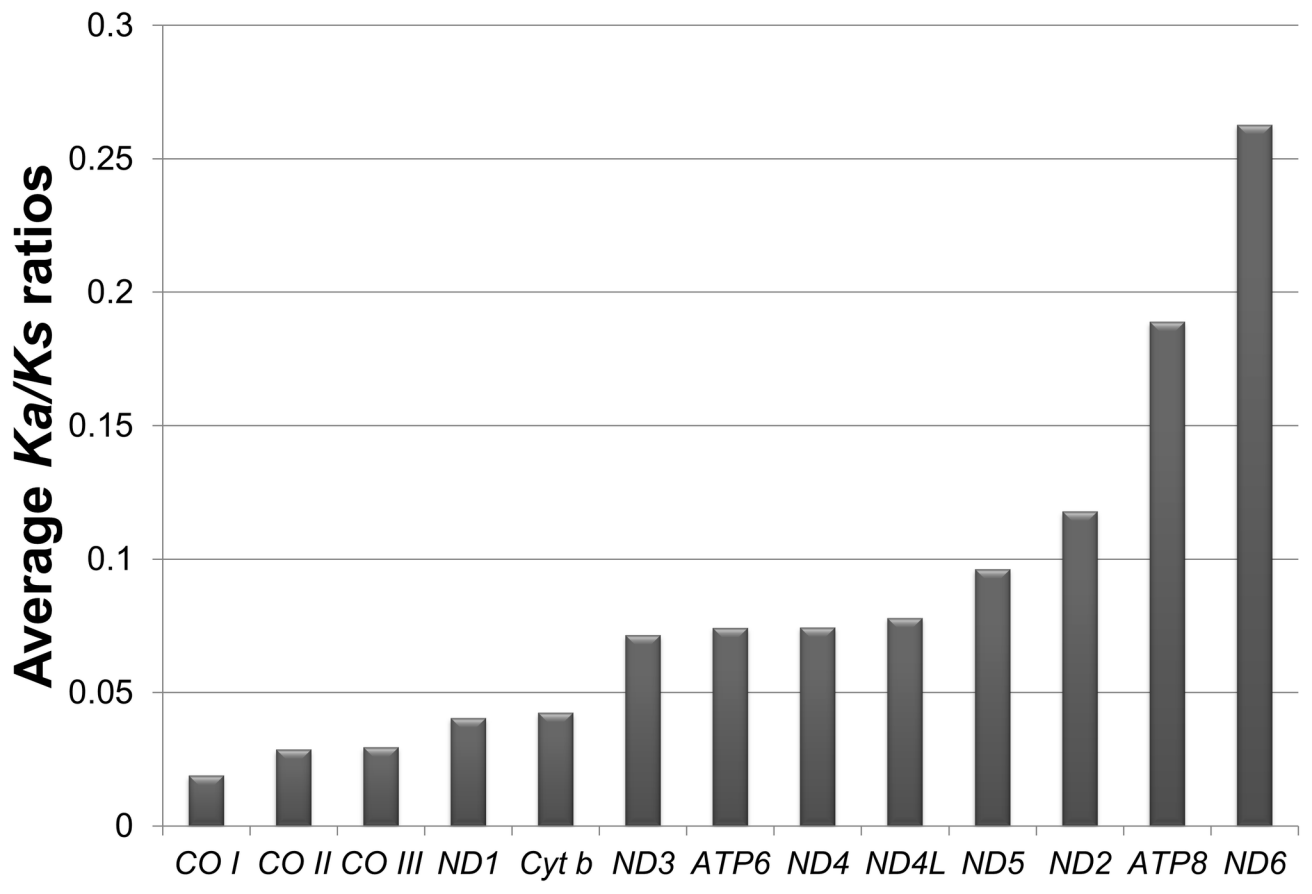
Average *Ka* /*Ks* ratios of 13 protein-coding genes. *Ka* /*Ks* is the ratio of non-synonymous substitutions rate (*Ka*) to synonymous substitutions substitution rate (*Ks*).

In order to analyze the level of selective pressures imposed on different protein-coding genes, we calculated the ratio of the rate of non-synonymous substitutions (*Ka*) to the rate of synonymous substitutions (*Ks*) (Figure 5). Our results show that all 13 genes have the pairwise comparative *Ka*/*Ks* ratios smaller than 1, indicating a signal of strong purifying selection against deleterious mutations on all mt protein genes in these groupers. However, the average *Ka*/*Ks* ratio (0.019 to 0.26) differs substantially among individual genes, suggesting varying functional constraints. Among these genes the ratio is highest in *ND6* (0.26), implying this is under the least purifying selective pressure.

### Phylogenetic analyses

Recent phylogenetic analyses based on several mitochondrial and nuclear genes have challenged traditional views of grouper systematics [19,20]. To test these revised classifications on the mt genome level, we performed partition Bayesian and maximum likelihood (ML) analyses using the concatenated nucleotide sequences (10,963 bp) of the 12 H-strand coded mt protein genes. We excluded the ND6 gene as its rapidly evolving sequence may have led to multiple substitutions at some sites and such homoplasy would reduce the resolution of our phylogenetic analysis.

Both phylogenetic analyses yielded congruent tree topologies with strong support on all nodes of concern (Figure 2). They uniformly agreed on the monophyly of Epinephelinae with a bootstrap value of 100% on the ML tree and a posterior probability of 1 on the Bayesian tree. While groupers were traditionally considered as the subfamily Epinephelinae belonging to the family Serranidae, a large-scale phylogenetic study of percomorph assemblages suggested elevating the monophyletic grouper species (traditional Epinephelinae) to familial status as they were not allied with the remaining members of the polyphyletic Serranidae [20]. In our study, the Serranidae species *Hypoplectrus* *gemma* clusters together with the family Percidae and they together comprise a sister clade to the grouper species with high support values (100% for Baysian and 81% for ML) (Figure 2), lending further corroboration to the elevation of subfamily Epinephelinae to family Epinephelidae.

In terms of the relationships within the family Epinephelidae, our analyses provide high support (100% for both methods) for the initial divergence of genus *Plectropomus* (Figure 2). Although only two species are available from the genus *Plectropomus* , the monophyletic nature of this group [19] allows us to use these species to draw inference for the entire genus. Therefore, our study is indicative of a basal evolutionary status of *Plectropomus* in the Epinephelidae lineage in agreement with previous studies of molecular and morphological phylogeny [19,34,35]. Craig and Hastings (2007) [19] proposed a taxonomic change placing a clade of *Epinephelus* species into a new genus designated *Hyporthodus* based on a set of distinct morphologically characters. This is corroborated by our phylogenetic analyses (100% support for both methods) with the two representatives of the *Hyporthodus* genus, *H* *. septemfasciatus* and *H* *. octofasciatus* , branching from the remaining *Epinephelus* species and grouping with *T* *. dermopterus* (Figure 2). Furthermore, our resulting trees show that two monotypic genera *Anyperodon* and *Cromileptus* ( *A* *. leucogrammicus* and *C* *. altivelis* ) are deeply nested within the clade of the genus *Epinephelus* , and another monotypic genus *Aethaloperca* ( *A* *. rogaa* ) grouped with the species of genus *Cephalopholis* (Figure 2), which is consistent with prior studies using different genetic markers [19,57]. Although these monotypic genera have their unique morphological features, such derived traits are phylogenetically uninformative for tracing the evolution along a lineage. Our results agree with the taxonomic revision to synonymize *Anyperodon* and *Cromileptus* within the genus *Epinephelus* , and *Aethaloperca* within genus *Cephalopholis* , reflecting the monophyletic taxon [19].

Short Mt gene fragments exhibit limitations in resolving complicated phylogenetic relationships in many fish lineages [17]. The added informative sites from longer DNA sequences allow these deeper branches and higher-level relationships to be more fully resolved [18]. Accordingly, more genetic data (e.g. mt genomes) along with extensive taxon sampling is necessary to fully elucidate the phylogenetic relationships among groupers and their higher-level affinities to other percomorph lineages.

## Conclusion

We characterized and compared the complete mt genomes of 22 groupers including 17 newly sequenced species. In studying the evolution of their mt genomes we identified three distinct genome organizations, two of which have never previously been reported. These include the addition of a second *tRNA-Ile* in genus *Variola* and a second *tRNA-Asp* in *C* *. argus* .

Looking at the constituents of the mt genome in more detail, we identified a lineage-specific modification of the *ATP6* start codon to CTG or TTG in the more derived epinephelid clade. Examining the non-coding mt genome CR, all species outside *C* *. argus* contained six conserved sequence blocks rather than the three typically found in teleosts. Comparison of the divergence rates among constituents of the mt genome identified *rRNA* and *tRNA* as the most conserved gene types, followed by protein-coding genes, and the non-coding O_L_ and CR as the most divergent. Among the protein-coding genes divergence rates varied with *COI*, *II*, and *III* being the most conserved while *ND6* and *ATP8* are the most divergent.

Applying these new mt genomic sequences to test recent suggested taxonomic reorganization of the groupers, our phylogenetic analyses corroborated the rearrangement of grouper lineages. The results of this work, besides offering insight into the evolution of the grouper mt genome, provides important resources for the further study of grouper species identification, conservation genetics, speciation and other evolutionary biology studies.

## Materials and Methods

### Sample collection, PCR ampliﬁcation, and sequencing

Specimens of 17 grouper species were obtained from local fish markets in coastal areas of mainland China and Taiwan. Species identifications were performed according to FAO Groupers of the World [1,21] and tissue samples (mostly dorsal muscle) were collected. All experiments were conducted in accordance with the guidelines approved by the Institutional Animal Care and Use Committee at the Xiamen University. Total genomic DNA was then isolated using standard phenol–chloroform extraction and ethanol precipitation methods. Contiguous and overlapping segments of the grouper mt genomes were amplified using a set of 16 primer pairs and then sequenced. The primers, PCR conditions, and sequencing reactions were based on the previously described methods in Zhuang et al. [58] with slight modifications.

### Mitochondrial genome sequence assembly and gene annotation

Sequencing results were first manually proofread and edited using ChromasPro v.1.42 (Technelysium) then the 17 mt genomes were assembled using Sequencher v.4.7 (Gene Codes Corp.). These complete mt genome sequences have been deposited in GenBank under the Accession numbers listed in Figure 2 and Table S1. Protein-coding and rRNA genes were identified by BLAST searches (http://www.blast.ncbi.nlm.nih.gov/Blast.cgi) and annotated based on alignments with the mt genomes of closely related species found in the GenBank database. Most of the tRNA genes and their secondary structures could be predicted with tRNAscan-SE [59]. However, both the tRNA-Ser (AGY) gene and *V* *. louti* ’s extra copy of tRNA-Ile gene instead were identiﬁed by inspecting anti-codon sequences and their tRNA-like secondary structure because they cannot form perfect clover-leaf structure and therefore were not detected by the computer program. These sequencing and annotation results were then combined to map the organization of each species’ mt genome (Figure 1).

### Sequence analyses

Mt genomes were analyzed for 22 grouper species, the 17 sequenced in this study, and 5 species available from GenBank (Accession numbers in Figure 2 and Table S1). Nucleotide compositions were calculated using the program DNASTAR (DNASTAR Inc.) while AT and GC-skew were each computed using the formulas (A-T)/ (A+T) and (G-C)/ (G+C) [38]. Tandem Repeat Finder was used to identify the repetitive sequences in the control region [60]. Pairwise sequence identities for entire mt genomes and each gene type and *Ka/Ks* ratios for protein-coding genes were calculated using the program MEGA 5.0 [61]. We tested for significant differences in sequence variability of different regions using a one-way analysis of variance (ANOVA).

### Phylogenetic analyses

At the time of this study, complete mt genome sequences were available in GenBank from representatives of 27 families in the suborder Percoidei. We utilized all available species from the families Epinephelidae, Percidae and Serranidae, and selected one species from each remaining family in our phylogenetic analyses. In total, 51 Percoidei species (Figure 2) and the outgroup species *Pseudolabrus* *sieboldi* (Labrodei, Labridae; GenBank Accession Number: AP006019) were included.

The concatenated sequences of 12 protein-coding genes on mt H-stand were aligned with codon constraint using CLUSTAL X [62] with minor manual adjustments (Figure S2). Individual gene sequences were also aligned and jModelTest was used to infer the best evolution model of nucleotide substitution [63]. Likelihood ratio tests were conducted to compare different models and the best selected based on both Akaike information criterion (AIC) and Bayesian information criterion (BIC). Among the 12 genes, GTR+I+G was selected as the best model for *ND1*, *ND2*, *ND4*, *ND4L*, *ND5*, *ATP6*, and *ATP8*, TVM+I+G was selected for *COI*, *COIII*, and Cyt*b*, and TrN+I+G was selected for *COII* and *ND3*. The 12 genes were split into three model-defined partitions which were used in the following phylogenetic analyses.

A partitioned Bayesian phylogenetic analysis was performed using MrBayes v. 3.1.2 [64]. Two independent analyses were run with Markov chain Monte Carlo (MCMC) sampling with four chains. Trees and parameters were sampled every 250 generations over a total of three million generations, with the first 10% of the samples discarded as burn-in. Acceptable convergence to the stationary distribution was checked using Tracer 1.4.1 [65]. A majority consensus tree with Bayesian posterior probabilities (BPP) was computed. Partitioned maximum likelihood phylogenetic analysis was performed with GARLI v.0.951 [66]. Each analysis was run with 100 replicates using a random starting tree. Search replicates were evaluated by likelihood score, and only that with the best score was retained. Node supports of the best tree were evaluated with 1000 bootstrap replications, which were then processed by Sumtree [67] to generate a majority consensus tree with bootstrap values. 

## Supporting Information

Figure S1 **Sequence alignment of the central conserved domains and the conserved sequence blocks in the mitochondrial control regions.** Dashed lines indicate gaps introduced by the alignment. Asterisks indicate nucleotide identity in the column. The conserved sequence blocks are shown in red and boxed. This excludes the grouper species *Cephalopholis* *argus* .
(PDF)

Figure S2 **Sequence alignment of the 12 mitochondrial protein-coding genes (excluding *ND6*) used for phylogenetic analyses.** Multiple sequence alignment was generated using CLUSTAL X [62] with minor manual adjustments. The manual modifications are highlighted in yellow.
(DOCX)

Table S1 **GenBank accession numbers for mitochondrial genome sequences of the Percoidei species used in phylogenetic analysis.**
(DOCX)
