# Supplementary material for: A Comprehensive Description and Evolutionary Analysis of 22 Grouper (Perciformes, Epinephelidae) Mitochondrial Genomes with Emphasis on Two Novel Genome Organizations
Source: PLoS One. 2013 Aug 9;8(8):e73561. doi: 10.1371/journal.pone.0073561 (PMC3739747; doi:10.1371/journal.pone.0073561)
Supplement: Figure S1 — Sequence alignment of the central conserved domains and the conserved sequence blocks in the mitochondrial control regions. Dashed lines indicate gaps introduced by the alignment. Asterisks indicate nucleotide identity in the column. The conserved sequence blocks are shown in red and boxed. This excludes the grouper species Cephalopholis argus . (PDF) [file pone.0073561.s001.pdf]

CSB-F

```
E. bruneus          ATAGTAGAATAGACTGCACAGTAAGAACCTACCAACTAGACTTAAGTAA-----TGCATA
E. moara            ATAACA-AATAAACTGCACAGTAAGAACCTACCAACAAGAGTTAAGTAA-----TGCATA
C. sonnerati        TTCTTCAAATAGAATGCACAGTAAGAACCTACCATCAGTTGATATCTCA----ATGCCCA
E. epistictus       A--ATCAAATAGCATGCACAGTAAGAACCGACCAATAAGTTATTTCTTA----ATGAACT
E. fuscoguttatus    G--AACAAATAGCCTGCACAGTAAGAACCTACCAACCAGTAACAGGCTA----ATGCATA
A. leucogrammicus   A--AACAAATAGAATGCGCAGTAAGAACCTACCAACCAGCTGTAAGA-A----GCGCATA
E. awoara           AAATTAAACAGCATTGCACAGTAAGAACCGACCAACAGTTGATTACTTA----ATGCATG
E. akaara           ATATCAAACAATCATGCACAGTAAGAACCTAGCAATAATTTATTACTTA----ATGCATG
V. albimarginata    TATCTCAGTAACTGGACGCAGTAAGAACCTACCAACCTGTGATTTCTGA----ATGCATC
V. louti            AAACCCAACAACCAGAAGCAGTAAGAACCTACCAACTTATGATTTCTTA----ATGCATC
C. altivelis        AGGACAAATAAA-ATGCACAGTAAGAACCTACCTATAAGTGATATCTTA----ATGCATG
E. areolatus        ACTCAAAATA-TTATGCACAGTAAGAACCGACCAACCAATAGTTCCA------GCGCATA
T. dermopterus      CAATTAAATA-TAATATACAGTAAGAACCTACCAACCAGTGATTTCTAAAATCGTGCATA
E. coioidus         TAATTGTAATGATTTGCGCAGTAAGAACCTACCAACTAGTA-----TAAAGTAATGCATA
A. rogaa            T-----GATTG--ATCCGCAATAATAAGGTTATAAATTGCAGC-------------CATT
E. lanceolatus      A-----AACTATAATGCGCAGTAAGAACCTACCAACCAGCACCATGACAAA--GCATACG
E. trimaculatus     C--TTCAAATATAATGCACAGTAAGAACCGACCAATTATTGATAACTTA----ATGCAAG
P. leopardus        AA-----ACCAAAGTATGCAGTAAGAACCGAGCAACAAATGATTTCTTA----AGGCTAA
P. areolatus        AG-----AATTAACTATGCAGTAAGAACCGACCTACAGGTGATATCTAA----ATCCTAA
E. octofasciatus    AAT--CAAATAGATTGCACAGTAAGAACCGATCAACGGATGATTTCTTA----ATACATA
E. septemfasciatus  AAT--CAAATAGACTGCACAGTAAGAACCGACCAACAAATGATTTCTTA----ATACATA
                                       ****** **
```

CSB-E

```
E. bruneus          CGGTT-ATTGATAGTGAGGGACAATAATT-GTGGGGTTTCACTTATTGAACTATTCCTG
E. moara            CGGTT-ATTGATAATGAGGGACAATAACT-GTGAGGGTCCCACTCAGTGAATTATTCCTG
C. sonnerati        CGGTTAT-TGAAGGTGAGGGACAATTATTTGTGGGGGTTTCACAACTTGAATTATTCCTG
E. epistictus       CTCTTATATGAAGGTGAGGGACAATAATT-GTGAGGGTTTCACTTAGTGAATTATTCCTG
E. fuscoguttatus    CGGTTAT-TGATAATGGCAGACAATAATT-GTGAGGGTCTCACTTAGTGAATTATTCCTG
A. leucogrammicus   TTATTAT-TGATAGTGATGGACAATAACT-GTGGGGGTTTCACTTAGTGAACTATTCCTG
E. awoara           A-ACTTATTGAAGGTGAGGGACAATAATT-GTGAGGGTTTATCATAGTGAACTATTCCTG
E. akaara           A-ACTTATTGAAGGTGAGAGACAATAATT-GTGGGGGTTTCTCATAGTGAATTATTCCTG
V. albimarginata    CGACT-ATTGAAGGTGAGGGACAACTATT-GTGGGGGTTTCACTTAGTGCACTATTCCTG
V. louti            CTACT-ATTGAAGGTGAGAGACAAATACT-GTGGGGGTTTCACTTAGTGCACTATTCCTG
C. altivelis        ATACTTATTGATAATGGCCGACAACAATT-GTGGGGGTTTCACAGTATGAATTATTCCTG
E. areolatus        ATATG-ATTGAAGGTGAGGGACAATAATT-GTGGGGGTTTCACACAGTGATTTATTCCTG
T. dermopterus      CTCTT-ATTGAAAATGATGGACAAAAATC-GTGAGGGTTTCACTTCTTGAATTATTCCTG
E. coioidus          CGGTTATTGATGGTCAAGGACAGTAATT-GTGGGGGTTTCACGTAATGAACTATTCCTG
A. rogaa            T---CAACTGAAAATGTTGG-CAATTAAT-G-AAGGGTTGCTGTAACTTCATTATTCCTG
E. lanceolatus      G---TTATTGATAATGATAGACAATTACT-GTGGGGGTTTCACTTAGTGAACTATTCCTG
E. trimaculatus     A-ACTGATTGAGGGTCAGGGACAAAAATC-GTGGGGGTCCACACAGTGATTTATTCCTG
P. leopardus        CAATT-ATTGAAAATGAGGGGCAATTTCTAGTGGGGGTCGCACA-ATTGCACTATTCCTG
P. areolatus        CTCTT-CTTGAAGGTGAGGGACAATTATTAGTGGGGGTCGCACA-ACTGCATTATTCCTG
E. octofasciatus    CTCTT-ATTGAGGGTGAGGGACAAGAAATTGTGGGGGTTTCACTCAGTGAACTATTCCTG
E. septemfasciatus  CTCTT-ATTGAAGGTGAGGGACAAGATACTGTGAGGGTTACACTTAATGAATTATTCCTG
                             *    *     * ***     *   ****          *    *********
```

CSB-D

```
E. bruneus          GCATTTGGTTCCTACTTCAGGGCCATAAC--CTGATAACATTCCTCACACTT-TCATTGA
E. moara            GCATTTGGTTCTTACTTCAGGGCCATGAC--TTGATTATATTCCTCACACTT-TCATTAA
C. sonnerati        GCATTTGGTTCCTACTTCAGGTCCACGAA--TTGAT-TTATTCCTTCCACTT-TCATCGA
E. epistictus       GCATTTGGTTCCTACTTCAGGAACATAAA--TTGATATTACTCCTCATACTT-TCATTGA
E. fuscoguttatus    GCATTTGGTTCCTATTTCAGGGCCATAAA--TTGGC--AACTCCTCATTTATATTATTTA
A. leucogrammicus   GCATTTGGTTCCTACTTCAGGGCCATATA--TAGAC-ATATTCCTCACACTT-TCATTGA
E. awoara           GCATTTGGTTCCTACTTCAGGGCCATAAA--TATCAACATTTCCCCATGAAT-TGATCGA
E. akaara           GCATTTGGTTCCTATTTCAGGGCCATAAA--T-CTAATTTCTCCCCACAGAT-TTATTGA
V. albimarginata    GCATTTGGTTCCTACTTCAGGGCCATTAA--TTGATAATCGTCCACATTCTT-TCATCGA
V. louti            GCATTTGGTTCTTACTTCAGGGCCATGAA--TTGATT-TCATCCTCATTCTT-TC-TTGA
C. altivelis        GCATTTGGTTCCTATTTCAGGGCCATGGA--ATGAT--TTAACCTCTATATT-TTATTGA
E. areolatus        GCATTTGGTTCCTATTTCAGGGCCATAAA--CTGACAAT-TTCCCCACACAT-TTATTGA
T. dermopterus      GCATTTGGTTCCTATTTCAGGGCCATAAA--TTGATATTACTCCTCATACTT-CCATTGA
E. coioidus         GCATTTGGTTCCTACTTCAGGGCCATGTA--TCGAAAT-ATTTCACACACTT-TCATTGG
A. rogaa            GCATTTGGTTCCTAAGTCAGGTCCATTAAAGTTAATGG-ACCTCTATAAAGT-TAGTTTG
E. lanceolatus      GCATTTGGTTCCTACTTCAGGGTCAAAAA--TTGATATTATTCCTCACACTT-TCATTGA
E. trimaculatus     GCATTTGGTTCCTACTTCAGGGTCATAAAA-TATTCTTGATTCCTCATAATACCTATCGA
P. leopardus        ACATCTGGTTCCTATTTCAGGGCCATAAGCATTAATA--A-TACTCTTACTT-TCATCGA
P. areolatus        ACATCTGGTTCCTACTTCAGGTCCATGATAATTCATA--ACTCCCCCCACTT-TCATTGA
E. octofasciatus    GCATTTGGTTCCTACTTCAGGGCCATGACTTGATATT--ATTCCTTACACTT-TCATCGA
E. septemfasciatus  GCATTTGGTTCCTACTTCAGGGCCATAAATTGATATT--ACTCCTCACACTT-TCATTAA
                     *** ****** **  *****  **                  *            *
```

```
                        CSB-C                                   CSB-B
E. bruneus              CGCTG--ACATAAGTTAATGATGTTAAC--CATTAGA-TTCGTTA--CCCAGCAAGCCGG
E. moara                CGCTG--GCATAAGTTAATAGTGTTAAC--CATTAGG-TTCATTA--CCCAGCAAGCCGA
C. sonnerati            CGCT--TGCATAAGTTAATGGTGGTAAT--CATTCGA-CTCGTTA--CCCACCAAGCCGA
E. epistictus           CGCT--TACATAAGTTGATGGTGGTAAT--CATTTGA-CTCGTTA--CCCAGCAAGCCGA
E. fuscoguttatus        CACT--TGCATAAGTTAATAGTGTCGAC--CATTAAAACTCGTTA--CCCAACAAGCCGA
A. leucogrammicus       CGCT--TACATAAGTTAATGGTGTCGAT--CATTAGA-CTCGTTA--CCCACCAAGCCGA
E. awoara               CGCT--TGCATAAGTTAATGGTGGTAAT--CATTCGA-CTCGTTA--CCCAGCAAGCCGA
E. akaara               CGCT--TGCATAAGTTAATGGTGGAAAT--CATTTAA-CTCGTTA--CCCAGCAAGCCGA
V. albimarginata        CGCT--TGCATAAGTTAATGTTGTTAAT--CATACGA-CTCGTTA--CCCAGCTAGCCGG
V. louti                CGCT--TGCATAAGTTAATGTTGTTAAT--CATACGA-CTCGTTA--CCCAACTAGCCGG
C. altivelis            AACT---GCATAAGTTAATGCTGTTAAC--CATTCAA-CTCGTTA--CCCAGCAAGCCGA
E. areolatus            CGCT--TGCATAAGTTAATGGTGTTAAA--CATACGA-CTCGTTA--CCCAGCAAGCCGG
T. dermopterus          CGCT--TGCATAAGTTAATGGTGTTAAC--CATGAAA-TCCGTTA--CCCAACAAGCCGA
E. coioidus             CCCT--TGCATAAGTTAATGCTGTTAAA--CATCAAA-CCCGTTA--CCCAACATGCCGA
A. rogaa                CAAT--TGCATAGGTTAATGGTGGCAAC--CATTCAG-TTTAATAAATTTGGCAAGC-AA
E. lanceolatus          CCCTGGTGCATAAGTTAATAGTGTTAACGGCATTAAA-CTCGTTA--CCCAGCAAGCCGA
E. trimaculatus         CGCT--TGCATAAGTGAATGGTGATAAT--AATATGA-TTCGTTA--CCCAACAAGCCGA
P. leopardus            CGCTGG--CATAAGTTAATGGCGGCATA--CAATCGA-CTCGTTA--CCCACCTAGC-GA
P. areolatus            CGCTGG--CATAAGTTAATGGTGGTGTA--CAATCGA-CTCGTTA--CCCACCTAGC-AA
E. octofasciatus        CGCT--TGCATAAGTTAATGGTGTCAT---CATTCGA-CTCGTTA--CCCAGCAAGCCGA
E. septemfasciatus      CGCT--TACATAAGTTAATAGTGTTAAT--CATTTAA-TTCATTA--CTCAACAAGCCGA
                           *    **** **  **   *        *           **       *  **

                                        CSB-A
E. bruneus              GCATTCACTCCAGAGGGTGGGGGGTTCTCTTTTATTTCTTTTC---CTTTCAACAGACAC
E. moara                GCCTTCATTCCAGAGGGTGGGGGGTTCCCTTTTATTT-TTTTC---CTTTCAACAGGCAT
C. sonnerati            GCGTTCACTCCAGCGGGTAAGGGGTTCC--TTTTTTCTTTTTT--CCTTTCAATAGACAT
E. epistictus           GCGTTCATTCCAGAGTGTGGGGGGTTTC--TTTTTTCTTTTT---CCTTTCAATAGACAT
E. fuscoguttatus        GCCTTCATTCCAGAGGGTGGGGGGTAAT--CTCTATTTTTTTC--CTTTTCAATAGGCAT
A. leucogrammicus       GCGTTCACTCCAGAGGATGGGGGGTTTT--CTCTATTTTTTTT--CCTTTCAATAGACAT
E. awoara               GCGTTCATTCCAGCGGGTAGGGGGTTTT--TTTTTTCCTTTTT--CCTTTCA-TAACCAT
E. akaara               GCATTCATTCCAGAGTGTAAGGGGTTTT--CTTTTCTCTTTTT--CCTTTCA-TAACCAT
V. albimarginata        GCATTCTTTCCAGAGTGTAAGGGGTTTT-CCTTTTTTCTTTTT--CCTTTCAACTGGCAT
V. louti                GCATTCTTTCCAGAGTGTAAGGGGTTTTTCCTTTTCTCTTCTT--CCTTTCCTCTGGCAT
C. altivelis            GCACTCTTTCCAGAGGGTGGGGGGTTTC-TCTTATTTTTTTTT--CCTTTCAATAGACTT
E. areolatus            GCGTTCATTCCAGAGTGTAAGGGGTATT---TTTTTCTCTTTT--TCCTTTCGTGAGCAT
T. dermopterus          GCATTTATTCCAGAGGGTGGGGGGTTTC---TTCTTTTTTTTT--CCTTTCAACAGACAT
E. coioidus             GCATTCATTCCAGAGGATAGGGGGTTGGATTTTTCTTTTTTC----CTTTCGACAGGCAT
A. rogaa                GCATTTACTCCGAAGGGGCAAGTGTTAATTTATTTTTTCTTTGGGGTTTTCAGCAACTAT
E. lanceolatus          GCGTTTCATCCAGAGGGTAGGGGGTTGAATCTTTTTTTTTTTTTCCTTTTTCATTGACAT
E. trimaculatus         GCGTTTACTCCAGAGTGTAGGGGTTTCC---TCTTTTATTTTTATCCTTTCAACAGACAT
P. leopardus            GCGTTCACTCCAGAAGGCGGAGGGTTGTCTTTCTTCTTTTTCC---TTTTCCCTT-ACAT
P. areolatus            GCATTCTTTCAAGAGGACAGAGGGTT-CCTTTTTTCTTTTTCC---TTTTCTCTT-ACAT
E. octofasciatus        GCGTTTATTCCAGAGGGTAGGGGGTTTTTCTTTTTTTTTTTCC---TTTTCAATGGACAT
E. septemfasciatus      GCGTTTATTCCAGAGGGTAGGGGGTT-TTTCTTTTTTTCCTCT---ATTTCATTAGACAT
                        ** *    **           *  *                       **

E. bruneus              TTCAGAGTG---TAAGAAACGCT--AATGATTGTAGGTGGGACAATTACTGCTCTGTA--
E. moara                TTCAGAGTG---TAAGAAAAGGCTGAAAGTTTGAAGGTGGTACA-TCACTTTGCAGCT--
C. sonnerati            TTCACAGTG---TATGTAATCT---AAAATAAAAGGTGGA-AC--ACATCCTTTGCC---
E. epistictus           TTCAAAGTG---TAAAAAATCT---AATGAAAAAGGTGGT-ACTTACTTCTATTGTAA--
E. fuscoguttatus        CTCAGAGTG---TAGATAAACTTA-AAGGTTGAGGGTGGT-ATAATTAGGTATTGTT---
A. leucogrammicus       TTCAGAGTG---TAAGCGATCAAATAAAGTTGAAGGTTGT-ACATACTATAACTACTGCA
E. awoara               TTCACAGTG---ATTGAAAAA-AAAAACGAAGAAGGTAGAACTTTCAATAACTTGGTTTT
E. akaara               TTCATAGTG---ATTGGAAAA-AGCAA--ATAAAGGTAGTATTAATCA-AACTTGTTTCA
V. albimarginata        TTCACAGTG---CACACAGT--ATTAACTAATAAGGTTGA-ACATTTAGTCTTTGCCTGC
V. louti                TTCACAGTG---CATACGAC--AATAACAATCAAGGCAGA-ACATTTAGTCCTTGCAGAA
C. altivelis            TTCAGAGTG---TAAGCAATCTAATAATGGCAGTGGTTCATACATTAAATTATGACTTGG
E. areolatus            TTCACAGTG---TTAGAGGCGCTAGAACTCAC-TAAGGTT-GAACATATTCCCTGCTTGC
T. dermopterus          TTCACAGTG---TAAGAGATATAACAATGGATGTGGTATT-AAACTCTCTGTTTTAATGA
E. coioidus             TTCACAGTG-----CAACGGTCTCAAGAAACGAAGGTGGT-ACATAACATATTTCAGTAA
A. rogaa                CTTAAAGTTGTGTTCAGGGGTTAGAACTAATGAAGGTAGT-ATAAGACTTAAATGTATTT
E. lanceolatus          TTCAGAGTGA----ATTTAATTTAAAC--ATGGAAGTGGT-ACAATAGTTTTATGTAATT
E. trimaculatus         TTCACAGTG-----TAAGAAATCAGTTTTATTAAGGTTGT---ACATACACCTTGCTTGT
P. leopardus            TTCACAGTG---TAAGCCGAAATAACAGGAACAAGGTGGT---ATAAACACTTTGGATAC
P. areolatus            CTCACAGTG---TAGGAAAAATTAAAA--AACAAGGTGGA---ACAGTATCTTTATGAAA
E. octofasciatus        TTCACAGTG---TAA-AGAATCTAACT--ATAAAGCTGTA---CTCATCTCTGCTCAAAT
E. septemfasciatus      TTCACAGTG---TAAAGAATCATAATA--ATAAAGGTGGT---ACTCATTTTCTGCTCAC
                         * * ***
```

```
E. bruneus          -ATAAATATAAACTATGCAGGCTAGAAGAGCATTCTT--ACAGATAACTACATAAC-TGA
E. moara            -GAACACA-AGGTTATGCAGGCTAAAAAGACATTCTTTTAAAAATAATTACATAAC-TGA
C. sonnerati        -CCGCGTAAATGTTGTGCGTGGTGGAAAGAGTTTAATTTATG--AAT-TCCATAAC-TGA
E. epistictus       -TTGCAGAATAAGTATGAATATTAGAAAGATATTCTTATAAT--AATATACATAAT-TGA
E. fuscoguttatus    -TTAAATAAGGTATATGTATGTTGAAAAGATATTCTTATAAG--AAT-TTCATAAC-TGA
A. leucogrammicus   -TAGCCTATAAAAAATGTATGTTAAAAATATATTACTATAAA--TAGTTACATAAC-TGT
E. awoara           AGAAGAC-ATATGTAACCATG-TTATAAAACATTCTTATAAT--TACTTACATAAG-TGA
E. akaara           AATAAAT-ACGTGTGATTATG-TTAAGAAATATTCTTATA----TATTTCCATAAC-TGA
V. albimarginata    TTGATATGGTGTTTGTAGGTC-TTGAAAGACATTCATAAAA---GACTCCCATACTTTTA
V. louti            ACCGACTAAAACTTGTAA-TC-TTCAAAGATATTAACAGAA---GACTTGCATAAGATTA
C. altivelis        --CAAATGAAATATGTATGT--TGGGAAAATATATTTTACG---ATATTGCATACC-TGA
E. areolatus        AACAAGT-ATGTGTATGT-TA-GAAAGGTATA-TCTTATATT--A--TTACATAAC-TGA
T. dermopterus      ATAAATA-ATGTGTGTAA-TA-CAAGATAATAATCTTACAAT--AAATAACATAAG-TGA
E. coioidus         AAGTGATAGGAAAATG-CATGTTAGAAAGATATTCTT-TAA--TTAGTTTCATAAC-TGA
A. rogaa            TAGTA--AATAAGTTG-AATGGTGGTAAGAATTTCTTATAAATTTAATTGCATGTT-TGA
E. lanceolatus      AACAAACAGTTTAATGTAATGTTAAAAAGATATTACTATAA---TAGTTACATAAC-TGA
E. trimaculatus     TACAAGCGAGGTGTAA---TGTTAGAAAGACATATCTTACTT--TACATACATAAC-TGA
P. leopardus        GAAGCCC-T--TAAATTAACGTTAACTAAACTATAACC-GAA--GAATTACATTAA-ATT
P. areolatus        TAGGTAT-AGGTTCGTTCATGC-AATTAAACTACAATAAGAA--GACATACATAAG-GGA
E. octofasciatus    TATAAAT-TA-TATGTGTGTAA-AAAGATAATCTTATAAAAC--AAATTGCATAAG-TGA
E. septemfasciatus  CAATAAG-AAATATATGTCTGTAAAAAAGATAATCTTATAAAT-AAATTGCATAAG-TGA
                                                                 ***

                    CSB-1
E. bruneus          TTTCAAGGACATAAATATGCCTATTC---TACTCAGAACACCCCCGTAATGT-AGGATAC
E. moara            TTTCAAGAACATAAATATACTTATCC---TACTCAGAGCACCCCCCTAATATTAGGATGA
C. sonnerati        TTTCAAGAGCATAA--TTGATTGATT--TTACTCGAAAGATCTCTAAGAT--ACCCCCGG
E. epistictus       TTTCAAGGACATAA--GTAATTTAATCCTTAACCAAAATATCTCTAAGTTCTACCCCCTT
E. fuscoguttatus    TATCGAGGACATAA--ATAACTATTCCATCAAAC--ATCTCCTATAAGAT--ACCCC---
A. leucogrammicus   TTTCAAGGACATAA--ATACTTATTC---TACTCGAGACATCTCCTATAAAGATACCCC-
E. awoara           TATCAAGAGCATAA--GTAGTTGAT---TTTCTCGAAACATAACTATA-AGAATACCCCG
E. akaara           TATCATGTACATAA--GTAGTTGAT---TTACTCGAAACATAACTATA-AGAATACCCCC
V. albimarginata    TATCTCGAGCATAAA-GTGCCTTCTA--CTACTTAGGAGAT----ATCTAAGATGCCCCT
V. louti            TATCACGAGCATAAT-ACACCTATTA--CTACTTGGAGAGT----ATCTAAGATGCCCCT
C. altivelis        TTTCAAGAACATATA-GCAGTTAGTA--TTGCTCGAAACACCCCCATATAGATCACCCCC
E. areolatus        TTTCAAGGACATAA--ATAATTAATT--TCACTCGAAACATATCTATAAGA--TACCCCC
T. dermopterus      TTTCAAGGACATAA--ATGGTTTATG--ACACTCGAAACTCCTTCATAAGACTTATACCC
E. coioidus         TTTCAAGGGCATAAT---AATTACAT--CTACTCAAAACACC-TCCTGTAAGATCCCCCT
A. rogaa            TTTCGTGAGCATAATTATAACTAACC--CTAGCCGAAGAATA-ACTCATAT-ATTACCCC
E. lanceolatus      TTTCAAGGACATAACATAACTTTATT--CCGCTCGAAACATCCACCTATAAGATCACCCT
E. trimaculatus     TTTCACGAACATAA------GTAATA--TAACTCGAAACATATCTATAAGATATACCCC-
P. leopardus        CTTTGATA-CATAT---TCGTAACAAAATAAATCGAAAA--ATTTGCTTTTAA-CTGAAT
P. areolatus        TTTCGAGAACATAAA--TTATAGCAAAGTAA-TCGAAAA--ATTTAAACTTAA-CTGATG
E. octofasciatus    TTTCAAGAGCATAAA--TGAT--TTATTTTACTCGAAAC--TTCT---CTTA------TA
E. septemfasciatus  TTTCAAGAGCATAAA--TAAT--TTATTTTACTCGAAACTTATCTACAAGTAATACCCCA
                      *       ****

                                                  CSB-2
E. bruneus          CC---CCGGAGGATTATTATCCATTTAAACCCCCC--TACCCCCCTAAACCCCTGAGATT
E. moara            CCG-GTCTGGTGGTTTTTATCCGTTTAAACCCCCC--TACCCCCCTAAACCCCTGAGATT
C. sonnerati        ----------GGGTTTTTTTCTCCTTAAACCCCCC--TACCCCCCTAAACTCCTGAAGTA
E. epistictus       CCACC-----AAGGTTTTTTTTCGTTAAACCCCCC--TACCCCCCTAAACCCTTGAAGTC
E. fuscoguttatus    -----------GGGGTTTTGTTCGTTAAACCCCCC--TACCCCCCTAAACCCCTGAGATC
A. leucogrammicus   -----------GGGGTTTTGTCCGTTAAACCCCCC--TACCCCCCTAAACCCCTAAGATC
E. awoara           ----------GGGGTTTTTTCTCGTTAAACCCCCC--TACCCCCCTAAACCCCTGAGATC
E. akaara           ----------GGGGGTTTTTCTCGTTAAACCCCCC--TACCCCCCTAAACCCCTGAGATC
V. albimarginata    -----------GGTTTTTCTTCGCGTAAACCCCCCCCATACCCCCCCCACTCTTAAAACC
V. louti            ------------GGTTTCCTCGCGTAAAACCCCCCCCAA-CCCCCCCCACTCCTAAAGTT
C. altivelis        ---------GGGGCTTATGTTCGTTTAAACCCCCC--AAACCCCCC--------GAGATC
E. areolatus        ----------GGGGTTTTTTTTCGTTAAACCCCCC--TACCCCCCTAAACCCCTGAGATC
T. dermopterus      -----------CGGAAGTTGTTCGTAAAACCCCCCC-TACCCCCCCAAACCCCTGAGATC
E. coioidus         GGG-------GTTTCGTCGT----TTAAACCCCCC--TACCCCCCTAAACCCCTGAGATC
A. rogaa            CAG-------GTTTTATCCTCC----AAACCCCCC--TACCCCCCTAAACTCCTAAGATC
E. lanceolatus      GGGTGTAAAAA-TTTTTTCGTTCGTTAAACCCCCCC-TACCCCCCCAAACCCCTGAGATT
E. trimaculatus     ------------GGGGTTTTTCCGTTAAACCCCCC--TACCCCCCTAAACCCCTGAGATC
P. leopardus        ATTCTTTGGGAGTTTCTTCGTAGAAGGAACCCCCCC-TCCCCCC--ACACTCCTAAAATT
P. areolatus        ATTCAT--GGATCCCTTCGTAGAAGGGAACCCCCC--CCCCCCCCT-CACTCCTAAAATT
E. octofasciatus    AGATACCCCCGGAATTTATTCG--TTAAACCCCCCC-TACCCCCCTAAACTCGTGAGATC
E. septemfasciatus  CGAATTTTTT-GAATTTTTTCG--TTAAACCCCCC--TACCCCCCTAAACTCCTGAGATC
                                              ********      *****        *    *
```

```
                                                         CSB-3
E. bruneus           CCTAACACCCC-TGTAAACCCCCC--GGAAACAGGGTTAAACCTCAAGTAATAG------
E. moara             CCTAACACCCC-TGTAAACCCCCC--GGAAACAGGGCTAAACCTCAAGTAATAA------
C. sonnerati         TCTAACATTCC-TGTAAACCCCCC-TGGAAACAGG--AAAACCTCAAGCAATAA-----G
E. epistictus        TCTAACACTCC-TGCAAACCCCCC--GGAAACAGG--AAAACCTCAGGTAGTAA-----A
E. fuscoguttatus     CTTAACACCCC-TGCAAACCCCC---GAAAACAGGAATAGACCTCGAGTGGTGA-----A
A. leucogrammicus    CTTAACA-TCC-TGTAAACCCCCC--GGAAACAGGACCAGATCTCGGGTAATAA-----A
E. awoara            CTTAACACTCC-TGTAAACCCCCC--GGAAACAGG-AAGAATCCCAAGTAGT--------
E. akaara            CTTAACACTCC-TGTAAACCCCC---GGAAACAGG-AAGAATCCCAAGTAAT--------
V. albimarginata     TCTACTACTT--TTTATACCC---TT-AAAATAATAACTAACCCTAGGAAATAACCGGCG
V. louti             ACTATTACTT--TTTAA-CCCCC-TTAAAGACTGGG-CTAACCCTAAGAAACGTCTCACA
C. altivelis         TCTAACACTCC-TGCAAACCCCC---GGAAACAGG-ATAAATCTCAAGTAATAA-----G
E. areolatus         CTTAACACTCC-TGTAAACCCCCC--GGAAACAGG-AAAGACCCCAAGTAGTTTCT---G
T. dermopterus       CTTAACACTCC-TGTAAAACCCCCTCGAAAACAGG--AAAACCTCAAGTCGCAAGC---C
E. coioidus          CTTAACAC-CC-TGTAAACCCCCC--GGAAACAGGACTAAACCTCAAGTGGTA------A
A. rogaa             CTTATTATCCT-TGCAAACCCCCCC-GAAAACAAGGGAAGAACCTCAACTAGTTCC---A
E. lanceolatus       CCTAACACTCC-TGTAAACCCCCC--GGAAACAGGACTAAATCTCAAGTGGTA------A
E. trimaculatus      CTTAACACTCC-TGTAAACCCCC---GGAAACAGG-AAGAATCCCAAGTAGTCTCT----
P. leopardus         TCTAAGACTT--TGACAAACCCC-AAAAAAACAG--AAAAACCTCTAGTAGTAAG-----
P. areolatus         TCTAAGCCTC--TGACAGGCTTTC--TAAAACAGG--AAATCCCCTAGTAACAC------
E. octofasciatus     ATTAACAACCCCTGTAAACCCCCC--GGAAACAGG--AAGACCTCAAATAATAAT-----
E. septemfasciatus   ACTAACAACCCCTGTAAGCCCCCC--GGAAACAGG--AAAACCTCAGATAGTAAA-----
                       **        *      *        * *           *

E. bruneus           ATTTTTAACCTAAAATGTG-TTTATTACACTAATGTAA-TTTTT--AATTTG--------
E. moara             GTTTTTAACCTAAAATGTG-TTTATTACACTAATGTAA-TTTTT--AATTTG--------
C. sonnerati         TCTATAGGCCCAAAATGTGCTGATTTAAAATATTATAA-TATTA--AATTT---------
E. epistictus        CTTTTAA-CCTAAAATGCG-TGTATTATATTATTATAG-TATTT--AATTT---------
E. fuscoguttatus     TTTTTAA-TCTAAAATACG-TTTATTACACTAATGTAA-TTTTTTTAATTT---------
A. leucogrammicus    TTTTTAA-TCTAAAATGCG-TTTATTATACTAATGTAA-TTTTT--AACTT---------
E. awoara            TCAAAAAGTAAACTTACTAAATCAAAATGTGTTTATTA-TACT--AATGTAATCTTTAAT
E. akaara            TTAAAAAATAAGATTATTGAATTAAAATGTGTTTATTA-TACT--AATGTAATCTTTAAT
V. albimarginata     TTTTT-AAGTAGACTCTTGCCCCC-CCCGTATTTGTAG-TATTGCAAACTCGCTTTTTCC
V. louti             TTTCTCAAAAAGACTTTTGCCTCCTCCCATATTTGTAG-TATTGCAAACTCACTTTCTCT
C. altivelis         TTTTTGACCTAAAATTTAATTTTTACATTAATGTA-AA-TATT--AAATTT---------
E. areolatus         TAGCAACTTGTAAATTAAAATTCGTTA-ATTATAATAA-TATAATTAATTT---------
T. dermopterus       TATTAACCT--AAAATACACCCCTTTATATTATTATAA-TGT--TTAAATT---------
E. coioidus          ATTCTTAGCCTAAAATACG-TCTATTACACTAATGTAA-TTTTTTTAATTT---------
A. rogaa             ACCATAACCCAAAGATACA-CCTATTATATTATTATAA-TATTTCAACA-----------
E. lanceolatus       ATTTTTAACTTAAAATGCG-TTTATTACATTAATGTAA-CTTTTTAATT-----------
E. trimaculatus      GTGATAAGCTTATATTATTTAAAGTGCAAATAATACAC-TAATGTAATTCTCAATTTT--
P. leopardus         CACGCAA---TTAAACACGTGTAATTACAATATTGCAAATGTTTAAAAAAAAATACCTCT
P. areolatus         CACGCAA---TTAAACGCATGTGTTTACATTATTACAAATATGTTTTAAAAAACACCTCT
E. octofasciatus     TTTATTAGCCTAAAATGTGTTCATTTACATTATTATAA-TATTTAAATT-----------
E. septemfasciatus   TTTATTAGCCCAAAATGTGTTCATTTACATTATTATAA-TATTTAAATT-----------
```
