# Supplementary material for: A Comprehensive Description and Evolutionary Analysis of 22 Grouper (Perciformes, Epinephelidae) Mitochondrial Genomes with Emphasis on Two Novel Genome Organizations
Source: PLoS One. 2013 Aug 9;8(8):e73561. doi: 10.1371/journal.pone.0073561 (PMC3739747; doi:10.1371/journal.pone.0073561)
Supplement: Figure S2 — Sequence alignment of the 12 mitochondrial protein-coding genes (excluding ND6) used for phylogenetic analyses. Multiple sequence alignment was generated using CLUSTAL X [62] with minor manual adjustments. The manual modifications are highlighted in yellow. (DOCX) [file pone.0073561.s002.docx]

*A. rogaa* ATGATCTCA---ACACTTTTTACACATATTATTAACCCCCTAGCCTATATTGTCCCCGTG

*C. argus* ATGATCTCA---ACGCTTTTTACACATATTATCAACCCCTTAGCCTACATTGTGCCCGTA

*C. sonnerati* ATGATCTCA---ACACTTTTCACCCACATTATTAACCCTTTAGCCTACATCGTGCCCGTA

*E. fuscoguttatus* ATGATCTCA---ACACTTTTCACCCACATTATTAACCCCTTAGCCTATATCATCCCTGTT

*E. coioides* ATGATCTCA---ACACTCTTTACCCACATTGTAAACCCCTTAGCTTACATCATTCCCGTT

*E. bruneus* ATGATCTCA---ACACTTTTTACCCACGTCATCAACCCCTTAGCTTACATCATCCCAGTT

*E. moara* ATGATCTCA---ACACTTTTTACCCATATCATCAACCCCTTAGCTTACATCATCCCAGTT

*E. lanceolatus*  ATGATCTCA---ACACTTTTCACTCACATTATTAACCCCTTAACCTACATCATCCCTGTC

*A. leucogrammicus*  ATGATCTCA---ACACTTTTTACTCACATCATTAACCCCTTAGCTTACATCATCCCAATC

*C. altivelis* ATGATCTCA---ACACTTTTTACCCACGTTATCAACCCCCTAGCCTACATCATCCCTATC

*E. epistictus* ATGATCTCA---ACACTTTTCACCCATATTATCAACCCCTTGGCCTATATTGTCCCCGTC

*E. octofasciatus* ATGATCTCA---ACGCTTTTCACACATATTATTAACCCTTTAGCCTACATCGTTCCAGTA

*E. septemfasciatus* ATGATCTCA---ACGCTTTTCACACATATTATTAACCCTTTAGCCTACATCGTTCCAGTA

*T. dermopterus* ATGATCTCA---ACGCTTTTCACCCACATCATTAACCCCCTGGCCTATATCGTACCAGTA

*E. awoara* ATGATCTCA---ACGCTTTTCACTCATATTATTAATCCTTTAGCCTACATTGTCCCCGTA

*E. akaara* ATGATCTCA---ACGCTTTTTACACATATTATCAACCCTTTAGCCTACATTGTTCCTGTA

*E. trimaculatus* ATGATCTCA---ACGCTTTTCACCCACATTATTAACCCTTTAGCCTACATCGTCCCCGTA

*E. areolatus* ATGATCTCA---ACGCTTTTTACCCATATCATTAATCCCTTAGCCTATATCGTTCCCGTA

*V. albimarginata* ATGATCTCA---ACACTTATTACCCATATTATTAACCCTCTAGCTTACATCGTTCCCGTT

*V. louti* ATGATTTCA---ACACTTATTACCCACATTATCAACCCTTTAGCCTACATCGTCCCCGTT

*P. leopardus* ATGATTTCA---TACCTTTTCACGCACATTATTAACCCTTTAGCCTATATCGTGCCCGTC

*P. areolatus* ATGATTTCA---TTCCTTTTTACACATATTATTAACCCTTTGGCCTATATCGTGCCCGTC

*E. radiosum* ATGATTTCA---ATGCTTATTACCCACATCATCAACCCTCTAGCATTTATTGTGCCCGTC

*P. sieboldi* ATGCTCTCT---ACCATCATCACCCACATTGTCAACCCTTTGGCCTTCATTGTCCCCGTT

*E. armatus* ATGATCTCA---ACACTTATTACACATATCCTCAACCCTTTAGCCTTTATCGTCCCCGTT

*R. oxyrhynchus* ATGATCTCA---GCCCTAATAACCCAAATCCTTAACCCCCTAATCTTTATCGTCCCCGTT

*K. cinerascens* ATGATCTCA---ACACTCATCACCCATATTATCAACCCATTAGCCTTCATCGTCCCTGTT

*T. chatareus* ATGACCTCT---GCCCTCATAACACATATTATAAATCCCCTAGCCTTTATCGTGCCCGTT

*D. berycoides* ATGATTTCA---ACACTCATCACCTACATTATTAACCCCCTGGCCTTCATCGTACCCGTC

*H. typus* ATGATTTCA---ACACTCATCACCCACATTATTAACCCCCTGGCTTTTATCGTGCCTGTT

*M. argenteus* ATGATTACA---ACACTCATCACCCATATCATTAACCCACTAGCATTTATTGTGCCTGTC

*S. chuatsi* ATGATCTCA---ACACTCATCACCCACATCATTAACCCACTAGCTTTCATTGTTCCAGTT

*O. fasciatus* ATGATCTCA---ACACTCATCACCCATATTATTAACCCCCTAGCCTTCATCGTGCCCGTC

*P. trilineatum* ATGATCTCA---ACACTCATTACCCATATTATTAATCCTCTCGCCTGCATTGTACCTGTT

*M. salmoides* ATGATCTCA---ACACTCATCACCCATATCATCAATCCCCTAGCCTTCATTGTACCTATC

*P. tile* ATGATCTCA---ACACTCATCACCCATATCATTAACCCCCTGGCCTTTATTGTGCCAGTT

*L. argentimaculatus* ATGATCTCA---ACACTCATTACCCACATCATCAACCCCCTAGCCTTCATCGTCCCAGTC

*E. struhsakeri* ATGATTTCA---ACACTCATCACCCACATTATTAACCCCCTAACCTTTATCGTACCTGTT

*B. albus* ATGATATCA---GTACTGATCACCCATATTGTCAACCCCCTTATCTTTATTGTCCCCGTC

*C. auripes* ATGCCCTCA---ACACTTATCATTTATCTAATTAACCCCCTAACCTTTATTATCCCCGTT

*C. melampygus* ATGATCACA---GCATTAATTACCCACCTACTTAATCCCCTGGCCTTTATTGTCCCTGTC

*L. calcarifer* ATGATTTCA---ACACTTACCACACACATTATTAATCCCTTAGCATTCATTATTCCTGTT

*S. maena* ATGCTATCC---ACATTGTTTACCCACCTAATCAATCCCTTGGCCTTCATCGTCCCGGTC

*P. auriga* ATGCTCTAT---ACATTGTTTACTCACTTAATTAACCCACTGGCCTTTATCGTCCCCGTT

*C. lucidus* ATGATCCAC---ACTCTTATAACACACATTATTAACCCCCTTACACTCATTGTACCCGTT

*S. sihama* ATGTTCAAC---ACAATTTTTACCCACATCATCAATCCCCTGGCTTCCATTGTCCCCGTC

*C. loricula* ATGGTCTCA---TTATTTATAACCCACCTCATTAACCCGTTAGCTTTCATCGTTCCAGTC

*A. trutta* ATGTTTCCC---GTGATTTCATCTCACATCTTAAACCCCCTAGCCTTCATCGTGCCTGTC

*H. gemma* ATGATCTTAAATATTATTTTCACGCACCTTATTAACCCGCTAGCCTACATCGTACCTGTT

*P. flavescens* ATGATTTCA---ACATTGATCACCCACATTATTAACCCCTTAGCCTTTATCGTACCCGTC

*P. macrolepida* ATGATTTCA---GTACTTATTACCCATATTATTAATCCCCTAGCCTTCATCGTCCCCGTC

*S. canadensis* ATGATCTCA---ATATTCATCACCCACATTATTAATCCTTTAGCCTTTATCGTGCCTGTT

*A. rogaa* CTTCTAGCCGTCGCCTTTCTTACACTAGTAGAACGAAAAGTCTTGGGTTATATACAATTC

*C. argus* CTACTAGCAGTCGCCTTCCTTACACTAGTTGAACGTAAAGTCTTAGGTTATATACAATTT

*C. sonnerati* CTTCTAGCAGTTGCTTTCCTCACCCTAGTCGAACGAAAAGTTTTAGGATACATACAATTC

*E. fuscoguttatus* CTATTAGCTGTTGCCTTCCTCACATTAGTAGAACGAAAAGTCCTTGGATACATGCAATTT

*E. coioides* CTCCTAGCCGTTGCCTTCCTCACACTAGTGGAACGAAAAGTCCTCGGGTATATACAATTT

*E. bruneus* CTCCTGGCCGTTGCCTTCCTTACATTAGTAGAACGAAAAGTTCTTGGGTATATACAATTT

*E. moara* CTCCTGGCCGTTGCCTTCCTTACATTAATAGAACGAAAAGTCCTCGGATACATACAATTT

*E. lanceolatus*  CTCCTAGCTGTTGCCTTTCTTACATTAGTAGAACGAAAAGTCCTCGGATACATGCAATTT

*A. leucogrammicus*  CTCTTAGCCATTGCCTTTCTCACATTAGTAGAGCGAAAAGTCCTTGGGTATATACAATTT

*C. altivelis* CTCCTAGCCATTGCCTTCCTCACACTAGTAGAACGGAAAGTCCTCGGGTATATACAATTT

*E. epistictus* CTCCTAGCCGTTGCCTTCCTTACGCTAGTTGAACGAAAAGTCCTAGGGTATATACAATTT

*E. octofasciatus* CTACTGGCTGTTGCCTTCCTTACACTAGTTGAACGAAAAGTCCTAGGCTATATACAATTT

*E. septemfasciatus* CTACTGGCTGTTGCCTTCCTTACACTAGTTGAACGAAAAGTCCTAGGCTATATACAATTT

*T. dermopterus* CTACTAGCCGTTGCCTTCCTTACACTAGTTGAACGAAAAGTCCTGGGATATATGCAATTC

*E. awoara* CTTCTAGCTGTTGCTCTCCTTACACTGGTTGAACGAAAAGTCCTAGGATATATGCAATTC

*E. akaara* CTTCTGGCTGTTGCTTTCCTTACACTAGTTGAACGAAAAGTCCTAGGATATATACAATTT

*E. trimaculatus* CTTCTAGCCGTTGCTTTCCTTACACTAGTTGAACGAAAAGTCCTGGGGTACATACAATTC

*E. areolatus* CTTCTAGCCGTTGCCTTCCTCACACTAGTCGAACGAAAAGTCCTAGGATATATACAATTT

*V. albimarginata* CTTCTAGCCGTCGCTTTCCTTACGCTAGTCGAACGAAAAGTCCTAGGCTATATGCAACTA

*V. louti* CTACTAGCCGTCGCTTTCCTCACCCTAGTTGAACGAAAAGTCTTAGGCTATATACAACTA

*P. leopardus* CTCCTAGCTGTTGCCTTCCTTACACTCCTAGAACGAAAAGTCCTAGGCTACATGCAAATA

*P. areolatus* CTTTTAGCCGTTGCCTTCCTTACACTCCTAGAACGGAAGGTCCTAGGTTATATGCAAATA

*E. radiosum* CTTTTGGCTGTTGCTTTTTTAACCCTCCTCGAACGAAAAGTTCTAGGCTATATGCAGTTA

*P. sieboldi* CTCTTAGCCGTCGCCTTCTTAACCCTCCTTGAACGAAAAGTCTTGGGCTACATGCAGCTG

*E. armatus* CTATTAGCTGTTGCCTTTTTAACCCTCATTGAACGAAAGGTACTCGGCTATATACAACTA

*R. oxyrhynchus* CTACTAGCTGTTGCCTTCTTCACCCTCCTTGAACGAAAAGTGCTCGGCTACATGCAACTA

*K. cinerascens* CTCCTAGCCGTTGCTTTCCTAACCCTAATCGAACGCAAAGTGCTAGGCTACATACAACTA

*T. chatareus* CTCTTGGCCGTAGCCTTCCTCACCCTCATCGAACGAAAAGTCCTAGGCTATATACAACTA

*D. berycoides* CTACTAGCTGTTGCCTTCCTAACCCTTATCGAACGAAAAGTGCTCGGCTACATGCAACTA

*H. typus* TTACTAGCTGTTGCCTTCCTAACCCTAATCGAACGAAAAGTACTTGGCTACATACAACTA

*M. argenteus* TTGCTAGCTGTTGCTTTCCTGACCCTTCTTGAACGAAAAGTTCTAGGCTACATACAACTA

*S. chuatsi* CTTCTAGCCGTTGCTTTTCTAACCCTACTTGAACGTAAAGTGCTTGGCTACATACAACTG

*O. fasciatus* TTACTAGCCGTTGCCTTCCTAACCCTACTCGAACGCAAAGTGCTTGGCTACATACAACTA

*P. trilineatum* CTTCTAGCCGTTGCCTTTTTAACCCTTCTTGAACGAAAAGTGCTAGGTTATATACAACTA

*M. salmoides* CTCCTAGCTGTTGCCTTCCTAACCCTTCTCGAACGGAAAGTTCTGGGCTATATACAATTA

*P. tile* CTCCTAGCCGTCGCCTTCCTTACCCTAATCGAACGAAAAGTACTTGGCTATATACAACTA

*L. argentimaculatus* CTCCTAGCCGTTGCTTTCCTAACCCTTCTTGAACGAAAAGTCTTAGGTTATATACAACTG

*E. struhsakeri* CTACTAGCTGTTGCCTTCTTAACCTTGCTTGAACGAAAAGTGCTAGGTTATATGCAACTG

*B. albus* CTCCTTGCCGTCGCTTTTCTAACACTCGTAGAACGAAAAGTACTCGGTTATATGCAACTG

*C. auripes* TTACTAGCCGTTGCTTTCTTAACATTACTTGAACGAAAAGTCCTAGGCTATATACAACTG

*C. melampygus* CTTCTAGCCGTTGCCTTCCTTACCCTAATTGAACGAAAAGTCCTAGGCTATATGCAACTA

*L. calcarifer* CTCCTAGCCGTTGCCTTCTTAACCCTGATTGAACGAAAGGTCTTGGGCTACATGCAACTC

*S. maena* CTTCTAGCCGTTGCCTTCCTTACCCTTCTTGAACGAAAAGTTCTCAGCTATATGCAACAC

*P. auriga* CTTCTAGCCGTTGCTTTTCTAACACTACTTGAACGCAAAGTCCTAGGTTATATACAACTA

*C. lucidus* CTTCTAGCCGTCGCCTTCCTTACACTTCTTGAACGAAAAGTACTCGGCTATATACAACTA

*S. sihama* CTGTTAGCTGTAGCATTCCTTACCCTTCTCGAACGAAAAGTTTTAGGTTACATACAACTG

*C. loricula* CTCCTGTCCGTTGCCTTCCTAACTCTACTTGAACGAAAAGTGCTAGGGTACATGCAATTT

*A. trutta* CTACTAGCCGTAGCCTTTCTTACTCTACTAGAACGGAAAGTTCTAGGCTACATACAACTC

*H. gemma* CTTCTAGCAGTAGCATTTCTTACTCTGCTTGAGCGAAAAATTTTAGGATACATGCAATTA

*P. flavescens* CTCCTGGCTGTTGCCTTCTTAACCCTACTTGAACGAAAAGTACTTGGTTACATACAATTA

*P. macrolepida* CTCTTAGCTGTTGCCTTTCTAACCCTCCTTGAACGAAAAGTTCTTGGGTATATGCAACTA

*S. canadensis* CTACTAGCTGTTGCCTTCCTAACCCTGCTTGAACGAAAAGTGCTTGGTTATATACAACTA

*A. rogaa* CGAAAAGGCCCTAACATTGTTGGACCTTACGGGCTGCTACAACCAATCGCAGATGGTGTA

*C. argus* CGAAAGGGTCCCAACATTGTTGGACCTTATGGGCTGCTACAGCCAATTGCAGACGGTGTT

*C. sonnerati* CGAAAAGGTCCTAACATCGTCGGACCCTACGGTCTACTTCAACCCATTGCAGATGGTGTC

*E. fuscoguttatus* CGAAAAGGTCCTAACATCGTCGGACCCTATGGCCTTCTACAACCAATTGCCGACGGAGTA

*E. coioides* CGAAAAGGCCCTAATATCGTAGGGCCTTACGGCCTACTACAACCAATCGCCGACGGAGTA

*E. bruneus* CGAAAAGGCCCTAACATCGTTGGACCCTACGGCCTCCTACAACCAATTGCCGACGGAGTA

*E. moara* CGAAAAGGCCCTAACATCGTTGGACCCTACGGCCTCCTACAACCAATCGCCGACGGGGTA

*E. lanceolatus*  CGAAAAGGACCTAACATCGTTGGACCCTATGGCCTTCTACAACCAATCGCCGACGGAGTA

*A. leucogrammicus*  CGAAAAGGCCCTAACATCGTCGGACCCTACGGCCTCCTACAACCAATTGCCGATGGAGTA

*C. altivelis* CGAAAGGGTCCCAACATCGTTGGACCCTACGGTCTCCTACAACCAATCGCCGACGGGGTA

*E. epistictus* CGAAAAGGCCCTAACATCGTTGGACCCTACGGCCTCTTACAACCAATCGCTGATGGCGTA

*E. octofasciatus* CGAAAGGGCCCTAATATCGTTGGGCCCTACGGCCTCTTACAACCAATCGCCGACGGGGTA

*E. septemfasciatus* CGAAAGGGCCCTAATATCGTTGGGCCCTACGGCCTCTTACAACCGATCGCCGACGGAGTA

*T. dermopterus* CGAAAGGGGCCTAACATCGTCGGGCCCTATGGCCTTCTACAGCCCATCGCCGACGGAGTA

*E. awoara* CGAAAAGGCCCAAATATCGTTGGACCCTACGGCCTTCTTCAACCAATCGCCGACGGAGTA

*E. akaara* CGAAAAGGCCCAAACATCGTCGGGCCTTACGGCCTTCTCCAACCAATCGCCGATGGGGTA

*E. trimaculatus* CGAAAAGGCCCTAACATCGTCGGACCCTATGGTCTCCTTCAACCAATCGCCGACGGCGTA

*E. areolatus* CGAAAAGGCCCAAACATCGTTGGACCCTACGGTCTCCTTCAACCAATCGCCGACGGAGTT

*V. albimarginata* CGAAAAGGCCCAAATATTGTAGGACCCTATGGTCTACTTCAACCAATTGCTGATGGTGTA

*V. louti* CGAAAAGGCCCGAATATTGTTGGACCCTATGGTTTACTCCAACCAATTGCTGACGGTGTA

*P. leopardus* CGCAAAGGCCCAAATATCGTTGGTCCTTATGGACTTCTACAACCCATCGCCGATGGGGTA

*P. areolatus* CGTAAAGGTCCGAACATCGTAGGTCCTTATGGTCTTCTCCAACCCATTGCCGACGGGGTA

*E. radiosum* CGTAAGGGTCCTAATATTGTGGGACCTTATGGATTATTACAGCCCATCGCCGACGGTGTA

*P. sieboldi* CGGAAAGGCCCTAATATCGTTGGACCTTACGGACTACTTCAGCCCATTGCAGACGGGGTA

*E. armatus* CGAAAAGGCCCTAACATCGTTGGACCCTACGGCCTCCTCCAACCTATTGCTGACGGAGTA

*R. oxyrhynchus* CGAAAAGGCCCTAACATCGTCGGCCCCTACGGCCTTCTCCAGCCCATTGCCGATGGTGTA

*K. cinerascens* CGAAAAGGCCCAAACATCGTTGGCCCCTACGGCCTGTTACAACCTATTGCCGACGGAGTG

*T. chatareus* CGAAAAGGACCAAACATCGTAGGACCTTACGGCCTCCTTCAGCCTATTGCTGACGGAGTA

*D. berycoides* CGAAAAGGCCCAAACATTGTCGGGCCCTATGGTCTCCTGCAGCCCATTGCCGATGGGGTA

*H. typus* CGAAAAGGTCCAAACATTGTCGGACCATATGGCCTCCTGCAACCCATCGCCGACGGGGTT

*M. argenteus* CGAAAAGGACCAAACATTGTTGGCCCTTACGGCCTCCTCCAACCTATCGCTGATGGCGTT

*S. chuatsi* CGAAAAGGTCCAAACATTGTAGGACCCTACGGCCTCCTACAACCCATCGCCGACGGAGTG

*O. fasciatus* CGAAAAGGCCCCAACATTGTTGGCCCTTACGGCCTCCTACAGCCCATCGCCGACGGAGTA

*P. trilineatum* CGAAAAGGGCCAAATATCGTTGGACCCTACGGCCTCCTTCAACCCATCGCCGATGGTGTC

*M. salmoides* CGAAAAGGCCCAAACATTGTAGGCCCATACGGCCTCCTTCAACCCATCGCCGATGGGGTA

*P. tile* CGAAAAGGACCAAACATTGTAGGCCCCTACGGTCTTCTTCAACCAATCGCCGACGGAGTT

*L. argentimaculatus* CGAAAAGGCCCAAACATCGTAGGCCCCTATGGACTCCTTCAACCCATCGCTGACGGAGTC

*E. struhsakeri* CGAAAAGGGCCAAACATTGTAGGGCCTTACGGCTTATTACAACCCATTGCCGACGGAGTT

*B. albus* CGAAAAGGCCCGAACGTAGTAGGCCCTTACGGCCTACTACAACCAATCGCCGACGGGGTC

*C. auripes* CGTAAAGGACCAAACGTTGTAGGCCCTTACGGCCTTCTCCAGCCCATCGCAGACGGCCTT

*C. melampygus* CGAAAAGGCCCTAACGTAGTAGGACCCTACGGACTCCTGCAACCTATCGCTGACGGAGTT

*L. calcarifer* CGAAAAGGTCCTAACATTGTCGGACCCTACGGCCTACTTCAACCCATCGCCGATGGAGTG

*S. maena* CGAAAAGGACCTAACATTGTTGGCCCCTACGGCCTTCTTCAACCCATCGCTGATGGCGTA

*P. auriga* CGAAAGGGCCCCAACATCGTTGGCCCCTACGGTCTCCTCCAACCTATCGCCGATGGAGTA

*C. lucidus* CGAAAAGGCCCTAATATTGTAGGCCCCTACGGCCTCCTACAACCCATCGCCGATGGCATT

*S. sihama* CGAAAAGGACCCAATGTTGTAGGCCCATATGGACTTCTTCAACCAATCGCCGATGGTGTA

*C. loricula* CGGAAAGGCCCTAATATTGTAGGCCCCTACGGAATTTTTCAACCCATCGCTGATGGGATT

*A. trutta* CGAAAGGGCCCAAACATTGTAGGACCTTACGGACTTCTCCAACCAATCGCTGATGGAGTA

*H. gemma* CGTAAAGGCCCTAATATCGTAGGGCCCTACGGACTATTCCAACCTATCGCTGATGGGGTA

*P. flavescens* CGAAAAGGCCCTAATATTGTAGGGCCTTATGGGCTGCTGCAGCCAATCGCCGATGGTGTT

*P. macrolepida* CGAAAGGGCCCGAATATTGTCGGACCCTACGGACTCCTTCAACCTATCGCTGACGGCGTA

*S. canadensis* CCAAAAGGTCCAAACATTGTAGGGCCTTACGGCCTATTGCAGCCAATTGCTGATGGTGTT

*A. rogaa* AAACTATTTACCAAAGAACCAGTCCGCCCCTCCACTGCCTCCCCTATTCTCTTTCTACTG

*C. argus* AAACTATTTACTAAGGAACCAGTCCGTCCTTCTACCGCTTCCCCTATTCTTTTCCTGCTG

*C. sonnerati* AAACTATTCACTAAAGAACCAGTTCGACCTTCAACCGCCTCCCCCATTCTATTCCTCCTA

*E. fuscoguttatus* AAACTATTTACTAAAGAACCCGTTCGCCCCTCAACCGCCTCCCCCATCCTATTCCTCCTC

*E. coioides* AAATTATTTACCAAAGAGCCCATCCGCCCTTCAACTGCCTCCCCCATCCTATTTCTTCTC

*E. bruneus* AAATTATTTACTAAAGAACCCGTCCGTCCCTCGACCGCCTCCCCCATTTTATTCCTCCTC

*E. moara* AAATTATTTACTAAAGAACCCGTCCGCCCCTCAACCGCCTCCCCCATTTTATTCCTCCTC

*E. lanceolatus*  AAACTATTTACTAAAGAACCCATCCGCCCCTCAACTGCCTCCCCTATCCTATTTCTCCTC

*A. leucogrammicus*  AAACTATTTACTAAAGAACCTATTCGCCCCTCAACCGCCTCTCCCATCCTATTCCTCCTC

*C. altivelis* AAACTATTCACCAAAGAACCTGTCCGCCCTTCAACCGCCTCCCCTATTCTATTTCTCCTC

*E. epistictus* AAATTATTTACCAAAGAACCCGTCCGCCCATCAACCGCCTCCCCAATCCTCTTTCTCCTC

*E. octofasciatus* AAATTATTCACCAAAGAACCTATTCGCCCCTCAACTGCCTCCCCAATCCTCTTCCTCCTC

*E. septemfasciatus* AAACTGTTTACCAAAGAACCTATTCGCCCATCAACTGCCTCCCCAATCCTCTTCCTCCTC

*T. dermopterus* AAACTATTCACCAAAGAACCCATCCGCCCCTCAACTGCCTCCCCAATTCTCTTCCTCCTC

*E. awoara* AAACTATTTACCAAAGAGCCCGTTCGTCCCTCAACCGCCTCCCCTATTCTTTTTCTCCTC

*E. akaara* AAGCTATTTACCAAAGAGCCCGTTCGTCCCTCAACCGCCTCACCCATTCTTTTTCTTCTC

*E. trimaculatus* AAATTATTCACCAAAGAGCCTGTTCGCCCCTCAACCGCTTCTCCTATCCTCTTCCTTCTC

*E. areolatus* AAACTATTCACTAAAGAGCCTGTCCGACCCTCAACCGCCTCCCCCATCCTCTTCCTCTTC

*V. albimarginata* AAACTCTTTACCAAAGAACCCGTACGACCTTCCACTGCATCCCCAATTTTATTTCTCCTA

*V. louti* AAACTCTTTACCAAAGAACCCGTCCGACCTTCCACTGCATCCCCAATTTTGTTTCTCTTA

*P. leopardus* AAACTATTTACTAAAGAGCCCATTCGCCCCTCTACCTCCTCCCCATTCTTATTCCTCTTA

*P. areolatus* AAACTTTTTACCAAGGAGCCTATTCGCCCCTCTACCTCCTCTCCTTTCCTATTCCTCTTA

*E. radiosum* AAGCTATTTATCAAAGAGCCTGTCCGCCCTTCAACCGCCTCCCCGCTACTCTTTCTTCTT

*P. sieboldi* AAGCTATTCATTAAGGAACCTGTTCGGCCTTCCACCGCATCCCCGGTTCTATTTCTTCTT

*E. armatus* AAACTATTTATCAAAGAACCCGTGCGACCTTCCACCTCCTCCCCCGTTTTATTTCTTTTA

*R. oxyrhynchus* AAACTGTTCATTAAAGAACCAGTCCGCCCCTCAACTTCTTCACCAGTCCTCTTCCTCCTA

*K. cinerascens* AAACTCTTTATCAAAGAACCAGTCCGACCCTCCACCTCCTCCCCCGTCCTATTCCTTCTA

*T. chatareus* AAACTATTTATCAAAGAACCCATCCGTCCCTCAACCGCATCCCCAGTCCTATTCTTATTA

*D. berycoides* AAACTATTTATTAAAGAACCAGTACGACCCTCGACCTCCTCCCCAATTCTGTTTCTTCTA

*H. typus* AAACTATTCATTAAAGAACCCGTACGCCCCTCAACCTCCTCCCCCATTCTATTTCTCCTA

*M. argenteus* AAACTTTTCATCAAAGAACCTGTACGACCCTCCACCTCCTCCCCCCTATTATTTCTCCTC

*S. chuatsi* AAACTATTTATTAAAGAACCCGTGCGCCCCTCAACCTCTTCCCCCATTCTCTTCCTTCTA

*O. fasciatus* AAACTTTTCATTAAAGAACCAGTACGCCCTTCCACTTCCTCTCCCGTCCTATTTTTATTA

*P. trilineatum* AAACTCTTCATTAAAGAACCCGTACGCCCCTCAACCTCCTCTCCTCTGCTATTCCTTTTA

*M. salmoides* AAACTATTTATTAAGGAACCAGTCCGCCCCTCAACCTCCTCCCCAATTCTATTTATCTTA

*P. tile* AAACTATTTATTAAAGAACCTGTACGTCCCTCAACCTCCTCCCCCCTTCTATTTCTTTTG

*L. argentimaculatus* AAACTATTCATTAAAGAGCCAGTACGACCCTCCACCTCTTCCCCCCTTCTATTCCTTCTA

*E. struhsakeri* AAACTGTTCATTAAAGAACCTGTGCGACCCTCAACTTCCTCCCCAATCCTCTTCCTCCTG

*B. albus* AAACTATTTATTAAAGAGCCCGTAAAACCATCCACCTCTTCTCCCATCCTATTCCTCCTA

*C. auripes* AAACTATTTATTAAAGAACCCGTACGCCCCTCAACTTCCTCCCCCCTTCTCTTCCTTCTA

*C. melampygus* AAACTATTTATTAAAGAACCAGTACGACCTTCCACCTCTTCCCCAATTCTCTTCCTCCTT

*L. calcarifer* AAATTATTTATTAAAGAACCCATCCGACCATCCACTTCTTCCCCCCTCCTCTTTCTTATA

*S. maena* AAACTATTTATTAAAGAACCTGTACGACCCTCAACCTCTTCCCCCATTCTCTTTCTATTT

*P. auriga* AAACTGTTCATCAAAGAGCCCGTACGACCATCAACCTCCTCCCCCATTCTCTTTTTACTA

*C. lucidus* AAACTCTTTATTAAGGAACCCGTCCGACCCTCTACCTCATCCCCCATCCTATTTATTATT

*S. sihama* AAATTGTTTATTAAAGAACCCATCCGACCCTCTACCTCTTCTCCAGTCCTATTCTTGGCC

*C. loricula* AAACTTTTCATTAAAGAGCCCATTAAACCTTTAACCTCCTCCCCCTTCCTATTCCTAGCC

*A. trutta* AAACTTTTCATCAAAGAGCCAGTCCAACCCTCAACCTCGTCCCCCGTCTTATTTTTACTT

*H. gemma* AAACTCTTCATTAAAGAACCTATCCGACCATCAACTTCATCACCTCTCCTGTTCCTATTT

*P. flavescens* AAACTCTTTATTAAAGAACCTGTCCGACCTTCTACTGCTTCCCCTGTTCTTTTTCTTCTT

*P. macrolepida* AAACTATTTATTAAAGAACCAGTGCGCCCCTCCACCGCTTCCCCCGTCCTTTTCCTTCTG

*S. canadensis* AAACTCTTCATTAAAGAGCCTATTCGCCCTTCCACCTCTTCCCCCGTCCTTTTTCTATTA

*A. rogaa* GCCCCTATACTCGCCCTTACCCTTGCCCTCACCCTATGAGCCCCTCTCCCCATACCCTAT

*C. argus* GCCCCCATGCTTGCCCTTACCCTTGCCCTCGCCCTATGGGCCCCCCTTCCCATGCCTTAC

*C. sonnerati* GCCCCAATACTTGCCCTCACCCTAGCTCTCACCCTATGAGCCCCCCTCCCAATACCCTTT

*E. fuscoguttatus* GCCCCAATCTTAGCCCTTACTCTTGCCCTCACTCTATGAGCTCCCCTCCCTATACCATAC

*E. coioides* GCCCCAATATTAGCCCTTACTCTTGCCCTTACTCTATGAGCCCCCCTCCCTATACCATAC

*E. bruneus* GCCCCAATATTAGCCCTTACTCTTGCCCTTACCCTATGGGCCCCTCTTCCCATACCATAC

*E. moara* GCCCCGATATTGGCCCTTACTCTTGCCCTTACCCTATGAGCCCCCCTTCCCATACCATAC

*E. lanceolatus*  GCCCCAATATTAGCCCTCACCCTTGCCCTCACCCTATGAGCCCCCCTCCCCATACCATAC

*A. leucogrammicus*  GCCCCAATATTAGCCCTCACTCTCGCCCTCACCCTATGAGCCCCACTCCCCATACCATAC

*C. altivelis* GCCCCAGTATTAGCCCTCACTCTTGCCCTCACCCTATGAGCCCCCCTTCCAATACCGTAC

*E. epistictus* GCCCCAATATTAGCCCTTACCCTCGCCCTAACTCTATGAGCCCCCCTCCCTATACCGTAC

*E. octofasciatus* GCTCCAATGTTAGCCCTTACTCTCGCCCTTACCTTATGAGCCCCCCTCCCCATACCATAC

*E. septemfasciatus* GCTCCAATATTAGCCCTTACTCTTGCCCTTACCTTATGAGCCCCCCTCCCCATACCATAC

*T. dermopterus* GCCCCAATATTAGCCCTCACTCTTGCCCTCACCCTATGGGCCCCCCTCCCCATACCATAC

*E. awoara* GCCCCAATGCTAGCCCTTACCCTTGCCCTCACCTTATGGGCCCCTCTTCCCATCCCATAC

*E. akaara* GCTCCAATATTAGCCCTCACCCTCGCCCTCACCTTGTGAGCCCCCCTTCCTATGCCATAT

*E. trimaculatus* GCCCCAATACTAGCCCTTACCTTAGCCCTTACTCTGTGAGCCCCCCTCCCCATGCCATAC

*E. areolatus* GCCCCAATATTAGCCCTCACCCTTGCCCTCACCTTATGGGCCCCCCTTCCCATACCATAC

*V. albimarginata* GCCCCTATACTAGCCTTAACACTAGCCCTTACCCTTTGAACCCCTCTCCCTATACCTTAC

*V. louti* GCTCCTATACTAGCTCTAACACTGGCCCTTACCCTCTGAGCCCCTCTCCCTATACCTTAC

*P. leopardus* GCCCCTACTCTAGCCCTCACACTAGCCCTTATGCTCTGGGCCCCAATACCTATACCTTAC

*P. areolatus* GCCCCTACTTTAGCACTTACACTAGCCCTTACGCTCTGAGCCCCAATACCCATACCTTAC

*E. radiosum* ACCCCAATTCTTGCTCTCACACTTGCCCTGACCCTCTGGGCCCCCATGCCTATGCCGTAC

*P. sieboldi* GCCCCAATACTTGCCCTTACCCTAGCCCTCCTCCTTTGAGTCCCCATACCCCTTCCCTAT

*E. armatus* ACCCCTATACTGGCCCTTACCCTTGCACTCACTCTCTGAGCCCCCATACCCCTACCCTAC

*R. oxyrhynchus* ACCCCCATATTGGCCCTCACCTTGGCCCTTGCACTTTGAACACCTCTTCCCTTACCATAT

*K. cinerascens* ACCCCCATACTGGCACTCACCCTCGCCCTCACACTATGAGCCCCAATACCTCTCCCCTAC

*T. chatareus* ACCCCAACAATAGCCCTAACCATTGCTCTTACTCTATGAGCCCCTATACCCATCCCATAC

*D. berycoides* ACCCCGATACTGGCCCTCACACTAGCCCTTACCCTCTGAGCACCTCTGCCCATGCCCTAC

*H. typus* ACCCCCATATTAGCCCTCACTCTTGCCCTCACCCTATGAGCACCCATGCCCATCCCTTAC

*M. argenteus* ACCCCCATGCTAGCCCTGACACTCGCCCTCACCCTGTGGTCTCCCATGCCTCTACCATAC

*S. chuatsi* ACCCCGATGCTAGCCCTTACACTTGCCCTCACCCTGTGAGCCCCAATACCCCTTCCCTAC

*O. fasciatus* ACCCCCATACTAGCACTAACCCTTGCTCTTACACTATGAGCCCCAATACCTCTCCCTTAC

*P. trilineatum* ACCCCTATATTAGCCCTAACGCTCGCACTCACCCTATGAGCCCCTATGCCACTACCCTAC

*M. salmoides* ACACCCATATTAGCCCTAACCCTGGCCCTCACATTATGAGCCCCTATACCTCTGCCCCAT

*P. tile* ACCCCAATACTAGCCCTTACCCTAGCTCTCACCCTATGGGCCCCTATGCCCCTCCCATAC

*L. argentimaculatus* ACCCCTATGCTGGCCCTAACACTTGCTCTCACCCTATGAGCCCCGATACCACTCCCCTAC

*E. struhsakeri* ACCCCGATATTAGCCCTCACACTCGCCCTTACCCTATGGGCCCCAATGCCCCTGCCGTAC

*B. albus* GCCCCCATACTCGCGCTTACTCTCGCCCTCACCCTATGGGCCCCGATGCCCCTCCCATAC

*C. auripes* ACCCCCGCCCTGGCTCTTACACTGGCTCTCACACTATGAGCCCCCATACCCCTACCTTAC

*C. melampygus* GCCCCCATATTAGCACTAACCCTGGCTCTCACACTCTGAGCACCAATGCCTCTCCCCTAC

*L. calcarifer* ACCCCCATACTAGCCCTTACTTTGGCCCTCACCCTCTGAACCCCAATACCCCTCCCATAC

*S. maena* ACCCCAATGCTTGCACTAACACTTGCCCTCACTCTTTGAGCCCCAATACCTCTTCCCTAC

*P. auriga* GCCCCCATACTTGCACTCACATTGGCCCTCACCCTCTGAGCCCCAATGCCTATGCCATAC

*C. lucidus* ACCCCTATATTGGCCCTCACACTAGCTCTAACACTTTGAGCACCCATACCTTTGCCCTAC

*S. sihama* GCCCCCATACTGGCTCTCACCTTAGCTCTCACCCTTTGAGCCCCAATGCCCCTTCCACAC

*C. loricula* ACACCGATTATGGCCTTAACACTAGCACTCACCCTATGAGCACCCATGCCCATGCCACAC

*A. trutta* GCCCCAATACTTGCTTTGACCCTCGCTTTAACCCTCTGAGCCCCAATACCCTTGCCGTAT

*H. gemma* TCACCAATACTAGCCCTAACCCTTGCTCTTACTCTTTGAGCCCCCCTTCCCCTCCCGTAC

*P. flavescens* GCCCCTATTCTCGCCCTCACACTAGCCCTCACCCTTTGAGCCCCTATGCCTATGCCCTAC

*P. macrolepida* ACCCCTATTCTCGCCTTAACACTTGCCTTGACCCTATGGGCCCCAATGCCCATACCTTAC

*S. canadensis* GCCCCTATCCTTGCACTCACCCTTGCTCTAACCCTTTGGGCTCCAATGCCTATACCATAC

*A. rogaa* CCCATCCTTGACCTAAACTTAGGTATTCTCTTTATTCTAGCCCTCTCCAGTCTAGCAGTA

*C. argus* CCGATCCTAGACCTAAACCTAGGCATCCTCTTTATTCTAGCCCTTTCTAGCCTAGCAGTA

*C. sonnerati* CCCATCCTAGACCTAAACCTAGGCATTCTCTTTATTCTAGCCCTCTCAAGCCTAGCAGTA

*E. fuscoguttatus* CCCATCCTAGACCTAAACCTGGGAATCCTTTTCATCCTAGCACTATCCAGCTTAGCAGTG

*E. coioides* CCCATCCTAGATTTAAACCTGGGAATCCTCTTCATCCTAGCACTATCCAGCCTAGCAGTA

*E. bruneus* CCCATCCTAGACCTAAACCTAGGGATTCTTTTTATCCTGGCACTGTCCAGTCTAGCAGTA

*E. moara* CCCATCCTAGACCTAAACCTAGGAATTCTTTTTATCCTAGCACTCTCCAGCCTAGCAGTA

*E. lanceolatus*  CCCATTCTAGACCTAAATCTAGGGATTCTCTTCATTCTTGCACTATCTAGCTTAGCAGTA

*A. leucogrammicus*  CCTATTTTAGACCTAAATCTAGGAATTCTCTTCATCCTCGCACTATCTAGCCTAGCGGTA

*C. altivelis* CCAATCCTAGACCTAAACCTAGGAATCCTTTTTATCCTGGCACTCTCCAGCCTAGCAGTA

*E. epistictus* CCCATCTTAGACTTAAACTTAGGCATCCTCTTTATCCTCGCACTATCCAGCCTAGCAGTC

*E. octofasciatus* CCTGTCCTTGATTTAAATCTAGGTATTCTTTTTATCCTAGCACTGTCCAGTCTAGCAGTA

*E. septemfasciatus* CCTATCCTCGATTTAAACCTAGGTATTCTTTTTATTCTAGCACTATCCAGTCTAGCAGTA

*T. dermopterus* CCCATCCTCGATTTAAATCTGGGCATTCTCTTTATCCTCGCACTATCCAGTCTAGCAGTA

*E. awoara* CCTATTCTAGACCTGAATCTAGGAATTCTTTTTATTTTAGCACTTTCTAGTTTAGCGGTG

*E. akaara* CCTATTCTAGACTTAAATCTGGGGATTCTCTTCATTTTAGCACTTTCTAGTTTAGCAGTA

*E. trimaculatus* CCCATTCTAGACTTAAACCTGGGTATTCTCTTCATCCTGGCACTATCAAGTCTGGCAGTA

*E. areolatus* CCTATCCTCGACCTAAATCTAGGGATCCTCTTTATTTTAGCACTATCCAGCTTAGCAGTA

*V. albimarginata* CCTATTATTGACCTAAACCTTGGTATTCTATTTATCCTCGCACTTTCTAGCCTAGCAGTT

*V. louti* CCTATTATTGACCTAAACCTTGGCATCCTATTTATCCTCGCACTTTCCAGCCTAGCAGTC

*P. leopardus* CCAATTATTAACCTCAACCTCGGAATCCTCTTTATCCTTGCACTTTCCAGCCTTGCAGTC

*P. areolatus* CCAATTATTGACCTCAACCTCGGGATCCTCTTTATCCTGGCACTTTCTAGCCTTGCAGTT

*E. radiosum* CCTGTTGTGGACCTCAATCTCGGAATTCTGTTTATTCTTGCCTTATCAAGCCTAGCCGTT

*P. sieboldi* CCGGTTGCCGACCTAAACCTTGGCATCCTCTTTATTCTCGCCATGTCCAGCCTCGCGGTA

*E. armatus* CCAGTAACGGACCTTAACCTAGGAATTTTATTCATTTTAGCCCTCTCCAGTCTTGCAGTA

*R. oxyrhynchus* CCAATAGCCGACCTTAACCTGGGCATTCTATTCATTCTAGCCCTATCCAGCCTTGCAGTG

*K. cinerascens* CCAGTGACCGACCTAAATTTAGGCATTCTCTTCATCCTAGCCCTTTCTAGTCTTGCAGTC

*T. chatareus* CCAGTCACGGACCTAAACTTAGGAATCCTTTTTATTCTAGCACTATCAAGCCTAGCAGTC

*D. berycoides* CCCGTGGCGGACCTTAACCTAGGCATCCTATTTATCCTAGCCCTATCCAGTCTCGCCGTA

*H. typus* CCAGTAACAGATCTCAACCTAAGCATTCTATTTGTTTTAGCCCTATCTAGCCTTGCGGTC

*M. argenteus* CCCGTCATCGACCTAAACCTAGGCATCCTATTTATCCTAGCCCTATCCAGTCTTGCAGTC

*S. chuatsi* CCAGTAATTGACCTAAACTTAGGTATCTTATTTATCTTAGCCCTCTCCAGCCTTGCAGTC

*O. fasciatus* CCTGTAGTTGACCTAAATCTTGGCATTCTATTTATCCTAGCCCTTTCCAGCCTTGCAGTC

*P. trilineatum* CCAATGGTTGACCTAAATCTAGGTATCCTGTTTATTCTAGCCCTTTCTAGCCTCGCGGTC

*M. salmoides* CCCGTAACAGACCTCAACCTGGGCATCCTTTTTATCCTAGCCTTATCTAGCCTTGCTGTA

*P. tile* CCCGTGATCGACTTAAATCTCGGTATCTTATTTATCCTTGCACTATCCAGCCTTGCAGTC

*L. argentimaculatus* CCTGTAATCGACCTTAACCTGGGCATCTTGTTTATTCTGGCCCTATCCAGCCTAGCAGTC

*E. struhsakeri* CCAGTTATTGATCTAAACCTAGGCATTTTATTTGTCCTTGCCCTATCCAGCCTGGCGGTC

*B. albus* CCAGTCGTTGACTTAAACCTAGGGATTCTTTTTGTACTAGCCCTCTCCAGCCTGGCAGTT

*C. auripes* CCCGTTATTGACCTAAACCTTGGCATTCTATTTATTCTTGCCTTATCTAGTCTCGCAGTG

*C. melampygus* CCCGTAGCCGACCTAAACCTAGGCATTTTATTTATCCTAGCACTGTCAAGCCTCGCCGTA

*L. calcarifer* CCAGTAGCCGACCTAAACCTGGGAATTTTATTTATCCTAGCGCTGTCCAGCCTCGCAGTA

*S. maena* CCCGTTATTGACCTCAACCTCGGGGTCCTTTTTATCTTGGCCCTCTCTAGCCTCGCAGTT

*P. auriga* TCAGTTATTGACCTTAACCTAGGGATTCTTTTCATCCTTGCCCTCTCTAGTCTTGCAGTA

*C. lucidus* CCCATTATTGACCTCAACCTTAGCATTTTATTTATTTTAGCCCTCTCTAGCCTCGCCGTT

*S. sihama* CCCGTTGTAGACCTCAACCTTGGCATCCTCTTCATCCTCGCACTATCCAGCCTCGCTGTG

*C. loricula* GCAGTGATTGACCTCAACTTAGGAGTACTGTTTATCCTTGCCCTATCAAGCCTTGCAGTG

*A. trutta* CCCGTTACAGACCTTAACCTAGGCATTCTATTTATCCTTGCCCTTTCAAGCCTGGCAGTC

*H. gemma* CCCGTAGTTGATCTTAATTTAGGTGTCCTTTTCATTTTAGCTCTTTCAAGTCTCGCAGTT

*P. flavescens* CCTGTATTTGACCTTAACCTAGGAATCTTATTCCTATTAGCCCTATCAAGCATGGCCGTA

*P. macrolepida* CCTGTTGTAGATCTCAACCTCGGGATTTTATTTCTTCTAGCACTATCTAGCCTGGCCGTT

*S. canadensis* CCCGTTATTGACCTTAACCTTGGAATCTTATTCCTCCTGGCCCTATCAAGTCTAGCAGTC

*A. rogaa* TATTCCATCCTAGGTTCAGGATGAGCATCAAATTCAAAATATGCCCTAATCGGGGCCCTA

*C. argus* TACTCCATTTTAGGCTCAGGTTGAGCATCAAATTCAAAATATGCCCTGATCGGAGCCTTG

*C. sonnerati* TATTCAATTCTAGGCTCAGGCTGAGCATCCAATTCAAAATATGCCCTAATCGGAGCCTTA

*E. fuscoguttatus* TACTCCATCCTAGGTTCAGGCTGAGCATCAAATTCAAAATATGCCCTCATCGGAGCACTA

*E. coioides* TACTCTATCTTAGGCTCAGGCTGAGCATCAAATTCAAAATACGCCCTCATTGGGGCACTA

*E. bruneus* TACTCCATCCTAGGCTCAGGATGAGCATCAAATTCAAAATATGCCCTCATCGGGGCACTA

*E. moara* TACTCCATCCTAGGCTCAGGATGAGCATCAAATTCAAAATATGCCCTCATCGGGGCACTA

*E. lanceolatus*  TACTCAATCCTAGGCTCAGGCTGAGCCTCAAATTCAAAATATGCTCTCATTGGAGCACTG

*A. leucogrammicus*  TACTCTATTCTAGGTTCAGGCTGAGCATCAAATTCAAAATATGCTCTCATTGGAGCACTT

*C. altivelis* TACTCTATCCTAGGCTCAGGCTGAGCATCAAATTCAAAATATGCCCTCATCGGAGCACTA

*E. epistictus* TACTCCATTCTAGGCTCAGGCTGAGCATCAAATTCAAAATATGCTCTCATTGGAGCCCTA

*E. octofasciatus* TACTCTATTCTAGGTTCAGGCTGAGCATCAAATTCAAAATATGCTCTCATCGGGGCCCTA

*E. septemfasciatus* TACTCTATTCTAGGCTCAGGCTGAGCATCAAATTCAAAATATGCCCTCATCGGGGCCCTA

*T. dermopterus* TACTCCATCCTAGGCTCAGGATGAGCATCAAATTCAAAATACGCCCTCATTGGGGCCTTA

*E. awoara* TACTCCATTTTAGGGTCAGGTTGAGCATCCAACTCAAAATATGCCCTCATTGGGGCCCTA

*E. akaara* TATTCCATTCTAGGCTCAGGTTGAGCATCCAACTCAAAATATGCCCTCATCGGGGCCCTA

*E. trimaculatus* TATTCCATCTTAGGTTCAGGCTGAGCATCCAACTCAAAATATGCCCTCATTGGGGCCTTA

*E. areolatus* TATTCTATTTTAGGTTCAGGCTGAGCATCCAATTCAAAATATGCACTCATCGGGGCCCTC

*V. albimarginata* TATTCAATTTTAGGTTCAGGATGAGCCTCAAATTCAAAATATGCCCTTATCGGAGCCCTG

*V. louti* TACTCAATTCTAGGCTCAGGATGAGCCTCAAATTCAAAATATGCCCTTATTGGGGCCTTA

*P. leopardus* TATTCCATCCTAGGCTCAGGATGAGCATCCAACTCAAAATACGCCCTAATCGGAGCCCTC

*P. areolatus* TACTCCATCCTAGGCTCAGGATGAGCATCCAACTCAAAATACGCTCTAATCGGGGCCCTC

*E. radiosum* TACTCAATTCTAGGCTCAGGCTGAGCCTCTAATTCAAAGTATGCTCTTATTGGCGCTTTG

*P. sieboldi* TATTCTATTCTTGGCTCAGGATGGGCCTCAAATTCCAAATATGCCCTGATCGGGGCCCTA

*E. armatus* TACTCTATTTTAGGCTCAGGTTGAGCATCCAATTCAAAATATGCCCTCATTGGAGCGCTA

*R. oxyrhynchus* TACTCAATCCTGGGCTCTGGCTGAGCTTCAAATTCAAAATATGCCCTAATTGGAGCCCTC

*K. cinerascens* TACTCAATTTTAGGATCAGGATGAGCATCCAATTCAAAATACGCCCTAATTGGGGCACTC

*T. chatareus* TACTCAATTCTAGGCTCAGGATGAGCATCAAATTCAAAATATGCCCTTATCGGAGCACTC

*D. berycoides* TACTCAATCCTAGGGTCAGGGTGGGCCTCCAATTCAAAATATGCTCTCATTGGTGCCTTA

*H. typus* TACTCAATCTTAGGTTCCGGCTGAGCCTCAAACTCAAAATACGCCCTCATTGGCGCCCTA

*M. argenteus* TATTCTATCCTAGGCTCAGGCTGAGCTTCAAATTCAAAATACGCCCTCATCGGTGCCCTG

*S. chuatsi* TATTCAATTCTAGGCTCAGGCTGAGCATCAAATTCAAAATACGCCCTAATCGGTGCCCTT

*O. fasciatus* TATTCAATCCTCGGATCAGGCTGAGCCTCAAATTCAAAATATGCCCTAATCGGGGCCTTA

*P. trilineatum* TACTCAATTCTAGGATCAGGCTGAGCATCCAACTCAAAATATGCCCTCATTGGGGCCCTA

*M. salmoides* TACTCAATTTTAGGCTCAGGATGAGCATCAAATTCTAAGTATGCCCTCATCGGGGCCCTA

*P. tile* TATTCAATCTTAGGCTCAGGGTGAGCATCAAATTCAAAATACGCACTAATCGGGGCCCTA

*L. argentimaculatus* TATTCCATTCTAGGGTCAGGATGAGCATCAAACTCAAAATATGCACTAATCGGGGCTCTC

*E. struhsakeri* TACTCTATTCTAGGGTCAGGCTGAGCATCCAACTCAAAATATGCCCTCATTGGTGCCCTG

*B. albus* TATTCAATCCTAGGGTCAGGATGGGCATCCAACTCAAAATACGCCCTCATTGGGGCACTA

*C. auripes* TACTCAATTCTAGGGTCAGGCTGAGCATCCAACTCAAAATACGCACTCATTGGGGCCTTA

*C. melampygus* TATTCAATTTTAGGCTCAGGTTGAGCATCAAATTCAAAATACGCCCTAGTTGGGGCCCTC

*L. calcarifer* TACTCAATCCTCGGCTCAGGCTGAGCATCCAACTCAAAATACGCACTAATCGGAGCCCTT

*S. maena* TATTCTATTCTTGGGTCAGGATGGGCATCCAATTCAAAATATGCCCTTATTGGAGCCTTA

*P. auriga* TACTCTATCCTTGGATCAGGATGAGCATCAAACTCAAAATATGCCCTCATCGGGGCCCTA

*C. lucidus* TACTCAATCCTCGGATCAGGCTGAGCATCCAACTCAAAATATGCACTCATCGGAGCTCTT

*S. sihama* TACTCTATTTTAGGCTCTGGCTGAGCCTCAAATTCTAAATACGCACTTATTGGTGCCCTC

*C. loricula* TATTCTATTTTGGGCTCAGGCTGAGCCTCAAATTCAAAATATGCTCTCATTGGGGCCCTG

*A. trutta* TACTCAATCCTCGGATCAGGCTGAGCATCAAATTCAAAATACGCCCTCATCGGAGCCCTA

*H. gemma* TATTCAATTTTAGGTTCTGGATGAGCATCAAATTCAAAGTATGCATTGATTGGAGCTCTA

*P. flavescens* TATTCCATTTTAGGCTCAGGCTGAGCATCCAACTCTAAATATGCCCTCATTGGAGCCCTG

*P. macrolepida* TATTCAATTTTAGGCTCAGGGTGGGCCTCCAATTCAAAATATGCCCTAATCGGCGCGCTG

*S. canadensis* TACTCAATTTTAGGCTCAGGATGGGCCTCCAATTCCAAATATGCATTAATTGGTGCCTTA

*A. rogaa* CGAGCCGTAGCCCAAACTATTTCATACGAAGTTAGCCTTGGGTTAATCCTATTAAATGCC

*C. argus* CGAGCCGTAGCACAAACCATTTCATATGAAGTTAGTCTTGGATTAATCTTGCTAAATGCC

*C. sonnerati* CGAGCCGTAGCACAAACCATCTCCTACGAGGTTAGCCTAGGCCTAATCCTCCTAAACGCT

*E. fuscoguttatus* CGAGCCGTAGCACAAACCATTTCCTATGAAGTTAGCCTTGGATTAATCCTTTTAAACGCA

*E. coioides* CGAGCCGTAGCACAAACTATCTCCTACGAAGTCAGCCTAGGCTTAATTCTTTTAAACGCA

*E. bruneus* CGAGCCGTAGCACAAACTATTTCCTACGAAGTCAGCCTAGGATTAATTCTTTTAAATGCA

*E. moara* CGAGCCGTAGCACAAACTATTTCCTACGAGGTCAGCCTAGGATTAATTCTTTTAAATGCA

*E. lanceolatus*  CGAGCCGTAGCACAAACTATCTCCTATGAAGTCAGCCTAGGCCTAATTCTTTTAAACGCA

*A. leucogrammicus*  CGAGCTGTAGCACAAACCATTTCCTACGAAGTTAGCCTAGGATTAATCCTTCTAAACGCA

*C. altivelis* CGAGCCGTAGCACAAACTATTTCCTACGAGGTCAGCCTAGGACTAATCCTTTTAAACGCA

*E. epistictus* CGGGCCGTAGCACAAACTATTTCATATGAAGTTAGCCTAGGATTAATCCTTTTAAACGCA

*E. octofasciatus* CGAGCCGTAGCACAAACTATTTCCTACGAAGTTAGCCTAGGACTAATCCTCCTAAATGCA

*E. septemfasciatus* CGAGCCGTAGCACAAACTATTTCCTACGAAGTCAGCCTAGGACTAATCCTCCTAAATGCA

*T. dermopterus* CGCGCCGTTGCACAAACTATTTCATACGAAGTCAGCCTAGGACTGATCCTTTTAAACGCA

*E. awoara* CGAGCTGTAGCGCAGACTATCTCATATGAAGTTAGCCTAGGGCTTATTCTTTTAAACGCA

*E. akaara* CGAGCTGTAGCACAAACTATTTCATATGAAGTCAGCCTGGGACTAATCCTCTTAAACGCA

*E. trimaculatus* CGAGCTGTAGCACAAACTATTTCATACGAAGTTAGCCTAGGCCTAATCCTTCTAAACGCA

*E. areolatus* CGAGCTGTGGCACAAACCATTTCTTACGAAGTTAGCCTAGGACTAATCCTTCTAAATGCA

*V. albimarginata* CGGGCCGTTGCCCAAACTATTTCATACGAAGTCAGCTTAGGTCTTATCCTTCTAAATATT

*V. louti* CGGGCCGTTGCCCAAACTATTTCATATGAAGTCAGCTTAGGTCTTATCCTTCTAAATATT

*P. leopardus* CGTGCCGTAGCACAGACTATCTCATATGAAGTCAGCCTTGGATTAATCTTACTAAACGCT

*P. areolatus* CGTGCCGTAGCACAGACTATCTCATATGAGGTCAGCCTTGGACTAATTTTACTAAACGCT

*E. radiosum* CGTGCCGTAGCTCAGACAATTTCCTATGAGGTTAGCCTAGGATTAATTCTTTTGAACATC

*P. sieboldi* CGAGCCGTAGCTCAAACCATCTCCTATGAGGTGAGCCTCGGACTAATCCTGCTAAATATT

*E. armatus* CGGGCAGTAGCCCAAACCATCTCCTACGAAGTTAGTCTTGGACTTATTCTTTTAAATGCT

*R. oxyrhynchus* CGAGCCGTTGCACAAACCATCTCGTACGAAGTAAGTATAGGACTAATCCTCCTCTGCGTT

*K. cinerascens* CGAGCTGTGGCCCAAACCATTTCATACGAAGTCAGTCTAGGACTCATCCTTTTAAACGCC

*T. chatareus* CGAGCCGTGGCCCAAACCATCTCCTACGAAGTAAGTCTCGGCCTCATCCTCCTAAACGCT

*D. berycoides* CGAGCCGTCGCCCAAACCATCTCATATGAAGTTAGCCTAGGGCTCATCCTTCTAAATGCC

*H. typus* CGAGCCGTTGCCCAAACTATTTCATACGAAGTCAGCCTGGGGCTCATCCTCCTAAATGCC

*M. argenteus* CGGGCCGTAGCCCAAACCATTTCCTATGAGGTTAGCTTGGGATTAATTCTCCTCAACGCC

*S. chuatsi* CGAGCCGTAGCGCAAACCATCTCATACGAAGTTAGCCTCGGACTCATCCTCTTAAACGCC

*O. fasciatus* CGAGCCGTAGCCCAAACCATTTCATATGAAGTTAGCCTCGGACTCATTCTCTTAAACGCT

*P. trilineatum* CGAGCCGTAGCCCAAACCATTTCATATGAAGTTAGCCTAGGATTAATCCTCCTAAACATT

*M. salmoides* CGGGCCGTAGCCCAGACTATTTCATATGAAGTTAGCCTTGGGCTAATCCTCTTAAATACC

*P. tile* CGGGCCGTTGCCCAAACCATTTCCTACGAAGTAAGCCTAGGCTTGATTCTTCTCAATGCT

*L. argentimaculatus* CGAGCTGTTGCCCAAACCATCTCCTACGAAGTCAGCCTCGGACTAATTCTACTTAATGCC

*E. struhsakeri* CGTGCCGTAGCCCAAACCATCTCGTATGAAGTAAGCCTAGGCCTTATCCTTCTAAACGCT

*B. albus* CGAGCCGTAGCCCAAACCATCTCGTATGAAGTCAGCCTAGGACTAATCCTTCTAAACACT

*C. auripes* CGGGCAGTAGCCCAAACCATCTCATATGAAGTCAGCCTAGGACTAATTCTACTAAACATT

*C. melampygus* CGAGCCGTAGCCCAAACCATTTCATATGAAGTTAGCCTCGGCCTAATTCTCCTAAACATT

*L. calcarifer* CGAGCCGTAGCCCAAACTATTTCATATGAAGTAAGCCTAGGACTTATCCTCCTATGCACC

*S. maena* CGGGCCGTCGCCCAAACCATTTCTTATGAAGTAAGCCTCGGACTTATCCTCCTATCAGCA

*P. auriga* CGAGCTGTAGCCCAGACTATTTCATATGAAGTAAGCCTTGGACTTATTCTTCTATGCACA

*C. lucidus* CGGGCCGTCGCTCAAACCATCTCGTATGAAGTGAGCCTGGGCTTAATTTTACTATGCACC

*S. sihama* CGAGCCGTCGCTCAAACTATTTCGTATGAAGTCAGCCTCGGATTAATTCTTCTTTGCGTA

*C. loricula* CGAGCCGTAGCCCAAACTATTTCTTATGAAGTAAGTCTAGGGTTAATTCTCTTAAGCGCA

*A. trutta* CGAGCTGTAGCCCAGACAATCTCCTATGAAGTCAGCCTTGGCCTCATTCTCCTTAATATC

*H. gemma* CGAGCAGTAGCCCAAACTATTTCATATGAAGTTAGTCTAGGCCTTATTCTCCTTGCAGTA

*P. flavescens* CGAGCCGTCGCCCAGACAATTTCTTATGAGGTTAGTCTTGGCCTAATTCTTTTAAATGTA

*P. macrolepida* CGTGCTGTTGCCCAGACAATTTCCTATGAAGTAAGCCTAGGACTAATTCTCTTAAACATC

*S. canadensis* CGGGCCGTGGCCCAAACAATTTCTTACGAGGTTAGCCTAGGACTTATTCTACTAAATACC

*A. rogaa* ATCATCTTTACCGGAGGCTTTACACTGCACACCTTTAACATTGCCCAAGAAGCTATCTGA

*C. argus* ATTATTTTCACTGGGGGCTTTACACTTCACACCTTCAATATCGCCCAAGAAACCATCTGA

*C. sonnerati* ATTATTTTCACCGGAGGATTTACCCTACAAACCTTTAACATCGCCCAGGAAGCAATCTGA

*E. fuscoguttatus* ATTATCTTCACAGGAGGCTTCACCCTACAAACCTTCAACACAGCCCAAGAAACCGTCTGA

*E. coioides* ATTATTTTTACAGGAGGCTTCACCCTACAAACCTTCAATACAGCTCAAGAAACTGTCTGA

*E. bruneus* ATCATCTTCACAGGAGGCTTCACCCTACAAACCTTTAACACAGCCCAAGAAACCGTCTGA

*E. moara* ATCATTTTCACAGGAGGCTTCACCCTACAAACCTTCAATACAGCCCAAGAAACCGTCTGA

*E. lanceolatus*  ATTATTTTCACAGGGGGCTTCACCCTACAAACCTTTAACACAGCCCAAGAAACCGTCTGA

*A. leucogrammicus*  ATTATCTTCACAGGAGGCTTCACCCTGCAAACCTTCAACACAGCCCAAGAAACTGTCTGA

*C. altivelis* ATTATCTTTACAGGGGGCTTCACCCTACAAACCTTTAACACAGCCCAAGAAACCGTCTGA

*E. epistictus* ATTATTTTCACAGGGGGTTTTACCCTACAAACCTTTAGCACAGCCCAAGAAGCCATCTGA

*E. octofasciatus* ATTATCTTTACAGGAGGTTTCACCCTACAAACCTTCAACACAGCCCAAGAAGCTATCTGA

*E. septemfasciatus* ATTATCTTTACAGGAGGTTTCACCCTGCAAACCTTCAACACAGCCCAAGAAGCTATCTGA

*T. dermopterus* ATTATTTTTACAGGGGGTTTCACCCTACAAACCTTCAATACAGCCCAAGAAGCTATCTGA

*E. awoara* ATTATTTTTACGGGCGGTTTTACACTACAAACCTTTAGCACAGCCCAAGAAGCCACCTGA

*E. akaara* ATCATTTTTACGGGCGGTTTTACCCTACAAACCTTTAGCACAGCCCAAGAAGCTACTTGA

*E. trimaculatus* ATTATTTTTACAGGAGGCTTTACCCTACAAACCTTTAGCACAGCCCAAGAAGCCACTTGA

*E. areolatus* ATTATCTTTACAGGAGGCTTCACCTTACAAACCTTTAGCACAGCCCAAGAAGCCACTTGA

*V. albimarginata* ATCATCTTCACGGGAGGGTTTACACTGCAAACCTTTAGCGTTGCTCAGGAAGCCATTTGA

*V. louti* ATTATCTTTACAGGAGGATTCACACTACAAACCTTTAACGTTGCCCAAGAAGCCGTCTGA

*P. leopardus* ATTATCTTTACAGGAGGCTTCACCCTCCAAACTTTCAACACAGCCCAAGAAGCTATTTGA

*P. areolatus* ATTATCTTCACAGGAGGCTTCACCCTTCAAACCTTCAACACCGCCCAAGAAGCTATTTGA

*E. radiosum* ATCATTTTCACCGGGGGCTTCACACTACAAACCTTTAACGTGGCCCAAGAGAGCATTTGA

*P. sieboldi* ATTATTTTTACTGGCGGGTTCACCCTACAGACTTTCAACATCGCCCAAGAGACTGTCTGA

*E. armatus* ATTATCTTTACTGGGGGATTTACATTACAAACTTTCAATGTTGCCCAGGAAAGTATCTGA

*R. oxyrhynchus* ATCCTTTTTACAGGAGGCTTCACACTACAAACCTTCAACATCACCCAAGAAAGCATCTGA

*K. cinerascens* ATTATCTTCACTGGAGGATTTACCCTACAAACCTTTAACGTGGCCCAAGAAAGCGTCTGA

*T. chatareus* ATTATCTTCACCGGGGGTTTTACCCTCCAAACCTTCAACACTGCTCAAGAAAGTGTCTGA

*D. berycoides* ATTATCTTCACTGGAGGCTTTACCCTACAAACCTTTAACGTTGCCCAAGAAAGTGTCTGA

*H. typus* ATCATCTTCACCGGGGGATTTTCGTTACAAACCTTTAATGTGGCCCAAGAAAGCATCTGG

*M. argenteus* ATCATTTTCACCGGTGGCTTTACACTTCAAACTTTTAATGTAGCCCAAGAAAGCATCTGA

*S. chuatsi* ATTATCTTCACCGGCGGCTTCACCCTGCAAACCTTCAACATTGCCCAAGAAAGCGTTTGA

*O. fasciatus* ATTATCTTTACAGGAGGCTTCACATTACAAACCTTTAATATTGCCCAAGAAAGCATCTGA

*P. trilineatum* ATTATTTTTACTGGGGGATTCACATTACAAACCTTCAATGTTGCCCAAGAAAGTGTATGA

*M. salmoides* ATTATTTTCACGGGGGGTTTTACTCTTCAAACCTTCAACGTAGCCCAAGAAAGCGTCTGA

*P. tile* ATCATTTTTACGGGAGGCTTCACACTCCAAACCTTTAACGTTGCTCAAGAGAGCATCTGA

*L. argentimaculatus* ATCATTTTTACCGGAGGCTTTACACTCCAAACCTTCAATGTTGCCCAAGAAAGTATTTGA

*E. struhsakeri* ATTATTTTTACTGGCGGATTTACGCTACAAACCTTCAATGTTGCTCAAGAAAGCATCTGA

*B. albus* ATTATCTTCACGGGAGGTTTTACACTACAAATCTTTAACGTAGCACAAGAAAGCGTCTGA

*C. auripes* ATTATTTTTACCGGAGGCTTTACACTCCATACCTTTAATGTAGCCCAAGAAAGCACATGA

*C. melampygus* ATTATCTTCACAGGCGGCTTCACCCTCCAAACCTTCAACACTGCTCAAGAAAGCATCTGA

*L. calcarifer* ATCATCTTTACAGGGGGCTTTACCCTTCAAACATTTAGCACTGCCCAAGAAGGCATTTGA

*S. maena* ATCATCTTTACAGGAGGCTTCACCCTGCAAATCTTCAACATTGCCCAAGAAAGCATTTGA

*P. auriga* GTTATCTTTACAGGAGGATTCACCCTACAAACCTTTAGCGTTGCTCAAGAAAGCATTTGA

*C. lucidus* ATTATCTTCACGGGAGGCTTCACCCTCCAAACCTTTAATATTGCCCAAGAAAGCATCTGA

*S. sihama* ATCATCTTCACCGGAGGCTTCACTCTACAAACCTTTAACGTCACCCAAGAAAGCATCTGA

*C. loricula* ATTATTTTTACCGGAGGCTTCACACTACAGACCTTTGGCACTGCTCAAGAGAGCGTGTGA

*A. trutta* ATCATCTTTACAGGGGGTTTTACACTCCAGATCTTTAACACAGCTCAAGAGGCTATCTGA

*H. gemma* ATCATCGTTGCGGGAGGATTTACACTCCAGACTTTTAATATTGCCCAAGAAAGTATCTGA

*P. flavescens* ATTATTTTCACTGGGGGCTTTACCCTACAAACCTTTAACGTAGCCCAAGAAAGCGTCTGA

*P. macrolepida* ATCATTTTTACAGGAGGCTTTACCCTACAAACCTTCAACGTAGCCCAAGAAAGTATTTGA

*S. canadensis* ATTATTTTTACAGGCGGCTTCACACTACAAACCTTTAACGTAGCTCAAGAGAGCGTTTGG

*A. rogaa* CTTCTACTACCAGCCTGACCTCTAGCCGCAATATGGTATATTTCCACCCTAGCAGAAACC

*C. argus* TTACTACTACCGGCCTGACCTCTCGCTGCAATGTGATACATTTCTACCCTAGCAGAGACT

*C. sonnerati* CTACTACTGCCAGCCTGACCCTTGGCCGCAATATGATACATTTCCACTTTAGCAGAAACT

*E. fuscoguttatus* CTTCTACTACCAGCCTGACCCCTAGCCGCAATATGGTATATTTCCACACTAGCCGAAACC

*E. coioides* CTCCTATTACCAACCTGACCCCTAGCCGCAATATGATATATTTCTACATTAGCCGAAACA

*E. bruneus* CTTCTACTACCAGCCTGACCTCTGGCCGCAATATGGTACATCTCCACATTAGCCGAAACT

*E. moara* CTTCTACTACCAGCCTGACCTCTAGCCGCAATATGGTACATCTCCACATTAGCCGAAACT

*E. lanceolatus*  CTTTTACTACCAGCATGACCCCTGGCCGCAATATGATATATCTCTACATTAGCCGAGACT

*A. leucogrammicus*  CTTCTACTACCAACTTGACCTCTAGCTGCAATATGATACATCTCCACACTAGCCGAAACC

*C. altivelis* CTCCTTTTACCAGCCTGACCTCTAGCCGCAATGTGGTATATCTCCACATTAGCCGAAACC

*E. epistictus* CTCCTACTACCAACCTGACCTCTAGCCGCAATATGATACATCTCTACATTAGCAGAAACC

*E. octofasciatus* CTTCTACTACCAGCCTGACCTCTAGCTGCAATATGATACATTTCCACCCTAGCAGAAACC

*E. septemfasciatus* CTTCTATTACCAACCTGACCTCTAGCTGCAATATGATACATCTCCACCCTAGCAGAAACC

*T. dermopterus* CTCCTACTGCCAACCTGACCTCTGGCCGCAATATGATATATCTCTACCTTAGCAGAAACC

*E. awoara* CTTCTCCTACCAGCCTGACCTTTAGCCGCAATATGATACATCTCCACACTAGCAGAAACT

*E. akaara* CTCCTCCTGCCAGCCTGACCTTTAGCCGCAATATGATACATTTCCACACTAGCAGAAACT

*E. trimaculatus* CTTCTACTACCAGCCTGACCATTAGCTGCAATATGGTATATTTCCACACTAGCAGAAACT

*E. areolatus* CTACTACTACCAGCCTGACCTCTAGCTGCAATATGATATATTTCTACACTAGCAGAAACT

*V. albimarginata* ATACTCCTGCCTGCCTGGCCATTAGCCGCGATATGGTATATCTCTACCCTAGCGGAGACT

*V. louti* ATACTCTTACCGGCCTGACCACTAGCTGCAATATGGTATATTTCCACCTTAGCAGAAACT

*P. leopardus* CTAATCTTGCCAACCTGACCATTAGCTGGGATATGATACATTTCAACCCTTGCAGAAACA

*P. areolatus* CTAATTTTACCAACTTGACCATTAGCTGGAATATGATACATCTCAACCCTTGCAGAGACA

*E. radiosum* CTAATTATGCCCGCTTGACCCCTTGCCGCAATATGGTATATTTCTACGCTTGCAGAAACC

*P. sieboldi* CTAGTTCTACCTGCCTGACCCCTAGCGGCAATATGATATATCTCAACCTTAGCCGAGACC

*E. armatus* CTAGTTCTGCCGGCCTGACCCCTAGCTGCTATATGATATATCTCAACCTTAGCAGAAACC

*R. oxyrhynchus* CTTATTCTACCCGCCTGACCTCTTGCGGCAATGTGATACATTTCAACACTAGCAGAAACA

*K. cinerascens* CTAATTTTACCAGCCTGACCATTAGCCGCAATATGGTATATCTCAACCCTAGCAGAAACC

*T. chatareus* TTAATTCTGCCTGCCTGACCCCTAGCTGCAATATGATACATCTCCACCCTAGCTGAGACC

*D. berycoides* TTAATCTTACCAGCCTGACCACTAGCCGCAATATGATATATCTCAACTTTAGCAGAAACC

*H. typus* TTGATCTTACCAGCCTGACCCCTAGCCGCAATATGATATATCTCAACTTTAGCAGAAACC

*M. argenteus* TTAATCCTACCAGCCTGACCTTTAGCCGCAATATGATACATTTCAACACTAGCAGAAACC

*S. chuatsi* TTGATCTTACCAGCCTGGCCTTTAGCCGCTATATGATACATTTCAACCCTAGCAGAAACC

*O. fasciatus* CTAATCTTACCAGCTTGACCCCTAGCTGCAATATGATACATCTCAACCTTAGCAGAAACT

*P. trilineatum* CTAGTCCTCCCAGCTTGACCCCTAGCCGCAATGTGATATATTTCAACCTTAGCAGAAACT

*M. salmoides* CTAATTTTTCCAGCCTGACCCCTAGCTGCCATATGATATATTTCAACCCTGGCCGAAACT

*P. tile* TTAATTCTTCCAGCTTGGCCCCTCGCTGCAATATGATATATTTCTACCCTAGCAGAAACC

*L. argentimaculatus* CTAATTATACCCGCCTGACCTCTCGCCGCAATATGGTACATTTCCACACTAGCAGAAACC

*E. struhsakeri* TTAATTCTCCCAGCCTGACCTTTAGCCGCAATATGATACATCTCAACTTTAGCAGAAACC

*B. albus* CTAATTCTACCTGCCTGACCTCTCGCCGCAATATGGTATATTTCAACACTAGCAGAGACC

*C. auripes* CTGATCTTGCCCGCCTGACCTTTAGCTGCAATATGGTATATTTCAACACTAGCCGAGACA

*C. melampygus* CTTGTAATGCCAGCATGACCCCTAGCCGCCATATGATATATTTCAACACTAGCAGAAACC

*L. calcarifer* CTGATCCTCCCCGCCATACCACTAGCTGCCATATGGTACATTTCCACACTAGCAGAGACA

*S. maena* CTTATCCTCCCAGCATGACCCCTAGCCGCAATGTGATACGTCTCTACACTCGCAGAAACT

*P. auriga* CTAATTATTCCAACATGACCCCTAGCCGCAATATGATATATTTCTACGCTAGCAGAGACC

*C. lucidus* CTGATTCTCCCTGCCTGACCTCTAGCCGCAATATGATACATTTCTACACTTGCAGAAACC

*S. sihama* CTCCTTGTGCCTGCATGACCCCTAGCCGTAATATGATATATTTCAACCCTAGCAGAAACT

*C. loricula* CTAATCTTACCAGCCTGACCCTTAGCAGCCATATGATATATTTCAACACTAGCAGAGACC

*A. trutta* TTAATTATCCCCACTTGGCCACTCGCCGCAATATGATATATTTCAACTTTGGCTGAAACC

*H. gemma* CTCATTCTTCCCTCCTGACCCTTAGCAGCAATATGATATATTTCTACGCTGGCAGAAACT

*P. flavescens* TTAATCCTCCCAGCCTGGCCACTCGCCGCCATATGATATATCTCGACCCTCGCAGAAACA

*P. macrolepida* TTAATTGCACCCGCCTGACCCCTTGCCGCAATATGATATATTTCGACCCTCGCAGAAACC

*S. canadensis* TTAATTTTACCAGCCTGGCCCCTAGCCGCAATATGGTATATCTCTACCCTCGCAGAGACT

*A. rogaa* AACCGAGCACCTTTTGATTTAACAGAAGGTGAATCGGAACTGGTGTCAGGTTTTAATGTA

*C. argus* AACCGAGCACCTTTTGACCTAACAGAAGGTGAATCGGAACTGGTATCAGGCTTCAACGTC

*C. sonnerati* AACCGAGCACCCTTCGACCTAACCGAAGGTGAATCAGAGTTAGTCTCAGGTTTTAACGTA

*E. fuscoguttatus* AACCGAGCACCTTTCGACTTAACCGAAGGCGAATCAGAACTAGTCTCAGGCTTTAACGTA

*E. coioides* AACCGAGCACCCTTCGACTTAACCGAAGGCGAATCAGAACTAGTCTCAGGCTTTAATGTA

*E. bruneus* AACCGAGCACCTTTCGACTTAACCGAAGGCGAGTCAGAACTAGTCTCAGGCTTCAATGTA

*E. moara* AACCGAGCACCTTTCGACTTAACCGAAGGTGAGTCAGAGCTAGTTTCAGGCTTCAATGTA

*E. lanceolatus*  AACCGAGCACCCTTCGACTTAACTGAAGGTGAATCAGAACTAGTATCAGGTTTTAATGTA

*A. leucogrammicus*  AACCGAGCACCTTTCGACCTAACTGAAGGTGAATCAGAACTAGTCTCAGGCTTCAATGTA

*C. altivelis* AACCGAGCACCCTTTGATTTAACTGAAGGAGAGTCAGAACTAGTATCAGGTTTTAATGTA

*E. epistictus* AACCGAGCACCCTTCGACCTAACTGAAGGCGAATCAGAATTAGTCTCGGGTTTTAACGTA

*E. octofasciatus* AACCGAGCACCCTTCGATCTAACCGAAGGTGAGTCAGAACTAGTTTCAGGCTTCAACGTA

*E. septemfasciatus* AACCGAGCACCCTTCGATTTAACCGAAGGTGAATCAGAACTAGTTTCAGGCTTCAACGTA

*T. dermopterus* AACCGAGCACCATTCGATTTAACCGAGGGTGAATCAGAGCTAGTTTCAGGCTTTAACGTA

*E. awoara* AACCGGGCCCCCTTCGACTTAACTGAAGGGGAATCAGAGCTAGTCTCAGGCTTTAACGTA

*E. akaara* AACCGAGCACCCTTCGACCTGACTGAAGGAGAGTCGGAACTAGTCTCAGGCTTCAACGTA

*E. trimaculatus* AACCGAGCACCTTTTGACTTAACCGAAGGAGAATCAGAGCTAGTTTCCGGCTTTAACGTA

*E. areolatus* AACCGAGCACCATTTGACCTAACTGAAGGAGAGTCAGAGCTAGTCTCTGGCTTCAACGTA

*V. albimarginata* AACCGAGCACCCTTCGATTTAACTGAGGGTGAATCAGAGCTAGTTTCAGGATTCAACGTA

*V. louti* AACCGAGCACCCTTTGATTTAACTGAGGGTGAGTCAGAACTAGTTTCAGGCTTTAATGTA

*P. leopardus* AACCGTGCACCTTTCGACCTCACCGAAGGGGAATCGGAACTAGTTTCAGGCTTCAATGTA

*P. areolatus* AACCGTGCACCTTTCGATCTCACTGAAGGTGAGTCAGAACTAGTTTCAGGCTTTAATGTG

*E. radiosum* AATCGTGCACCCTTTGATCTTACTGAGGGGGAGTCAGAGCTAGTCTCGGGTTTTAATGTT

*P. sieboldi* AACCGTGCCCCCTTTGATCTAACAGAAGGTGAGTCCGAGCTGGTGTCGGGCTTTAACGTT

*E. armatus* AATCGTGCCCCTTTTGATTTAACAGAAGGGGAATCGGAACTAGTCTCAGGCTTTAATGTA

*R. oxyrhynchus* AACCGTGCCCCCTTTGATTTAACTGAAGGGGAGTCCGAACTAGTCTCCGGGTTCAATGTA

*K. cinerascens* AACCGTGCCCCCTTCGATCTCACAGAAGGAGAATCTGAACTAGTCTCAGGCTTTAATGTA

*T. chatareus* AATCGTGCCCCCTTCGACCTAACCGAGGGCGAATCTGAACTAGTCTCAGGCTTTAACGTA

*D. berycoides* AACCGTGCCCCCTTCGACCTGACCGAAGGTGAGTCCGAGCTAGTCTCAGGCTTTAACGTA

*H. typus* AACCGTGCCCCCTTTGACCTAACTGAAGGAGAATCAGAACTAGTCTCAGGCTTTAACGTA

*M. argenteus* AATCGGGCCCCTTTTGACCTCACCGAGGGGGAATCTGAGCTAGTCTCAGGATTTAATGTA

*S. chuatsi* AATCGAGCACCGTTCGACCTAACTGAAGGTGAATCAGAACTAGTATCAGGCTTCAACGTA

*O. fasciatus* AACCGTGCCCCCTTCGACTTAACTGAAGGCGAATCCGAACTAGTTTCAGGCTTCAACGTA

*P. trilineatum* AACCGTGCCCCCTTTGACCTCACGGAAGGTGAATCTGAATTAGTCTCAGGCTTCAATGTT

*M. salmoides* AACCGCGCCCCTTTCGATTTAACTGAGGGCGAGTCTGAACTAGTCTCAGGTTTTAATGTA

*P. tile* AACCGTGCCCCCTTTGACCTCACTGAAGGAGAATCTGAACTAGTCTCAGGCTTCAACGTA

*L. argentimaculatus* AACCGTGCCCCCTTTGACCTCACAGAAGGAGAATCCGAACTAGTCTCAGGCTTTAACGTA

*E. struhsakeri* AACCGTGCCCCCTTTGACCTCACCGAAGGTGAATCTGAGCTAGTATCGGGCTTCAATGTA

*B. albus* AACCGTGCCCCCTTTGACCTCACAGAAGGAGAATCAGAACTAGTTTCCGGCTTCAACGTA

*C. auripes* AACCGTGCCCCCTTTGATCTGACTGAGGGCGAATCCGAGCTTGTCTCAGGTTTCAATGTA

*C. melampygus* AACCGAGCCCCCTTCGACCTAACTGAAGGAGAATCCGAACTAGTCTCTGGCTTTAACGTA

*L. calcarifer* AACCGCGCCCCGTTCGACCTCACTGAAGGAGAATCAGAACTTGTCTCAGGCTTTAACGTA

*S. maena* AACCGAGCCCCCTTTGACCTAACCGAAGGTGAGTCCGAACTCGTTTCTGGTTTTAATGTT

*P. auriga* AACCGAGCCCCATTCGACCTTACTGAAGGAGAGTCCGAACTAGTATCCGGGTTCAACGTC

*C. lucidus* AACCGAGCCCCCTTCGACCTCACTGAAGGCGAATCCGAACTCGTTTCAGGCTTCAATGTA

*S. sihama* AATCGAGCCCCCTTTGACCTAACAGAAGGAGAATCCGAACTAGTTTCGGGCTTTAACGTA

*C. loricula* AACCGGGCCCCCTTTGACCTCACAGAAGGAGAATCTGAGCTAGTCTCGGGATTTAACGTA

*A. trutta* AACCGAGCCCCTTTCGACCTTACTGAAGGTGAATCAGAACTAGTCTCAGGGTTCAATGTT

*H. gemma* AATCGAGCACCCTTCGATTTGACTGAGGGAGAATCTGAACTAGTTTCAGGATTCAATGTA

*P. flavescens* AACCGTGCCCCCTTCGATCTCACCGAAGGTGAGTCAGAACTGGTCTCAGGGTTTAATGTC

*P. macrolepida* AATCGTGCCCCCTTTGACCTTACGGAGGGGGAATCAGAACTAGTCTCAGGGTTTAACGTT

*S. canadensis* AACCGTGCACCTTTTGATCTCACCGAAGGAGAGTCAGAACTAGTCTCAGGGTTTAATGTG

*A. rogaa* GAATACGCAGGAGGACCTTTCGCCCTATTTTTCTTAGCAGAATACGCAAATATTCTTCTT

*C. argus* GAATACGCAGGGGGGCCTTTCGCCCTGTTTTTCCTAGCAGAATACGCGAACATCCTCCTC

*C. sonnerati* GAATACGCAGGAGGTCCTTTCGCCCTATTCTTTCTCGCAGAATATGCAAACATCCTCCTA

*E. fuscoguttatus* GAATATGCGGGAGGCCCTTTCGCCCTATTCTTTTTAGCAGAATACGCAAACATCCTCCTT

*E. coioides* GAATATGCAGGAGGCCCTTTCGCCTTGTTTTTCTTAGCAGAATACGCAAACATCCTCCTT

*E. bruneus* GAGTACGCAGGAGGCCCTTTCGCCCTATTTTTCCTGGCAGAATACGCAAACATTCTCCTT

*E. moara* GAGTACGCAGGGGGCCCTTTCGCTCTATTTTTCCTGGCAGAATACGCAAACATCCTCCTT

*E. lanceolatus*  GAATACGCAGGGGGTCCTTTCGCCCTATTCTTCCTAGCAGAGTACGCAAACATCCTTCTT

*A. leucogrammicus*  GAATACGCAGGAGGCCCTTTCGCCCTATTCTTTCTAGCAGAGTATGCAAATATCCTCCTT

*C. altivelis* GAATACGCAGGAGGCCCCTTTGCCCTATTCTTCCTGGCAGAGTACGCAAACATCCTCCTT

*E. epistictus* GAATATGCAGGAGGCCCTTTCGCCCTATTTTTTCTAGCAGAGTATGCAAATATTCTTTTT

*E. octofasciatus* GAGTATGCAGGGGGCCCCTTTGCCCTATTTTTCCTAGCAGAATACGCAAACATCCTTCTC

*E. septemfasciatus* GAATATGCAGGTGGCCCCTTTGCCCTATTTTTCCTAGCAGAATACGCAAACATTCTTCTC

*T. dermopterus* GAATATGCAGGAGGCCCTTTTGCCCTATTTTTTTTAGCAGAATACGCAAACATTCTCCTC

*E. awoara* GAATATGCAGGGGGCCCCTTCGCCTTATTTTTCCTGGCAGAATACGCAAACATTCTCCTT

*E. akaara* GAATATGCAGGAGGTCCCTTCGCCCTATTTTTCCTAGCAGAATACGCAAATATTCTCCTT

*E. trimaculatus* GAATATGCAGGAGGCCCTTTCGCCCTATTTTTCTTAGCAGAATACGCAAACATTCTCCTT

*E. areolatus* GAATACGCAGGAGGGCCCTTCGCTTTATTTTTCCTGGCAGAATATGCAAATATTCTTCTT

*V. albimarginata* GAGTATGCTGGAGGACCCTTTGCCCTATTCTTCTTAGCAGAATACGCAAATATTTTACTA

*V. louti* GAATATGCCGGAGGACCCTTTGCCCTCTTCTTCCTAGCAGAGTACGCAAACATTTTACTA

*P. leopardus* GAATATGCAGGGGGCCCTTTCGCTTTATTCTTTTTAGCGGAATACTCAAACATCCTTCTT

*P. areolatus* GAATATGCAGGAGGCCCTTTCGCCTTATTCTTCTTAGCAGAGTACTCAAATATTCTACTC

*E. radiosum* GAATACGCAGGGGGCCCTTTCGCTTTGTTTTTCCTGGCAGAATACGCGAATATTTTACTT

*P. sieboldi* GAGTACGCGGGCGGGCCCTTCGCGCTATTTTTCCTAGCCGAGTACGCAAATATTCTACTC

*E. armatus* GAATATGCAGGAGGGCCCTTCGCACTCTTTTTCCTAGCAGAGTATGCCAACATTTTACTT

*R. oxyrhynchus* GAGTATGCAGGAGGACCCTTCGCTCTATTTTTCCTGGCAGAATACGCCAACATTCTCCTC

*K. cinerascens* GAATATGCAGGAGGACCTTTTGCTTTATTTTTCCTGGCAGAATACGCCAACATCCTTCTA

*T. chatareus* GAGTACGCAGGAGGGCCCTTTGCCTTATTCTTCCTAGCAGAATATTCCAACATCCTATTA

*D. berycoides* GAATATGCAGGGGGCCCCTTCGCCCTATTTTTCCTGGCAGAATATGCTAACATCCTACTC

*H. typus* GAATATGCCGGAGGCCCATTCGCCCTGTTCTTCCTAGCAGAATACGCCAACATCCTACTC

*M. argenteus* GAATATGCAGGGGGGCCCTTTGCCCTATTTTTCCTGGCAGAATACGCCAACATCTTACTT

*S. chuatsi* GAATATGCAGGAGGCCCCTTTGCCCTATTCTTCCTAGCAGAATATGCCAACATCTTACTT

*O. fasciatus* GAATACGCAGGAGGCCCTTTCGCCCTATTCTTTCTAGCAGAATACGCCAATATTTTACTT

*P. trilineatum* GAGTACGCAGGTGGCCCCTTCGCCTTATTCTTCCTGGCAGAATACGCTAACATCCTACTT

*M. salmoides* GAGTACGCAGGAGGGCCCTTTGCCCTCTTTTTCCTGGCAGAATATGCCAACATTTTACTT

*P. tile* GAATACGCAGGAGGGCCCTTCGCCCTATTTTTCTTAGCAGAGTACGCTAATATTCTCCTC

*L. argentimaculatus* GAATACGCAGGCGGCCCTTTTGCCCTATTTTTCCTAGCAGAATACGCCAACATTCTCCTC

*E. struhsakeri* GAGTATGCAGGAGGCCCCTTTGCCCTATTTTTCCTAGCAGAATACGCCAACATCTTGCTC

*B. albus* GAATATGCAGGAGGGCCTTTTGCCCTCTTTTTCTTAGCCGAATACGCCAACATCCTACTC

*C. auripes* GAATACGCAGGAGGCCCCTTCGCCCTATTTTTCCTGGCGGAATATGCCAATATTCTTCTT

*C. melampygus* GAATACGCAGGAGGCCCATTCGCACTGTTCTTCCTGGCCGAGTATGCTAACATTCTTTTA

*L. calcarifer* GAATATGCAGGAGGACCATTCGCCCTATTTTTCCTGGCAGAATATGCCAACATTCTGCTG

*S. maena* GAATATGCAGGGGGCCCCTTCGCCCTTTTTTTCCTCGCCGAGTACGCCAACATTCTCCTT

*P. auriga* GAATATGCAGGAGGCCCATTTGCCCTTTTCTTCCTAGCTGAATACGCCAACATCCTTCTT

*C. lucidus* GAATACGCAGGCGGCCCATTTGCCCTATTTTTCCTAGCAGAATACGCCAACATTCTCCTT

*S. sihama* GAATATGCAGGGGGGCCATTTGCCCTATTCTTTCTAGCAGAATATGCCAACATCCTTCTT

*C. loricula* GAATATGCAGGAGGCCCTTTCGCACTATTTTTCCTAGCCGAATACTCCAACATTCTACTC

*A. trutta* GAATACGCGGGGGGACCTTTCGCCCTATTTTTCCTGGCAGAATACGCAAACATCCTCCTA

*H. gemma* GAATATGCAGGTGGCCCCTTTGCCTTGTTCTTTTTGGCAGAATATGCCAATATCCTTCTG

*P. flavescens* GAATACGCCGGAGGACCCTTTGCGTTATTTTTCTTGGCAGAGTATGCAAACATTCTACTT

*P. macrolepida* GAGTACGCCGGAGGCCCCTTTGCTCTATTTTTCTTAGCAGAATATGCAAATATTCTGCTA

*S. canadensis* GAATACGCCGGAGGTCCCTTCGCCCTATTTTTCCTAGCAGAATATGCAAATATCTTACTT

*A. rogaa* ATAAATACACTTTCTGCTATCTTATTCCTAGGAGCCTCCCACATCCCCACTATACCTGAA

*C. argus* ATAAATACACTTTCTGCCACCCTATTCCTAGGAGCCTCCCACATTCCTGTCATGCCTGAG

*C. sonnerati* ATAAATACACTTTCCGCCACTTTATTCCTAGGAGCATCACACGTCCCCTCCATTCCCGAA

*E. fuscoguttatus* ATAAACACACTCTCCGCCACTCTTTTCCTGGGAGCTTCTCACGTCCCTATTACCCCAGAA

*E. coioides* ATAAACACCCTCTCTGCCACCCTTTTCCTGGGAGCCTCTCACATCCCCACCACCCCTGAA

*E. bruneus* ATAAATACCCTCTCTGCTACTCTTTTCCTAGGAGCCTCCCACATCCCCGCCGCCCCAGAA

*E. moara* ATAAACACCCTCTCTGCCACTCTTTTCCTAGGAGCCTCCCACATCCCCACCACCCCAGAA

*E. lanceolatus*  ATAAATACCCTCTCCGCCACTCTTTTCCTAGGAGCCTCCCACATCCCCACCATCCCAGAA

*A. leucogrammicus*  ATAAATACTCTTTCCGCCACCCTTTTCCTAGGAGCCTCCCACATCCCCACCACCCCAGAA

*C. altivelis* ATGAACACCCTCTCTGCCACCCTTTTCCTAGGAGCCTCCCACATCCCCACCGTCCCAGAA

*E. epistictus* ATGAATACCCTCTCCGCCACCCTTTTCTTAGGGGCCTCCCACATCCCTGCCGCCCCAGAA

*E. octofasciatus* ATAAATACCCTCTCTGCCACCCTCTTCCTAGGAGCCTCCCACATTCCCACCATCCCAGAA

*E. septemfasciatus* ATAAATACCCTCTCTGCCACCCTTTTCCTAGGATCCTCCCACATTCCTACCCTCCCAGAA

*T. dermopterus* ATAAATACCCTATCCGCCACCCTTTTCCTAGGGGCTTCCCACATCCCCGCCCTTCCCGAG

*E. awoara* ATAAATACCCTCTCTGCCACCCTTTTTTTAGGAGCCTCCCACATCCCCGCCACCCCTGAA

*E. akaara* ATAAATACCCTCTCCGCCACCCTTTTCTTAGGTGCCTCCCACATCCCCGCCACCCCTGAA

*E. trimaculatus* ATAAACACCCTTTCCGCCACCCTCTTCCTAGGAGCCTCCCACATCCCTACCACCCCTGAG

*E. areolatus* ATAAACACCCTGTCCGCCACCCTCTTCCTAGGAGCCTCCCATATCCCCGCCACCCCTGAA

*V. albimarginata* ATAAACACACTCTCAGCCACCCTATTCATGGGAGCCTCCCACATCCCCACCATCCCAGAA

*V. louti* ATAAACACACTCTCAGCTACCCTATTCATAGGAGCCGCCCATATCCCTACTATCCCAGAA

*P. leopardus* ATAAACACACTCTCCGCCACACTATTTTTAGGGGCATCTCACATCCCTACCCTTCCCGAA

*P. areolatus* ATAAATACACTCTCCGCCACACTATTTTTAGGAGCATCCCACATCCCAACCTTCCCCGAA

*E. radiosum* ATAAATACCCTATCCGCCACACTCTTCCTGGGGGCCTCTCACATCCCAACCCTCCCCGAA

*P. sieboldi* ATAAATACACTCTCTGCTACTCTTTTCCTTGGCGCCTCCCACGTACCGGCTTTCCCAGAG

*E. armatus* ATAAATACCCTCTCTGCTACCCTGTTTCTAGGCGCATCCCATATTCCAACAATTCCAGAA

*R. oxyrhynchus* ATAAATACCCTCTCCGCCACCCTGTTTCTAGGCGCCTCACATATTCCACTAGTCCCTGAA

*K. cinerascens* ATAAACACACTCTCCGCCACATTATTTTTAGGAGCATCCCACATCCCAACACTCCCCGAA

*T. chatareus* ATAAACACACTCTCTGCAACACTATTCCTAGGCGCATCCCACATCCCCACAATCCCTGAA

*D. berycoides* ATAAATACGCTCTCCGCCACACTATTTTTAGGAGCCTCCCACATTCCAACAATCCCGGAA

*H. typus* ATAAACACACTCTCCGCAACACTATTCCTGGGAGCTTCCCACATTCCCACAATTCCAGAA

*M. argenteus* ATAAATACACTTTCCGCCACACTCTTCCTAGGGGCTTCACATATTCCCACATTACCAGAA

*S. chuatsi* ATGAATACACTTTCCGCCACCCTTTTCTTAGGCGCAGCACACATCCCAACAATCCCAGAA

*O. fasciatus* ATAAACACACTCTCCGCAACACTATTTCTAGGAGCCTCCCACATCCCCACCTTTCCAGAA

*P. trilineatum* ATAAATACACTTTCCGCTACCCTCTTCTTAGGAGCCTCTCATATTCCCACATTTCCAGAA

*M. salmoides* ATAAATACACTTTCTGCCACACTTTTCCTAGGGGCCTCCCACATTCCCACGATTCCAGAA

*P. tile* ATAAATACACTCTCCGCCACCCTTTTCCTAGGAGCCTCCCACATCCCAACCATTCCAGAA

*L. argentimaculatus* ATAAATACACTTTCCGCCACCCTCTTCCTAGGAGCCTCACATATCCCCACCATCCCAGAA

*E. struhsakeri* ATAAACACACTCTCCGCCACTCTCTTCCTAGGAGCTTCGCACATCCCGACCATCCCAGAA

*B. albus* ATAAATACACTTTCCGCCATCCTTTTCCTGGGAGCATTTCACCTTCCAACCCTACCAACA

*C. auripes* ATAAATACACTATCTGCCACCTTATTTCTAGGAGCCTGCCACATTCCCTTATTCCCCATA

*C. melampygus* ATAAATACCCTTTCAGCCACCCTATTCTTAGGAGCCTCACACATCCCCACCCTTCCGGAA

*L. calcarifer* ATAAACACACTCTCCGCAACCCTATTCCTTGGGGCTACACACCTGCCCACCATCCCCGAA

*S. maena* ATAAATACCCTTTCTGCCACGCTATTCCTCGGGGCTTCCCATGTTCCGACCCTTCCAGAA

*P. auriga* ATAAATACACTTTCTGCCACACTATTCCTGGGAGCCTTCCATATACCAACATTCCCCGAA

*C. lucidus* ATAAATACACTCTCGGCCACTTTATTCTTGGGTGCCTCCCACATTCCCACCATCCCTGAA

*S. sihama* ATGAATACACTTTCCGCCACCCTTTTCCTAGGCGCCTCTCACATTCCACTGATTCCTGAA

*C. loricula* ATAAATACCCTTTCCGCCGTCCTCTTCCTAGGAGCCACCTACCTGCCCCTCACCCCCATA

*A. trutta* ATAAACACTCTTTCCGCCACATTATTCCTAGGTGCATCCCATATCCCAACGATCCCCGAG

*H. gemma* ATAAATACCCTTTCTGCCATCTTATTTTTAGGAGCAACCCACGCCCCCACAATTCCAGAA

*P. flavescens* ATAAACACACTTTCTGCGACCCTGTTCTTAGGGGCTTCTCACATTCCTTCTCTTCCTGAA

*P. macrolepida* ATAAACACCCTTTCCGCCACGCTCTTTTTAGGGGCCTCTCACATCCCGACCTTGCCCGAA

*S. canadensis* ATGAATACACTCTCCGCAACTTTATTCCTAGGAGCCTCCCACATTCCCTCCATCCCTGAG

*A. rogaa* CTAACAGCTGCCAATCTAATAACAAAAGCAGTCCTCCTATCCGTCCTATTCCTATGAGTT

*C. argus* TTTACAGCTGCTAACCTAATAACAAAAGCAGCCCTCCTATCTATTCTGTTCTTGTGAGTC

*C. sonnerati* TTTACGGCCATAAACCTGATAACAAAAGCAGCCCTTCTTTCTGTACTCTTCCTATGAGTC

*E. fuscoguttatus* CTAACAACCGTAAATTTAATAACAAAAGCAGCACTCCTTTCCCTACTTTTCCTCTGAGTC

*E. coioides* ATAACAGCCATAAATTTAATAACAAAAGCAGCTCTCCTCTCCCTGCTCTTCCTCTGAGTT

*E. bruneus* CTAACAGCTGTCAATTTAATAACAAAAGCAGCACTCCTTTCTCTACTCTTCCTATGAGTT

*E. moara* CTAACAGCTGTTAATTTAATAACAAAAGCAGCACTCCTTTCCCTACTCTTCCTATGAGTT

*E. lanceolatus*  ATAACAACTATAAATCTAATAACAAAAGCAGCACTACTCTCCCTACTTTTCCTCTGAGTC

*A. leucogrammicus*  CTAACAGCCATTAACTTAATAACAAAGGCAGCACTCCTCTCCCTCCTCTTCCTTTGGGTT

*C. altivelis* CTAACGGCCATAAACCTAATGACAAAAGCAGCACTCCTCTCACTACTTTTCCTCTGAGTT

*E. epistictus* CTAACAGCCATAAACCTAATAACAAAAGCAGCACTCCTCTCCCTACTATTCCTTTGAGTC

*E. octofasciatus* CTAATAACCCTAAATCTAATGACAAAAGCAGCCCTCCTTTCTCTACTTTTTCTGTGGGTT

*E. septemfasciatus* CTAACAGCCCTAAATCTAATGACAAAAGCAGCCCTCCTTTCTCTACTTTTTCTATGAGTT

*T. dermopterus* CTCACAGCTATAAACATAATAACAAAAGCAGCCCTCCTCTCTCTACTCTTCCTGTGAGTC

*E. awoara* CTAACAGCCATAAACCTAATAACAAAAGCAGCACTCCTTTCGCTACTTTTCCTCTGAGTC

*E. akaara* CTAACAGCCGTAAATCTAATAACAAAAGCAGCACTCCTCTCCTTACTCTTTCTCTGAGTC

*E. trimaculatus* CTAACAGCCATAAACCTAATAACAAAAGCAGCACTCCTCTCTTTACTCTTCCTGTGGGTA

*E. areolatus* CTGACAGCCATAAATTTAATAACTAAAGCAGCCCTCCTCTCCTTATTATTCCTCTGAGTC

*V. albimarginata* CTTACAGCCATAAACCTCATAACTAAAGCAGCACTCCTCTCAGTCCTTTTCTTATGGGTC

*V. louti* CTCACAGCTATGAACCTTATAACTAAAGCAGCGCTCCTCTCAGTCCTCTTCTTATGAGTC

*P. leopardus* CTAACTGCCGCCAATCTAATAACTAAAGCAGCACTTCTATCAGTTTTATTCCTATGAGTT

*P. areolatus* CTTACCGCCGCCAACTTAATAACAAAAGCAGCGCTTCTGTCAATCCTTTTCCTGTGAGTC

*E. radiosum* CTTACTGCTATTAACTTGATGACTAAGGCAGCCCTCCTATCGGTGGTCTTTCTCTGGGTG

*P. sieboldi* CTCACTGCGGTAAACCTCATGACGAAGGCGGCCCTTCTCTCCGTGCTCTTCCTTTGGGTG

*E. armatus* CTCACCGCGGTTAATTTAATGACCAAAGCGGCCTTACTTTCAATTGTTTTCCTATGAGTT

*R. oxyrhynchus* TTCACCGCTATTAACCTCATGACCAAAGCCGCCCTCCTTTCAGTTCTCTTCCTCTGAGTT

*K. cinerascens* CTCACTGCTATGAACCTCATAACCAAGGCAGCACTCCTCTCAATCGTCTTCCTCTGAGTT

*T. chatareus* CTCACTGCCATAAACCTCATAACTAAAGCAGCATTCCTGTCCATCCTCTTCCTCTGAGTT

*D. berycoides* CTCACCACGCTCAACCTAATAACCAAAGCAGCACTTCTTTCAATAGTCTTCCTCTGAGTT

*H. typus* CTCACGGCAATCAACCTAATAACTAAAGCAGCACTTCTTTCAATTGTGTTCCTATGAGTT

*M. argenteus* TTAACTGCAATCAACCTAATGACCAAGGCAGCCCTACTATCGATTGTATTCCTTTGGGTT

*S. chuatsi* CTCACTGCAGTCAACCTAATAACTAAAGCCGCCCTCCTTTCAATCGTATTCCTTTGAGTT

*O. fasciatus* CTTACCGCTATAAACCTTATAATCAAAGCAGCCCTCCTCTCAGTTGTCTTCCTATGAGTT

*P. trilineatum* CTTACCGCAATAAATTTAATGACTAAAGCAGCCCTCCTTTCAGTCGTATTCCTGTGAGTT

*M. salmoides* CTTACTACCATTAACCTAATAACCAAAGCTGCCCTCCTCTCAGTCATCTTCCTATGAGTT

*P. tile* CTCACTGCAATAAACCTAATGACCAAAGCAGCCTTCCTCTCTATTGTTTTCCTATGAGTA

*L. argentimaculatus* CTTACCGCAATCAACCTCATAACTAAAGCAGCTTTCCTCTCAATTGTCTTCCTATGAGTT

*E. struhsakeri* CTAACCGCAGTTAACCTAATAACTAAAGCAGCCCTACTTTCAATTGTGTTTCTTTGAGTC

*B. albus* CTAACTGCAATCAACCTAATAACTAAAGCAGCCCTACTATCCGTAGTTTTCTTATGGGTC

*C. auripes* CTTACCACGATTAATATAATGACAAAAGCTGCCCTCCTCTCAATTCTCTTCTTATGAGTC

*C. melampygus* CTTACCGCCATTAACCTCATAACTAAAGCAGCCCTCCTTTCTATCGTCTTCCTCTGAGTC

*L. calcarifer* CTCACCACCATCAACCTGATAACAAAAACAGCACTACTATCCGTACTATTCTTATGAGTC

*S. maena* CTTACCGCAATAAACCTCATGACTAAGGCTGCCCTCTTGTCAGTAATATTCTTATGAGTC

*P. auriga* CTGACCGCAATAAATTTAATGACTAAAGCAGCACTACTTTCAATTGTTTTCCTATGGGTT

*C. lucidus* TTAACCGCCATAAACATTATAACTAAGGCAGCCTTCCTCTCCGTCCTATTCCTATGAGTC

*S. sihama* CTCACCGCCGTTAATCTCATGACAAAAGCTGCCTTTCTCTCAGTACTCTTCGTTTGAACC

*C. loricula* CTTACAACAAGCAGCCTTATAGGCAAAGCAACCCTCCTCGCGGTCGCCTTCTTATGAGTA

*A. trutta* CTTACTGCCATGAATCTAATATTTAAAGCATCCCTTCTTTCCATAGTCTTCCTCTGAGTG

*H. gemma* CTTACTGCCATCAACCTTATGACTAAAACAGCTTTGCTTTCTATTGTATTCCTCTGAGTA

*P. flavescens* CTTACCGCTGTGAATCTAATGACTAAAGCAGCCCTCCTCTCAGTTGTATTTCTCTGGGTT

*P. macrolepida* CTCACTGCTCTTAATTTAATAACTAAAGCCGCCCTCCTTTCTGTTGTCTTCCTTTGAGTC

*S. canadensis* CTTACTGCTATTAACCTTATAACTAAAGCAGCTCTTCTTTCCATCGTGTTTCTCTGGGTT

*A. rogaa* CGAGCCTCCTATCCACGATTCCGCTACGACCAGCTAATACACCTGATCTGAAAAAATTTC

*C. argus* CGAGCCTCCTACCCACGATTTCGCTACGACCAACTAATACATCTAATCTGAAAAAACTTC

*C. sonnerati* CGAGCCTCCTACCCACGATTCCGTTATGACCAACTAATGCACCTAATCTGAAAAAATTTC

*E. fuscoguttatus* CGAGCCTCCTACCCTCGATTCCGATACGACCAATTAATACACTTAATTTGAAAAAACTTT

*E. coioides* CGAGCCTCCTACCCTCGATTCCGATACGACCAGTTAATACATTTAATCTGAAAAAACTTT

*E. bruneus* CGAGCCTCCTACCCCCGGTTCCGATACGACCAACTAATGCATTTAATCTGAAAAAACTTC

*E. moara* CGAGCCTCCTACCCCCGCTTCCGATACGACCAATTAATGCATTTAATCTGAAAAAACTTT

*E. lanceolatus*  CGAGCCTCCTACCCCCGTTTTCGATATGACCAATTAATACATTTGATCTGAAAAAACTTT

*A. leucogrammicus*  CGAGCCTCTTACCCCCGATTCCGTTATGACCAGTTAATGCATTTAATCTGAAAAAACTTC

*C. altivelis* CGAGCCTCCTACCCTCGATTCCGATACGACCAGCTAATACATTTAATCTGAAAAAACTTT

*E. epistictus* CGAGCCTCCTACCCCCGATTCCGATACGACCAGCTAATACACCTAATCTGAAAAAACTTT

*E. octofasciatus* CGAGCCTCCTACCCACGATTCCGATATGACCAACTAATGCACTTAATCTGAAAAAATTTC

*E. septemfasciatus* CGAGCCTCCTACCCACGATTTCGATATGACCAACTAATGCACTTAATCTGAAAAAACTTC

*T. dermopterus* CGAGCCTCGTACCCCCGGTTCCGATATGATCAACTAATGCACCTGATCTGAAAAAATTTT

*E. awoara* CGAGCCTCTTACCCTCGATTCCGATACGACCAACTAATGCATTTAATCTGAAAAAATTTT

*E. akaara* CGAGCCTCCTACCCTCGATTCCGGTACGACCAACTAATGCATTTAATCTGAAAAAACTTC

*E. trimaculatus* CGAGCCTCTTACCCCCGTTTCCGGTATGATCAACTAATACACCTAATCTGAAAAAATTTT

*E. areolatus* CGAGCCTCCTACCCCCGATTCCGATATGACCAGCTAATGCACCTAATCTGAAAAAACTTT

*V. albimarginata* CGAGCCTCTTACCCACGATTTCGTTACGACCAATTAATGCATCTAATCTGAAAAAACTTT

*V. louti* CGAGCCTCTTACCCACGATTTCGTTACGACCAATTAATGCATCTTATCTGAAAAAACTTT

*P. leopardus* CGGGCCTCATACCCACGATTTCGTTACGACCAGCTAATGCACTTAATTTGAAAAAATTTC

*P. areolatus* CGAGCCTCATACCCCCGATTTCGTTACGATCAACTCATGCACCTGATCTGAAAAAACTTC

*E. radiosum* CGGGCCTCTTACCCTCGGTTTCGGTACGACCAACTAATGCACCTCATCTGGAAGAATTTT

*P. sieboldi* CGGGCATCTTACCCGCGGTTCCGGTACGACCAGCTCATGCACCTGATTTGGAAAAATTTT

*E. armatus* CGAGCCTCCTACCCTCGCTTCCGTTATGACCAACTAATGCATCTTATTTGAAAAAACTTT

*R. oxyrhynchus* CGAGCCTCTTACCCACGATTCCGATATGACCAACTTATGCACCTCATTTGAAAAAACTTC

*K. cinerascens* CGAGCCTCATACCCTCGATTCCGATACGATCAACTCATGCACTTGATCTGAAAAAACTTC

*T. chatareus* CGAGCCTCCTACCCCCGATTCCGCTACGATCAACTAATACACCTAGTCTGAAAAAACTTC

*D. berycoides* CGAGCCTCATACCCGCGATTTCGCTACGACCAACTTATACACCTTATCTGAAAAAACTTC

*H. typus* CGAGCCTCATATCCCCGATTCCGATATGATCAACTCATGCACCTCATTTGAAAAAACTTC

*M. argenteus* CGAGCCTCCTACCCCCGATTTCGGTACGATCAACTCATGCACCTCATCTGAAAAAACTTC

*S. chuatsi* CGGGCCTCATACCCCCGATTCCGGTACGACCAGCTTATGCACCTGATCTGAAAAAACTTC

*O. fasciatus* CGAGCCTCCTACCCTCGATTCCGATATGACCAACTCATACACCTTATTTGAAAAAACTTT

*P. trilineatum* CGAGCCTCCTACCCCCGATTCCGATATGACCAACTTATGCACCTCATTTGAAAAAACTTC

*M. salmoides* CGAGCATCCTACCCCCGATTTCGCTATGACCAGCTCATGCACTTAATTTGAAAAAACTTC

*P. tile* CGAGCCTCGTACCCCCGATTTCGGTACGACCAACTCATGCACCTAATCTGAAAAAACTTC

*L. argentimaculatus* CGAGCCTCCTACCCCCGATTCCGATACGACCAGCTCATGCACTTAATTTGAAAAAACTTC

*E. struhsakeri* CGAGCCTCATATCCCCGATTCCGGTATGACCAACTCATGCACCTCATCTGAAAAAACTTT

*B. albus* CGAGCCTCATACCCCCGATTCCGATATGACCAACTCATGCACCTCATCTGAAAAAACTTC

*C. auripes* CGAGCCTCATACCCTCGTTTCCGATACGACCAGCTCATACACCTGATCTGAAAGAACTTC

*C. melampygus* CGAGCATCCTACCCCCGATTCCGATACGACCAGCTTATGCACCTTATCTGAAAAAACTTC

*L. calcarifer* CGAGCCTCTTACCCTCGCTTCCGATATGATCAGCTTATGCACCTAATCTGAAAAAATTTT

*S. maena* CGGGCTTCCTACCCCCGATTCCGCTACGACCAGCTTATACACCTAATTTGAAAAAACTTC

*P. auriga* CGAGCTTCCTACCCCCGATTTCGATATGACCAGTTAATACACCTAATCTGAAAAAACTTT

*C. lucidus* CGAGCCTCCTACCCCCGATTCCGATATGATCAACTCATGCACCTAATCTGAAAAAACTTC

*S. sihama* CGAGCTTCCTACCCCCGATTCCGATATAATCAACTAATGCATCTCATCTGAAAGAGTTTC

*C. loricula* CGAGCCTCTTACCCCCGATTTCGGTATGACCAACTTATACACCTCATTTGAAAAAGTTTC

*A. trutta* CGAGCCTCCTACCCACGTTTCCGCTACGACCAACTTATACACCTAATTTGAAAAAATTTC

*H. gemma* CGTGCCTCTTACCCCCGTTTCCGCTACGACCAGCTGATACACCTAATCTGAAAAAATTTC

*P. flavescens* CGAGCCTCCTACCCTCGATTTCGGTATGATCAACTAATACACCTTATTTGAAAAAATTTC

*P. macrolepida* CGGGCCTCCTACCCCCGATTCCGGTATGACCAACTCATGCACCTAATCTGAAAGAATTTC

*S. canadensis* CGAGCCTCCTACCCTCGCTTCCGTTATGACCAGTTAATGCACCTAATTTGGAAAAATTTC

*A. rogaa* CTCCCACTTACACTAGCCTTAGTAATCTGACATCTTGCACTTCCCATTGCATTCGCTGGG

*C. argus* CTTCCACTTACACTAGCCCTGGTTATCTGACATTTGGCGCTGCCCATTGCATTCGCTGGA

*C. sonnerati* CTTCCACTTACACTAGCCCTGGTCATCTGACACCTTGCACTTCCTATTGCATTCGCTGGA

*E. fuscoguttatus* CTTCCCCTTACACTGGCTTTAGTCATCTGACATCTAGCGCTCCCTATTGCACTAGCCGGC

*E. coioides* CTTCCCCTCACACTAGCTTTGGTTATCTGACACCTCGCGCTCCCCATTGCATTAGCTGGA

*E. bruneus* CTTCCCCTTACACTAGCTTTAGTCATTTGACACCTTGCGCTCCCTATCGCATTTGCCGGA

*E. moara* CTTCCCCTTACACTAGCTCTAGTCATTTGACACCTTGCGCTCCCTATCGCATTTGCTGGA

*E. lanceolatus*  CTCCCCCTCACACTAGCTCTGGTCATTTGACATCTTGCACTCCCCATTGCACTGGCAGGC

*A. leucogrammicus*  CTCCCTCTCACACTAGCCTTAGTTATTTGACACCTCGCACTTCCCATCGCACTAGCTGGT

*C. altivelis* CTTCCTCTTACACTAGCCTTGGTTATTTGACACCTCGCACTTCCTATTGCACTAGCTGGA

*E. epistictus* CTCCCACTCACATTAGCCTTAGTTATTTGACACCTCGCACTCCCCATTGCATTCGCTGGA

*E. octofasciatus* CTTCCACTTACACTAGCTTTAGTTATCTGACACCTTGCGCTCCCTATTGCATTCGCCGGA

*E. septemfasciatus* CTTCCACTTACACTAGCCTTAGTTATCTGACACCTTGCGCTCCCTATTGCATTCGCCGGA

*T. dermopterus* CTCCCTCTCACACTAGCCTTAGTTATCTGACACCTCGCGCTCCCCATTGCATTCGCCGGA

*E. awoara* CCTCCACTCACACTAGCCCTAGTTATCTGACACCTTGCACTCCCTATCGCATTCGCCGGA

*E. akaara* CTTCCACTCACACTAGCCTTAGTTATCTGACACCTCGCACTCCCCATCGCGTTCGCCGGC

*E. trimaculatus* CTCCCACTTACGCTAGCCCTAGTAATTTGACACCTCGCACTCCCAATTGCATTCGCTGGT

*E. areolatus* CTTCCACTCACACTGGCTTTAGTTATTTGACACCTTGCACTCCCCATTGCATTCGCCGGC

*V. albimarginata* CTACCACTTACACTAGCACTAGTTATTTTACACTTAGCGCTTCCCATTGCATTTGTTGGT

*V. louti* CTACCACTAACATTGGCACTAGTTATTTTACACTTAGCACTTCCCATCGCATTTGTTGGT

*P. leopardus* CTCCCTCTTACACTAGCTCTGGTCATCTGACACCTAGCCCTCCCCATCGCATTTGCTGGT

*P. areolatus* CTCCCTCTCACTCTAGCCCTAATCATCTGACACTTAGCCCTCCCCATTGCATTCGCTGGC

*E. radiosum* TTACCCCTAACCTTATCCCTGGTCATTTGACACCTTGCGCTTCCCATTGCATTTGCTGGC

*P. sieboldi* CTTCCCCTCACCCTGGCCCTTGTTATCTGACACCTTGCACTGCCAACTGCCTTCGCGGGG

*E. armatus* TTACCTCTTACATTAGCCCTGGTTATCTGACACCTTGCCCTTCCCATTGCATTTGCTGGA

*R. oxyrhynchus* CTTCCCCTTACACTAGCCCTGATTATCTGACACTTAGCCCTCCCCATTGCATTTGCAGGC

*K. cinerascens* CTCCCCCTCACACTAGCCTTAGTCATCTGACATCTAGCCCTTCCCATCGCATTCGCGGGA

*T. chatareus* CTCCCAATTACACTAGCCCTGGTAATCTGACACCTTGCACTTCCAATTGCTTTCGCCGGA

*D. berycoides* CTGCCTCTCACACTAGCACTGGTTGTATGACACCTCGCCCTTCCCATTGCATTAGCAGGT

*H. typus* TTACCCCTCACCCTAGCCCTGGTCATCTGACACCTCGCCCTTCCCATTGCATTTGCAGGC

*M. argenteus* CTACCCCTTACACTAGCCCTGGTTATCTGACACTTAGCCCTTCCCATTGCATTTGCAGGA

*S. chuatsi* CTACCTCTCACACTATCCCTAGTTATTTGACACCTTTCACTTCCCATTGCATTCGCAGGA

*O. fasciatus* CTTCCACTGACACTGGCCCTAGTCATCTGACACCTGGCCCTCCCCATTGCATTCGCAGGG

*P. trilineatum* CTTCCTCTGACATTAGCATTAGTAATCTGACACCTTGCCCTTCCTATTGCCTTCGCAGGA

*M. salmoides* CTACCCCTTACACTAGCCTTAGTCATTTGACACTTGGCCCTTCCTATTGCATTTGCAGGT

*P. tile* CTTCCCCTAACCCTCGCCCTGGTGATTTGACACCTCGCGCTTCCAATCGCATTCGCAGGC

*L. argentimaculatus* CTCCCGCTAACCCTCGCCCTAGTAATCTGACACCTTGCACTCCCCATCGCATTCGCAGGC

*E. struhsakeri* CTTCCCCTAACGCTGGCCTTAGTTATTTGACACCTAGCACTCCCCATCGCATTTGCAGGG

*B. albus* CTCCCCTTAACACTGGCCCTCGTTATCTGACATCTCGCCCTTCCGATTGCCCTTGCAGGC

*C. auripes* CTGCCCCTCACACTAGCCCTACTTATCTGACACCTCGCACTTCCCATCGCATTTGCAGGC

*C. melampygus* CTCCCTCTCACACTAGCACTTGTCATCTGACATCTAGCACTACCCATCGCATTCGCAGGC

*L. calcarifer* CTTCCCCTCACACTAGCCCTGGTAATTTGACACCTGGCACTCCCTATCGCATTCACTGGC

*S. maena* CTTCCTTTGACTCTTGCCCTAGTTATCTGACACCTTTCCCTCCCTGTCGCATTCACAGGG

*P. auriga* CTCCCTCTAACACTAGCCCTGGTCATTTGACACCTTGCCATTCCGATTGCATTTGCAGGC

*C. lucidus* CTCCCATTAACACTTGCCCTCGTCATCTGACACCTGGCACTTCCCATTGCACTTGCAGGC

*S. sihama* CTCCCTTTGACACTCGCCTTGGTCATCTGACACTTAGCCCTTCCAATTGCGTTCGCTGGC

*C. loricula* CTCCCTATAACATTGGCCCTAGTTATTTGACACTTATCAGTCACCATCGGATTTGCGGGA

*A. trutta* CTTCCCTTAACACTAGCCCTTGTTATCTGGCACCTCGCACTACCCATTGCATTCGCAGGC

*H. gemma* CTCCCCCTTACTTTAGCCCTTGTACTTTGACAACTAGCTATTCCAACTGCATTTGGAGGA

*P. flavescens* CTTCCCTTAACACTGGCCCTGGTTATCTGACACCTAGCACTTCCCATTGCATTTGCTGGC

*P. macrolepida* CTCCCACTAACTTTGGCCTTAGTTATTTGACATCTCGCCCTTCCCATTGCATTTGCTGGT

*S. canadensis* CTCCCACTAACATTAGCATTAGTCATTTGGCACTTAGCGCTCCCCATTGCATTTGCTGGC

*A. rogaa* TTACCACCTCAAATATAAATGAACCCATTCATCCTAGCCACTCTGCTGTTTGGTCTTGGC

*C. argus* CTACCGCCCCAAATGTAAATGAACCCATACATCTTAGCCACCCTGCTATTTAGTCTTGGC

*C. sonnerati* CTACCACCCCAAGTGTAAATGAACCCATACATTATAGCCACACTGCTATTTGGCCTTGGC

*E. fuscoguttatus* CTTCCCCCTCAAATCTAAATGAATCCATATATTTTAGCTATTTTACTATTCAGTCTCGGA

*E. coioides* CTTCCCCCTCAAATTTAAATGAATCCATATATTTGAGCTATCTTACTATTTAGCCTTGGA

*E. bruneus* CTCCCTCCTCAAATCTAAATGAATCCATATATCTTAGCTACTTTACTATTTAGTCTTGGA

*E. moara* CTCCCCCCTCAAATCTAAATGAATCCATATATCTTAGCTACTTTACTATTTAGCCTTGGA

*E. lanceolatus*  CTCCCCCCTCAAATTTAAATGAATCCATACATCTTAGCTATCCTACTACTCAGCCTTGGA

*A. leucogrammicus*  CTCCCCCCCCAAACCTAAATGAATCCATATATTTTAGCTACTTTACTATTCAGTCTTGGA

*C. altivelis* CTCCCCCCTCAATTTTAAATGAATCCATATATTTTAGCTACCTTTCTACTTAGTCTTGGA

*E. epistictus* CTTCCCCCTCAAATCTAAATGAACCCATATATTTTAGCCACCCTACTATTTAGTCTCGGA

*E. octofasciatus* CTTCCCCCTCAACTCTAAATGAATCCATATATTTTAGCCGCCCTATTATTTAGCCTCGGG

*E. septemfasciatus* CTTCCCCCTCAACTCTAAATGAATCCATACATCTTAACCGCCCTATTATTTAGCCTCGGG

*T. dermopterus* CTTCCCCCTCAAACCTAGATGAACCCATATATTTTAGCTGCCCTATTATTTGGCCTTGGA

*E. awoara* CTTCCCCCTCAAATTTAAATGAACCCATACATCTTAGCCACCCTATTACTTAGCCTTGGA

*E. akaara* CTCCCCCCTCAAATTTAAATGAACCCATATATCTTAGCCACTCTACTACTTAGCCTTGGA

*E. trimaculatus* CTTCCCCCTCAAATTTAAATGAATCCATACATTTCAGCTACCCTACTACTTAGCCTCGGA

*E. areolatus* CTCCCCCCTCAACTTTAAATGAACCCATACATCTTAGCCACCCTATTATTTAGCCTCGGA

*V. albimarginata* CTCCCTCCTCAAATCTAGATGAACCCATACATCTCAGCCACTCTCCTCTTCGGCCTAGGC

*V. louti* CTTCCTCCCCAAGTTTAAATGAACCCATACATCTCAGCCACACTCCTCTTCGGCCTAGGC

*P. leopardus* CTTCCCCCTCAACTATAAATGAATCCATACATTTTAGCCATCCTAGTACTTAGCTTGGGT

*P. areolatus* CTCCCCCCTCAGCTATAAATGAATCCATACATCTTAGCCATCCTAGTACTTAGCCTGGGT

*E. radiosum* TTACCCCCTCAACTATAGATGAACCCGTACATCTTAGCCACCCTCCTCTTTGGTTTAGGC

*P. sieboldi* CTACCGCCCCAACTATAAATGAATCCATACATTTTAGCCACCCTACTCATAGGACTTGGC

*E. armatus* CTACCCCCACAGCTATAAATGAACCCGTACATCTTAGCTACTCTGCTATTTGGACTAGGC

*R. oxyrhynchus* CTACCCCCGCAACTGTAGATGAGTCCATACATTCTAACCGTCCTACTATTTGGCCTTGGC

*K. cinerascens* CTTCCCCCACATTTATAGATGAACCCGTACATCTTAGCCACCTTGCTGTTTGGACTAGGC

*T. chatareus* TTACCCCCACAACTCTAAATGAACCCTACCATCCTGCCCATCTTACTACTTGGTCTAGTA

*D. berycoides* CTTCCCCCTCAGCTGTAAATGAACCCGTACATCTTAGCCACCCTGCTATTTGGACTAGGC

*H. typus* CTGCCCCCTCAACTCTAAATGAACCCGTACATCTTAGCCACCCTGCTATTTGGACTAGGC

*M. argenteus* CTACCCCCTCAGCTATAAATGAACCCGTACATCTTAGCCACCCTGCTATTTGGACTCGGC

*S. chuatsi* CTACCCCCTCAACTATAAATGAACCCGTACATCTTAGCCACCCTGCTATTTGGACTAGGC

*O. fasciatus* CTACCCCCACACCTATAAATGAACCCCTACCTCTTAGCCACTTTGCTATTTGGACTCGGC

*P. trilineatum* CTACCCCCACAACTATAAATGAGCCCGTACATCTTAGCCACCCTGCTATTTGGACTAGGC

*M. salmoides* CTACCGCCTATACTCTAAATGAACCCGTACATCTTAGCCACCCTACTATTTGGTTTAGGC

*P. tile* CTTCCCCCACAACTATAGATGAACCCGTACATCTTAGCCACCCTTTTATTCGGACTAGGC

*L. argentimaculatus* CTACCCCCCCAACTATAAATGAACCCGTACATCTTAGCCACCCTCTTATTCGGTCTAGGC

*E. struhsakeri* TTACCCCCTCAACTATAGATGAACCCGTACATCTTAGCCACCCTGCTATTCGGACTAGGC

*B. albus* CTCCCCCCCGTACTATAAATGAACCCATATGTCTTTGCCATCCTCCTCTCTGGACTTGGC

*C. auripes* CTCCCACCACAA---TAAATGAACCCCTACATCTTAACTGCCCTCCTATTTGGCCTTGGC

*C. melampygus* CTACCCCCTCAACTATAAATGAACCCTTACATTTTAGCCATCCTATTATTTGGTTTAGGT

*L. calcarifer* ATCCCACCCCAACTATAAATGAACCCATATGTCCTAATTACCCTCTTATTTGGACTAGGC

*S. maena* CTCCCCCCCCATCTCTAGATGATACCCCTTATCTTAGCCATTTTTCTCTTTACACTCGGA

*P. auriga* CTACCCCCTCACCTCTAAATGAATCCCTTCATCTTAGTTACCCTGCTCTTTGCACTAGGC

*C. lucidus* CTCCCCCCTCAGCTATAGATGAATCCCATCGTTTCATGCACCTTATTAATTACTCTCGGA

*S. sihama* CTCCCCCCACAGATTTAGATGAATCCTTACATTCTAGCCACACTGATTTTCGGACTAGGG

*C. loricula* GTCCCCCCTCAGACGTAGATGACGGTTACTACGCTCACTATCCTACTTCTCGCACTCGGT

*A. trutta* CTCCCCCCTCACCTCTAAATGAACCCGTATATTCTAGCTGTCCTCCTATCCGGGTTAGGC

*H. gemma* CTTCCTCCTCAGTCCTAAATGAATCCATACGTTTTATTTTTCCTCTTATTTGGCCTTGGC

*P. flavescens* CTCCCCCCTCAGCTATAAATGAACCCGTACATCTTAGCCACCCTTCTTTTTGGTTTAGGC

*P. macrolepida* CTGCCACCACAGCTATAGATGAACCCGTACATCTTAGCCGCCCTACTTTTTGGTTTAGGC

*S. canadensis* CTCCCCCCTCAGCTATAAATGAACCCGTACATCTTAGCCACCCTACTTTTTGGTTTAGGC

*A. rogaa* CTAGGGACCACAATTACATTTGCAAGCTCTCACTGATTATTAGCTTGAATGGGGTTAGAA

*C. argus* CTAGGGACCACAATCACATTTGCAAGCTCTCACTGATTATTAGCTTGAATAGGGCTAGAG

*C. sonnerati* TTAGGGACCACAATTACATTCGCAAGCTCCCACTGACTCCTAGCCTGAATAGGACTAGAA

*E. fuscoguttatus* CTAGGGACCACAATCACACTCACAAGCTCCCACTGGCTTTTTGCCTGAATAGGCTTAGAA

*E. coioides* CTGGGAACCACCATCACACTCACAAGCTCCCATTGACTCTTTGCCTGAATAGGCTTAGAA

*E. bruneus* CTAGGAACCACCATTACACTCACAAGCTCTCACTGACTATTTGCCTGAATAGGCTTAGAA

*E. moara* CTAGGAACCACCATTACACTCACAAGCTCTCACTGACTATTTGCCTGAATAGGCTTAGAA

*E. lanceolatus*  CTAGGAATCACCATCACACTCACAAGCTCCCACTGACTATTCGCCTGAATAGGCTTAGAA

*A. leucogrammicus*  CTAGGAACTACTATCACACTTACAAGCTCTCACTGACTCTTTGCCTGAATGGGCTTAGAA

*C. altivelis* CTAGGAACCACCATTACACTTACAAGCTCCCATTGACTCTTTGCCTGAATAGGCCTAGAA

*E. epistictus* TTAGGAACCACCATTACATTTGCAAGCTCTCACTGACTACTCGCCTGAATAGGCCTAGAA

*E. octofasciatus* CTAGGAACCACAATTACATTCATAAGCTCCCACTGATTACTCGCCTGAATAGGTTTAGAA

*E. septemfasciatus* CTAGGAACCACAATTACATTCATAAGCTCCCACTGATTACTCGCCTGAATAGGCCTAGAA

*T. dermopterus* CTGGGGACCACAGTTACATTCGCTAGCTCCCACTGACTACTCGCCTGAATAGGTCTAGAA

*E. awoara* CTAGGAACCACTATTACATTTGCAAGTTCACACTGATTACTCGCCTGAATAGGCCTAGAA

*E. akaara* CTAGGAACCACTATTACATTTGCAAGCTCCCACTGATTACTCGCCTGAATAGGCCTAGAA

*E. trimaculatus* TTAGGAACCACTATTACATTTGCAAGCTCCCACTGATTACTTGCCTGAATAGGCCTAGAA

*E. areolatus* CTAGGAACCACCATTACATTTGCAAGCTCCCACTGACTGCTCGCCTGAATAGGACTAGAA

*V. albimarginata* CTAGGAACTACTATTACATTTGCAAGCTCCCATTGACTTTTAGCCTGAATAGGTCTTGAA

*V. louti* CTAGGAACCACTATTACATTTGCAAGCTCCCATTGACTTTTAGCCTGAATAGGTCTTGAG

*P. leopardus* TTAGGAACTGCTCTGACACTGACAAGTTCACATTGACTGCTTGCCTGAATAGGACTTGAA

*P. areolatus* TTAGGAACTTCTCTAGCACTGACAAGTTCACATTGACTGCTTGCCTGAATAGGGCTTGAA

*E. radiosum* CTAGGCACTACAGTCACATTCGCGAGCTCACATTGACTTCTGGCCTGAATGGGACTTGAA

*P. sieboldi* CTAGGGACCACAATTACATTTGCAAGCTCTCACTGGCTTCTCGCATGAATGGGGCTTGAA

*E. armatus* ATGGGAACTACAATTACGTTTGCAAGCTCACATTGATTACTTGCCTGAATAGGACTTGAA

*R. oxyrhynchus* CTCGGGACCACAATTACATTCGCAAGCTCACACTGACTTCTTGCCTGAATAGGCCTTGAA

*K. cinerascens* CTAGGAACCACAATCACATTTGCAAGCTCCCACTGACTCCTTGCCTGAATAGGACTTGAA

*T. chatareus* TTAGGCACCACAATCACATTTATAAGCTCACACTGACTAGTAGCATGAATAGGCCTAGAA

*D. berycoides* CTAGGAACCACAATCACATTCGCAAGCTCACATTGATTGCTCGCCTGAATAGGACTTGAA

*H. typus* CTAGGAACCACAATCACATTCGCAAGCTCACACTGACTCCTCGCCTGAATAGGACTTGAA

*M. argenteus* CTAGGAACCTCAATCACCTTCGCAAGCTCACACTGACTACTTGCCTGAATAGGCCTTGAA

*S. chuatsi* CTAGGAACCACAATCACCTTTGCCAGCTCTCACTGACTCCTGGCCTGAATAGGACTTGAA

*O. fasciatus* CTAGGAACCACAATTACATTCACAAGCTCCCACTGACTCCTTGCTTGAATAGGACTTGAG

*P. trilineatum* CTAGGGACTACCATTACATTCGCAAGCTCCCACTGGCTCCTTGCCTGAATAGGCCTTGAA

*M. salmoides* CTGGGGACCACAATTACATTTGCAAGCTCCCACTGACTCCTTGCCTGAATAGGACTTGAA

*P. tile* CTAGGAACTACAATCACATTCGCAAGCTCACATTGACTCCTTGCCTGAATAGGACTTGAA

*L. argentimaculatus* CTAGGAACTACCATTACATTCGCGAGCTCACACTGACTCCTCGCTTGAATAGGACTTGAA

*E. struhsakeri* CTAGGAACCACAATTACATTTGCAAGCTCCCACTGACTGCTAGCATGAATAGGACTTGAA

*B. albus* CTAGGTACCACCCTCACCTTCGCAAGCTCCCACTGGCTCCTCGCTTGAATAGGCCTGGAA

*C. auripes* CTAGGCACTACAATTACACTAGCAAGCTCACACTGACTGCTAGCCTGAATAGGCCTAGAA

*C. melampygus* TTAGGAACAACAATCACATTCGCGAGCTCACACTGACTGCTTGCCTGAATGGGCCTAGAA

*L. calcarifer* CTAGGAACCACCATTACATTTACAAGTTCACACTGACTACTCGCCTGAATAGGACTAGAA

*S. maena* CTAGGAACCACAATCACTTTTATAAGCTGCCATTGAGTACTCGCCTGAATTGGCCTTGAA

*P. auriga* CTAGGAACTACAATCGCATTTGCAAGCTCCCACTGATTGCTTGCCTGAATAGGCCTTGAA

*C. lucidus* CTTGGAACTACAATTACATTTGCAAGCTCCCACTGATTTCTCGCCTGAATAGGCCTTGAG

*S. sihama* CTTGGCACCACCATCACCTTCGCGAGCTCACACTGGCTCCTTGCCTGAATGGGGCTTGAA

*C. loricula* ACTGGTACCTCCCTCACATTCGTCAGCTCTCACTGACTTTTAGCCTGAATAGGCCTAGAA

*A. trutta* CTAGGTACTACAATTACACTCGCGAGCTCGCACTGGCTCCTTGCGTGGATAGGACTAGAA

*H. gemma* CTAGGAACAACAATTACCTTCGCGAGCTCACACTGACTTCTAGCCTGAATAGGTCTAGAA

*P. flavescens* CTAGGAACCACAATTACATTTGCTAGCTCACATTGACTGCTCGCCTGAATGGGACTTGAA

*P. macrolepida* CTAGGCACCACAATCACATTCGCGAGCTCTCACTGACTTCTCGCCTGAATGGGCCTTGAA

*S. canadensis* CTAGGCACCACAATTACATTTGCAAGCTCACATTGGCTGCTTGCCTGGATAGGCCTTGAA

*A. rogaa* ATAAATACACTAGCCATCATTCCCCTTATAGCCCAACAGCACCACCCCCGAGCAGTTGAA

*C. argus* ATAAATACACTAGCTATTATTCCCCTCATAGCTCAACAACACCACCCCCGAGCAGTTGAA

*C. sonnerati* ATTAATACCCTAGCCATCATCCCCCTCATAGCCCAACAACATCACCCCCGAGCAGTCGAG

*E. fuscoguttatus* ATTAATACCCTAGCTATCCTTCCCCTTATAGCCCAACAACATCACCCCCGAGCAGTTGAA

*E. coioides* ATCAACACCCTAGCCATCCTCCCCCTTATAGCCCAACAACACCACCCCCGAGCAGTTGAA

*E. bruneus* ATTAATACTTTAGCCATCCTCCCCCTCATAGCCCAGCAACACCACCCCCGAGCAGTTGAA

*E. moara* ATTAATACTCTAGCCATCCTCCCCCTCATAGCCCAACAACACCACCCCCGAGCAGTTGAG

*E. lanceolatus*  ATTAATACCCTAGCCATTCTCCCCCTTATAGCCCAACAACACCACCCTCGAGCAGTTGAA

*A. leucogrammicus*  ATTAATACCCTAGCCATCCTACCCCTTATGGCCCAACAACACCACCCCCGAGCAGTTGAA

*C. altivelis* ATTAACACCCTAGCCATCCTCCCTCTAATAGCCCAACAACACCACCCCCGAGCAGTCGAA

*E. epistictus* ATTAATACCCTAGCCATCCTCCCCCTTATAGCCCAACAGCACCACCCCCGAGCAGTTGAA

*E. octofasciatus* ATAAGCACCCTGGCCATCCTCCCACTCATAGCCCAACAACATCACCCCCGGGCAGTTGAA

*E. septemfasciatus* ATAAATACCCCGGCCATCCTTCCACTCATAGCCCAGCAGCATCACCCCCGAGCAGTTGAA

*T. dermopterus* ATAAATACCTTAGCCATTCTCCCACTTATAGCCCAACACCACCACCCCCGAGCAGTTGAA

*E. awoara* ATTAACACCCTTGCCATCCTCCCCCTCATGGCCCAACAACATCACCCCCGGGCAGTTGAA

*E. akaara* ATCAATACCCTTGCCATCCTCCCCCTTATAGCCCAACAACATCACCCCCGGGCAGTTGAA

*E. trimaculatus* ATTAATACCCTTGCCATTCTACCTCTCATAGCCCAACAGCATCACCCTCGAGCAGTCGAA

*E. areolatus* ATTAACACTCTTGCTATCCTTCCCCTCATAGCCCAACAACATCACCCCCGAGCAGTCGAA

*V. albimarginata* ATAAATACCCTAGCCATTATTCCACTAATAGCCCAACAACACCACCCCCGAGCGGTTGAA

*V. louti* ATAAATACCTTAGCTATTATTCCACTAATAGCCCAACAACACCACCCCCGCGCAGTTGAA

*P. leopardus* ATCAACACCCTAGCCATTATTCCATTAATAGCGCAATACCATCACCCCCGCGCAGTTGAA

*P. areolatus* ATCAACACCCTGGCCATTATTCCATTAATGGCACAATACCACCACCCCCGCGCAGTTGAG

*E. radiosum* ATGAATACTCTAGCCATTATTCCCCTGATGGCACAACACCACCACCCCCGCGCAGTAGAA

*P. sieboldi* ATAAACACCCTTGCCATTATCCCTCTAATAGCTCAACACCACCACCCCCGGGCAGTAGAG

*E. armatus* ATAAATACCCTTGCCATCATTCCTCTTATAGCCCAACACCATCACCCACGAGCCGTGGAG

*R. oxyrhynchus* ATCAACACCCTCGCTATCCTTCCACTGATAGCCCGACATCACCACCCCCGAGCAGTAGAG

*K. cinerascens* ATGAACACCCTCGCCATTATCCCCCTTATGGCCCAACACCACCACCCCCGAGCAGTTGAA

*T. chatareus* ATTAATACCCTAGCCATTATTCCACTCATAGCCCAACACCACCACCCCCGAGCAGTAGAA

*D. berycoides* ATAAATACCCTCGCCATCATTCCCCTAATAACCCAACATCACCACCCACGAGCAGTTGAA

*H. typus* ATAAACACCCTCGCCATTATTCCCCTAATAGCTCAACACCACCACCCCCGAGCAGTCGAA

*M. argenteus* ATAAACACCCTCGCTATTATTCCACTCATAGCCCAACACCACCACCCACGAGCAGTTGAA

*S. chuatsi* ATAAATACGCTCGCCATTATTCCACTTATAGCCCAACACCACCACCCACGAGCAGTAGAA

*O. fasciatus* ATTAATACCCTCGCCATCATTCCCCTTATGGTACAACACCACCACCCCCGAGCTGTTGAA

*P. trilineatum* ATAAATACCCTCGCCATTATTCACTTATTAGCCCAACACCACCACCCACGAGCAGTTGAA

*M. salmoides* ATAAATACCCTAGCCATCATCCCCATTATGGCCCACCTCCACCACCCACGGGCAGTAGAA

*P. tile* ATAAATACCCTTGCAATCATCCCTCTAATAGCCCAACACCACCACCCTCGAGCAGTTGAA

*L. argentimaculatus* ATAAATACCCTAGCCATTATTCCCCTCATAGCCCAACACCATCACCCACGAGCAGTCGAA

*E. struhsakeri* ATAAACACCCTCGCCATTATCCCCTTAATAGCCCAACACCACCACCCACGGGCAGTAGAA

*B. albus* ATTAACACCTTAGCCATCATCCCTCTCATAGCCCGACACTCCCACCCACGAGCAGTCGAA

*C. auripes* ATCAACACTCTTGCCATTATTCCACTAATAGCCCAACACCATCACCCACGGGCAGTTGAA

*C. melampygus* ATCAATACCCTGGCCATTATCCCCTTAATAGCCCAACACCACCACCCCCGAGCGGTTGAA

*L. calcarifer* ATTAATACTCTAGCCATTATTCCCCTGATAACCCAACACCACCACCCCCGAGCCGTTGAA

*S. maena* ATCAACACTTTAGCTATTCTTCCCCTCATTGCCCGACACAGTCACCCCCGAGCCGTCGAG

*P. auriga* ATCAACACTCTAGCCATCCTTCCCCTTATAGCTCAATACCATCATCCGCGCGCGGTCGAA

*C. lucidus* ATTAATACCCTTGCTATTTTACCACTCATAGCCCAACACCACCACCCCCGGGCAACTGAA

*S. sihama* ATTAATACCTTAGCTATTATTCCACTGATAGCCCAACTTCACCACCCCCGAGCCGTAGAG

*C. loricula* ATTAGCACCCTTGCTATCCTTCCGCTCATGGCTCAACACCACCATCCCCGAGCTGTTGAA

*A. trutta* ATCAATACCCTCGCCGTTCTCCCCCTCATGACTCAAAATCATCACCCACGAGCAGTTGAA

*H. gemma* ATTAATACTTTAGCTATTATCCCTCTCATAGCCCAGTCACATAATCCCCGAGCAGTCGAA

*P. flavescens* ATAAATACTCTCGCTATTATTCCCCTGATAGCCCAACATCACCACCCACGAGCAGTTGAA

*P. macrolepida* ATAAATACTCTAGCCATTATCCCCCTTATAGCACAGCATCATCACCCTCGAGCAGTAGAA

*S. canadensis* ATTAATACTCTCGCCATTATTCCCCTAATAGCACAACACCACCACCCACGAGCAGTTGAG

*A. rogaa* GCTACCACTAAATATTTCCTCACCCAAGCAACCGGAGCAGCCACATTACTATTTGCTAGC

*C. argus* GCTGCCACCAAGTATTTTCTTACCCAAGCAACCGGAGCAGCCACACTACTATTTGCCAGC

*C. sonnerati* GCATCCACCAAGTACTTTCTAACCCAAGCAACCGGAGCAGCAACATTACTATTTGCCAGC

*E. fuscoguttatus* GCTACCATAAAATACTTCCTCACCCAAGCAACAGGAGCAGCTACCCTTCTATTTGCCAGC

*E. coioides* GCCACCATAAAATATTTCCTCACTCAAGCAACAGGAGCAGCTACCCTTCTGTTTGCCAGT

*E. bruneus* GCCACCATAAAATATTTCCTTACCCAAGCAACAGGAGCAGCTACCCTCTTATTTGCCAGC

*E. moara* GCCACCATAAAATATTTCCTCACCCAAGCAACAGGAGCGGCTACCCTCTTATTTGCCAGC

*E. lanceolatus*  GCCACCATTAAATATTTTCTTACCCAAGCAACAGGGGCAGCCACCCTACTATTCGCCAGC

*A. leucogrammicus*  GCCACCATAAAATATTTCCTCACCCAAGCAACAGGAGCAGCTACCCTCCTATTTGCCAGC

*C. altivelis* GCCACTATAAAATATTTCCTCGTCCAAGCAACAGGGGCAGCCACCCTGCTATTCGCCAGC

*E. epistictus* GCCACCACAAAATATTTTCTTACCCAAGCAACAGCAGCAGCCACCTTACTATTTGCCAGC

*E. octofasciatus* GCTGCCACAAAATATTTCCTCACCCAAGCAACTGGGGCAGCTACCCTATTATTTGCCAGT

*E. septemfasciatus* GCTACCACAAAATACTTCCTCACCCAAGCAACTGGAGCAGCTACTCTACTATTTGCCAGT

*T. dermopterus* GCTGCCACAAAATATTTTCTCACCCAAGCAACCGGAGCTGCTACCCTATTATTTGCCAGC

*E. awoara* GCCACCACAAAATACTTCCTCACCCAAGCAACAGGGGCAGCTACCCTACTATTTGCTAGC

*E. akaara* GCTACCACAAAATATTTTCTCACTCAAGCAACAGGAGCAGCTACCCTGCTATTTGCCAGC

*E. trimaculatus* GCCACTACAAAATATTTCCTCACCCAAGCAACAGGCGCAGCAACCCTATTATTTGCCACC

*E. areolatus* GCCGCCACAAAGTATTTCCTCACTCAAGCAACAGGGGCAGCAACCCTTCTATTCGCCAGC

*V. albimarginata* GCAGCCACTAAATACTTTCTCACTCAAGCAACAGGAGCGGCCACACTACTTTTCGCCAGT

*V. louti* GCAAGTACTAAATATTTTCTTACTCAAGCAACAGGAGCAGCCACACTACTTTTCGCTAGT

*P. leopardus* GCCACTACTAAATACTTCTTAACCCAAGCAACCGGGGCTGCCACATTACTGTTTGCAAGT

*P. areolatus* GCCACTACCAAATATTTTTTAACCCAAGCAACCGGAGCCGCCATGTTACTATTTGCAAGT

*E. radiosum* GCCACAACTAAGTATTTTCTTACCCAAGCCACTGCGGCGGCCATACTTCTTTTTGCTAGC

*P. sieboldi* GCCACCACAAAATACTTCTTAACTCAGGCCACCGCCGCGGCCATGCTACTATTTGCCAGC

*E. armatus* GCTACCACTAAATATTTTTTGACCCAGGCTACCGCAGCCGCTATGCTTCTTTTCGCCAGC

*R. oxyrhynchus* GCCACTACTAAATATTTCCTAACACAAGCAACTGCAGCCGCCGTACTCCTCTTTGCAAGC

*K. cinerascens* GCCACTACTAAATACTTTTTGACCCAAGCTACCGCAGCAGCCATGTTACTCTTTGCAAGC

*T. chatareus* GCCACCACTAAATATTTCCTTACTCAAGCCACTGCGGCCGCCATATTACTTTTTGCTAGC

*D. berycoides* GCAACCATTAAATATTTCCTAACTCAAGCCACTGCTGCTGCCATACTACTCTTTGCAAGT

*H. typus* GCAACCACTAAATACTTTCTCACCCAAGCCACTGCCGCCGCCATACTACTCTTTGCAAGC

*M. argenteus* GCAACCACCAAATATTTCCTTACCCAAGCAACAGCGGCCGCCATGCTATTATTTGCAAGT

*S. chuatsi* GCAACTACTAAATACTTTCTTACCCAAGCCACCGCAGCCGCCATACTACTTTTTGCCAGC

*O. fasciatus* GCAACCACTAAATATTTTTTAGTCCAAGCCACCGCAGCAGCTATATTACTATTTGCAAGC

*P. trilineatum* GCAACCACTAAATACTTCCTCACCCAAGCAACAGCAGCTGCCATATTACTATTTGCAAGT

*M. salmoides* GCCACCACCAAATACTTTCTTACCCAAGCCACTGCAGCTGCCATACTTCTTTTTGCTAGC

*P. tile* GCAACCACCAAATACTTCCTTACCCAAGCCACTGCCGCCGCAATACTACTATTCGCAAGT

*L. argentimaculatus* GCCACCACTAAGTACTTCCTCACCCAAGCCACCGCCGCCGCGATACTACTCTTTGCAAGC

*E. struhsakeri* GCCACTACAAAATATTTCCTCACCCAGGCAACAGCGGCTGCTATACTACTTTTTGCAAGC

*B. albus* GCAGCCACTAAATACTTCCTCGCCCAAGCCACAGCAGCTGCTATACTCCTTTTTGCAAGC

*C. auripes* GCTACCACCAAATATTTCCTCACCCAAGCAACAGCAGCTGCTACACTCCTTTTTGCAGGC

*C. melampygus* GCAACTACAAAATATTTCCTTACACAAGCTACAGCTGCCGCTATACTCCTATTTGCAAGC

*L. calcarifer* GCAACAACCAAATACTTCCTCACTCAGGCCACCGCCGCAGCCATACTTCTTTTTGCAAGC

*S. maena* GCAACCACTAAATATTTTATTGCCCAAGCTACAGCAGCCACCATACTTTTATTTGCAGGC

*P. auriga* GCAACCACTAAATATTTCATCACCCAAGCCACAGCAGCCGCCATACTATTATTTGCAAGT

*C. lucidus* GCCACCCTTAAATACTTCCTCACACAAGCAACTGCAGCCTCTACCCTTCTCTTTGCTACT

*S. sihama* GCTTCAACAAAATACTTCCTCACACAAGCTACAGCAGCAGCCATACTTCTCTTCTCAGCA

*C. loricula* GCAGCTACTAAGTACTTTCTTATTCAAGCGACGGCAGCCGCCGTACTTTTATTCGCAAGC

*A. trutta* GCTACAACTAAATACTTTCTAATCCAAGCCACTGCAGCTGCCACACTCCTCTTTGCCTCC

*H. gemma* GCCACTACTAAGTACTTCCTGACCCAAGCAACCGCCGCCGCTATACTCCTGTTTGCAAGC

*P. flavescens* GCTACTACTAAATATTTCCTCACCCAAGCAACTGCAGCAGCTATACTTCTTTTTGCCAGC

*P. macrolepida* GCAACAACTAAATATTTCCTAACTCAGGCAACTGCAGCCGCTATACTCCTCTTTGCCAGC

*S. canadensis* GCCACCACTAAATATTTCCTCACCCAAGCAACTGCAGCAGCCATACTTCTCTTTGCCAGC

*A. rogaa* ACCACCAACGCCTGACTAACCGGCCAATGGGACATCCAACAAATGTCTCACCCACTCGCA

*C. argus* ACCACTAACGCCTGACTAACAGGCCAATGAGATATCCAACAAATATCTCACCCCCTTGCA

*C. sonnerati* ACCACCAATGCCTGACTTACTGGCCAATGAGATATTCAACAAATATCTCACCCCATCGCA

*E. fuscoguttatus* ACCACCAATGCATGACTAACAGGCCAATGAGACATCCTACAAATATCCCACCCTCTTGCA

*E. coioides* ACTACCAACGCCTGACTAACAGGCCAATGAGACATCCTACAAATAACCCACCCCCTCTCA

*E. bruneus* ACAACCAATGCATGGCTAACAGGCCAATGAGACATCCTACAAATGTCTCACCCCCTCGCA

*E. moara* ACGACCAATGCATGACTAACAGGCCAATGGGACATCCTACAAATATCCCACCCCCTCGCA

*E. lanceolatus*  ACCACCAATGCATGACTAACAGGCCAATGAGACATCCTACAAATATCTCACCCCTTCTCA

*A. leucogrammicus*  ACTACAAATGCATGACTAACAGGCCAGTGGGATATCCTACAAATATCACACCCCTTTGCA

*C. altivelis* ACCACCAATGCATGACTGACAGGCCAATGAGATATCCTACAAATATCTCACCCCCTCGCC

*E. epistictus* ACCACCAACGCCTGACTAACCGGCCAATGAGATATTCTACAAATATCTCACCCCCTTACT

*E. octofasciatus* ACTACCAATGCGTGGTTAACAGGTCAATGAGATATTCTACAGATATCTCACCCCCTCGCA

*E. septemfasciatus* ACTACTAATGCATGATTAACAGGTCAATGAGATATCCTACAGATATCTCACCCCCTCACA

*T. dermopterus* ACTACTAATGCATGACTAACAGGTCAGTGAGATATCCTACATATGTCCCACCCCCTGGCG

*E. awoara* ACTACCAATGCATGATTAACAGGCCAATGGGATATCCTACAGATATCTCACCCCCTTGCT

*E. akaara* ACTACTAATGCATGATTAACAGGCCAATGAGATATTCTACAAATATCCCACCCCCTTGCA

*E. trimaculatus* ACCACCAATGCATGATTAACAGGACAATGAGATATCCTACAGATATCCCACCCCCTCGCA

*E. areolatus* ACTACCAATGCTTGACTAACAGGACAATGGGATATTTTACAAATATCCCACCCCCTCGCA

*V. albimarginata* ACTACTAATGCATGACTTACAGGACAGTGGGATATTCAACAAATAACACATCCCCTTCCA

*V. louti* ACTTCTAATGCATGGCTCACAGGACAATGAGACATTCAACAAATAACACACCCTCTTCCA

*P. leopardus* ACCACCAATGCATGGCTTACAGGACAATGAGAAATTCAACAAATAACACACCCAGTTCCT

*P. areolatus* ACCACCAATGCATGGCTTTCAGGACAATGAGAAATTCAACAAATAACACACCCAGTCCCT

*E. radiosum* ACCACTAATGCTTGACTTACAGGTCAATGGGATATTCAGCAGATGGCCCATCCTCTTCCC

*P. sieboldi* ACCACTAACGCCTGGCTCACCGGACAGTGGGACATTCAGCAAATGTCGCACCCCCTCCCT

*E. armatus* ACCACTAACGCCTGGCTAACAGGACAATGGGAAATTCAACAAATATCCCACCCCCTACCT

*R. oxyrhynchus* ACAACAAATGCCTGGCTAACAGGACAATGAGACATTTACCAAATGACACACCCCCTCCCC

*K. cinerascens* ACAACCAATGCCTGACTCACAGGACAATGGGACATCCAACAGATATCCCACCCCCTCCCT

*T. chatareus* CTTACAAACGCTTGACTAACAGGACAATGAGATATTCAACAAATAACCCACCCCCTTCCT

*D. berycoides* ACTACTAATGCCTGACTAACTGGACAATGAGACATTCAACAGATGTCCCACCCCCTCCCC

*H. typus* ACTACCAATGCCTGATTAACTGGACAGTGAGACATTCAACAGATATCCCACCCACTTCCT

*M. argenteus* ACTACCAACGCTTGACTGACCGGACAATGAGATATCCTACAGATATCTCACCCCCTCCCA

*S. chuatsi* ACCACTAATGCTTGACTTACCGGACAATGAGACATTCAACAGATATCACACCCCCTCCCC

*O. fasciatus* ATAACCAACGCCTGACTCACAGGACAATGAGACATCCAACAAATATCACACCCTCTCCCT

*P. trilineatum* ACAACAAACGCTTGACTCACAGGACAGTGAGACATTCAACATATAACTCACCCCCTCCCC

*M. salmoides* ACTACTAACGCCTGGCTCACAGGACAATGAGATATTCAACAAATAACCCACCCCCTCCCC

*P. tile* ACCACAAACGCATGACTCACAGGACAATGAGACATCCAACAGATGTCTCACCCCCTTCCC

*L. argentimaculatus* ACCACCAATGCATGACTCACCGGACAATGAGATATCCAACAAATATCCCACCCCCTCCCT

*E. struhsakeri* ACTACCAACGCCTGACTCACAGGACAATGAGATATTCAACAAATATCACACCCACTTCCC

*B. albus* ACTACCAATGCTTGACTGACAGGACAATGAGAAATTCAACAAATATCACACCCCCTCCCC

*C. auripes* ACTACCAATGCCTGACTTTCCGGCCAATGGGACATCCAACAGGTGTCTCACCCATTTCCA

*C. melampygus* ACAACCAACGCCTGACTAACTGGCCAATGAGATATTCAACAAATAACCCACCCGATCCCA

*L. calcarifer* ACTACCAATGCTTGACTCACCGGACAATGAGAAATCCAACAAATAACACATCCCTTCCCA

*S. maena* ATATCAAGCGCCTGGCTTACTGGACTCTGAGAAATTCCCCTGACAGCACACCCTGTCCCT

*P. auriga* GCTATTAATGCCTGACTTGTTGGACAATGAGACATTCAACAAATAGCGCACCCTCTTCCC

*C. lucidus* ACCACAAACGCCTGATTAAACGGACAATGAGATATTCAACATATAACACACCCACTTCCT

*S. sihama* ACTACTAACGCCTGACTAACTGGACAGTGGGACATCCTTCAAATGTCACACCCTCTGCCA

*C. loricula* ACCACTAATGCCTGAATTTCGGGGCACTGGGACCTTCAACAAACAAGCCACCCCTTACCC

*A. trutta* ACCACAAACGCTTGACTAACCGGACAGTGAAGTATTGAACAAATAACACACCCGCTACCC

*H. gemma* ACCACCAACGCCTGATTGACTGGACAATGGATGATTGACCAAATAACTCACCCCTTCCCG

*P. flavescens* ACCACCAATGCCTGACTTACAGGACAATGGGATATTCAACAGATAACCCATCCTCTACCA

*P. macrolepida* ACTACCAACGCTTGATTAACAGGACAGTGGGATATTCAACAAATGTCCCACCCCCTTCCC

*S. canadensis* ACCACTAATGCTTGGCTTACAGGACAATGGGATATTCAACAAATATCTCACCCTCTGCCT

*A. rogaa* ACCTCGATAGTTATCCTAGCTCTCTCCCTAAAAATTGGCCTAGCCCCCTTACACACATGA

*C. argus* ACCACAATAATTATCCTGGCGCTTTCCCTAAAAATTGGTCTAGCCCCCTTACACACATGA

*C. sonnerati* ACCACAATAGTCATCCTAGCCCTTTCCCTAAAAGTAGGCCTCGCCCCCCTACACACATGA

*E. fuscoguttatus* ACCACTATAGCCATTCTTGCCCTCTCCCTAAAAATTGGCCTTGCCCCCCTACATACATGG

*E. coioides* ATTACAATAGTTATTCTTGCCCTCTCCCTAAAAATTGGCCTCGCACCTTTACACACATGA

*E. bruneus* ATCACTATGGTGATTCTTGCCCTCTCCCTGAAGGTTGGCCTTGCCCCATTACACACATGG

*E. moara* ATTACTATGGTGATTCTTGCCCTCTCCCTGAAAGTTGGCCTTGCCCCCTTGCACACATGA

*E. lanceolatus*  ATCACACTAGCCATCCTTGCTCTCTCCCTAAAAATTGGTCTCGCCCCTCTACATACATGA

*A. leucogrammicus*  ACCACTATGGTTATCCTTGCCCTCTCCCTAAAAATTGGTCTCGCCCCCCTACACACATGA

*C. altivelis* ACCACTACAGCCATCCTTGCTCTCTCCCTAAAGATCGGCCTCGCCCCCTTACATACATGA

*E. epistictus* ACCACTATGGCTATCCTCGCCCTCTCCTTAAAAGTAGGCCTCGCCCCGCTACACACATGA

*E. octofasciatus* ACTACTCTAGCTATTCTTGCCCTTTCCCTAAAAATTGGTCTCGCCCCCCTACATACATGA

*E. septemfasciatus* ACCACTCTAGCTATTCTTGCCCTCTCCCTAAAAGTTGGTCTCGCCCCCCTACACACATGA

*T. dermopterus* ACTACTTTAGCTATTCTTGCCCTCTCCCTAAAAATCGGACTCGCCCCCCTACACACATGA

*E. awoara* ACCACCCTAGCTATCCTTGCCCTCTCCCTAAAAGTAGGACTCGCCCCCCTGCACACATGA

*E. akaara* ACCACTCTGGCTATCCTTGCCCTCTCCCTAAAAGTGGGGCTTGCCCCACTACATACATGA

*E. trimaculatus* ACCACTATTGCCATCCTCGCCCTCTCCCTAAAAGTAGGCCTTGCACCATTACACACGTGA

*E. areolatus* ACCACCATCGCTATTCTCGCCCTTTCCCTAAAAGTAGGCCTTGCCCCACTACACACGTGA

*V. albimarginata* ACCACCTTAATTATCCTTGCCCTATCTCTTAAAGTCGGCCTCGCCCCCCTACACTCATGA

*V. louti* ACTACACTGATTATCCTCGCCCTATCCCTCAAGGTTGGCCTTGCCCCCCTGCACTCTTGA

*P. leopardus* ACCACTATAATTATTATAGCCTTGTCATTAAAAATTGGCCTTGCCCCATTGCATACCTGA

*P. areolatus* ACTACAATAATTATCTTAGCCCTGTCCTTAAAAACCGGTCTTGCCCCACTGCACACCTGA

*E. radiosum* ATTACCCTAATTACTCTCGCCCTAGCACTAAAGATTGGTCTTGCACCTGTTCATTCATGG

*P. sieboldi* CTTACCCTAATCACCATAGCCCTAGCTCTGAAGATTGGGCTCGCCCCAGTTCACTCCTGA

*E. armatus* GTTACGATAATCACCCTGGCTCTTGCACTAAAAATCGGGCTTGCTCCTGTTCACTCATGA

*R. oxyrhynchus* ATTACACTCATTACTTTCGCCCTAGCACTTAAAGTTGGTCTTGCACCCGCCCACTTCTGA

*K. cinerascens* ATTACAATAATCACCCTTGCCCTCGCCCTAAAAATTGGACTTGCCCCCGTCCACTCCTGA

*T. chatareus* ACCACTATGATTACCCTCGCTCTAGCACTAAAAATTGGCCTAGCTCCTATACACTCATGA

*D. berycoides* ATTACCATGATTACCCTCGCCCTCGCCCTTAAAATTGGACTCGCCCCAACTCACGCTTGA

*H. typus* GTCACTATAATTACTCTTGCCCTTGCCCTAAAAATTGGACTAGCCCCGGTCCATTCCTGG

*M. argenteus* ATCACCATAATTACCATTGCCCTCGCCCTAAAAATTGGTTTAGCCCCCCTCCACTCCTGA

*S. chuatsi* GTTACCTTAATTACCCTTGCCCTTGCATTAAAAATTGGCCTTGCCCCAGTTCATGCCTGA

*O. fasciatus* ATCACAATAATTACCCTTGCTCTTGCACTAAAAACAGGCCTCGCCCCAGTCCATGCTTGA

*P. trilineatum* GTCACCATAATTACCATTGCTCTTGCCCTAAAAATTGGACTTGCCCCCCTCCACTCATGG

*M. salmoides* GTTACTATAATTACCCTCGCCCTAGCACTAAAAATTGGGCTTGCCCCCGTACACTCCTGA

*P. tile* GTCACCATAATTACTATTGCCCTCGCCCTAAAAATTGGCCTTGCCCCCACACACTCATGA

*L. argentimaculatus* ATTACTATGATTACCATTGCCCTCGCCCTTAAAATTGGTCTTGCCCCCACACACTCATGA

*E. struhsakeri* ATCACAATAATCACCATTGCCCTCGCCCTAAAAATTGGCCTTGCTCCTCTGCATTCGTGG

*B. albus* ATTACAATAATTTCGGCCGCCCTCGCACTAAAAATTGGACTCGCCCCATTTCACTCTTGA

*C. auripes* GCAACCCTACTTACCCTCGCTCTCGCCCTAAAAATTGGCCTTGCCCCCTTCCACACCTGA

*C. melampygus* ACTACAATAATTACACTAGCCCTAGCCCTTAAAATCGGACTAGCCCCAATACACTCTTGA

*L. calcarifer* ACAACCATAATCATTCTCGCCCTAGCACTAAAAGTAGGCCTAGCCCCCATACACTCCTGA

*S. maena* GTGACATTAGCTACCCTTGCCATCGCCTTAAAAATTGGGCTTGCCCCAATACATTCCTGA

*P. auriga* ATTACATTAATCTCCCTCGCCCTAGCCTTAAAAATTGGCCTTGCTCCACTACACTCCTGA

*C. lucidus* ATTACCCTATTCACCATCGCCCTTGCCCTAAAAATTGGCCTAGCCCCCCTCCACATTTGA

*S. sihama* GTCACAATAGTCACTCTTGCCCTTGCCCTAAAGATTGGTCTTGCACCTCTTCACTCGTGA

*C. loricula* CTCACCCTACTCACCCTGGCCCTCGCACTTAAGATTGGACTTGCCCCCCTACACTCATGA

*A. trutta* ACCACCCTGATCACCCTTGCCTTGGCATTAAAAATTGGCCTTGCCCCAGTTCATGCTTGA

*H. gemma* ACAACTCTTATTGTTTTAGCACTAGCCCTAAAGATTGGCTTAGCCCCATTCCACTCTTGA

*P. flavescens* ATTGCCCTTATTACCCTGGCCTTAGCATTAAAAATTGGGCTAGCCCCGCTCCACTCATGA

*P. macrolepida* ATCACCCTCATTACTCTTGCATTAGCACTAAAAATTGGCCTTGCACCTGTTCACTCGTGG

*S. canadensis* ATTACTCTTATTACCCTTGCTTTAGCATTAAAAATTGGCCTTGCACCAGTTCACTCATGG

*A. rogaa* CTACCCGAAGTATTACAAGGCCTAGACCTAACTACAGGCCTAATTCTCTCAACCTGACAA

*C. argus* CTACCCGAAGTCCTTCAAGGCCTAGACTTAATTACAGGCCTAATTCTTTCAACCTGGCAG

*C. sonnerati* CTACCAGAAGTACTTCAAGGATTAGACCTAACCACAGGACTCATTTTATCTACCTGACAA

*E. fuscoguttatus* CTACCTGAAGTACTACAAGGGTTAGACTTAACTACAGGACTTATCTTGTCAACCTGACAG

*E. coioides* CTACCCGAAGTACTCCAAGGGTTAGACTTAACTACAGGTCTCATTCTATCAACCTGACAA

*E. bruneus* CTGCCCGAGGTACTTCAAGGATTAGACTTAACCACAGGCCTCATTTTATCAACCTGGCAA

*E. moara* CTGCCCGAAGTACTTCAAGGGTTAGACTTAACCACAGGCCTCATTTTATCAACCTGGCAA

*E. lanceolatus*  TTACCTGAAGTACTCCAAGGACTAGACTTAACTACAGGACTTATTCTATCAACCTGACAA

*A. leucogrammicus*  CTACCTGAAGTACTCCAAGGGTTAGACCTAACTACAGGACTTATCTTATCGACCTGACAG

*C. altivelis* TTACCTGAAGTACTCCAAGGACTGGACTTAACCACAGGACTCATCTTATCAACATGACAA

*E. epistictus* TTGCCTGAAGTACTTCAAGGGTTAGATCTAACTACAGGACTTATTCTATCAACCTGACAA

*E. octofasciatus* CTGCCAGAAGTACTCCAAGGGTTAGACCTAACTACAGGTCTCATTTTATCGACCTGACAA

*E. septemfasciatus* CTGCCAGAAGTACTCCAAGGGTTAGACCTAACTACAGGTCTCATTTTATCGACCTGACAA

*T. dermopterus* CTACCGGAAGTACTCCAAGGACTAGACCTGACCACAGGACTCATCCTGTCGACCTGACAA

*E. awoara* TTACCCGAAGTGCTCCAAGGATTAGACCTAACCACAGGACTCATCTTATCAACTTGACAA

*E. akaara* TTACCCGAAGTGCTCCAAGGATTAGACCTAACCACAGGACTCATCTTATCGACCTGACAA

*E. trimaculatus* CTACCCGAAGTCCTCCAAGGACTAGACCTAGCTACAGGACTTATCCTATCAACCTGACAA

*E. areolatus* CTGCCCGAAGTACTCCAAGGACTAGACCTAACCACTGGCCTTATTCTATCAACCTGACAA

*V. albimarginata* CTACCTGAAGTCCTCCAAGGCCTTGACCTAGTCACTGGCCTTATTATGTCTACCTGACAA

*V. louti* CTACCTGAAGTTCTTCAAGGCCTTGATCTAACCACCGGCCTTATCATATCCACCTGACAA

*P. leopardus* CTTCCTGAGGTTCTTCAAGGTTTAGACCTCACCACAGGCCTAATCATATCGACCTGACAA

*P. areolatus* CTTCCTGAAGTCCTTCAAGGTTTAGACCTCACTACAGGCTTAATCATATCGACCTGACAA

*E. radiosum* CTTCCTGAGGTTCTTCAGGGGTTAGACCTTACTACCGGACTCATCCTCTCCACCTGACAA

*P. sieboldi* CTCCCTGAAGTCCTTCAAGGCCTAGACCTTACCACAGGCCTTATTCTCTCCACTTGGCAA

*E. armatus* CTTCCCGAAGTCCTTCAGGGCTTAGACCTAACTACCGGCCTTATTCTTTCCACCTGACAA

*R. oxyrhynchus* CTACCTGAAGTCCTCCAAGGCCTAGATCTAACTACTGGTCTCCTCCTCTCCACATGACAA

*K. cinerascens* CTGCCCGAAGTCCTTCAAGGCCTTGATCTCACAACAGGCCTTCTTTTATCCACATGACAA

*T. chatareus* CTCCCAGAAGTCCTACAAGGGTTAGACCTCATAACAGGACTCATCCTCTCTACCTGGCAA

*D. berycoides* CTCCCCGAAGTCCTCCAAGGCCTAGACCTCACTACCGGCCTTATCCTCTCTACCTGACAA

*H. typus* CTCCCCGAGGTCCTCCAAGGCCTGGACCTTACCACGGGCCTCATCCTATCTACCTGACAA

*M. argenteus* CTCCCCGAGGTCCTTCAGGGCCTAGACTTAACTACCGGGCTCATCCTTTCCACCTGGCAA

*S. chuatsi* CTACCTGAAGTCCTTCAAGGATTGGACCTCACCACAGGCCTTATTCTCTCAACCTGACAA

*O. fasciatus* CTGCCAGAAGTCCTTCAAGGACTCGATCTTACTACCGGCCTCCTTCTGTCTACCTGACAA

*P. trilineatum* TTACCAGAAGTTCTTCAAGGCCTAGACCTAACCACCGGGCTTCTTCTTTCCACCTGGCAA

*M. salmoides* CTTCCAGAAGTACTTCAAGGACTGGATCTCACCACAGGCTTAATCCTATCAACCTGACAA

*P. tile* CTCCCAGAAGTCCTTCAAGGATTAGACCTTACTACTGGGCTTATCCTCTCAACCTGACAA

*L. argentimaculatus* CTGCCAGAAGTCCTACAAGGACTAGACCTCACTACCGGACTCATCCTCTCAACCTGACAA

*E. struhsakeri* CTACCTGAAGTTCTCCAAGGACTGGACCTTACTACAGGACTTATTCTGTCTACCTGACAA

*B. albus* ATGCCCGACGTCCTTCAAGGCCTAGATCTCACCACAGGCCTTATTATAACAACCTGACAA

*C. auripes* CTACCTGAAGTCCTTCAAGGACTTGACTTAACCACCGGCCTAATCCTTTCTACATGACAA

*C. melampygus* CTCCCCGAGGTTCTCCAAGGCTTAGACCTTACCACAGGCCTTATTTTATCCACATGACAA

*L. calcarifer* CTCCCCGAAGTATTACAAGGACTTAGCCTCACTACCGGGCTTATCCTATCCACTTGACAA

*S. maena* CTCCCAGAAGTTCTTCAAGGAACCGATTTTACTACGGGCCTAATTTTATCCACCTGGCAG

*P. auriga* CTCCCCGAGGTTATCCAAGGCGTTGACCTCATTACAGGCTTAATTCTCTCTACCTGACAA

*C. lucidus* CTCCCTGAAGTCCTCCAAGGACTAGACCTTGTAACAGGACTTATTATATCCACCTGACAA

*S. sihama* CTTCCAGAAGTGCTTCAAGGGCTAGATCTCACCACAGGCCTCGTCCTGTCCACATGACAA

*C. loricula* CAACCTGAGGTCCTCCAGGGCCTAAACTTAAACACGGGCATTATCCTAGCCACATGACAA

*A. trutta* CTCCCCGAGGTCCTTCAAGGACTAGACCTTACCACAGGCTTAATCTTATCTACCTGACAA

*H. gemma* CTACCAGAAGTGCTTCAGGGATTAGACTTAACAACAGGGATAATCCTTTCTACCTGGCAA

*P. flavescens* CTTCCTGAAGTTCTCCAAGGATTAGATCTTACTACCGGACTAATCCTCTCCACCTGACAA

*P. macrolepida* TTACCTGAAGTTCTTCAGGGACTAGATCTTACGACCGGACTCATCCTCTCCACCTGACAA

*S. canadensis* CTACCCGAAGTCCTTCAAGGCCTCGACCTTACCACAGGTCTCATTCTTTCTACTTGGCAA

*A. rogaa* AAACTAGCCCCATTCGCCCTACTTACCCAAATTCAA------------CCCGCCAAC---

*C. argus* AAACTAGCCCCCTTTGCCCTACTTATTCAAATTCAA------------CCTACTAAC---

*C. sonnerati* AAACTGGCCCCCTTCGCCCTACTCATCCAAATTCAA------------CCCGCCAGC---

*E. fuscoguttatus* AAATTAGCACCCTTCGCCCTACTTCTCCAAATTCAA------------CCCACCAAC---

*E. coioides* AAACTGGCACCCTTCGCCCTACTCCTTCAAATTCAA------------CCCACCAAC---

*E. bruneus* AAGCTAGCACCCTTCGCCCTATTTCTTCAGATTCAA------------CCCACTAAC---

*E. moara* AAACTAGCACCCTTCGCCCTATTTCTTCAAATTCAA------------CCCGCCAAC---

*E. lanceolatus*  AAACTAGCACCATTCGCCCTACTCCTTCAAATTCAA------------CCCACCAAC---

*A. leucogrammicus*  AAACTAGCACCTTTTGCCCTACTCCTTCAAATCCAA------------CCCACCAAC---

*C. altivelis* AAACTGGCACCCTTCGCCCTACTTATTCAAATTCAA------------CCCACCAAC---

*E. epistictus* AAATTAGCTCCCTTCGCCCTACTCCTTCAAATCCAA------------CCCGTCAAC---

*E. octofasciatus* AAATTGGCCCCCTTCGCACTACTTCTTCAAATCCAA------------CCTACCAAC---

*E. septemfasciatus* AAACTGGCCCCCTTTGCACTACTTCTTCAAATCCAA------------CCCACCAAC---

*T. dermopterus* AAACTAGCCCCCTTCGCCCTACTTCTCCAAATACAA------------CCCACCAAC---

*E. awoara* AAGCTAGCACCCTTCGCCCTACTTCTTCAAATCCAA------------CCTGCCGAC---

*E. akaara* AAACTAGCACCCTTCGCCCTACTTCTTCAAATTCAA------------CCTGCTGAC---

*E. trimaculatus* AAACTAGCGCCCTTCGCCCTACTTCTCCAAATTCAG------------CCCACCAAC---

*E. areolatus* AAACTAGCACCCTTCGCCTTACTCCTTCAAATTCAG------------GCCACTAAC---

*V. albimarginata* AAGCTTGCCCCTTTTGCCCTCTTAATTCAAATTCAA------------CCTGCAGAC---

*V. louti* AAGCTTGCCCCTTTCATCCTCCTAATTCAAATTCAA------------CCCGCAGAT---

*P. leopardus* AAACTAGGACCCTTCGCACTACTCCTTCAAATACAA------------TCTGCACAC---

*P. areolatus* AAACTCGCACCATTTGCTCTGCTCCTTCAAATACAA------------CCTGCACAC---

*E. radiosum* AAACTCGCCCCCTTTGCCCTTTTGCTTCAAATTCAA------------CCAGCTAAC---

*P. sieboldi* AAACTTGCCCCCTTCGCCCTCCTTCTGCAGATTCAG------------CCCACTAGC---

*E. armatus* AAACTTGCCCCCCTAGCCCTCCTCCTCCAAATTCAA------------CCTGCTAAC---

*R. oxyrhynchus* AAACTAGCACCCTTCGCCCTGTTACTCCAGATTTGC------------CCCTCCAAC---

*K. cinerascens* AAACTAGCCCCCTTTGCCTTACTTCTACAAATTCAA------------CCCACCAAC---

*T. chatareus* AAACTTGCCCCATTTGCCCTCCTACTACAAATTCAA------------CAAACCACT---

*D. berycoides* AAACTTGCCCCTTTCGCCCTCCTCCTACAAATCCAG------------CCCACAAAT---

*H. typus* AAACTTGCCCCCTTCGCACTTCTACTACAAATTCAA------------CCCACCAAC---

*M. argenteus* AAACTTGCCCCCTTCGCCCTCCTCCTACAAATCCAG------------CCTACTAAT---

*S. chuatsi* AAACTTGCCCCTTTCGCCCTGCTTCTCCAAATTCAA------------CCGGCCAAT---

*O. fasciatus* AAACTGGCTCCCTTTGCCCTACTCCTGCAAATTCAA------------CCCACCAAC---

*P. trilineatum* AAACTTGCTCCTTTCGCTCTGCTACTACAAATTCAA------------CCGACCAAC---

*M. salmoides* AAACTTGCCCCTTTTGCCCTTCTCCTTCAAATTTAC------------CCCGCAAAC---

*P. tile* AAACTAGCCCCCTTTGCCCTTCTTCTTCAAATTCAA------------CCCGCCAAC---

*L. argentimaculatus* AAACTGGCCCCCTTCGCCCTTCTCCTCCAAATTCAG------------CCCACCAAC---

*E. struhsakeri* AAACTTGCCCCTTTCGCCCTCCTTCTTCAGATCCAA------------CCAACCAAC---

*B. albus* AAACTAGCCCCCTTTGCCCTCCTCCTCCAAATACAA------------CCCGCCAAC---

*C. auripes* AAACTTGCCCCCTTCGCCCTACTTATCCAAATTCAA------------CTTGTTAAC---

*C. melampygus* AAACTTGCTCCCTTCGCCCTATTCCTACAACTTCAA------------CCCAATAAC---

*L. calcarifer* AAATTAGCCCCATTTGCCCTCCTCCTGCAAATCCAA------------CCCGCCGAT---

*S. maena* AAACTCGCCCCAATTGCCCTCCTCTTGCAATTCCAA------------CCTACAAAC---

*P. auriga* AAACTTGCCCCCTTCGCCCTCCTCCTCCAACTCCAG------------CCCGCCAAC---

*C. lucidus* AAACTTGCCCCCTTCGCCCTCCTCCTTCAAATTTAT------------CCAGCCAAC---

*S. sihama* AAGCTAGCCCCTTTCGCACTACTTCTGCAAATCCAA------------CCAGAGAAC---

*C. loricula* AAGCTAGCCCCATTTGCTATTCTCCTTCAAATCCAA------------ACCCCTAAC---

*A. trutta* AAAATTGCTCCTTTTATTCTCCTCGTACAAATTAAC------------TCCCCCGAC---

*H. gemma* AAACTCGCCCCATTCTCCCTGCTCCTACAACTTCCTATTTCCCCCTCCTCGCAAACAATT

*P. flavescens* AAACTCGCCCCATTTGCCCTATTACTTCAAATTCAA------------CCTGCCAAC---

*P. macrolepida* AAACTTGCCCCCTTTGCCCTCCTGCTTCAGATTCAA------------CCCGCCAAC---

*S. canadensis* AAACTCGCCCCTTTTGCCCTTCTACTACAACTTCAA------------CCTGCTAAC---

*A. rogaa* CCAACCCTCCTAATTATTCTTGGTATCACTTCTACCCTTGTAGGAGGATGAGGAGGCCTT

*C. argus* TCAACTATCCTAATTATTTTAGGTATTGCCTCCACCCTTGTAGGGGGGTGAGGGGGCCTT

*C. sonnerati* CCAACCATTCTCATTACGCTTGGAATCACCTCTACCCTTGTAGGAGGCTGAGGAGGCCTT

*E. fuscoguttatus* CCCTCAATCCTAGTAATACTTGGTGTTACCTCAACTCTTGTTGGTGGTTGAGGAGGGCTT

*E. coioides* CCCCTAATTTTAGTAATACTCGGTATTACCTCAACCCTAGTTGGTGGCTGAGGAGGGCTG

*E. bruneus* CCCCAAATCTTAATCATACTGGGCATTACCTCAACCCTAGTTGGTGGCTGAGGAGGGCTT

*E. moara* CCCCAAATCTTAATTATACTGGGCATTACCTCTACCCTTGTCGGTGGCTGAGGGGGGCTA

*E. lanceolatus*  CCCCTAATCTTAATGATACTCGGCATTACCTCAACCCTCGTTGGTGGTTGAGGAGGGCTA

*A. leucogrammicus*  CCTTCAATCCTAGTAATCCTAGGAATTACCTCAACCCTTGTTGGCGGTTGAGGAGGCCTT

*C. altivelis* CCTCTGATCCTAGTAGTACTCGGTATTACTTCAACCCTTATTGGTGGTTGAGGGGGGCTC

*E. epistictus* CCCTCTATTCTGATTATACTTGGTATTACCTCCACCTTTGTTGGCGGCTGAGGAGGACTA

*E. octofasciatus* CCTTCAATCTTAATCATGTTAGGCATTATCTCAACCCTTGTTGGAGGCTGAGGAGGGCTT

*E. septemfasciatus* CCTTCAGTCTTAATTATACTAGGTGTTATCTCAACCCTTGTTGGGGGATGAGGAGGGCTT

*T. dermopterus* CCCTCAATTTTAATCATACTTGGAGTTGCCTCAACCCTTGTCGGCGGCTGAGGTGGACTC

*E. awoara* CCCACAATTTTAATTATCCTCGGTGTCACCTCAACCCTCGTTGGAGGCTGAGGAGGGCTT

*E. akaara* CCCACAATCTTGATCATTCTTGGCATTACCTCAACCCTTGTTGGAGGCTGAGGGGGGCTT

*E. trimaculatus* CCTGCAATCCTAATCACTCTTGGCATTGCCTCAACCCTAATTGGAGGTTGAGGAGGTCTC

*E. areolatus* CCCACAATCCTAATTATCCTCGGCATTACCTCCACCTTAATTGGAGGCTGAGGGGGGCTC

*V. albimarginata* CCTAACCTTCTCATTGTCTTGGGCCTAACCTCCACCCTTGTAGGGGGCTGAGGTGGGCTT

*V. louti* CCTAGCCTCCTTATTGTCTTAGGCCTAACCTCTACTCTTGTAGGAGGCTGAGGTGGACTT

*P. leopardus* CCCACTATTCTCATCATCTTGGGTCTCACTTCAACGCTCGTAGGCGGCTGAGGAGGCCTA

*P. areolatus* CCCGCTATTCTCGTCATTTTAGGTCTCACCTCAACGCTTGTTGGTGGCTGAGGAGGTTTA

*E. radiosum* CCCACTATTCTTATTGCTTTTGGTTTGGCATCCACACTTGTTGGGGGCTGGGGAGGTCTA

*P. sieboldi* TCGGCCACCCTCATTGCTCTGGGGGTAGCCTCTACCCTTGTAGGAGGATGAGGCGGCTTG

*E. armatus* TCAACAATTCTGATTTTCCTCGGCCTTATGTCAACCCTAGTCGGAGGCTGAGGTGGCCTT

*R. oxyrhynchus* TCCCCCCTCCTAATCGCACTTGGCCTTGCCTCTACGCTCATTGGCGGATGGGGTGGATTA

*K. cinerascens* TCGACCATCCTAATTATACTAGGGCTAGCATCCACCCTCGTGGGAGGATGAGGTGGTCTA

*T. chatareus* GCCCCCACACTCATCATGTTAGGACTCGCATCCACCCTTATTGGTGGCTGAGGCGGCTTA

*D. berycoides* TCAACAATCCTGATTGTACTGGGACTTACATCAACCCTCGTAGGCGGCTGAGGGGGTCTA

*H. typus* TCAACTCTCCTTGTCATACTAGGCCTCGCCTCTACCCTTGTAGGGGGCTGAGGAGGACTA

*M. argenteus* TCTACCATTCTAATCGTACTGGGACTTACGTCTACCCTCGTTGGTGGCTGAGGAGGCCTC

*S. chuatsi* TCAACCATTTTAATTATTCTAGGACTCATGTCCACCCTAGTTGGGGGCTGAGGTGGTCTT

*O. fasciatus* TCAACACTCCTTATTTTATTAGGCTTATCATCCACCCTTGTAGGAGGATGAGGAGGCTTA

*P. trilineatum* TCAACAATCCTTATTATTCTAGGGATTGCTTCAACCCTTGTTGGGGGCTGAGGAGGACTA

*M. salmoides* TCAACCCTCCTTATTATTTTAGGCCTGCTATCAACACTAGTGGGAGGCTGAGGGGGCCTA

*P. tile* TCATCACTCCTAATCTTATTAGGCCTTATATCGACCCTCGTCGGGGGATGAGGTGGACTA

*L. argentimaculatus* TCATCCCTACTCATCATTCTAGGCCTAATGTCTACCCTTGTCGGAGGATGAGGGGGACTA

*E. struhsakeri* TCAACCATCTTGATTGCACTAGGACTTGCGTCAACCCTCGTCGGAGGGTGAGGAGGACTA

*B. albus* TCCACCATACTATTAATTTTGGGACTAGCCTCAACCTTAATTGGTGGCTGAGGAGGGCTA

*C. auripes* CCAAGCCTAGTAATACTTCTAGGCCTCCTTTCTATTTTAGTAGGGGGCTGAGGAGGACTT

*C. melampygus* CCCACCCTCCTAATTCTCCTAGGCATTTCATCAACCCTAATTGGAGGCTGAGGTGGACTA

*L. calcarifer* CCCTTCATCCTCATTACATTGGGCCTGACCTCAACTCTAGTCGGCGGCTGAGGAGGACTT

*S. maena* TCGTCACTCTTGATTGCTTTAGGAATCTCATCTATTATGATTGGAGGCTGAGGAGGTATT

*P. auriga* TCATCACTCCTACTAGCCTTAGGAATTATATCGGCCCTTACTGGAGGCTGAGGTGGCCTA

*C. lucidus* TCTACCCTCCTCATTATACTAGGCTTAGCATCAACCCTTATTGGAGGCTGAGGCGGACTA

*S. sihama* TCGCTTCTCCTGATTGCGCTAGGGCTTATTTCCACTTTAATTGGGGGGTGGGGAGGCCTA

*C. loricula* TCACCCCTCCTTATTACCCTAGGCATTGCCTCGATTTTTGTGGGGGGATGAGGGGGACTG

*A. trutta* CCAACCATCCTAATGGCGCTAGGCCTTATATCCACCCTCGTTGGCGGCTGGGGCGGACTG

*H. gemma* CCTGGCCTGATTGTCTTGCTAGGTGCGATTTCTTTACTAGTAGGAGGCTTTGGAGGCCTA

*P. flavescens* TCATCAATCCTTATCATTTTAGGTTTAATATCCACCCTTGTAGGCGGCTGAGGTGGACTA

*P. macrolepida* TCAACAATTCTTATTGCTTTTGGCCTGGCCTCCACCCTTGTTGGAGGATGAGGAGGACTA

*S. canadensis* TCAACTATTCTTATTATTTTAGGCCTAACCTCCACCCTAGTGGGTGGCTGGGGAGGACTA

*A. rogaa* AATCAAACACAGCTCCGAAAAGTCCTTGCTTACTCTTCCACCGCCCACCTAGGCTGAATA

*C. argus* AACCAAACACAACTTCGAAAAATCCTCGCTTACTCTTCCACCGCCCACCTAGGCTGAATG

*C. sonnerati* AACCAAACGCAACTACGAAAAATCCTAGCTTACTCCTCCACCGCCCACTTAGGCTGAATA

*E. fuscoguttatus* AATCAAACACAACTCCGAAAAATTCTAGCATACTCCTCCACAGCCCACCTAGGCTGAATG

*E. coioides* AATCAAACACAGCTCCGGAAAATTCTAGCATACTCCTCTACAGCCCACTTAGGCTGAATA

*E. bruneus* AATCAAACACAACTCCGAAAAATCCTAGCATACTCCTCCACAGCCCACTTAGGCTGAATA

*E. moara* AATCAAACACAACTCCGAAAAATCCTAGCATACTCCTCCACAGCCCACTTAGGCTGAATA

*E. lanceolatus*  AACCAAACACAACTCCGAAAAATTCTAGCATACTCCTCCACAGCCCACTTAGGTTGAATA

*A. leucogrammicus*  AACCAAACACAACTCCGAAAAATTTTAGCATACTCTTCCACAGCCCACTTAGGCTGAATA

*C. altivelis* AATCAAACACAACTCCGAAAAATCCTAGCATACTCCTCCACAGCACACCTAGGCTGAATG

*E. epistictus* AACCAAACACAACTACGAAAAATCCTGGCATACTCTTCCACAGCCCACTTAGGCTGAATA

*E. octofasciatus* AATCAAACACAGCTCCGAAAAGTCCTGGCATACTCCTCTACAGCTCACCTAGGCTGAATA

*E. septemfasciatus* AATCAAACACAACTCCGAAAAGTCCTAGCATACTCCTCTACAGCTCACCTAGGCTGAATA

*T. dermopterus* AACCAAACACAACTCCGAAAAGTCCTAGCCTACTCCTCTACAGCCCACTTAGGCTGAATA

*E. awoara* AATCAAACACAGCTCCGGAAAATTATAGCATACTCCTCCACAGCCCACTTAGGCTGAATA

*E. akaara* AACCAAACACAACTTCGAAAAATCATAGCATACTCCTCCACAGCCCACCTAGGTTGAATA

*E. trimaculatus* AACCAAACACAACTTCGAAAAATTATAGCATACTCCTCCACAGCCCACCTAGGTTGAATA

*E. areolatus* AACCAAACACAACTCCGAAAAATTATAGCATATTCCTCTACAGCTCACCTGGGTTGAATA

*V. albimarginata* AATCAAACCCAACTCCGTAAAATCCTAGCCTACTCCTCAATTGCCCACCTGGGCTGGATA

*V. louti* AACCAAACCCAGCTTCGTAAAATCCTAGCCTACTCCTCAATTGCCCACCTCGGTTGAATA

*P. leopardus* AACCAAACACAACTCCGAAAAATCCTAGCCTATTCATCAATTGCCCACCTAGGCTGAATA

*P. areolatus* AACCAGACACACCTCCGAAAAATCCTAGCCTACTCATCAATTGCCCACTTAGGCTGAATG

*E. radiosum* AATCAAACACAGCTCCGTAAAATTCTTGCCTATTCATCCATCGCCCATCTTGGCTGAATA

*P. sieboldi* AATCAGACACAACTTCGGAAGATCCTAGCCTATTCTTCCATTGCCCACCTAGGCTGAATA

*E. armatus* AATCAAACTCAATTACGGAAAATCCTCGCATACTCTTCAATTGCACACCTCGGCTGAATG

*R. oxyrhynchus* AACCAAACACAACTACGTAAGATCCTCGCATACTCCTCCATCGCCCACCTTGGCTGAATA

*K. cinerascens* AACCAAACCCAGCTACGTAAAATCCTTGCATACTCCTCAATCGCCCACCTTGGCTGAATG

*T. chatareus* AACCAAACACAACTACGAAAAATCCTCGCCTACTCTTCAATTGCCCACCTCGGCTGAATG

*D. berycoides* AACCAAACCCAACTACGAAAAATCCTTGCCTACTCCTCAATCGCTCACCTTGGCTGAATA

*H. typus* AACCAGACCCAACTACGGAAAATTCTTGCCTACTCCTCAATCGCGCATCTAGGCTGAATA

*M. argenteus* AACCAAACTCAACTGCGAAAAATTCTCGCCTACTCCTCAATTGCACACCTAGGCTGAATA

*S. chuatsi* AACCAAACTCAACTACGAAAAATCCTTGCCTACTCCTCAATCGCCCATCTAGGCTGAATA

*O. fasciatus* AACCAAACCCAACTGCGTAAAATCCTCGCCTATTCCTCAATTGCACACCTCGGCTGAATG

*P. trilineatum* AACCAGACTCAACTACGAAAAATTCTCGCCTACTCCTCAATTGCCCACCTAGGTTGAATA

*M. salmoides* AACCAAACCCAGCTCCGAAAAATCCTTGCCTATTCCTCCATCGCCCATCTTGGATGAATA

*P. tile* AACCAAACACAACTACGGAAAATCCTTGCCTATTCATCAATCGCCCACCTTGGTTGGATG

*L. argentimaculatus* AACCAAACACAACTACGAAAAATCCTCGCCTACTCATCAATTGCTCACCTCGGCTGAATG

*E. struhsakeri* AACCAAACACAACTACGAAAGATCCTCGCCTACTCCTCAATTGCTCACCTTGGCTGAATA

*B. albus* AACCAAACACAACTACGAAAAATCCTCGCTTACTCCTCAATCGCACACCTCGGATGAATA

*C. auripes* AATCAAACCCAACTCCGAAAGATCCTCGCATATTCTTCAATTGCCCACCTCGGCTGAATA

*C. melampygus* AACCAAACGCAACTTCGTAAAATCCTGGCATACTCCTCAATCGCCCATCTAGGCTGAATA

*L. calcarifer* AACCAAACACAACTACGAAAAATCCTTGCTTACTCTTCAATCGCTCACCTGGGCTGAATG

*S. maena* AACCAAACCCAAACCCGTAAGATCCTGGCCTACTCATCAATTGCTCACCTAGGATGAATG

*P. auriga* AATCAAACCCAATTGCGAAAAGTCCTTGCCTACTCCTCTATTGCTCACCTCGGCTGAATA

*C. lucidus* AACCAAACCCAACTACGAAAAATCCTTGCCTATTCCTCAATCGCCCACCTCGGCTGAATA

*S. sihama* AACCAAACCCAGCTGCGCAAGATTCTAGCCTACTCCTCCATCGCCCACCTCGGCTGAATG

*C. loricula* AACCAAACCCAACTACGAAAAGTGCTCGCCTACTCCTCAATTGCCCACTTAGGTTGAATG

*A. trutta* AACCAAACCCAGCTGCGTAAAATTCTAGCTTACTCCTCAATTGCCCACCTAGGTTGAATA

*H. gemma* AATGAAATTCAACTTCGCAAGCTTCTAGCTTACTCCTCTATTGGTCATATGGGTTGAATA

*P. flavescens* AACCAAACCCAGCTACGTAAAATCCTTGCTTATTCCTCAATTGCCCACCTTGGCTGGATA

*P. macrolepida* AATCAAACTCAGCTTCGTAAGATTCTTGCCTACTCATCCATCGCCCACCTTGGCTGAATG

*S. canadensis* AATCAAACCCAACTTCGTAAAATTCTTGCTTATTCCTCAATTGCTCACCTAGGTTGGATA

*A. rogaa* ATTTTAGTCCTACAATTTTCACCCCCGCTAACACTACTTACCCTCGTCACATATCTAATT

*C. argus* ATTTTAGTTCTACAATTTTCACCCCCGCTGACATTGCTGGCCCTCCTTACATATTTAATT

*C. sonnerati* ATCCTCATCCTACAATTCTCCCCTTCACTAACAATATTAACCCTCCTTACATACCTAATC

*E. fuscoguttatus* ATTCTAATTCTCCAATACTCACCCTCCTTAGCCCTCCTAACCCTAATTACCTACCTAATC

*E. coioides* ATTTTAATTCTACAATTCTCACCCTCCTTAGCCTTCTTAACCCTAATTACATACCTAATC

*E. bruneus* ATTTTAATCCTCCAATTCTCGCCCTCCTTAGCCCTCCTGACCTTAATTACCTACTTGATC

*E. moara* ATTTTAATCCTCCAGTTCTCACCCTCCTTAGCCCTCCTAACCTTAATTACATACTTGATC

*E. lanceolatus*  ATTTTAATCCTCCAATTCTCCCCCTCACTGGCCCTCCTAACCCTGGTTATGTACTTAATC

*A. leucogrammicus*  ATCCTCATCCTCCAATTCTCACCCCCTCTCACCCTTCTAACCCTAATCACATACCTAGTC

*C. altivelis* GTTCTTATCCTCCAATTCTCACCCTCCCTAGCCCTCCTAACCCTAGCCACATACCTAATC

*E. epistictus* ATTCTGGTACTCCAATTCTCACCCTCCCTAACCCTCCTAACCTTGCTCACATATTTAATC

*E. octofasciatus* ATTTTAATCCTTCAATTCTCACCCACCTTGACCCTCCTAGCCCTGCTTACATACCTAATC

*E. septemfasciatus* ATTCTAATCCTTCAATTCTCACCCACCTTGACCCTCCTAGCCCTACTCACATACCTAATC

*T. dermopterus* ATCTTAATTCTTCAATTTTCTCCCTCCTTAACACTCCTAACCCTGCTTACATACCTAGTC

*E. awoara* ATTCTGATCCTTCAGTTCTCACCCTCTTTAACCCTCCTAACCCTACTTACATACCTAATT

*E. akaara* ATCCTTGTCCTTCAATTCTCACCCTCTTTAACCCTCCTTACCTTATTTACATACCTAGTC

*E. trimaculatus* ATTCTAGTCCTCCAATTCTCACCCTCACTAACCCTCCTAACCCTATTTACCTACTTAATT

*E. areolatus* ATCTTAGTCCTTCAATTCTCCCCCACCCTAACCCTTCTAACTCTGCTAACGTACCTAGTA

*V. albimarginata* ATTTTGGTACTCCAATTCTCACCTTCCTTAACACTCCTTACCCTTCTTACATACCTCGTA

*V. louti* ATCCTTATTCTCCAATTCTCGCCTTCTTTAACACTCCTTACCCTCCTTACATATCTCGTA

*P. leopardus* ATTTTAGTACTACAATTCTCCCCCTCACTTACGCTTCTAACCCTGATAACATACCTAGTT

*P. areolatus* ATCTTAGTACTACAATTCTCCCCCTCACTCACACTCCTGGCCCTAATAACATACCTGGTA

*E. radiosum* ATTCTTGTTCTTCAGTTCTCCCCCTCTTTAACCCTACTTACCCTACTAACATATTTTGTA

*P. sieboldi* ATTTTGGTCCTCCAGTTCGCGCCTTCCCTCACCCTACTGACCCTTCTCACCTACTTCGTG

*E. armatus* ATTTTAGTTTTACAATTCTCCCCCTCCCTAATGCTCTTGACCCTCCTCACCTATATTATC

*R. oxyrhynchus* ATCCTAGTTCTTCAATTTGTCCCCTCCCTAACCCTCCTTACTCTCCTGACATATTTTGTA

*K. cinerascens* ATTCTAGTCCTACAATTCTCCCCTTCCCTCACCCTCCTAACCCTTCTGACATACTTCGTA

*T. chatareus* ATCCTAGTCCTCCAATTTTCACCCGCACTTACCCTATTAACACTCCTCGTATACCTAATC

*D. berycoides* ATTCTTATTCTACAATTCTCCCCTTCACTCACCCTTCTAACCCTTATCACATACTTTATT

*H. typus* ATTTTAATTCTACAATTTTCCCCCTCCCTCACCCTCCTAACCCTTCTTACCTACTTCATC

*M. argenteus* GTTTTAATTTTACAATTTTCCCCTTCACTCACCTTCATGACCCTTATCACCTACTTTATC

*S. chuatsi* ATCCTAATCTTACAATTTTCCCCCTCCCTTACACTGTTGGCTCTTCTCACGTACTTTATT

*O. fasciatus* ATCCTAATTCTGCAATTTTCTCCCTCTCTCACTCTCTTAACCCTCCTAATATACTTTATA

*P. trilineatum* GTGTTAGTTCTACAATTCGCCCCTTCCCTCACACTCCTTACCCTAATCACATATTTTATC

*M. salmoides* ATCCTCATCCTACAATTTTCCCCCTCCCTAACCCTTCTAGCTCTCCTTACATACCTCGTC

*P. tile* ATCCTAGTACTACAATTTTCCCCCTCCCTCACCCTTTTAACCCTTCTTACATACCTTGTT

*L. argentimaculatus* ATCCTCGTACTACAGTTCTCTCCCTCCCTCACACTACTAACCCTCATCACATACTTCATC

*E. struhsakeri* GTCTTAGTACTCCAATTTTCCCCCTCTCTCACCTTCCTAACCCTCCTCACATACTTTATT

*B. albus* GCCCTAATTCTACAATTTATACCCTCCCTCACACTCCTAGCCCTACTCACGTACTTCATT

*C. auripes* ATTCTAGTATTACAATTCTCCCCTTCCCTTGCCCTCCTCACCCTAATAGTATACTTTATT

*C. melampygus* ACTCTCATCATCCAATTCTCCCCTTCCCTGACCCTTTTAACCCTCTTAACCTATTTTGTT

*L. calcarifer* ATACTCATCCTCCAATTCTCCCCGACCATCACACTTCTTACCCTAATTACATATCTCCTG

*S. maena* ACCCTCGTCCTACAATTCTCTCCATCTCTTGCAATACTTTCCCTTATTATTTATTTCAAC

*P. auriga* ATTCTTGTTATGCAATTCTCCCCCTCCCTCGCCATTCTGGCCCTTATTGTTTACTTCATT

*C. lucidus* ATTCTCGTCCTACAATTCTCCCCTGCCCTCACCCTCTTAACCCTCTTCCTATATATTATT

*S. sihama* ATCCTAGTCCTGCAGTTTTCGCCCGCCCTTACCCTTCTCTCCCTCCTCACCTACTTCGTA

*C. loricula* ATATTAGTGTTACAATATTCACAACCTCTAGCCTTACTCGCTCTCCTGCTCTATATTATT

*A. trutta* ATTTTAATCATGCAATTCTCACCTACCCTTGCCCTCCTTGCCCTTATAACCTACTTCATC

*H. gemma* ATCCTTGTAAAAGGCTTCTGCCCGCAAATTATGCTCTTTACGCTTTGCATTTATATCGTC

*P. flavescens* ATTCTTGTACTTCAATTCTCCCCCTCCCTCACACTTCTTACACTACTAACATATATCGTT

*P. macrolepida* ATTCTTGTACTTCAATTCTCCCCCTCCCTAACCCTTCTCACCCTACTGACATATTTTATT

*S. canadensis* ATTCTTGTACTTCAATTCTCACCCTCTCTAACACTCCTTACCCTACTGACATACCTTATT

*A. rogaa* ATAACATCATCAACATTCCTTGTATTCAAGCTTAACAAGGCAACAAGCATTAATATGCTT

*C. argus* ATAACATCATCAACATTCCTTGTATTCAAACTTAACAAATCGACAAGCATTAACATACTT

*C. sonnerati* ATAACATCATCAACATTCCTTGTATTTAAATTAAATAAAGCCACAAACATTAACATACTT

*E. fuscoguttatus* ATAACATCCTCAACATTCCTTGTATTCAAACTAAACAAATCAACAAACATTAATATACTC

*E. coioides* ATAACATCCTCAACATTTCTCGTGTTTAAACTAAACAAATCAACAAACATTAACATACTC

*E. bruneus* ATAACTTCCTCAACATTCCTTGTATTCAAACTAAACAAATCAACAAGCATCAACATACTC

*E. moara* ATAACATCCTCAACATTCCTTGTGTTCAAACTAAACAAATCAACAAGCATCAACATACTC

*E. lanceolatus*  ATAACATCCTCAACATTCCTTGTATTTAAACTAAACAAATCAACAAACATTAACATACTC

*A. leucogrammicus*  ATAACATCCTCAACATTTCTTGTGTTCAAACTAAACAAATCAACAAACGTCAACATACTC

*C. altivelis* ATAACATCCTCAACATTTCTCGTATTTAAACTAAACAAGTCAACAAACATCAACACACTA

*E. epistictus* ATAACATCCTCAACATTCCTTGTATTTAAACTAAACAAATCTACGAGCATTAACATGCTC

*E. octofasciatus* ATAACATCCTCAACATTCCTCGTATTTAAACTAAACAAATCCACAAGCATTAATATACTC

*E. septemfasciatus* ATAACATCCTCAACATTCCTCGTATTTAAACTAAACAAATCCACAAGCATCAATATACTC

*T. dermopterus* ATAACAACCTCAACATTCCTCCTATTCAAACTAAACAAATCAACAAGCATTAACATACTC

*E. awoara* ATAACATCCTCAACATTCCTTGTATTTAAACTAAACAAGTCAACAAGCATCAACATACTA

*E. akaara* ATAACATCCTCAACATTCCTTGTATTAAAACTAAATAAATCAACAAGCATCAATATACTA

*E. trimaculatus* ATAACAACCTCTACATTCCTTGTATTTAAATAAAGCAAATCAACGAACATTAACATACTA

*E. areolatus* ATAACATCCTCAACATTCCTCGTATTTAAACTAAACAAATCAACAAGCATTAATATGCTA

*V. albimarginata* ATAACATTCTCAATATTTCTCGTCTTCAAACTTAACAAATCAACCAACATCAATATATTA

*V. louti* ATGACATTCTCGATATTCCTCGTATTTAAACTCAACAAATCAACTAGCATCAATATGCTA

*P. leopardus* ATGACATTATCTACATTCCTTGTTTTCAAACTAAACAAAGCAACAAGCATCAACATACTC

*P. areolatus* ATGACATTATCCACATTCCTTGTTTTTAAACTAAATAAAGCAACAAGCGTTAATATACTC

*E. radiosum* ATGACATTCTCGACATTTCTTGTATTTAAACTAAACAAGTCCGCGAGCATTAATATGTTG

*P. sieboldi* ATAACCCTCTCAACTTTCCTTGTATTCAAACTAAACAACTCGACCACCGTAAACGCTCTA

*E. armatus* ATAACATTCTCTACATTCCTCGTATTTAAACTAAATGATGCTACCAACATTAATACACTT

*R. oxyrhynchus* ATAACATTCTCAACATTCCTAGTATTCAAACTAAACAACGCCAAAAGTATCAACAGCCTA

*K. cinerascens* ATAACATTCTCAACATTCCTTGTATTCAAAATTAACAAATCAACCAACATCAATACACTT

*T. chatareus* ATAACAACTTCAACATTCCTCGCATTCAAACTCAACAAAGTAACAAATATTAACTCACTA

*D. berycoides* ATAACATTCTCAACATTCCTTGTCTTCAAGTTAAATAAAGCTACTAACATTAATGCACTT

*H. typus* ATGACATTCTCAACATTCCTTGTATTCAAACTAAATAAATCAACCAACATTAACGAACTC

*M. argenteus* ATGACATTCACAACTTTCCTCGTATTTAAATTAAACAAGTCAACCAACATCAATTCCCTT

*S. chuatsi* ATAACATTCTCAACATTCCTTGTATTTAAACTAAATAAATCAACTACCATTAATGCCCTC

*O. fasciatus* ATGACAAGTTCAACCTTCCTCACATTTAAACTAAATAACTCAACCAACATCAACACACTT

*P. trilineatum* ATAACATTTTCAACATTCCTTGTATTTAAACTAAACAAATCAACCAACATCAACTCTCTT

*M. salmoides* ATGACATTCTCAACATTCCTTGTATTTAAACTTAATAAATCAACCAATATTAATACCCTC

*P. tile* ATGACATTCTCAACATTCCTTGTATTTAAACTAAACAAAGCAACCAACATCAACACTCTC

*L. argentimaculatus* ATAACATTTTCAACATTCCTTGTATTTAAACTAAACAAGGCAACCAATATTAACACCCTC

*E. struhsakeri* ATAACATTCTCAACATTCCTTGTATTCAAACTAAACAAGTCAACTAACATTAATGCCCTC

*B. albus* ATGACATCCTCCACATTCCTTGCATTCAAGTTAACCAACTCTGCTACTATCAACGCAATC

*C. auripes* ATATCATCCGCAACATTCCTCGTCTTCAAACTAAACAATTCAACCAATATCAACGCACTC

*C. melampygus* ATAACTTTCTCAACATTCCTTGTATTCAAAATTAACAAAGCAACAAACGTCAATTCTTTG

*L. calcarifer* ATAACCACCTCAACATTCCTTGTCTTCAAACTAAACAAAGCAACAAACATTGCCACACTA

*S. maena* ACTGCAGCCGCAGCCTTTCTTCTATTCAAAATGTTTAAATCAACAACCATTAACCTACTC

*P. auriga* ATAACATTTTCAGCATTCCTTACATTCAAACTAAATAGCTCAACCAATATTAACTCCCTC

*C. lucidus* ATAACCTTCTCAACATTCCTTGTATTCCACCTAAACAACGCAACCAATATTAATACCCTA

*S. sihama* ATGACCTTCTCAATGTTCCTGATCTTCAAGGTCAATAAAGCCACGACCGTAAACTCATTA

*C. loricula* GTTACATACGCCACATTCACCCTCTTCACCCTTAAGAATGTTACCTCCGTAAAAGCACTT

*A. trutta* ATAACATTCTCAGCCTTCCTAATATTTAAACTTTATAAAGCAACAAGCATTAACACACTT

*H. gemma* ATGAATTGTGCTATATTTATAACCTTGTCCACCAAAAAAATTACTACTACTAACTTTCTA

*P. flavescens* ATAACATTCTCAACATTTCTTGTATTTAAGCTGAATAAATCAACTAGTATTAACATACTT

*P. macrolepida* ATAACAATCTCCACATTTCTTGTGTTTAAACTGAGTAAATCAACTAACATTAATATACTC

*S. canadensis* ATAACCTCCTCAACATTTCTTGTATTCAAACTAAATAAATCAACCAACATTAATATGCTT

*A. rogaa* GCCACCTCCTGAGCAAAAGCCCCTGCACTTACATCCCTTACACCCCTCATTCTTCTATCA

*C. argus* GCCACCTCCTGAGCAAAAACTCCCATGCTTACTGCCCTTACACCTCTCATTCTTTTATCC

*C. sonnerati* GCTACCTCCTGAGCAAAAGCCCCCGCCCTTACATCCCTCGTCCCCCTCATCCTTCTATCA

*E. fuscoguttatus* GCCACCTCCTGAGCAAAAGCCCCCGCACTAACAACCCTCACCCCCCTTGTCCTGCTCTCA

*E. coioides* GCCACCTCTTGAGCAAAAGCCCCCGCACTAACCACCCTTACCCCCCTCATTTTACTATCA

*E. bruneus* GCTACCTCCTGAACAAAAGCCCCTGCACTAACAACCCTCACTCCCCTTATTTTACTATCA

*E. moara* GCCACCTCTTGAACAAAAGCCCCTGCACTAACAACCCTCACCCCCTTAATTTTACTATCA

*E. lanceolatus*  GCCACCTCCTGAGCAAAAGCCCCCGCACTAACAACCCTCACCCCCCTCATCTTACTATCA

*A. leucogrammicus*  GCCACTTCTTGAGCAAAAGCCCCTGCACTAACAACCCTCACCCCTCTTATTCTCCTATCG

*C. altivelis* GCTACCTCCTGAGCAAAAGCCCCCGCATTAACAACTCTCACCCCCCTTATTCTACTATCA

*E. epistictus* GCCACCTCTTGAGCAAAAGCTCCAGCACTAACAGCCCTCACCCCACTTATTCTACTATCA

*E. octofasciatus* GCCACCTCTTGAGCGAAAACCCCCGCACTAACAACCCTTACCCCTCTTATTTTATTATCT

*E. septemfasciatus* GCCACCTCTTGAGCGAAAACCCCCGCACTAACAACCCTTGCCCCCCTTATTTTACTATCA

*T. dermopterus* GCCACCTCTTGAGCAAAAACCCCCGCACTGACAACCCTTACACCCCTCGTCCTACTGTCA

*E. awoara* GCTACCTCTTGAGCAAAAACCCCTGCACTAACAACCCTCACCCCTCTTATCCTCTTGTCC

*E. akaara* GCTACCTCTTGAGCAAAAGCCCCTGTACTAACAACCCTTACCCCCCTTATTCTACTGTCC

*E. trimaculatus* GCCACCTCTTGAGCAAAAGCCCCCGCATTGACAACCCTCACCCCCCTCATCCTCCTGTCC

*E. areolatus* GCCACCTCTTGAGCAAAAACCCCCGCACTAACCACACTCACCCCCCTCATTCTCCTGTCC

*V. albimarginata* GCCACCTCTTGAGCCAAAGCCCCCGCACTTACGTCCCTTACACCCCTTATTCTCCTCTCA

*V. louti* GCCACCTCTTGAGCCAAGGCCCCTGTACTCACATCCCTTACACCCCTTATTCTCCTCTCA

*P. leopardus* TCTATTTCTTGAACTAAGACCCCTACACTTACAGCCCTTGCCCCCCTTATTCTTCTATCA

*P. areolatus* GCTATCTCCTGAACTAAAACTCCAACATTAACAGCCCTAACTCCTCTCATTCTTCTGTCA

*E. radiosum* GCTACTTCTTGAGCTAAGACTCCTGCCCTTACGGCCCTCACCCCCCTTGGTCTCCTATCA

*P. sieboldi* GCAACTTCTTGGGCGAAAGCCCCAGCTTTAACCGCCCTCACTCCATTAATTCTGCTTTCG

*E. armatus* GCCACTTCTTGAACGAAAGCTCCAGCGCTTACATCTCTCGCTCCTCTCGTACTTCTTTCA

*R. oxyrhynchus* GCCGTATCTTGAGCTAAAACCCCAGTGCTCACAGCGCTGACCCCCCTAATTCTACTGTCC

*K. cinerascens* GCAACCTCTTGAGCAAAAACCCCCACACTCACCGCCCTGACTCCCCTCATCCTTCTTTCC

*T. chatareus* GCAACCTCTTGAACTAAAGCCCCCGTCATTGCATCCCTCACCCCACTTATTCTCCTTTCA

*D. berycoides* GCTACCTCCTGAGCGAAAGCCCCTGCACTTACTTCCCTAACGCCCCTTATCCTTCTGTCC

*H. typus* GCCTCCTCCTGAACAAAAGCCCCCGCACTCACTTCCCTAACACCCCTCGTTCTACTATCA

*M. argenteus* GCCACCTCCTGAGCAAAAGCCCCCGCACTCACCTCCTTAACCCCTCTAGTGCTCCTGTCC

*S. chuatsi* GCCACCTCATGAGCAAAAGCCCCCATACTCACATCCCTTGCCCCACTCATTCTCCTTTCA

*O. fasciatus* GCCACTTCGTGGGCAAAAGCCCCCGTAATCACCACCCTCGCCCCCCTCATCCTCCTCTCC

*P. trilineatum* GCTACTTCCTGGGCAAAAGCCCCCGCACTTACCTCCCTTGCCCCCCTGGTACTACTATCC

*M. salmoides* GCCATTTCTTGAGCTAAGATACCCGCACTTACTTCTCTAACTCCCCTTATCTTGCTATCA

*P. tile* GCCATCTCGTGGGCCAAGACTCCCGCACTAACTTCCCTTGCCCCCCTCGTCCTACTTTCC

*L. argentimaculatus* GCCATCTCCTGGGCTAAAGCCCCTGCACTAACTTCCTTAACCCCCCTCGTCTTACTCTCC

*E. struhsakeri* GCCGCCTCTTGAACAAAAGCTCCCGTACTTACCGCCCTCGCACCCCTCGTGCTCCTATCC

*B. albus* GCCTCCTCCTCCACAAAAACACCGGCCCTCACAGCACTTATGCCCCTTGTCCTCCTCTCA

*C. auripes* GCAACCTCCTGAGCAAAAGCCCCTATCATCACCTCCTTAACACCCCTAGTTCTATTATCC

*C. melampygus* GCAATTTCCTGAACCAAAACACCGATCATCACCTCTTTAGCCCCCTTAGTCCTTCTCTCA

*L. calcarifer* GCCATCTCATGAACTAAGACCCCCGCCCTAACATCCCTCACCCCCCTAATTCTCCTCTCA

*S. maena* GCTATCATTCGGACTAAGGGCCCAACAATCACTGCTCTTGGACCCCTGGTGCTACTCTCC

*P. auriga* GCCATAGCCTGACCAAAAGCTCCTGTTGTTACAGCCCTCGCCCCCCTCATTCTTTTATCC

*C. lucidus* GCCACCACCTGAACCAAAACCCCCGCACTCACTGCTCTTACACCCCTCCTCCTCCTCTCC

*S. sihama* GCAGTTGCATGGACAAAAATGCCTGCCCTCACCTCCCTCGCCCCCCTCGTACTACTCTCA

*C. loricula* TTTGCCCTCGGAGCAAAAAACCCCATCCTCACCACTCTACTGCCTCTCCTCCTCCTATCT

*A. trutta* GCAACCTCTTGAACGAAAGCCCCCGTCATCATAACCTTAGCCCCTCTCAGCCTTCTTTCG

*H. gemma* GCCATTTTCTGACCCTTCAGCCCCGGCCTGTCGATTACGGCATGCTTTCTCCTACTTTCC

*P. flavescens* GCCACCTCTTGAGCGAAAGCACCAGCTCTTACCGCCCTTGCTCCCTTGGTTCTTCTATCC

*P. macrolepida* GCCACATCTTGGGCCAAAGCACCTGCTTTAACAGCCCTCGCCCCATTAGTGCTCCTGTCA

*S. canadensis* GCCACATCCTGGGCAAAAGCACCAGCTCTTACAGCCCTTACCCCCTTAATTCTCCTATCC

*A. rogaa* CTAGGGGGGCTCCCCCCACTAACCGGCTTCATGCCAAAATGACTTATCCTCCAAGAACTA

*C. argus* CTAGGAGGGCTTCCTCCGCTAACAGGCTTTATGCCAAAGTGACTCATCCTTCAAGAACTT

*C. sonnerati* TTAGGAGGCCTCCCACCCCTAACAGGATTTATGCCAAAATGATTAATTCTCCAAGAACTA

*E. fuscoguttatus* CTAGGAGGCCTCCCACCACTAACAGGTTTTATGCCAAAATGACTTATCCTCCAAGAACTT

*E. coioides* CTAGGAGGCCTTCCACCATTAACAGGTTTCATGCCAAAATGACTTATCCTTCAAGAACTT

*E. bruneus* CTGGGGGGCCTTCCACCACTAACGGGCTTTATACCAAAATGACTTATTCTTCAAGAACTT

*E. moara* CTAGGGGGCCTTCCACCACTAACAGGCTTTATACCAAAATGACTTATTCTTCAAGAACTT

*E. lanceolatus*  CTAGGGGGCCTTCCACCACTTACAGGCTTTATACCAAAATGACTTATCCTCCAAGAACTT

*A. leucogrammicus*  CTGGGGGGCCTTCCACCACTAACAGGCTTCATACCAAAATGACTCATCCTCCAAGAACTT

*C. altivelis* CTAGGAGGCCTCCCACCACTAACAGGCTTCATACCAAAATGACTCATCCTCCAAGAGCTT

*E. epistictus* TTAGGAGGCCTCCCACCACTAACAGGTTTTATGCCAAAATGACTTATTCTCCAAGAACTA

*E. octofasciatus* CTGGGGGGCCTCCCACCATTAACAGGTTTCATGCCAAAATGGCTCATTCTCCAAGAATTG

*E. septemfasciatus* CTAGGAGGCCTCCCACCATTAACAGGTTTCATGCCAAAATGACTCATTCTCCAAGAATTA

*T. dermopterus* CTAGGGGGCCTCCCCCCATTAACAGGCTTTATACCAAAATGACTCATCCTTCAAGAACTG

*E. awoara* CTAGGAGGCCTTCCACCATTAACAGGCTTTATACCAAAATGACTTATCCTTCAAGAATTA

*E. akaara* CTAGGAGGCCTTCCACCATTAACAGGCTTCATGCCAAAATGACTTATTCTTCAAGAACTA

*E. trimaculatus* CTAGGAGGCCTTCCACCACTAACAGGTTTTATGCCAAAATGACTCATCCTCCAAGAACTT

*E. areolatus* CTGGGGGGCCTTCCACCATTAACAGGCTTCATACCAAAATGACTTATCCTTCAAGAACTA

*V. albimarginata* CTAGGAGGGCTTCCCCCACTAACCGGGTTTATGCCAAAATGATTAATCCTTCAAGAACTT

*V. louti* TTAGGAGGACTTCCCCCACTAACCGGATTTATGCCAAAATGATTAATCCTTCAAGAACTT

*P. leopardus* TTAGGAGGCCTCCCTCCCTTAACCGGATTTATGCCTAAATGACTTATCCTTCAAGAACTT

*P. areolatus* TTAGGAGGTCTCCCCCCTTTAACCGGATTCATACCTAAATGACTTATCCTTCAGGAACTT

*E. radiosum* TTAGGGGGGCTTCCTCCACTCACGGGCTTTATGCCGAAATGACTTATTCTTCAAGAGCTC

*P. sieboldi* CTGGGCGGACTACCCCCGCTGACCGGCTTTATACCAAAGTGGCTCATTCTTCAGGAGCTA

*E. armatus* CTTGGTGGACTTCCACCACTCACTGGCTTTATACCAAAATGACTTATTCTACAAGAACTA

*R. oxyrhynchus* CTAGGTGGCCTTCCCCCTCTCACAGGATTTATACCCAAATGACTAATCCTTCAAGAACTA

*K. cinerascens* CTAGGGGGTCTTCCACCACTCACAGGATTCATGCCAAAATGACTAATCCTCCAAGAACTG

*T. chatareus* TTAGGAGGTCTCCCTCCTTTAACAGGTTTTATGCCAAAATGACTAATCCTTCAAGAACTA

*D. berycoides* CTAGGAGGCCTCCCCCCTTTAACCGGCTTCATACCAAAATGACTAATTCTTCAAGAGCTA

*H. typus* CTGGGAGGTCTCCCCCCACTAACCGGGTTCATACCAAAATGGCTAATTCTTCAAGAACTA

*M. argenteus* CTAGGCGGTCTCCCCCCATTAACCGGCTTCATGCCAAAATGATTAATTCTTGCAGAACTA

*S. chuatsi* CTAGGCGGTCTACCCCCACTAACAGGATTTATACCAAAATGACTAATCCTCCAAGAACTA

*O. fasciatus* CTAGGAGGACTGCCCCCACTTACCGGCTTCGTACCCAAATGACTAATCCTCCAAGAACTA

*P. trilineatum* TTAGGGGGCCTTCCCCCGCTGACCGGCTTCATGCCAAAATGACTTATTCTTCAAGAACTA

*M. salmoides* TTAGGAGGCCTCCCCCCACTGACAGGCTTTATGCCAAAATGACTGATTCTGCAAGAACTC

*P. tile* TTAGGAGGCCTTCCCCCACTGACCGGCTTCATACCAAAATGACTAATCCTTCAAGAACTT

*L. argentimaculatus* CTAGGCGGCCTCCCCCCACTAACAGGCTTCATGCCAAAATGACTCATCCTGCAAGAGGTA

*E. struhsakeri* CTAGGCGGCCTCCCCCCTCTGACGGGCTTTATGCCAAAGTGACTTATTCTGCAAGAATTG

*B. albus* TTAGGAGGCCTCCCCCCACTAACCGGATTTATACCTAAATGACTCATCCTTCACGAACTA

*C. auripes* CTAGGGGGCCTTCCACCCCTTACTGGCTTCATGCCAAAGTGACTAATCCTACAAGAACTA

*C. melampygus* TTAGGAGGCCTTCCCCCACTAACAGGCTTCATACCAAAATGACTAATTCTCCAAGAACTC

*L. calcarifer* CTCGGAGGCCTCCCCCCACTAACCGGATTTATGCCCAAATGACTTATCCTTCAAGAACTT

*S. maena* CTAGCCGGCCTCCCCCCCTTAACTGGCTTTTTAGCAAAATGATTCATCCTTCACGAACTA

*P. auriga* CTTGGAGGTCTGCCCCCCCTCACCGGTTTTATGTCAAAATGACTTATCCTTCAAGAATTA

*C. lucidus* CTAGGAGGCCTCCCCCCACTTTCAGGCTTTATGCCAAAATGACTAATCCTCCAAGAACTA

*S. sihama* CTAGGAGGGTTACCACCCCTCACGGGTTTCATGCCTAAATGACTTATCCTGCAAGAGCTC

*C. loricula* TTAGCTAGCCTCCCCCCGCTAACCGGCTTCCTAGCGAAACTACTCATCCTGAAAGAACTG

*A. trutta* CTCGGCGGGCTCCCCCCATTTACCGGGTTCATGCCAAAATGACTCATCCTACAAGAACTA

*H. gemma* CTCGCCGGCCTGCCCCCTCTTACAGGCTTTATGTCCAAGTGATTAATCCTACAAGAGCTA

*P. flavescens* CTAGGAGGCCTTCCACCATTAACCGGCTTTATACCAAAATGACTTATTCTTCAGGAACTA

*P. macrolepida* CTAGGCGGCCTCCCCCCCTTAACAGGCTTTATACCCAAGTGACTCATTCTTCAAGAACTC

*S. canadensis* CTCGGAGGACTTCCCCCTCTAACTGGCTTTATGCCAAAATGGCTTATTCTTCAAGAACTA

*A. rogaa* ACCAAACAAGACCTAGCACCCGCCGCCACACTAGCCGCAATAACAGCCCTACTAAGTTTA

*C. argus* ACTAAGCAAGATTTAACACTCATTGCCACATTAGCCGCAATAACAGCCCTCTTAAGCCTA

*C. sonnerati* ACCAAACAAGACCTAGCACCAGCTGCCACGCTGGCCGCACTAACCGCCCTATTAAGCCTC

*E. fuscoguttatus* ACCAAACAAGAACTAGCACCCATCGCTACACTAACCGCACTAACCGCCCTTCTAAGCTTA

*E. coioides* ACCAAACAAGAACTAGCACCTATCGCTACACTAACTGCACTAACTGCCCTATTAAGCCTG

*E. bruneus* ACCAAACAAGAACTAGCACCCATCGCCACACTAACCGCACTAACCGCCCTACTTAGCCTA

*E. moara* ACCAAACAGGAACTAGCACCCATCGCCACACTAACCGCACTAACCGCCCTACTCAGCCTA

*E. lanceolatus*  ACCAAACAAGAACTAGCACCTATCGCCACACTAGCTGCACTAACCGCCCTACTAAGCCTG

*A. leucogrammicus*  ACCAAACAAGAACTAGCCCCCATCGCTACGCTAACCGCACTAACCGCTCTACTAAGCTTG

*C. altivelis* ACTAAACAAGATCTAGCACCCATCGCTACACTAACCGCGCTGACAGCCCTACTAAGCTTG

*E. epistictus* ACCAAACAAGATTTAACCCCAATCGCCACACTAGCTGCACTAACCGCACTATTAAGTCTC

*E. octofasciatus* ACTAAACAAGCCCTCGCACCCATTGCTACCCTAGCTGCACTAACTGCTCTACTAAGCCTA

*E. septemfasciatus* ACTAAACAAGCCCTCGCACCCATTGCCACACTAGCAGCACTAACTGCTCTACTAAGCCTA

*T. dermopterus* ACTAAGCAAGACCTCGCGCCCGTCGCTACACTAGCCGCATTAACCGCCCTACTGAGCCTA

*E. awoara* ACCAAACAAGACCTAGCACCCATTGCCACCCTAGCCGCACTCACCGCCCTATTAAGCCTG

*E. akaara* ACCAAACAAAATCTAGCGCCTATTGCCACCCTAGCCGCACTCACCGCCCTATTAAGCCTG

*E. trimaculatus* ACCAAACAAGACTTAGCACCCGTCGCCACGCTTGCCGCACTTACCGCCCTACTAAGCCTA

*E. areolatus* ACCAAACAAGACCTAGCACCCATTGCCACACTAGCAGCTCTCACCGCCCTATTAAGCCTG

*V. albimarginata* ACTAAACAAGACCTTGCACTTACCGCCACACTAGCTGCACTGACCGCCCTCCTAAGCTTG

*V. louti* ACTAAACAGGACCTCGCACCTACCGCCACACTAGCGGCACTAACCGCCCTCCTAAGCCTA

*P. leopardus* GCCAAACAAGATCTCGCCCTCACCGCCACCCTAGCAGCAATTACCGCGCTGCTCAGCCTA

*P. areolatus* ACTAAACAAGACCTAGCCCTCACCGCTACCCTAGCAGCAATCACCGCACTGCTCAGCCTA

*E. radiosum* ACCAAGCAAGATCTTGCTCCAACGGCAACCCTTGCCGCAATATCAGCCCTCCTTAGCCTT

*P. sieboldi* ACCAAGCAAGACCTTGCACTTACAGCCACCGTCGCCGCCCTTAGCGCCCTCCTAAGCCTA

*E. armatus* GCTAAACAAGATCTTGCCCCCACTGCAACCCTGGCTGCACTTACTGCTCTTTTAAGTCTC

*R. oxyrhynchus* ACAAAACAAGGACTAGCCCCCACAGCAACCCTCGCCGCCCTATCCGCACTCCTTAGCCTC

*K. cinerascens* ACAAAACAAGACCTCGCACCCGCCGCCACACTAGCTGCACTAACCGCCCTCCTCAGCCTG

*T. chatareus* ACTAAACAAGATCTCGGTACATTGGCCACATTAACTGCACTAACTGCTCTACTCAGCTTG

*D. berycoides* GCCAAACAAGACCTCGCCCCAACAGCCACACTAGCTGCCTTAACCGCCCTCCTCAGCTTA

*H. typus* ACCAAACAAGACCTCGCCCCCACAGCTACTTTAGCTGCCCTAACTGCCCTTCTCAGCCTA

*M. argenteus* ACTAAACAAGACCTCGCCCCTACAGCCACACTAGCGGCACTAACCGCCCTTCTCAGCCTA

*S. chuatsi* ACCAAACAAGGCCTCGCCCCTACAGCCACACTAGCTGCCCTAACTGCCCTCCTAAGCCTA

*O. fasciatus* ACAAAACAAGACCTCGCACCCACTGCCACCCTAGTGGCCCTAACCGCCCTACTAGGTTTA

*P. trilineatum* ACTAAACAAGACCTTGCTCCTACCGCCACTTTAGCTGCACTTACCGCCCTTCTAAGCTTA

*M. salmoides* ACTAAACAGGAACTCCCCACCACAGCAACTTTAGCAGCACTTACCGCCCTTCTCAGCCTT

*P. tile* ACTAAGCAGGACCTGGCACCAACAGCCACACTAGCCGCACTAACAGCCCTTCTAAGTCTA

*L. argentimaculatus* ACCAAACAAGACCTAGCCCCCACAGCCACACTAGCCGCACTAACAGCCCTCCTAAGCCTA

*E. struhsakeri* GCTAAACAAGACCTTGCCCCGGCAGCCACATTAGCCGCACTAACTGCCCTCCTTAGCCTC

*B. albus* ACCAAACAAGACCTCGTCCCTATTGCCACCTTTGCTGCCCTTACTGCTCTCCTCAGTCTT

*C. auripes* ACTAAACAAAGCCTTACCCCCATAGCCACGCTAGCCGCACTAGCCGCTTTACTCAGCTTA

*C. melampygus* ACAAAGCAAGATCTACCCCTCTTAGCCACAATAGCCGCGCTAACAGCCCTTCTAAGCCTA

*L. calcarifer* ACCAAACAAGACCTATCAACACTAGCGACACTAACCGCACTCACTGCCCTCCTCAGCCTA

*S. maena* ACTAAACAAGATCTCTCATCTTTAGCCACACTCGCAGGTGTCTCCGCACTCCTAAGTCTC

*P. auriga* ACTAAACAAGGACTCTTAGCCCTCGCAACACTAGCAGCCCTTTCAGCCCTTCTAAGCCTC

*C. lucidus* ACTAAGCAAGACCTAGCTTTGACCGCCACTCTAGCTGCACTCACCGCACTCCTCAGCCTT

*S. sihama* GCGAGCCAGGACCTCCCTGCAATCGCCACCATCGCAGCCCTCTCGGCCTTACTAAGCCTG

*C. loricula* ACCAAACAAGGCCTCACCCTAGCGGCCGCCTTAGCCATTATAGGCACCCTCCTCAGCGCA

*A. trutta* ACTAAACAGGGGCTAGCCGCTACTGCCACCTTTGCAGCCCTTACTGCACTCCTTAGCCTC

*H. gemma* GCAAAACAAGATTTAGGAGGGCTCGCTACAGTTGCAGCCCTAACTGCACTCCTAAGCCTT

*P. flavescens* GCCAAGCAAGACTTAGCCCCCACAACAACCGTAGCTGCAATATCAGCTCTTCTGAGCCTT

*P. macrolepida* GCCAAACAGGACCTAGCCCTAACAGCAACCCTGGCTGCAATAACAGCCCTCCTTAGTCTC

*S. canadensis* GCCAAACAAGACCTCGCCCCCACAGCAACCCTAGCCGCAATATCAGCCCTTTTAAGCCTT

*A. rogaa* TACTTCTACTTACGACTCACCTACGCGATAACACTCACAATATCCCCCAACAACCTAACA

*C. argus* TACTTCTATCTCCGGTTAACCTACGCAATAACATTAACAATGTCCCCCAACAACTTAACA

*C. sonnerati* TACTTCTACCTGCGATTGACCTACGCAATAACACTTACAATGTCTCCAAACAACGTAACA

*E. fuscoguttatus* TACTTTTACCTACGGCTAACATACGCCATAACACTCACCATGTCCCCTAACAACGTAACA

*E. coioides* TACTTTTACTTACGACTAACATATGCCATAACACTCACCATATCTCCTAATAACGTAACA

*E. bruneus* TATTTCTACTTACGATTAACATACGCTATGACACTCACCATGTCCCCTAATAACGTGACA

*E. moara* TATTTCTACTTACGATTAACATACGCTATAACACTCACCATATCCCCTAATAACGTAACA

*E. lanceolatus*  TACTTCTACTTACGACTAACATACGCCATAACACTTACCATATCTCCCAACAACATCACA

*A. leucogrammicus*  TACTTCTACCTACGATTAACATACGCCGCAACACTTACCATGTCCCCTAATAATATTACA

*C. altivelis* TACTTCTACTTACGACTAACCTATGCCATAACACTTACTATGTCTCCTAACAACGTAACA

*E. epistictus* TACTTCTACCTACGATTAACATACGCCATAACACTCACCGTCTCCCCTAACAACGTAACA

*E. octofasciatus* TATTTTTATTTACGATTAATATATGCTATAACACTCACCATGTCCCCCAATAACGTAACA

*E. septemfasciatus* TATTTCTATTTACGATTAACATACGCTATAACACTCACCATATCCCCTAATAACGTAACA

*T. dermopterus* TATTTTTACTTACGCCTAACATATGCTATAACACTCACCATATCCCCCAACAACGTAACA

*E. awoara* TACTTCTACTTACGATTAACATATGCTATAACACTCACCATATCGCCTAATAACGTAACA

*E. akaara* TACTTTTATTTACGGCTAACATATGCTATAACACTTACCATGTCCCCTAATAACGTAACA

*E. trimaculatus* TATTTTTACCTACGACTGACATACGCCATGACACTTACCATGTCCCCCAACAACATCACA

*E. areolatus* TATTTTTACCTACGATTATCATACGCCATGACACTCACCATATCCCCCAATAACGTAGCA

*V. albimarginata* TACTTCTACTTACGACTCACCTATGCAATAGCCCTCACCATGTCCCCTAATAACTTAACC

*V. louti* TACTTCTACTTACGACTTACATATGCAATAACCCTTACCATATCTCCCAATAACTTAACT

*P. leopardus* TACTTCTACCTTCGGCTTACATACGCAATAACCCTAACAATGGCCCCCAACAACCTAGCA

*P. areolatus* TACTTCTACCTTCGGCTTACATACGCAATAACCCTAACAATAGCTCCTAACAACTTAACA

*E. radiosum* TACTTTTATTTACGCCTTTCCTATGCAATAGCCCTGACTATGTCCCCTAACAATATTGCA

*P. sieboldi* TACTTCTACCTGCGATTGTCCTACGCCATGACCCTTACTATGTCCCCAAACTCCATCGCT

*E. armatus* TACTTCTACCTACGACTCTCGTACGCTATAGCTCTAACCATCTCCCCTAACAACTTATTG

*R. oxyrhynchus* TTCTTCTACCTACGACTCTCCTACGCTGCAACACTAACAATTTCCCCTAATAACCTCGCC

*K. cinerascens* TACTTTTACCTCCGACTCTCATACGCAATTACACTTACTATATCCCCCAATAACCTTACA

*T. chatareus* TATTTCTACCTCCGCCTATCATACGCAATGACCCTAACCGTCTCCCCAAGTAACCTAACC

*D. berycoides* TATTTTTACCTACGGCTCTCGTACGCAATAACACTAACCATCTCCCCAAACAACCAAACC

*H. typus* TACTTCTACCTCCGACTCTCATACGCAATAACACTAACCATAGCCCCAAACAATTTAACC

*M. argenteus* TACTTTTACCTACGACTTTCTTACGCCCTAACCCTAACCATCTCCCCTAATAACCTAGTT

*S. chuatsi* TATTTTTACCTACGCCTCACATACGCAATAACCCTTACCATATCCCCCAACAATCTCACC

*O. fasciatus* TACTTCTACCTCCGACTCTCCTACGCAATGACACTTACTATGTCCCCCAACAACCTAGCC

*P. trilineatum* TATTTTTACCTACGACTCTCATATGCTATAACCTTAACAATATCGCCTAACAATCTAACT

*M. salmoides* TACTTCTACCTACGACTCTCCTACGCCATAACATTGACTATGTCCCCCAATAACCTTGCT

*P. tile* TACTTCTACCTTCGCCTTACATACGCGATGACTCTAACTATTTCCCCAAACAGCCTCTCC

*L. argentimaculatus* TACTTTTACCTCCGCCTCACATACGCAATAACCCTTACCATTTCTCCCAACAGCCTATCT

*E. struhsakeri* TACTTCTACCTACGGCTTTCATACGCAATAACCCTAACTATATCCCCTAATAATCTGGCC

*B. albus* TACTTCTATCTTCGCCTTTCCTACGCAATAACCCTCACTATGTCCCCAAACAACTTAACA

*C. auripes* TACTTCTATCTACGACTCTCATACGCAATAACCCTGACCGCCTCTCCAAACACCTTAACA

*C. melampygus* TACTTTTACCTACGCCTTTCTTACGCAATAACCCTAACTATATTTCCCAACAACCTAGCA

*L. calcarifer* TTCTTTTATCTACGCCTATCTTATGCAATAGCCCTTACCCTGTCCCCAAATAATGCCACC

*S. maena* TTTTTTTATATACGCCTTGCATTTGCAATAATCCTCACTAACCCCCCAGACAACTTTACT

*P. auriga* TATTTTTATCTCCGCTTAGCATACGCCCTCACTCTAACCCTATCCCCCAATACCCTGACT

*C. lucidus* TACTTCTACCTGCGTTTGTCATACGCCCTTGCCCTAACAATATTCCCTAACAATCCATCA

*S. sihama* TATTTTTACCTGCGCCTCTCGTACGCTATGACTCTAACCATGTCCCCTAACAATATTTCG

*C. loricula* TTCTTTTACGTACGAATCTCCGTAGCAACAGCCCTCACCCTCGCTCCCAATGTCATAACA

*A. trutta* TACTTCTACCTACGACTATCCTATGCAATAACCCTCACCGTATCACCTAACACCCTTACG

*H. gemma* TTTTATTATCTTCGTCTCTCATACGCCGTTGCCCTTACTATCTCCCCTAATAATTTAACA

*P. flavescens* TATTTTTACCTCCGTCTTTCCTATGCAATAACTTTAACCATGTCCCCCAATAACTTAACA

*P. macrolepida* TACTTCTATCTACGAATTTCATATGCCATAACCCTAACTATGTCCCCTAACACCCTGACA

*S. canadensis* TATTTTTACCTACGACTCTCCTACGCAATAGCTTTGACAATGTCCCCCAATAATTTAACA

*A. rogaa* GGAACCACCCCTTGACGTCTCCCATCCTTCCAATTTACACTTCCCCTCGCAACCCTGACT

*C. argus* GGAATTACCCCCTGACGCCTTCCATCTTCCCAATTAACACTTCCTCTCGCCACCTCAACT

*C. sonnerati* GGATTAGCCCCTTGACGTCTCCCATCCTCCCAACTCACACTTCCACTCGCCACCACAACA

*E. fuscoguttatus* GGCACAACCCCATGACGCCTTCCATCCCTACAACTGACACTCCCACTTGCCATCCTAACC

*E. coioides* GGCACTGCCCCATGACGTCTCCCATCCCTACAACTAACACTTCCACTTGCCACCCTAACT

*E. bruneus* GGCACAGCCCCCTGACGCCTCCCATCCCTACAACTAACACTCCCCCTTGCTACCTTAACC

*E. moara* GGTACAACCCCCTGACGCCTCCCATCCCTACAACTAACACTCCCCCTTGCTACCTTAACC

*E. lanceolatus*  GGCACAACCCCATGACGCCTCTCATCCCTACAACTAACACTCCTACTTGCCACCCTAACC

*A. leucogrammicus*  GGTGCAACCCCATGACGTTTATCATCCATCCAATTAACACTCCCCCTTTCTGCTCTAACC

*C. altivelis* GGCACAGCTCCATGACGCCTCCCATCCCTGCAACTAGCACTCCCACTTGCCACCCTGACC

*E. epistictus* GGTACAGCCCCCTGACGCCTCCCAACCTCACAATTAACACTCCCGCTTACTACCCTAACC

*E. octofasciatus* GGCACAGCCCCATGACGCCTTCCGTCCCTACAACTAACACTCCCACTCGCTACTTTAACC

*E. septemfasciatus* GGTACAGCCCCATGACGCCTCCCGTCCCTACAACTAACACTCCCCCTCGCTACTTTAACC

*T. dermopterus* GGCACAACCCCGTGACGCCTCCCATCCTTACAACTCACCCTCCCTCTCGCTATTTTAACC

*E. awoara* GGTTTAGCCCCATGACGCCTGCATTCCTCCCAACTAACGCTTCCTCTTGCCACCTTGACT

*E. akaara* GGTACAGCCCCGTGACGCCTTCATTCCTCACAACTAACACTCCCACTTGCCACTTTAACC

*E. trimaculatus* GGTACAACCCCCTGACGCCTCCCCTCTTCACAGTTTACACTTCTACTCGCCATTATAACT

*E. areolatus* GGCCTAACCCCCTGACGTCTCCACTCCCCACAACTAACACTCCCACTCGCCATTCTAACC

*V. albimarginata* GGCACCACCCCTTGACGCCTCCAATCACTTCAACCCACACTACCCCTTGCTATTTCAACA

*V. louti* GGCACTACACCTTGACGCCTCCAGTCACTGCAGCCCACACTACCCCTTGCTATCTCAACA

*P. leopardus* GGTCTCACCCCATGACGACTACAAAACACACAAACCACCCTACTCCCAGCTCTATCTACC

*P. areolatus* GGTCTCACCCCCTGACGACTACAAAGTGGACAGACCACCCTACTCCCAGCCTTATCCACC

*E. radiosum* GGTACAACTCCTTGACGGCTTCAACACGCACAGTTTACTCTACCCTTGGCCATCACGGCT

*P. sieboldi* GGCACCACGCCTTGACGGCTTCCGTCTACTCGACTGACGTTACCCCTTGCCATCACCACT

*E. armatus* GCTACTACCCCCTGACGCCTTCCCTCCTCGCAACTTACACTGCCCTTGGCCGTCTCAACC

*R. oxyrhynchus* AGCATCCCGACCTGACGCCTCCCCTCAACCCAATCCACCCTCCCCCTTGCCTTCTCTACA

*K. cinerascens* GGAACAACCCCATGACGACTCCCATCAACACAACTCTCTATACCTCTTGCCTTCTCGACC

*T. chatareus* GGGACCACCTCCTGACGATTCTACCCCTCCCAACCCACTATACCCTTAGCCATTGCAACC

*D. berycoides* GGGACCACCCCCTGACGCCTTCCCTCCTCACAACTCACCCTCCCCCTAGCCATCTCTACT

*H. typus* GGGACTGCCCCGTGGCGCCTCCCCTCGTCACAGCTCACATTACCCCTCTCCACCTCCACC

*M. argenteus* GGGACGACCCCTTGACGACTGCCCTCCACCCAACTCACTCTGCCACTTGCAATCTCTACC

*S. chuatsi* GGAACTACCCCCTGACGCCTCCCCTCCACACAACTAACCCTACCTCTAGCCGTCTCAACT

*O. fasciatus* GGAACCACCCCATGACGCCTCCGCCCCTCGCAATTCACAATACCTATTGCCCTTTCAACC

*P. trilineatum* GGAACAACCCCATGACGGCTCACCTCCCCCCAACTCACTTTACCACTAGCCATTTCAACC

*M. salmoides* GGTGTTACACCCTGACGTCTCCCATCTGCACAACTCACGCTTCCTCTCGCTGTTACAACC

*P. tile* GGGACAACCCCCTGACGACTGCCAACAACACAACTAACCCTCCCACTTGCCGTCTCTACC

*L. argentimaculatus* GGGACAACCCCTTGACGTCTCCCAACAACACAACTAACACTTCCCCTCGCCATTTCTGCT

*E. struhsakeri* GGCACTACCCCCTGACGACTCCCCTCGTCTCAACTCACGCTGCCCGTCGCTGTTGCCACC

*B. albus* GGCGCCTCTTTCTGACGGCTGCAGACCCCCCAACCCACACTACCCTTGGCTGTAACCACC

*C. auripes* GGCACTACTCCATGACGACTTACCTCCTCACAGCTAACCCTGCCCCTTGCCATCTCCACC

*C. melampygus* GGAACTACACCATGACGCTTCCACACCCCCCAGCTAAACCTCCCACTAGCCATTTCAACC

*L. calcarifer* GGAACCACTCCCTGACGACTCTCATCCACCCAATCTAACCTGCCACTAGCCCTCTCAACT

*S. maena* GGTATGATCGTCTGACGCAATTCAACATTCAACATAACCCTCTGCCTCTCTTTCACCACC

*P. auriga* GGGACAGCCCCATGGCGTCACCCCACATCCCACCTAACATTATTCCTATCGACCACTACC

*C. lucidus* GGTACTTCCCCTTGACGCCTCCAATCACCCCAAATCATATACCCCCTCGCCCTTTCAATT

*S. sihama* GGTGCTGCCCCCTGACGCCTCCCCCAAACCCAGCTTACCCTCCCGCTTGCCGCATCCTCC

*C. loricula* AGCACAGCCCCCTGGCGACTTATAACCTCCCGACTCACTATGCCGATCTCCATCAGCACT

*A. trutta* GGTACAACTCCTTGGCGATTCTACTCCCCCCAGTCCACTCTCCCACTATCAATGACTGTT

*H. gemma* GGTACCGCCCCTTGGCGCCTCCACTCGGCTAACCCCTCCCTCCCCTTATCCATTTTTACT

*P. flavescens* GGAACAACACCTTGACGACTACAATCCTCACAACTCACACTCCCCCTAGCCGTCTCAACC

*P. macrolepida* GGCACTACCCCTTGACGACTGCAACATTCACAGTTTACGCTTCCCCTAGCAATAACAACC

*S. canadensis* GGAATAACCCCATGGCGGCTTCAATTCTCACAACTAACGCTTCCACTAGCTATTTCAACT

*A. rogaa* ATAGCAACAATCTCCCTCCTTCCCCTTACTCCCGCCGCAACAGCACTCCTAACCTTA---

*C. argus* ATGGCAACAATTTCTCTTCTCCCTCTCGCCCCCGCTGCAACAGCACTCCTTACCTTG---

*C. sonnerati* ATAGCAACCATTGCCCTCCTTCCCCTCACCCCTACCATAATTGCACTACTTACATTA---

*E. fuscoguttatus* ACGGCAACAATCTCGCTACTCCCACTTACCCCTACTATCTCAGCACTACTAATTCCA---

*E. coioides* ACAATAACAATCTCCCTACTCCCACTTACCCCCACTATTATGACACTGTCCATTCTA---

*E. bruneus* ACAGCAACAATCTGCCTACTTCCACTTACTCCCACTATAACAGCACTATTCAACCTA---

*E. moara* ACAGCAACAATCTGCCTACTCCCACTTACCCCTACTATAGCGGCACTATTCAACCTA---

*E. lanceolatus*  ATAGCAACAATTTCTCTACTCCCACTTACCCCTGCTATTACAATACTATTTACCCCATCC

*A. leucogrammicus*  ATAATAACTATCTCCCTACTCCCACTCACCCCTACCATAACAACACTATTCACCCTA---

*C. altivelis* ATAACAACAATCTCTCTGCTCCCGCTCACCCCCACTGTAACAACACTATTTATACTA---

*E. epistictus* ATGGCAACAATCTCCTTACTCCCTCTCACCCCTACCATGCTAGCACTATTAACCCTA---

*E. octofasciatus* CTAGCAACAATCTCCTTACTTCCACTTGCTCCCACCATACTTGCACTACTAACCCTA---

*E. septemfasciatus* CTAGCAACAATCTCCTTACTTCCGCTTGCTCCCACCATGCTTGCACTACTAACACTG---

*T. dermopterus* GTGGCAACAATCTCCCTACTCCCACTTGCCCCCACCATTCTCGCTGTACTAACCCTG---

*E. awoara* GTAGCAACAATCCTTCTTCTTCCACTAACCCCAACAATACTAACTCTACTAACCCTA---

*E. akaara* ACAGCAACTATCCTCCTACTCCCACTAACCCCAACAATACTAACTCTATTGACCGTA---

*E. trimaculatus* ATACTTACAATCTCCCTACTACCCTTCACCCCAACCATATCAACTCTATTAACCCTA---

*E. areolatus* ATATCAACAATCTCCTTGCTCCCCCTCACCCCAACCATCTTAACCCTTCTAACCCTG---

*V. albimarginata* GTAGCTACCCTCTCCCTGCTACCCCTAACCCCTGCAATAATAGCCCTACTAACTCTT---

*V. louti* ATAACCACCCTCTCCCTGCTACCCCTAACCCCTGCAGTAGTAGCCCTACTAACTCTT---

*P. leopardus* TCAGCAACACTTCTCCTTCTTCCACTTACACCAGCAACAATTTCACTACTTTCCCTC---

*P. areolatus* TCAATAACACTTCTCCTTCTTCCACTAACCCCAGCAACACTTTCAATACTCTCCCTC---

*E. radiosum* ACCGCTACCCTTCTGCTTCTTCCACTAGCCCCTGCAGTCGCGGCACTACTGGTCCTT---

*P. sieboldi* ACCTCGGCCATGCTCCTTCTGCCCATTACCCCCGCCACCCTAGCACTCCTCTCGCTG---

*E. armatus* GTTGCCACACTTTCACTATTACCCCTAACCCCTGCCGCACTAGCACTCTTAACTCTT---

*R. oxyrhynchus* ATAGCCACTATTACACTCCTTCCCCTCACCCCTGCTGCAGCCACACTATTAGCCCTC---

*K. cinerascens* ATAGCAACCCTCCTGCTCCTACCCCTAACCCCTGCCACAGTTGCATTGCTGGCCCTT---

*T. chatareus* ATAGCATCAATAACCCTTCTCCCCCTAACCCCCGCAACAGTTGCCCTCCTAACCCCC---

*D. berycoides* ATGGCAACTCTTTCCCTACTTCCCCTCACTCCCGCCACAGTGGCACTTCTGACCCTC---

*H. typus* ACGGCCACCCTGCTTCTCCTACCCCTCGCCCCCGCCGCAACAGCACTCCTGGCCCTC---

*M. argenteus* ATAGCCACCATCTCCCTTCTCCCCCTTACCCCTGCTATAATCGCACTATTAACCCTT---

*S. chuatsi* ACAGCCACCTTATCCTTATTACCCCTCACCCCAGCAGCAGTTGCCCTATTAACCCTC---

*O. fasciatus* ATAACCACCCTATCACTACTACCCCTAACCCCAGCCATAATTGCACTGCTCACCCAG---

*P. trilineatum* ATGGCTACCATCCTACTTCTACCCGTCACCCCTGCAACAATCGCATTGTTGACCCTT---

*M. salmoides* ACAATAACCCTGGCACTCCTCCCCCTTACCCCAGCCATTACTGCAACACTCACTCTT---

*P. tile* ATAGCAACCCTCTCTCTCCTCCCTCTTACCCCCGCTGTAATAGCATTACTAACCCTC---

*L. argentimaculatus* ACCGCCACGGTCGCCCTCCTCCCCCTCACCCCTGCTATAATAGCCCTTCTAACCCTC---

*E. struhsakeri* ATGGCCGCACTCTCCCTCCTACCCCTTACTCCAGCCACGGTTGCACTACTAACCCTC---

*B. albus* ATATCCACCATCATGTTACTGCCTCTAACCCCAACCGCAGTTGCTTTATTGAGCCCC---

*C. auripes* GTAGCCACTATCTCCCTCCTCCCACTAACACCCGCTATAGTGGCCCTACTAACCCTT---

*C. melampygus* TCAGCTACAATTCTCCTCCTCCCCCTCACCCCCGCCATTACAGCCCTCCTCACCCTG---

*L. calcarifer* ACCACCTCAATCCTTCTCCTCCCCCTCGCCCCCGCCGCAATAGCCCTTTTCACCCCC---

*S. maena* GTTGGCTCCCTCATCCTTATCCCCCTCGCCCCAGCAATCGCAGTTATACTGTCGATA---

*P. auriga* GTCGCCGCAGTATTCTTCCTTCCCCTAACCCCAGCAATTGTAATACTCCTATCCCTA---

*C. lucidus* ACAGCTGCCCTCACCCTTCTTCCCCTAACCCCAACCGTCACAGCACTAATAATGCCC---

*S. sihama* CTGGCCTCCCTTTCGCTCCTCCCTTTAACCCCTGCAGCCCTCGCCCTCTTAAAATTT---

*C. loricula* GTGCTAGCGGTTGCTCTCCTTCCCCTCTCCCCCACCCTGCTTGCGCTACTCACATAC---

*A. trutta* GTAGCAGCCCTCTCCCTCTTGCCACTCACTCCTGCTGTGGCTGCCCTTCTAACTCCT---

*H. gemma* ACAATAGGCCTCATACTCCTCCCCATCACCCCTAGCCTATTCTCGCTATTCATAATC---

*P. flavescens* CTGGCCACTCTCCTTCTTCTTCCCCTAACCCCTGCAACAGTTGCTCTAATCACCCTC---

*P. macrolepida* ACCGCCACCCTCCTGCTCCTGCCACTAACCCCTGCAGTCGTTGCACTTCTCACCCTC---

*S. canadensis* ATAGCCACCCTTTTACTTCTTCCCCTAACCCCCGCAGCCGTCGCACTGCTTACCCTT---

*A. rogaa* TAAATGCCCCAACTCCTCCCAACACCATGATTTGGAACACTACTCTTTGCCTGGGTAGTC

*C. argus* TAAATGCCCCAGCTCCTTCCAATACCATGATTCGGAACACTACTCTTTGCCTGAGTAGTC

*C. sonnerati* TAAATGCCTCAGCTCCTTCCAATACCCTGATTTGGTACGCTGCTCTTTGCTTGAGTAGTA

*E. fuscoguttatus* TAGATGCCCCAACTCCTCCCACTACCATGATTTGGTACACTACTCTTTGCCTGAATGATT

*E. coioides* TAAATGCCCCAACTCCTCCCACTACCCTGATTTGGTACACTACTCTTCGCTTGAGCAGTA

*E. bruneus* TAGATGCCCCAACTCCTCCCACTACCCTGATTCGGTACACTACTCTTTGCCTGAGTAATC

*E. moara* TAGATGCCCCAACTCCTCCCACTACCCTGATTCGGCACACTACTCTTTGCCTGAGTAGTT

*E. lanceolatus*  TAAATGCCCCAACTCCTCCCACTACCCTGATTCAGTACACTACTCCTTGCCTGAGTAATT

*A. leucogrammicus*  TAAATGCCCCAACTCCTCCCCCTACCCTGATTCGGTACACTACTCTTTGCCTGAGTAGTT

*C. altivelis* TAAATGCCCCAACTCCTCCCACAACCTTGATTCGGCACACTACTCTTTGCCTGAGTAGTT

*E. epistictus* TAAATGCCCCAACTCCTCCCACTACCCTGATTCGGCACACTACTCTTTGCTTGAGTGGTT

*E. octofasciatus* TAAATGCCCCAGCTTCTCCCACTCCCCTGATTCGGTACACTACTCTTTGCCTGAGTAGTT

*E. septemfasciatus* TAAATGCCCCAGCTTCTCCCACTCCCCTGATTCGGTACACTACTCTTTGCCTGAGTAGTT

*T. dermopterus* TAAATGCCCCAACTCGCCCCACTGCCTTGATTTGGTACGCTGCTTTCTGCCTGAGTGGTC

*E. awoara* TAAATGCCCCAACTCCTCCCACAACCCTGATTTGGTACACTACTCTTTGCCTGAGTGGTC

*E. akaara* TAAATGCCCCAACTCCTCCCACAACCCTGATTTGGCACACTACTCTTTGCCTGAGTGGTC

*E. trimaculatus* TAAATGCCCCAACTCCTCCCATTACCCTGATTTGGCACACTGCTCTTTGCTTGAGTAGTT

*E. areolatus* TAAATGCCCCAACTCCTCCCACTACCCTGATTCGGAACACTACTCTTTGCCTGAGCAGTA

*V. albimarginata* TAGATGCCCCAGCTCCTTCCAACGCCCTGGTTTCCCACTCTACTCTTCGCTTGAGTAGTC

*V. louti* TAGATGCCCCAGCTCCTTCCAACGCCCTGGTTTCCCACTCTACTCTTCGCTTGAGTAGTC

*P. leopardus* TAAATGCCTCAACTGCTCCCCACCCCTTGATTTATAACCTTAATTTTCGCTTGAGTGGTG

*P. areolatus* TAAATGCCCCAACTGCTCCCAACCCCTTGATTTATAACATTAGTTTTTGCTTGAGCAGTA

*E. radiosum* TAAATGCCTCAACTCAACCCCACACCTTGATTTGCTATTTTAGTTTTTACCTGACTAATT

*P. sieboldi* T--ATGCCTCAACTCAATCCCTCCCCCTGGTTTGCCATCCTTCTCTTCTCATGACTCGTC

*E. armatus* TA-ATGCCCCAACTTAACCCCACACCTTGATTTGCTACTCTAATTTTTTCTTGACTTATT

*R. oxyrhynchus* TA-ATGCCCCAGCTCAATCCCGCGCCTTGGTTTGCCATTCTCGTCTTCTCTTGACTAGTT

*K. cinerascens* TA-ATGCCTCAACTCAATCCCGCACCTTGATTTGCCATCCTTGTCTTCTCCTGAATAATT

*T. chatareus* TA-ATGCCCCAGCTGAATCCAGCACCTTGATTTACTATTCTAATCTTCTCCTGACTTATT

*D. berycoides* TAAATGCCCCAGCTAAACCCTGTGCCCTGGTTTGCTATCCTAATATTCTCTTGACTAATT

*H. typus* TA-ATGCCTCAACTCAATCCCGCACCTTGATTTGCCATTCTAATCTTTTCTTGACTAATT

*M. argenteus* TA-ATGCCTCAACTCAATCCCGCGCCTTGATTTGCCATTCTAGTCTTTTCCTGACTAGTT

*S. chuatsi* TAAATGCCTCAACTCAACCCCGCACCCTGATTTGCCATTTTAGTCTTCACCTGACTAATT

*O. fasciatus* TA-ATGCCTCAGCTCAACCCCGCTCCCTGATTTACTATTCTTATCTTCTCCTGAATAATC

*P. trilineatum* TAAATGCCCCAGCTTAACCCTTCGCCGTGATTTGCCATTTTAATCTTTTCATGAGTGACA

*M. salmoides* TA-ATGCCCCAACTCAACCCCGCACCTTGATTTGCCATTCTAGTCTTTTCGTGACTAATT

*P. tile* TA-ATGCCCCAACTCAACCCCGCTCCCTGATTTGCCATTCTAGTCTTCTCCTGACTAGTC

*L. argentimaculatus* TA-ATGCCTCAACTCAACCCCGCTCCTTGATTTGCCATTCTAGTCTTCTCCTGACTAGTT

*E. struhsakeri* TAAATGCCTCAACTCAATCCCGCACCTTGATTTGCTATTCTAGTCTTCTCCTGACTAGTC

*B. albus* TA-ATGCCACAACTTAACCCCCTCTGCTGATTTAACATATTAGCCTTCTCTTGAATAGCT

*C. auripes* TA-ATGCCTCAACTTGACCCGGCGCCGTGATTTAACATTCTTGTTTTTTCCTGGCTAGTT

*C. melampygus* T--ATGCCCCAACTAAACCCCGCACCCTGATTCGCCATCCTAACCTTCTCCTGACTAATC

*L. calcarifer* TAAATGCCTCAACTAAACCCCGCCCCATGGCTCGCCATCCTAACCTTTTCCTGGCTCATC

*S. maena* TA-ATGCCCCAATTAAACCCCGCCCCGTGATTAACCATTTTGGTTTTTTCCTGAGTAATT

*P. auriga* TAAATGCCCCAACTAAACCCCGCACCCTGGTTCGCCATCCTGGTATTTTCGTGAGTAATT

*C. lucidus* TA-ATGCCCCAATTAAACCCCTCCCCATGACTAGCCATCATAATTTTTTCATGACTAACA

*S. sihama* TA-ATGCCCCAGCTTGACCCCGCCCCGTGGTTCGCTATTTTCATCTTTTCTTGAATTGTC

*C. loricula* TA-ATGCCTCAACTTAACCCAAACCCCTGATTTGCCATTCTACTATTCACCTGAATAGTC

*A. trutta* TAAATGCCCCAACTTAACCCCACCCCCTGATTTCCCATTATAGCCTTCTCTTGACTAGTC

*H. gemma* TAAATGCCTCAACTCAACCCTGCCCCTTGATTTGCAATCCTAGTCTTCTCATGACTCGTT

*P. flavescens* TAAATGCCTCAACTCAACCCCGCACCTTGATTTGCTATTCTAGTCTTCACATGACTAGTC

*P. macrolepida* TAAATGCCCCAACTCAACCCCGCGCCCTGATTCGCCATTCTAGTTTTTACTTGACTAATT

*S. canadensis* TAAATGCCTCAACTCAACCCCGCGCCCTGATTTGCTATTCTAGTCTTCACATGACTAGTC

*A. rogaa* TTTTTAGGTTTTTTCCCTAAAAAAGTAATAACTCACACATTCCCATATGAACCTGCATCA

*C. argus* TTTTTAGGTTTTTTCCCCAAAAAAGTAATAGCACACACATTTCCATATGAGCCTGCATCT

*C. sonnerati* TTCTTAGGATTATTCCCTAAAAAAGTAATGACCCACACCTTTCCGTACGAACCCGCACCA

*E. fuscoguttatus* TTCTTAACCTTCTTCCCTAAAAAAGTAATAGCCCACACCTTCCCTTATGAGCCTGCCTCC

*E. coioides* TTCTTAATCTTCTTCCCCAAAAAAGTAACAGCCCATACTTTCCCTCACGAACCCGCCTCC

*E. bruneus* TTCTTAATCTTCTTCCCTAAAAAAGTAACGGCCCACACATTCCCTTACGACCCTGCCCCT

*E. moara* TTCTTAATCTTCTTTCCTAAAAAAGTAACGGCCCACACATTCCCTTACGACCCTACCCCC

*E. lanceolatus*  TTCCTAACTTTCTTCCCTAAAAAAGTAACGGCTCACACTTACCCCTATAATCCTACCCCC

*A. leucogrammicus*  TTCTTAGCCTTCTACCCTAAAAAAGTAACAGCCTATGCTTTCCCTTACGAACCTGCCCCC

*C. altivelis* TTCCTAATTTTTTACCCAAAAAAAGTAACAGCCCACACCTTCCCTTACGAACCTGTCTCC

*E. epistictus* TTCCTAGCCTTCTTCCCCAAAAAAGTAATAGCACATGATTTCCCCTACGAACCTACATCC

*E. octofasciatus* TTTCTAGGTTTCTTCCCAAAAAAAGTAATAGCTCACGTTTTCCCTTATGAGCCTATGTCC

*E. septemfasciatus* TTTTTAGGCTTCTTCCCAAAAAAAGTGATAGCCCACGTTTTCCCTTATGAGCCTATGTCC

*T. dermopterus* TTCTTAGGCTTCTTCCCCCAAAAAGTAACGGGCCACATTTTCCCATACGAGCCCGTGTCT

*E. awoara* TTCCTAGCCTTCTTCCCTAAAAAAGTAATAGCTCATACCTTTCCTTATCAACCTACATCT

*E. akaara* TTCCTAGCCTTCTTCCCTAAAAAAGTAATAGCTCACACCTTTCCTTACCAACCCACACCT

*E. trimaculatus* TTCCTAGCCTTCTTCCCTAAAAAAGTCATAGCCCACACTTTTCCATATCAACCCACACCT

*E. areolatus* TTCTTAACCTTCTTCCCTAAAAAAGTGATAGCCCACACTTTCCCCTACCAACCTGCACCC

*V. albimarginata* TTCCTTATCCTCTTCCCTAAAAAAGTTATAGCACACACCTTCCCTCATGAACCAGCATCC

*V. louti* TTCCTCACCCTCTTCCCTAAAAAAGTTATAGCACACACCTTCCCTCATGAACCAGCATCC

*P. leopardus* TTCTTAGGCTTTGTACCCACAAAAGTTATGAGCCACTCCTTCTCTAACGAACCTACGTCT

*P. areolatus* TTCTTATGTTTTGCACCTGCAAAAGTTAAAAGCCACTCCTTCTCTAATGAGCCTGCATCT

*E. radiosum* TTTTTAGCTATTGTTCCCACAAAAATTCTGGCCCACACTTACCCTAATGAGCCTACGTCT

*P. sieboldi* TTCCTGATCGTTATCCCACCTAAAGTTGCAGCCTACACATTCCCGAATGAGCTAACAGCC

*E. armatus* TTTTTAACTGTAATTGTGCCCAAGGTTTTAAGCCATACTTTTCCTAACGAACCCACACCA

*R. oxyrhynchus* CTACTAGTCGTTATTCCCCCTAAAGTCGTAGCCCACACATTCCCTAAAGAGCCGACCCTT

*K. cinerascens* TTCCTAGTCGTTATCCCTCCAAAAGTTATAGCCCACACCTTTCCAAACGAACCCACCCCT

*T. chatareus* TTCTTAACAGTCCTTCCCCCAAAAATCATGGCTCACTCTTACCCAAATGTACTCACCCAT

*D. berycoides* TTCCTAACTGTAGTACCCCCGAAAGTCTTGGGCCATGTCTCCCTAAATGACTTCGTCCTT

*H. typus* TTCCTAACTGTGGTCCCCCCAAAAGTTATGGCCCACACATTCCCAAATGAGCCCTCCCCC

*M. argenteus* TTCCTAACCGTTCTTCCCCCAAAAGTTATAGCCCACACTTTCCCAAATGAGCCAACCTCT

*S. chuatsi* TTCCTAACCGTTATTCCTCCTAAAGTGCTAGCTCATACTTTCCCCAACGAGCCAACTCTT

*O. fasciatus* TTTTTAATTGTTATCCCACCAAAAGTTATAGCCCACACTTTCCCATACGAACCCACACCT

*P. trilineatum* TTTTTAATTGTCATCCCTCCAAAAATTATGGGTCATACCTTTCCAAACGACCCCAGCCCA

*M. salmoides* TTCCTAACCGTCCTTCCTCCTAAAGTCCTAGCACACACTTTCCCTAATGAACCCTCACTC

*P. tile* TTCCTAACCATTCTTCCCCCTAAAGTTATAGCCCATACTTTCCCAAACGAGCCCACCTCT

*L. argentimaculatus* TTCCTAACCATTCTTCCCCCTAAAGTTATAGCCCACACTTTCCCAAATGAGCCAACCCCT

*E. struhsakeri* TTCCTAACAATTCTTCCTCCAAAAGTTATAGCCCACACCTTCCCAAACGAGCCAACCCCT

*B. albus* TTCACAACCGTAATCCCCACAAAAATTAAAAATGTGTCTTTCCCAAACTTCCCTGAACTT

*C. auripes* TTTCTTGTTATTATCCCCCCGAAAATCATAGCCCACACTTTCCCTAACGAGCTTAACCCA

*C. melampygus* TTTCTTACAGTTCTTCCCTCTAAGGTTATGTCCCACACATACCCAAACGAGCCAACCCCT

*L. calcarifer* TTCCTAACAGTAATTCCTCCTAAAATTCTAGCCCACTCTTTCCCTAATGAACCTACCCCC

*S. maena* TTTCTTACCATCTTACCCTTAAAAATTGTAGCCCACACTTTCCCTAACGAGCCAACACTT

*P. auriga* TTTCTTACCATCCTACCCCCCAAAATCATAGCCCATACTTTCCCCAACGAGCCCACTCTT

*C. lucidus* TTTCTAATTATTGTCCCACCCAAAGTTATAGCTCATATTTTCCCAAATGAACCATCCCCA

*S. sihama* TTCCTTATTTTCCTTCCCCCTAAAGTAATAGCTCACTCTTTCCCTAACGATCCTACTGCC

*C. loricula* TTCCTTTACTTCCTCCCTACAAAAATCATAGGCCACACCTTCCCAAGCGAACAAACCGCT

*A. trutta* TTCTTAATTGTTTTACCCCCCAAAATCATCGCCCACGCCACCCCAAATGTACCTACACCC

*H. gemma* TTTTTAATTGTAATCCCGCCAAAAGTAATAAACTATACATATCCTAACGAACCTTCCCCT

*P. flavescens* TTTTTAACTATTATTCCCACAAAAATCCTGGCTCACACCTACCCTAATGAGCCTACATCT

*P. macrolepida* TTCCTAATTATTGTCCCCACAAAAATTCTAGCCCACACCTACCCCAATGAACCCACCTCA

*S. canadensis* TTTTTAACTATTGTTCCCACAAAAATTCTGGCCCACACCTACCCCAATGAACCTACGTCT

*A. rogaa* CTCAAGCCACAAAAACTAGAAAAAGCCCCTTGAAACTGACCCTGAGCGTAAGCTTTTTTG

*C. argus* TTCAAACCACAAAAGCTAGAAAAAACCCCTTGAAACTGACCTTGAGCGTAAGCTTCTTTG

*C. sonnerati* CTTAAATCACAAAAGTTAGACAAAACCCCCTGAAACTGACCCTGAGCGTAAGCTTTTTCG

*E. fuscoguttatus* ATTAAGCCTCAAAAGCTGGTAAAAACCTCTTGAAACTGACCCTGAGCGTAAGCTTTTTTG

*E. coioides* CTCAAGCCTCAAAAGCTAGAGAAAACCTCTTGAAATTGACCCTGAGTGTAAGTTTTTTTG

*E. bruneus* CTCAAGTCTCGAAAGCTAGAGAAAACCCCTTGAAATTGACCATGAGTGTAAGTTTTTTTG

*E. moara* CTCAAGCCTCAAAAGCTAGAGAAAACCTCTTGAAATTGACCATGAGTGTAAGTTTTTTTG

*E. lanceolatus*  CTTAAACTCCAAAAACTAGAGAAAACCTCTTGAGATTGACCCTGAGTGTAAGCTTCTTTG

*A. leucogrammicus*  CTTAAGCCTCAAAAGCTAGAAAAGACTTCTTGAAACTGACCCTGAGTGTAAGTTTTTTTG

*C. altivelis* CTTAAGTCTCAAAAACTAGAAAAAACCTCTTGAAACTGACCCTGAGCGTAAGCTTTTTTG

*E. epistictus* CTTAAGCCTCAAAAACTAGAAAAAACCCCCTGAAACTGGCCCTGAGTGTAAGCTTTTTTG

*E. octofasciatus* CTCAAGCCTCAGAAGCTAGAGAAAATCTCTTGAAATTGGCCCTGAATGTAAGTTTTTTCG

*E. septemfasciatus* CTCAAGCCTCGAAAGCCAGAGAAAATCTCCTGAAATTGACCATGAGTGTAAGTTTTTTCG

*T. dermopterus* CCTAAGCTTCAAAAGCTGGAGAGAGCATCTTGAGACTGACCCTGAGTGTAAGCTTTTTTG

*E. awoara* CTTAAACCCCAAAAACTAGAAAAAACCTCCTGAAACTGACCCTGAGCGTAAGCTTTTTTG

*E. akaara* CTCAAACCCCAAAAACTAGAGAAAACCTCCTGAAACTGACCCTGAGTGTAAGCTTTTTTG

*E. trimaculatus* CTTAAACCTCAAAAATTAGAAAAAACCTCCTGAAACTGACCCTGAGTGTAAGTTTTTTTG

*E. areolatus* CTCAAACCCCAAAAACTAGAAAAAACCTCCTGAAACTGACCCTGAGTGTAAGCTTTTTCG

*V. albimarginata* CTAAAACCACAAAAATTAGAAAAAACATCCTGAAACTGACCTTGAGCTTAAGCTTTTTTG

*V. louti* CTAAAGCCACAGAAATTAGAAAAAACATCTTGAAACTGACCCTGAACTTAAGCTTTTTTG

*P. leopardus* GTAAAACCACAAAAACTAGAAAAGACATCTTGAAACTGACCATGGGCTTAAGCTTCTTCG

*P. areolatus* GTTAAGACACAGAAATTAGAAAAGACTTCTTGAAACTGACCATGATCTTAAGCTTCTTTG

*E. radiosum* CAAAGCACTGAGAAACCTAAAACAGAACCCTGAACCTGACCATGACACTAAGCTTCTTTG

*P. sieboldi* CAAAGCACTCAAACCCCCGTAACAGAGCCCTGAAACTGACCATGATCCTAAGCTTTTTCG

*E. armatus* CAAAGCACAGAAAAACCAAAAACAGAACCCTGAAACTGACCATGACACTAAGCTTCTTCG

*R. oxyrhynchus* CAAAGTGCAGAAAAACCTAAAACAGAACCCTGAAACTGACCATGACACTAAGCTTCTTCG

*K. cinerascens* CAAAGCACAGAAAAGGCTAAAACAAACCCCTGAAACTGACCATGACACTAAGCTTTTTCG

*T. chatareus* CAAAACACAAAAAAAACTAAAACCGAAGCCTGACCCTGACCGTGACACTAAGCTTCTTTG

*D. berycoides* CAAAGCATTAAAGCCTTTAAAACGGCATCCTGAAACTGACCATGATACTAAGCCTTTTCG

*H. typus* CAAAGCACAGAAAAACCCAAAACAGAGCCCTGAAACTGACCATGACATTAAGCTTTTTCG

*M. argenteus* CAAAGCACAGAAAAACCCAAAACAGAGCCCTGAACCTGACCATGACACTAAGCTTTTTCG

*S. chuatsi* CAAAGCGCAGAAACCCCTAAAACAGAGCCCTGAAACTGACCATGACACTAAGCTTCTTTG

*O. fasciatus* CAAAGCGCGGAAAAACCCAAAACAGACTCTTGAAACTGACCATGACACTAAGCTTTTTTG

*P. trilineatum* CAAAATACAGCAAAACCTAAAACAGGATCTTGACACTGACCTTGGCAATAAGCTTCTTTG

*M. salmoides* CAAAGCACTGAGACCCCTAAAACAGAGCCCTGAAACTGACCATGACACTAAGCTTTTTCG

*P. tile* CAAAGTACTGAGAAACCCAAAACAGAGCCCTGAACCTGACCATGACACTAAGCTTCTTCG

*L. argentimaculatus* CAAAGCACTGAGAAACCTAAAACTGAGCCCTGAACCTGACCATGACACTAAGTTTCTTCG

*E. struhsakeri* CAAAGCACACAAAAACCCAAAACAGAACCCTGAACCTGACCATGACATTAAGCTTTTTTG

*B. albus* AAAGAAACAGACAAACCTGAGACTAAATCCTGAACCTGACCATGGCTCTAAGCTTTTTTG

*C. auripes* CAGGGCACCCACAAACCCAAAATGAGCTCCTGAGCCTGGCCATGGCACTAAGCTTCTTTG

*C. melampygus* CAAAGCACAGAAAAACCTAAAACAGAAGCCTGATCTTGACCTTGGCAATAAGCTTCTTCG

*L. calcarifer* CACAGCACAAAAGCTCCTAAAACTGATCCCTGAATGTGACCATGGCATTAAGCTTTTTTG

*S. maena* AAAAGCGCTGATACACCTAAAACAGAGTCCTGAACCTGACCATGGTAGCAAGTTTTTTTG

*P. auriga* CAAAGCACTGAAAAGCCTAAGACAGAGCCCTGAACCTGACCATGATACTAAGCTTTTTTG

*C. lucidus* CAAAGCACAGAAACTCTAAGCATAGAAACCTGAAACTGACCATGACTGTAAGCCTCTTCG

*S. sihama* CAAGAGCAAAAAAAAACTAAAACCCCTGCTTTAACCTGACCATGGCCGTAAGTTTTTTTG

*C. loricula* CAAACCCCTCACTTCCCACAACCAGAAAACTGACCCTGACCATGACTTTAAGCCTATTTG

*A. trutta* TTAAGTGCCCGAAAAGAAAAAGGCAACACCTGAAATTGACCATGGCTTTAAGCTTCTTTG

*H. gemma* CAAAGCTCTCAAGCACCTAAAACTGAATCTTGAAACTGACCATGGCACTAAACTTTTTTG

*P. flavescens* CAAAGCACAGAAAAACCTAAAACAGAGCCCTGAACCTGACCATGACACTAAGCTTCTTTG

*P. macrolepida* CAGAGTACTGAGAAACCTAAGACAGAGCCCTGAACCTGACCATGACACTAAGCTTCTTTG

*S. canadensis* CAAAGCACTGAGAAACCTATACCAGAGCCCTGAACCTGACCATGATACTAAGCTTCTTTG

*A. rogaa* ACCAATTTATAAGCACAACATATATAGGAATTCCCCTAATTGCACTAGCACTAGTATTTC

*C. argus* ACCAATTTATAAGCACAACATACATAGGAATCCCTCTAATCGCATTAGCACTAGTATTCC

*C. sonnerati* ATCAATTCATAAGCACAACCTACCTGGGAATTCCTTTAATTGCAATTGCACTAACATTCC

*E. fuscoguttatus* ACCAGTTTATAAGTACAACATGTCTAGGAATTCCCCTAATTGCATTAGCACTAACCTTTC

*E. coioides* ACCAGTTTATAAGCACAACATATCTAGGAATCCCTTTAATCGCACTAGCACTAACCTTTC

*E. bruneus* ACCAGTTTATAAGCACAACATACTTAGGGATCCCCTTAATTGCACTAGCGCTAACCTTTC

*E. moara* ACCAGTTTATAAGCACAACATACTTAGGGATCCCCTTAATCGCACTAGCATTAACCTTTC

*E. lanceolatus*  ACCAGTTTATAAGCACAACATATTTGGGCATCCCTTTAATCGCACTAGCACTGATCTTTC

*A. leucogrammicus*  ACCAATTTATAAGCACAACATACTTAGGTATTCCCCTAATTGCACTAGCACTTACCTTTC

*C. altivelis* ACCAATTTATAAGCACAACGTACTTAGGAATTCCTTTAATTGCACTAGCACTAACCTTTC

*E. epistictus* ATCAATTTATAAGCACAACATATTTAGGAATTCCCTTAATTGCACTAGCATTAACATTTC

*E. octofasciatus* ATCAGTTCATAAGCACAACATATCTAGGCGTCCCTCTAATTGCACTAGCACTAATATTCC

*E. septemfasciatus* ATCAGTTTATAAGCACAACATATTTAGGAATCCCTCTAATTGCACTGGCACTAACATTCC

*T. dermopterus* ACCAATTTATAAGCACGACATATTTAGGGGTCCCCCTAACCCTACTGGCCCTAACATTCC

*E. awoara* ACCAATTTATAAGCACAACATATCTAGGAATCCCCTTAATTGCACTAGCACTTATTTTTC

*E. akaara* ACCAATTTATAAGCACAACATATTTAGGAATTCCCTTAATTGCGCTAGCACTCATATTTC

*E. trimaculatus* ATCAATTTATAAGCACAACATACCTAGGAATTCCTTTAATTGCACTAGCACTTATATTTC

*E. areolatus* ATCAATTTATAAGCACAACATACCTGGGGATTCCCTTAATTGCACTAGCACTTATTTTTC

*V. albimarginata* ACCAATTTATAAGCACCACATACCTGGGTATTCCACTCATCGCGCTCGCACTTACATTCC

*V. louti* ACCAATTTATAAGCACCACATACCTAGGTATTCCACTCATCGCGCTCGCACTCACATTTC

*P. leopardus* ATCAATTTATAAGCACCACTTATTTAGGAATTCCGCTTATTGCCATGGCATTAGTGTTTC

*P. areolatus* ACCAATTTATAAGCACCACTTATTTAGGCATCCCACTTATTGCTCTTGCATTAACGTTCC

*E. radiosum* ACCAATTTATAAGCCCCACATTTATAGGAATTCCCTTAATAGCTCTAGCCCTCTCCCTCC

*P. sieboldi* ACCAATTCATAAGCCCCGTCTTCCTAGGCATCCCCCTGATTGCCCTCGCCCTAGTACTCC

*E. armatus* ATCAATTTATAAGTCCCGTATGCTTAGGCATTCCCCTAATTGTTCTTGCCCTCACCCTCC

*R. oxyrhynchus* ACCAATTTATATCCCCCTCCCTCCTCGGAATTCCCTTAATAGCCCTTGCTTTAACCCTGC

*K. cinerascens* ATCAATTTATGAGCCCTGTGTTCCTAGGCATCCCTTTGATTGCCCTCGCCCTCACCCTCC

*T. chatareus* ACCAATTTATAAGCCCAACATATCTTGGCATCCCCCTAATAGCACTAGCCCTCACACTAC

*D. berycoides* ACCAGTTTATAAGCCCAACATTTCTAGGAGTCCCTCTAATTGTCCTAGCTCTGACCCTCC

*H. typus* ACCAATTTATGAGCCCCTCGTACCTGGGTATCCCCTTAATAGCCCTCGCCCTTACTCTCC

*M. argenteus* ACCAGTTTATGAGCCCCACCTACCTGGGTATTCCCCTTATAGCCCTCGCACTTAGTCTTC

*S. chuatsi* ACCAATTCATGAGCCCCACATATTTAGGTATCCCCTTAATGGCTCTAGCCTTAAGCCTCC

*O. fasciatus* ACCAGTTTATGAGCCCCGTTTTCCTGGGCATTCCCCTGATTGCCCTTGCATTAACCCTTC

*P. trilineatum* ACCAATTTATAAGCCCCACATATTTGGGCATTCCCTTAATAGCACTCGCACTTACCCTCC

*M. salmoides* ACCAATTTATGAGCCCCACTTACCTAGGCATCCCTCTAATTGTCCTAGCCCTCAGCCTCC

*P. tile* ACCAATTTATGAGCCCCAACTACCTAGGCATTCCCCTAATAGCCCTTGCCCTCAGCCTCC

*L. argentimaculatus* ATCAATTTATAAGCCCCTCCTACCTGGGCATCCCCTTAATGGCTCTCGCCCTTAGCCTCC

*E. struhsakeri* ACCAATTCATGAGCCCCTCATACCTAGGCATTCCCCTAATAGCCCTTGCCCTCAGCCTCC

*B. albus* ATCAATTTGCAAGCCCAACATACCTGGGAATCCCCCTAGTGGCGATTGCCCTCAGCCTGC

*C. auripes* ATCAATTTCTAAGCCCAGTACTTTATGGAATCTCCCTAACAGCTCTTGCCCTAACACTTC

*C. melampygus* ATCAATTTATAAGCCCAGTTTATTTAGGCGTACCACTAATAGCACTTGCTTTGACACTAC

*L. calcarifer* ACCAATTTATATCTCCAAATTTTTTAGGAATTCCGCTGATTGCTATGGCCATTGCCCTCC

*S. maena* ATCAATTCGCAAGCCCATTCTTTTTAGGAATCCCGCTAATAGCGCTCGCCCTACTTCTCC

*P. auriga* ACCAATTTTCAAGCCCATTTTTTATAGGAATCCCCCTCATAGCCCTCGCCCTCGTTCTCC

*C. lucidus* ACCAGTTTATATCCCCCACATACCTAGGAGTACCACTACTAGCGATTGCTCTCACCCTCC

*S. sihama* ACCAATTCATAAGTCCTGTTTTTATGGGCATCCCCTTAATTGCCTTAGCCATCTCTCTAC

*C. loricula* ACCAATTCTCATACCCTTGACATATAGGCATCCCTCTAGTAGCCATCGCTCTCACACTCC

*A. trutta* ACCAATTTGAAAGCCCTATTCTTTTAGGCACACCCCTGATCGTCCTAGCCCTCACCCTCC

*H. gemma* ATCAATTCTTAAGCCCTTCCTTTTTAGGTATTCCTCTCATAGCCCTAGCTCTAACCCTGC

*P. flavescens* ATCAATTTATGAGCCCCACATATTTAGGCATTCCCCTTATAGCCCTCGCTTTAACTCTCC

*P. macrolepida* ACCAATTCATGAGCCCCACATTTATAGGTGTCCCTCTTATGGCCCTAGCCCTCTCTCTCC

*S. canadensis* GCCAATTTATGAGCCCCACATATCTCGGGATTCCCCTTATAGCCCTTGCCCTAACCCTCC

*A. rogaa* CCTCCATCCTCTATCCAACACCAACAACACGATGATTAAACAACCGACTTATTACACTAC

*C. argus* CATCCATCCTATATCCAACATCAACATCACGATGATTAAATAATCGACTTGTGACACTAC

*C. sonnerati* CTGCTATCCTGTATCCAACACCTACAACCCGATGACTAAATAATCGACTTTTAACCCTAC

*E. fuscoguttatus* CTTCTATTTTATACCCTACAGCCACAACTCGATGACTAAACAACCGACTACTCACACTAC

*E. coioides* CCTCTATTCTATACCCTACAACCACAACCCGATGACTAAATAACCGGTTACTTACATTAC

*E. bruneus* CTTCCATCTTATACCCTACGACCTCAACCCGATGACTAAACAACCGACTACTTACGCTAC

*E. moara* CTTCCATCTTATACCCTACAACCTCAACCCGGTGACTAAACAACCGACTGCTTACACTAC

*E. lanceolatus*  CTTCTATTTTATACCCCACTATTACAACCCGGTGGCTTAACAACCGACTACTCACACTAC

*A. leucogrammicus*  CCGTTATTCTATACCCTACAACTACAACCCGATGATTAAACAACCGACTACTTACACTTC

*C. altivelis* CCATCATCTTATACCCCACAACTACAACCCGATGACTAAACAACCGACTATTAACACTAC

*E. epistictus* CCTCTATTCTTTATCCCACACTCACAACCCGATGGTTAAATAACCGACTTCTTACACTAC

*E. octofasciatus* CTTCTATTCTGTACCCCACAACCACAACCCGATGGTTAAATAACCGACTTTTAACACTAC

*E. septemfasciatus* CCTCTATTCTGTACCCCACAACCACAACCCGGTGGTTAAATAACCGGCTCTTAACACTAC

*T. dermopterus* CTATTATTCTCTATCCCACAGCCACAGCCCGCTGACTAAATAACCGACTTTTAACACTTC

*E. awoara* CCACTATTCTTTATCCTACGCAAACAGACCGCTGACTAAATAACCGACTCCTTACCTTAC

*E. akaara* CTTCCATTCTATACCCTACACAAACAAATCGGTGACTAAATAACCGACTTCTTACTTTAC

*E. trimaculatus* CCTCCATCCTCTACCCCACAACAACAACCCGCTGATTAAATAATCGACTTCTCACTTTAC

*E. areolatus* CTACCATCCTCTACCCCACACTAACAACCCGATGATTAAATAACCGGCTCCTTACCCTAC

*V. albimarginata* CTTCACTTCTATACCCCACAGCATCAACCCGATGACTTAATAACCGACTCCTAACCCTAC

*V. louti* CCTCACTTCTATACCCTACAGTATCAACCCGATGACTTAATAATCGGCTCGTAACCCTGC

*P. leopardus* CTTGCCTCCTTTTTCCTACCTTAACGACACGATGACTAAACAACCGTCTCACAACACTCC

*P. areolatus* CTTACCTCCTCCTCCCCTCTTCAACAACACGATGATTAAACAATCGCCTCGTGACACTCC

*E. radiosum* CCTGGCTTCTCTACCCTGCGCCTTCTGCTCGGTGATTAAACAACCGTTTTCTTGCCCTTC

*P. sieboldi* CATGAATTCTTTTCCCCAAACCTTCCGCACAATGAATCACTAACCGACTCCTCACCCTTC

*E. armatus* CATGAATCCTCTTTCCTTCTTTTGCCTCTCGATGACTTCCCAACCGATTACTAGGCCTCC

*R. oxyrhynchus* CATGAACACTCTTTCCTGCCCCCACACCCCGATGACTCCATAATCGAATATTAACCCTTC

*K. cinerascens* CTTGAATCCTATTCCCAACCCCGTCCCCTCGATGACTAAACAACCGTATATTAGCCCTCC

*T. chatareus* CATGAGTCTTATTCCCCACCCCCTCTGCCCGATGATTAAACAATCGAGTACTAACCCTTC

*D. berycoides* CCTGACTCCTCTTCCCTATCCCCTCCAACCGATGATTAAATAACCGATTCCTAACACTTC

*H. typus* CCTGAGTCCTATACCCCACTCCCTCACACCGATGACTAAACAACCGCCTATTAACCCTTC

*M. argenteus* CATGAATTCTCTTCCCGACTCCTTCCACCCGATGACTAAACAATCGTCTACTCACCCTTC

*S. chuatsi* CTTGAATCCTTTACCCCATCCCCTCCTCCCGATGACTCAACAACCGACTATTAGCCCTTC

*O. fasciatus* CGTGAATCCTTTTCCCCACCCCCACATCCCGATGATTAAATAACCGCTTACTCGCCCTAC

*P. trilineatum* CCTGAATCCTCTTCCCAACTCCCACTACCCGATGACTCGATAATCGCCTATTAACCCTTC

*M. salmoides* CATGAGTACTCTTCCCCACTCCCTCCTCCCGATGACTAAATAATCGCCTTTTAACCCTTC

*P. tile* CTTGAACTCTTTATCCAACTCCCTCAACACGATGGTTAAACAACCGTCTACTAACCCTTC

*L. argentimaculatus* CCTGAACTCTGTACCCAACCCCCTCTACACGATGATTAAACAACCGTCTGTTGACACTCC

*E. struhsakeri* CCTGAATTCTCTTCCCAACTCCCTCCGCCCGATGACTAAATAACCGAATGTTAACCCTCC

*B. albus* CCTGAGTTTTATACCCTACACCCTGCGGACGATGACAAAGCAACCGGCTACTAACAGTCC

*C. auripes* CTTGAGTTCTTTTTCCAACCCCAACCTCCCGCTGACTTAACAATCGTCTCTTATCCCTCC

*C. melampygus* CCTGAGTCCTCTACCCAACCCCGTCCGCACGATGACTAAACAATCGACTACTTACCCTAC

*L. calcarifer* CATGGACCCTCTATCCAACACCATCAACTCGATGATTAAGCAACCGACTACTAACCCTTC

*S. maena* CGTGAGTCCTTGTCCCGACCCCCTCAGCCCGATGACTCGATAACCGCCTGGTAACACTAC

*P. auriga* CCTGAACCCTATTTCCAGCACCTTCTTCTCGTTGACTAAATAACCGCCTAGCAACCCTCC

*C. lucidus* CCTGAATTTTCTTCCCAGCCCCCACCTTCCGATGACTCCATAACCGCCTACTAGTACTAC

*S. sihama* CTTGAGTTTTATTCCCCACCCCCTCATCGCGTTGATTAACTAACCGCGTTTTAACCCTTC

*C. loricula* CCTGAATTCTTTACCCAACACCCTCTGCCCGGTGACTAAATAACCGAATATTGACCTTCC

*A. trutta* CCTGACTGCTTATCCCGACACCCACTTCTCAATGACTAAACAATCGGCTGCTATCCACCC

*H. gemma* CGTGAGTCCTATTTCCTTCTCCCTCTTCCCGATGACTTAACAATCGTGTCGTGACACTTC

*P. flavescens* CCTGAGTCCTTTATCCTACCCCCTCTGCTCGATGATTAAACAACCGCTTCCTCGCTCTAC

*P. macrolepida* CTTGAATCCTCTACCCTGCCCCCTCCGCTCGATGACTAAACAACCGACTTCTTTCCCTTC

*S. canadensis* CCTGGATCCTTTACCCTACACCCTCTGCTCGATGGCTAAACAACCGCTTCCTTGCATTAC

*A. rogaa* AAAGCACATTTATTAACCGCTTTACACATCAACTGCTCCTACCACTAAATGTAGGCGGAC

*C. argus* AAAGCACATTTATCAACCGCTTTACTCATCAACTGCTTTTGCCATTAAATGTAGGCGGAC

*C. sonnerati* AAAGTGTATTCATCAACCGCTTCACTCACCAACTTCTACTTCCCCTAAACGTAGGCGGAC

*E. fuscoguttatus* AAAGCGCATTTATCAATCGCTTCACTCACCAACTACTCCTGCCTCTAAATGCAGGTGGCC

*E. coioides* AAAGCGCATTCATCAATCGCTTTATTCACCAGCTTCTCCTGCCCTTGAATGTAAGCGGCC

*E. bruneus* AAAACGCATTCATCAATCGCTTCACTCACCAACTACTTCTACCCCTAAATGTGGCTGGCC

*E. moara* AAAACGCGTTCATCAATCGCTTCACTCACCAACTACTTCTACCCCTAAACGTGGCTGGCC

*E. lanceolatus*  AAAGCGCGTTCATTAACCGCTTTACGCACCAACTTCTACTGCCCCTAAATGTAGGCGGCC

*A. leucogrammicus*  AAGGCGCATTCATCAACCGTTTTACTCAACAACTTCTCCTGCCCTTAAATGTAGCAGGCC

*C. altivelis* AAAACACATTCATTAATAGTTTTATTCACCAACTACTCCTACCCCTAAATGTAAGCGGAC

*E. epistictus* AAAGCGCATTCATTAACCGCTTTACCCACCAACTGCTCTTACCCTTAAATGTCGGCGGAC

*E. octofasciatus* AAAGCGCATTCATTAACCGCTTCATCCATCAACTACTATTACCTTTAAATGTAAGCGGAC

*E. septemfasciatus* AAAGCGCATTCATTAACCGCTTCATCCATCAACTACTACTACCTTTAAATGTAAGCGGAC

*T. dermopterus* AAAGCGCATTCATCAACCGCTTCACCCGGCAACTGCTGCTGCCTTTGAATGCGGGGGGAC

*E. awoara* AAAACGCATTCATTAATCGCTTCACCCACCAGCTACTCCTGCCCTTAAATGTTGGAGGCC

*E. akaara* AAGACGCATTTATTAACCGCTTCACCCACCAACTACTTCTACCCTTAAATGTAGGAGGAC

*E. trimaculatus* AAGGCGCATTCATCAACCGCTTTATTCACCAACTTCTCCTGCCCTTAAACGTAGCTGGTC

*E. areolatus* AAAGCGCATTTATTAACCGCTTTACCCATCAACTACTTCTGCCCCTAAATGTTAATGGGC

*V. albimarginata* AGAGCACATTTATTAACCGTTTTGTTCACCAACTTCTTTTACCATTAAACGTTCAAGGTC

*V. louti* AAGGCACATTTATTAATCGTTTTATTCACCAACTTCTTTTACCACTAAATGTCCAAGGTC

*P. leopardus* AAGGCTTGGTCATCAACCGCTTTACCCATCAACTACTATCACCACTAAATGTAGCAGGTC

*P. areolatus* AAGGCCTATTTATTAATCGCTTTACCCATCAACTTCTACTGCCACTTAATGTACGAGGTC

*E. radiosum* AAAGCTGATTTATTAACCGCTTTACCCAACAGATACTTCTTCCATTGAACGCTGGGGGAC

*P. sieboldi* AAGGATGGTTCATTAATCGATTTACTCAACAACTCTTTCTTCCCTTAAACACCCCGGGGC

*E. armatus* AAAACTGATTCCTCAGTCAATTTACTCAACAGCTTCTTTTACCATTAAGCCCTGCCGGAC

*R. oxyrhynchus* AAAACTGATTTATTGGTCGCTTCTCTCAAGAACTCCTCCTCCCACTAAGCCCTGCTGGAC

*K. cinerascens* AAAACTGATTTATTAACCGCTTTACCCAACAACTTCTCCTGCCCCTCAGCTTAGGCGGCC

*T. chatareus* AAGGCTGATTTATTAATCGATTTACTTATCAACTTCTCCTCCCCCTGAACCCCGGAGGCC

*D. berycoides* AAACCTGATTTATTTACCGATTTGTCCGACAACTTTTCCTCCCCATACACCGATCAGGGC

*H. typus* AAGGCTGATTCATCGGCCAATTCACCAAACAACTTCTCTTGCCCGTAAACCTAGGCGGGC

*M. argenteus* AAAGTTGATTTATTAATCGATTTACTCAACAGCTCCTCCTGCCCCTAAATCCAGGGGGCC

*S. chuatsi* AAAACTGATTCATCAACCGATTCACCCAGCAACTTCTCCTACCCCTAAGCCCCGGGGGCC

*O. fasciatus* AAAGCTGATTCATTAACCGGTTTACACAACAACTTCTTCTACCCCTGAGCTTAGGAGGCC

*P. trilineatum* AAAACTGATCTATCAACCGATTCACCCAACAACTTCTTCTACCTATCAATGTAGGAGGAC

*M. salmoides* AGGGCTGATTTGTAAATCGATTTACTCAACAACTCCTCCTACCACTCAACCCGGGGGGTC

*P. tile* AAGGCTGATTTATCAACCGATTTACACAACAACTTCTTCTGCCCCTAAATCCCGGAGGCC

*L. argentimaculatus* AAAGTTGATTCATCAACCGATTTACCCAGCAACTCTTACTACCCCTAAATCCTGGAGGCC

*E. struhsakeri* AAAATTGATTTATTAACCGGTTTACCCAACAACTTCTTCTACCCCTAAACCCCGGGGGTC

*B. albus* AAAACTGATCTATCGCCCGATTTACCCAACAGCTACTCCTGCCCTTAAATGTAGGAGCCC

*C. auripes* AAAGTTGATTCATTAGTCGCTTCACACAGCAACTCCTCCTACCTCTAAACCCTGCAGGCC

*C. melampygus* AAGGCTGATTCATTAACCGCTTTACACAACAACTTCTCCTCCCCTTAAACCCAGGAGGGC

*L. calcarifer* AAAACTGATCAATCAACCGATTTACTCAACAGCTTCTTCTGCCTTTAAATCAAGAGGGGC

*S. maena* AAAACTGATTCCTTGGCATATTCACACGACAACTTCTTCTACCAATCAATCGCCCAGGGC

*P. auriga* AAGGCTGATTCGTTAGCTCATTTGCACGTCAACTTCTTATGCCTGTCAACCAACCTGGAC

*C. lucidus* AAGGCTGGTTCATCAACCGATTTACACACCAAATTTTTACCCCCTTAAGCCAAGGCGGAC

*S. sihama* AAAACTGATTCCTTGGCCGATTCGCTAATCAATTAATAATACCCCTAAACGTCGGGGGGC

*C. loricula* AAGGCTGATTCATCGCCCGATTTACTTACCAAACCTTCATACCCCTAAATGTCGGAGGAC

*A. trutta* AAAACTGATTCATTAGCCGCACCACTCGAGAACTTTTCATTCCTGTCAACCATCCAGGAC

*H. gemma* AAAGCTGGTTTATTAATCGCTTTACTCAACAACTTTTACTTCCCCTTAATAAAGGAGGAC

*P. flavescens* AAGGTTGATTTATTAACCGCTTTACCCAACAACTTCTTCTCCCTTTAAGCCTAGGCGGTC

*P. macrolepida* AAGGTTGATTTATTAATCGCTTTACGCAGCAGCTTCTTCTCCCATTGAACACAGGAGGCC

*S. canadensis* AAGGCTGGTTTATTAACCGATTTACTCAACAGCTTCTTCTTCCTTTAAGCCTAGGAGGCC

*A. rogaa* ATAAATGAGCCACTATTTTAGCCTCCTTAATAATGTTTTTAATTTCATTAAACATATTAG

*C. argus* ATAAGTGAGCCACCATCCTGGCCTCCTTAATAATGTTCTTAATTTCGTTGAATATACTAG

*C. sonnerati* ATAAATGAGCAACCATCCTAGCTTCATTAATGATCTTCTTAATCTCACTAAACATATTAG

*E. fuscoguttatus* ATAAATGGGCCACTCTCTTGGCTTCCCTGATACTCTTTTTAATCTCACTAAATATGCTAG

*E. coioides* ATAAATGAGCCACCCTTCTAGCCTCCCTAATGCTCTTCTTAATTTCACTCAACATGCTCG

*E. bruneus* ACAAGTGAGCCACTCTTCTGGCCTCCTTAATACTCTTTTTAATCTCACTAAACATGCTCG

*E. moara* ACAAGTGAGCCACTCTCCTGGCCTCCTTAATACTCTTTTTAATCTCGCTAAACATGCTCG

*E. lanceolatus*  ACAAGTGAGCTACCCTTCTAGCCTCCCTAATACTCTTCTTAATCTCGCTTAACTTGCTCG

*A. leucogrammicus*  ATAAATGAGCCACCCTTTTAGCCTCCCTAATACTCTTTTTAATCTCATTAAACATGCTCG

*C. altivelis* ATAAATGAGCCACCCTCCTAGCCTCCTTAATACTCTTTTTAATTTCACTAAACATGCTCG

*E. epistictus* ATAAATGAGCTACCTTGTTGGCCTCTTTAATAATCTTTTTAATCTCCCTAAACATGCTTG

*E. octofasciatus* ATAAATGAGCCACCATCTTGGCCTCCCTAATACTCTTTTTAATCTCGTTAAACATGCTTG

*E. septemfasciatus* ATACATGAGCCACCATCTTGGCCTCCCTAATACTCTTTTTAATCTCGTTAAATATACTTG

*T. dermopterus* ACAAATGAGCCACTATCCTGACCTCTTTAATACTATTTCTGATCTCAATAAACATGTTGG

*E. awoara* ATAAATGAGCCACCCTTCTGGCATCCTTAATGCTCTTTTTAATTTCACTAAACATGCTAG

*E. akaara* ATAAATGAGCTGCCCTTTTAGCATCCTTGATGCTCTTTTTAATTTCACTTAATATGTTAG

*E. trimaculatus* ATAAATGAGCCACCCTCCTAGCCTCCTTAATGCTTTTCCTAATTTCGCTAAACATGCTCG

*E. areolatus* ACAAATGAGCCGCCCTTCTAGCATCCTTAATGCTCTTTTTAATTTCACTGAACATGTTAG

*V. albimarginata* ACAAATGAGCTGTAATCCTTACATCCTTAATAATCTTTTTAATTTCCCTAAACATGCTTG

*V. louti* ATAAGTGAGCTGTAATCCTTACATCCTTGATAATCTTTTTAATTTCCCTAAATATGCTCG

*P. leopardus* ACAAATGAACCCTTCTCTTCGCTTCCCTAATACTATTTCTTATTTCCATTAATTTACTAG

*P. areolatus* ACAAATGAGCCCTTATTTTGACTTCGCTGATAATCTCCCTTCTTTCCATAAATCTACTAG

*E. radiosum* ATAAGTGAGCCGCCCTCTTGGCATCTTTAATAATTTTTTTAATTACCCTAAACATACTCG

*P. sieboldi* ACAAATGGGCGGTTATTCTCACCTCGTTAATACTTTTCCTCATCTCCCTCAACATGCTTG

*E. armatus* ACAAATGAGCCCTCCTATTTGGCTCCCTTATAATTTACCTTATTTCCCTTAATATACTAG

*R. oxyrhynchus* ACAAATGAGCTATGCTATTCGCCTCCCTAATATTATACCTTATTTCCCTAAACATGCTAG

*K. cinerascens* ACAAATGAGCCGTTCTTTTGACCTCACTCATGTTATTCCTCATCTCCCTCAACATGCTAG

*T. chatareus* ATAAATGAGCAACGCTCCTCGCCTCACTAATAGTTTTCCTCCTTTCACTGAATATGCTCG

*D. berycoides* ATAAATGAGCTGTTCTACTTACCTCTTTAATGCTATTCCTCATCACTCTTAATATGCTTG

*H. typus* ATAAATGAGCCGTACTACTAACCTCTCTTATACTATTCCTAATCACCCTAAACATGCTAG

*M. argenteus* ACAAATGAGCCCTGCTATTTAGCTCGCTAATACTATTCCTCATCACCCTCAATATGCTCG

*S. chuatsi* ATAAATGAGCTGCATTATTTACCTCCCTAATATTATTCCTAATCACTCTAAACATGTTAG

*O. fasciatus* ATAAATGAGCCATCTTATTGACCTCTTTAATACTATTTCTTATTTCCCTTAACATGCTTG

*P. trilineatum* ACAAATGGGCCCTTATTCTAACATCCCTTATACTATTCCTTATCACCATTAATATGTTAG

*M. salmoides* ATAAATGGGCCATCCTATTTACATCCCTAATATTATTCCTCATCACCCTTAATATACTTG

*P. tile* ACAAATGAGCCCTGATCCTGACATCCCTAATACTCTTCCTTATCACCCTCAATATGCTAG

*L. argentimaculatus* ACAAATGAGCCCTACTCCTAACATCGCTTATACTATTCCTTATTACCCTTAACATGCTCG

*E. struhsakeri* ACAAATGAGCCCTTATCCTGACTTCGCTTATGCTTTTCCTCATTACCCTTAATATGCTTG

*B. albus* ACAAATGAGCTCTCCTCCTCACCTCATTAATAGTATTTCTCTTAACCCTCAACTTACTAG

*C. auripes* ATAAGTGAGCCCTTATCTTTTGCTCACTTATAGTATTTCTAATTTCTCTTAATTCACTTG

*C. melampygus* ATAAATGAGCAACTCTGCTCACTTCCTTAATAATCTTTTTAATTACACTTAACATACTAG

*L. calcarifer* ACAAATGAGCCGCCCTATTGGCCTCCTTAATAATTTTCCTGCTATCACTAAATATACTAG

*S. maena* ATAAATGAGCTTTAATGCTCTCATCACTTATGATCTTTCTCCTTAGCCTTAATCTGCTCG

*P. auriga* ACAAATGAGCTTTAATTTTAACTTCCCTAATGGTCTTCCTGCTCGGCCTTAATCTACTCG

*C. lucidus* ATAATTGAGCTTTAATACTCGCTTCCCTAATACTCTTCCTTATTACACTTAATATTCTAG

*S. sihama* ACAAGTGAGCCCTGCTTTTTGCATCGCTAATAATCTTCCTCCTGTCCATAAATTTACTAG

*C. loricula* ACAAATGAGCCCTCCTCCTAGCCTCTCTCATGATCTTCCTCATTTCCCTAAACATGCTGG

*A. trutta* ATAAATGAACAGTACTTTTAATATCACTTCTTATATTTTTAATTACTCTAAACATGTTAG

*H. gemma* ATAAATGGGCTACCCTTCTAACATCTTTAATAATCTTTTTAATTACCTTAAATATGCTGG

*P. flavescens* ACAAATGAGCCGCGCTCTTAACTTCCTTAATAATCTTTTTAATTACCATCAACATGCTAG

*P. macrolepida* ACAAATGAGCCGCCCTCTTAGCCTCACTAATAATTTTTTTAATTACCTTAAATATGCTTG

*S. canadensis* ATAAATGGGCCGCCCTCTTAACTTCCTTAATAATTTTTCTAATTACCCTTAATATACTGG

*A. rogaa* GACTACTTCCCTACACCTTTACTCCCACTGCCCAGCTGTCCCTAAACCTAGGATTTGCAA

*C. argus* GCCTTCTTCCCTATACCTTCACCCCTACTGCCCAACTATCCCTTAATCTGGGTTTCGCAG

*C. sonnerati* GCCTTCTCCCATACACCTTCACCCCAACTGCTCAACTATCCCTTAACCTAGGATTTGCAG

*E. fuscoguttatus* GACTTCTACCCTATACCTTCACCCCCACTGCCCAACTATCCCTTAACTTAGGGTTTGCAG

*E. coioides* GACTCCTGCCCTACACTTTTACCCCTACCGCCCAACTATCCCTTAATCTAGGGTTTGCAG

*E. bruneus* GACTCCTGCCCTATACTTTCACCCCTACCGCCCAACTATCTCTTAACTTAGGGTTTGCAG

*E. moara* GACTCCTGCCCTATACTTTCACCCCAACCGCCCAACTATCTCTTAACTTAGGATTTGCAG

*E. lanceolatus*  GACTTCTACCCTACACTTTTACCCCTACTGCCCAACTATCCCTTAACTTAGGATTTGCAG

*A. leucogrammicus*  GACTCCTACCCTACACTTTTACCCCTACTGCCCAACTATCCCTTAACCTAGGCTTTGCAG

*C. altivelis* GACTCCTGCCCTACACTTTTACCCCTACTGCCCAACTATCCCTTAACCTAGGATTCGCAG

*E. epistictus* GGCTTCTACCCTATACTTTCACCCCTACCGCTCAACTATCCCTTAACCTAGGATTTGCAG

*E. octofasciatus* GGCTCTTGCCTTACACTTTTACCCCAACTGCCCAGCTATCCCTTAACCTAGGCTTCGCCG

*E. septemfasciatus* GGCTTTTGCCTTACACTTTTACCCCAACTGCCCAACTATCCCTTAACCTAGGCTTCGCCG

*T. dermopterus* GGCTTCTACCCTACACTTTCACCCCAACTGCCCAACTATCCCTTAACCTGGGCCTCGCCG

*E. awoara* GGCTCTTACCCTATACTTTCACACCTACCGCCCAATTGTCCCTCAACCTAGGACTTGCTG

*E. akaara* GACTCTTACCCTACACTTTCACCCCTACCGCCCAATTGTCCCTCAACCTGGGATTTGCAG

*E. trimaculatus* GACTTCTACCCTACACCTTTACCCCCACTGCCCAGCTCTCCCTTAACCTGGGATTCGCGG

*E. areolatus* GATTACTACCCTACACTTTCACTCCTACTGCCCAGTTATCTCTTAACCTAGGATTTGCAG

*V. albimarginata* GACTTCTCCCATATACCTTTACACCTACAGCCCAGCTATCCCTTAACCTTGGTTTTGCCA

*V. louti* GACTCCTCCCATACACCTTTACACCTACAGCCCAGCTATCACTTAACCTTGGTTTTGCTA

*P. leopardus* GCCTTCTTCCATATACCCACACGCCCACTGGTCAGCTATCCCTAAACCTGGGATTCGCTG

*P. areolatus* GACTTCTTCCCTATACCTACACACCCACTGCTCAGCTATCCCTAAACCTGGGGTTCGCTA

*E. radiosum* GCCTTCTCCCTTATACCTTTACACCCACCACACAATTATCCCTTAACCTAGGACTAGCAG

*P. sieboldi* GCCTCCTTCCATACACCTTTACACCCACAACCCAACTGTCCTTAAATATGGGCTTTGCAG

*E. armatus* GCCTTCTTCCATATACCTTCACCCCAACAACACAACTGTCGTTAAACCTAGGCCTTGCAG

*R. oxyrhynchus* GCCTCCTCCCATATACATTTACACCTACCACCCAACTATCTCTAAACCTAGGGCTCGCAG

*K. cinerascens* GCCTCCTCCCATACACCTTTACCCCTACAACACAGCTATCCCTCAACATGGGCCTCGCAG

*T. chatareus* GCCTCTTACCCTATACCTTTACCCCAACCACCCAACTGTCCCTAAATATAGGACTTGCAG

*D. berycoides* GTCTGCTTCCCTACTCCTTTACACCCACTACACAACTGTCCCTTAATTTAGGCCTTGCGG

*H. typus* GCCTTCTTCCATATACTTTTACCCCTACCACGCAGCTATCTTTAAACATAGGCCTCGCAG

*M. argenteus* GCCTCCTCCCCTACACCTTTACCCCTACCACCCAACTATCCCTCAACATGGGCCTTGCAG

*S. chuatsi* GACTTCTGCCTTACACATTTACCCCTACCACACAATTGTCCCTCAATATAGGCCTTGCAG

*O. fasciatus* GCCTTCTCCCATATACCTTCACCCCCACAACACAGCTATCCCTCAACATAGGCCTTGCAG

*P. trilineatum* GACTTCTTCCCTATACCTTCACTCCCACAACTCAATTGTCCCTTAATATAGGTCTTGCAG

*M. salmoides* GCCTCCTCCCGTACACTTTTACACCCACCACTCAACTATCCCTCAACATAGGCCTTGCAG

*P. tile* GGCTGCTCCCATACACATTTACCCCAACTACACAACTGTCCCTCAACATGGGCCTTGCAG

*L. argentimaculatus* GACTACTCCCTTACACATTTACCCCAACCACACAATTATCCCTCAACATAGGACTTGCTG

*E. struhsakeri* GACTCCTTCCCTACACCTTTACCCCAACCACACAATTGTCCCTCAACATGGGCCTTGCAG

*B. albus* GCCTTCTTCCATACACATTTACACCCACTACGCAACTATCACTCAACCTCGGCTTCGCCG

*C. auripes* GGCTCCTACCTTACACATTCACCCCAACCACCCAACTCGCCCTTAATATAGGCTTTGCAG

*C. melampygus* GACTTCTCCCTTACACCTTCACGCCTACTACGCAACTTTCTCTCAATATGGGACTAGCAG

*L. calcarifer* GCCTCCTTCCATACACCTTCACCCCCACCACACAACTATCTCTCAACCTAGGTCTCGCAG

*S. maena* GCCTCCTCCCCTATACCTTTACCCCTACTACCCAACTTTCGATTAACCTAGGCTTTGCAG

*P. auriga* GCCTCCTCCCCTACACCTTTACCCCCACTACCCAACTATCTATTAATCTGGGCTTTGCTG

*C. lucidus* GCCTCCTCCCCTACACCTTCACCCCCACAACTCAACTATCCCTCAACCTGGCCCTTGCCT

*S. sihama* GGCTTCTACCATACACTTTTACGCCCACAACCCAACTGTCATTAAACATAGGCTTAGCAG

*C. loricula* GCCTTCTCCCCTATACTTTCACGCCCACTACACAGTTATCGCTTAATCTCGCCCTTGCAG

*A. trutta* GCCTCCTTCCGTACACCTTTACCCCTACCACCCAACTCTCCCTCAACCTAGGCCTGGCGT

*H. gemma* GGCTCCTTCCCTATACCTTCACACCTACTACCCAGCTTTCACTTAATCTTGGACTTGCAA

*P. flavescens* GCCTCCTCCCTTATACTTTTACCCCTACTACTCAGCTGTCCCTTAATTTAGGACTCGCAG

*P. macrolepida* GCCTGCTACCTTATACCTTTACCCCCACCACGCAGCTATCCCTTAATTTAGGGCTCGCAG

*S. canadensis* GACTCCTTCCTTATACCTTTACACCTACCACCCAATTATCCCTCAATTTAGGACTTGCAG

*A. rogaa* TTCCTCTATGAATAGCAACAGTAATCATTGGTATACGAAACCAACCAAACCACGCCTTAG

*C. argus* TTCCCCTGTGAATGGCAACAGTAATTATTGGGCTACGAAACCAACCAAACCACGCCTTAG

*C. sonnerati* TCCCCCTTTGAATAGCTACAGTAATTATTGGTATGCGAAACCAACCAAATCATGCATTAG

*E. fuscoguttatus* TCCCTCTCTGATTAGCCACTGTTATTATCGGAATACGAAACCAACCTAATCATGCACTAG

*E. coioides* TCCCCCTCTGACTGGCCACTGTCATCATTGGATTACGAAACCAGCCAAATCACGCACTAG

*E. bruneus* TCCCCCTCTGATTAGCTACTGTTATTATTGGAATACGAAACCAACCAAATCACGCACTAG

*E. moara* TCCCCCTCTGATTAGCTACTGTTATCATTGGAATACGAAACCAACCAAATCACGCACTAG

*E. lanceolatus*  TCCCCCTTTGATTAGCCACTGTTATTATTGGAATACGAAACCAACCAGACCACGCACTAG

*A. leucogrammicus*  TCCCCCTCTGACTGGCCACTGTTATTATTGGCATGCGAAACCAACCAAACCACGCACTAG

*C. altivelis* TCCCTCTTTGACTAGCCACTGTAATTATTGGGATACGAAACCAACCAAATCACGCACTAG

*E. epistictus* TCCCCCTCTGATTGGCAACCCTCATTATTGGAATACGAAACCAACCAAATCACGCCCTAG

*E. octofasciatus* TCCCCCTTTGATTGGCAACTGTCATTATTGGAATACGAAACCAACCAAATCACACTCTGG

*E. septemfasciatus* TCCCCCTTTGATTGGCAACTGTCATTATTGGAATGCGAAACCAACCAAATCATGCTCTAG

*T. dermopterus* TCCCTCTCTGATTGGCAACTGTCATTATTGGGATACGAAACCAGCCAAACCAGGCCTTAG

*E. awoara* TACCCCTCTGACTAGCAACCCTTATCATCGGAATGCGAAACCAACCAAACCACGCCCTAG

*E. akaara* TCCCCCTTTGATTGGCAACCCTTATTATTGGAATACGAAACCAACCAAACCATGCCCTAG

*E. trimaculatus* TCCCCCTTTGACTAGCAACCGTTATTATCGGAATGCGAAACCAACCAAACCACGCCCTAG

*E. areolatus* TCCCTCTCTGATTAGCAACCGTAATTATTGGTATGCGGAACCAACCAAACCATGCACTAG

*V. albimarginata* TCCCCCTTTGAATAGCAACAGTAATTATTGGTATACGCTACCAACCAAATCACGCTCTAG

*V. louti* TCCCTCTTTGAATGGCAACAGTAATTATTGGGATACGCTACCAACCAAACCACGCACTAG

*P. leopardus* TCCCTCTTTGATTAACCACAGTAATTATTGGGTTCCGATACCAGTTTAACCATGCCCTTG

*P. areolatus* TCCCTCTTTGAATAGCCACAGTAATTATTGGTTTCCGATACCAACTAAACCATGCCCTTG

*E. radiosum* TGCCTCTCTGACTAGCAACCGTAATTATTGGGATACGAAACCAGCCCACCCATGCTCTAG

*P. sieboldi* TCCCCCTCTGACTAGCTACCGTTCTAATTGGCATGCGGAATCAACCAACCGTGGCGCTAG

*E. armatus* TTCCCCTCTGACTAGCCACAGTACTTATTGGAATACGAAATCAACCAACTATTGCCTTAG

*R. oxyrhynchus* TTCCACTCTGACTGGCAACCGTAATTATTGGTATGCGAAACCAACCAACTATTGCCCTCG

*K. cinerascens* TACCCCTTTGACTAGCCACAGTAATTATTGGATTACGAAATCAACCAACCATTGCCCTAG

*T. chatareus* TCCCTCTCTGACTGGCAACAGTAATTATTGGGATACGAAACCAACCAACCCATGCCCTAG

*D. berycoides* CCCCTCTTTGATTAGCAACAGTTCTTATTGGGGTGCGAGGCCAACTAGTCTACTCTCTAG

*H. typus* TTCCCCTCTGAATAGCGACCGTCGTCATTGGGTTTCGCAATCAACCAACCATTGCACTAG

*M. argenteus* TACCTCTCTGGTTAGCAACGGTAATTATTGGTATGCGCAATCAGCCAACCGTCGCGCTAG

*S. chuatsi* TTCCACTCTGGCTAGCAACCGTTATTATTGGTATACGTAACCAACCAACTATCGCACTTG

*O. fasciatus* TACCACTCTGACTGGCAACAGTACTCATCGGCCTCCGAAACCAGCCAACTATTGCCCTCG

*P. trilineatum* TACCTCTCTGATTAGCCACAGTCATCATTGGTCTCCGGAACCAACCGACTGTCGCCCTAG

*M. salmoides* TTCCTCTTTGGTTGGCAACCGTAATTATTGGTATGCGAAATCAACCAACCATTGCCCTAG

*P. tile* TTCCCCTTTGACTGGCAACAGTAATTATTGGAATGCGAAACCAGCCAACCATTGCACTAG

*L. argentimaculatus* TCCCCCTCTGATTGGCCACAGTCATCATCGGGATACGAAACCAACCAACCATTGCGCTAG

*E. struhsakeri* TCCCCCTTTGACTGGCAACAGTCATTATTGGAATGCGAAACCAACCAACAATTGCCCTAG

*B. albus* TTCCCCTCTGACTGGCGACAGTAATCATCGGCATATGAAATAAGCCTACTGATGCGCTAG

*C. auripes* TCCCCTTTTGACTGGCTACAGTCTTAACAGGCCTGCGAAATCAGCCGAATGTTGCCATTG

*C. melampygus* TACCTCTCTGACTCGCCACTGTTATTATTGGAATACGAAACCAACCCACCCATGCTCTCG

*L. calcarifer* TCCCCCTGTGACTCGCAACAGTAATTATTGGAATGCGAAACCGACCAACCCACGCCCTAG

*S. maena* TTCCCCTATGGTTAGCGACAGTACTAATTGGAATACGAAACCAGCTTAACCACTCTCTAG

*P. auriga* TCCCCCTCTGGTTGGCTACAGTTCTTATTGGATTCCGGTACCAGCCAAACTTTTCTTTAG

*C. lucidus* TCCCCCTTTGACTAGCCACAGTTCTTATAGGACTACGTAATCAACCAACCGCTGCCCTAG

*S. sihama* TCCCATTGTGACTTGCCACCGTAATTATTGGATTGCGAACACAACTGACCGTCTCACTGG

*C. loricula* TACCACTATGACTGGCAACTGTAGCTATCGGAATGCGAAACCAGCCAACAGCAGCTTTAG

*A. trutta* TCCCTCTATGACTTGCAACAGTCATTATTGGCATGCGAAACCAACCAACTGAAGCCCTTG

*H. gemma* CCCCCCTATGATTAGCAACCGTCCTAATTGGGATGCGTAATCAGCCCACTCACGCGTTAG

*P. flavescens* TACCCCTTTGACTGGCCACAGTCCTTATTGGAATACGAAACCAGCCGACACATGCCCTCG

*P. macrolepida* TCCCCCTTTGATTAGCAACAGTAATTATTGGTATGCGAAACCAACCAACCCACGCCCTCG

*S. canadensis* TACCACTCTGGCTAGCAACAGTCCTTATTGGGATACGAAATCAACCTACACATGCCCTTG

*A. rogaa* GTCACCTCCTACCAGAAGGAACCCCTAACCTCCTAATTCCTATACTGATTATTATCGAAA

*C. argus* GACACCTCCTGCCAGAAGGGACCCCTAACCTTCTAATTCCTATACTGATTATTATCGAAA

*C. sonnerati* GTCACCTCCTACCAGAAGGTACCCCAAACCTCCTAATTCCAATACTAATCGTTATCGAAA

*E. fuscoguttatus* GACACCTTTTACCAGAAGGCACCCCTAACCTACTAATCCCGATACTCATTATCATCGAAA

*E. coioides* GTCATCTTTTACCAGAGGGCACCCCCAACCTCCTAATCCCTATACTTATTGTCATCGAAA

*E. bruneus* GCCACCTTCTACCAGAAGGCACCCCTACTCTCCTAATCCCAATACTCATTGTTATCGAAA

*E. moara* GTCACCTTCTACCAGAAGGCACCCCTAATCTCCTAATCCCTATACTCATTGTCATCGAAA

*E. lanceolatus*  GACACCTCCTACCAGAAGGCACCCCTAACCTCCTAATCCCTATACTCATTATTATCGAAA

*A. leucogrammicus*  GACACCTCCTACCAGAAGGTACCCCTAACCTTCTAATCCCAATACTTATCATCATCGAAA

*C. altivelis* GACACCTTCTTCCAGAAGGCACCCCTAACCTCCTAATCCCTATACTCATCATCATCGAAA

*E. epistictus* GACACCTCCTACCAGAAGGAACCCCCAACCTCCTAATTCCAATGCTTATTGTTATCGAAA

*E. octofasciatus* GTCACCTTCTACCAGAAGGTACCCCTAACCTCCTAATTCCCATACTTATTATTATCGAAA

*E. septemfasciatus* GACACCTTCTACCAGAAGGAACCCCTAGTCTCCTAATCCCAATACTAATTATTATCGAAA

*T. dermopterus* GACACCTCCTACCAGAAGGGACCCCCAACCTTCTAATCCCAATGCTTATTATCATTGAAA

*E. awoara* GACATCTTCTACCAGAAGGGACTCCCAATCTCCTAATCCCTATACTTATCGTCATTGAAA

*E. akaara* GACATCTTCTACCAGAAGGGACGCCCAATCTCCTAATCCCTATACTTATCGTTATTGAAA

*E. trimaculatus* GTCATCTTCTACCAGAAGGGACACCAAATCTTTTAATTCCTATACTAATTATTATCGAAA

*E. areolatus* GCCACCTCCTGCCAGAAGGAACCCCCAACCTCCTGATTCCTATACTTATTATCATCGAAA

*V. albimarginata* GACACCTTCTTCCAGAAGGTACACCTAACCTACTCATTCCTATTCTAATTATTATCGAAA

*V. louti* GACACCTTCTTCCAGAAGGTACACCCAACCTGCTTATCCCTATCCTAATTATTATCGAAA

*P. leopardus* CACACTTCCTCCCAGAAGGAACCCCTAACCTCTTGATTCCTGTTTTAATTATTATCGAAA

*P. areolatus* GACACTTGCTCCCAGAAGGAACCCCTAGCCTTTTAATTCCTATTTTAATTATTATCGAAA

*E. radiosum* GCCATCTCCTACCAGAGGGCACCCCTGGACCTCTCATCCCTGTTCTTATTGTTATCGAGA

*P. sieboldi* GCCATCTTCTCCCTGAGGGAACCCCAACTCCACTAATCCCCGTCCTAATCATTATTGAAA

*E. armatus* GACACCTCCTTCCAGAAGGTACCCCCACACCTCTAATTCCAGTCCTTATTATTATCGAAA

*R. oxyrhynchus* GACATCTCCTCCCAGAAGGTACTCCTACTTTACTTATCCCCATCCTCATTATCATCGAAA

*K. cinerascens* GCCACCTCCTTCCAGAAGGGACCCCCACCCCCCTGATCCCAGTACTAATCATCATCGAAA

*T. chatareus* GTCACCTTCTACCAGAAGGAACTCCTACCCTTCTGATCCCCGTACTAATCATTATCGAGA

*D. berycoides* CCCACCTTGTACCAGAAGGCACTCCCACGCTTCTAATTCCAGTACTAGTTATTATTGAAA

*H. typus* GACATCTTCTGCCAGAAGGAACCCCGACCCCTCTGATCCCCGTCCTGATTATTATCGAAA

*M. argenteus* GCCATCTCCTACCAGAAGGAACCCCTACCCCCTTAATTCCCGTTCTTATCATCATCGAAA

*S. chuatsi* GCCACCTTCTCCCTGAAGGTACCCCCACCCCCCTGATTCCCGTCCTGATCATTATCGAAA

*O. fasciatus* GACACCTCCTTCCAGAAGGTACTCCCACCCCACTAATCCCCGTGCTAATTATTATCGAAA

*P. trilineatum* GACACCTTCTTCCAGAAGGAACCCCTGCCCCTCTGATCCCAGTTCTAATTATTATCGAAA

*M. salmoides* GACACCTTCTTCCAGAAGGTACCCCCACCCTCCTAATCCCTGTTCTAATTATTATCGAAA

*P. tile* GCCATCTTCTGCCAGAAGGAACCCCTACCCTTCTGATTCCAGTCCTCATCATTATCGAAA

*L. argentimaculatus* GTCACCTTCTGCCAGAAGGGACTCCCACACTCCTAATCCCCGTTCTTATTATCATCGAAA

*E. struhsakeri* GACATCTCCTGCCGGAAGGAACACCCACCCTTCTGATTCCCGTCTTAATTATTATCGAAA

*B. albus* GACACCTCCTACCAGAAGGTACCCCCGCGCCTCTGATCCCCGTCCTAATTATCATCGAGA

*C. auripes* CCCACATTCTGCCTGAAGCCACCCCTGGACCCCTGGTCCCAGTGTTGATTATAATTGAAA

*C. melampygus* GTCATCTCCTTCCCGAAGGAACCCCAACCCTCCTAATCCCCGTCCTTATTATCATCGAAA

*L. calcarifer* GCCACCTTCTCCCAGAGGGAACCCCCTCCCTGCTAATTCCAATCCTAATTATCATCGAAA

*S. maena* CACACTTACTGCCAGAAGGAACCCCTCTGCTGCTAATTCCAATTCTGATCATTATTGAAA

*P. auriga* CCCATCTTCTACCAGAAGGAACCCCCCTACTCCTTATTCCTGTCCTAATTATTATCGAAA

*C. lucidus* GACACCTATTACCAGAAGGCACACCCACACCCCTAATCCCCATCCTAATTATCATCGAAA

*S. sihama* CCCATCTCCTGCCAGAAGGCACTCCGACAGCCCTGGTCCCCGTCTTAATCGTGATCGAAA

*C. loricula* GACACCTTCTTCCAGAAGGAACACCTACTGCCCTAATCCCTATTCTCGTAGTCATCGAAA

*A. trutta* GCCACCTTCTTCCAGAAGGCACCCCCACCCTTCTCATCCCTGTTCTAATTATTATTGAGA

*H. gemma* GCCACCTTCTTCCAGAAGGAACCCCCGGCCCTCTAATTCCTGTTCTGATTGTCATCGAAA

*P. flavescens* GACACCTTCTCCCTGAAGGAACCCCTGGCCCTCTAATCCCTGTTCTCATTATTATCGAAA

*P. macrolepida* GACATCTTCTACCAGAAGGAACCCCCGGGCCTCTCATTCCTGTCCTTATTGTCATCGAAA

*S. canadensis* GACATCTTCTTCCTGAAGGTACCCCTGGTCCTCTTATCCCTGTTCTCATCATCATCGAGA

*A. rogaa* CAATCAGCCTGTTTATCCGACCCCTGGCCCTAGGCGTTCGACTTACCGCCAACTTAACAG

*C. argus* CAATCAGCCTATTTATTCGACCACTTGCCCTGGGGGTTCGACTCACTGCCAACTTGACAG

*C. sonnerati* CAATTAGCCTATTCATCCGACCCCTTGCTCTAGGAGTCCGACTTACTGCCAACTTAACAG

*E. fuscoguttatus* CAATTAGCCTATTCATCCGCCCCTTAGCTCTAGGCGTTCGACTTACCGCCAACCTAACAG

*E. coioides* CAATCAGCCTATTTATCCGCCCCCTAGCCTTAGGTGTTCGACTAACCGCTAACCTAACAG

*E. bruneus* CAATCAGCCTATTTATCCGCCCCTTGGCCTTAGGTGTACGACTAACTGCTAACCTAACAG

*E. moara* CAATCAGCCTATTTATCCGCCCCCTGGCCTTAGGTGTACGACTAACTGCTAACCTAACAG

*E. lanceolatus*  CAATCAGCCTATTCATCCGCCCCTTAGCCTTAGGCGTACGGCTAACCGCCAACCTAACAG

*A. leucogrammicus*  CAATCAGCCTGTTCATCCGTCCCCTTGCTCTAGGCGTTCGACTAACCGCCAACCTAACAG

*C. altivelis* CAATTAGCCTATTCATCCGCCCATTAGCCCTAGGCGTACGACTTACCGCCAATCTGACAG

*E. epistictus* CAATTAGCCTATTTATTCGACCATTAGCACTAGGTGTACGACTAACCGCTAACCTGACAG

*E. octofasciatus* CAATTAGCCTATTCATCCGACCCCTGGCCCTGGGTGTACGACTAACCGCTAACCTAACGG

*E. septemfasciatus* CAATTAGCCTATTCATCCGGCCCCTGGCCCTGGGTGTACGACTAACCGCTAACCTAACAG

*T. dermopterus* CAATTAGCCTATTCATCCGACCCCTCGCCCTGGGCGTACGATTAACCGCCAACCTAACAG

*E. awoara* CAATTAGCCTATTTATCCGACCTTTGGCGTTAGGAGTTCGACTAACTGCCAACCTAACAG

*E. akaara* CAATTAGCCTATTCATCCGACCCCTAGCCCTAGGCGTTCGGCTGACTGCTAATCTAACAG

*E. trimaculatus* CAATTAGCCTCTTTATCCGACCCCTGGCGCTAGGTGTGCGGTTAACTGCCAACCTAACAG

*E. areolatus* CAATTAGCCTCTTCATCCGACCTTTAGCACTAGGCGTACGGCTAACTGCCAACCTGACAG

*V. albimarginata* CAATTAGCTTATTCATTCGACCCTTAGCTCTAGGAGTCCGATTAGCAGCAAACCTAACAG

*V. louti* CAATTAGCTTATTTATTCGACCCTTGGCTCTAGGCGTCCGATTAGCAGCAAACCTAACAG

*P. leopardus* CTATTAGCCTTTTCATTCGCCCCCTTGCACTAGGAGTACGACTAGCAGCTAACTTAACAG

*P. areolatus* CTATTAGCCTCTTCATTCGCCCCCTCGCACTTGGAGTACGATTAGCAGCTAACCTAACAG

*E. radiosum* CAATTAGTCTACTCATTCGCCCTCTTGCCCTGGGAGTCCGACTTACAGCTAATCTTACTG

*P. sieboldi* CGATTAGTTTATTCATTCGACCAATTGCCCTGGGCGTCCGACTCACGGCCAATCTTACGG

*E. armatus* CAATTAGCCTCTTCATTCGCCCTCTCGCCCTTGGCGTTCGGTTAACAGCTAATCTCACAG

*R. oxyrhynchus* CAATCAGCTTATTTATTCGGCCTGTTGCACTAGGAGTGCGATTAACAGCCAACCTTACAG

*K. cinerascens* CAATTAGCCTTTTCATTCGCCCCCTTGCGCTCGGAGTCCGGCTGACAGCCAACCTCACAG

*T. chatareus* CAATCAGCCTCTTTATTCGCCCACTAGCTCTAGGGGTACGACTAACAGCTAACCTTACAG

*D. berycoides* CAATTAGTCTACTTATTCGACCCCTTGCCCTGGGGGTTCGACTAACAGCTAACCTCACAG

*H. typus* CAATTAGCCTGTTCATTCGCCCTCTGGCCCTAGGCGTACGACTAACAGCCAACCTTACAG

*M. argenteus* CAATTAGTCTATTTATCCGCCCTCTTGCCCTAGGGGTTCGACTTACCGCTAATCTTACCG

*S. chuatsi* CAATTAGTCTATTTATTCGCCCTCTAGCCCTAGGAGTACGGCTAACAGCAAACCTAACAG

*O. fasciatus* CAATTAGCCTTTTCATCCGTCCTCTTGCGTTAGGCGTACGACTAACAGCTAACCTCACAG

*P. trilineatum* CAATTAGCTTATTTATTCGACCCCTAGCTCTAGGTGTTCGATTAACAGCCAACCTCACGG

*M. salmoides* CAATTAGCCTATTTATTCGCCCCCTAGCCTTGGGCGTGCGACTGACAGCAAATTTGACAG

*P. tile* CAATCAGCCTGTTTATTCGTCCCCTAGCCCTAGGAGTTCGACTTACAGCCAACCTCACAG

*L. argentimaculatus* CAATTAGCCTATTTATCCGCCCCCTAGCGCTAGGAGTTCGATTAACAGCCAACCTCACAG

*E. struhsakeri* CAATTAGCCTATTTATTCGTCCCTTAGCCCTAGGAGTACGGCTAACAGCTAATCTTACCG

*B. albus* CAATTAGCCTATTTATTCGGCCCCTCGCCCTAGGAGTACGGCTCACTGCCAACCTCACAG

*C. auripes* CCGCTAGCTTATTCATCCGCCCGCTAGCACTAGGAGTACGACTAACCGCTAATCTTACCG

*C. melampygus* CAATTAGCCTATTCATCCGCCCCCTTGCCCTAGGCGTTCGACTTACAGCCAACCTCACAG

*L. calcarifer* CAATCAGTCTCCTCATCCGTCCAATTGCTCTCGGAGTCCGACTAACAGCCAATCTCACAG

*S. maena* CAATTAGCTTAATAATTCGGCCCCTCGCCTTAGGAGTACGGCTTACAGCTAACCTTACTG

*P. auriga* CAATTAGCTTAATGATTCGCCCTCTTGCCCTTGGAGTACGACTTACAGCAAACCTAACCG

*C. lucidus* CAATCAGCTTGCTTATTCGCCCCCTAGCCCTGGGTGTCCGACTGACTGCCAACCTCACAG

*S. sihama* CGATTAGCCTCTTTATTCGCCCCCTTGCCCTGGGCGTACGGCTTACTGCTAACCTCACAG

*C. loricula* CAATTAGCCTATTTATCCGACCCGTTGCCCTAGGAGTTCGACTCACGGCGAACCTAACAG

*A. trutta* CGGCCAGCCTTCTCATTCGCCCCCTAGCACTTGGTGTGCGACTCACCGCTAACCTCACAG

*H. gemma* CAATTAGTCTCTTTATTCGCCCATTGGCCCTGGGAGTTCGACTCACAGCCAATCTAACAG

*P. flavescens* CAATTAGCCTATTCATTCGCCCTCTTGCTTTAGGAGTCCGACTTACGGCTAACTTAACTG

*P. macrolepida* CAATTAGTCTATTTATTCGACCCCTCGCTCTAGGAGTCCGGCTAACAGCAAATCTCACCG

*S. canadensis* CAATTAGCCTATTTATTCGCCCCCTTGCCCTAGGAGTTCGACTTACAGCCAACCTTACAG

*A. rogaa* CAGGTCACCTACTAATTCAATTAATTTCGACAGCCGCATTTGTTCTTCTACCCTTAATAC

*C. argus* CAGGTCACTTACTAATCCAACTAATCTCAACAGCTGCATTCGTCCTTCTGCCCTTAATAC

*C. sonnerati* CCGGTCATCTCTTAATTCAGTTAATTTCAACAGCTGCCTTTGTTCTCCTACCAATAATGC

*E. fuscoguttatus* CTGGCCACCTACTCATTCAACTAATCTCCACAGCTGCATTTGTGCTCCTACCACTTATGC

*E. coioides* CTGGTCACTTACTTATTCAATTAATCTCCACAGCTGCCTTTGTACTCCTACCACTTATGC

*E. bruneus* CTGGCCACCTGCTCATTCAATTAATCTCCACAGCTGTATTTGTACTCCTACCACTCATAC

*E. moara* CAGGCCACCTGCTCATTCAATTAATCTCTACAGCTGTATTTGTACTCCTACCACTCATAC

*E. lanceolatus*  CTGGTCACTTACTTATTCAACTAATCTCCACAGCCGCATTTGTACTTCTTCCACTCATAC

*A. leucogrammicus*  CCGGCCACTTACTTATTCAACTAATTTCCACGGCCGCATTCGTACTCCTGCCACTTATAC

*C. altivelis* CTGGCCATCTGCTCATTCAATTAATTTCTACAGCTGCATTTGTGCTCCTACCGCTTATAC

*E. epistictus* CTGGACATTTACTCATCCAATTAATCTCCACAGCCGTATTTGTACTCCTGCCCCTTATGC

*E. octofasciatus* CTGGTCACCTACTTATTCAATTAATCTCAACAGCTGCCTTTGTGCTTCTACCACTTATAC

*E. septemfasciatus* CTGGTCACCTGCTTATTCAACTAATCTCAACAGCTGCCCTTGTGCTTCTACCACTTTTAC

*T. dermopterus* CCGGCCATCTACTTATTCAATTAATCTCAACGGCAGCCTTCGTACTCCTGCCACTAATAC

*E. awoara* CCGGCCATTTACTCATTCAACTAATCTCTACAGCTGTATTTGTTCTTCTACCACTTATAC

*E. akaara* CTGGTCATTTACTCATTCAACTAATCTCCACAGCTGTATTTGTTCTCCTACCACTTATAC

*E. trimaculatus* CTGGTCACCTGCTCATTCAACTGATTTCCACAGCTGCATTTGTATTACTACCTCTCATGC

*E. areolatus* CTGGCCACTTGCTTATTCAGCTAATTTCCACAGCCGCATTTGTTCTTCTACCACTTATGC

*V. albimarginata* CTGGCCATCTATTGATTCAGCTCATCTCCACAGCTGCCTTTGTCCTAATACCTATGATGC

*V. louti* CTGGCCATCTATTAATTCAACTAATTTCCACAGCCGCCTTCGTCCTTATGCCCATGATAC

*P. leopardus* CCGGACATTTACTTATTCAATTAATTTCCACAGCTGTCTTTGTTCTCTTACCATTATTAC

*P. areolatus* CTGGACATTTACTCATCCAACTAATCTCCACAGCTGTCTTTGTCCTCTTGCCATTAATAC

*E. radiosum* CAGGCCACCTTTTGATTCAACTTATTGCCACGGCCGCGTTTGTTTTACTACCCCTAATGC

*P. sieboldi* CAGGCCACTTACTTATCCAGCTAATTGCAACCGCTGCCTTTGTACTAGCTCCAATGATAC

*E. armatus* CAGGTCACTTACTGATTCAACTAATCGCAACCGCCGTATTTGTTCTTCTACCACTGATAC

*R. oxyrhynchus* CAGGCCACCTCCTAATTCAACTAATCGCCACGGCTGCTTTTGTACTCCTGCCGCTCATAC

*K. cinerascens* CCGGCCATCTCCTAATTCAACTAATCGCCACAGCCGCCTTTGTCCTCATGCCTTTAATAC

*T. chatareus* CCGGACATCTCCTTATTCAACTCATCGCCACAGCCGCCTTCGTCCTCCTCCCACTCATAC

*D. berycoides* CCGGCCACCTTTTAATCCAACTAATTTCTACAGCTACCTTCGTTCTCGTAACCACAATAC

*H. typus* CCGGACATCTTTTAATTCAACTAATCGCCACGGCTGCCTTTGTTCTCCTGCCCTTAATAC

*M. argenteus* CCGGACATCTCCTAATTCAACTAATTGCCACAGCTGCCTTCGTCCTTCTTCCCCTAATAC

*S. chuatsi* CCGGACATCTTCTGATTCAACTAATCGCGACAGCTGCATTTGTACTTCTTCCACTTATAC

*O. fasciatus* CCGGCCACCTCCTTATTCAACTAATTGCCACCGCTGCCTTCGTCCTCATACCTTTAATAC

*P. trilineatum* CCGGACATCTTTTAATTCAGCTAATCGCCACAGCCGCCTTCGTCCTCCTCCCACTAATGC

*M. salmoides* CCGGACATCTCCTTATTCAATTAATCGCTACTGCCGCCTTCGTACTCCTCCCCCTTATAC

*P. tile* CTGGCCACCTACTAATTCAGCTCATCGCTACAGCTGCCTTTGTCCTTCTCCCTCTAATGC

*L. argentimaculatus* CCGGCCATCTCCTAATTCAACTAATTGCCACAGCTGCTTTCGTCCTCCTCCCCCTAATAC

*E. struhsakeri* CCGGCCACCTCCTGATTCAACTAATTGCCACAGCTGCATTCGTATTATTACCCTTAATGC

*B. albus* CCGGCCACCTCCTAATTCAACTGATTTCAACTGCTGTCTTTGTTTTAGTACCCCTAATGC

*C. auripes* CCGGCCATCTTCTTATTCAACTAATTTCCACAGCCACATTTGTTCTTACCCCCTTAATAC

*C. melampygus* CTGGCCACCTACTTATTCAACTTATTGCCACAGCCGCATTTGTTCTCCTCCCTCTAATGC

*L. calcarifer* CAGGACACTTACTAATTCAACTCATCGCCACCGCTTCTTTTGTCCTCCTTCCACTAATAC

*S. maena* CTGGCCATCTTCTCATTCAACTTATTTCAACGGGACTTTTTGTTATACTTTCCCTACAGC

*P. auriga* CCGGCCATCTCCTTATTCAACTTATTTCAACAGGTATATTTGTCCTTCTCCCCCTGCAAC

*C. lucidus* CTGGTCATCTTTTAATACAACTAACATCTACAGCCGCTTACGTCCTTCTACCCATGATAC

*S. sihama* CTGGCCATCTCCTGATCCAGCTAATTTCCACGGCCGCCTTTGTCCTGCTACCTCTCATGC

*C. loricula* CCGGCCACCTTCTGATCCAACTAGTCTCAACAGCCGTTATGGTCCTTCTACCTATGATGC

*A. trutta* CTGGTCACCTTTTAATTCAACTAGTCGCGACTGCCACAATAGTACTCCTTCCCCTAATAC

*H. gemma* CCGGACATCTTTTAATCCAGCTTATCGCAACAGCTGCCTTTGTGCTTCTGCCTATGATAC

*P. flavescens* CAGGCCACCTTTTAATTCAACTCATTGCCACCGCCGCCTTTGTCCTTCTACCCCTAATAC

*P. macrolepida* CCGGCCACCTATTAATTCAACTCATTGCCACAGCTGCATTTGTTCTTTTACCTCTAATAC

*S. canadensis* CAGGCCACCTCTTAATTCAACTCATCGCCACCGCCGCCTTCGTTCTTCTACCCCTAATAC

*A. rogaa* CAACCGTGGCCATCCTTACATCAATCGTACTTGTTCTCCTAACACTCTTAGAAATTGCCG

*C. argus* CAGCCGTAGCTATGATTACATCAATTGTGCTTGTTCTCCTAACACTACTAGAAGTTGCTG

*C. sonnerati* CAACCGTAGCTATCATCACATCAGTAGTACTAATTCTCCTAACACTACTAGAAGTTGCCG

*E. fuscoguttatus* CAACTGTAGCTATTCTTACAGCAACAGTCTTAGTCCTTTTAACACTGCTAGAAGTTGCCG

*E. coioides* CAACCGTAGCTATTCTTACAGCAACAGTCCTGGTTCTCTTAACACTACTAGAAGTTGCCG

*E. bruneus* CAACCGTGGCTATTCTTACAGCAACAGTTCTAGTTCTTTTAACACTACTAGAAATTGCCG

*E. moara* CAACCGTAGCTATTCTTACAGCAACAGTCCTAGTTCTTTTAACACTGCTAGAAATTGCCG

*E. lanceolatus*  CAACTGTAGCCATTCTTACAGCAACAGTCCTAGTTCTTTTAACATTACTAGAAATTGCCG

*A. leucogrammicus*  CAACCGTTGCTATTCTCACTGCAACAGTCCTAATCCTTTTAACACTGTTAGAAGTCGCCG

*C. altivelis* CAGCCGTAGCCATCCTAACAACAACAGTCCTAATTCTTTTAACACTACTAGAAATTGCTG

*E. epistictus* CAACCGTAGCCATTCTTACAGCAGTAGTACTAGTTCTTTTAACACTACTAGAAGTTGCCG

*E. octofasciatus* CCACTGTAGCTATTCTCACAACAACTGTCCTAGTTCTTTTAACACTACTAGAAATTGCCG

*E. septemfasciatus* CCACAGTAGCTATCCTCACAGCAACTGTCCTAGTTCTTTTAACACTGCTAGAGATTGCCG

*T. dermopterus* CCACTGTAGCCCTCCTTACAACAACCGTATTATTACTCCTAACACTCCTAGAGATTGCGG

*E. awoara* CCTCTGTGGCCATTCTTACAGCAACAGTTCTAGTTTTATTAACACTTCTAGAGGTCGCCG

*E. akaara* CTTCTGTAGCCATTCTTACAGCAACAGTTCTAGTTCTATTAACACTTCTAGAGGTCGCCG

*E. trimaculatus* CTGCTGTAGCCATTCTTACAGCAACAGTTTTAGTCTTACTAACACTACTAGAAGTCGCCG

*E. areolatus* CTGCCGTAGCTATTCTTACAACAACAGTCCTAGTCCTACTAACACTTCTAGAAGTTGCCG

*V. albimarginata* CGACAGTAGCGCTTATCACAACAACAGTTCTGGTCCTACTTACACTACTAGAAATTGCCG

*V. louti* CAACAGTAGCACTAATTACAACAACAGTCCTAGTCCTACTTACATTGCTAGAAATTGCCG

*P. leopardus* CTTCTGTAGCAATTGTTACAGCCATGGTTCTTGTTCTTCTTACCCTCCTAGAGATTGCCG

*P. areolatus* CAACCGTTGCAATTATTACAACTATGGTTCTTGTCCTCCTCACCCTTTTAGAGATTGCCG

*E. radiosum* CTACAGTAGCAATCTTAACCTCAACAGTCCTTGTTCTTCTAACACTGCTAGAAATCGCCG

*P. sieboldi* CTACCGTCGCGCTTCTAACGGCCGCCCTCCTCTTCCTACTAACTCTCCTAGAAGTAGCCG

*E. armatus* CAGCTGTCTCAGCTATTACAGCACTTTTACTTCTTCTCCTCACCCTTCTAGAAATTGCAG

*R. oxyrhynchus* CAACCGTTGCCATTCTAACAGGAATCCTGCTGTTCCTCCTCACCCTTTTAGAAGTTGCAG

*K. cinerascens* CAACCGTAGCAATTCTCACAACAGCACTACTGTTTCTCCTCACCCTTCTAGAAGTAGCAG

*T. chatareus* CAACCGTGGCCGTCCTAACAGCTATTCTACTACTCCTTCTTACCCTACTAGAAGTGGCCG

*D. berycoides* CCCTGGTATCATTCTTTACAGCAACACTACTGGTACTACTTACCATCCTCGAGATCGCCG

*H. typus* CAACTGTGGCAATTGTAACATCAATATTGTTATTCCTACTTAGCTTACTAGAAGTTGCCG

*M. argenteus* CAACTGTAGCAATTCTTACAGCAACACTTTTATTTCTTCTTACCCTTTTAGAGGTTGCCG

*S. chuatsi* CCACAGTAGCAATTCTCACAGCAACACTACTATTCCTACTTACCCTCTTAGAAGTTGCCG

*O. fasciatus* CAACCGTTGCAATCCTCACAGGAACACTACTATTCCTTCTAACCCTGTTAGAAGTGGCCG

*P. trilineatum* CTGCCGTGGCCATTCTTACAGCAACACTTCTTTTCCTCCTAACATTGCTGGAAGTTGCCG

*M. salmoides* CCACAGTAGCGATTCTTACTGTAACCCTGCTGTTTCTTCTAACCCTTTTAGAGGTTGCCG

*P. tile* CCACTGTTGCAATTCTCACAGCAACACTTCTATTTCTCTTAACCCTACTAGAAGTGGCCG

*L. argentimaculatus* CAACCGTCGCAATTCTCACAGCAACGCTTCTATTCCTCCTAACACTTCTAGAAGTAGCTG

*E. struhsakeri* CTACCGTGGCCATTCTCACAGCAACATTACTGTTCCTACTAACCCTCCTAGAAGTCGCCG

*B. albus* CCGTAGTGGCAGTCCTCACTGCAACACTACTATTTCTACTCACGCTACTAGAAGTTGCCG

*C. auripes* CCGGCGTTGCACTCCTGACAATAATTTTACTTTTCCTGCTATCGCTCCTAGAAGTAGCGG

*C. melampygus* CCACAGTAGCCATCTTAACAACCATTCTCCTATTCTTACTTACACTGCTAGAAGTGGCAG

*L. calcarifer* CAACGGTGGCCGTCCTGACAACTGCCCTACTGTTTTTACTCACCCTCCTTGAAGTGGCCG

*S. maena* CAACCGTGGCAATTCTTACAGGTATTCTCCTTCTAATACTCTCCCTTCTTGAGGTAGCTG

*P. auriga* CTACTGTGGCAATTCTCACAGGCGTCCTACTCCTAATGCTCTCCATGTTAGAAGTTGCTG

*C. lucidus* CCCCAGTAGCAGCCCTTACCATGATTTTACTATTTCTACTCACCCTACTTGAAATTGCCG

*S. sihama* CGACTGTAGCTATCCTTACGACAATCCTGCTCTTCTTGCTAACGCTCTTAGAAGTAGCCG

*C. loricula* CAACAGTAGCAATACTTACCGCAGTCCTGCTACTCATGTTAACCCTCCTAGAAGTTGCCG

*A. trutta* CAGCCGTTGCCCTAACAACCGCAGCCCTGCTTTTCCTCCTTACAATTTTGGAAATTGCTG

*H. gemma* CAACAGTAGCAATTCTTACGGCTACTGTACTGGTTCTTCTTACACTTCTAGAAGTTGCCG

*P. flavescens* CTGTCGTAGCAATCTTAACTTCCACAGTCCTTGTTCTCCTAACCCTACTAGAAGTTGCCG

*P. macrolepida* CCACAGTAGCCATCCTAACTTCAACAGTACTTGTCCTCCTCACCCTCCTGGAAATCGCCG

*S. canadensis* CAATAGTGGCAATCCTAACTTCCACAGTTCTTGTCCTCCTTACCCTATTAGAAGTTGCTG

*A. rogaa* TTGCAATAATCCAAGCTTACGTATTTGTTCTATTATTAACCCTTTACCTACAAGAAAACA

*C. argus* TTGCAATAATCCAAGCTTATGTGTTTGTCTTACTACTAACTCTTTACCTACAAGAAAACA

*C. sonnerati* TTGCAATAATTCAAGCCTACGTATTCGTACTGCTACTCACACTCTACCTACAAGAAAACA

*E. fuscoguttatus* TAGCAATAATCCAAGCCTACGTCTTTGTATTGCTACTAACACTCTATTTACAAGAAAATA

*E. coioides* TAGCAATAATTCAAGCTTATGTGTTCGTACTACTACTAACACTTTACCTACAAGAAAACA

*E. bruneus* TAGCAATAATTCAAGCCTATGTGTTTGTATTACTACTAACGCTCTACTTACAAGAAAACA

*E. moara* TAGCAATAATTCAAGCCTATGTGTTTGTACTACTACTAACGCTCTACTTACAAGAAAACA

*E. lanceolatus*  TGGCAATAATCCAAGCCTATGTATTCGTATTGCTACTAACACTCTACTTACAAGAAAATA

*A. leucogrammicus*  TCGCAATAATCCAAGCTTATGTTTTCGTACTACTTCTAACACTATACTTACAAGAAAACA

*C. altivelis* TAGCAATGATTCAAGCTTACGTATTCGTATTGTTACTAACACTCTACCTACAAGAAAACA

*E. epistictus* TAGCAATGATTCAAGCCTACGTGTTCGTATTACTATTAACACTCTACCTACAAGAAAACA

*E. octofasciatus* TAGCAATAATTCAAGCCTATGTATTTGTATTGCTACTAACACTCTACTTACAAGAGAATA

*E. septemfasciatus* TAGCAATAATTCAAGCCTATGTATTTGTATTACTACTAACACTCTACTTACAAGAGAACA

*T. dermopterus* TAGCAATAATTCAAGCCTATGTGTTTGTACTACTACTAACGCTCTACCTACAAGAGAACA

*E. awoara* TAGCTATAATCCAAGCCTACGTCTTCGTACTGCTTCTAACACTATACCTACAAGAAAACA

*E. akaara* TAGCTATAATTCAAGCCTACGTCTTCGTACTACTTCTGACACTATACCTACAAGAAAACA

*E. trimaculatus* TTGCAATAATTCAAGCGTACGTTTTCGTACTACTACTAACACTATACCTACAAGAGAACA

*E. areolatus* TAGCAATGATTCAAGCCTATGTCTTCGTTCTGCTACTAACACTTTATCTACAAGAAAACA

*V. albimarginata* TAGCTATAATTCAAGCCTACGTCTTTGTATTACTCCTAACACTTTACCTCCAAGAAAACA

*V. louti* TAGCTATAATTCAAGCCTACGTCTTTGTTCTCCTCCTAACTCTTTACCTCCAAGAAAATA

*P. leopardus* TAGCAATAATCCAAGCTTATGTATTCGTTCTACTCCTAACACTTTATCTTCAAGAAAACC

*P. areolatus* TTGCAATAATCCAAGCCTATGTATTTGTATTACTCCTAACACTTTACCTTCAAGAAAACA

*E. radiosum* TAGCTATAATTCAAGCCTACGTATTTGTTCTCCTCTTAACCCTTTATTTACAGGAAAACG

*P. sieboldi* TCGCAATGATCCAGGCCTACGTCTTCGTCCTTCTCTTAAGCCTCTACTTACAAGAAAACG

*E. armatus* TCGCAATAATCCAAGCTTACGTCTTTGTACTCCTTTTAAGCCTTTACCTACAAGAAAACG

*R. oxyrhynchus* TAGCCATGATTCAGGCCTACGTATTTGTCCTCCTTTTAAGCCTCTACCTACAAGAAAACG

*K. cinerascens* TAGCAATGATTCAAGCCTATGTATTTGTTCTTCTTCTAAGCCTCTACCTACAAGAAAACG

*T. chatareus* TAGCAATGATTCAAGCTTATGTATTCGTACTCCTCTTAAGCCTCTACCTACAAGAAAACG

*D. berycoides* TAGCTATAATTCAAGCCTACGTATTTGTACTTCTCTTAAGCCTCTATCTTCAAGAAAACG

*H. typus* TAGCAATAATTCAAGCCTATGTCTTCGTACTTCTCTTAACCCTCTACCTACAAGAAAACG

*M. argenteus* TCGCTATAATCCAAGCCTACGTCTTTGTTCTACTCCTAAGCCTTTACCTACAAGAAAACG

*S. chuatsi* TTGCTATAATTCAAGCTTACGTCTTCGTCCTTCTCTTAAGCCTTTACCTACAAGAAAACG

*O. fasciatus* TAGCCATAATTCAAGCCTACGTATTTGTGCTCCTCCTCAGCCTATACCTCCAAGAAAACG

*P. trilineatum* TCGCAATAATTCAAGCCTATGTTTTCGTTCTTCTCTTAAGCCTTTATCTCCAAGAGAATG

*M. salmoides* TGGCAATAATTCAGGCCTACGTCTTTGTTCTTCTTTTAAGCCTCTACCTACAAGAAAACG

*P. tile* TCGCAATAATCCAAGCTTATGTCTTCGTCCTTCTCCTAAGCCTCTACCTACAAGAAAACG

*L. argentimaculatus* TAGCAATAATTCAAGCTTATGTCTTTGTCCTCCTTCTAAGCCTCTACCTACAAGAAAACG

*E. struhsakeri* TAGCTATAATTCAAGCCTACGTCTTTGTCCTACTCTTAAGCCTCTACCTACAAGAAAACG

*B. albus* TGGCAATAATTCAAGCCTATGTGTTTGTCCTTCTCCTCAGCCTGTACCTCCAAGAAAACG

*C. auripes* TCGCTATAATCCAAGCATACGTATTTGTTCTTCTTCTAAGTCTATACTTACAAGAAAATC

*C. melampygus* TAGCAATGATTCAAGCCTACGTATTTGTTCTACTACTAAGCTTGTACCTACAAGAAAACG

*L. calcarifer* TTGCTATAATTCAAGCTTATGTCTTCGTCTTACTTCTGAGCCTCTACCTACAAGAAAACG

*S. maena* TTGCAGTCATTCAAGCCTATGTATTTATCCTTCTTCTAAGCCTCTACCTGCAAGAAAACG

*P. auriga* TTGCAGTAATTCAGGCTTATGTCTTTATTCTTCTCTTAAGCCTCTACCTACAAGAAAACG

*C. lucidus* TAGCCATGATTCAAGCCTATGTCTTTGTTCTCCTGCTAAGCCTCTACCTACAAGAAAACG

*S. sihama* TGGCAATAATCCAAGCCTACGTGTTTGTTTTATTATTAAGCCTTTATCTCCAAGAAAACG

*C. loricula* TCGCAATAATTCAGGCCTATGTATTTGTTCTTCTCCTGAGCCTGTACCTACAAGAAAACG

*A. trutta* TAGCCATGATCCAGGCTTATGTATTCGTCTTACTCTTAAGCCTATACCTACAGGAAAATG

*H. gemma* TAGCAATAATTCAAGCTTACGTCTTTGTTCTACTAGTCACACTTTATCTACAAGAAAACG

*P. flavescens* TAGCTATAATTCAAGCCTACGTATTTGTTCTTCTCCTATCTCTCTATCTACAAGAAAACG

*P. macrolepida* TAGCTATAATCCAAGCCTACGTATTTGTTCTTCTCTTAACCCTTTACCTACAAGAAAACG

*S. canadensis* TAGCCATAATCCAGGCCTACGTATTTGTCCTTCTCCTATCCCTCTATCTTCAAGAAAACG

*A. rogaa* TCTAATGACCCCCGTTCACTTTGCTTTTTCATCAGCCTTCATCCTAGGCCTTACAGGCCT

*C. argus* TTTAATGACCCCTATTCACTTTGCCTTTTCATCAGCGTTTATGCTAGGCCTCACAGGCCT

*C. sonnerati* TCTAATGACCCCCGTTCACTTCGCTTTCTCATCAGCCTTCATGCTAGGCCTCACAGGCCT

*E. fuscoguttatus* TTTAATGACCCCCGTACACTTCGCTTTTTCATCAGCCTTTATTTTGGGCCTAACAGGCCT

*E. coioides* TTTAATGACCCCTGTGCACTTTACTTTTTCATCCGCTTTTATTCTAGGCCTAACAGGCCT

*E. bruneus* TTTAATGACCCCTGTGCACTTTGCTTTTTCATCCGCTTTTATCTTGGGCCTAACAGGTCT

*E. moara* TTTAATGACCCCTGTGCACTTTGCTTTTTCCTCCGCTTTTATCTTGGGCCTAACAGGCCT

*E. lanceolatus*  TTTAATGACCCCCGTGCACTTCGCTTTTTCATCCGCTTTTATCTTAGGCCTGTCAGGCCT

*A. leucogrammicus*  TTTAATGACCCCCGTGCACTTTGCTTTTTCATCTGCTTTTATCTTGGGCCTTACAGGCCT

*C. altivelis* TCTAATGACCCCTGTACACTTTGCCTTTTCATCCGCCTTTATCCTAGGCCTAACAGGCTT

*E. epistictus* TTTAATGACCCCCGTGCACTTTGCTTTTTCATCCGCTTTCATTTTAGGCCTAACAGGCCT

*E. octofasciatus* TTTAATGACCCCTGTGCACTTTGCTTTTTCATCCGCTTTTATTTTGGGCTTAACAGGCCT

*E. septemfasciatus* TTTAATGACCCCCGTGCACTTCGCTTTTTCATCCGCTTTTATCTTAGGCCTGTCAGGCCT

*T. dermopterus* TTTAATGACCCCCGTGCACTTTGCCTTTTCATCCGCTTTTATCTTGGGCCTAACAGGCCT

*E. awoara* TCTAATGACTCCCGTGCACTTTGCTTTCTCATCCGCTTTCATCCTGGGCCTTACAGGCTT

*E. akaara* TCTAATGACCCCCGTACACTTTGCTTTCTCATCCGCTTTCATCCTAGGCCTTACAGGCTT

*E. trimaculatus* TCTAATGACCCCTGTGCACTTTGCTTTTTCATCCGCTTTCATTCTGGGCCTCACAGGCCT

*E. areolatus* TCTAATGACTCCCGTGCACTTCGCTTTCTCATCCGCTTTCATTCTGGGCCTCACAGGCTT

*V. albimarginata* TCTAATGACTCCGGTGCACTTTGCTTTTTCATCAGCCTTTTTATTAGGCCTCACGGGCCT

*V. louti* TCTAATGACCCCGGTGCACTTTGCTTTTTCATCAGCCTTTCTATTAGGCCTCACGGGCCT

*P. leopardus* TCTAATGACCCCTATCCACTTCACCTTTTCATCAGCATTTATGCTCAGTCTTGCAGGTGT

*P. areolatus* TCTAATGACCCCTATCCACTTCACCTTTACATCAGCATTTATGCTCAATCTTGCGGGTGT

*E. radiosum* TTTAATGACCCCCGTTCACTTTGCTTTCTCATCAGCTTTTATTCTTGGACTAACAGGCCT

*P. sieboldi* TCTAATGACCCCTGTTCACTTCGCTTTCTCAACAGCATTTATTTTAGGCCTCACGGGATT

*E. armatus* TCTAATGACCCCCGTTCACTTCGCCTTTTCATCGACCTTCATTCTAGGACTTGCAGGTCT

*R. oxyrhynchus* TTTAATGACTCCCACCCATTTTGCTTTTTCAACAACCTTCATTCTTGGATTGATAGGCCT

*K. cinerascens* TTTAATGACTCCCGTTCACTTTGCCTTCTCATCAACTTTTTTACTGGGGTTGACAGGCCT

*T. chatareus* TCTAATGACCCCCACACACTTTGCTTTCTCATCAGCCTTCCTCCTAGGCCTGACAGGACT

*D. berycoides* TCTAATGACCCCCGTCCACTTCGCCTTCTCATCAGCCTTTATGCTAGGCTTAACAGGCCT

*H. typus* TCTAATGACCCCTGTCCACTTCGCTTTTTCATCAGCCTTTATACTAGGCCTGACAGGCTT

*M. argenteus* TCTAATGACTCCCGTTCACTTCACCTTTTCGGCCACCTTCATACTAGGACTAACAGGCCT

*S. chuatsi* TCTAATGACCCCCGTTCACTTCACTTTTTCATCGACCTTCTTACTAGGGTTAACAGGACT

*O. fasciatus* TCTAATGACCCCCGTCCACTTTGCCTTCTCATCAACCTTTCTACTAGGGTTGACAGGCCT

*P. trilineatum* TCTAATGACCCCCGTCCACTTCGCTTTTTCAGCAGCCTTTATGTTAGGATTAGCCGGCCT

*M. salmoides* TTTAATGACCCCTGTTCACTTTGTTTTTTCATCAACCTTCCTCCTGGGCCTCACAGGCCT

*P. tile* TCTAATGACCCCAGTTCACTTCACCTTCTCCTCAGCCTTCATACTAGGACTAACAGGGCT

*L. argentimaculatus* TCTAATGACCCCCGTTCACTTCACCTTCTCCTCAGCCTTCATACTAGGATTGACAGGGCT

*E. struhsakeri* TCTAATGACCCCCGTTCACTTCACTTTTTCATCGGCCTTTATACTAGGATTAACAGGCCT

*B. albus* TTTAATGACCCCTGTCCACTTCGCCTTTTCATCCGCCTTTACACTAGGCCTGATAGGTCT

*C. auripes* TCTAATGACACCCACACATTTTGCTTTTTCATCAGCCTTCATGTTGGGCCTATCCGGCCT

*C. melampygus* TCTAATGACCCCTGTTCACTTTGCCTTCTCATCAACATTTATACTAGGATTAGCAGGCTT

*L. calcarifer* TATAGTGACACCTTCTCACTTTGCCTTTACGTCTGCATTCATACTTGGCCTCACCGGTCT

*S. maena* TTTAATGACCCCTGTTCACTTCACCTTTTCATCTGCCTTCATGTTAGGACTAACAGGCCT

*P. auriga* TTTAATGACCCCCATTCACTTTACCTTCTCCTCCGCATTTATGTTAGGCTTAACAGGCCT

*C. lucidus* TCTAATGACCCCCACCCATTTTGCTTTCTCCGCAGCCTTCATTCTGGGATTAGCAGGCTT

*S. sihama* TCTAATGACTCCCGTCCACTTCGCCTTCTCTACTACTTTCCTCCTAGGCTTAACAGGTTT

*C. loricula* TCTAATGACTCCTGCTCACTTCGCCTTCTCGTCAGCCTTTATGCTAGGCCTCGCAGGCCT

*A. trutta* TATAATGACTCCTATCCACTTCGCCTTCTCATCGACATTCATGTTAGGGCTTACCGGGCT

*H. gemma* TTTAATGACACCTACTCACTTCTCGTTTTCACTAGCATTTATTCTGGGACTAATAGGACT

*P. flavescens* TCTAATGACCCCAGTTCACTTTGCTTTCTCATCAGCCTTCATCCTAGGACTAACAGGCCT

*P. macrolepida* TCTAATGACCCCCGTTCACTTTGCTTTTTCATCAGCCTTCATCCTAGGGCTAACCGGCCT

*S. canadensis* TCTAATGACCCCCGTTCACTTTGCTTTTTCATCAGCCTTCATTCTAGGGCTAACAGGCCT

*A. rogaa* AGCATTTCACCGAACCCATCTTCTCTCCGCTCTTTTATGCCTGGAGGGAATAATACTCTC

*C. argus* AGCATTTCACCGTATTCACCTTCTCTCCGCTCTTTTATGCCTAGAAGGAATAATACTCTC

*C. sonnerati* AGCATTTCACCGAACCCATCTTCTCTCCGCCCTTTTATGTCTAGAAGGAATGATGCTGTC

*E. fuscoguttatus* AGCATTCCACCGAACCCACCTTCTCTCTGCCCTCCTATGCTTAGAAGGAATAATGCTCTC

*E. coioides* AGCATTTCACCGAACCCACCTCCTCTCTGCCCTCCTCTGTTTAGAAGGAATAATGCTCTC

*E. bruneus* GGCATTCCATCGAACCCACCTTCTCTCCGCCCTCCTCTGTCTAGAAGGCATAATGCTCTC

*E. moara* GGCATTCCATCGAACCCACCTTCTCTCCGCCCTCCTCTGTCTAGAAGGCATAATGCTCTC

*E. lanceolatus*  AACATTTCACCGAACCCACCTTCTCTCTGCCCTCCTATGTTTAGAAGGAATAATACTCTC

*A. leucogrammicus*  AGCATTCCACCGAACCCACCTTCTCTCCGCCCTCCTGTGTTTAGAAGGAATAATGCTCTC

*C. altivelis* GACATTCCACCGAACCCACCTCCTCTCCGCCCTCCTGTGCTTAGAAGGAATAATACTCTC

*E. epistictus* AGCATTCCACCGAACCCACCTTCTCTCCGCTCTTTTATGCTTAGAAGGTATAATGCTCTC

*E. octofasciatus* GGCATTCCACCGAACCCACCTTCTCTCTGCTCTCCTATGTTTAGAAGGAATAATACTCTC

*E. septemfasciatus* AACATTTCACCGAACCCACCTTCTCTCTGCCCTCCTATGTTTAGAAGGAATAATACTCTC

*T. dermopterus* AGCATTTCACCGAACCCACCTTCTCTCTGCTCTCCTCTGTTTAGAGGGGATAATGCTTTC

*E. awoara* GGCATTTCACCGAACCCACCTTCTCTCTGCCCTCCTATGTTTAGAAGGAATAATGCTCTC

*E. akaara* GGCATTTCACCGAACCCATCTTCTCTCTGCCCTCCTATGTCTAGAAGGAATAATACTCTC

*E. trimaculatus* AGCATTCCACCGAACCCATCTTCTTTCCGCCCTTCTGTGCTTAGAGGGTATAATGCTCTC

*E. areolatus* AGCATTCCACCGAACCCATCTTCTTTCTGCTCTCCTCTGTTTAGAAGGAATAATGCTCTC

*V. albimarginata* AGCATTCCATCGAACCCATCTCCTTTCTGCCCTCCTATGCTTGGAAGGGATAATACTTTC

*V. louti* AGCATTCCATCGAACCCATCTTCTTTCCGCCCTCCTATGCTTAGAAGGGATAATACTTTC

*P. leopardus* TGCATTCCAACGAACACACCTTATTTCTGCCCTCCTCTGCTTAGAAGGCATAATACTCTC

*P. areolatus* TGCATTCCAACGAACACACCTTATCTCTGTCCTCCTTTGTTTAGAAGGCATAATACTCTC

*E. radiosum* AGCGTTTCATCGCACCCATCTTCTTTCCGCCCTCTTATGCTTAGAAGGAATAATACTCTC

*P. sieboldi* GACATTCCACCGGACTCACCTTTTATCCGCCCTCCTCTGCCTAGAAGGCATAATACTATC

*E. armatus* AGCATTCCACCGAACCCATCTCCTCTCTGCTCTTCTTTGCTTAGAGGGAATAATACTCTC

*R. oxyrhynchus* TGCATCCCACCGAACACACCTCCTCTCCGCTCTCCTCTGTCTAGAAGGTATAATGCTTTC

*K. cinerascens* TGCATTCCATCGAACCCACCTTCTCTCCGCCCTCCTATGTCTAGAGGGCATAATACTTTC

*T. chatareus* GGCATTCAACCGATTTCATCTTCTCTCCGCCCTTCTCTGCCTAGAGGGTATAATATTATC

*D. berycoides* AGCATTTCATCGAACCCACCTCCTTTCCGCCCTCCTATGCCTAGAGGGAATAATACTTTC

*H. typus* AGCATTCCATCGAACCCACCTCCTCTCTGCCCTCCTGTGCCTAGAAGGAATAATACTCTC

*M. argenteus* AGCATTTCATCGGACCCACCTTCTTTCCGCCCTCTTATGTCTAGAAGGAATAATACTCTC

*S. chuatsi* AGCCCTCCACCGAACCCATCTTCTCTCTGCTCTCCTATGTTTAGAGGGAATAATACTCTC

*O. fasciatus* CGCATTCCATCGAACCCATCTTCTCTCTGCCCTCTTATGCTTAGAAGGCATAATACTTTC

*P. trilineatum* AGCATTTCACCGAACTCATCTCCTCTCCGCCTTACTGTGCTTAGAGGGTATAATACTTTC

*M. salmoides* AGCCCTTCACCGAACCCACCTCCTCTCTGCCCTGCTCTGCTTAGAAGGAATGATGCTATC

*P. tile* AGCATTCCACCGAACCCACCTTCTCTCCGCACTCCTGTGTCTAGAAGGAATAATGCTTTC

*L. argentimaculatus* AGCATTCCACCGAACCCACCTTCTCTCCGCCCTCCTCTGCTTAGAAGGGATAATACTTTC

*E. struhsakeri* AGCATTCCATCGAACCCACCTTCTCTCTGCCCTACTGTGTTTAGAGGGGATAATACTCTC

*B. albus* GGTATTTCACCGAACCCACCTCCTCTCAGCCCTCTTGTGCTTAGAAGGTATAATACTCTC

*C. auripes* CGCCCTTCACCGGACACATCTTCTTTCTGCCCTTTTATGTTTAGAAGGAATAATGCTCTC

*C. melampygus* AGCATTCCATCGCTATCACCTTCTCTCCGCCCTACTCTGTCTAGAAGGAATAATACTCTC

*L. calcarifer* CGCCCTTCACCGCTATCACCTTCTCTCTGCCCTCTTGTGCTTAGAAGGAATGATACTTTC

*S. maena* CGCATTCCAGCGGACCCATCTTTTATCAGCCCTTCTCTGCTTAGAAGGAATAATACTGTC

*P. auriga* AGCGCTCCACCGGACCCATCTGTTATCAGCCCTTCTCTGCCTAGAAGGAATAATGTTATC

*C. lucidus* AGCATTCCATCGAACTCACCTCCTCTCAGCCCTTCTATGCCTAGAAGGAATAATGCTCTC

*S. sihama* AGCGTTTCACCGAACCCATCTACTCTCGGCCCTGCTATGCTTGGAAGGTATAATGCTATC

*C. loricula* AGCCTTCCATCGAACACACCTCCTCTCTGCACTCCTCTGCCTAGAAGGAATGATGTTATC

*A. trutta* CGCATTTCATCGAACCCACCTTTTGTCTGCATTGCTATGCCTAGAAGCTATGATACTCTC

*H. gemma* TGCGTTCCATCGAACTCATCTCCTATCTGCTCTCTTGTGTTTAGAAGGAATAATACTTTC

*P. flavescens* GGCATTCCACCGCACCCATCTTCTCTCCGCTCTTTTATGTCTGGAAGGAATAATGCTTTC

*P. macrolepida* GGCATTCCATCGCACCCACCTTCTCTCCGCCCTTTTATGCTTAGAGGGAATGATGCTTTC

*S. canadensis* GGCATTCCATCGCACCCACCTTCTCTCCGCCCTTCTATGCTTAGAGGGAATGATGCTTTC

*A. rogaa* CCTTTTCATTGCCCTTTCCCTTTGAACCCTTCAATTAAACTCCACAAGTTTTTGCGCAGC

*C. argus* CCTTTTTATTGCCCTCTCCCTCTGAACCCTTCAACTAAATTCTACAAGTTTTTGTGCGGC

*C. sonnerati* CCTATTTATTGCCCTATCCCTTTGAACCCTTCAACTAAACTCCACAAGCTTTTGTACAGC

*E. fuscoguttatus* CCTGTTCATTGCCCTCTCCCTCTGAACTGTCCATTTAAACTCCACAAGCCTCTGCGCAGC

*E. coioides* CCTATTTATAGCCCTCTCCCTCTGAACTGTCCAATTAAACGCCACAAGCCTCTGCGCAGC

*E. bruneus* CCTGTTTATTGCCCTCTCCCTCTGAACTGTCCAATTAGACTCCACAAGCCTCTGTGCAGC

*E. moara* CCTATTTATTGCCCTCTCCCTCTGAACTGTCCAATTAAACTCCACAAGCCTCTGTGCAGC

*E. lanceolatus*  CTTATTCATTGCCCTCTCCCTCTGAACTGTTCAATTAAACTCCACAAGCCTTTGCGCAGC

*A. leucogrammicus*  CCTGTTCATTGCCCTCTCCCTTTGAACCGTTCAATTGAACGCCACAAGTTTATGCGCAGC

*C. altivelis* CCTGTTCATTGCCCTCTCCCTCTGAACTGTCCAATTAAACTCAACAAGCCTCTGTCCAGC

*E. epistictus* CCTGTTTATTGCCCTATCCCTCTGAACTGTTCTACTAAACTCCACAAGCTTCTGTGCAGC

*E. octofasciatus* CCTCTTCATTGCCCTCTCCCTCTGAACTGTACAGTTGAACTCCACAAGCTTATGTGCGGC

*E. septemfasciatus* CTTATTCATTGCCCTCTCCCTCTGAACTGTTCAATTAAACTCCACAAGCCTTTGCGCAGC

*T. dermopterus* CCTGTTCATTGCCCTCTCCCTCTGAACTATCCAATTAAATTCCACAAGCTTGTGTGCAGC

*E. awoara* CCTGTTCATTGCCCTCTCCCTTTGAACCGTTCAATTAAATGCCACAAGCTTCTGCGCGGC

*E. akaara* CCTGTTCATTGCCCTCTCCCTTTGAACTGTTCAATTAAACGCCACAAGCTTCTGCACAGC

*E. trimaculatus* CCTATTTATTGCCCTCTCCCTTTGAGCTGTTCAATTAAACTCTACAAGCTTCTGTACAGC

*E. areolatus* CTTGTTTATTGCCCTTTCCATCTGAACCGTTCAATTAAACTCCACAAGCTTCTGTACAGC

*V. albimarginata* TCTCTTTATTGCATTCTCCCTTTGAACTCTACGGCTAGACTCCACAAGTCTATCTGCAGC

*V. louti* TCTCTTCATCGCATTCTCCCTTTGAACACTACGGCTAGACTCCACAAGCCTATCTGCTGC

*P. leopardus* TCTTTTCCTAGCCTTCTCGCTTTGAACCCTTCAATTAAGTTCCGCAAGCTTTTCTGCAGC

*P. areolatus* TCTTTTCCTAGCCTTCTCGCTTTGAACCCTCCAATTAAGTTCAGCAAACTTTTCTCCAGC

*E. radiosum* TCTATTTATTGCCCTCTCTCTCTGAACCCTTCAACTAGACTCTACTAGTTTCTCGGCAGC

*P. sieboldi* TCTCTTCATTGCTCTCTCACTGTGAACCCTACAATCAGGGTCCACCTGCTTCTCAGTCGC

*E. armatus* TCTATTTATTGCCCTATCACTATGGACTCTTCAATTAGATTCCACCAATTTTTCAGCCTC

*R. oxyrhynchus* TTTATTTATTGCTTTCTCCCTGTGAACCCTTCAACTGGGCTCTTCTAGCTTTTCCGCTTC

*K. cinerascens* TTTATTTATTGCCCTCTCCCTTTGAACCCTGCAACTTGATTCCACCAACTTCTCACCCTC

*T. chatareus* CCTATTTGTTGCCCTCTCCCTATGAACCCTGCAACTAGATACCACTAACTTCTCAGCCTC

*D. berycoides* TTTATTTATTGCCCTCTCTTTATGAACACTAGAGCTGGACTCTACCAACTTTTCAACTTC

*H. typus* CCTATTTATTGCCCTCTCCTTGTGGACACTCCAGCTAGATTCCACCAACTTTTCGGCATC

*M. argenteus* CCTGTTCATTGCCCTTTCCCTATGAACCCTTCAACTAAACGCCACCAACTTTTCAGCCTC

*S. chuatsi* CCTGTTTATTGCACTCTCCCTATGGACCCTACAACTAGACTCCACTAACTTCTCAGCCTC

*O. fasciatus* TTTGTTTATTGCACTGTCCCTCTGAACCCTCCAACTTGATTCCACCAGCTTCTCAACCTC

*P. trilineatum* CCTATTTATTGCCCTCTCCCTATGAACACTGCAACTCAATGCCACCAGCTTTTCAGCCGC

*M. salmoides* TTTATTTATTGCGCTCTCCCTATGGACACTTCAATTAGACTCAACCAGCTTCTCAGCTGC

*P. tile* TCTATTTATTGCCCTCTCCCTATGAGCCCTACAACTAAGCACCACCCACTTCTCAGCATC

*L. argentimaculatus* TTTATTTATTGCCCTTTCTCTGTGAGCCCTACAACTAAGCACTACACACTTCTCAGCCTC

*E. struhsakeri* TCTATTTATTGCCCTCTCCTTATGAACCCTGCAACTAAACACTACCAGCTTCTCAGCTTC

*B. albus* CTTATTCATCGCCCTCTCCGTATGAACCCTCCAACTAAACGCTACCAACTTCGCAATCTC

*C. auripes* CCTCTTCCTGGCCCTCTCCCTATGGGCCCTCCAACTTAACGTTTCCATCTTTTCCGCCTC

*C. melampygus* ACTCTTCGTTGCCCTCTCCCTCTGGACCCTTCAACTAGATTCCACTAGCTTTTCAGCCTC

*L. calcarifer* CCTGTTCATTGCCCTCTCACTCTGAACCCTCCAACTTGACTCTACTTGCTTTTCAGCATC

*S. maena* TTTATTTGTTGCTTTATCCCTGTGAGCCCTCCAGTTAAGTTCAATCAACTTCTCAGCTTC

*P. auriga* TTTATTTATTGCCCTCTCACTATGAGCCCTCCAACTAAGCACGGTTAGCTTCTCAGCCTC

*C. lucidus* CCTATTCATTGCCCTCTCTCTATGAACCCTTCAACTAGATTCCACCAACCTCTCAGCCTC

*S. sihama* CCTGTTCGTGGCTCTCTCCCTATGAACTCTTGAATTAAGCTCCACCAACTTTTCAGCAGC

*C. loricula* GCTATTTATTGCCATGTCCCTTTGAGCATTACAGCTAGACGCCACGGCCTTTTCGCCAGC

*A. trutta* CCTCTTCATTGCCCTCTCAATCTGAGCCTTACAACTAGGCTCCTCTAACTTTTCAACTTC

*H. gemma* CCTATTTGTTGCGCTTTCCCTTTGAACCTTGCAACTTGGCTCATCTAGCTTCTCCGCCGC

*P. flavescens* TCTATTTATTGCCCTCTCCCTTTGAACTCTGCAATTAGACTCCACAAACTTCTCAGCAGC

*P. macrolepida* TTTATTCATCGCGCTCTCTCTCTGAACCTTGCAACTAGACTCTACAAACTTCTCGGGGGC

*S. canadensis* TTTATTTATTGCCCTCTCCCTTTGGACCTTACAATTAGATTCCACAAACTTTTCAGCAGC

*A. rogaa* CCCTATACTTCTGCTGGCTTTCTCAGCCTGTGAAGCAAGCGCAGGGCTCGCTCTACTAGT

*C. argus* CCCAATACTCTTATTAGCCTTTTCAGCTTGCGAAGCAAGTGCAGGACTTGCCCTGCTAGT

*C. sonnerati* CCCTATACTACTACTAGCTTTTTCAGCCTGCGAAGCAAGTGCAGGACTCGCCCTACTAGT

*E. fuscoguttatus* CCCTATACTACTACTAGCTTTCTCAGCTTGCGAAGCAAGCGCAGGCCTCGCCCTACTGGT

*E. coioides* CCCCATACTATTACTAGCTTTTTCAGCCTGTGAAGCAAGCGCAGGACTCGCCCTACTGGT

*E. bruneus* CCCCATACTACTGCTGGCCTTCTCAGCTTGTGAAGCTAGCGCAGGCCTAGCCCTACTAGT

*E. moara* CCCCATACTACTACTGGCCTTCTCAGCCTGCGAAGCAAGCGCAGGCCTAGCCCTACTAGT

*E. lanceolatus*  CCCTATATTACTACTGGCTTTCTCAGCCTGTGAAGCAAGCGCAGGACTCGCTCTACTAGT

*A. leucogrammicus*  TCCTATACTGCTATTAGCCTTCTCAGCTTGTGAAGCAAGCGCAGGACTTGCTCTACTAGT

*C. altivelis* CCCCATACTACTTCTAGCTTTCTCAGCTTGCGAAGCAAGTGCAGGACTTGCCCTGCTAGT

*E. epistictus* CCCTATATTATTACTAGCTTTCTCAGCTTGTGAAGCAAGCGCAGGGCTCGCCCTTTTAGT

*E. octofasciatus* CCCTATACTATTACTGGCTTTTTCAGCCTGTGAGGCAAGTGCAGGACTCGCCCTACTGGT

*E. septemfasciatus* CCCTATATTACTACTGGCTTTCTCAGCCTGTGAAGCAAGCGCAGGACTCGCTCTACTAGT

*T. dermopterus* CCCCATGTTGCTTCTGGCTTTCTCAGCCTGTGAAGCAAGCGCAGGACTTGCCCTGCTAGT

*E. awoara* CCCTATACTATTGCTAGCTTTCTCAGCCTGTGAAGCAAGTGCAGGACTCGCCCTACTGGT

*E. akaara* CCCTATATTGTTACTAGCTTTCTCAGCCTGTGAAGCAAGCGCAGGACTCGCTCTACTGGT

*E. trimaculatus* CCCTATGCTACTACTAGCCTTCTCAGCTTGCGAAGCAAGTGCAGGACTCGCCCTACTGGT

*E. areolatus* TCCCATACTACTACTTGCTTTCTCAGCCTGTGAAGCAAGCGCAGGACTTGCCCTGCTAGT

*V. albimarginata* ACCTATACTCCTCCTAGCATTTTCAGCCTGCGAAGCCAGCGCGGGTCTTGCACTATTAGT

*V. louti* ACCCATACTCCTATTAGCATTTTCAGCTTGCGAAGCCAGCGCAGGTCTTGCATTATTAGT

*P. leopardus* ACCAATGCTACTCTTAGCATTTTCAGCTTGTGAAGCAAGCGCAGGCCTTGCTCTCTTAGT

*P. areolatus* AGCAATACTCCTCCTAGCATTTTCAGCTTGTGAAGCAAGCGCAGGTCTTGCCCTCTTAGT

*E. radiosum* TCCAATGCTCCTCCTAGCATTTTCAGCCTGCGAAGCAAGTGCAGGTCTTGCCCTTCTAGT

*P. sieboldi* CCCTATACTCCTCCTAGCTTTTTCAGCCTGTGAAGCGGGGGCCGGCCTGGCCCTCCTTGT

*E. armatus* CCCTATGCTACTTCTGGCATTTTCAGCTTGCGAAGCAAGCGCAGGCCTAGCATTACTGGT

*R. oxyrhynchus* CCCTATGATCCTTCTTGCCCTTTCGGCTTGCGAAGCAAGCGCAGGGCTCGCCCTCCTGGT

*K. cinerascens* GCCCATGCTCCTATTAGCATTTTCAGCTTGCGAGGCAAGCGCAGGCCTAGCCCTACTAGT

*T. chatareus* TCCCATGCTTTTACTAGCATTTTCAGCTTGTGAAGCAAGCGCAGGTCTTGCCCTACTAGT

*D. berycoides* CCCCATACTCCTTCTCGCGTTTTCAGCTTGTGAAGCAAGTGCAGGTCTTGCATTACTAGT

*H. typus* TCCCATACTTCTTCTTGCATTCTCAGCTTGTGAAGCAAGTGCAGGACTTGCCTTACTAGT

*M. argenteus* ACCCATGCTCCTCCTTGCATTCTCAGCCTGCGAAGCAAGCGCAGGTTTAGCATTACTAGT

*S. chuatsi* CCCTATACTACTACTAGCTTTCTCAGCTTGCGAAGCAAGTGCCGGACTCGCTCTATTGGT

*O. fasciatus* CCCTATACTCTTACTCGCATTTTCAGCTTGCGAAGCAAGTGCAGGACTTGCCCTGCTAGT

*P. trilineatum* CCCCATGCTTCTTTTAGCATTTTCAGCCTGTGAAGCAAGTGCAGGACTCGCCCTTCTAGT

*M. salmoides* CCCCATACTCCTGCTAGCTTTCTCAGCTTGCGAAGCAAGCGCTGGCCTTGCCCTACTAGT

*P. tile* CCCAATACTTCTACTCGCATTCTCAGCCTGTGAAGCAAGTGCAGGGCTTGCTCTTCTAGT

*L. argentimaculatus* ACCAATACTTCTGCTCGCATTCTCGGCCTGCGAAGCAGGTGCAGGACTTGTCATCCTAGT

*E. struhsakeri* TCCCATGCTTCTGCTAGCATTTTCAGCTTGTGAAGCAAGCGCAGGCCTTGCACTACTAGT

*B. albus* TCCAATACTTTTACTGGCATTTTCAGCCTGCGAAGCAAGCGCAGGCCTTGCCCTCCTAGT

*C. auripes* ACCCATGCTACTCCTCGCATTTTCAGCCTGTGAGGCAAGTGCTGGTCTAGCCCTCCTGGT

*C. melampygus* ACCTATACTTCTGCTGGCCTTCTCAGCATGTGAAGCAAGCGCAGGACTCGCTCTTCTAGT

*L. calcarifer* TCCCATGCTCCTCCTGGCTTTCTCAGCATGTGAAGCAAGCGCTGGACTTGCACTCCTAGT

*S. maena* TCCCCTCCTCCTCTTAGCACTTTCTGCCTGTGAAGCAAGTGCAGGACTTGCACTCCTAGT

*P. auriga* TCCCCTTCTCCTCCTTGCATTCTCAGCCTGTGAAGCAAGCGCAGGATTAGCATTGCTAGT

*C. lucidus* CCCCATACTTCTCCTAGCATTCTCAGCCTGCGAGGCAAGCACAGGTCTCGCACTACTAGT

*S. sihama* GCCTATACTCCTTTTAGCCTTTTCCGCTTGTGAAGCGAGCGCAGGGCTCGCCCTACTGGT

*C. loricula* ACCAATGCTTTTATTAGCATTCTCAGCTTGTGAAGCTAGCGCGGGCTTAGCCCTTCTAGT

*A. trutta* GCCAATACTTTTGCTAGCCTTTTCAGCCTGCGAAGCAAGCGCAGGACTAGCTCTGCTAGT

*H. gemma* TCCCCTACTTTTATTAGCATTTTCTGCTTGTGAGGCTAGTGCGGGCCTAGCCTTGCTTGT

*P. flavescens* TCCTATACTTCTATTGGCATTTTCAGCATGCGAAGCAAGCGCAGGCCTTGCCCTTCTCGT

*P. macrolepida* CCCCATACTCCTACTTGCATTTTCAGCCTGTGAAGCAAGTGCAGGCCTCGCCCTCCTGGT

*S. canadensis* CCCAATGCTTCTCCTAGCATTCTCAGCCTGTGAAGCAAGCGCAGGTCTTGCCCTTCTAGT

*A. rogaa* AGCAACAGCCCGAACTCACGGAACCGATCGCCTCCAAAGCCTTAATCTACTGCAATGCTA

*C. argus* AGCGACAGCCCGAACTCACGGAACCGACCGTCTCCAAAGCCTTAACTTGCTACAGTGCTA

*C. sonnerati* AGCAACAGCCCGAACTCACGGAACCGACCGCCTCCAAAGCCTTAATCTTCTACAATGCTA

*E. fuscoguttatus* AGCAACAGCCCGCACTCACGGAACCGACCACCTTCAAAACCTTAACCTGCTACAATGCTA

*E. coioides* AGCAACAGCTCGCACTCACGGAACCGACCACCTTCAAAACCTTAACCTTCTACAATGCTA

*E. bruneus* AGCAACAGCCCGCACTCACGGAACCGACCACCTTCAAAACCTTAATCTACTACAGTGCTA

*E. moara* AGCAACAGCCCGCACTCACGGAACCGACCACCTTCAAAACCTTAACCTACTACAGTGCTA

*E. lanceolatus*  AGCAACAGCCCGCACCCACGGAACCGACAACCTCCAAAACCTCAACCTACTACAATGCTA

*A. leucogrammicus*  AGCAACAGCTCGCACTCACGGAACCGACCACCTCCAAAACCTTAACCTTCTACAATGCTA

*C. altivelis* AGCAACAACCCGCACTCATGGAACCGACCACCTTCAAAACCTCAACCTATTACAGTGCTA

*E. epistictus* AGCAACAGCCCGCACTCACGGAACCGACCATCTTCAAAGCCTCAACCTACTACAGTGTTA

*E. octofasciatus* AGCAACAGCCCGCACTCATGGAACCGATCACCTCCAAAACCTCAACCTCTTACAATGTTA

*E. septemfasciatus* AGCAACAGCCCGCACCCACGGAACCGACAACCTCCAAAACCTCAACCTACTACAATGTTA

*T. dermopterus* AGCAACAGCCCGCACTCACGGAACTGACCACCTCAAAAACCTCAACCTCCTACAATGTTA

*E. awoara* AGCAACAGCCCGCACTCATGGAACCGACCACCTTCAAAGTCTCAACCTACTCCAATGCTA

*E. akaara* AGCAACAGCCCGCACTCATGGAACCGACCACCTTCAAAGCCTCAACCTACTTCAATGCTA

*E. trimaculatus* AGCAACAGCCCGCACTCATGGAACCGACCATCTTAAAAGCCTTAATTTACTACAGTGTTA

*E. areolatus* AGCAACAGCCCGCACTCATGGAACCGACCATCTTAAAAATCTCAACTTATTACAATGTTA

*V. albimarginata* TGCAACAGCCCGAACCCACGGAACCGACCGCCTTCAAAGTCTCAACCTTCTACAGTGTTA

*V. louti* TGCAACGGCCCGAACCCACGGAACCGACCGCCTTCAAAGCCTCAACCTTCTACAGTGTTA

*P. leopardus* AGCAACTGCACGCACCCATGGAACCGACCGCTTACAAAGTCTCAACCTCCTACAATGCTA

*P. areolatus* AGCAACTGCACGCACCCATGGAACCGACCGCTTACAAAACCTCAGCCTTCTACAATGCTA

*E. radiosum* GGCCACCGCTCGCACCCATGGGACCGATCGCCTCCAAAGCCTAAGCCTCTTGCAATGCTA

*P. sieboldi* AGCTACTGCTCGGACACACGGCACTGATCGCCTCCAAAACCTTAACCTCCTACAATGCTA

*E. armatus* TGCCACAGCCCGCACACACGGATCCGACCGCCTACAAACCCTTAATCTTCTACAATGCTA

*R. oxyrhynchus* AGCTACCGCCCGAACACACGGTTCTGATCACCTTCAAACCCTAAACCTCCTACAATGCTA

*K. cinerascens* AGCTACTGCTCGAACCCATGGCTCCGACCGCCTCCAAAGTCTAAACCTCCTACAATGCTA

*T. chatareus* AGCTACCGCCCGCACCCACGGTACTGATCGCCTACAAAGCTTAAACCTCCTAAAATGCTA

*D. berycoides* TGCCACCGCCCGTACACACGGAACCGACCGCCTACGGAGCCTAAACCTCCTACAATGTTA

*H. typus* AGCAACAGCTCGGACTCACGGAACTGATCGCCTGCAGAACCTAAACCTCCTACAATGCTA

*M. argenteus* AGCTACTGCCCGTACCCACGGAACCGACCGCCTACAGAGCCTAAACCTCCTACAATGCTA

*S. chuatsi* AGCTACAGCCCGTACCCACGGGTCTGACCGCTTACAAAGCCTAAATCTCCTACAATGCTA

*O. fasciatus* AGCCACCGCCCGAACCCACGGCTCCGACCGTCTCCAAAACCTAAACCTCCTACAATGCTA

*P. trilineatum* AGCGACTGCCCGTACCCACGGCACCGATCGCCTGCAGAACCTAAACCTATTACAGTGCTA

*M. salmoides* AGCCACCGCTCGAACCCACGGCTCTGATCGTCTACAAAATCTTAACCTCCTACAATGCTA

*P. tile* AGCCACCGCCCGAACCCACGGAACCGACCGCCTTCAGAGCTTAAACCTCCTACAATGCTA

*L. argentimaculatus* AGCTACCGCCCGTACCCACGGAACTGATCGTCTTCAGAGCCTAAACCTCCTACAATGCTA

*E. struhsakeri* AGCCACCGCCCGCACTCACGGAACCGACCGCCTACAAAGCCTAAACCTCCTACAATGCTA

*B. albus* CGCCACCGCCCGCACACACGGCTCCGACCGCCTACAAACCTTTAACCTCCTACAATGCTA

*C. auripes* AGCTACTACCCGAACTCACGGCAGCGACCGCATACAAAACCTTAATCTTCTCCAATGCTA

*C. melampygus* CGCCACCGCCCGAACGCACGGAACGGACCGCCTACAAAGCCTAAACCTACTACAATGCTA

*L. calcarifer* AGCCACTGCCCGCACTCATGGCACTGACCATCTCCAAAGTCTCAACCTCCTACAATGCTA

*S. maena* AGCAACTGCTCGCACCCACGGCTCTGATCGTTTACAAACCTTAAATCTCTTACAATGCTA

*P. auriga* AGCCACCGCCCGCACTCATGGCTCCGACCGTCTGCAAAGCTTAAACCTCCTACAATGCTA

*C. lucidus* CGCCACCACCCGAACACACGGAACCGATCGCCTACAAAGCCTAAACCTACTGCAATGCTA

*S. sihama* TGCTACCGCCCGAACCCATGGCACAGACCGCCTGCAAAGCCTTAACCTCTTACAATGCTA

*C. loricula* AGCGACAGCTCGCACCCACGGCACCGACCGCTTACAAAGCCTAAACCTCCTACAATGCTA

*A. trutta* AGCTACATCCCGAACTCATGGAGGTGACCGCCTGCAAGGCCTCAACCTCCTTCAATGCTA

*H. gemma* AGCAACTGCTCGAACCCATGGCACTGACCGACTTCAAAACTTAACTCTTCTACGATGCTA

*P. flavescens* AGCCACAGCCCGAACCCACGGAACCGACCGATTACAAAACCTTAATCTCCTACAATGCTA

*P. macrolepida* AGCCACCGCTCGAACTCACGGAACAGATCGTCTCCAAAGCCTTAACCTCTTACAATGCTA

*S. canadensis* AGCCACCGCCCGAACCCATGGCACTGACCGACTACAAAGCCTTAATTTATTACAATGCTA

*A. rogaa* AAAATTTTAATCCCTACTCTCATGCTTGTCCCAACGGCCTGGCTTACCCCTGCCAAATGA

*C. argus* AAAATTTTAATTCCCACCCTTATACTCGTCCCAACAACCTGATTTGCTCCCGCCAAATGG

*C. sonnerati* AAAATTCTTGTCCCAACCCTCATATTAGTCCCGACAGCTTGATTAACCCCTCCTAAATGA

*E. fuscoguttatus* AAAGTCCTTATCCCTACTCTAATACTAATTCCAACTGCCTGATTAACCCCAGCCAAATGA

*E. coioides* AAAGTCCTCATCCCCACCCTAATACTTATCCCAACTGCCTGATTAACCCCAACCAAATGA

*E. bruneus* AAGATCCTCATCCCCACCCTAATGCTTATCCCAACTGCCTGACTAACCCCGGCCAAATGA

*E. moara* AAAATCCTCATCCCCACCCTAATGCTTATCCCAACTGCCTGACTAACCCCCGCCAAATGA

*E. lanceolatus*  AAAATCCTCATCCCTACCCTAATACTCATTCCAACTGCCTGATTAACTCCAGCCAAATGA

*A. leucogrammicus*  AAAATCCTCATCCCCACCTTAATGCTTATCCCAACTATCTGACTGACCCCAGCTAAATGA

*C. altivelis* AAAGTCCTCATCCCCACCCTAATGCTTGTCCCAACTGCCTGATTAACCCCAGCTAAATGG

*E. epistictus* AAAATTCTTATCCCAACCCTAATGCTTGTTCCAATAGCCTGACTAACTCCGGCCAAATGA

*E. octofasciatus* AAAATCCTTATCCCAACTCTAATGCTTATCCCAACAGCCTGACTGGCCCCAGCCAAATGG

*E. septemfasciatus* AAAATCCTTATCCCAACCCTAATGCTTGTCCCAACAGCCTGACTGGCCCCAACCAAATGA

*T. dermopterus* AAAATCCTTATCCCAACCCTAATGCTTGTCCCTACAACCTGACTAGCCCCAGCCAAATGA

*E. awoara* AAAGTCCTCATCCCTACCTTAATACTTGTCCCAACTGCCTGATTGACCCCAGCCAAATGA

*E. akaara* AAAGTCCTCATCCCTACTTTAATACTTGTCCCAACTGCCTGACTTACCCCAGCCAAATGA

*E. trimaculatus* AAAATCCTCATTCCTACTTTAATGCTTATTCCAACCGCTTGATTAGCCCCAACCAAGTGA

*E. areolatus* AAGATCCTCATCCCTACTTTAATGCTAGTTCCAACTACCTGGCTGACCCCAGCTAAATGA

*V. albimarginata* AAAATTCTTATCCCTACACTCATACTAGTACCAACAACGTGACTGACCTCCCCCAAATGA

*V. louti* AAAATCCTCATCCCTACTCTCATACTAATTCCAACAACATGATTATCTTCTCCTAAATGG

*P. leopardus* AAAATCCTCATTCCTACCCTTATACTAGTACCAACAACTTGACTTACTCCTGCCAAATGA

*P. areolatus* AAAATTCTTATTCCGACTCTTATACTAGTCCCAACAACTTGACTTACCCCTACCAAATGA

*E. radiosum* AAGATTTTAATTCCAACCCTTATGCTTATTCCGACTGCTTGGGGGACACCAGCCAAATGA

*P. sieboldi* AAAATCCTTATTCCAACTCTCATGCTAATCCCAACTACTTGACTCTCCTCCACTAAATGA

*E. armatus* AAAATCCTAATTCCCACCCTCATGCTAGTACCAACAATTTGAATAGTCCCCGGCAAATGA

*R. oxyrhynchus* AAAGTACTCATCCCAACGCTTATGCTAATTCCCACTACTTGACTTACACCCCAAAAATGA

*K. cinerascens* AAAATCCTCATCCCTACCCTTATGCTAATCCCTACCACCTGACTGGCCCCGGCCAAATGA

*T. chatareus* AAAATCCTTATCCCTACCCTTATGCTTGTCCCAACCGCCTGAATGTCCTCCCCCAAGTGA

*D. berycoides* AAAGTTCTTATTCCAACCCTCATGCTTATCCCAACTATCTGAATGACCCCCGCCAAGTGG

*H. typus* AAAATCCTAATCCCAACCCTAATGCTAGTACCAACAACCTGAATAGTCCCCGCCAAATGA

*M. argenteus* AAAATCCTCATTCCAACACTAATGCTTATTCCTACCGCTTGATTAACACCCTCCAAGTGA

*S. chuatsi* AAAATCCTAATCCCCACCTTAATGCTAGTGCCAACAATTTGATTCTCCCCCGCTAAGTGA

*O. fasciatus* AAAGTTCTTATCCCAACCCTCATACTCATTCCAACCACATGACTGGCACCCTCCAAATGA

*P. trilineatum* AAAATTCTTATTCCCACACTAATGCTCGTACCAACAGCCTGACTCGCTCCTGCTAAATGG

*M. salmoides* AAAATTCTGGTCCCTACCCTGATGCTAGTCCCAACAATCTGAATAACCCCTGCCAAATGA

*P. tile* AAAATCTTAATCCCCACCCTCATGCTCGTTCCGACAACTTGGCTAGTCTCTGCCAAATGA

*L. argentimaculatus* AAAATTCTAATTCCAACCCTAATGCTTGTCCCAACAACTTGGCTAGTTTCGGCCAAATGA

*E. struhsakeri* AAAATCCTCATTCCAACCCTAATGCTTGTCCCAACCGCCTGAATAGCCCCTGCCAAATGA

*B. albus* ATAATCCTTATCCCCACCCTAATACTAATTCCTACCGCCTGAATTGTTCGCCCCCAGTGG

*C. auripes* ATTATTCTTGTCCCCACCTTTCTACTGGTCCTAACAACTTGGCTTACCCCCGCCAAATGA

*C. melampygus* AAAATTTTAATTCCAACTCTTATGTTAGTACCAACAACCTGATTAACGCCCGCCAAATGA

*L. calcarifer* AAAATGATTATCCCCACAATTATGCTCATTCCTACAACTTGACTAACACCCGCAAAATGG

*S. maena* AAAATTCTAGTCCCCACTCTTATGCTAATCCCAACAACCTGACTAGTTTCAGGTAAATGA

*P. auriga* AAAATTCTTCTTCCTACCTTTATGTTGGTTCCAACAATCTGACTAACTCCCACCAAATGA

*C. lucidus* AAAATTCTTATTCCCACATTAATGCTCATTCCAACAACCTGGCTAACCTCCGCCAAATGA

*S. sihama* AAAGTACTCATCCCGACTCTTATACTCATTCCCACAATCTGACTTTCCCCTAAAAAATGA

*C. loricula* AAAGTCCTCATTCCCACCTTAATGCTAGTCCCAACAATTTGGGCTATTCGCCCCAAACAC

*A. trutta* AAGATCCTCATCCCAACGCTTATACTTGTTCCAGTAGCCTGACTTACCCCTCATAAATGA

*H. gemma* AAAATCTTAATCCCAACCCTCTCATTAATATTGACAACCTGAATGACCCCCGGAAAGTGG

*P. flavescens* AAAATTCTTATTCCTACACTTATGCTAATTCCAACCGCCTGGGGGACCCCAGCTAAATGA

*P. macrolepida* AAAATCCTTATTCCAACACTTATGCTTATTCCAACTGCTTGAGGGGCCCCAGCTAAATGA

*S. canadensis* AAAATTTTAATTCCCACACTTATGCTAATCCCAACTGCCTGGGGGGCTCCCGCCAAATGG

*A. rogaa* CTTTGACCCACAACCCTTGCCCACAGCATACTAATTGCACTAATAAGCCTCTCCTGATTA

*C. argus* CTTTGACCCACAACTCTTACCCATAGCATACTAATTGCACTAATAAGCCTCTCCTGATTA

*C. sonnerati* CTTTGGCCCACAACCCTTGCCCACAGTATAATAATTGCACTATTCAGCCTCTCCTGATTA

*E. fuscoguttatus* CTTTGACCTACGACCCTTACCCACAGTATACTAATTGCACTAATCAGCCTTTCATGGTTA

*E. coioides* CTTTGGCCTACAACCCTTACCCACAGCATACTAATTGCATTAATTAGCCTTTCATGACTA

*E. bruneus* CTCTGGCCCACGACCCTTGCCCACAGTATACTAATTGCATTAATCAGCCTCTCATGATTA

*E. moara* CTCTGGCCCACAACCCTTGCCCACAGCATACTAATTGCATTAATCAGCCTCTCATGACTA

*E. lanceolatus*  CTCTGACCTACAACCCTCGCCCATAGTATACTAATTGCACTAATCAGCCTTTCATGGCTA

*A. leucogrammicus*  CTCCGACCTACAACCCTCACCCACAGCATATTAATTGCACTAGTCAGCCTTTCATGACTA

*C. altivelis* CTCTGACCCACAACTCTTGCCCATAGCATACTGATTGCATTAATTAGCCTCTCATGGTTA

*E. epistictus* CTTTGATCCACAATACTTGCCCATAGCATACTAATTGCACTAATTAGCCTCTCATGACTA

*E. octofasciatus* CTCTGACCTACAACCCTTACCCACAGCATACTAATTGCACTAATTAGCCTTTCGTGACTA

*E. septemfasciatus* CTCTGACCCACAACCCTTACCCACAGCATACTAATTGCACTAATTAGCCTTTCGTGACTA

*T. dermopterus* CTCTGGCCCACAACCCTCACCCATAGTATACTAATTGCACTAATCAGCCTCTCATGGCTA

*E. awoara* CTTTGACCCACAACCCTCGCCCACAGCATATTAATTGCACTTATTAGCCTTTCATGACTA

*E. akaara* CTCTGACCTACAACCCTCGCCCACAGCATATTAATTGCACTTATTAGCCTCTCATGACTA

*E. trimaculatus* CTCTGGCCCACAACCCTCACCCACAGTATACTAATTGCACTAATCAGCCTTTCATGACTA

*E. areolatus* CTCTGACCCACGACCCTTACACACAGCATACTAATTGCACTAATTAGCCTCTCATGATTA

*V. albimarginata* CTATGACCCACAACTTTAGCCCACAGCATACTAATTGCATTAGCTAGCCTTATTTGATTA

*V. louti* TTATGACCCACAACTTTAGCCCACAGCATACTCATTGCATTAACCAGCCTTATTTGACTA

*P. leopardus* CTATGACCAACAACCTTAACACACAGCCTTCTTATTGCACTAGCAAGCCTTTCCTGACTA

*P. areolatus* CTATGACCAACAACCCTGGTACATAGTCTTCTTATCGCATTAGCAAGCCTTTCCTGACTA

*E. radiosum* CTCTGGCCAACCACCCTAGTCCACAGCCTCGTTATTGCCCTAGCTAGTTTGGCTTGATTA

*P. sieboldi* GTTTGGCAAGCGACCACAGCTAACAGTCTGCTGATTGCCGTCCTTAGCCTAATTTGGATA

*E. armatus* CTTTGGCCAACCACCTTGCTCCAAAGCCTACTAATTGCCCTAGCCAGCTTCTCATGACTA

*R. oxyrhynchus* CTGTGGCCTACCACACTATTTCACAGCCTGGCCGTAGCCTTGGTAAGCCTTACCTGGCTT

*K. cinerascens* CTATGACCCACAACACTACTCCACAGCCTAATCGTAGCCGTGGCCAGCCTTTCCTGATTA

*T. chatareus* CTCTGGCCCACGGCCCTTACCCAAAGCTTTATTATTGCACTACTTAGTCTCTCCTGACTA

*D. berycoides* TTGTGGCCTACAACCCTTCTCCACAGCCTAATTATTGCACTTGTTAGTCTCACTTGATTT

*H. typus* CTCTGACCTACCACCCTTCTCCACAGCCTAATTATTGCATTAACCAGCCTCCTTTGACTA

*M. argenteus* CTATGGCCCACCTCCCTCCTCCATAGTCTAATCATTGCATTAGCTAGCCTCACCTGACTA

*S. chuatsi* CTCTGACCCTCCTCCCTTCTCCACAGCCTAGTAATCGCCCTGGCCAGCCTTACCTGACTA

*O. fasciatus* CTGTGACCTACAACCCTGCTTCACAGCCTATTAGTGGCCCTTATTAGCCTTACCTGACTG

*P. trilineatum* CTGTGACCTACAACCCTCCTACACAGTTTAGTAATTGCCCTTGCCAGCCTCAGCTGACTA

*M. salmoides* CTCTGACCCGCAACCCTTCTTCATAGTCTAACAATTGCCCTTATTAGCCTAACTTGATTA

*P. tile* TTATGACCAACCGCACTTCTGCACAGCCTAGTAATCGCACTAGCCAGCCTCACCTGACTA

*L. argentimaculatus* CTCTGACCCACAGCCCTCTTACACAGCCTCATCATCGCACTAGCTAGCCTTACCTGACTA

*E. struhsakeri* CTATGGCCCACAACCCTCATGCACAGCCTAGTAATTGCACTAGCCAGCCTCACCTGATTA

*B. albus* CTGTGGCCTACTGCCCTAGCCCACAGCCTGGTAATTGCACTAGCCAGCCTCTTCTGGTTC

*C. auripes* CTATGACCCACCACCCTTATACACAGTCTAATCATTGCCCTTGCTAGCCTCCATTGGTTA

*C. melampygus* CTATGACCAACCACCTTGGCCCACAGTTTCGTCATTGCCTTGGCCAGCCTAACCTGACTA

*L. calcarifer* CTATGACCCGCAACTCTTGCCCACAGCCTCCTTATTGCCATAGATAGCACAACCTGACTA

*S. maena* CTATGGCCAACAGTCCTCCTTCACAGTTTTATTATTGCTCTAGCTAGCTTCTCCTGACTG

*P. auriga* CTGTGACCCACAACCCTTCTACACAGTTTCGTTATTGCCCTAATCAGCCTTTCCTGACTA

*C. lucidus* TTATGACCCACCACCCTCGCGCACAGCCTATTAATCGCCTTAGCCAGCCTCAGCTGATTA

*S. sihama* TTATGACCAACTGCTCTGGCCCAGAGCTTATTAATTGCATTAATTAGTCTGTTATGATTA

*C. loricula* CTTTGACCCGCAGCTCTCGCGTACAGCCTAATAATCGCGTTAACTAGCTTTACCTGACTA

*A. trutta* TTGTGACCCACAACCCTCACCCACAGCCTTATTATTGCACTTGCTAGCCTAGCTTGGCTA

*H. gemma* CTTTGGCCCGTAACCTTGACGCATAGCCTGATTATTGCTTTATTCAGCCTTACATGACTT

*P. flavescens* TTATGACCTACAACCCTTGCCCACAGTCTAGTTATTGCCCTTATTAGTTTAACATGGTTA

*P. macrolepida* CTTTGACCCACAACCCTCGCCCACAGTCTAATCATCGCACTTGCAAGTCTGACTTGACTA

*S. canadensis* CTTTGGCCAACAACCCTCGCCCACAGCCTTATCATTGCTCTCATTAGCCTAACTTGGTTA

*A. rogaa* AAAAACACCATGGAAACAGGCTGATCTTCCCTCAATACACTTATAGCAACAGACCCTTTA

*C. argus* AAGAGCACCATGGAGACAGGCTGATCCACTCTTAATACACTTTTGGCAACAGACCCTTTA

*C. sonnerati* AAAAATACCATAGAAACAGGCTGATCTACTATCAACCCTTTCATAGCAACAGATCCCCTA

*E. fuscoguttatus* AAACATGCAATAGAAACAGGTTGATCTACTTTAAACCTGTTTATAGCCACAGACCCTTTA

*E. coioides* AAACACGCAATAGATACAGGCTGATGCACCCTAAACCTATTCATAGCTACAGACCCTTTA

*E. bruneus* AAACACGCTATAGAAACAGGCTGATCTACCCTAAGCCTATTTATAGCCACAGACCCCTTA

*E. moara* AAACACGCTATAGAGACAGGCTGGTCTACCCTAAACCTATTTATAGCCACAGACCCCTTA

*E. lanceolatus*  AAACATGCAATAGAAACAGGCTGAACCACCCTAAGCCTATTCATAGCTACAGACCCCTTA

*A. leucogrammicus*  AAACATGCAATAGATACAGGCTGATCTACCCTAAACCTGTTCATAGCCACAGACCCCCTG

*C. altivelis* AAACACGCAATAGAAACAGGCTGATCCACCCTAAGCTTATTTATAGCCACAGACCCCCTT

*E. epistictus* AAATATTCAATAGAAACAGGCTGAACTGCCCTAAGCATGTCTATAGCCACAGACCCCCTA

*E. octofasciatus* AAAAATGCAATAGAAACAGGCTGATCTACCCTTAACCCCTTCATAGCTACAGACCCTTTA

*E. septemfasciatus* AAAAGTGCAATAGAAACAGGCTGATCTACCCTTAACCTCTTCATAGCTACAGACCCCTTA

*T. dermopterus* AAAAATGCAATAGAAACAGGCTGATCTACCCTTAGCCCCTTCTTAGCCACAGACCTCTTA

*E. awoara* AAGTATGCATTAGAAACAGGTTGATCCACTCTAAACCTATTTATAGCAACAGACCCCCTA

*E. akaara* AAACATGCGATAGAAACAGGTTGATCTACCCTAAACCTATCTATAGCAACAGACCCCCTA

*E. trimaculatus* AAAAATACAATAGAAACAGGCTGATCTACACTAAACCTCTTCATAGCCACAGACCCTTTA

*E. areolatus* AAATGTTCAATAGAAACAGGCTGATCCACCCTAAATTCGTTTATAGCCACAGACTCCTTA

*V. albimarginata* AAAAACGCAATAGATACGGGCTGATCTGTTATTAACCCATACATGGGAACTGACCCTCTC

*V. louti* AAAACCACAATAGACACCGGCTGATCCCTCATTAACCCATATATGGGAACCGATCCTCTC

*P. leopardus* AACTATTCTTCAGAAACAGGTTGAACGTCACTCAACCCCTACATAGCAACAGACCCCCTC

*P. areolatus* AGCTATTCTTCAGAAACAGGCTGAGCTTCACTTAACCCTTATATAGCAACAGACCCTCTT

*E. radiosum* AAGAACACGTCAGAAACTGGTTGATCAAATCTTGGACTTTATATAGCCACGGACCCCTTG

*P. sieboldi* AAGAAACCATGGGACACGGCCTGGGTCCACCTTACACCCTATATGGGCGTCGACCCTCTT

*E. armatus* AAAAATTTTTCAGAGACAGGCTGATCCTCCCTTAACCCCTATATGGCAACAGATGCTCTC

*R. oxyrhynchus* GAAAACCTGACAGAAGCAGGTTGATCTTGTCTTAACCAATTCATAGCAACAGACCCCCTC

*K. cinerascens* AAAAACCTCTCAGAAACCGGCTGATCATGCCTTAACCCTTACATAGCAACAGACCCCCTT

*T. chatareus* AAAAACCTATCAGAAACAGGCTGATCCTCTCTAGGCCCTTACATAGCCACAGACCCTCTC

*D. berycoides* AAAAACCTCTCAGAAACAGGCTGAACTTCTCTTAACCTTTACATAGCAACAGACCCCCTC

*H. typus* AAAAACCTATCAGAAACTGGCTGAACTTCCCTTAACTTGTATTTAGCAACCGACCCCCTC

*M. argenteus* AAAAATCTATCAGAAACAGGGTGGTCATCCCTCAACACCTATATAGCAACAGACCCCCTA

*S. chuatsi* AAAAACCTGTCAGAGACAGGGTGATCCTCTCTTAACCTCTACATGGCAACAGACCCCCTC

*O. fasciatus* AAAAACCTTTCAGAAACCGGCTGATCCTGCCTCAACCTTTATATAGCTACAGACCCACTT

*P. trilineatum* AAAAACATTTCAGAGACGGGATGGTCCTTCCTCAGCCCTTACATAGCCACAGACCCCCTC

*M. salmoides* AAAAATCTATCAGAGACTGGCTGGTCCTCTCTTAACCTCTACATGGCCACAGACCCCCTT

*P. tile* AAAAACCTCTCAGAAACAGGCTGATCCTGCCTTAATCCATATATGGCAACCGACCCCCTA

*L. argentimaculatus* AAAAACCTTTCAGAAACAGGCTGATCTTGCCTCAACCCCTACATAGCAACAGACCCCTTA

*E. struhsakeri* AAAAATTTGTCAGAGACGGGGTGATCCGCCCTCAACCCCTACATAGCAACAGATCCCCTC

*B. albus* AAAAACTTAACAGAGGTCGGAATATCTTGTCTAAACCTCTATATGGCAACAGACACCATC

*C. auripes* GAAAATATGTCAGAAGTAGGGTGGTCGCACCTCAGCACCTACATGGCAACCGACCCCCTT

*C. melampygus* AAAAACCTTTCTGAAACAGGCTGATCCTGCTTAAACAACTACATAGCAACCGACGCCTTA

*L. calcarifer* ACCTGTCTAGCTGAAACCAGCTGATCCACCCTTAACTCTTACGTAGCAGTTGACCCCATC

*S. maena* CCTAATTTGTCAGAGACCGGCTGATCGCACCTCAGCCTCTACTTAGGCACAGACCCTCTT

*P. auriga* ACTAACTTCTCAGAAACCGGCTGAACCTCTCTCAACCCCTACCTAGCCACTGACCCTCTC

*C. lucidus* AAAAACCTATCGGAAACAGGGTGAACTTCTCTTAATCTTTATATAGCAACAGACCCCCTC

*S. sihama* AAAAACCTCTCAGAAGTGGGCTGGACGACCCTGAACCTTTATATGGCCACAGATCCCTTG

*C. loricula* AAAAACCTCTCAGAGACAGGATGATCCTGCTTAAGCCCATACCTAGCAACCGATCCCCTT

*A. trutta* GCAAGCCTGTCAGAAGCAGGCTGAACATCCCTTAACCTTTACATAGCAACTGATCTGCTG

*H. gemma* CAATCAACAACAGAAACAGGCTGAATGAGTTTAAATTCTTATATGATAATTGACTCCATC

*P. flavescens* AAAAATATGTCAGAGATCGGCTGGTCGAGCCTGGGGCTTTATATAGCAACAGATCCCCTC

*P. macrolepida* AAAAATGCATCAGAGACCGGGTGGTCTAGCTTAAACTCCTACATGGCCACAGACCCCTTA

*S. canadensis* AAAAATATGTCTGAAACCGGCTGGTCAAGTCTAGGACTTTATATAGCAACAGACCCCCTC

*A. rogaa* TCAACACCCCTCTTAGTCCTTACATGTTGACTACTTCCCTTAATAATTCTAGCAAGCCAA

*C. argus* TCCACACCCCTCTTAGTCCTCACATGTTGACTACTCCCCCTTATGATTCTAGCAAGTCAA

*C. sonnerati* TCTACCCCCCTTCTGGTTCTCACATGCTGATTACTCCCTTTGATGATCTTGGCAAGTCAA

*E. fuscoguttatus* TCTACACCCTTGTTAGTTCTCACATGCTGGCTTCTCCCCCTAATAATTCTAGCAAGCCAA

*E. coioides* TCCACACCTCTACTGGTTCTCACATGCTGACTCCTCCCCCTAATAATCCTAGCAAGCCAA

*E. bruneus* TCTACACCTCTATTAGTCCTCACATGCTGACTCCTCCCCCTAATAATCCTAGCAAGTCAA

*E. moara* TCTACACCTCTATTAGTCCTCACATGCTGACTCCTCCCCCTGATAATCCTAGCAAGCCAA

*E. lanceolatus*  TCCACACCCTTACTAGTCCTCACATGCTGACTCCTCCCCCTAATAATCTTAGCAAGCCAA

*A. leucogrammicus*  TCCACACCTCTACTAGTCCTCACATGCTGGCTTCTCCCCCTAATAATCCTAGCAAGCCAA

*C. altivelis* TCTACCCCCCTACTAGTTCTTACATGCTGGCTCCTTCCCTTAATAATCCTAGCAAGCCAG

*E. epistictus* TCTACACCCCTACTAGTCCTTACATGCTGACTCCTTCCCCTGATAATCCTAGCAAGTCAA

*E. octofasciatus* TCTACACCTCTATTAGTCCTTACATGCTGACTCCTTCCCCTGATAATTCTAGCAAGCCAA

*E. septemfasciatus* TCTACCCCTCTATTAGTCCTTACATGCTGACTCCTTCCCCTAATAATTCTAGCAAGCCAA

*T. dermopterus* TCCACGCCCCTACTAGTCCTTACATGCTGGCTCCTCCCTTTAATGATCCTAGCAAGCCAA

*E. awoara* TCTACACCCCTGCTTGTTCTTACATGCTGGCTTCTTCCCCTTATAATCCTAGCAAGCCAA

*E. akaara* TCTACACCCCTACTAGTTCTTACATGTTGACTTCTCCCCCTTATAATTCTAGCAAGCCAA

*E. trimaculatus* TCCACCCCTCTACTAGTTCTCACATGCTGACTCCTCCCTCTTATAATTTTAGCAAGCCAA

*E. areolatus* TCTACCCCTCTATTGGTCCTTACATGCTGGCTTCTCCCTTTAATAATTCTAGCAAGTCAA

*V. albimarginata* TCCACCCCTCTTTTAGTCCTAACCTGCTGACTTTTACCTCTTATAGTCCTAGCGAGCCAA

*V. louti* TCCACCCCTCTTTTAGTCCTGACCTGCTGACTACTTCCTCTTATAATTCTAGCAAGCCAA

*P. leopardus* TCCACTCCTCTTCTGGTCCTAACCTGCTGACTCCTCCCACTAATAATTCTTGCCAGCCAA

*P. areolatus* TCCACCCCTCTCCTGATCCTAACCTGCTGACTTCTTCCGTTAATAATCCTCGCTAGTCAA

*E. radiosum* TCCACCCCCCTTCTTATCCTCACCTGCTGACTCTTGCCCCTTATAATTCTGGCCAGCCAG

*P. sieboldi* TCGAGCCCTCTCCTTGTCCTATCGTGCTGGCTTCTTCCCCTTATGATTATTGCAAGCCAG

*E. armatus* TCAACCCCCCTCCTAGTCCTCACTTGCTGACTTTTACCACTAATAATTCTTGCAAGTCAA

*R. oxyrhynchus* TCAACCCCCCTCCTAGTTCTCACATGCTGACTTCTACCACTAATAATCCTTGCAAGCCAA

*K. cinerascens* TCAACCCCTCTCCTGGTCCTCACCTGCTGGCTTCTTCCCTTAATAATCCTTGCAAGTCAA

*T. chatareus* TCAACACCTCTCTTAGTCCTAACCTGCTGACTTCTCCCCCTCATAATTCTCGCAAGCCAA

*D. berycoides* TCAACCCCCCTCTTAATCCTAACCTGTTGACTACTCCCCCTTATAATCCTTGCAAGCCAA

*H. typus* TCAACCCCTCTCCTAGTTCTTACCTGTTGACTACTACCCCTCATAATCCTTGCAAGCCAA

*M. argenteus* TCCACCCCCCTTTTAGTCCTCACCTGTTGACTTCTACCTCTCATAATCCTTGCCAGCCAA

*S. chuatsi* TCTACCCCCCTGCTAGTCCTCACCTGCTGGCTTTTACCCCTCATAATTCTTGCTAGCCAA

*O. fasciatus* TCAACCCCCCTCCTAGTTCTTACTTGCTGACTACTACCTCTAATGATTCTTGCAAGCCAA

*P. trilineatum* TCCACCCCCCTACTAGTCTTAACCTGCTGACTTCTTCCCCTCATGATTCTTGCTAGTCAA

*M. salmoides* TCCACTCCCCTCCTAGTACTAACTTGCTGACTCCTCCCACTCATAATTCTTGCAAGCCAA

*P. tile* TCTACCCCTCTCTTAGTCCTTACCTGCTGACTCCTTCCTCTTATAATTCTCGCCAGTCAA

*L. argentimaculatus* TCCACGCCCCTCCTAGTCCTAACATGCTGACTTCTGCCCCTTATAATTCTTGCCAGCCAA

*E. struhsakeri* TCCACTCCCCTTCTGGTCCTCACCTGCTGACTATTGCCCCTAATGATTCTTGCCAGCCAA

*B. albus* TCCACCCCCCTGCTAATCCTCACCACCTGACTGTTACCCCTCATAATTCTAGCCAGCCAA

*C. auripes* TCTACACCCCTCTTAATCCTAACCTGCTGACTCCTTCCCCTTATAATCCTCGCCAGCCAG

*C. melampygus* TCCACCCCTCTTTTAGTCCTCACCTGCTGACTTCTTCCCCTTATAATTCTTGCAAGCCAA

*L. calcarifer* TCAATCCCCCTCCTAATACTGACCTGCTGACTACTACCACTCATGATTCTGGCAAGCCAA

*S. maena* TCCACTCCCCTCCTAGTCTTAACATGCTGACTTCTTCCACTAATAATCCTCGCCAGCCAA

*P. auriga* TCCTCCCCTTTATTAATCTTAACCTGCTGGCTTCTTCCACTCATAATTCTGGCTAGTCAA

*C. lucidus* TCAACCCCCCTCCTAGTACTCACCTGCTGACTCCTCCCCCTCATAATCCTTGCAAGCCAG

*S. sihama* TCAACTCCCCTTCTTGTACTCACTTGCTGACTTCTCCCACTTATAATTTTGGCTAGCCAA

*C. loricula* TCCACCCCCCTCCTAGTCCTTACTTGCTGGTTGCTGCCCCTCATGATTCTCGCCAGCCAA

*A. trutta* TCAACCCCACTTCTCGTCCTAACCTGCTGACTGCTCCCGCTAATAATTTTAGCCAGCCAA

*H. gemma* TCCAGCCCCTTACTTGTCCTTACTTGCTGGCTCTTACCTTTAATAATTATTGCAAGTCAA

*P. flavescens* TCAACACCCCTTCTAGTTCTAACCTGTTGGTTACTCCCCCTAATAATCTTAGCAAGCCAA

*P. macrolepida* TCCACCCCCCTGCTTGTTCTTACCTGCTGGCTCTTACCCCTTATAATCCTAGCAAGCCAG

*S. canadensis* TCAACGCCTCTCTTAGTCCTTACTTGCTGGCTATTACCCCTAATGATCTTAGCAAGTCAA

*A. rogaa* AACCACACCGCAACAGAACCAATCAACCGACAACGCATATATATTACACTCCTAACGTCT

*C. argus* AATCACACCGCAACAGAGCCCATTAACCGACAACGAATATACATTACCCTCTTAACATCA

*C. sonnerati* AACCACACAGCAACAGAACCAGTCAATCGCCAACGAATATATATCACACTTCTAACATCC

*E. fuscoguttatus* AACCATACAGCAACAGAGCCAATCAACCGTCAACGCATATATATTTCACTACTTACATCC

*E. coioides* AACCACACAGCAACAGAACCAATTAACCGCCAGCGCATATATATTTCACTATTAACATCC

*E. bruneus* AACCACACAGCAACAGAACCCATTAACCGCCAGCGCATATATATTTCATTACTAACATCT

*E. moara* AACCACACAGCAACAGAACCCATTAACCGCCAACGCATATATATTTCATTACTAACATCT

*E. lanceolatus*  AACCACACAGCAACAGAACCAATCAACCGCCAGCGCATATACATTTCACTACTAACATCC

*A. leucogrammicus*  AACCACACAGCAGCTGAACCAATCAATCGCCAACGCATATACATCTCACTACTTACATCC

*C. altivelis* AACCACACAGCAATAGAACCAATTAACCGTCAACGCATGTACATTACACTACTTACATCC

*E. epistictus* AACCACACAGCAACAGAGCCAATTAACCGCCAACGTATATACATTACACTGCTAACATCC

*E. octofasciatus* AACCACACAACAACAGAACCAATCAACCGCCAACGTATGTATATTACACTACTAACATCT

*E. septemfasciatus* AGCCACATAACAACAGAACCAATCAATCGCCAACGTATGTATATTACACTACTAACATCT

*T. dermopterus* AACCACACAGCGACAGAACCAATCAACCGCCAACGTATATATATTACACTACTGACATCC

*E. awoara* AACCACACAGCAACAGAACCTATTAATCGCCAACGAATATACATCACATTACTAACATCC

*E. akaara* AACCACACAGCAACAGAACCTATCAATCGTCAACGCATGTACATTTCATTACTAACATCC

*E. trimaculatus* AATCACACAGCTACAGAACCAATTAACCGCCAACGTATATATATTACACTACTAACGTCT

*E. areolatus* AATCACACAGCAACAGAGCCAATCAATCGCCAACGCATATATATTATATTATTAACATCC

*V. albimarginata* AACCACACAACCCTTGAACCACTTAACCGCCAACGGATATATATTACACTCCTCGCATCC

*V. louti* AACCACACAACCTTAGAACCACTTAACCGCCAACGAATATACATTACACTTCTTACATCC

*P. leopardus* AACCACACAGCCCAAGAACCAATTAGCCGCCAACGAATATATATTACTCTTCTAACATCC

*P. areolatus* AATCACATAGCCCACGAACCAGCCAGCCGCCAACGAATATACATTTCCCTTCTAACATCC

*E. radiosum* AGCCACACAACCTCAGAGCCACTTAATCGCCAACGAATATATATTACACTCCTGACTTCC

*P. sieboldi* AACCACATATCATCCGAGCCCGTCCACCGACAGCGCCTGTACATTACTCTCATGATTAGC

*E. armatus* AACCACACAGCTCTTGAACCTTTAAATCGACAGCGAATGTATATTACGCTACTTACATCC

*R. oxyrhynchus* AACCACACAAGCTCCGAGCCAATTAACCGACAACGAATATATATCTCCCTTTTAACTTCC

*K. cinerascens* AACCACACAGCCCTGGAACCAATTAATCGACAACGAATGTACATCACCCTCCTGACATCC

*T. chatareus* AACCACATAACCCCCGAACCCATCAACCGACAACGAATATACATTACACTCCTAACTTCC

*D. berycoides* AACCACACAGCTCTCGAACCCCTTAACCGACAACGAACATACATTACTCTCCTGACATCC

*H. typus* AACCACACGGCCCTTGAGCCCATCAACCGGCAACGAATATACATCACCCTTCTAACATCC

*M. argenteus* AATCACACAGCCTCCGAACCAGTCAACCGCCAACGAATGTACATCTCTCTTCTTACATCA

*S. chuatsi* AACCACACAGCCCTGGAGCCCATCAATCGGCAACGAATATACATTACACTCCTGACGTCA

*O. fasciatus* AACCACACAGCCCTTGAACCCATTAATCGTCAACGAATATATATTACACTTCTAACATCC

*P. trilineatum* AACCATACAGCGCTAGAACCTATTAACCGCCAACGGATATACATCTCCCTTCTGACATCC

*M. salmoides* AATCATACAACCTCTGAGCCCATTAACCGTCAACGTATATTTATTACGCTCCTTACATCC

*P. tile* AACCACACAGCCCAGGAACCTATTAATCGCCAACGAATGTACATTACCCTCCTCACCTCC

*L. argentimaculatus* AATCACACAGCTTTAGAGCCCATTAATCGTCAACGAATGTACATCACCCTCCTCACCTCC

*E. struhsakeri* AACCATACGAATCTTGAACCAATCAACCGACAACGAATATATATTACACTCCTCACATCC

*B. albus* GCCCACCTATGCCAGGAACCACTCAACCGACAACGAGTGTACATCTCACTCCTAGCATCT

*C. auripes* AACCATACAAAACATGAACCTGTAAGTCGACAACGAATGTACATTACACTCCTCACATCC

*C. melampygus* AACCACACATCCTCAGAACCAATTAATCGCCAACGAATATACATCACACTACTGACCTCC

*L. calcarifer* AACCACTTAGCCCTAGAACCTGCTAACCGACAACGAACTTACATTTCCCTATTGACCTCC

*S. maena* AACCACACTGCCTCAGAACCTATTAATCGACAACGAATGTACATTACGCTCCTAACATCT

*P. auriga* AACCACACCGCCACAGACCCAGCTAACCGGCAACGAATATATATTACTCTCCTCACATCT

*C. lucidus* AACCACACAACTACTGAACCTATCAACCGACAACGAATATACATTACTCTCCTGACATCC

*S. sihama* AATCACACGGCACTGGAACCCCCTAACCGCCAACGTATGTACATTTCGCTCTTAACATCC

*C. loricula* AACCACTTGGCCTCTGAACCTATTGGTCGCCAACGAATATATATTACCCTACTAACAGCC

*A. trutta* AACCATACAGCCGCAGAACCTACTAATCGTCAACGAACGTACATTACCCTTCTAACCTCC

*H. gemma* CACCATACAGCAAATGAACCCATTAACCGCCAACGCTTGTATATTACTTTGCTGACCTCC

*P. flavescens* AATCATACAGCACTAGAACCCCTAAGCCGCCAACGAATATACATCACATTACTAACGTCC

*P. macrolepida* AATCATACAGCCTCAGAGCCCCTGAACCGTCAGCGAATGTATATTACCCTCCTGACATCC

*S. canadensis* AACCATACAGCATTAGAACCTTTAAACCGCCCACGTATATATATTACCCTTTTAACATCC

*A. rogaa* CTACAAATTTTTCTTATCATGGCTTTCGGTGCTACTGAGGTAATTATATTCTACATTATA

*C. argus* CTACAAATTTTTCTTATTTTAGCTTTCGGTGCAACCGAAGTAATTATGTTCTACGTAATG

*C. sonnerati* CTGCAAATCTTCCTTATCCTAGCTTTCGGCGCAACCGAAGTAATCATGTTTTATGTAATG

*E. fuscoguttatus* CTCCAAATCTTCCTCATTCTAGCTTTCGGCGCAACTGAAGTTATTATATTTTACATTATA

*E. coioides* CTCCAAATCTTCCTTATCTTAGCCTTCGGGGCAACTGAAATAATTATGTTTTACGTCATA

*E. bruneus* CTCCAAATCTTCCTCATTCTGGCCTTTGGCGCAACTGAGGTGATCATATTTTATATTATA

*E. moara* CTCCAAATCTTCCTCATTTTAGCCTTTGGCGCAACCGAGGTAATCATATTTTACATTATA

*E. lanceolatus*  CTCCAAATCTTCCTCATTTTAGCCTTCGGAGCAACCGAAATAATCATGTTTTATATTATA

*A. leucogrammicus*  CTCCAATTTTTTCTTATCCTGGCTTTTAGTGCAACTGAACTAATTATGTTTTATGTTATA

*C. altivelis* CTCCAGATCTTCCTTATTTTAGCCTTTGGCGCAACCGAAATAATCATATTTTACATTATA

*E. epistictus* CTACAAATCTTCCTCATTCTAGCCTTTAGCGCAACCGAGGTTATTATGTTTTATGTTATA

*E. octofasciatus* CTTCAAATTTTCCTCATTCTAGCCTTTGGCGCAACCGAAGTAATCATGTTCTACGTCATG

*E. septemfasciatus* CTTCAAATTTTCCTCATTTTAGCCTTTGGCGCAACCGAAGTAATCATGTTCTACGTAATG

*T. dermopterus* CTTCAAATTTTCCTCATCTTAGCCTTTGGTGCAACTGAAGTAATTATGTTTTACGTCATG

*E. awoara* CTTCAAATTTTCCTCATTTTAGCCTTCGGCGCAACCGAAGTAATCATGTTTTATGTTATA

*E. akaara* CTTCAAATTTTCCTCATTCTAGCCTTCGGCGCAACCGAAGTAATCATATTTTATGTTATA

*E. trimaculatus* CTTCAGATCTTCCTTATTCTAGCCTTCGGCGCAACTGAGTTAATTATATTTTATGTAATA

*E. areolatus* CTTCAAATTTTCCTTATTTTAGCCTTCGGTGCAACCGAATTAATTATATTCTACGTTATA

*V. albimarginata* CTCCAAATTTTCCTTATTATAGCTTTTGGTGCCACAGAAGTAATTATATTTTATGTAATA

*V. louti* CTCCAAATTTTTCTTATTTTGGCTTTTGGTGCCACAGAAGTAATCATATTCTATGTAATA

*P. leopardus* TTACAAATTTTCCTAATTATAGCCTTCAGTGCCACCGAAATTATTATGTTTTATGTTATA

*P. areolatus* TTACAAATTTTCCTAATTATGGCCTTCAGTGCCACTGAAATTATTATGTTCTACGTTATA

*E. radiosum* CTTCAGTTTTTTCTTATTTTAGCCTTTGGCGCCACCGAGGTAATCATATTTTACGTAATA

*P. sieboldi* CTCCAATCCTTTCTCATCCTTGCCTTCAGTGCCACTGAGGTTATCATATTTTATGTCATA

*E. armatus* CTACAAGCCTTCTTAATTATGGCCTTTAGCGCCACCGAGGTAATCTTATTTTATGTTATG

*R. oxyrhynchus* CTCCAAATCTTCCTAATCATAGCCTTTGGAGCCACCGAGATTATTATGTTTTATGTTATA

*K. cinerascens* CTACAAGCCTTCCTCATTATGGCCTTCAGCGCTACCGAAATCATTATGTTTTATGTTATA

*T. chatareus* CTCCAATTCTTCCTAATCTTGGCCTTTAGTGCTACAGAAATTATTATGTTCTACGTCATA

*D. berycoides* TTACAGATCTTTCTGATCCTAGCCTTCAGCGCTACCGAAATTATTATATTCTATATTATA

*H. typus* CTGCAAGCTTTCCTGATTATAGCCTTCAGTGCCACGGAAATTATCATGTTCTACGTAATA

*M. argenteus* CTACAGGTCTTCCTAATCATAGCCTTTAGTGCTACAGAAATTATTATGTTCTACGTTATA

*S. chuatsi* TTACAAATTTTCCTAATCTTAGCCTTCAGTGCAACCGAAATTATTATATTTTACGTCATA

*O. fasciatus* CTACAACTCTTCCTAATCATAGCCTTTAGTGCCACCGAAATCATCATGTTCTACATCATG

*P. trilineatum* CTGCAAATTTTCCTAATTATGGCTTTTGGCGCCACTGAAATCATTATGTTTTATGTTATA

*M. salmoides* CTACAAGCCTTCCTAATTATGGCCTTTGGCGCCACCGAAATTATTATGTTTTATGTTATA

*P. tile* CTACAAATTTTCCTAATTATAGCCTTTGGTGCCACTGAGATTATTATGTTCTATATCATG

*L. argentimaculatus* CTACAAATCTTCCTAATTATGGCTTTTGGGGCAACCGAAATCATTATATTCTACGTCATA

*E. struhsakeri* CTACAAATTTTCCTAATTATAGCCTTCGGCGCTACAGAAATTATTATGTTCTATGTCATG

*B. albus* CTACAATTCTTCCTAATCTTAGCCTTTAGCGCCACCGAAATTATTATATTTTACATCATA

*C. auripes* CTGCAAATCCTCCTAATCATGGCTTTCGGTGCCACCGAACTTATGATGTTCTACGTAATA

*C. melampygus* CTTCAGTTTTTCCTTATTCTAGCTTTCGGAGCGACTGAAGTAATTATATTTTATGTTATG

*L. calcarifer* CTACAATTCTTCCTGATCCTAGCATTTAGCGCCACAGAAATAGCTTTATTTTACATCGCA

*S. maena* CTACAGTTCTTCTTGATTCTAGCCTTCGGGGCCACCGAATTGATTATATTTTATGTTATG

*P. auriga* TTACAATTTTTCCTAATCCTAGCCTTTGGCGCAACCGAGGCTATCATATTTTACATCATA

*C. lucidus* CTACAAATCTTCCTTATCTTAGCCTTCGGAGCCACCGAAATAATTATGTTTTACGTAATG

*S. sihama* CTTCAATTCTTCCTCATCCTAGCATTCAGCGCAACAGAAATTATCATGTTTTACATTATG

*C. loricula* CTACAAATCTTTTTAATCATAGCATTTGGGGCTACTGAAATTATTATATTTTATGTTATA

*A. trutta* CTACAACTTTTCCTAATCCTAGCCTTTGGAGCCACTGAAGTCATCATATTCTACGTCATA

*H. gemma* TTACAGTTTTTCCTTATCTTAGCATTTAGTGCTACCGAACTAATTATGTTTTACATTATA

*P. flavescens* CTTCAATTTTTTCTTATCCTGGCTTTTAGTGCTACAGAAGTAATTATATTTTATGTTATA

*P. macrolepida* CTTCAGTTTTTCCTTATCCTCGCCTTCAGCGCCACAGAAGTAATTATATTTTATGTCATA

*S. canadensis* CTCCAATTCTTTCTTATTCTGGCTTTCAGTGCCACTGAAGTAATCATATTTTATGTCATA

*A. rogaa* TTCGAAGCAACCCTTATCCCGACTCTAATTATTATTACCCGCTGAGGCAACCAAACAGAA

*C. argus* TTTGAAGCAACTCTCATCCCAACCTTAATTATCATTACTCGCTGAGGAAATCAAACAGAA

*C. sonnerati* TTTGAAGCTACACTCATCCCAACACTAATCATTATTACCCGCTGAGGTAATCAGACAGAA

*E. fuscoguttatus* TTTGAAGCAACTCTCATCCCAACTCTAATCCTTATTACCCGATGAGGGAATCAAACAGAA

*E. coioides* TTTGAAGCAACTCTTATCCCAACCCTGATTCTCATCACCCGATGAGGTAATCAAACAGAG

*E. bruneus* TTTGAAGCAACTCTTATCCCAACCCTAATCCTCATTACCCGGTGAGGAAACCAAACAGAA

*E. moara* TTTGAAGCAACCCTTATCCCAACCCTAATCCTCATTACCCGGTGAGGAAACCAAACAGAA

*E. lanceolatus*  TTTGAAGCAACTCTCATCCCAACCTTAATCCTTATTACCCGATGAGGTAACCAAACAGAA

*A. leucogrammicus*  TTTGAAGCAACCCTTATCCCCACCTTAATCCTTATTACCCGCTGAGGAAATCAAAAAGAA

*C. altivelis* TTCGAAGCAACCCTCATCCCAACCCTAATTCTCATCACCCGATGAGGAAACCAAACAGAA

*E. epistictus* TTTGAAGCAACCCTTATCCCCACCCTAATCCTCATTACTCGATGAGGTAACCAAACAGAA

*E. octofasciatus* TTTGAAGCCACCCTTATTCCAACCCTCATCCTTATTACTCGATGGGGAAATCAAACGGAA

*E. septemfasciatus* TTTGAAGCCACCCTTATTCCAACCCTTATCCTTATTACTCGGTGGGGAAATCAGACGGAG

*T. dermopterus* TTCGAAGCAACCCTCATCCCAACCTTAATCCTTATCACCCGGTGGGGTAATCAAACAGAA

*E. awoara* TTTGAAGCAACTCTTATCCCAACCCTAATTCTCATCACTCGATGGGGCAATCAAACAGAA

*E. akaara* TTTGAAGCAACTCTTATCCCAACCCTAATCCTCATCACTCGATGAGGTAATCAAACAGAA

*E. trimaculatus* TTTGAATCAACCCTCATCCCAACCCTAATCCTTATCACCCGGTGGGGTAATCAGACAGAG

*E. areolatus* TTTGAGTCCACCCTTATCCCAACCCTAATCCTCATTACTCGATGGGGGAATCAAACAGAA

*V. albimarginata* TTTGAAGCAACTTTAATCCCAACTCTAATGATTATTACACGATGAGGTAATCAAACAGAA

*V. louti* TTTGAAGCAACTTTAATCCCCACTCTGATAATTATTACACGATGAGGTAATCAGACAGAA

*P. leopardus* TTTGAAGCTACCCTAATCCCAACCCTAATTCTTATCACCCGGTGAGGAAATCAGACTGAA

*P. areolatus* TTTGAAGCTACCCTGATTCCAACATTAATTCTTATCACCCGATGAGGTAACCAAACTGAA

*E. radiosum* TTTGAAGCCACTCTAATTCCCACATTGATTATTATCACCCGGTGAGGTAATCAAACAGAG

*P. sieboldi* TTCGAGGCAACGCTGATTCCTACGCTTTTCCTCATCACCCGCTGAGGAAACCAGACAGAA

*E. armatus* TTTGAAGCCACCCTTATCCCAACCTTGATTCTTATTACTCGGTGAGGTAACCAAGCAGAA

*R. oxyrhynchus* TTTGAAGCCACCCTTATTCCAACGCTAATCCTCATCACACGGTGAGGAAACCAGACAGAA

*K. cinerascens* TTTGAAGCCACCCTTATCCCAACCCTCATCCTCATTACCCGCTGAGGTAATCAAACAGAA

*T. chatareus* TTTGAAGCAACCCTCATTCCAACCCTCATCATTATTACCCGATGAGGTAATCAAACAGAA

*D. berycoides* TTTGAAGCCACCCTAATCCCAACCTTAATTATTATTACCCGATGAGGCAACCAAACGGAG

*H. typus* TTTGAGGCCACCCTAATCCCCACCTTAATTCTCATCACACGATGGGGAAACCAAACAGAA

*M. argenteus* TTCGAAGCGACCCTAATCCCAACACTATTCCTCATCACCCGATGAGGAAACCAAACAGAA

*S. chuatsi* TTTGAAGCTACTCTTATCCCAACCCTAATTCTAATCACCCGATGAGGCAATCAAACAGAA

*O. fasciatus* TTTGAAACTACCCTCATTCCCACCCTAATCCTCATCACCCGCTGAGGAAACCAGACAGAA

*P. trilineatum* TTTGAAGCCACCCTCATTCCCACCCTTATTCTCATTACTCGCTGAGGGAACCAAACAGAA

*M. salmoides* TTTGAAGCCACCCTCATCCCCACCTTGTTTCTCATCACCCGATGAGGTAACCAAACAGAA

*P. tile* TTTGAAGCAACCCTCATCCCTACGCTATTTCTCATCACCCGCTGAGGAAACCAAACAGAG

*L. argentimaculatus* TTCGAAGCTACACTTATTCCCACACTCTTCCTCATTACCCGTTGAGGGAACCAAACAGAA

*E. struhsakeri* TTTGAAGCCACCCTTATTCCCACACTGATCCTCATCACCCGATGAGGCAACCAAACCGAA

*B. albus* TTTGAAGCCACCCTTATTCCCACCCTATTCCTAATCACCCGATGGGGTAACCAAACAGAG

*C. auripes* TTCGAAGCCACTCTAATCCCCACCCTAATTATTATTACCCGATGAGGAAACCAGAAAGAA

*C. melampygus* TTTGAAGCCACCCTTATCCCCACCTTAATTATTATTACGCGCTGAGGAAACCAAACTGAA

*L. calcarifer* TTCGAAGCCACCCTCATCCCCACCCTGATTATTATTACTCGATGAGGTAACCAAGCAGAA

*S. maena* TTTGAAGCAACACTGATCCCCACACTAATCCTAATCACCCGATGAGGAAATCAGGCAGAA

*P. auriga* TTTGAAGCAACCCTAATTCCAACACTAATCCTTATTACCCGATGAGGAAATCAAACAGAA

*C. lucidus* TTTGAAGCCACTCTGATTCCAACCCTGTTTCTCATCACCCGCTGAGGTAATCAAGCAGAG

*S. sihama* TTCGAGGCCACCCTGATTCCCACACTGATGCTCATTACCCGCTGAGGTAACCAAGCAGAA

*C. loricula* TTTGAGGCCACACTATTACCAACTCTCGTGCTAATTACCCGATGAGGTAACCAAACAGAA

*A. trutta* TTTGAAGCCACTTTAATCCCAACATTAATTATTATCACCCGCTGAGGAAACCAGACAGAA

*H. gemma* TTTGAAGCTACTTTACTACCTACCCTTGTAATTATTACACGATGGGGAAACCAAGCAGCG

*P. flavescens* TTTGAAGCCACCCTCATCCCCACCCTAATTATTATCACCCGCTGAGGAAACCAAACAGAG

*P. macrolepida* TTTGAAGCTACCCTCATCCCCACACTTATTATTATTACCCGTTGAGGGAATCAGACAGAA

*S. canadensis* TTTGAGGCCACCCTTATTCCCACCCTAATCATTATTACCCGATGGGGTATCCAAACGGAA

*A. rogaa* CGCCTTAACGCAGGAGTTTACTTTTTATTTTACACCCTAGCAGGATCCTTACCCCTACTT

*C. argus* CGCCTTAATGCAGGAGTTTATTTCTTATTTTATACTTTAGCAGGATCTCTACCTCTACTT

*C. sonnerati* CGACTCAACGCAGGCGTTTATTTCCTATTCTACACACTAGCAGGCTCCCTCCCACTTCTA

*E. fuscoguttatus* CGCCTTAACGCAGGTATTTACTTCTTATTTTACACCTTAGCAGGCTCCCTCCCCCTACTT

*E. coioides* CGTCTTAATGCAGGAGTTTACTTCTTATTCTACACCTTAGCAGGCTCCCTCCCCCTACTC

*E. bruneus* CGCCTTAACGCAGGGGTTTACTTCTTATTCTACACCCTGGCAGGCTCTCTCCCCTTGCTT

*E. moara* CGCCTTAACGCAGGGGTTTACTTCTTATTCTACACCCTAGCAGGCTCTCTCCCCTTACTT

*E. lanceolatus*  CGCCTTAACGCAGGGGTTTACTTCCTATTTTACACCTTAGCAGGCTCCCTCCCCCTACTC

*A. leucogrammicus*  CGCCTTAACGCAGGCGTCTACTTCTTATTCTACACCCTAGCAGGTTCCCTCCCCTTACTC

*C. altivelis* CGCCTTAACGCAGGAGTTTACTTCCTATTCTATACCCTAGCAGGCTCCCTCCCCCTACTT

*E. epistictus* CGCCTCAACGCAGGGGTTTATTTTTTATTTTACACCCTAGCAGGTTCCCTCCCCTTGCTT

*E. octofasciatus* CGTCTTAACGCAGGAGTCTACTTTTTGTTTTACACCCTAGCAGGCTCCCTCCCCCTACTT

*E. septemfasciatus* CGTCTTAACGCAGGAGTCTATTTTTTGTTTTATACCCTAGCAGGCTCCCTTCCCCTACTT

*T. dermopterus* CGTCTTAACGCAGGGGTTTACTTCTTATTTTACACCCTAGCAGGCTCCCTCCCCCTACTA

*E. awoara* CGCCTCAACGCAGGAGTCTACTTCCTATTTTATACCTTGGCAGGTTCCCTCCCCTTACTT

*E. akaara* CGCCTCAACGCAGGGGTCTACTTCTTATTCTATACATTGGCAGGCTCCCTTCCCCTACTT

*E. trimaculatus* CGCCTCAATGCAGGAGTTTACTTCCTATTCTATACACTGGCAGGCTCCCTTCCCCTACTC

*E. areolatus* CGTCTTAATGCAGGGGTTTACTTCTTATTTTATACCCTAGCAGGCTCCCTTCCTCTTCTT

*V. albimarginata* CGCCTTAATGCAGGTGTCTACTTTTTATTTTATACTCTTGTAGGCTCCCTACCACTTCTA

*V. louti* CGTCTTAATGCAGGTGTCTATTTTTTATTTTATACTCTTGTAGGTTCACTACCACTTCTC

*P. leopardus* CGTCTCAACGCAGGCATTTATTTTCTATTTTATACCCTAGCAGGCTCACTACCCCTCCTC

*P. areolatus* CGCCTTAACGCAGGTGTTTACTTCCTATTTTACACTTTAGCGGGCTCACTACCCCTCCTC

*E. radiosum* CGGCTTAACGCAGGGGTTTATTTTCTTTTCTATACCCTAGCGGGATCACTTCCACTACTA

*P. sieboldi* CGCCTAAACGCGGGGACCTATTTCCTCTTCTACACCCTGGCAGGCTCCCTCCCCCTTCTT

*E. armatus* CGACTAAACGCAGGTACATATTTTTTATTTTACACCTTAGCAGGATCACTCCCACTACTA

*R. oxyrhynchus* CGCCTTAACGCCGGCACTTACTTCCTATTTTATACCCTAGCGGGCTCCCTCCCCCTTCTA

*K. cinerascens* CGCCTCAACGCTGGAACTTATTTTCTCTTTTACACACTAGCAGGGTCCCTACCCCTTCTT

*T. chatareus* CGCCTCAATGCAGGAACCTATTTCTTATTCTATACCCTAGCAGGCTCTCTTCCCCTCCTA

*D. berycoides* CGTCTTAACGCAGGCACCTATTTCCTGTTTTATACCTTAGCGGGCTCCCTCCCCCTACTA

*H. typus* CGGCTTAATGCAGGCACTTACTTCCTATTCTACACCTTAGCAGGTTCCCTGCCTCTCCTA

*M. argenteus* CGCCTTAATGCAGGCACTTATTTCCTGTTCTACACCCTGGCGGGATCACTACCCCTCCTC

*S. chuatsi* CGACTCAATGCAGGGACCTACTTCTTATTCTACACTTTAGCAGGCTCTCTCCCGCTTCTT

*O. fasciatus* CGCCTCAACGCCGGCACTTACTTTCTTTTCTACACACTAGCAGGGTCCCTGCCCCTCCTA

*P. trilineatum* CGACTCAATGCAGGCACTTACTTCTTATTTTATACCCTAGCAGGCTCACTTCCCCTTCTC

*M. salmoides* CGCCTTAATGCAGGCACCTACTTCCTATTTTATACCCTAGCGGGCTCACTCCCACTTCTC

*P. tile* CGACTAAACGCCGGCACTTATTTCCTATTTTACACGCTAGCAGGATCACTTCCTCTTCTT

*L. argentimaculatus* CGTCTTAATGCTGGCACCTACTTCCTATTCTACACCCTGGCAGGATCCCTCCCCCTCCTC

*E. struhsakeri* CGACTTAACGCAGGCACCTACTTTTTATTCTACACCCTAGCAGGCTCATTGCCCCTTCTC

*B. albus* CGCCTTAACGCTGGCACTTACTTCCTATTCTACACCCTGGCAGGGTCTTTGCCACTTCTT

*C. auripes* CGCCTTAATGCAGGCATGTACTTCCTCTTCTACACTCTAGCAAGCTCCCTCCCACTACTT

*C. melampygus* CGTCTTAACGCAGGGACCTACTTCCTTTTCTACACTTTAGCAGGCTCCCTCCCCCTTCTT

*L. calcarifer* CGCATTACCGCAGGAGTTTACTTCCTATTCTACACCCTAATAGCCTCCCTCCCCCTACTT

*S. maena* CGGCTTAATGCAGGGACCTATTTCTTATTTTATACACTTGCTGGCTCACTCCCGCTTCTG

*P. auriga* CGACTTAATGCCGGAACTTATTTCTTATTTTATACTTTAGCCGGCTCCCTGCCGCTCCTT

*C. lucidus* CGACTTAACGCAGGCACTTATTTTTTATTTTATACTCTAGCCGGCTCACTCCCCCTACTT

*S. sihama* CGACTTAATGCCGGAACCTACTTCCTATTTTACACCCTTGCAGGCTCCCTCCCTCTCCTC

*C. loricula* CGACTAAGTGCAGGATTTTATTTCCTGTTCTACACCCTAGCAGCATCCCTCCCCCTCCTC

*A. trutta* CGCCTGAACGCGGGGACTTACTTCCTATTTTACACACTGGCTGGATCCCTCCCCCTACTT

*H. gemma* CGGTTAAACGCAGGAATCTATTTCCTGTTTTATACTCTTGCGGGATCTCTACCCCTTCTG

*P. flavescens* CGACTCAATGCAGGAGTCTACTTCCTATTTTATACACTAGCAGGGTCCCTTCCACTACTT

*P. macrolepida* CGGCTTAACGCAGGTATCTATTTCTTATTTTATACTCTCGCAGGATCACTTCCCCTGTTA

*S. canadensis* CGACTCAATGCAGCATTCTACTTCCTATTTTATACACTAGCAGGGTCCCTTCCACTGCTT

*A. rogaa* GTTGCCCTCCTACTCCTACAAAATCACACTGGCACACTCTCCCTATTTACACTCCCATTC

*C. argus* GTTGCACTTTTAGTTTTACAAAATTACACTGGCACACTTTCCCTATTTACACTTCCATTC

*C. sonnerati* GTTGCCCTGCTACTCCTACAAAACCACACTGGAACCCTATCCCTATTCACCCTCCCCTTC

*E. fuscoguttatus* GTTGCTCTTCTACTCTTGCAAAACTACACCGGAACACTCTCTCTATTTACTCTACCATTT

*E. coioides* GTTGCTCTCCTACTTCTACAAAATTACACCGGGACACTTTCTCTATTTACCCTACCATAC

*E. bruneus* GTTGCCCTTCTACTCCTACAAAATTACACCGGAACACTCTCCCTATTCACCCTACCATTC

*E. moara* GTTGCCCTTCTGCTCCTACAAAATTACACCGGGACACTCTCCTTGTTCACCCTACCATTC

*E. lanceolatus*  GTCGCTCTCCTGCTTCTCCAAAACTACACTGGAACACTCTCCCTATTTACCCTACCATTC

*A. leucogrammicus*  GTCGCCCTTCTACTACTACAAAACCACACCGGAACACTCTCCTTACTCACCCTACCATTC

*C. altivelis* GTTGCCCTTTTGCTTCTGCAAAACCACACCGGAACACTCTCCTTATTTACTCTACCATTT

*E. epistictus* GTTGCCCTCCTTCTCCTACAAAACTACACCGGAACACTTTCTCTTTTCACCCTACCATTC

*E. octofasciatus* GTTGCTCTCCTACTTCTACAAAATTACACTGGAACACTCTCTTTACTTACCCTACCATTC

*E. septemfasciatus* ATTGCTCTTCTACTTCTACAAAACTATACTGGAACACTCTCTTTACTTACCCTACCATTC

*T. dermopterus* GTTGCCCTTCTCCTCCTACAAAACTATACAGGGACACTCTCCTTACTAACTTTACCATAC

*E. awoara* GTTGCCCTCCTCCTCCTACAAAACTACACGGGGACACTCTCTCTATTCACCCTACCATTC

*E. akaara* GTCGCCCTTCTCCTCCTCCAAAACTACACCGGGACACTCTCCTTATTTACCCTACCATTC

*E. trimaculatus* ATTGCCCTTCTCCTCCTACAAAACTACACCGGAACACTCTCCCTACTCACACTTCCTTTC

*E. areolatus* GTCGCCCTCCTACTCCTACAAAACTATACCGGGACACTCTCCCTACTCACACTACCATTT

*V. albimarginata* GTTGCCCTGTTACTTCTCCAAAACTACACAGGTACACTCTCCATGCTTACACTCCAATAT

*V. louti* GTCGCCCTCTTACTCCTCCAAAACTACACGGGCACACTCTCCATGCTAACACTTCAATAC

*P. leopardus* GTCGCCCTCCTAGTACTCCAAAACCACTCAGGCACACTATCATTACTAACCCTCCCATAT

*P. areolatus* GTCGCCCTCCTAGTACTTCAAAACCACTCAGGCACACTATCACTATTAACCCTCCCATAC

*E. radiosum* GTCGCTCTCCTTCTCCTACAAAACAGTTCAGGCACTCTTTCTTTGATTACCCTTCAATTT

*P. sieboldi* GTGGCCCTCCTGCTATTGCAAAATACCACTGGCTCTCTCTCGATGCTGACCCTTCAATAC

*E. armatus* GTTGCCCTTCTTCTCCTTCAAAATAACACAGGGACCCTTTCACTCCTAACCCTACAATAC

*R. oxyrhynchus* GTTGCCCTTCTTCTCCTTCACAACGACACCGGGACCCTCTCACTTCTGGTACTCCAATAC

*K. cinerascens* GTTGCCCTCTTACTACTCCAAAACAACACCGGAACTCTCTCCCTACTGACCCTTCAATAC

*T. chatareus* GTCGCTCTCCTCCTCCTTCAAAATAGCACCGGGACTCTTTCCCTCATTACCCTTCAATAC

*D. berycoides* GTCGCCCTCCTTCTCCTTCTAAATAATACAGGCTCCCTCTCCCTCTTAACCCTTCAATAT

*H. typus* GTTGCCTTGCTACTCCTACAAAACAGTACGGGAACCCTCTCCCTCCTAACCCTGCAATAC

*M. argenteus* GTTGCCCTCCTGCTTCTTCAAAACAGTACAGGAACTCTGTCCCTACTAACCCTTCAATAC

*S. chuatsi* GTAGCCCTTCTACTTCTACAAAACAACACAGGCACTCTCTCCATACTTACCCTACAATAT

*O. fasciatus* GTCGCCCTCCTGCTCCTTCAAAATACCACCGGAACCCTCTCCTTACTAACCATTCAATAT

*P. trilineatum* GTTGCCCTTTTACTCCTACAAAATAGTACAGGGACCCTCTCCCTACTTACCCTTCAATAC

*M. salmoides* GTTGCCCTCCTGCTACTTCAAAACAGCACAGGCACCCTCTCCCTATTAACCCTTCAGTAC

*P. tile* GTCGCACTCCTTCTTCTCCAAAACAGCACCGGAACCCTCTCTCTCCTAACACTTCAATTC

*L. argentimaculatus* GTTGCGCTCCTCCTCCTTCAAAACAATACTGGAACCCTCTCCCTCCTTACACTACAATAC

*E. struhsakeri* GTTGCACTGCTTCTTCTTCAAAACAGTACAGGAACCCTTTCCCTACTAACCCTTCAGTAC

*B. albus* GTCGCACTGCTTCTACTTCAAAATACTACAGGAACCCTCTCCCTATTAACACTACAATAT

*C. auripes* GTTGCCCTCCTAATGCTACAAAAAAATGTTGGAACTCTATCTCTTCTTCCCGCCCCCCTT

*C. melampygus* GTCGCACTACTTCTTTTACAAAACAATACAGGCTCACTCTCACTACTCACCCTCCAATAC

*L. calcarifer* GTTGCCCTCCTCGTGCTCCAACACCATGCAGGATCTTTATCAATCCTAACAACACAATTC

*S. maena* GTTGCTCTTCTTATCCTCCAAAATAGCACAGGATCCCTTTCTTTACTTACCCTTCCATAT

*P. auriga* GTAGCTCTCCTCTTCCTTCAAAATACTGCAGGGTCCCTTTCATTCCTCACCCTCTCGTAC

*C. lucidus* GTTGCCCTCCTCCTTCTCCAAAATAGCACCGGGACTCTTTCTCTCCTAACTCTGCAATTT

*S. sihama* GTCGCACTCATTCTTCTTCAAAATAATCTCGGTACCCTCTCTCTCTTGACTGTGCAATTC

*C. loricula* ATTGCCCTACTACTTCTCCAAAACACTACGGGGACACTATCCCTACTAACCCTCCAATAC

*A. trutta* GTAGCCCTCCTATTAATACAAAACAGCACAGGGACCCTATCTCTCCTTACTCTTCAGTAC

*H. gemma* GTAGCGCTTCTTTTGCTTCAAAATCACCTAGGAACCCTTTCCCTATTAATCCTCCCATAT

*P. flavescens* GTTGCCCTCCTCCTCCTGCAAAACAGCACTGGTACCCTCTCCCTGCTTACCCTACAATTT

*P. macrolepida* GTCGCACTCCTTCTTCTTCAGAACAACGTCGGCACCCTCTCACTAGTCACCCTTCAATTC

*S. canadensis* AGTGCATTGCTCCTTCTACAAAATAGCACTGGTACACTCTCTATGCTTACCCTACAATTC

*A. rogaa* TCCCACCCCTTGCACCTATCATCAAACGCCGACAAACTCTGATGAGCAGGCTGCCTCATC

*C. argus* TCACACCCCTTACACCTCTCATCGAACACTGACAAACTCTGATGGGCCGCCTGCCTCCTT

*C. sonnerati* TCCCACTCCCTCCACCTTTCATCCTACGCTGATAAACTCTGATGAGCAGGATGTCTCCTA

*E. fuscoguttatus* TCCTGCCCTCTCCACCTTTCATCCTATACCGATAAACTATGATGAGCAGGCTGCCTACTA

*E. coioides* TCCTGCCCCCTTCACCTATCATCCTACACCGATAAGCTATGATGAGCGGGCTGCCTACTA

*E. bruneus* TCCCACCCCCTCCATCTCTCATCCTATACCGACAAACTATGATGAGCGGGCTGCCTACTA

*E. moara* TCCCACCCCCTCCATCTCTCATCCTACACCGACAAACTATGATGAGCGGGCTGCCTACTA

*E. lanceolatus*  TCCAGCCCCCTGCATCTCTCATCCTATGCCGACAAACTATGATGAACAGCCTGCCTACTG

*A. leucogrammicus*  TCCTCCCCCCTCCACCTATCATCCTACGCCGACAAGCTATGATGAGCAGGCTGTCTACTA

*C. altivelis* TCCTCCCCCCTACACCTTTCATCTTACAGCAGTAAATTATGATGACTAAGCTGCCTACTA

*E. epistictus* TCACACCCCTTTCATCTCTTATCCTATGCCGACAAACTTTGATGAGCGGGCTGCCTACTA

*E. octofasciatus* TCGCACCCCCTTCATCTGTCATCCAATGCCGACAAGCTTTGATGAGTAGGTTGTCTACTA

*E. septemfasciatus* TCACACCCCCTTCATCTATCATCTAATGCCGACAAGCTTTGATGAGTAGGCTGTCTACTA

*T. dermopterus* TCACACCCCCTTCACCTGTCATCCAATGCCGACAAGCTCTGATGAGCAGGCTGTCTACTG

*E. awoara* TCACACCCCCTCCACCTCTCATCTAACGGCGATAAACTATGATGAGCAGGCTGCCTACTA

*E. akaara* TCTCACCCTCTCCACCTCTCATCCAATGCTGACAAGCTATGATGAGCAGGCTGCCTACTA

*E. trimaculatus* TCCCACCCCCTCCACCTCTCATCCAATGCCGACAAACTATGATGAGCAGGCTGCCTACTA

*E. areolatus* TCCCCTTCCCTCCACCTCTCATCTAATGCTGATAAACTTTGATGAGCAGGTTGCCTACTA

*V. albimarginata* TCTCACCCCCTTCAACTTACCTCTAATGCTAGTAAGTTGTGATGAGCAGGTTGCCTTGTA

*V. louti* TCTTACCCCCTTCAACTTACCTCCAACGCCAGCAAATTATGATGAGCAGGTTGTCTTGTC

*P. leopardus* ACATGTCCACTCCAACTCTCTATAAACACCGACAAATTATGATGAGTAGGCTGTCTCATA

*P. areolatus* ACATGCCCGCCCCAACTCTCCCTAAACGCTGATAAACTATGATGAGCGGGTTGCCTAATA

*E. radiosum* ACAGACCCCTTTCAACTTACTTCTTTCGCAGACAAACTATGATGGGCAGGCTGCCTCCTA

*P. sieboldi* GCCAAACCCCTTGTCTATTCCAGCCATGCAGACAACTTTTGGTGGGCCGCATGTTTGCTT

*E. armatus* ACCAATCCCCTCCTGCTTTCAACTTATGCAGACAAACTATGATGAGCAGGCTGCTTACTG

*R. oxyrhynchus* TCCAACCCAATCCCTCTAACAACTTACGCTGACAAGTTATGATGAGCCGCCTGCTTACTG

*K. cinerascens* TCCAACCCAATCCCTCTTACAACCTACGCAGATAAACTATGATGAGCAGGCTGCCTCCTG

*T. chatareus* ACAAATGCCATTCAACTCTCGTCCTACGCAGACAAGCTATGATGAGCAGGTTGCCTACTA

*D. berycoides* TCTGACCCTCTTCAACTTGTGACCTATGCAGATAAACTCTGATGAGCAGGCTGTCTACTA

*H. typus* TCTGACCCAATTCAACTTTCCACCTACGCAGACAAACTATGATGGGCAGGCTGCCTATTA

*M. argenteus* TCCAACCCCCTCCTTCTCACCACCTACGCCGATAAACTATGATGAGCAGGCTGCCTGTTG

*S. chuatsi* TCCGACCCTATATTACTTTCAACCTACGCCGACAAACTCTGATGAGCAGGTTGTCTACTG

*O. fasciatus* TCCAGCCCCCTCCTGCTTACAACCTATGCAGACAAATTATGATGAGCTGGCTGCCTATTA

*P. trilineatum* TCAAATCCCGTTCCTCTAACCACATATGCAGACAAACTATGATGAGCAGGCTGCTTAGTG

*M. salmoides* TCCACCCCTCTTTCACTTACAACCTATGCTGACAAACTTTGGTGGGCCGGATGCCTACTA

*P. tile* TCCAACCCCCTTCAACTCACAACCTACGCAGACAAGCTGTGATGAGCCGGCTGTCTACTG

*L. argentimaculatus* GCCAACCCCCTTCAGCTCACAACCTATGCAGACAAATTATGGTGAGCGGGCTGTCTACTA

*E. struhsakeri* TCCAACCCCGCCCATCTTGTTACTTACGCAGACAAACTATGATGAGCAGCCTGTCTCCTG

*B. albus* TCAGAGCTTACAATATCTAACTCTTACTCATCCAAATTCTGATGAGTGGCCCTCATACTA

*C. auripes* CCAATTACAGGAATGCCTATAACAAGCTCAACCAAATTATGATGAGTGGCCTGCCTTTTA

*C. melampygus* TCCAACCCCATTCCTCTTTCCACCTATGCAGATAAACTCTGATGAGCAGGCTGTCTCTTA

*L. calcarifer* CTGTACCCAACCTACTACACATCCTACTCAAACAAACTATGATGGGCAGGATGTATCCTA

*S. maena* TCTCCCCCAATACAACTTTCCTTGTACAGCCACAAACTCTGGTGGGCCGCTTGCTTATTG

*P. auriga* TTAACCCCCCTCCAACTCCTAACTTACGGAGACAAGCTCTGATGAGCCGCCTGCTTTTTA

*C. lucidus* TCAGGCCCCACCATCCTAACTTCCTTTGCAGATAAACTCTGATGAGCAGGCTGTTTACTA

*S. sihama* TCTGACCCCGTTTCCATAACTACCACTGCAGACAAACTGTGATGAGCCGGATGTTTATTA

*C. loricula* TCCAACCCCCTACACCTCACCTCTTATGCCGACAAAATCTGATGAGCCAGCTGTCTACTG

*A. trutta* ACCCTCCCCCTTCAACTAACCTCCTACGCAGACAAATTATGATGAGCAGGCTGCCTTCTG

*H. gemma* TTTCCCCCATTAGAACTTTCTTCTTATGCAAGCAAACTATGATGAGTGGCCTGCCTTCTT

*P. flavescens* TCAGACCCCCTTCAACTTACCTCCTACGCAGATAAACTATGATGAGCAGGCTGTCTTCTA

*P. macrolepida* TCAGACCCACTTCAACTTACCTCTTTCGCGGACAAACTATGATGAGCAGGATGCCTCCTA

*S. canadensis* TCAGACCCACTACTACTCACTTCCTACGCAGACAAATTATGGTGGGCAGGCTGCCTTCTA

*A. rogaa* GCTTTCCTTGTAAAAATGCCACTATATGGCGTACACCTCTGACTACCAAAAGCACACGTT

*C. argus* GCCTTCCTAGTCAAAATGCCATTATACGGTGTTCATCTCTGATTACCAAAAGCACACGTC

*C. sonnerati* GCATTTCTTGTTAAAATACCACTATACGGCGTCCACCTTTGACTACCAAAAGCACACGTT

*E. fuscoguttatus* GCATTCCTTGTTAAAATACCACTATATGGTGTACACCTTTGACTCCCCAAAGCACACGTT

*E. coioides* GCATTCCTTGTTAAAATACCACTGTATGGCGTCCATCTCTGACTTCCTAAAGCACACGTT

*E. bruneus* GCATTTCTTGTTAAAATACCACTATATGGTGTTCACCTCTGACTCCCTAAAGCACACGTT

*E. moara* GCATTTCTTGTTAAAATGCCACTGTATGGTGTACACCTCTGACTCCCCAAAGCACACGTT

*E. lanceolatus*  GCATTCCTTGTTAAAATACCACTATACGGCGTCCACCTATGACTCCCTAAAGCACACGTT

*A. leucogrammicus*  GCATTCCTTGTTAAAATGCCCTTGTATGGTGTACACCTCTGACTCCCTAAAGCACACGTT

*C. altivelis* GCATTTCTTGTTAAAATACCACTATATGGTGTTCACCTCTGACTTCCCAAAGCACACGTT

*E. epistictus* GCATTCCTTGTCAAAATACCCCTATATGGGGTACATCTTTGACTTCCTAAAGCACACGTT

*E. octofasciatus* GCGTTTCTTGTTAAAATACCACTATATGGTGTACACCTTTGACTCCCAAAAGCACACGTT

*E. septemfasciatus* GCATTTCTTGTTAAAATACCACTATATGGGGTACACCTTTGACTCCCAAAAGCACACGTT

*T. dermopterus* GCATTTCTTGTTAAAATACCACTGTACGGCGTACACCTCTGACTCCCAAAAGCCCACGTT

*E. awoara* GCCTTCCTTGTCAAAATGCCCCTATACGGTGTACATCTTTGACTGCCCAAAGCGCACGTT

*E. akaara* GCATTTCTTGTTAAAATACCACTATACGGCGTTCATCTTTGACTACCCAAAGCACACGTT

*E. trimaculatus* GCATTTCTTGTTAAAATACCACTATACGGCGTCCATCTTTGACTACCCAAAGCACATGTT

*E. areolatus* GCATTTCTTGTTAAAATACCACTTTACGGCGTCCACCTTTGGTTACCAAAAGCACATGTT

*V. albimarginata* GCATTCTTAGTAAAAATACCTTTATATGGTGTACACCTTTGACTACCCAAAGCACACGTT

*V. louti* GCATTCTTAGTAAAAATACCTTTATATGGTGTCCACCTTTGACTGCCCAAAGCACACGTT

*P. leopardus* GCATTTTTAGTAAAAATACCCCTGTATGGAGTCCACTTATGGCTTCCCAAAGCCCACGTA

*P. areolatus* GCCTTCCTGGTCAAAATGCCGTTGTACGGGGTTCACTTATGACTCCCCAAGGCCCACGTA

*E. radiosum* GCGTTCTTAGTAAAAATACCTCTCTATGGAGTACACCTATGGCTGCCTAAAGCTCACGTA

*P. sieboldi* GCATTTTTAGTCAAAATACCACTTTACGGCGTCCATCTCTGGCTGCCTAAAGCCCACGTA

*E. armatus* GCCTTCCTAGTAAAAATACCACTTTATGGCGTCCACCTCTGACTTCCTAAAGCTCATGTA

*R. oxyrhynchus* GCATTCTTAGTTAAAATACCACTATACGGCGCCCACCTCTGACTCCCTAAAGCACACGTC

*K. cinerascens* GCATTCCTAGTAAAAATACCACTATACGGTGCCCACCTTTGACTTCCTAAGGCACATGTT

*T. chatareus* GCATTCCTAGTAAAAATACCCCTATATGGCGTTCACCTCTGACTCCCTAAAGCACATGTT

*D. berycoides* GCGTTTCTGGTGAAAATACCACTATATGGGGTACACCTCTGGCTCCCTAAAGCACACGTT

*H. typus* GCCTTCTTGGTAAAGATACCACTATACGGAGTCCACCTATGACTTCCCAAAGCACATGTT

*M. argenteus* GCCTTCCTAGTAAAAATACCACTTTATGGGGTCCATCTTTGACTTCCTAAAGCACACGTT

*S. chuatsi* GCTTTTCTAGTAAAAATACCTCTTTACGGAGTCCACCTCTGACTTCCTAAAGCACACGTT

*O. fasciatus* GCCTTTCTAGTAAAAATACCCCTATATGGTGCTCACCTTTGACTTCCCAAAGCCCATGTT

*P. trilineatum* GCATTCCTAGTCAAAATGCCCCTGTACGGAGTACATCTCTGACTTCCAAAGGCACACGTC

*M. salmoides* GCCTTCCTAGTTAAGATGCCACTTTACGGGGTCCATCTCTGGCTTCCTAAGGCACATGTA

*P. tile* GCCTTCCTAGTGAAAATACCCCTCTACGGCGTCCACCTTTGACTTCCCAAAGCCCACGTA

*L. argentimaculatus* GCCTTCCTAGTAAAAATACCACTCTACGGCGTCCATCTCTGACTTCCCAAAGCCCACGTA

*E. struhsakeri* GCATTCTTAGTGAAAATACCCCTCTATGGCGTTCACCTTTGACTCCCCAAGGCCCATGTC

*B. albus* GCATTTCTTGTAAAAATGCCACTATATGGAGCACACCTTTGACTCCCTAAAGCCCACGTT

*C. auripes* GCTTTCTTAGTAAAAATACCACTATACGGCGCCCACCTCTGACTCCCTAAAGCACACGTA

*C. melampygus* GCCTTCTTGGTAAAAATACCTCTGTACGGCGTCCACCTTTGACTTCCTAAAGCCCACGTT

*L. calcarifer* GCCTTCTTAGTCAAACTCCCCCTTTATGGAGTTCACTTATGACTCCCAAAAGCACACGTA

*S. maena* GCCTTCCTAGTTAAAATGCCACTGTATGGAATTCATCTCTGGCTTCCAAAAGCCCACGTA

*P. auriga* GCCTTTCTGGTAAAAATGCCCCTATATGGCGTCCATCTATGACTTCCTAAAGCCCACGTA

*C. lucidus* GCATTCCTAGTCAAAATACCACTCTACGGCGTTCACCTTTGACTCCCTAAAGCTCACGTC

*S. sihama* GCATTTCTCGTGAAAATACCCCTTTATGGCGTCCACCTCTGACTACCTAAAGCCCACGTT

*C. loricula* GCTTTCCTAGTTAAGATGCCACTCTATGGGGTTCACCTCTGACTCCCCAAGGCCCATGTC

*A. trutta* GCCTTCCTAGTTAAAATGCCTCTCTACGGGGTTCATCTCTGACTACCTAAAGCACACGTA

*H. gemma* GCTTTTTTAGTCAAAATGCCTCTGTATGGAGTTCATCTCTGACTCCCAAAGGCCCACGTA

*P. flavescens* GCCTTTTTAGTAAAAATACCCCTCTACGGAGTCCACCTCTGACTACCTAAAGCCCACGTA

*P. macrolepida* GCATTTTTAGTAAAAATGCCCTTGTATGGTGTCCACCTGTGACTGCCCAAAGCCCATGTA

*S. canadensis* GCCTTTTTAGTAAAAATGCCTCTCTATGGGGTCCACCTATGGCTCCCTAAAGCACACGTC

*A. rogaa* GAAGCCCCAGTTGCAGGATCTATAGTCCTAGCCGCAGTCCTCCTAAAACTAGGAGGTTAC

*C. argus* GAAGCCCCAGTTGCAGGATCAATGGTCCTAGCTGCAGTTCTCCTAAAACTAGGAGGTTAC

*C. sonnerati* GAAGCCCCCGTTGCAGGATCCATAGTACTTGCAGCAGTCCTATTAAAACTAGGGGGGTAC

*E. fuscoguttatus* GAAGCCCCTGTTGCAGGATCAATAGTACTTGCCGCAGTCCTCTTAAAACTGGGTGGTTAT

*E. coioides* GAAGCTCCTGTTGCAGGCTCAATAGTCCTTGCCGCAGTACTCTTAAAACTAGGCGGTTAC

*E. bruneus* GAAGCCCCCGTTGCAGGGTCAATAGTACTTGCCGCAGTCCTCTTAAAACTAGGAGGTTAC

*E. moara* GAAGCCCCCGTTGCAGGATCAATAGTGCTTGCCGCAGTCCTCTTAAAACTAGGAGGTTAT

*E. lanceolatus*  GAAGCCCCCATTGCAGGATCAATAGTACTTGCTGCAGTCCTCTTAAAACTAGGAGGTTAT

*A. leucogrammicus*  GAGGCCCCCATTGCTGGGTCAATAGTACTTGCTGCAGTCCTCTTAAAACTAGGAGGTTAT

*C. altivelis* GAAGCCCCCGTCGCGGGGTCAATAGTACTTGCCGCAGTCCTCTTAAAACTAGGAGGTTAC

*E. epistictus* GAAGCCCCTATTGCAGGATCGATAGTACTTGCCGCAGTTCTCCTAAAATTAGGAGGTTAT

*E. octofasciatus* GAGGCACCCATTGCAGGATCAATAGTTCTTGCCGCAGTCCTGTTAAAACTAGGAGGTTAC

*E. septemfasciatus* GAAGCACCCGTTGCAGGATCAATAGTTCTTGCCGCAGTTCTGTTAAAACTAGGGGGCTAC

*T. dermopterus* GAAGCCCCTGTTGCAGGATCAATAGTCCTTGCTGCAGTACTGTTAAAACTAGGGGGCTAC

*E. awoara* GAGGCCCCTGTTGCAGGGTCAATAGTACTTGCTGCAGTCCTCCTAAAACTAGGAGGCTAC

*E. akaara* GAAGCCCCTGTTGCAGGATCAATAGTACTTGCTGCAGTCCTCCTTAAACTAGGGGGCTAC

*E. trimaculatus* GAAGCCCCCGTCGCAGGATCTATAGTACTTGCCGCAGTACTCCTAAAACTAGGAGGCTAC

*E. areolatus* GAGGCCCCCGTTGCAGGATCAATAGTCCTTGCCGCAGTTCTCCTGAAACTAGGAGGCTAC

*V. albimarginata* GAAGCCCCTGTGGCCGGATCTATAGTCCTTGCAGCAGTCCTTCTTAAGCTCGGAGGCTAC

*V. louti* GAAGCCCCCGTAGCTGGATCTATGGTTCTTGCAGCTGTACTTCTTAAGCTCGGAGGTTAC

*P. leopardus* GAAGCCCCCGTTGCAGGCTCAATAGTCCTTGCTGCAGTACTTCTAAAACTGGGAGGATAC

*P. areolatus* GAAGCCCCAGTTGCAGGTTCAATAGTCCTTGCTGCAGTACTTCTAAAACTCGGAGGGTAC

*E. radiosum* GAAGCCCCTATTGCGGGTTCTATAATCCTTGCGGCCGTCCTTCTTAAGCTAGGGGGCTAC

*P. sieboldi* GAGGCCCCTGTAGCCGGCTCCATGATCCTTGCCGCTGTCCTCCTAAAACTTGGCGGCTAC

*E. armatus* GAAGCTCCAGTTGCTGGCTCCATAATCCTTGCTGCCGTACTCCTTAAGCTAGGGGGTTAC

*R. oxyrhynchus* GAAGCCCCCGTTGCCGGCTCCATAGTCCTTGCCGCAGTCCTTCTAAAGCTAGGGGGCTAC

*K. cinerascens* GAAGCCCCAGTAGCGGGCTCCATAATTCTTGCCGGCGTCCTCCTAAAATTAGGAGGGTAC

*T. chatareus* GAGGCCCCCATTGCAGGCTCTATAGTTCTTGCCGCCGTCCTCCTAAAATTAGGCGGATAT

*D. berycoides* GAAGCCCCTATCGCAGGCTCCATAGTTCTTGCCGCCGTTCTCCTAAAACTAGGAGGCTAT

*H. typus* GAGGCCCCCGTCGCAGGCTCCATAATCCTTGCCGCCGTCCTACTAAAACTAGGAGGCTAC

*M. argenteus* GAAGCCCCAGTTGCGGGCTCCATGGTTCTTGCTGCTGTCCTCCTAAAACTAGGGGGTTAC

*S. chuatsi* GAAGCACCCGTCGCCGGCTCCATAATCCTTGCTGCCGTGCTCTTAAAACTAGGGGGATAT

*O. fasciatus* GAGGCCCCAGTGGCAGGCTCTATAATTCTTGCCGCAGTCCTCCTGAAACTAGGAGGATAC

*P. trilineatum* GAAGCCCCCGTAGCAGGCTCAATAGTTCTTGCTGCTGTCCTACTAAAACTAGGGGGCTAT

*M. salmoides* GAAGCCCCAATTGCTGGCTCTATAATCCTCGCTGCCGTCCTCCTTAAACTTGGAGGTTAC

*P. tile* GAAGCCCCCGTTGCAGGGTCTATAGTACTTGCCGCTGTCCTCCTAAAATTAGGCGGTTAC

*L. argentimaculatus* GAAGCCCCCGTCGCAGGGTCTATAATCCTTGCTGCTGTTCTACTAAAACTAGGAGGCTAC

*E. struhsakeri* GAAGCCCCAGTTGCAGGCTCCATGATTCTTGCTGCTGTACTTCTAAAACTAGGGGGATAT

*B. albus* GAAGCCCCCGTTGCAGGCTCTATAATCCTTGCCGCAGTCCTCCTAAAATTAGGCGGTTAC

*C. auripes* GAAGCCCCCATTGCCGGCTCTATAATTCTTGCTGCAGTCCTTCTGAAACTTGGCGGCTAC

*C. melampygus* GAGGCTCCAATTGCAGGCTCAATAGTCCTCGCGGCAGTACTTCTAAAACTTGGGGGTTAC

*L. calcarifer* GAAGCCCCCGTTGCAGGTTCAATGATTCTTGCCGCCGTTCTTCTCAAACTAGGAGGTTAT

*S. maena* GAGGCACCTGTCGCAGGCTCAATAATTCTTGCTGCCGTATTACTAAAACTTGGGGGGTAC

*P. auriga* GAAGCACCCGTAGCGGGCTCCATAATCCTTGCTGCTGTCTTACTAAAGCTGGGAGGGTAC

*C. lucidus* GAAGCCCCAATCGCAGGCTCAATAGTCCTTGCTGCCGTCCTCCTAAAACTAGGAGGCTAC

*S. sihama* GAAGCACCCGTTGCTGGCTCAATAATTCTTGCTGCCGTCCTCCTAAAGTTGGGGGGCTAT

*C. loricula* GAAGCCCCAGTTGCAGGTTCTATAGTCCTTGCCGCTGTCCTGCTAAAATTAGGGGGATAC

*A. trutta* GAAGCCCCAATTGCAGGCTCCATAGTACTTGCAGCTGTCCTACTCAAACTAGGAGGTTAC

*H. gemma* GAAGCCCCTGTTGCGGGGTCTATAGTGCTAGCCGCAGTTCTACTTAAGCTTGGAGGCTAC

*P. flavescens* GAGGCTCCTGTTGCAGGCTCAATAATCCTAGCAGCTGTCCTACTAAAACTAGGAGGATAT

*P. macrolepida* GAAGCCCCCGTCGCAGGCTCTATGATTCTTGCAGCCGTTCTTTTAAAACTCGGGGGCTAC

*S. canadensis* GAAGCACCCGTTGCAGGTTCAATATTCTTAGCAGCCGTTTTACTTAAACTAGGAGGTTAC

*A. rogaa* GGGATAATACGAATAATAATTATACTCGACCCCTTAACTACGCAACTAAGCTACCCCTTC

*C. argus* GGGATAATACGCATAATAATTATACTAGACCCCCTAACTACACAACTAAGCTACCCCTTC

*C. sonnerati* GGAATAATACGAATAATGGTCATACTAGACCCCCTCACTAAAGAACTATGTTACCCCTTC

*E. fuscoguttatus* GGAATAATACGAATAATCGTTATACTAGACCCCCTCACTAAAGACCTAAGCTACCCCTTT

*E. coioides* GGAATGATACGAATAGTCGTTATATTAGACCCCCTCACCAAAGACCTAAGCTACCCCTTC

*E. bruneus* GGAATAATACGAATAATTGTTATACTAGACCCCCTTACCAAAGACCTAAGCTACCCTTTC

*E. moara* GGAATAATGCGAATAATTGTTATACTAGACCCCCTCACCAAAGACCTAAGCTACCCTTTC

*E. lanceolatus*  GGAATAATACGAATAATTGTTATACTAGACCCCCTTACCAAAAACCTAAGCTACCCCTTC

*A. leucogrammicus*  GGAATAATACGAATAATAGTTATACTAGACCCCCTCACCAAAAGCCTCAGCTTCCCTTTC

*C. altivelis* GGAATAATACGAATAATTGTTATATTAGACCCCCTTACCAAAGACCTAAGCTACCCCTTC

*E. epistictus* GGAATAATGCGCATACTCGTTATACTAGACCCCCTTACCAAAGAACTAAGCTACCCCTTC

*E. octofasciatus* GGAATAATGCGAATAATTGTTATACTGGACCCCCTTACCAAAGAACTAAGCTACCCCTTT

*E. septemfasciatus* GGAATAATGCGAATAATTATTATACTGGACCCCCTTACCAAAGAACTAAGCTACCCCTTT

*T. dermopterus* GGGATGATGCGAATAATCCCTATACTAGACCCCCTCACCAAAGAACTAGGGTACCCCTTT

*E. awoara* GGAATAATGCGAATAATCGTTATACTAGACCCGCTTACCAAAGAGCTAAGTTACCCCTTC

*E. akaara* GGAATAATGCGAATAATTATTATGCTAGACCCACTTACCAAAGAACTAAGCTACCCTTTT

*E. trimaculatus* GGAATGATGCGGATAATTGTTATGTTAGAACCACTTACTAAAGAATTAAGTTACCCCTTC

*E. areolatus* GGAATAATGCGAATAGTTGTTATGCTTGAACCACTCACCAAAGAATTAAGTTATCCCTTC

*V. albimarginata* GGCATAATACGAATAATCACCATGCTAGACCCATTAACCAAAGAACTATGCTACCCCTTC

*V. louti* GGCATGATACGAATAATGGTTATACTAGACCCAATAACCAAAGAACTATGCTACCCCTTC

*P. leopardus* GGTATAATACGAATAATTTTAGTACTTGACCCCTTGACAAAAGAACTAGGTTACCCATTC

*P. areolatus* GGCATGATACGAATTATCTTAGTACTCGACCCTTTAACAAAAGAACTGAGCTACCCTTTT

*E. radiosum* GGCATAATGCGGATTGTTGTCGTACTTGAACCCCTAACTAAAGACTTAGCTTACCCATTT

*P. sieboldi* GGCATAATGCGCATCCTCATTATTCTAGACCCCTTAACAAAGGAGCTTGGCTACCCCTTT

*E. armatus* GGTATAATACGAATCATGGTTGTTCTCGAACCCCTCACCAAGGAATTAAGTTATCCATTC

*R. oxyrhynchus* GGAATGATGCGCATGATAATTATGCTAGAGCCTTTAACCAAACAACTAAGCTATCCCTTT

*K. cinerascens* GGTATAATACGAATAATAACTATACTAGAGCCACTTACCAAAGAACTAAGCTACCCATTT

*T. chatareus* GGCATAATACGAATAATAATTGCCCTAGAAGCCCTCACCAAAGAACTAAGCTACCCATTT

*D. berycoides* GGCATAATACGAATACTAGTTATACTAGACCCGCTCACTAAGGAACTAAGCTACCCCTTT

*H. typus* GGTATGATACGAATACTAGTTATGCTAGAACCCCTCACTAAAGAACTAAGCTACCCATTT

*M. argenteus* GGCATGATACGAATGTTAGTAGTCCTAGAACCCCTCACTAAGGAACTAAGCTATCCCTTC

*S. chuatsi* GGCATAATACGAATAATGGTCATGTTAGAGCCCCTAACAAAAGAACTAAGCTACCCCTTT

*O. fasciatus* GGTATAATACGAATAATAACCATACTAGAGCCCCTTACCAAAGAGCTAAGCTACCCCTTT

*P. trilineatum* GGCATGATGCGAATGCTAGTAATACTAGAACCCCTCACTAAAGAACTAAGCTACCCCTTC

*M. salmoides* GGCATGATACGAATGATAATTGTTCTAGAGCCCCTTACCAAGGAACTCAGCTACCCTTTT

*P. tile* GGAATAATGCGAATACTAGTAGTCCTTGAGCCCCTAACCAAAGAACTGAGCTACCCCTTC

*L. argentimaculatus* GGAATAATACGAATACTAGTAATGCTAGAGCCCCTAACCAAAGAATTAAGCTACCCCTTC

*E. struhsakeri* GGAATGATACGTATACTTGTTATGCTAGAACCACTCACCAAAGAATTAAGTTACCCATTC

*B. albus* GGTATAATTCGAGTACTCATGATACTAGAGCCCGTGGCTAAACAACTATGCTACCCCTTC

*C. auripes* GGAATAATACGTATACTCCCAATACTAGAACCCCTGACTAAAGAACTAAGCTATCCCTTT

*C. melampygus* GGTATAATACGAATAATAATCATACTAGAGCCCTTAACCAAAGAACTTAGCTATCCATTC

*L. calcarifer* GGGATAATGCGTATAATAGCCATCTTAGACCCCCTCACTAAAGAACTAAGTTACCCCTTC

*S. maena* GGAATAATCCGTATTCTAACAATTCTTGAACCCTTAACTAAGGAACTAAGCTACCCCTTT

*P. auriga* GGTATAATACGCATGCTTACAATCTTCGAATCCTCAACTGAAAAAATAAGCTACCCATTT

*C. lucidus* GGCATGATACGAATTCTCCCCATACTAGAACCTCTAACCAAAGAGTTAAGCTACCCCTTC

*S. sihama* GGAATAATGCGAATGTTGGTAATCCTAGAGCCACTCACCAAGGAGCTAAGCTATCCTTTT

*C. loricula* GGCATGATGCGCATACTAACTGTCCTCGAACCCCTTACCAAGGAACTAAGCTACCCATTT

*A. trutta* GGGATGATGCGAATAATAACGATACTAGACCCCCTAACTAAACAACTAAGTTACCCCTTC

*H. gemma* GGCCTTATTCGAATAACCATTATTCTGGACCCTTTAACCAAAGAACTCAGCTATCCTTTC

*P. flavescens* GGTATAATACGCATAGTCATCATACTTGACCCCCTTACCAAGGACCTGAGTTTCCCGTTT

*P. macrolepida* GGGATGCTACGTATCGTAGTAATACTGGAACCCCTCACCAAAGAGCTAAGCTACCCCTTC

*S. canadensis* GGACTAATACGTATCGTCGTAATACTTGACCCACTAACCAAAGACCTAAGCTACCCATTC

*A. rogaa* CTTATCTTCGCACTTTGAGGAGTAATCATAACAGGTTCAATCTGCCTCCGTCAAACTGAT

*C. argus* CTCATCTTCGCACTCTGAGGGGTGGTCATAACAGGGTCAATTTGTCTCCGCCAAACTGAT

*C. sonnerati* TTAATCTTCGCACTATGAGGAGTAATCATAACAGGCTCTATTTGTCTCCGCCAAACCGAC

*E. fuscoguttatus* CTTATCTTTGCATTATGGGGGGTTATTATAACGGGCTCAATCTGTCTCCGTCAAACCGAC

*E. coioides* CTCATCTTTGCATTATGAGGAGTAATCATAACAGGCTCGATCTGTCTTCGCCAGACCGAC

*E. bruneus* CTTATCTTCGCATTATGGGGGGTTATTATAACAGGCTCGATCTGTCTTCGCCAGACTGAC

*E. moara* CTCATCTTCGCATTATGAGGGGTTATCATAACAGGCTCAATCTGCCTTCGCCAGACTGAC

*E. lanceolatus*  CTCATCTTTGCATTATGAGGGGTGATCATAACAAGCTCAATCTGTCTCCGTCAAACTGAC

*A. leucogrammicus*  CTTGCCTTTGCGTTATGGGGGGTTGTTATAACGGGCTCCATTTGCCTCCGCCAAACTGAC

*C. altivelis* CTCATCTTCGCACTATGAGGGGTCATTATAACAGGCTCAATCTGTCTCCGTCAAACTGAC

*E. epistictus* CTTATTTTCGCACTATGAGGCGTTATCATAACAGGCTCAATCTGTCTCCGCCAAACTGAC

*E. octofasciatus* CTTATCTTCGCATTATGAGGTGTTATCATAACAGGCTCAATCTGTCTTCGCCAAACTGAC

*E. septemfasciatus* CTTATCTTCGCATTATGGGGCGTTATCATAACAGGCTCAATCTGCCTTCGCCAGACTGAC

*T. dermopterus* CTTATCTTCGCATTATGAGGTGTAATTATAACAGGCTCAATCTGCCTTCGCCAAACGGAC

*E. awoara* CTTATCTTCGCATTATGAGGGGTTATCATAACAGGCTCTATCTGCCTCCGCCAAACTGAC

*E. akaara* CTTGTCTTCGCGTTATGGGGGGTGATCATGACAGGCTCAATCTGTCTCCGCCAAACTGAC

*E. trimaculatus* CTTATCTTTGCATTATGAGGTATTATCATAACAAGCTCAATTTGCCTCCGCCAAACAGAC

*E. areolatus* CTTATTTTTGCACTATGAGGAGTCATTATAACAAGCTCGATTTGTCTCCGCCAAACTGAT

*V. albimarginata* ATTATTCTTGCCCTGTGGGGTGTTGTAATAACAGGCTCAATTTGCCTACGACAAACTGAC

*V. louti* ATTGTCTTTGCCCTATGAGGTGTTGTAATAACAGGCTCAATTTGCCTACGGCAAACCGAC

*P. leopardus* ATTATCCTTGCACTCTGAGGGGTAATTATAACCGGCTCCATCTGCCTCCGCCAAACTGAC

*P. areolatus* ATCATCCTCGCACTTTGAGGGGTAATCATGACCGGCTCCATCTGCCTCCGCCAAACCGAC

*E. radiosum* ATTATCTTTGCACTATGAGGGGTAGTTATAACAGGGTCTATTTGCCTACGTCAAACGGAT

*P. sieboldi* ATCGTCCTAGCCCTGTGAGGGGTTATTATGACGGGCTCTATCTGCCTACGTCAAACGGAC

*E. armatus* ATTATTTTTGCCCTCTGGGGAGTAATTATAACAGGCTCAATCTGTCTTCGACAAACGGAT

*R. oxyrhynchus* ATCATCTTTGCCCTCTGAGGAATCGTAATAACAGGCTCAATTTGCCTCCGCCAAACAGAC

*K. cinerascens* ATTATCTTCGGCCTCTGAGGCGTAATCATAACAGGCTCCATCTGCCTACGCCAAACAGAC

*T. chatareus* ATCATTCTAGCACTCTGAGGAGTTATCATAACAGGCTCAATTTGCTTACGACAAACAGAC

*D. berycoides* ATTATCTTTGCACTCTGAGGAGTAATTATAACAGGCTCAATTTGCTTACGCCAAACAGAC

*H. typus* ATCATCTTTGCACTTTGAGGTGTCGTCATAACAGGCTCGATCTGCCTTCGCCAAACAGAC

*M. argenteus* ATCATCTTCGCATTATGAGGCGTAATCATAACAGGCTCAATTTGCTTACGTCAAACAGAC

*S. chuatsi* CTTATCTTCGCCCTTTGAGGGGTAATTATAACCGGCTCAATTTGCTTACGCCAAACAGAC

*O. fasciatus* ATCGTTTTTGCCCTCTGAGGCGTAATCATAACAGGCTCAATCTGCCTACGCCAAACAGAC

*P. trilineatum* ATCATCTTCGCCCTCTGAGGAGTAATCATAACAGGCTCAATTTGCTTACGGCAAACAGAC

*M. salmoides* ATTATCTTTGCACTCTGAGGAGTAATCATAACAGGCTCAATCTGTTTACGCCAAACGGAC

*P. tile* CTCATCCTCGCACTCTGAGGAGTAATCATGACTGGCTCAATCTGCCTACGACAAACGGAC

*L. argentimaculatus* ATTATCCTAGCGCTATGAGGAGTAATTATGACCGGCTCAATCTGCTTACGCCAAACAGAT

*E. struhsakeri* ATCATCTTCGCCCTCTGAGGGGTCATTATAACAGGCTCCATCTGCCTACGTCAAACAGAC

*B. albus* ATCATTCTCGCCCTCTGAGGTGTGATTATAACAGGCTCTACATGCCTACGCCAAACAGAC

*C. auripes* ATTCTCCTCGCACTTTGAGGGGTTGTAATGACAGGATCAATCTGCATGCGCCAAACAGAC

*C. melampygus* ATTATCTTCGCCCTCTGAGGAGTAATTATAACAGGATCTATTTGCCTGCGCCAAACAGAC

*L. calcarifer* ATCATTTTTGCCCTATGAGGCCTCGTCATAATAGGTTCAACCTGCCTTCGTCAAGCTGAC

*S. maena* ATTATTTTTGCACTCTGGGGAGTAATTATAACTGGCTCAATTTGTTTACGACAAACTGAC

*P. auriga* ATTATCTTTGCCCTCTGAGGTGTAATTATAACCGGCTCAATCTGCTTGCGCCAAACCGAC

*C. lucidus* ATCATTTTTGCACTTTGAGGGGTAATCATAACTGGCTCAATTTGTTTACGCCAAACAGAC

*S. sihama* ATTGTACTCGCGCTCTGAGGAGTCATCATAACTGGCTCTATTTGTTTACGCCAAACAGAC

*C. loricula* ATTATTCTCGCGCTTTGAGGTGTAATTATGACAGGATCAATTTGCCTCCGCCAAACTGAC

*A. trutta* ATCGCCTTTGCCCTATGAGGCGTGATTATGACAGGGTCAATTTGCCTCCGGCAAACAGAC

*H. gemma* ATTGTAATTGCCTTATGGGGTGTAATTATGACAAGCTCAATCTGTCTCCGCCAGACAGAC

*P. flavescens* ATTATCTTTGCTCTCTGAGGCGTAATTATAACCGGGTCAATCTGCTTACGTCAAACAGAC

*P. macrolepida* ATTATCTTCGCTCTATGAGGAGTAATCATAACGGGCTCAATTTGCCTACGTCAAACGGAC

*S. canadensis* ATTATTTTTGCCCTCTGGGGGGTAATTATAACCGGGTCTATTTGCCTACGACAAACAGAC

*A. rogaa* CTAAAATCCCTAATTGCCTACTCTTCGGTAAGTCACATAGGCCTAGTTGTAGGAGGAATT

*C. argus* CTAAAATCTTTAATTGCTTACTCTTCAGTAAGCCACATAGGCCTTGTTGTAGGAGGTATT

*C. sonnerati* CTCAAATCTCTAATTGCTTACTCCTCAGTAAGCCACATAGGCCTAGTAGTAGGAGGCATC

*E. fuscoguttatus* CTAAAATCCCTAATTGCTTACTCTTCAGTAAGCCACATAGGCTTAGTAGTAGGGGGAATC

*E. coioides* CTGAAATCTCTAATTGCTTACTCCTCAGTAAGCCACATAGGCCTTGTAGTAGGAGGAATT

*E. bruneus* CTAAAATCTCTAATTGCTTACTCCTCAGTAAGCCACATGGGCTTAGTAGTAGGAGGGATC

*E. moara* CTAAAATCTCTAATTGCTTACTCCTCAGTAAGCCACATAGGCTTAGTGGTAGGAGGAATC

*E. lanceolatus*  CTAAAATCCCTAATTGCTTACTCCTCAGTAAGCCACATAGGTTTAGTAGTAGGAGGGATC

*A. leucogrammicus*  CTAAAGTCCCTAATTGCTTACTCTTCAGTAAGCCACATGGGGCTTGTAGTAGGAGGAATC

*C. altivelis* TTAAAATCTCTGATTGCATACTCCTCAGTAAGCCACATGGGCCTAGTGGTAGGAGGAATT

*E. epistictus* CTAAAATCCCTAATCGCATACTCATCAGTAAGCCACATAGGTCTAGTAGTAGGAGGAATC

*E. octofasciatus* CTTAAGTCTCTAATTGCATACTCTTCAGTAAGCCACATGGGTTTAGTAGTAGGGGGAATC

*E. septemfasciatus* CTTAAGTCTCTAATTGCATACTCCTCAGTAAGCCACATGGGTTTAGTAGTAGGAGGTATC

*T. dermopterus* CTCAAGTCTCTAATTGCATACTCTTCAGTAAGCCACATAGGTTTAGTAGTAGGGGGCATT

*E. awoara* CTAAAATCCCTAATTGCTTACTCCTCAGTAAGCCACATAGGTTTAGTAGTTGGGGGGATT

*E. akaara* CTAAAATCCCTAATTGCATATTCCTCAGTAAGTCACATAGGCTTAGTGGTTGGGGGCATC

*E. trimaculatus* CTAAAATCCCTAATCGCTTACTCCTCAGTAAGCCACATGGGCTTAGTAGTAGGAGGAATC

*E. areolatus* CTAAAATCTCTAATCGCATACTCCTCAGTAAGCCATATAGGCCTAGTAGTAGGAGGTATC

*V. albimarginata* CTAAAATCTCTAATTGCTTACTCTTCAGTTAGCCACATAGGCTTAGTTGTCGGCGGGATC

*V. louti* CTGAAGTCTCTAATTGCTTACTCTTCAGTTAGTCACATGGGCTTAGTTGTTGGTGGGATC

*P. leopardus* CTAAAATCCCTAATTGCCTACTCTTCAGTTAGCCATATGGGCCTGGTAATTGGAGGAATC

*P. areolatus* CTAAAATCCCTAATTGCCTACTCCTCAGTTAGCCACATAGGGCTCGTAATCGCGGGAATT

*E. radiosum* CTGAAATCCCTTATTGCCTACTCCTCAGTAAGCCACATGGGTTTAGTCGTCGGGGGTATT

*P. sieboldi* CTAAAGTCCCTTATCGCCTACTCCTCTGTTGGTCACATGGGCCTTGTAGTAGGGGGCATT

*E. armatus* CTTAAGTCCCTAATCGCTTACTCATCAGTAAGTCATATGGGACTTGTAGTTGGGGGTATT

*R. oxyrhynchus* CTAAAATCCCTTATCGCTTACTCATCTGTAAGTCACATAGGCCTCGTCGTAGGAGGAATT

*K. cinerascens* CTTAAATCCCTAATCGCTTACTCATCAGTAAGCCACATGGGACTTGTTGTAGGAGGTATC

*T. chatareus* CTAAAATCCCTCATCGCCTACTCATCCGTAAGCCATATAGGACTCGTCGCAGGTGGAATC

*D. berycoides* CTGAAATCCCTCATCGCTTACTCATCAGTCAGTCATATAGGACTGGTTGTAGCAGGCATC

*H. typus* CTAAAGTCCCTCATTGCCTACTCATCAGTAAGTCATATAGGACTAGTCGTAGGCGGAATT

*M. argenteus* CTAAAATCCCTCATTGCATATTCATCAGTGAGCCATATGGGCCTTGTGGTCGGAGGAATC

*S. chuatsi* TTAAAATCTCTCATTGCTTATTCATCAGTTAGCCACATAGGCCTAGTTGTAGGGGGCATC

*O. fasciatus* CTAAAATCCCTAATTGCATACTCCTCAGTAAGCCACATGGGTCTCGTAGTAGGAGGAATC

*P. trilineatum* CTGAAATCCCTAATTGCCTACTCCTCCGTTAGCCACATAGGCCTGGTAGTAGGAGGCATT

*M. salmoides* CTAAAGTCCCTAATCGCCTACTCCTCCGTCGGTCACATGGGCCTAGTTGTTGGGGGCATT

*P. tile* CTAAAATCCCTTATTGCCTATTCCTCAGTTAGCCATATGGGACTAGTAGTCGGAGGAATC

*L. argentimaculatus* CTAAAATCCCTCATCGCCTACTCCTCCGTCAGCCACATGGGCCTCGTAGTAGGTGGCATC

*E. struhsakeri* CTAAAGTCTCTCATTGCTTACTCCTCAGTAAGCCACATAGGCCTAGTAGTAGGTGGCATC

*B. albus* TTAAAATCCCTAATCGCTTACTCATCAGTAAGTCACATAGGATTAGTGATTGCTGGCATC

*C. auripes* CTAAAGTCTCTTATCGCCTACTCATCTGTCAGCCACATAGGGCTGGTGATTGCAGCTATT

*C. melampygus* CTAAAATCACTCATTGCCTATTCATCAGTAAGCCACATGGGCCTAGTAGCAGGAGGTATT

*L. calcarifer* CTAAAGTCCATAATTGCCTACTCATCAGTAGCTCACATAGGCCTCGTCGTAGCGGGGATC

*S. maena* CTCAAATCCCTCATTGCCTATTCCTCCGTTAGTCATATGGGCCTGGTAGTGGGAGGAATC

*P. auriga* CTAAAATCTTTAATTGCTTACTCATCTGTAAGCCACATAGGACTAGTAATTGGGGGTATT

*C. lucidus* CTAAAATCCCTCATCGCTTATTCTTCCGTCGGCCACATGGGCCTGGTGGTAGGCGGAATC

*S. sihama* CTTAAATCCCTGATTGCTTACTCATCAGTAAGCCACATGGGACTCGTCACTGGAGGAATT

*C. loricula* CTCAAGTCCCTCATCGCCTACTCTTCAGTAAGCCATATGGGCCTTGTAGTCGGAGGGATC

*A. trutta* TTAAAAGCCCTGATTGCTTATTCCTCAGTGAGTCATATGGGTCTCGTTGCAGGAGGAATT

*H. gemma* CTCAAATCACTAATTGCCTACTCATCTGTAAGCCACATAGGCTTAGTAGTCGCAGGGATT

*P. flavescens* CTAAAATCGCTTATCGCTTACTCCTCCGTAAGTCATATAGGCCTCGTTGTAGGAGGAATT

*P. macrolepida* CTGAAATCCCTTATCGCCTACTCCTCAGTCAGCCACATGGGACTGGTAGTGGGAGGAATT

*S. canadensis* CTGAAATCACTCATCGCTTATTCTTCCGTAAGCCATATAGGCCTAGTCGTAGGGGGAATT

*A. rogaa* TTAATTCAAACACCATGAGGGTTTACAGGCGCACTAATCCTCATAATCGCTCACGGACTA

*C. argus* TTAATCCAGACACCATGAGGGGCTTCAGGTGCGCTAATCCTCATAATTGCCCACGGGTTA

*C. sonnerati* CTAATTCAGACACCCTGAGGATTTGCAGGAGCACTAATCCTTATAATTGCCCACGGATTA

*E. fuscoguttatus* CTAATTCAAACCCCCTGAGGATTTACAGGAGCCTTAATTCTTATAATCGCTCACGGACTA

*E. coioides* CTAATCCAAACCCCTTGAGGATTTACAGGAGCCCTAATCCTCATAATTGCCCACGGACTA

*E. bruneus* TTAATCCAAACCCCTTGAGGGTTTACAGGAGCCTTAATCCTTATAATCGCCCACGGATTG

*E. moara* TTAATCCAAACCCCCTGAGGATTCACAGGAGCCTTAATCCTTATAATCGCCCACGGGTTA

*E. lanceolatus*  CTGATCCAAACACCCTGAGGATTCACAGGAGCCCTAATCCTTATAATTGCCCACGGATTA

*A. leucogrammicus*  TTAATTCAAACCCCCTGAGGATTTTCAGGGGCCTTAATCCTTATAATCGCCCACGGACTA

*C. altivelis* CTAATTCAAACCCCCTGAGGATTTACAGGGGCCTTAATCCTTATAATTGCCCACGGACTA

*E. epistictus* TTAATCCAAACCCCCTGAGGCTTCACAGGTGCCCTAATTCTTATAATCGCCCACGGGTTA

*E. octofasciatus* CTAATTCAAACCCCTTGAGGGTTTACAGGAGCCCTAATTCTTATAATCGCCCACGGATTA

*E. septemfasciatus* CTAATTCAAACCCCTTGAGGGTTTACAGGAGCCCTAATTCTTATAATCGCCCACGGATTA

*T. dermopterus* CTAATTCAAACCCCCTGAGGGTTCACAGGGGCCCTTATTCTTATAATTGCCCACGGACTG

*E. awoara* CTCATTCAAACTCCCTGAGGGTTCACAGGGGCCTTAATCCTTATAATTGCTCACGGGTTA

*E. akaara* CTTATTCAAACCCCCTGAGGGTTTACAGGAGCCCTAATCCTCATAATTGCCCACGGGTTG

*E. trimaculatus* CTTATCCAAACCCCCTGGGGATTTACAGGAGCTCTAATCCTTATAATTGCCCACGGACTA

*E. areolatus* CTCATCCAAACCCCTTGAGGATTTACAGGAGCCTTAATCCTCATAATCGCCCACGGACTA

*V. albimarginata* TTAATCCAAACACCCTGAGGATTTGCAGGCGCACTCATCCTTATAATTGCACATGGACTT

*V. louti* TTAATCCAAACGCCCTGAGGATTTGCAGGCGCACTTATTCTTATAATTGCACACGGACTT

*P. leopardus* CTCATCCAAACGCCCTGAGGCTTCACCGGAGCACTAATTCTTATGATCGCCCACGGACTT

*P. areolatus* CTTATTCAAACGCCCTGAGGTTTTACTGGCGCACTAATTCTCATGATTGCCCACGGACTA

*E. radiosum* CTTATTCAAACCCCATGAGGCTTCTCGGGAGCTCTCATCTTAATAATTGCACACGGCCTA

*P. sieboldi* CTAATTCAAACCCCATGGGGCTTCGCGGGAGCTTTGATCCTTATGATTGCTCACGGCTTA

*E. armatus* TTAATTCAAACCCCCTGAGGCTTCACAGGCGCCCTAATCCTTATAATTGCCCACGGCCTC

*R. oxyrhynchus* CTAATCCAGACACCATGAGGATTCACAGGAGCACTTATTCTTATAATTGCTCATGGCTTA

*K. cinerascens* CTCATTCAAACCCCATGAGGCTTCACCGGCGCACTAATCCTTATAATCGCACACGGCCTA

*T. chatareus* CTCATTCAAACACCCTGAGGATTTACCGGAGCACTTATTCTAATAATCGCTCACGGACTA

*D. berycoides* CTAATCCAAACGCCCTGAGGCTTTACTGGCGCAATTATTCTTATAATTGCACACGGCTTA

*H. typus* CTCATCCAAACGCCCTGGGGCTTTACTGGTGCACTTATTCTTATAATCGCACACGGACTA

*M. argenteus* CTCATTCAAACCCCCTGAGGCTTCACCGGGGCTCTTATCCTAATAATTGCTCACGGCCTA

*S. chuatsi* CTCATCCAAACCCCATGAGGATTTACAGGAGCCCTCATCCTTATAATTGCACATGGCCTA

*O. fasciatus* CTTATTCAAACACCATGAGGATTCACTGGAGCCCTAATCCTTATAATCGCACATGGCCTA

*P. trilineatum* CTCATCCAAACCCCATGGGGATTCACAGGCGCCCTAATTCTTATGATTGCACATGGCCTA

*M. salmoides* CTCATTCAGACCCCCTGAGGCTTTACAGGGGCCCTTATTCTTATAATTGCACATGGACTT

*P. tile* CTCATCCAAACACCCTGAGGCTTCACCGGTGCCCTTATCCTAATAATTGCCCACGGCCTG

*L. argentimaculatus* CTAATTCAAACCCCCTGAGGTTTCACCGGGGCCCTTATCCTAATAATTGCCCATGGTCTA

*E. struhsakeri* CTCATCCAAACGCCCTGAGGCTTTACTGGGGCTCTTATTCTTATGATCGCACACGGCTTA

*B. albus* TTAACACTGTCCCCCTGAGGTATCACCGGCTCCCTCATCCTAATAATCTCCCACGGCCTA

*C. auripes* CTAATCCAATCACCCTGAGCACTTTCAGGTGCAATAATCCTCATAATTGCACACGGCTTA

*C. melampygus* CTTATTCAAACACCCTGAGGCTTTACAGGAGCCCTTATTCTAATAATTGCCCACGGCCTC

*L. calcarifer* TTAATCCAAACCCAATGAAGCTTCACCGGTGCACTCATCCTCATGATCGCACACGGTCTG

*S. maena* CTCATCCAAACCCCCTGGGGCCTTTCCGGAGCACTAATTCTTATGATTGCTCACGGCTTA

*P. auriga* CTAATTCAAACCCCTTGGTCCTTTACTGGGGCTCTAACCCTGATAATCGCTCATGGATTA

*C. lucidus* CTTATCCAAACCCCCTGAGGACTCACAGGGGCCCTTATTCTCATGATTGCCCACGGACTC

*S. sihama* CTTATTCAAACTCCTTGGGGGTTCACGGGGGCACTGATTCTTATGATCTCCCACGGACTT

*C. loricula* CTAATCCAAACAGCCTGAGGCTTCACAGGAGCGTTAATTCTCATAATTGCCCACGGCTTA

*A. trutta* TTGATTCAAACACCATGAGGGTTTTCAGGCGCCCTTATCCTCATAATTGCACATGGATTA

*H. gemma* CTTATTCAAACACCCTGAGGACTAGCTGGTGCAATGATCTTAATAATTTCACACGGGCTT

*P. flavescens* CTAATTCAAACGCCCTGAGGCTTTTCTGGGGCCCTTATTCTCATAATTGCCCACGGCCTA

*P. macrolepida* CTTATTCAAACCCCCTGAGGCTTCTCGGGGGCTCTTATTTTAATAATCGCCCACGGATTA

*S. canadensis* CTTATCCAAACACCCTGGGGCTTCTCGGGAGCACTTATTCTTATAATTGCTCACGGTCTA

*A. rogaa* ACATCCTCAGCCTTATTCTGCTTAGCCAATACGAACTATGAACGAACACACAGCCGAACC

*C. argus* ACATCTTCCGCCTTATTCTGCTTAGCCAATACAAACTATGAGCGAACACATAGCCGAACT

*C. sonnerati* ACATCTTCCGCCCTATTCTGCTTAGCTAATACAAACTATGAACGAACACACAGCCGAACC

*E. fuscoguttatus* ACCTCTTCTGCCTTATTCTGTTTGGCCAACACAAACTATGAACGAACACATAGTCGAACT

*E. coioides* ACCTCCTCCGCCCTATTCTGTTTAGCCAACACAAACTACGAACGAACACACAGTCGAACC

*E. bruneus* ACTTCTTCTGCCCTATTCTGTTTAGCCAATACAAACTACGAACGAACGCATAGCCGAACC

*E. moara* ACTTCTTCTGCTCTATTCTGTTTAGCCAATACAAACTACGAACGAACACACAGCCGAACC

*E. lanceolatus*  ACTTCTTCCGCCTTATTCTGTCTAGCTAATACAAATTATGAACGAACACACAGCCGAACC

*A. leucogrammicus*  ACCTCCTCCGCCCTATTCTGCCTAGCCAATACAAATTACGAACGAACACACAGCCGGACT

*C. altivelis* ACCTCTTCCGCCCTATTCTGCCTAGCCAACACAAACTACGAACGAACACACAGCCGGACT

*E. epistictus* ACATCCTCCGCCCTATTCTGCCTAGCCAATACAAACTATGAACGAACACATAGCCGAACC

*E. octofasciatus* ACATCCTCCGCCCTATTCTGCTTAGCTAACACAAACTACGAACGCACACACAGCCGAACT

*E. septemfasciatus* ACATCCTCCGCCCTATTCTGCTTAGCTAACACAAACTACGAACGCACACACAGCCGAACT

*T. dermopterus* ACATCCTCCGCCCTATTCTGCTTAGCCAATACAAACTACGAACGTACACACAGCCGAACC

*E. awoara* ACATCTTCTGCCCTATTCTGTTTAGCCAATACAAACTACGAACGAACACACAGCCGAACC

*E. akaara* ACATCTTCTGCTTTATTTTGTTTAGCCAACACAAACTACGAACGAACACACAGCCGAACC

*E. trimaculatus* ACATCATCTGCTTTATTCTGCCTAGCCAACACAAACTATGAACGAACACACAGCCGGACT

*E. areolatus* ACATCTTCCGCCCTTTTCTGCCTAGCCAACACAAACTACGAACGAACACACAGCCGAACC

*V. albimarginata* ACATCTTCCGCCTTATTCTGCCTAGCTAATACCAATTACGAACGTACACACAGTCGAACC

*V. louti* ACATCTTCCGCCTTATTCTGTCTAGCCAATACTAACTACGAACGTACACATAGTCGAACC

*P. leopardus* ACATCTTCAGCCCTTTTCTGTTTAGCCAACACCAACTATGAGCGCACACATTCTCGAACC

*P. areolatus* ACATCTTCGGCCCTTTTCTGCTTAGCTAACACCAACTACGAGCGCACACACTCTCGAACC

*E. radiosum* ACATCTTCTGCTCTTTTTTGTCTGGCTAATACAAATTACGAGCGCACCCACAGCCGAACA

*P. sieboldi* ACCTCCTCTGCCCTCTTTTGCCTGGCCAACACCAACTACGAGCGCACTCATACCCGAACT

*E. armatus* ACATCTTCCGCCTTATTCTGTCTTGCAAACACTAATTATGAACGCACACATAGTCGAACC

*R. oxyrhynchus* ACCTCCTCCGCCCTCTTCTGCCTTGCAAATACTAACTACGAACGAACTCATAGTCGAACT

*K. cinerascens* ACATCCTCCGCCCTATTCTGCTTAGCAAACACCAACTACGAACGAACACATAGTCGAACC

*T. chatareus* ACATCCTCCGCTCTGTTCTGCCTCGCAAATACCAACTACGAACGCACCCACAGCCGGACT

*D. berycoides* ACTTCCTCCGCCCTATTCTGTTTAGCTAATACTAACTATGAGCGCACACATAACCGAACT

*H. typus* ACCTCCTCCGCCCTATTCTGCCTAGCAAATACCAATTATGAACGCACTCATAGCCGAACC

*M. argenteus* ACATCCTCCGCCCTATTCTGTCTGGCAAACACAAATTACGAACGTACACATAGCCGAACA

*S. chuatsi* ACATCCTCAGCCCTATTCTGCCTAGCAAATACCAACTACGAACGAACCCACAGCCGGACC

*O. fasciatus* ACCTCCTCCGCCCTATTCTGCCTAGCAAACACTAATTATGAACGAACACATAGCCGAACA

*P. trilineatum* ACATCCTCAGCCCTATTCTGCCTAGCTAACACTAATTATGAACGCACCCACAGTCGAACA

*M. salmoides* ACGTCCTCTGCCCTATTCTGCCTTGCAAACACAAACTACGAGCGTACACACAGCCGAACC

*P. tile* ACATCCTCTGCCCTCTTCTGCTTAGCTAACACCAACTATGAACGAACACACAGCCGAACA

*L. argentimaculatus* ACATCCTCAGCCCTCTTCTGTCTAGCAAACACTAACTATGAGCGCACACACAGCCGAACC

*E. struhsakeri* ACATCCTCCGCCCTATTCTGCCTAGCAAATACTAACTATGAACGCACACACAGCCGAACC

*B. albus* ACATCATCCGCCCTATTCTGCCTCGCAAACACCAACTATGAACGCACACACAGCCGAACT

*C. auripes* ACATCTTCCGCCATATTCTGCTTAGCAAATACCGCCTATGAACGAACACACAGCCGAACA

*C. melampygus* ACATCCTCTGCCCTCTTTTGCTTAGCAAATACTAATTACGAACGAACACATAGCCGAACA

*L. calcarifer* ACAGCCTCCGCCCTATTCTGCCTAGCCAATACCAACTACGAACGCACTCACAGTTGAGTA

*S. maena* ACATCTTCTGCCCTCTTCTGTTTAGCTAACACCAACTATGAACGTACCCACAGTCGAACA

*P. auriga* ACCTCCTCCGCACTCTTTTGTCTGGCTAACACCAATTATGAACGTACCCATAGCCGAACA

*C. lucidus* ACTTCCTCCGCCCTATTCTGCCTCGCCAACACAAACTACGAACGAACCCACAGCCGAACA

*S. sihama* ACGTCATCAGCCCTTTTCTGCTTAGCCAACACAAATTACGAGCGAACTCACAGTCGGACC

*C. loricula* ACTTCGTCCGCTCTCTTCTGCCTCGCAAATACTAACTACGAACGCACCCACAGCCGAACA

*A. trutta* ACATCCTCGGCCCTTTTCTGCTTAGCTAACACTAATTACGAGCGGACACACAGTCGAACC

*H. gemma* ACCTCTTCCGCCCTCTTCTGCCTTGCTAACACCAACTATGAACGAACCCACAGCCGGACT

*P. flavescens* ACATCCTCGGCTCTCTTCTGCCTAGCAAATACAAACTACGAACGAACTCACAGTCGAACA

*P. macrolepida* ACATCTTCTGCCCTCTTTTGCTTGGCAAACACAAACTACGAACGCACCCACAGTCGAACG

*S. canadensis* ACATCCTCGGCCCTCTTCTGCCTAGCAAATACAAACTACGAACGTACACACAGTCGAACT

*A. rogaa* ATACTCCTAGCACGCGGCCTACAAGTCGTTTTGCCTTTAATAACAACCTGATGATTCATT

*C. argus* TTGCTCTTAGCACGAGGTCTACAAATCTCCCTACCTTTAATAGCTACCTGGTGATTCATC

*C. sonnerati* ATGCTCTTAGCACGAGGCCTACAAGTTGTATTACCTCTTATAACAACATGATGATTTATT

*E. fuscoguttatus* ATACTCCTAGCACGCGGCCTACAAATCGTCCTTCCTCTTATAACAGCTTGATGATTCATT

*E. coioides* ATGCTTCTAGCCCGCGGCCTGCAAATCATTCTTCCCCTTATAACAGCTTGATGATTTATT

*E. bruneus* ATACTTCTAGCACGCGGCCTACAAATCGTGCTTCCCCTAATAACAGCCTGATGATTCATT

*E. moara* ATACTTCTAGCGCGCGGCCTACAAATCGTACTTCCCCTGATAACAGCCTGATGATTCATT

*E. lanceolatus*  ATACTGCTAGCACGCGGCTTACAAATCATTCTCCCCCTAATAACAACCTGATGATTCATT

*A. leucogrammicus*  ATAATCTTAGTACGCGGCCTACAAATAATTCTTCCTCTTATGACAGCCTGATGATTTATC

*C. altivelis* ATACTCCTAGCACGTGGCCTACAAATCGCCCTTCCCCTAATAACAGCCTGATGGTTTATT

*E. epistictus* ATACTCCTGGCACGAGGCCTACAAATTGTTTTTCCCCTAATAACAACCTGATGATTCATC

*E. octofasciatus* ATGCTTCTAGCACGTGGCCTACAAATTATTTTGCCCTTAATAACAGCCTGATGATTCGTT

*E. septemfasciatus* ATACTCCTAGCACGTGGCCTACAAATTATCTTGCCCCTAATAACAGCCTGATGATTTATT

*T. dermopterus* ATACTCTTAGCACGCGGCCTGCAGATTATCCTACCCCTAATGACAACCTGATGATTCATT

*E. awoara* ATGCTTCTAGCACGTGGGCTACAAATTATTCTTCCCTTGATAACAGCTTGATGATTCATT

*E. akaara* ATGCTCCTAGCACGTGGTCTACAAATTATTCTTCCCTTAATAACAGCCTGATGGTTTATT

*E. trimaculatus* ATGCTCCTAGCCCGCGGCCTACAAATTATTCTCCCCTTAATGACAACCTGATGATTCATT

*E. areolatus* ATACTATTAGCACGAGGCTTACAAATTATTTTACCTCTAATAACAGCCTGATGATTTATT

*V. albimarginata* ATACTCTTAGCCCGAGGGTTACAAGTGATTCTCCCCCTAATGACAACATGATGATTTATT

*V. louti* ATACTCTTAGCCCGAGGATTACAAGTAATCCTCCCACTAATAGCAACGTGATGATTTATT

*P. leopardus* ATGCTTTTAGCCCGGGGACTTCAAATAGTATTACCTCTAATGACCGCTTGATGATTTATT

*P. areolatus* ATGCTTTTAGCCCGAGGGCTTCAAATAGTCCTACCCCTAATAGCTGCTTGATGGTTCACT

*E. radiosum* ATACTCTTAGCCCGAGGCCTACAAATAGTACTACCCCTAATGGCAGCCTGATGATTTATT

*P. sieboldi* ATACTCTTGGCCCGCGGCCTTCAAATGGCCCTGCCACTGATGACAACCTGGTGGTTTATT

*E. armatus* ATGGTCTTAGCACGAGGTTTACAAATGGCCCTCCCACTTATAACATCCTGATGATTCATT

*R. oxyrhynchus* ATAGTATTGGCCCGAGGGCTTCAAATCGCTCTCCCCCTAATAACCACATGATGATTTATT

*K. cinerascens* ATAGTCCTAGCTCGAGGATTACAAATAGCCCTTCCCCTAATAACATCATGATGATTCATC

*T. chatareus* ATAATCTTAGCACGAGGCCTTCAAATAGTCCTCCCCTTAGCAGCCACATGATGATTCATC

*D. berycoides* TTAATTCTAACTCGAGGCCTACAAATGGCCCTTCCCTTAATAACAACGTGATGATTCATT

*H. typus* ATAATTCTAGCACGAGGCCTCCAAATAGCCCTTCCCCTAATAACAACTTGATGATTCATT

*M. argenteus* ATAGTACTAGCACGAGGACTTCAAATAGCCCTCCCCCTAATAACCACATGATGATTTATT

*S. chuatsi* ATAGTTCTAGCACGAGGACTACAAATGGCCCTTCCCCTAATAACAACATGATGATTTATT

*O. fasciatus* CTAGTCCTAGCCCGAGGCCTACAAATAGCCCTTCCCCTAATGACAACATGATGATTCATT

*P. trilineatum* ATAGTGCTTGCACGAGGACTCCAAATAGCCCTTCCTTTAATAACCACATGATGATTCATC

*M. salmoides* ATGGTCTTAGCCCGAGGCTTACAAATGGCCCTCCCCTTGATGACATCCTGATGATTTATT

*P. tile* ATAGTTCTAGCCCGAGGACTCCAAATGGCCCTCCCCCTTATAACAACCTGATGATTCATC

*L. argentimaculatus* ATGGTATTAGCACGAGGACTCCAAATGGCACTTCCGCTTATAACAACCTGGTGATTCATC

*E. struhsakeri* ATAGTTCTAGCACGAGGCCTACAAATAGCTCTTCCACTAATAACAACATGATGATTCATC

*B. albus* ATACTCCTAGCACGAGGCCTACAGACGGCCCTCCCATTGATAGCGATATGATGGTTCCTC

*C. auripes* ATACTCTTAACACGAGGTATGCAGATAGTTCTCCCATTAATAGTCTCCTGATGGTTTGCC

*C. melampygus* ATAATCCTTGCCCGCGGCCTACAAATAGTCCTTCCACTAATGGCAACCTGATGATTTATT

*L. calcarifer* ATACTACTGACACGAGGCCTACAAATAGCCCTCCCACTAATAACAGCATGATGATTCCTC

*S. maena* ATAGTCCTAGCCCGAGGCTTACAAATAATCCTTCCTCTTATGATAGTGTGATGATTTTTT

*P. auriga* ATGGTCCTAGCCCGAGGACTACAAATAGCCCTCCCACTCATAGCAGCATGATGATTTATC

*C. lucidus* ATAGCTCTCGCCCGAGGACTCCAAATAGCCCTACCCCTAATAACTACCTGATGATTTATT

*S. sihama* ATAGTTCTGGCCCGAGGGCTGCAGATAGCACTCCCACTGATAGCAGCCTGGTGATTCCTC

*C. loricula* ATACTATTAGCCCGAGGCCTACAAATAGCCCTGCCACTGATGACATCATGATGATTCATC

*A. trutta* ATACTCCTGGCACGAGGACTTCAGATTATTCTGCCCTTAATAGCGACATGGTGGTTTATC

*H. gemma* ATGCTTTTAGCTCGAGGTCTACAAATAATTCTCCCCCTTATGGCCACCTGGTGATTTATT

*P. flavescens* ATACTTCTGGCCCGCGGCCTTCAAATAGTACTACCCCTAATAACAGCCTGATGATTCCTT

*P. macrolepida* ATACTTCTCGCCCGCGGCCTCCAAATAGTACTGCCATTAATAACGGCCTGATGATTTATT

*S. canadensis* ATACTCCTCGCCCGCGGTCTACAAATAGTTCTACCACTAATAACAGCCTGGTGGTTCATT

*A. rogaa* GCCAGCCTCGCCAACCTAGCCTTACCTCCACTGCCAAATCTCATGGGGGAACTAATAATC

*C. argus* GCCAGTCTTGCCAATTTAGCCCTACCCCCACTACCAAATCTCATGGGTGAGTTAATAATC

*C. sonnerati* GCCAGTCTAGCTAACCTAGCCCTCCCTCCACTACCCAACCTCATAGGGGAACTAATAATT

*E. fuscoguttatus* GCCAGCCTTGCCAACCTTGCACTCCCCCCCCTTCCCAACCTTATAGGAGAATTAATAATC

*E. coioides* GCCAGCCTCGCCAACCTTGCACTCCCCCCACTCCCCAACCTCATGGGTGAATTAATAATC

*E. bruneus* GCTAGCCTTGCTAACCTTGCACTCCCCCCACTTCCCAACCTTATAGGTGAACTAATAATT

*E. moara* GCTAGCCTTGCTAACCTCGCACTCCCCCCACTTCCTAACCTTATAGGCGAACTAATAATT

*E. lanceolatus*  GCCAGCCTTGCTAACCTTGCACTACCCCCCTTCCCTAACCTCATAGGCGAGCTAATAATC

*A. leucogrammicus*  GCCAGCCTTGCTAACCTCGCACTCCCCCCACTCCCTAATCTTATGGGTGAATTAATAATT

*C. altivelis* GCTAGCCTTGCCAACCTCGCACTCCCTCCCCTCCCCAATCTAATAGGTGAATTAATAATT

*E. epistictus* GCCAGCCTTGCTAACCTTGCACTACCCCCACTACCTAACCTAATAGGAGAACTAATAATT

*E. octofasciatus* GCCAGCCTTGCTAACCTTGCACTTCCCCCACTACCTAATCTGATAGGTGAACTAATAATT

*E. septemfasciatus* GCCAGCCTTGCTAACCTTGCACTTCCCCCGCTACCTAATCTGATAGGTGAACTTATAATT

*T. dermopterus* GCCAGCCTCGCTAACCTTGCACTCCCCCCCCTACCCAATCTAATAGGAGAACTAATAATT

*E. awoara* GCTAGCCTTGCTAATCTTGCACTCCCCCCACTGCCTAATTTAATAGGAGAGCTCATAATT

*E. akaara* GCTAGCCTTGCTAATCTCGCACTTCCCCCACTACCTAATTTAATAGGAGAACTTATAATT

*E. trimaculatus* GCTAGCCTCGCCAACCTTGCACTTCCCCCACTACCAAACCTAATAGGTGAGCTAATGATT

*E. areolatus* GCTAGTCTCGCTAATCTTGCACTTCCCCCACTACCTAATCTAATGGGTGAACTAATAATT

*V. albimarginata* GCCAGCCTGGCCAACCTGGCCCTACCCCCGCTCCCAAACCTAATAGGAGAACTAATAATT

*V. louti* GCCAGCCTGGCCAACCTGGCTCTACCACCACTGCCAAATTTAATAGGAGAACTAATGATT

*P. leopardus* GCTAGCCTCGCAAACTTAGCCCTTCCCCCACTACCCAACCTTATAGCAGAACTAATAATT

*P. areolatus* GCCAGCCTCGCAAACTTAGCCCTCCCTCCACTACCCAACCTTATAGCTGAACTAATAATT

*E. radiosum* GCTAGCCTAGCCAACCTGGCCCTTCCCCCTCTTCCTAATCTTATAGGGGAACTAATAATT

*P. sieboldi* GCTAGCCTCGCCAATCTGGCACTTCCACCCCTCCCCAACCTCATGGCCGAGCTCATGGTG

*E. armatus* GCCAGCCTTGCCAATCTTGCCTTGCCCCCTCTCCCCAATCTCATAGGCGAACTAATAATT

*R. oxyrhynchus* GCTAGCCTCGCCAACCTAGCACTCCCTCCACTGCCAAACCTCATAGGAGAACTAATAATT

*K. cinerascens* GCCAGCCTTGCTAACCTGGCCCTCCCCCCTCTACCCAACTTAATGGGTGAACTAATGATT

*T. chatareus* GCTAGCCTTGCTAATCTGGCCCTTCCCCCACTACCAAACCTAATAGGAGAGTTAATAATC

*D. berycoides* GCCAGCCTCGCCAACCTGGCTCTTCCCCCACTACCAAACCTTATGGGCGAACTAATAATC

*H. typus* GCCAGCCTCGCCAACCTGGCCCTTCCCCCACTGCCAAACCTTATAGGAGAATTAATAATT

*M. argenteus* GCTAGCCTCGCCAACCTAGCACTTCCCCCACTACCCAACCTCATGGGAGAGTTAATAATT

*S. chuatsi* GCCAGCCTTGCTAACTTAGCCCTCCCCCCTTTACCTAACCTAATAGGAGAACTAATAATT

*O. fasciatus* GCCAGTCTCGCTAACCTGGCCCTACCCCCACTCCCCAACCTGATAGGAGAACTAATAATT

*P. trilineatum* GCCAGCCTAGCAAACTTAGCCCTCCCTCCCCTACCAAACCTTATAGGAGAATTAATAATT

*M. salmoides* GCCAGCCTAGCAAACCTAGCTCTCCCTCCCTTACCTAACCTAATAGGCGAGCTAATAATT

*P. tile* GCCAGCCTGGCCAATCTTGCCCTTCCCCCTCTACCCAATCTTATAGGAGAACTAATGATT

*L. argentimaculatus* GCCAGCCTAGCCAACCTCGCCCTCCCCCCTCTACCTAACCTCATGGGAGAACTAATAATC

*E. struhsakeri* GCCAGTCTTGCCAACCTAGCCTTGCCACCTCTACCAAATCTTATAGGCGAACTAATAATT

*B. albus* GCCTCTCTCGCCAACTTAGCCCTTCCCCCGTTACCCAACCTCATAGGAGAACTAATGATT

*C. auripes* GCTAGCCTCGCCAACCTAGCCCTCCCCCCGCTGCCAAACCTTATAGGAGAACTAGCCATT

*C. melampygus* GCAAGCCTAGCTAATCTTGCTCTCCCACCTCTACCAAATCTCATGGGAGAACTTATAATC

*L. calcarifer* GCCACGCTCGCCAACCTAGCATTACCCCCTCTACCCAACCTAATAGCAGAGCTAATAGTC

*S. maena* GCAAGCTTAGCCAACCTCGCCCTTCCCCCCCTCCCAAACCTTATAGGGGAACTAATAATT

*P. auriga* TCAACCCTAGCTAACCTGGCCCTCCCCCCCCTCCCCAACCTCATAGGTGAATTAATGATT

*C. lucidus* GCCAGCCTCGCCAACCTCGCCTTACCCCCACTCCCCAACCTTATGGCAGAACTAATAATT

*S. sihama* GCCAGCCTAGCAAACCTGGCACTCCCCCCCCAACCCCAATTTATGGGAGAACTAATAATC

*C. loricula* GCCAGTCTGGCCAATCTAGCCCTTCCCCCACTCCCCAACCTCATGGGAGAGCTAATAATC

*A. trutta* GCTAGCCTGGCTAATCTAGCCCTCCCCCCTCTCCCTAATCTTATGGGCGAACTCATAATT

*H. gemma* GCAAGCCTTGCCAACTTAGCCCTCCCTCCCCTTCCTAACCTGATGGCTGAACTTATAATT

*P. flavescens* GCCAGCCTTGCTAATTTAGCCCTTCCCCCACTTCCTAACCTTATAGGAGAACTAATAATC

*P. macrolepida* GCTAGCCTAGCTAATCTGGCCCTACCCCCCCTTCCTAATCTCATAGGAGAACTAATAATT

*S. canadensis* GCCAGCCTAGCTAACCTGGCTCTTCCCCCACTTCCAAACCTCATGGGGGAATTAATAATT

*A. rogaa* ATTACCTCCCTATTCAACTGATCATGATGAACACTAGCATTAACAGGAGCAGGTACCCTC

*C. argus* ATTACCTCTCTATTTAACTGATCATGATGAACGCTAGTACTAACAGGAGTGGGCACCCTC

*C. sonnerati* ATTACCTCCTTATTCAACTGATCCTGATGAACACTAGCATTAACAGGAGCAGGCACACTC

*E. fuscoguttatus* ATCACCTCCCTATTTAACTGATCTTGATGAACAATTGTACTAACAGGAGCGGGCACTCTT

*E. coioides* ATCGCCTCCCTATTTAACTGATCCTGATGAACAATTATTCTAACCGGAGGTGGCACCCTT

*E. bruneus* ATCACCTCCTTGTTTAATTGATCTTGATGAACAATTGCCCTAACCGGAGGAGGCACCCTT

*E. moara* ATCGCCTCCTTATTTAATTGATCTTGATGAACAATTGCCCTAACCGGAGGAGGTACCCTT

*E. lanceolatus*  ATTACCTCCCTATTTAACTGATCTTGGTGAACGATTGTACTAACTGGAGGAGGTACCCTC

*A. leucogrammicus*  ATTACCTCCCTATTTAACTGATCCTGATGAACAATTGCACTAACCGGAGCAGGCACCCTT

*C. altivelis* ATTACCTCCTTATTTAATTGATCCTGATGAACAATTGTACTAACCGGAGGGGGCACTCTT

*E. epistictus* ATTACCTCCCTATTTAACTGATCATGATGAACAATTGCACTAACAGGAGGAGGCACTCTC

*E. octofasciatus* ATTACTTCTTTGTTTAGCTGATCTTGATGAACACTTGTGTTAACAGGAGGGGGCACTCTT

*E. septemfasciatus* ATTACTTCTCTATTCAACTGATCATGATGAACACTTGTATTAACAGGAGGGGGCACTCTC

*T. dermopterus* ATTTCTTCCCTGTTTAACTGATCATGATGAACGCTCGTACTAACAGGGGTGGGCACCCTT

*E. awoara* ATCACCTCCCTATTTAACTGATCGTGATGAACAATTGTATTAACCGGAGGGGGAACCCTT

*E. akaara* ATTACCTCCCTGTTCAACTGATCATGGTGAACAATTGTATTAACCGGAGGAGGAACCCTT

*E. trimaculatus* ATTACCTCTTTATTTAACTGATCCTGATGAACAATTGTGTTTACAGGAGGGGGAACTCTT

*E. areolatus* ATTACCTCCCTATTTAACTGATCCTGATGAACAATTGTACTAACCGGAGGGGGGACCCTT

*V. albimarginata* ATCACATCCCTTTTTAACTGATCATGATGAACACTAGCATTAACTGGAGCAGGAACACTC

*V. louti* ATCACATCACTTTTTAACTGATCCTGATGAACCCTGGCACTAACCGGAGCAGGAACACTC

*P. leopardus* ATCGTCTCACTATTTAACTGATCATGATGGACACTTATCCTGACAGGAGCAGGAACCCTT

*P. areolatus* ATTGTATCGCTATTTAACTGATCATGATGAACTCTTATCCTAACAGGAGCAGGAACCCTT

*E. radiosum* GTCACCTCTTTATTTAACTGATCCTGGTGAACCCTTGCATTAACCGGGGCCGGGATGCTA

*P. sieboldi* ATCACCGGCATATTCAACTGATCTTGATGAACCCTTGCCTTAACAGGCACCGGCACCTTA

*E. armatus* ATTACTTCCTTATTCAACTGGTCCTGATGAACACTAGCACTTACAGGGGCTGGCACGCTC

*R. oxyrhynchus* ATTGTATCACTATTTAACTGATCCTGATGAACCCTTCTCCTAACAGGAGCCGGCACACTT

*K. cinerascens* ATCACCTCACTATTTAACTGATCCTGATGGACCCTTCTGCTAACAGGAGCCGGAACCCTC

*T. chatareus* ATTACCTCCCTATTCAACTGATCCTGATGAACTCTGGCCCTAACAGGGGCCGGAACCCTC

*D. berycoides* ATTACCTCCTTGTTCAACTGATCCTGATGAACCCTGGCTTTAACAGGAGCTGGCACCCTT

*H. typus* CTCACCTCACTATTCAACTGATCTTGATGAACCCTAGCTTTAACTGGGGCCGGGACCCTC

*M. argenteus* ATTACCTCTCTATTCAACTGATCCTGATGAACCCTTGTCCTAACAGGAGCCGGGACCCTA

*S. chuatsi* ATCACATCATTATTTAACTGATCCTGATGAACCCTCGCACTAACAGGGGCTGGTACCCTA

*O. fasciatus* ATCTCCTCCCTATTCAACTGATCCTGATGAACTCTACTATTGACAGGTGCCGGCACCCTT

*P. trilineatum* ATTACCTCACTATTTAACTGATCTTGATGAACCTTGGCCCTAACAGGAGCGGGAACCCTA

*M. salmoides* ATTACCTCCCTATTCAACTGATCCTGGTGAACCTTAGCCCTTACAGGAGCCGGAACATTA

*P. tile* ATCACCTCATTATTCAACTGATCATGATGAACCCTTATTTTAACAGGAGCGGGCACACTA

*L. argentimaculatus* ATTACATCCCTGTTTAACTGATCTTGATGAACCCTGGCCCTGACAGGAGCAGGCACCTTA

*E. struhsakeri* ATCACATCACTATTCAACTGATCGTGATGAACCCTCGCCCTAACAGGAGCCGGAACCCTG

*B. albus* ATCACCGCCGCATTAAACTGATCTTGATGAACTATTATTCTAACAGGAGCGGGCACCTTA

*C. auripes* ATTACCTCCCTGTTCAACTGGTCCCCTTGAACAATTATTTTAACCGGGGCAGGCACCCTC

*C. melampygus* ATCACATCCCTTTTCAACTGATCTTGATGAACTCTAGCTTTAACAGGAGCTGGTACACTC

*L. calcarifer* CTTACCGCACTATGCAACTGATCTCCATGGACAATCCTATTTACAGGGGCCGGTGTCCTA

*S. maena* ATTACGGCCCTCTTTGGATGATCTTGATGAACCTTAGCCCTCACAGGGGTCGGAACCTTA

*P. auriga* ATTTCTTCTCTCTTTAACTGATCATGATGAACCCTAGCCCTCACAGGGGCCGGGACCCTT

*C. lucidus* ATTACCTCACTATTCAACTGATCCTGATGAACCCTGGCCCTAACAGGTACAGGAACTCTT

*S. sihama* ATTACCTCCCTATTTACCTGGTCCTGATGAACCCTGGCCCTAACCGGCACAGGAACCCTA

*C. loricula* TTCACATCTCTCTTTAACTGATCATGATGAACCTTCATTCTTACAGGGGCAGGAACACTA

*A. trutta* ATTACCTCCCTTTTCAACTGATCCCCTTGAACACTAACCTTAACGGGAACGGGAACCCTA

*H. gemma* ATTACATCCCTGTTCTCCTGGTCCGCGTGAACTCTCGCTATCACAGGCTTAGGAACACTC

*P. flavescens* ATTACCTCGCTGTTTAACTGATCCTGATGGACTCTGGCATTAACCGGGGCTGGAACCCTA

*P. macrolepida* GTTACCTCCTTATTCAGCTGGTCCTGATGAACCCTTGCATTAACCGGGGCAGGCATGTTG

*S. canadensis* ATTACCTCCCTATTCAGTTGGTCCTGGTGGACTTTAGTATTAACCGGGGCTGGAACGCTA

*A. rogaa* ATCACTGCAAGCTACTCTCTCTACATATTCCTAACCACCCAACGAGGTCCACTTCCCTCT

*C. argus* ATTACCGCAAGTTATTCCCTCTACATGTTCTTAGCTACCCAACGGGGCCAACTCCCCTCT

*C. sonnerati* ATCACCGCTAGCTACTCTCTCTATATATTTTTAATAACTCAACGAGGCCCCCTCCCTTCA

*E. fuscoguttatus* ATCACCGCAAGCTACTCCCTTTACATGTTCCTTATAACCCAGCGAGGACCCCTTCCCTCA

*E. coioides* ATTACTGCAAGCTATTCCCTCTACATATTCTTAATAACCCAACGAGGCCCCCTCCCCTCA

*E. bruneus* ATTACTGCAAGCTACTCCCTCTACATATTCCTAATAACTCAACGAGGGCCCCTTCCCTCA

*E. moara* ATTACTGCAAGCTACTCCCTCTACATGTTCCTAATAACTCAACGAGGGCCCCTCCCCTCA

*E. lanceolatus*  ATCACCGCAAGCTACTCCCTCTACATGTTCCTGATAACTCAACGAGGGCCCCTCCCCTCA

*A. leucogrammicus*  ATCACCGCAAGCTACTCCCTCCACATGTTCCTAATAACCCAACGAGGAATCCTACCCTCA

*C. altivelis* ATTACTGCAAGCTACTCCCTCTACATATTCCTAATAACTCAACGAGGACCCCTCCCCTCA

*E. epistictus* ATCACCGCAAGCTACTCCCTTTACATATTCCTAATAACTCAACGGGGACCCCTCCCCTCA

*E. octofasciatus* ATTACTGCAAGCTACTCCCTCTACATATTCCTAATAACTCAACGAGGGCCCCTCCCCTCA

*E. septemfasciatus* ATTACTGCAAGCTACACCCTATACATATTCCTAATAACTCAACGAGGGCCCCTCCCCTCA

*T. dermopterus* ATTACTGCAAGCTACTCCCTATACATGTTCCTGATAACTCAACGAGGCCCCCTCCCCTCA

*E. awoara* ATCACTGCAAGCTACTCTCTCTACATGTTCTTAATGACTCAACGAGGCCCCCTCCCCTCA

*E. akaara* ATCACTGCGAGCTACTCTCTCTACATGTTCTTAATGACCCAACGAGGCCCCCTCCCCTCA

*E. trimaculatus* ATTACCGCAAGTTACTCTCTCTATATATTCTTAATAACTCAACGAGGCCCACTCCCCTCA

*E. areolatus* ATCACTGCAAGCTACTCCCTTTACATATTCCTCATAACCCAACGAGGCCCCCTCCCCTCA

*V. albimarginata* ATCACCGCAAGTTACTCCCTCTATATATTCCTCATAACCCAACGAGGCCCGCTTCCCCAA

*V. louti* ATTACCGCGAGCTACTCCCTTTATATATTCCTCATAACCCAGCGAGGCCCACTTCCCCAA

*P. leopardus* ATTACCGCCAGCTATTCCCTCTACATATTTCTAATAACTCAACGAGGTCCACTTCCCTCT

*P. areolatus* ATTACCGCTAGCTACTCTCTCTACATATACCTAATAACTCAACGGGGCCCACTTCCTCCC

*E. radiosum* ATTACCGCGAGCTACTCTCTCTATATGTTTCTTATAACTCAGCGGGGGCCCATTCCACCG

*P. sieboldi* ATTACTGCCGGCTATTCCCTCTACATGTTCCTTATGACCCAACGTGGCCAGCTCCCCGCC

*E. armatus* ATTACCGCGGGATACTCACTTTATATATTCCTTATGACCCAACGAGGGCCCCTCCCAGCA

*R. oxyrhynchus* ATTACCGCAAGCTACTCCCTATACATGTTCCTCATAACCCAACGCGGCCCCCTTCCAGCC

*K. cinerascens* ATTACCGCTGGCTACTCTCTCTACATATTCCTGATAACCCAACGAGGCCCACTCCCTGCG

*T. chatareus* ATCACCGCGGGCTATTCTCTCTACATATTCCTAATAACCCAACGAGGCCCTCTCCCAATA

*D. berycoides* ATTACCGCAAGTTACTCCCTCTACATATTCCTTATGTCCCAGCGAGGCCCACTCCCAGCA

*H. typus* ATCACCGCGGGCTATTCACTCTACATATTTCTCATGACCCAGCGGGGGCCACTCCCTGCA

*M. argenteus* ATCACTGCGGGCTATTCACTCTACATGTTCCTTATAACACAACGAGGCCCACTCCCAGCC

*S. chuatsi* ATCACTGCAGGCTACTCACTCTATATGTTCCTTATGACTCAACGGGGCCCACTTCCAGCC

*O. fasciatus* ATTACCGCAGGCTACTCTCTCTACATATTCCTAATAACCCAACGAGGACCGCTCCCAGCA

*P. trilineatum* ATTACCGCAGGTTACTCATTGTATATGTTCCTGATAACTCAGCGAGGCCCCCTCCCAGCA

*M. salmoides* ATTACTGCCGGCTATTCACTTTATATGTTTCTCATAACACAACGAGGCCCCCTCCCCGCA

*P. tile* ATCACTGCCGGTTACTCACTATATATGTTCCTGATGACTCAACGAGGCCCACTGCCAGCC

*L. argentimaculatus* ATTACCGCTGGCTACTCACTCTACATATTCCTTATAACCCAACGAGGCCCGCTGCCAGCA

*E. struhsakeri* ATCACTGCAGGCTATTCACTCTACATATTCCTCATGACTCAACGGGGCCCCCTCCCGGCA

*B. albus* ATCACAGCAGGCTACTCCCTCTACATATTCCTCATAACCCAACGAGGCCCACTCCCACCC

*C. auripes* ATTACTGCCGGCTATACCCTCTATATATTCCTTTCCATCCAACGCGGCACCCTTCCAACA

*C. melampygus* ATTACCGCCGGTTATTCCCTCTACATGTTCTTAATAACTCAACGAGGTCCTCTCCCAGGA

*L. calcarifer* ATTACCGCAGCCTACTCCCTCTATATGTTCCTGTTGACACAACGAGGTCGCCTACCTCCC

*S. maena* ATTACAGCAGGATATTCTCTTTACATGTTCCTTATAACCCAACGAGGCCAACTCCCAGCA

*P. auriga* ATTACTGCAGGATACTCTCTTTACATATTTTTAATAACACAACGAGGCCAACTCCCAACA

*C. lucidus* ATCACCGCGGGGTACTCCCTATACATATTTTTAATAACCCAACGCGGCCCCCTCCCAACA

*S. sihama* ATCACCGCGAGCTACTCCCTCTACATATTCCTTATAACCCAACGGGGCCCAACTCCAGCA

*C. loricula* ATCACAGCAGCCTACTCCCTCTATATATTTTTAATAACCCAACGAGGCCCTCTCCCGGCC

*A. trutta* ATCACCGCAGCATATTCTCTATATATGTTCCTAATGACACAACGGGGCCCTCTTCCAGCG

*H. gemma* ATTACTGCTAGCTACTCTCTTTACATGTTTTTAATAACACAACGGGGACAGCTAACTCAC

*P. flavescens* ATTACCGCGAGCTACTCTCTTTACATGTTTCTTATAACCCAGCGGGGCCCCATTCCAGCA

*P. macrolepida* ATTACCGCAAGTTACTCCCTCTACATGTTTCTTATAACCCAGCGAGGTCCCATTCCCCCA

*S. canadensis* ATCACCGCAAGTTATTCCCTTTATATATTTCTCATAACCCAACGAGGACCCATTCCAGCA

*A. rogaa* CACATCATCGCTCTTGACCCCTCCCACTCACGAGAACATCTACTAATAGCCCTCCACCTA

*C. argus* CACATTATTGCTCTCAACCCCTCTCACTCACGAGAACATTTACTATTGGCCCTTCATCTC

*C. sonnerati* CATATTATTGCTCTCGACCCCTCACACTCACGAGAACATCTTCTAATGGCCCTCCACCTC

*E. fuscoguttatus* CACATCATTGGCCTCGACCCCTCCCACTCACGAGAACATCTACTTATAACACTCCACCTC

*E. coioides* CACATCATCGGCCTTGACCCCTCCCACTCGCGAGAGCATTTACTCATAGCCCTCCACCTC

*E. bruneus* CACATCATTGGTCTCGACCCCTCTCACTCACGAGAACATCTACTTATAACCCTCCACCTT

*E. moara* CACATCATTGGTCTCGACCCCTCTCACTCACGAGAACATCTACTTATAACCCTCCACCTT

*E. lanceolatus*  CATATCCTTGGCCTTAACCCCTCCCATTCACGAGAACATCTACTTATAACCCTTCACCTC

*A. leucogrammicus*  CATATCCTAGGCCTTGACCCCTCCCACTCACGAGAACATCTACTCATAACACTCCACCTC

*C. altivelis* CATATTATTGGCCTTGACCCCTCCCACTCACGAGAACATTTACTTATAACCCTCCACCTT

*E. epistictus* CACATTATTAACCTAGACCCCTCCCACTCACGAGAACATCTGCTTATAACCCTTCACCTC

*E. octofasciatus* CACATTATTAACCTCGACCCCTCCCACTCACGGGAGCATCTACTAATAGCCCTCCACCTC

*E. septemfasciatus* CACATTATTGGTCTCGACCCCTCCCACTCACGAGAACACCTGCTAATGACCCTCCACCTC

*T. dermopterus* CACATCATTGGTCTTGACCCCTCCCACTCACGAGAGCACTTATTGATAGCCCTTCACCTC

*E. awoara* CACATTGTTGGTCTCGAACCCTCCCACTCACGAGAACATCTACTTATGGCTCTTCACCTC

*E. akaara* CACATCATTGGCCTCGAACCCTCCCACTCACGAGAACATCTACTTATGGCCCTTCACCTT

*E. trimaculatus* CACATTATTGGCCTCGACCCCTCCCACTCACGAGAACATCTACTCATAGCCCTTCACCTT

*E. areolatus* CACATCATTGGCCTTGACCCCTCCCACTCGCGAGAACACTTACTTATAGCCCTCCACCTC

*V. albimarginata* CACATTATTGCCTTAGACCCCTCCCACTCCCGAGAACACTTACTAATAACCCTTCACCTT

*V. louti* CACATCATTGCCCTAGACCCCTCCCACTCCCGAGAACACTTACTAATCACCCTTCACCTT

*P. leopardus* CACATTATTGCCCTAGCCCCCTCACACTCCCGAGAACATTTACTTATTGCACTACATCTA

*P. areolatus* CACATTATTGCCCTAGCCCCCTCACACTCCCGAGAACATCTACTTATCGCACTACATCTA

*E. radiosum* CACCTCGCTGCCTTGGACCCCTCCCATTCTCGAGAACATCTGCTTATGGCCCTCCACCTT

*P. sieboldi* CACATCATTAGCATCCCACCCTCATTCTCTCGAGAACACCTCCTGATGGCCCTCCACCTG

*E. armatus* CATGTTATAGCTCTTGACCCCTCACACTCCCGAGAACACCTCCTCATAGCCCTGCACCTT

*R. oxyrhynchus* CATATAACCGCCCTATCCCCCTCACACTCCCGAGAACATCTTCTCATTACCCTTCACCTC

*K. cinerascens* CACATCATTGCACTAGATCCATCACACTCCCGAGAACACCTCCTTATGGCCCTCCACCTC

*T. chatareus* CACATCATTGCCCTAGACCCCTCCCACTCTCGAGAACACCTTCTAATTATTCTCCACCTC

*D. berycoides* CACATTATTGCCCTAGTTCCCTCCCACACTCGAGAACATCTCCTAATAGCCCTCCACCTC

*H. typus* CACATCCTTGCCCTCGACCCCTCCCACTCTCGAGAACACTTACTCATGGCCCTCCACCTC

*M. argenteus* CACATTATTGCCCTCGACCCCTCCCACTCCCGAGAACATCTACTAATAGCCCTACACCTC

*S. chuatsi* CATATTATTGCCCTTGACCCCTCCCACTCTCGAGAACATTTACTAATAGCCCTTCACCTC

*O. fasciatus* CATATTATTGCACTAGACCCCTCACACTCTCGAGAGCACCTCCTCATAGCCCTCCACCTC

*P. trilineatum* CACATTATTGCCCTCGACCCCTCGCACTCTCGAGAGCACCTCCTAATAGCCCTTCACCTC

*M. salmoides* CACATTATTGCACTAGACCCCTCCCACTCCCGAGAACATTTACTTATGGCCCTCCATCTT

*P. tile* CACATCATCGCCTTAGACCCCTCTCACTCTCGAGAACACCTCCTGATAGCCCTCCACCTT

*L. argentimaculatus* CACATCATCGCCCTAGATCCCTCACATTCTCGAGAACATCTCCTAATGGCCCTCCACCTG

*E. struhsakeri* CATATCATTGCCCTAGACCCCTCCCACTCTCGAGAACACCTCCTAATAGCCCTGCACCTC

*B. albus* CATATTAAAAACATCGCCCCCTCCTACTCTCGAGAACACCTATTGATGGCCCTACACCTC

*C. auripes* CATATTATTGCCCTAGACCCCTCACACTCCCGAGAACACCTGCTGATAGCACTCCATATG

*C. melampygus* CACATTATTGCCCTCGAACCCTCCCACTCTCGAGAACACCTACTTATTCTCCTCCACATT

*L. calcarifer* CATATCAAATCCCTAGACCCCTCACACTCTCGAGAACACTTAGTTATCGCACTCCACTTA

*S. maena* CATATTACCACCGTAGAACCCTCCCACACACGAGAACATCTACTAATAGTGCTCCATCTT

*P. auriga* CATATTACTGCCCTAGAGCCCTCCCATTCACGAGAACACCTTCTAGTGGCCCTTCACCTT

*C. lucidus* CATATCATTGCCCTTGAACCCTCCCACTCCCGAGAACATCTATTAGTAGCCCTCCACCTA

*S. sihama* CACATGCTTGCACTTGACCCCTCCCACACACGAGAACACCTTCTAATAACCCTCCACCTT

*C. loricula* CACATCATCGCCCTAGACCCTTCTCACTCACGAGAGCACTTACTAATCACCCTTCACCTT

*A. trutta* CATATTATTGCCCTCGACCCCTCTCACACCCGAGAACACCTTGTCATAGCCTTACACCTC

*H. gemma* CATGTCGTTCACATTTCCCCCTCCCACACCCGAGAACACCTTCTTTTAGCCCTTCACTTA

*P. flavescens* CACATAATTGCATTAGAACCCACCCACTCTCGAGAGCACTTACTGATAGCCCTCCACCTC

*P. macrolepida* CACCTTATTGCCTTAGACCCCTCTCATTCTCGAGAACACTTGCTTATGGCCCTCCACCTT

*S. canadensis* CACATAATAGCCCTAGACCCCTCCCACTCTCGAGAACACTTGCTGATAGCCCTACACCTT

*A. rogaa* TTACCCCTTCTCCTACTTATCCTTAAACCTGAATTAATCTGAGGATGAACGGGCTATGCA

*C. argus* CTGCCTCTCCTCTTACTTATCTTCAAGCCCGAACTAATCTGAGGATGAGCGAGCTATGCA

*C. sonnerati* CTCCCTCTACTTCTCCTCATTCTTAAACCCGAACTAATCTGAGGGTGAACAGGCTATGCA

*E. fuscoguttatus* CTACCATTACTACTCCTCATTCTTAAACCCGAATTAATTTGAGGCTGAGCCGGCTATGCA

*E. coioides* TTACCCCTCCTTCTCCTCATTCTTAAGCCCGAACTAATTTGAGGATGAACCAGTTATGTA

*E. bruneus* TTGCCACTACTTCTCCTCATTCTTAAGCCCGAACTAATTTGAGGTTGAGCCGGCTATGCA

*E. moara* TTGCCACTACTTCTCCTCACTCTTAAACCCGAACTAATTTGAGGTTGAGCCGGCTATGCA

*E. lanceolatus*  TTGCCACTACTTCTCCTCATCCTTAAACCCGAACTAATCTGAGGTTGAACTGGTTATGCA

*A. leucogrammicus*  TTACCACTCCTTCTCCTCATTCTTAAACCCGAGCTAATTTGAGGTTGACCTGGCTATGCA

*C. altivelis* CTACCACTACTCCTCCTAATCCTTAAGCCTGAACTAATTTGAGGTTGGACTAACTATGCA

*E. epistictus* CTACCACTACTACTTCTCATCCTCAAACCTGAGCTGATCTGAGGTTGAGCCGGCTATGCA

*E. octofasciatus* TTGCCCCTACTCCTCCTCGTTCTTAAGCCCGAATTAATTTGAGGTTTAGCCGGCTATGCA

*E. septemfasciatus* TTACCCCTACTCCTCCTCGTTCTCAAGCCCGAGTTAATTTGAGGTTGAACCAGCTATGCA

*T. dermopterus* TTACCCCTACTCCTACTTATTCTTAAACCCGAGTTAATCTGAGGTTGAGCCGGCTATGTA

*E. awoara* CTACCACTCCTCCTCCTCATTCTTAAGCCCGAGTTGATTTGAGGCTGAGCCGGCTATGCA

*E. akaara* CTTCCGCTTCTACTCCTCATTCTTAAGCCCGAATTGATCTGAGGCTGAACCAACTATGCA

*E. trimaculatus* CTACCCCTTCTCCTCCTCATTCTTAAACCTGAACTGATCTGAGGTTGAACCCATTATGCA

*E. areolatus* CTGCCGCTCCTCCTCCTCACCCTTAAGCCTGAACTGATCTGAGGCTGGGCCAACTATGCA

*V. albimarginata* CTTCCTCTTATCTTATTAATTCTTAAACCTGAGTTAATTTGAGGATGAACTGGATATGTA

*V. louti* CTTCCTCTTATCCTATTAATTCTTAAACCTGAGTTAATTTGAGGATGAACGGGCTATGTA

*P. leopardus* CTTCCACTAATCCTAATTATCCTTAAACCAGAACTCATCTGAGGATGGGCCGGCTATGTA

*P. areolatus* CTTCCATTAATTCTAACTATCCTTAAACCAGAACTTATCTGAGGGTGAGCCGGCTATGTA

*E. radiosum* GTTCCACTAGTTCTTCTAATACTCAAACCTGAACTAATCTGGGGCTGGGCTTATTATGCA

*P. sieboldi* CTCCCCCTCCTCGCCCTTATCTTTAAACCAGGCCTAATTTGAGGCTGACTCGGATATGTC

*E. armatus* CTCCCCCTCCTTCTCCTTATTATTAAACCCGAACTAGTTTGAGGATGGGCCGCTTATGCA

*R. oxyrhynchus* CTCCCCCTCCTCCTTCTAATCCTTAAACCAGAACTAATTTGAGGCTGGACTTTTTATGAC

*K. cinerascens* CTCCCCCTACTCCTACTAATCCTCAAACCTGAACTGATTTGAGGTTGAGCCGCTTATGCA

*T. chatareus* CTTCCCCTGATTCTGCTTATTCTTAAGCCCGAACTAATTTGAGGATGAACAGCCTATGCA

*D. berycoides* CTCCCCCTAATTATACTGATCCTTAAACCAGAACTGATTTGAGGTTGATCTGCATATGCA

*H. typus* TTGCCCCTTATATTACTTATTCTTAAGCCTGAACTAGTCTGAGGGTGGACCGCCTATGCA

*M. argenteus* CTACCCCTAATTCTACTAATTCTTAAGCCCGAACTAATCTGAGGATGAGCTGCTTATGCA

*S. chuatsi* CTCCCCCTAATCCTGCTAATCCTCAAACCCGAATTAATTTGAGGGTGGGCCGCTTATGCA

*O. fasciatus* CTCCCCCTACTACTACTAATTCTCAAACCCGAATTAATTTGAGGCTGAACAGCTTATGCA

*P. trilineatum* CTCCCCCTAATTCTACTAATCCTCAAGCCTGAACTAATTTGAGGGTGAGCCGCCTATGCA

*M. salmoides* CTCCCCCTCCTGCTCCTAGTCCTCAAGCCGGAACTAATTTGAGGGTGAGCCGCCTATGCA

*P. tile* CTTCCCCTCCTTCTCCTTATTCTCAAACCCGAGCTAATTTGAGGTTGAACCGCCTATGCA

*L. argentimaculatus* CTTCCGCTCATCCTCCTAATCCTTAAACCAGAGCTAATTTGAGGCTGGACCGCCTATGCA

*E. struhsakeri* CTCCCCTTAATCCTGCTAATCCTCAAACCTGAACTAGTTTGAGGGTGAGCCACTTATGCA

*B. albus* CTCCCCCTAGTCCTCCTGATCCTTAAACCCGAGCTGATCTGAGGATGGACCGCCTATGCA

*C. auripes* CTGCCACTCTTCCTACTGATCCTTAAACCTGAACTAATTTGAGGCCTAGCCGGCTATGCA

*C. melampygus* CTTCCCCTAATTCTCCTTATTCTTAAACCAGAGCTCATCTGAGGATGAACCGCCTATGCA

*L. calcarifer* ACCCCTGTTACACTTCTAATCTTCAAACCAGAACTAATTTGAACCTGACTGATCTATGCA

*S. maena* CTCCCCTTAATCCTTCTTATCCTCAAACCCGAATTAATTTGAGGATGAACAGCCTATGCT

*P. auriga* CTCCCCTTAGGCCTCTTAATCCTTAAACCAGAATTAATTTGAGGGTGGACTGCCTATGCA

*C. lucidus* CTCCCCCTAATCCTCCTAATCCTTAAACCTGAACTAATTTGAGGCTGGACCGCTTATGCA

*S. sihama* CTCCCCCTAATACTTCTTATCCTAAAACCCGAACTGATCTGAGGCTGAACCACCTATGCA

*C. loricula* CTTCCCCTTCTTCTCCTAATTCTGAAACCAGAACTAATCTGAGGTTGGACTAACTATGCA

*A. trutta* CTCCCCCTAATTTGCCTTATCCTAAAGCCAGAGCTAATCTGCGGATGAAACACGTATGCC

*H. gemma* TTCCCCTTAATCCTTCTAATCATTAAGCCAGAGCTTGTCTGAGGCTGGGCAGCTTATGCA

*P. flavescens* CTCCCCTTACTCCTCCTAATACTTAAACCCGAGCTAATCTGAGGGTGGACATACTATGCA

*P. macrolepida* CTCCCCCTAATTCTCCTAATACTTAAGCCCGAGCTAATCTGAGGGTGGGCGTACTATGCA

*S. canadensis* CTTCCATTAGCCCTCCTTATACTTAAACCTGAGCTAATCTGGGGGTGGATATACTATGCA

*A. rogaa* CCCCCCTTCTGTAATCATAACATCAAGCTTAATCATCATCTTTTCTTTACTAGCCTATCC

*C. argus* CCCCTCTTCTATCATCATAACATCAAGCTTAATCATCATTTTTTCCTTACTGGCCTACCC

*C. sonnerati* CCCTACTTCTGTAATTATAACATCAAGCTTAATCATTATCTTCTCACTACTAATTTACCC

*E. fuscoguttatus* CACCCCTTCCATCACAATAACTTCAAGCCTAATCATTATTTTTTCCCTGCTTATTTTCCC

*E. coioides* CACCTCTTCCGTTATAATAACTTCAAGTCTGCTCATTATTTTTTCCCTGCTTATCTTCCC

*E. bruneus* CCCCTCTTCCCTTATAATAACTTCAAGCCTAATCATTATTTTTTCCCTGCTCATCTTTCC

*E. moara* CCCCTCTTCCATTATAATAACTTCAAGCCTAATCATTATTTTTTCCCTCCTCATCTTTCC

*E. lanceolatus*  CACCCCTTCCATTATAATAACTTCAAGCCTAATCATCATTTTTTCCCTACTCACCTTCCC

*A. leucogrammicus*  TGCACCTTCTATCACAATAACTTCAAGCCTGATCATTATCTTTTCCTTACTCATCTTCCC

*C. altivelis* CACCTCTCCTGTTATTATAACTTCAAGCCTAATTATCATTTTCTTCTTACTCACCTTCCC

*E. epistictus* CACCCCGTCCGTCATCCTAACTTCAAGCCTAATTATCATCTTTGCCTTACTAATCTTTCC

*E. octofasciatus* CACCCCTCCCATCATCATAACCTCAAGCCTGATCATTATTTTTTCTTTACTAATCTTCCC

*E. septemfasciatus* CACCCCTTCCGTCATCATAACCTCAAGCCTGATCATTATTTTCTCTTTACTAATTTTCCC

*T. dermopterus* CACCTCTCCTACCATCATAACCTCAAGCCTCATTATCATTTTTTCCCTGCTAATCTTCCC

*E. awoara* CACTCCTTCCATTATTATGACCTCAAGCCTGCTCATGATTTTTTCCCTGTTGATTTTTCC

*E. akaara* CACTCCTTCCATTATTATGACCTCAAGCCTGCTCATGATCTTTTCCCTGTTGATTTATCC

*E. trimaculatus* CATCCCTTCCGTCATTATAATCTCGAGTCTACTCATGATTTTCGCACTATTAGCTCTCCC

*E. areolatus* CACCCCTTCTATCATTATAACTTCAAGCCTACTTATTATTTTTTCCCTACTAATATTTCC

*V. albimarginata* CCCGACCCCTCTTATTATAACAACAAGCTTAGTACTTATTTTTGTTTTACTAACTTACCC

*V. louti* CCCGACCCCTCTCATCATAACAACAAGCTTAGTACTTATTTTTGTTTTACTAACATACCC

*P. leopardus* CAGTACTTCTCTCTTAATATCATCAAGCTTTATTATCATTATTATCTTATTATTTTACCC

*P. areolatus* CAGCACTTCTCTTTTAATATCATCAAGCTTTATTATTATTATCACCCTATTATTTTTCCC

*E. radiosum* CCCTACTGCCCTCATAATAACCTCGAGTTTAATTATTATTTTTTTGCTATTAACATTTCC

*P. sieboldi* ACCCTCAGCCCTTATGATAACCTCTAGTCTCATCTTCATTTTCTTCCTTCTAACTTACCC

*E. armatus* CACCTCTTCCCTAATCTTAACATCAAGCTTAATTACCATTTTTCTACTTCTAATTTATCC

*R. oxyrhynchus* TTCCACATCACTCACCATAGCCTCAAGCTTAGTCATCATTTTTGCTCTCCTTGCATATCC

*K. cinerascens* CACTACATCACTAATAATAACCTCCAGCCTAATCATTATCTTTACACTTCTCGCGTACCC

*T. chatareus* CCCTACCCCCCTCATGACAACATCCAGCCTTATAATTATCTTTACACTCCTGGCATACCC

*D. berycoides* CCCCACCCCTCTTATAATATCCTCAAGTTTAATCATTATTTTTACACTTTTAACCCACCC

*H. typus* TCCTACGTCACTTATAATAACATCGAGCTTAATCATCATCTTCACGCTTCTAACTTACCC

*M. argenteus* CCCCACTTCACTCATAATGACCTCCAGCCTGATCATTATCTTTGTGCTCCTAGCCTACCC

*S. chuatsi* CCCCACTTCCCTAATAATAACATCCAGCTTAATCATTATTTTTTTACTTTTAGCGTACCC

*O. fasciatus* ACCTACGTCACTTATAATAACCTCGAGCTTAATCATTATTTTCACGCTCCTCGCATACCC

*P. trilineatum* CCCCACTTCACTCATAATGACCTCCAGCTTAATTATCATTTTTACACTCTTAGTATACCC

*M. salmoides* TCCCACCTCACTCGTGATAACATCCAGCTTAATTACTATCTTCTTCCTCCTAACATTCCC

*P. tile* TCCCACCTCCCTAATAATAACCTCCAGCTTAATCATCATTTTTTCACTACTAGTCTATCC

*L. argentimaculatus* TCCCACCTCCCTAATAATAACCTCCAGCTTAATCATCATCTTTGCACTACTAATTTACCC

*E. struhsakeri* CCCCACTTCACTTATAATGACGTCGAGCTTGATCATTATCTTTACGCTCTTAGCATACCC

*B. albus* CTCCACCTCTCTCATAATGACCTCAAGCCTAGTCCTTATTTTTATATTACTGGCCTACCC

*C. auripes* TCCTACGCCTCTTATAATATCTTCCACCCTAATTCTCATTTTTATTACCCTACTATACCC

*C. melampygus* CCCTACCTCGCTTATAATAACCTCCAGCCTTATTATTATTTTTACACTCCTAGTCTACCC

*L. calcarifer* CCCAGCTTCACTTGCAATAACATCAAGCTTAATTATTATTTTTGCTGTCCTCGCATACCC

*S. maena* CTCAACTTCACTCATATTAACATCCAGCCTAATCCTGGTCTTCTTCCTACTTTCGTACCC

*P. auriga* CCCCTCCTCACTAATAATAGCATCCAGCTTAATTACCATTTTCCTGCTCCTTTTGTATCC

*C. lucidus* CACTACTTCACTTATAATGACCTCAAGCCTAATCACTATGCTCCTCCTCTTAGCCTACCC

*S. sihama* CCCAACTGCAATTATCTTATCATCCAGCTTAATCATTATTTTTGCACTACTATCGTACCC

*C. loricula* CTTAACACCCCTCACCATAACATCAAGCCTAATCCTTATCTTTGCACTACTGCTATACCC

*A. trutta* ACCGTCTCCTTTAACAATAACATCGACCATCACCATGATCTTCTTAATACTGGCGTATCC

*H. gemma* CCCTACTTCCTTAATAATAACATCAAGCTTAATTATTATCTTCTTCCTTCTAGCTTACCC

*P. flavescens* CCCCACTTCCCTGATAATGACCTCGAGCTTACTTATTATTTTTACACTACTAGCATACCC

*P. macrolepida* CCCCACTTCCCTTATAATAACCTCAAGTTTAATTATTATCTTTGTGTTACTAGCATACCC

*S. canadensis* CCCCACTTCCCTTATAATAACGTCGAGCTTGATTATTATTTTTACACTATTAACGTACCC

*A. rogaa* CGTACTTTCAACCCTAAACCCCCAACCCCAAAAAGACGACTGAGCCCTCTCACACGTAAA

*C. argus* TGTATTTTCAACCCTAAGCCCTCAACCCAAAAAAGACAACTGAGCTCTCTCACATGTAAA

*C. sonnerati* TGTCCTTTCAACCCTGAGCCCCCAACCCCAAAAAAACGACTGAGCCCTCTCACATGTAAA

*E. fuscoguttatus* CGTTCTAACAACTCTCAACCCCCTCCCCCAAAAAGAAAGCTGAGCCCTTACACATGTAAA

*E. coioides* TGTCTTAACAACCTTTAGCCCCCTCCCCCAAAAAGAAAGCTGGGCCCTCACACATGTAAA

*E. bruneus* TGTCCTAACAACTCTTAGCCCCCTTCCTCAAAAAGAAGCCTGAGCCCTTACACACGTAAA

*E. moara* TGTCCTAACAACTCTTAGCCCCCTTCCTCAAAAAGAAGCCTGAGCCCTTACGCACGTAAA

*E. lanceolatus*  TGTTCTAACAGCACTTAGTCCTCTCCCTCAAAAAGAAAATTGGGCCCTCACACATGTAAA

*A. leucogrammicus*  CCTCTTAACAACTCTTAACCCCCTCCCTCAAAAAGAAAGCTGAGCCCTCACACATGTAAA

*C. altivelis* GATTCTAACAACTCTCAACCCCCTCCCCCAAAAAGAAACCTGAGCCCTTACACATGTAAA

*E. epistictus* TGTCCTGACAACCCTTAGCCCCCTCCCTAAAAAAGAAAACTGAGCTCTCACACATGTTAA

*E. octofasciatus* TGTCCTAACAACTCTTAGCCCCTTCCCTCGAAAGGAAGACTGAGCCCTCACACATGTAAA

*E. septemfasciatus* TGTCCTAACAACTTTTAGCCCCCTCCCTCGAAAGGAAAACTGAGCCCTCACACATGTAAA

*T. dermopterus* CACCCTAACAGCTCTAAGCCCCCTCCCCCAAAAAGAAAACTGGGCCCTCACACATGTAAA

*E. awoara* TGTGCTAACAACCCTAAACCCCCTTCCCCGGAAAGAAGACTGAGCCCATACACATGTAAA

*E. akaara* TGTGCTGACAACCCTAAACCCCCTCCCCCGAAAAGAAGACTGAGCCCATACACATGTAAA

*E. trimaculatus* CGTATTAACAACCCTTAACCCACTCCCCCAAAAAGGAGACTGAGCCCTTTCACACGTAAA

*E. areolatus* CGTACTAACAACCCTTAACCCCCTTCCTCAGAAAGAAGACTGAGCCCTTACACACGTAAA

*V. albimarginata* TGTATTTACAACCCTCCACCCCAACCCCCGAAAACAGGACTGAGCCCTATCTCATGTTAA

*V. louti* TGTATTTACCACCCTCCATCCCAACCCCCGAAAACATGACTGAGCCATCTCCCATGTTAA

*P. leopardus* TATTATATCTACCCTAACTCCTGCACCCTTACCAAAAAACTGAGCTCTGTCCCACGTAAA

*P. areolatus* TATTCTATCTACTTTAACCCCCACACCCCTGCCAAAAACCTGAGCCTTGTCCCACGTAAA

*E. radiosum* TGTTTTTACTACACTAAACCCCGAGCCCCGAGGACCCAACTGGGCCCTCTCACACGTCAA

*P. sieboldi* AGTCATGACCACTCTTACCCTTAAACCCGAAGTTAACCCCGGACTTCCGGCGAACATTAA

*E. armatus* TGTTTTAACCACATTATCACCACAACCCCAAAAGGCAGATTGAGCCCTCTCCCAAGTGAA

*R. oxyrhynchus* TGTGCTCACAACCCTCACTCCCCAGCCCCGCCCTACAGACTGAGCCCTCTCCCACGTCAA

*K. cinerascens* TGTACTAACAACCCTCTCACCACAACCCCGGGAACCAAACTGAGCACTAACCCAAGTTAA

*T. chatareus* CATCTTCACAACTCTATCACCCTTCCCCCAAAAACATGATTGAGCCCTCTCCCACGTCAA

*D. berycoides* CCTACTCACAACCTTCACCCCACGGCCCTTGGCAACCAACTGAGCTCTGACTCAAACTAA

*H. typus* CGTGCTCACAACCTTCACTCCCCGATCCCATGGCACTAACTGGGCCCTTACCCAGGTTAA

*M. argenteus* CGTACTCACAACCCTAAACCCGACCCCCCGAAAAGCTGACTGAGCCCTCTCTCACGTTAA

*S. chuatsi* CGTATTCACAACCCTCACACCTCAACCATCCGGAACCAATTGAGCCCTCTCCCAAGTCAA

*O. fasciatus* CGTTCTCACAACGCTCACGCCCCAACCCCAACCATCAAACTGAGCATTGATCCAAGTAAA

*P. trilineatum* TGTACTAACAACCCTTAACCCTCACCCTCAAGAGCCCAACTGAGCCCTTGTCCAAGTTAA

*M. salmoides* CGTACTTACAACCCTAGCACCCCGCCCCCCAGAGCCCACCTGGGCCCTCTCCCATGTAAA

*P. tile* AGTCCTTACGACCCTAACCCCCCGCCCCCAAGATGCTAACTGAGCCCTCTCCCAAGTCAA

*L. argentimaculatus* CGTCCTTACTACCCTAAACCCCCTCCCCCGAGAAACCGACTGGGCCTTGTCCCATGTCAA

*E. struhsakeri* TGTACTCACAACCCTCAATCCCCGCCCCCGAGAAGCCGACTGAGCCTTATCTCAAGTTAA

*B. albus* AATCGTTACAACCCTGACCCCCATACCTAAAAGTCCCAACTGAGCCCTCACACATGTTAA

*C. auripes* GTTACTCACGACTCCAACCTCCCACCCCCTAAAAGAAACCTGAGCCCTCTATCAAGTAAA

*C. melampygus* AGTCCTAACCACACTAAATCCCCGCCCACAAGACCCAGAGTGAGCATTGACACAAGTGAA

*L. calcarifer* CATTCTGACAACCCTTTCTCCCCTCCCTCAAAACCAAAACTGGGCACTCTCCCACGTAAA

*S. maena* CCTTTTCACGACATTAACCCCAAACCCTTTACAAAATGACTGGGCACTTCTTAAAGTCAA

*P. auriga* CGTTCTAACAACGCTCAGCCCGAATCCCCAAAAAAACGACTGAGCACTTCTTAAAGTCAA

*C. lucidus* TGTTTTCACAACTCTTAACCCCGACCCCCGAGGCCCCGACTGATCCCTCTCCCAAGTTAA

*S. sihama* AATCATCTCAACCCTTAACCCCCAACCCCGCAAAAATCACTGAGCCCTCTCCCATGTAAA

*C. loricula* TGTCCTCACGACCCTCAACCCTAACCCCCGAAAAGCCAACTGGGCCCTCACCCAAGTAAA

*A. trutta* AGTATTAACAGCCCTATTCCCTGATCCTAAAAAACCTAACGCTCTCCGCGTCCGAGTAAA

*H. gemma* CATCTTCACCACTCTTAGTCCTGACCCTAAACACCCAGATTGGGCTGTCACCCATGTTAA

*P. flavescens* TGTGCTCACAACACTTAACCCCGCACCCCGAGAGCAAGACTGAGCCCTCTCCCATGTTAA

*P. macrolepida* CATCTTCACAACATTGAACCCTAGCCCCAAAGGGCAGGATTGGGCTCTTTCCCACGTTAA

*S. canadensis* TGTACTAACCACCTTTAACCCCCACCCCCAAGGACACAACTGGGCACTCTCTCATGTCAA

*A. rogaa* AACAGCAGTAAAACTAGCATTTTTCATTAGCCTCCTACCCCTCTTTCTTTTCCTAAATGA

*C. argus* GACAGCAGTAAAGCTAGCATTTTTTATTAGCCTTCTTCCACTATTCCTTTTCATAAATGA

*C. sonnerati* AACTGCAGTAAAACTGGCATTTTTTGTCAGCCTCCTCCCCCTCTTCCTCTTCCTCAATGA

*E. fuscoguttatus* AACAGCAGTAAAACTAGCCTTTTTTGTAAGTCTTCTCCCCCTCTTTTTATTCCTCAGCCA

*E. coioides* AACAGCAGTGAAACTAGCCTTTCTTGTCAGCCTTCTCCCCCTTTTCTTATTCCTTAGCCA

*E. bruneus* AACAGCAGTAAAACTAGCCTTCTTTGTTAGCCTCCTCCCCCTTTTCCTATTTCTCAGCCA

*E. moara* AACAGCAGTAAAACTAGCCTTCTTTGTTAGCCTCCTCCCTCTCTTCCTATTTCTCTGCCA

*E. lanceolatus*  AACAGCAGTAAAGCTAGCCTTCTTTGTTAGCCTTCTCCCCCTTTTCTTATTCCTCAGCCA

*A. leucogrammicus*  AACAGCAGTAAAACTAGCCTTTTTCGTCAGCCTTCTTCCTCTTTTCTTATTTCTTAGTCA

*C. altivelis* AACAGCAGTAATACTAGCCTTTTTTGTTAGCCTTCTTCCCCTTTTCTTATTCCTTAGCCA

*E. epistictus* AACAGCAGTGAAACTAGCCTTTTTCGTCAGCCTTCTACCCCTTTTCCTGTTTTTAGCTGA

*E. octofasciatus* AACAGCAGTAAAACTAGCCTTTTTTGTTAGCCTTCTCCCCCTCTTCTTGTTCCTTAACGA

*E. septemfasciatus* AACAGCAGTAAAACTAGCCTTCTTTGTCAGCCTTCTCCCCCTCTTCTTGTTCCTTAACGA

*T. dermopterus* GACAGCAGTAAAACTTGCTTTCTTTGTTAGCCTTCTTCCCCTCTTTTTATTCCTCAACGA

*E. awoara* GACGGCGGTGAAACTAGCCTTTTTTGTTAGTCTCCTCCCCCTTTTCTTATTCCTTAGTGA

*E. akaara* GACGGCGGTAAAACTAGCCTTTTTTGTTAGTCTCCTCCCCCTTTTCTTATTCCTTAGTGA

*E. trimaculatus* AACAGCTGTTAAACTAGCTTTTTTTGTCAGCCTCCTTCCCCTTTTCTTATTCCTCACTGA

*E. areolatus* GACAGCTGTAAAACTAGCCTTTTTTGTCAGTCTCCTCCCTCTCTTTTTATTTCTCACTGA

*V. albimarginata* AACAGCAGTTAAACTAGCTTTCTTTGTGAGCCTCTTACCCCTCTTTTTATTCCTTAATGA

*V. louti* AACAGCAGTTAAACTAGCCTTCTTTGTAAGCCTTTTACCCCTCTTTCTATTCCTCAATGA

*P. leopardus* AACAGCAGTTAAGTCGGCTTTTTTTGTTAGCTTGTTACCTCTATTTCTATTTCTTAATGA

*P. areolatus* AACTGCAGTTAAATTTGCTTTTTTTATCAGCCTATTACCCCTTTTTCTATTTCTTAACGA

*E. radiosum* AGGTGCAGTTAAGCTAGCTTTTCTGGTGAGTCTCCTTCCCCTCTTTCTATTCATAAACGA

*P. sieboldi* ACTCGCCGTCAAGATGGCCTTCCTGGTCAGCCTCGCCCCTCTCTTCCTCTTTCTTAATGA

*E. armatus* AACAGCGGTTAAGCTGGCTTTCCTTGTAAGCCTTATTCCCCTGTTCCTTTTCCTTAACGA

*R. oxyrhynchus* AACAGCCGTCAAACTAGCATTCTTTGTTAGCCTTCTCCCACTATGCCTATTCCTCAATGA

*K. cinerascens* AACAGCTGTTAAACTCGCTTTCTTCGTCAGCCTCCTCCCACTATTCCTTTTCCTCAACGA

*T. chatareus* AACAGCAGTTAAACTAGCCTTCTTCATCAGCCTCCTCCCTCTGTTTTTATTCTTAAATGA

*D. berycoides* GACAGCAGTAAAACTGGCCTTCTTCGTCAGCCTCCTTCCTCTGTTTCTATTTATTAGTGA

*H. typus* AACAGCAGTGAAACTAGCTTTCTTTGTCAGTCTCCTTCCCTTATTTCTATTCCTCAACGA

*M. argenteus* AACAGCAGTAAAACTAGCTTTCTTCATTAGCCTCCTTCCCTTATTCCTATTTCTCAATGA

*S. chuatsi* AACAGCAGTTAAACTAGCTTTCTTCACTAGCCTCCTTCCTTTGGGCCTTTTCCTCAACGA

*O. fasciatus* GACCGCAGTAAAGCTAGCTTTCTTTGTTAGCCTCCTTCCACTATTCCTATTTCTAAACGA

*P. trilineatum* AACAGCAGTAAAGCTCGCCTTCTTTGTTAGCCTTCTTCCCCTATTCTTATTCCTCAATGA

*M. salmoides* AACAGCAGTAAAGCTGGCCTTCTTTACAAGCCTCCTTCCTCTTGCCCTATTCCTTAACGA

*P. tile* AACAGCCGTAAAATTAGCCTTCTTTGTTAGTCTCCTCCCCCTCTTCCTCTTCCTCAACGA

*L. argentimaculatus* AACAGCAGTGAAGCTAGCTTTCTTTGTCAGCCTCCTTCCTCTGTTCCTCTTCCTCAACGA

*E. struhsakeri* AACAGCAGTCAAACTAGCCTTCTTTGTTAGCCTCCTCCCGCTGTTTCTGTTCCTCAATGA

*B. albus* AACAGCAGTCAAAACAGCATTCTTTGTCAGCCTCCTCCCCCTCTTCCTTTTCCTCAACGA

*C. auripes* AACAGCAGTAAAATTGGCCTTCTTCATTAGCCTCCTCCCCATATTCCTTTTCTTTAATGA

*C. melampygus* AACAGCAGTAAAACTTGCTTTCTTTGTTAGCCTCCTCCCCCTATTCATTTACCTCAACCA

*L. calcarifer* GACAGCAGTAAAACTAGCCTTCTTCATCAGCTTACTTCCCCTATTCCTATTCCTAAACGA

*S. maena* AACAGCAGTAAAGCTGGCCTTTTTTGTAAGCCTATTGCCACTGTTTTTTTTACTCAACGA

*P. auriga* AACAGCAGTTAAACTAGCCTTCTTCATCAGCCTCCTTCCCCTATTTTTATTCCTAAATGA

*C. lucidus* AACAGCGGTAAAACTCACTTTCTTTGTAAGCCTCCTTCCACTCTTCCTATTCCTTAATGA

*S. sihama* AACAGCAGTAAAACTGGCCTTTTTCGTTAGCCTCCTCCCTTTATTTCTGTTCTTTAATGA

*C. loricula* AGGGGCCGTCAAAATGGCCTTCTTCGTCAGCCTACTCCCACTTTGTCTTTTCCTAAACGA

*A. trutta* AACCGCAGTCAAAGCAGCATTTTACATTAGCCTCCTCCCCCTGACTATCTACATCTCCGA

*H. gemma* AACAGCTGTAAAATATGCTTTCTTCGTAAGCCTACTTCCCCTATTCATCTTCCTTAATGA

*P. flavescens* AACTGCAGTCAAACTGGCTTTCTTAGTCAGCCTTCTTCCGTTATTTTTATTCCTTAATGA

*P. macrolepida* GACTGCAGTTAAACTAGCTTTTTTAGTCAGCCTTCTCCCATTATCCCTATTTCTCAACGA

*S. canadensis* AACTGCAGTAAAACTGGCCTTTCTCGTCAGCCTTCTTCCTCTCTTCTTATTTCTTAACGA

*A. rogaa* GGGGGCGGAAGCAGTGATTACTTCATGAAGCTGAATAAACACTACTACTTTTGATATTAA

*C. argus* AGGAGCGGAAACAATCGTCACTTCGTGAAACTGAATAAACACTACTGCCTTCGATATCAA

*C. sonnerati* AGGAGCAGAAACAATCATTACATCATGAAACTGAATAAACACCACAACCTTCGATGTTAA

*E. fuscoguttatus* AGGAGCAGAAACCGTTATTACCTCATGAAACTGAATAAGTACATCAACCTTTGACATCAA

*E. coioides* AGGGGCAGAAACTGTTATTACCTCTTGAAACTGGATATCTACATCAACTTTTGACATCAA

*E. bruneus* AGGGGCAGAAACCGTTATTACCTCATGAAACTGAATAAGTACAGCAACTTTTGACATTAC

*E. moara* AGGGGCAGAAACCGTTATCACCTCATGAAACTGAATAAGTACATCAACCTTTGACATTAC

*E. lanceolatus*  AGGGACAGAGACCGTTATTACCTCATGAAATTGGATAAATACATCAACTTTTGACATCAA

*A. leucogrammicus*  GGGGGCAGAGACCGTTATTACCTCATGAAACTGAATAAACACATCAACTTTTGACATCAA

*C. altivelis* AGGAGCGGAAACCATTATTACCTCATGAAACTGAATAAATACATCAACTTTTGACATTAA

*E. epistictus* AGGAGCAGAAACCATTACCACTTCATGAACCTGGATAAACACAGCAACCTTTGACGTAAG

*E. octofasciatus* AGGGGCAGAAGCCGTTATTACCTCATGAAACTGAATAAACACCTCAACTTTCGACGTAAA

*E. septemfasciatus* AGGGACAGAAGCCATTGTTACCTCATGAAACTGAATAAATACCTCAACTTTCGACGTGAA

*T. dermopterus* AGGGGCAGAAACCATTACTACCTCGTGAAATTGAATAAACACCTCGACTTTCGACGTAAA

*E. awoara* GGGAGCAGAAACCATTGCTTCCTCATCAAGCTGAATAAATACACTAACCTTTGACATTAA

*E. akaara* GGGAGCAGAAACCATTGCTTCCTCATCAAGCTGAATAAATACACTAACTTTTGACATTAA

*E. trimaculatus* GGGGGTAGAAACCATTGCTTCCTCATCAAGCTGAATAAACACATCAACCTTTGACGTAAG

*E. areolatus* AGGGGTAGAAACCATTGCTTCCTCGTCAAACTGAATAAACACGTCAACCTTTGACGTTAA

*V. albimarginata* AGGGGCAGAAACAATTATTACCTCATGAAACTGAATGAATACCCAAACCTTCGATATCAA

*V. louti* AGGGGCAGAAACAATTATTACCTCATGAAACTGAATAAACACCCAAACCTTTGATATCAA

*P. leopardus* AGGTGCAGAAACCATTATCACTTCCTGAAGCTGAATAAATACTATAACATTTGACGTAAA

*P. areolatus* AGGCGCAGAAGCCATCATCACTTCCTGAAACTGAATAAATACCACAGCATTCGACGTAAA

*E. radiosum* CGGAGCCGAGACCATTGTGACCACCTGAAGTTGGGTTAACACCCTCGTTTTTGACATCAA

*P. sieboldi* GGGCACGGAGATGATCATCACCTCTTGGAGCTGAATAAACACACTAACCTTTGACGTTAA

*E. armatus* AGGCGCAGAAACAATTGTTACAAACTGAAACTGAATAAATACCGTTACTTTCGACATCAA

*R. oxyrhynchus* AGGCGCAGAGACAATCGTTACCAGCTGAAGCTGAATAAACACCGGCACCTTTGACATTAA

*K. cinerascens* AGGGGCAGAAACAATTATCACCAACTGAAACTGAATAAACACCCTAACCTTTGACGTAAA

*T. chatareus* AGGACTAGAAACAATCATCACCAACTGAAACTGAATAAACACTATCACCTTTGACATCAA

*D. berycoides* AGGAGCAGAAGTAATTATTACCAACTGAGGCTGAATGAACATTATAGCCTTTGATGTTAA

*H. typus* AGGTGCAGAAATAATTATCACCAACTGAAACTGAATAAACACCACAACCTTCGACGTAAA

*M. argenteus* AGGGGCAGAGACAATTATCACCAACTGAAACTGAATAAATACCAACACCTTCGATGTAAA

*S. chuatsi* AGGAGCAGAAGTAATCATTACTAACTGAAATTGAATAAATACCCTAACATTTGATGTTAA

*O. fasciatus* AGGAGCAGAAATAATTATTACCAGCTGAAACTGAATAAACACCCTAACCTTTGACGTGAA

*P. trilineatum* AGGGGCTGAAGCCATCATCACAAACTGAAATTGAATAAACACTACGACCTTTGACGTCAA

*M. salmoides* AGGTGCAGAAACTGTTATTACTAACTGGTCCTGGATAAACACCCTAACATTTGACATTAA

*P. tile* AGGCGCAGAGACGATCATCACCAACTGAAACTGAATAAACACCCTAACCTTCGACGTAAA

*L. argentimaculatus* AGGCGCGGAGACAATCATCACCAACTGGAACTGAATAAACACCTTAACCTTTGATGTAAA

*E. struhsakeri* AGGCGCAGAAACAATTATCACCAACTGAAACTGAATAAATACAATAACCTTCGATGTAAA

*B. albus* AGGCGCAGAAACAATTATCACCAACTGAAACTGGATTAACACCCAAACCTTCGATATCAA

*C. auripes* AGGTGCAGAGGCTATCATCACCAACTGAAACTGAATAAACACCCTAACATTTGATATTAA

*C. melampygus* AGGACTAGAAACCATCATCACTAACTGAAATTGAATAAATACCCTCACCTTCGACATCAA

*L. calcarifer* AGGCCTAGAAGTAATTATCACCAACTGAAACTGAATAAATATTGCCACCTTTGACATAAA

*S. maena* AGGCGCAGAAACCATTGTAACCAACTGAAACTGAACAAACACGCTGACTTTTGACATCAA

*P. auriga* AGGAGCAGAAGCCATCATCACCAATTGAAACTGAATAAATACCCTAACTTTTGACATTAA

*C. lucidus* AGGGGCAGAAATAATTGTCACCAATTGAACCTGAATAAATACCCTAACCTTTGATATTAA

*S. sihama* AGGAGCAGAGACAATTATTACTACTTGAACCTGAATAAACACCCTAACCTTCAGCATCAA

*C. loricula* GGGGGCAGAAACGATTATCACCAACTGAAGCTGAATAAACACCTTAACCTTCGACATTAA

*A. trutta* AGGGACAGAAACTGTCACAACCGGCTACAGCTGAGCAAATATCGACTCTTTCAGCATTAA

*H. gemma* AGGCGCAGAAGAAATTATTACTTCCTGAAACTGAATAAATACCCTAACCTTCGACGTCAA

*P. flavescens* GGGGGCAGAAACAATCGTAACTTCCTGAACCTGAATAAATACCCAAATCTTTGACATTAA

*P. macrolepida* GGGCGCAGAGACAATTATTACGTCCTGAAACTGAATAAACACCCTGGCCTTCGATATTAA

*S. canadensis* AGGAGCAGAGACGATTATTACCTCCTGGAACTGGATAAATACCTCAACCTTTGACGTAAA

*A. rogaa* CATTAGCTTTAAATTTGACCACTATTCCATTATCTTTACACCTATCGCCCTGTACGTTAC

*C. argus* CATCAGCCTAAAATTTGACCATTACTCCATTATCTTTACACCCATTGCCCTATATGTCAC

*C. sonnerati* TATTAGCTTTAAATTCGACCACTACTCCGTTATTTTCACACCAATTGCTCTATACGTAAC

*E. fuscoguttatus* TATCAGCCTGAAATTCGACCACTACTCTATTATATTTACACCCGTCGCCCTATATGTCAC

*E. coioides* CATTAGCCTGAAATTCGACCACTACTCTATTATATTTACACCCGTCGCCCTATATGTCAC

*E. bruneus* CATCAGCTTGAAATTTGACCACTACTCCGTCATATTTACACCCGTTGCCCTATATGTCAC

*E. moara* CATCAGCTTGAAATTTGACCACTACTCCATCATATTCACACCCGTTGCCCTATATGTCAC

*E. lanceolatus*  CATTAGCTTGAAATTCGACCACTACTCTATTATATTCACACCTGTCGCCCTGTATGTCAC

*A. leucogrammicus*  TATCAGCTTGAAGTTCGACTTTTACTCTATCATTTTCACACCTGTTGCCCTTTATGTTAC

*C. altivelis* TATTAGCCTAAAATTTGACCACTACTCCATTATATTTACGCCCGTCGCCTTATATGTCAC

*E. epistictus* TATTAGTCTAAAATTTGACTTCTACTCCATTATTTTTACACCTATTGCCCTCTACGTCAC

*E. octofasciatus* CATCAGCTTAAAATTCGACTACTACTCCATTGTTTTTACACCCATCGCCCTATATGTTAC

*E. septemfasciatus* TATCAGCCTAAAATTCGACTACTACTCCATTGTTTTTACACCCATCGCCCTATATGTTAC

*T. dermopterus* TATTAGCTTGAAATTCGACTACTACTCCATTGTTTTTACACCCATCGCCCTGTATGTCAC

*E. awoara* CCTCAGCTTAAAATTTGACCACTACTCTGTCATCTTCACACCTGTTGCTCTATATGTTAC

*E. akaara* CCTCAGCTTAAAATTTGACCACTACTCCGTCATCTTTACACCTGTTGCTTTATATGTCAC

*E. trimaculatus* CCTCAGCTTAAAATTCGATCACTATTCCATCATCTTTACACCTGTTGCCCTATACGTCAC

*E. areolatus* CCTTAGTTTAAAATTTGACCACTATTCCATTATTTTTACACCTGTGGCCTTATATGTTAC

*V. albimarginata* TATTAGCCTTAAATTTGATCACTACTCCATTATTTTCACCCCAATTGCCCTATATGTAAC

*V. louti* TATTAGCCTTAAATTTGATCACTACTCCATCATCTTCACCCCTATTGCCCTATACGTAAC

*P. leopardus* TATTAGCTTCAAATTTGACCACTACTCCATCATCTTCACCCCTATTGCTCTTTATGTAAC

*P. areolatus* TGTAAGCTTCAAGTTCGACTTCTACTCCATCATCTTTACCCCTATCGCCTTATATGTGAC

*E. radiosum* TATTAGCTTCAAGTTTGATCACTATTCGATTATTTTCACCCCAATTGCCCTTTATGTAAC

*P. sieboldi* CCTGAGTATCAAATTTGACCACTACTCAATTATCTTTACACCTATTGCTCTTTATGTCAC

*E. armatus* TATTAGCTTTAAGTTCGACCACTATTCTATTATTTTTACACCTATCGCTCTGTATGTGAC

*R. oxyrhynchus* CATCAGCCTAAAATTCGACCACTACTCAATTATCTTCACCCCTATCGCCCTATATGTAAC

*K. cinerascens* CATTAGCTTCAAATTTGACCACTACTCAATCATTTTTACCCCCATTGCCTTATACGTAAC

*T. chatareus* TATTAGCCTCAAATTTGACCACTACTCAATCATTTTCGTCCCCATTGCCCTCTACGTAAC

*D. berycoides* TGTTAGCTTTAAGTTCGACCACTATTCAATTATTTTTACCCCCATTGCCCTCTATGTCAC

*H. typus* CATCAGCTTTAAATTTGACCACTATTCAATCATCTTTACCCCCATTGCCCTCTACGTAAC

*M. argenteus* CATCAGCCTTAAATTCGACCATTATTCCATTATCTTCACCCCCGTCGCCCTCTACGTAAC

*S. chuatsi* TATTAGCCTCAAATTCGACCACTACTCAATTATCTTTACCCCAGTCGCCCTATATGTTAC

*O. fasciatus* TATCAGCTTCAAATTCGACCACTACTCTATTATTTTCACCCCTATTGCCCTCTACGTGAC

*P. trilineatum* TATCAGCTTTAAATTTGACCACTACTCCATTATCTTCACCCCCATTGCACTATACGTTAC

*M. salmoides* TATCAGCCTAAAGTTTGATCTTTACTCCGTCATCTTTACCCCCGTTGCCCTATACGTTAC

*P. tile* TATTAGCTTCAAATTTGACCACTACTCGATTATCTTCACCCCTATCGCACTATACGTTAC

*L. argentimaculatus* CATCAGCTTTAAATTTGACCACTACTCAATCATCTTCACCCCAATCGCTCTATACGTTAC

*E. struhsakeri* TATCAGCTTCAAATTTGACCACTATTCAATTATCTTCACCCCAATTGCCCTATATGTTAC

*B. albus* TATTAGCCTTAAATTCGACCACTACTCAATTATCTTCACCCCCGTCGCTCTTTACGTCAC

*C. auripes* TATTAGCTTTAAATTCGACCATTACGCCATCATTTTTACCCCCGTCGCTCTTTATGTGAC

*C. melampygus* CATTAGCCTAAAATTTGACCACTACTCCATTATCTTTACCCCCATTGCCCTCTACGTAAC

*L. calcarifer* CATCAGCCTTAAATTTGACCACTACTCAATTATCTTTACCCCAGTCGCACTATACGTGAC

*S. maena* CATTAGCTTCAAGTTTGACCACTACTCGACCATCTTTACCCCTATCGCTCTTTATGTGAC

*P. auriga* TATCAGCTTTAAATTTGACCACTATTCAATTATCTTTACCCCAATTGCCCTGTACGTAAC

*C. lucidus* CATTAGCTTTAAATTCGACCACTACTCAATTATTTTTACCCCCATTGCCCTCTATGTCAC

*S. sihama* CATTTCCTTCAAATTTGACTTCTACTCAATTGTCTTCACCCCTGTCGCACTATACGTTAC

*C. loricula* CATTAGCTTTAAATTTGATTATTACTCCGTTACATTTACCCCTATTGCCCTCTACGTCAC

*A. trutta* CTTCAGCCTAATGTTCGACCAATATTCCGTGTTCTTCCTGCCTGTCGCACTCTACGTATC

*H. gemma* TATTAGCTTCAAGTTTGACCATTATTCCATTATCTTTACGCCTGTTGCCCTTTACGTAAC

*P. flavescens* CATTAGCTTCAAATTTGATTTTTACTCGATTATCTTCACACCAATCGCACTCTATGTAAC

*P. macrolepida* TATTAGCCTTAAGTTTGACCATTACTCCCTCATCTTCACCCCTATCGCCCTCTATGTGAC

*S. canadensis* CATCAGCTTCAAATTTGACCATTATTCAGTTATTTTCACCCCTATTGCCCTATATGTAAC

*A. rogaa* ATGGTCCATTCTAGAATTTGCATCCTGATATATACACTCTGATCCTTACATAAATCGATT

*C. argus* ATGATCAATTCTAGAGTTTGCATCCTGATACATACACACCGACCCCCAAATAAACCGATT

*C. sonnerati* ATGGTCAATTCTAGAATTTGCATCCTGATATATACACGCCGACCCCTACATAAACCGGTT

*E. fuscoguttatus* ATGATCCATTCTCGAATTCGCATCCTGATATATACATGCCGACCCCAACATAAACCGATT

*E. coioides* ATGATCAATCCTAGAATTCGCATCCTGATATATACACGCCGATCCTAACATAAATCGATT

*E. bruneus* ATGATCCATCCTAGAATTTGCATCCTGATACATACATGCCGACCCCAACATAAACCGATT

*E. moara* ATGATCAATCCTAGAATTTGCATCCTGATACATACATGCCGACCCTAACATAAACCGATT

*E. lanceolatus*  ATGATCCATCCTAGAATTTGCATCCTGATATATACATGCTGATCCTAACATAAACCGATT

*A. leucogrammicus*  ATGATCAATCCTAGAATTCGCATCTTGGTACATGCATGCTGACCCTAACATAAACCGGTT

*C. altivelis* ATGATCAATCCTAGAATTTGCATCCTGGTATATACACGCTGACCCTAACATAAACCGATT

*E. epistictus* ATGATCAATTCTAGAATTCGCATCCTGATACATACATGCCGACCCCAATATAAACCGATT

*E. octofasciatus* ATGATCAATTCTAGAATTTGCATCCTGATATATACATGCTGACCCCAACATAAACCGATT

*E. septemfasciatus* ATGATCAATTCTAGAATTTGCATCCTGATATATACATGCTGACCCCAATATAAACCGATT

*T. dermopterus* ATGGTCAATTCTAGAGTTTGCATCCTGATATATACATGCCGACCCCAATATAAACCGATT

*E. awoara* ATGATCCATCCTAGAGTTTGCCTCCTGATATATACATGCTGATCCTAATATAAATCGGTT

*E. akaara* ATGGTCAATCCTAGAGTTTGCCTCCTGATATATACACGCTGACCCTAATATAAACCGGCT

*E. trimaculatus* ATGGTCAATCCTAGAATTTGCCTCATGATATATACATGCCGACCCTAATATAAATCGGTT

*E. areolatus* ATGGTCAATTCTAGAATTTGCCTCCTGGTATATACATGCCGACCCTAACATAAATCGGTT

*V. albimarginata* CTGGTCTATCCTAGAATTCGCATCCTGATACATACACTCTGACCCCTACATAAATCGCTT

*V. louti* CTGGTCCATCCTAGAGTTTGCATCTTGATACATACACTCTGACCCCTACATAAATCGCTT

*P. leopardus* TTGATCAATTCTAGAATTCGCATCTTGGTATATACACTCGGACCCTTTCATAGATCGATT

*P. areolatus* TTGATCTATTCTAGAATTCGCATCTTGGTACATACACTCAGACCCCTACATAAATCGATT

*E. radiosum* TTGATCTATCTTAGAGTTTGCATCCTGATATATGCATGCCGACCCTTACATGAACCGCTT

*P. sieboldi* TTGATCCATCCTAGAGTTCGCCACCTGGTATATGCACTCAGACCCTTACATTTTCCGATT

*E. armatus* CTGATCCATTCTTGAATTTGCCTCATGATATATGCATGCCGACCCATTTATGAATCGTTT

*R. oxyrhynchus* CTGATCTATTCTAGAATTCGCATCCTGATACATACACTCAGACCCCTACATAAATCGATT

*K. cinerascens* CTGATCTATCCTAGAATTCGCATCTTGATACATGCACGCAGACCCATTCATAAACCGATT

*T. chatareus* CTGATCAATTCTGGAATTTGCATCTTGGTACATACACGCAGACCCCTATATAAACCGATT

*D. berycoides* CTGATCCATCCTCGAATTCGCATCTTGATATATACATGCAGACCCGTACATAAACCGTTT

*H. typus* CTGATCAATCCTAGAATTCGCATCCTGATATATACACGCAGACCCCTACATAAACCGATT

*M. argenteus* CTGATCCATTCTCGAATTTGCATCCTGATATATACATGCAGACCCTTTCATAAATCGCTT

*S. chuatsi* ATGATCAATTCTTGAATTTGCATCTTGATACATACATGCAGACCCTTACATGAACCGATT

*O. fasciatus* TTGATCCATTCTAGAATTTGCATCCTGATACATGCATGCAGACCCTTACATAAACCGCTT

*P. trilineatum* TTGATCTATCCTGGAGTTCGCATCTTGATATATGCACGCCGACCCCTTTATGAACCGATT

*M. salmoides* TTGATCTATTCTTGAATTTGCATCTTGATATATGCACACCGACCCATACATAAACCGTTT

*P. tile* ATGATCTATTCTCGAATTCGCATCCTGATACATACATGCAGACCCCTTCATAAACCGCTT

*L. argentimaculatus* TTGGTCGATCCTAGAGTTCGCCTCCTGATACATGCACGCGGACCCCTTCATAAACCGTTT

*E. struhsakeri* CTGATCAATCCTTGAGTTCGCATCATGGTACATGCACGCAGACCCCTTCATAAACCGATT

*B. albus* TTGATCCATTCTAGAGTTCGCATCTTGATATATACACTCAGACCCTTACATAAACCGTTT

*C. auripes* CTGATCCATCCTTGAATTCGCAGCATGATACATGCACGCTGACCCACAGATAGACCGTTT

*C. melampygus* ATGATCCATTCTAGAATTTGCATCTTGATACATACACTCAGACCCGTACATAAACCGATT

*L. calcarifer* CTGGTCTATCCTGGAATTTGAATCTTGATATATACACGCTGACCCATACATAAACCGATT

*S. maena* ATGATCCATTCTAGAATTTGCATCCTGATATATGCATGCTGACCCAAACATAAACCGATT

*P. auriga* CTGATCAATCTTAGAATTTGCATCCTGATATATACACGCTGACCCATACATAAACCGATT

*C. lucidus* CTGATCCATTCTAGAATTTGCATCTTGATATATACACTCAGACCCCTACATAAACCGATT

*S. sihama* ATGATCTATTCTCGAATTCGCATCCTGATACATACATGCAGACCCCTTCATAAACCGCTT

*C. loricula* CTGAGCCATTCTAGAGTTTGCATCATGATACATGCACTCCGACCCTTTCATGAACCGATT

*A. trutta* CTGAGCAATCCTTGAATTCGCACTATGATACATGCACTCAGACCCCCAAGCGGACCGATT

*H. gemma* CTGATCTATCCTAGAGTTTGCATCCTGGTATATGCATGCAGACCCAAACATAAACCGCTT

*P. flavescens* CTGATCCATTTTAGAATTTGCATCTTGATATATGCATGCAGACCCTTTTATGAACCGCTT

*P. macrolepida* ATGATCCATTCTAGAGTTTGCCTCGTGATACATGCACGCCGACCCTTTCATAAACCGATT

*S. canadensis* TTGGTCCATTTTAGAGTTCGCATCTTGGTATATACATGCCGACCCCTTCATAAACCGGTT

*A. rogaa* CTTCAAATACCTACTAGTCTTCTTAATCGCAATAATTATCCTAGTTACAGCAAATAATCT

*C. argus* CTTTAAGTATTTATTAGTATTTTTAATCGCAATAATTATCCTAGTAACAGCAAACAATTT

*C. sonnerati* CTTCAAATATCTTTTAGTCTTCCTTATCGCAATAATTATTCTAGTAACAGCAAACAACTT

*E. fuscoguttatus* CTTTAAGTACCTTTTAATTTTCTTAATCGCAATGATTATTTTAGTTACAGCGAATAACCT

*E. coioides* CTTTAAATACCTCTTAATTTTCCTAATCGCAATAATTATTCTAGTCACAGCAAACAACCT

*E. bruneus* CTTTAAGTATCTCCTAATTTTCCTAATCGCAATAATTATTTTAGTCACAGCAAACAACCT

*E. moara* CTTTAAGTATCTCCTAATTTTCCTAATCGCAATAATTATTTTAGTCACGGCAAACAACCT

*E. lanceolatus*  CTTTAAGTACCTACTAATCTTCCTAATTGCAATAATTATCCTAGTTACAGCAAACAACCT

*A. leucogrammicus*  CTTTAAATACCTCCTAACCTTTCTCATCGCAATAATTATCCTAGTTACTGCAAACAACCT

*C. altivelis* CTTTAAATACCTTCTAATCTTCCTAATTGCAATAATTACCCTAGTCACAGCAAATAACCT

*E. epistictus* CTTTAAATATCTCCTAATCTTCCTAATCGCAATAATTATCCTAGTTACAGCAAACAACCT

*E. octofasciatus* TTTTAAATACCTCCTAATTTTCCTAATCGCAATAATTATTTTAGTTACAGCAAACAACCT

*E. septemfasciatus* TTTTAAATACCTCCTAATTTTCCTAATCGCAATAATTATTTTAGTTACAGCAAACAACCT

*T. dermopterus* CTTTAAATACCTCCTAATCTTCCTAATCGCAATAATCATCCTAGTCACAGCAAACAACCT

*E. awoara* CTTTAAATATCTTTTAATTTTCCTAATTGCAATAATTATCCTAGTTACTGCAAACAACCT

*E. akaara* CTTTAAATATCTTTTAATTTTCCTAATTGCAATAATTATCCTAGTTACAGCAAACAACCT

*E. trimaculatus* TTTTAAATATCTTTTAATTTTCCTAATTGCAATAATCATCCTAGTTACAGCAAACAACCT

*E. areolatus* CTTTAAGTATCTTCTAATCTTCTTAATCGCAATAATTATTCTAGTTACAGCAAACAACCT

*V. albimarginata* TTTCAAATACTTACTAATCTTCTTAATCGCAATAATTATTCTAGTTACAGCCAACAACCT

*V. louti* TTTCAAATATCTACTTATTTTCCTAATCGCAATGATTGTTCTAGTCACAGCTAACAATCT

*P. leopardus* CTTTAAGTACCTACTAATTTTTCTTATCGCTATAGTTATACTAGTCACAGCAAACAATAT

*P. areolatus* TTTTAAATATTTACTAATCTTCCTTATTGCCATAATCACACTAGTCACAGCAAACAATAT

*E. radiosum* CTTTAAATACCTTTTAATTTTTCTAATTGCTATAATTATTCTAGTCACAGCAAATAATCT

*P. sieboldi* CTTTAAATACCTTCTGATCTTCCTCGTCGCCATGATTATCCTTGTCACAGCTAACAACCT

*E. armatus* TTTCAAGTACCTCCTTGTCTTTCTTATTGCTATAATCGTACTTGTTACAGCAAATAACAT

*R. oxyrhynchus* CTTCAAATATCTCCTCATCTTCCTCATTGCAATAATCGTCCTAGTTACAGCTAATAACAT

*K. cinerascens* CTTCAAATATCTCCTAATTTTCCTCATTGCCATGGTAGTTCTAGTAACGGCAAACAATAT

*T. chatareus* CTTCAAATACCTCTTAATCTTCCTAATTGCCATAATTGTCCTAGTCACAGCAAACAACAT

*D. berycoides* TTTCAAATACCTCCTTATTTTCCTCATTGCCATAATCATTCTAGTAACAGCCAATAACAT

*H. typus* CTTCAAGTACCTCCTTGTCTTCCTTGTCGCCATGATTATTTTAGTGACAGCTAACAACAT

*M. argenteus* CTTCAAGTACCTCCTTATTTTCCTTATCGCCATAATTATCCTAGTAACAGCCAACAACAT

*S. chuatsi* TTTCAAATACCTCCTAGTCTTTCTAATTGCTATAATTACCCTAGTTACAGCAAACAATAT

*O. fasciatus* TTTTAAATACCTTCTTGTATTCCTCATTGCTATGATTATTCTAGTAACAGCAAACAACAT

*P. trilineatum* CTTCAAATATCTCCTTGTATTCCTCATTGCCATGATTATTCTTGTTACAGCAAACAACAT

*M. salmoides* CTTCAAATACCTGTTGATCTTTCTCATTGCCATGATTATTTTAGTGACAGCCAATAATAT

*P. tile* CTTCAAGTACCTTCTCGTTTTCCTAATCGCTATGATTATCCTTGTAACAGCCAATAACAT

*L. argentimaculatus* CTTCAAATACCTCCTTGTCTTCCTCATCGCCATAATCATCCTAGTCACAGCCAACAACAT

*E. struhsakeri* CTTTAAGTACCTCCTTGTCTTCCTTATCGCTATGATCATTCTAGTGACAGCCAACAACAT

*B. albus* CTTCAAGTACCTTCTCATCTTCCTTATCGCTATAATTATTCTAGTTACAGCTAACAACAT

*C. auripes* CTTCAAGTACCTTCTCACCTTCCTAATTGCCATAATTATCCTAGTAACAGCAAACAACAT

*C. melampygus* CTTTAAATACCTACTAATCTTCCTCATCGCTATAATTATTCTAGTTACCGCAAACAACAT

*L. calcarifer* CTTCAAATATCTACTAATTTTCCTCATTGCCATAATTATCCTAGTTACCGCAAACAACAT

*S. maena* CTTTAAATACCTCCTTATCTTTCTGATTGCAATGGTCATCTTGGTTACCGCAAACAACAT

*P. auriga* CTTCAAATATCTACTAATTTTCCTCATTGCCATGATTGTCCTAGTAACCGCAAACAATAT

*C. lucidus* TTTTAAATACCTTCTTATCTTCTTAATCGCCATAATCATCCTCGTCACAGCCAATAATAT

*S. sihama* CTTCAAGTACCTTCTCGTTTTCCTAATCGCTATGATTATCCTTGTAACAGCCAATAACAT

*C. loricula* TTTTAAATACCTTCTCATTTTCCTTATTGCCATAATTATCCTAGTTACTGCAAACAACCT

*A. trutta* TTTCAAATATCTTCTAGTCTTCCTCGTTGCAATACTTGTCCTAGTCACAGCAAACAACAT

*H. gemma* TTTTAAGTACCTTTTAACTTTCTTAATTGCTATAATCATTTTGGTTACTGCAAATAACCT

*P. flavescens* TTTCAAATACCTCCTTATCTTTCTAATTGCTATAATTGTCCTAGTTACAGCAAACAACCT

*P. macrolepida* TTTTAAATACCTTCTAATTTTCCTGATCGCTATAATTATTTTAGTTACAGCAAACAATCT

*S. canadensis* TTTCAAATATCTTCTTGTTTTTCTCATTGCTATAATTACGCTAGTTACGGCAAACAACCT

*A. rogaa* ATTCCAACTCTTTATTGGATGAGAGGGAGTTGGTATTATATCCTTCCTACTTATTGGATG

*C. argus* ATTCCAACTCTTTATCGGATGAGAAGGTGTTGGTATCATATCTTTCTTACTCATCGGATG

*C. sonnerati* ATTCCAACTCTTTATTGGCTGAGAGGGCGTAGGAATCATATCTTTCCTACTTATTGGATG

*E. fuscoguttatus* CTTCCAACTCTTCATCGGATGAGAAGGTGTAGGAATTATATCTTTCCTCCTCATCGGTTG

*E. coioides* ATTCCAACTCTTTATCGGATGAGAAGGCGTAGGAATCATATCCTTCCTTCTTATCGGCTG

*E. bruneus* ATTCCAACTCTTTATCGGATGAGAAGGCGTAGGAATTATATCCTTCCTTCTCATTGGCTG

*E. moara* GTTCCAACTCTTCATCGGATGAGAAGGCGTAGGAATTATGTCTTTCCTTCTCATTGGCTG

*E. lanceolatus*  ATTCCAACTCTTCATTGGGTGAGAAGGCGTAGGAATCATATCCTTCCTCCTTATCGGCTG

*A. leucogrammicus*  ATTCCAACTTTTCATTGGTTGAGAGGGCGTAGGAATTATATCCTTCCTCCTTATCGGTTG

*C. altivelis* ATTTCAACTCTTCATCGGATGAGAAGGAGTAGGGATTATGTCTTTCCTTCTCATTGGCTG

*E. epistictus* CTTTCAACTCTTCATTGGGTGAGAAGGTGTTGGAATCATATCATTCCTCTTAATTGGGTG

*E. octofasciatus* CTTCCAACTCTTTATCGGCTGAGAAGGAGTCGGAATCATATCCTTCCTCCTTATTGGTTG

*E. septemfasciatus* CTTCCAACTCTTTATCGGCTGAGAAGGGGTCGGAATTATGTCCTTTCTCCTCATTGGTTG

*T. dermopterus* CTTCCAGCTTTTTATTGGCTGAGAAGGGGTTGGAATCATGTCCTTCCTTCTTATCGGCTG

*E. awoara* CTTCCAACTCTTTATTGGCTGGGAAGGGGTTGGAATCATATCCTTCCTTCTAATTGGTTG

*E. akaara* CTTCCAGCTCTTTATTGGGTGAGAGGGGGTTGGAACCATATCTTTCCTCCTAATTGGTTG

*E. trimaculatus* CTTTCAACTCTTTATCGGGTGGGAAGGAGTCGGAATTATGTCCTTTCTCCTAATTGGCTG

*E. areolatus* CTTCCAGCTCTTTATTGGGTGAGAGGGGGTTGGAATTATATCTTTCCTCCTAATTGGCTG

*V. albimarginata* CTTCCAACTATTTATTGGGTGAGAAGGCGTCGGAATCATATCCTTCCTACTAATTGGCTG

*V. louti* CTTCCAACTTTTCATTGGCTGAGAAGGCGTTGGGATTATGTCCTTTCTACTAATTGGCTG

*P. leopardus* ATTTCAACTTTTTATTGGATGAGAAGGTGTAGGAATTATGTCTTTTCTCCTAATCGGTTG

*P. areolatus* ATTTCAACTTTTCATCGGATGGGAAGGTGTAGGAATTATGTCTTTTCTACTCATCGGCTG

*E. radiosum* CTTCCAACTTTTCATCGGGTGGGAAGGGGTCGGTATTATATCTTTCCTTCTCATCGGATG

*P. sieboldi* ATTCCAGTTGTTCATCGGATGGGAGGGAGTTGGCATCATGTCTTTCTTACTCATCGGGTG

*E. armatus* ATTTCAACTTTTTATTGGATGGGAAGGCGTAGGCATCATGTCTTTTCTTCTCATTGGCTG

*R. oxyrhynchus* ATTTCAACTTTTTATTGGCTGAGAAGGCGTCGGCATCATATCGTTCCTACTTATCGGCTG

*K. cinerascens* GTTCCAGCTCTTCATCGGCTGAGAAGGTGTCGGAATCATGTCATTCCTCCTCATCGGCTG

*T. chatareus* ATTTCAGCTCTTTATTGGCTGAGAAGGAGTAGGAATTATATCCTTCCTTCTTATCGGCTG

*D. berycoides* GTTTCAGCTCTTTATTGGATGAGAAGGCGTAGGCATCATATCCTTTCTCCTCATTGGCTG

*H. typus* ATTCCAACTGTTTATTGGCTGAGAAGGCGTGGGCATCATATCATTCCTCCTTATCGGTTG

*M. argenteus* GTTCCAACTCTTTATTGGCTGAGAAGGCGTCGGAATTATGTCCTTCCTACTCATTGGCTG

*S. chuatsi* ATTTCAACTTTTTATTGGCTGAGAAGGAGTAGGCATTATGTCCTTCCTTCTCATCGGCTG

*O. fasciatus* ATTTCAACTTTTTATTGGATGGGAAGGCGTAGGCATTATATCCTTCCTCCTAATTGGCTG

*P. trilineatum* ATTTCAAATTTTCATTGGCTGAGAGGGCGTAGGAATCATATCCTTCCTTCTCATCGGTTG

*M. salmoides* ATTTCAATTTTTTATCGGCTGAGAAGGTGTGGGTATCATGTCCTTTCTTCTTATCGGCTG

*P. tile* GTTCCAGATCTTTATCGGCTGAGAAGGCGTAGGCATCATATCCTTCCTCCTTATCGGCTG

*L. argentimaculatus* ATTCCAAATTTTCATCGGCTGAGAAGGAGTAGGTATCATGTCCTTCCTTTTAATCGGCTG

*E. struhsakeri* GTTCCAAATCTTTATTGGATGAGAAGGTGTAGGGATCATGTCATTCCTCCTCATCGGCTG

*B. albus* ATTCCAATTCTTCATCGGTTGAGAGGGTGTTGGCATCATATCATTTCTTTTAATCGGTTG

*C. auripes* ATTCCAACTGTTCATTGGGTGAGAAGGAGTAGGCATTATATCCTTCCTTCTCATTGGCTG

*C. melampygus* ATTCCAAATCTTCATTGGATGGGAAGGCGTCGGAATCATATCCTTCCTCCTAATCGGCTG

*L. calcarifer* ATTCCAACTCTTCATTGGCTGAGAAGGTGTGGGAATCATATCATTTCTCCTCATCGGCTG

*S. maena* ATTTCAACTCTTTATTGGCTGGGAAGGAGTTGGCATTATATCATTCCTCTTAATCGGCTG

*P. auriga* ATTCCAACTTTTTATTGGCTGAGAAGGGGTTGGAATTATATCCTTCCTCCTTATTGGCTG

*C. lucidus* GTTTCAACTATTCATCGGCTGAGAGGGCGTGGGGATCATATCCTTCCTCTTAATCGGCTG

*S. sihama* GTTCCAGATCTTTATCGGCTGAGAAGGCGTAGGCATCATATCCTTCCTCCTTATCGGCTG

*C. loricula* CTTCCAACTCTTTATTGGTTGAGAAGGCGTAGGAATTATATCCTTCCTCCTTATTGGCTG

*A. trutta* ATTCCAATTCTTCATCGGATGGGAAGGGGTTGGCATTATATCCTTCCTCCTTATCGGCTG

*H. gemma* TTTTCAACTTTTTATTGGCTGAGAGGGTGTGGGAATCATGTCTTTCCTTCTCATCGGCTG

*P. flavescens* TTTCCAACTCTTTATTGGGTGGGAAGGAGTAGGCATTATATCCTTCCTTCTTATCGGATG

*P. macrolepida* CTTTCAGCTCTTTATTGGGTGGGAGGGAGTCGGCATCATGTCCTTCCTTCTCATCGGGTG

*S. canadensis* ATTTCAAATTTTCATTGGTTGGGAAGGAGTGGGCATTATATCTTTCCTTCTTATCGGATG

*A. rogaa* ATGATACGGCCGAGCAGACGCAAACACTGCTGCTCTCCAAGCAGTTGTCTATAATCGAGT

*C. argus* ATGGTTTGGTCGAGCAGATGCGAATACCGCTGCTCTTCAAGCAGTTATTTATAACCGAGT

*C. sonnerati* ATGATATGGCCGAGCAGATGCAAACACCGCAGCCCTACAAGCAGTTGTTTATAACCGGGT

*E. fuscoguttatus* ATGACACGGCCGAGCAGACGCTAACACCGCAGCCCTACAAGCGGTCATTTACAATCGAGT

*E. coioides* ATGACACGGCCGAGCAGATGCCAACACCGCTGCCCTACAGGCAGTAATCTACAACCGAGT

*E. bruneus* ATGACACGGCCGAGCAGATGCTAACACCGCTGCCCTACAAGCAGTTATCTACAACCGAGT

*E. moara* ATGACACGGCCGAGCAGATGCTAACACCGCTGCCCTACAAGCAGTTATCTACAACCGAGT

*E. lanceolatus*  ATGACACGGCCGAACAGATGCTAATACCGCTGCCCTACAAGCAGTAATCTACAACCGAGT

*A. leucogrammicus*  ATGACATGGCCGAGCAGATGCTAACACTGCTGCTCTACAAGCAGTCATCTACAATCGAGT

*C. altivelis* ATGACATGGTCGAGCAGACGCTAACACCGCCGCCCTACAAGCAGTAATTTACAACCGAGT

*E. epistictus* ATGACATGGCCGAGCAGATGCAAATACCGCAGCCCTACAAGCAGTTATTTATAACCGAGT

*E. octofasciatus* ATGACATGGCCGAGCAGACGCAAATACCGCAGCCCTACAGGCAGTAATTTATAACCGAGT

*E. septemfasciatus* ATGACATGGCCGAGCAGACGCAAACACCGCAGCCCTACAGGCAGTAATTTACAACCGAGT

*T. dermopterus* ATGACACGGCCGAGCAGATGCAAACACCGCAGCCCTACAAGCAGTGATTTATAACCGGGT

*E. awoara* ATGACATGGCCGTGCAGATGCGAACACTGCAGCCCTACAGGCAGTAATTTATAACCGAGT

*E. akaara* ATGGCATGGCCGGGCGGACGCAAACACTGCAGCCCTACAAGCAGTTATTTACAACCGAGT

*E. trimaculatus* GTGGCACGGCCGGGCGGATGCAAATACCGCCGCCCTACAAGCAGTTATTTATAACCGGGT

*E. areolatus* ATGACACGGACGGGCAGACGCAAACACCGCAGCCCTACAAGCAGTTATCTACAACCGGGT

*V. albimarginata* ATGATACGGCCGGACGGACGCAAACACCGCCGCCCTCCAAGCAGTAATTTACAATCGGGT

*V. louti* ATGGTATGGCCGAGCAGACGCAAACACCGCTGCCCTCCAAGCAGTCATTTACAACCGAGT

*P. leopardus* ATGATACGGCCGGGCAGACGCTAACACCGCCGCTCTCCAAGCAGTCATTTATAACCGAGT

*P. areolatus* ATGATACGGCCGAGCAGACGCTAACACCGCCGCTCTCCAAGCAGTCATTTACAACCGAGT

*E. radiosum* GTGATACGGGCGGGCGGACGCAAACACTGCAGCCCTCCAAGCGGTGGTTTACAACCGAGT

*P. sieboldi* GTGATACGGCCGAGCGGACGCCAACACTGCTGCGATGCAAGCAGTCCTTTACAACCGTGT

*E. armatus* ATGATACGGACGGGCTGACGCCAACACCGCTGCCCTTCAAGCAGTTCTTTATAACCGCGT

*R. oxyrhynchus* ATGGTACGGACGAGCTGACGCAAATACTGCCGCCCTACAGGCGGTCGTATATAACCGTGT

*K. cinerascens* ATGATACGGCCGAGCAGATGCAAACACCGCCGCCCTCCAAGCAGTCATTTACAACCGAGT

*T. chatareus* ATGGTATGGTCGAGCAGATGCAAACACCGCAGCCCTGCAAGCAGTCCTCTACAACCGAGT

*D. berycoides* ATGACATGGCCGGGCAGACGCAAATACCGCTGCCCTCCAAGCAGTTATTTATAACCGAGT

*H. typus* ATGGTACGGACGGGCAGACGCTAATACCGCCGCCCTACAGGCAGTTCTTTACAACCGAGT

*M. argenteus* ATGATATGGCCGAGCAGATGCGAACACTGCCGCCCTCCAAGCAGTTCTCTACAACCGAGT

*S. chuatsi* ATGATATGGACGAGCCGACGCAAATACTGCTGCCCTCCAGGCAGTTCTTTATAACCGAGT

*O. fasciatus* ATGATATGGCCGAGCAGATGCAAATACCGCTGCTCTCCAGGCAGTAATTTATAACCGGGT

*P. trilineatum* ATGATATGGACGAGCAGACGCAAATACCGCTGCCCTCCAAGCAGTTCTCTACAACCGGGT

*M. salmoides* ATGATACGGCCGAGCAGACGCAAACACCGCCGCTCTTCAAGCAGTCCTCTATAACCGAGT

*P. tile* ATGATACGGCCGAGCAGACGCAAACACAGCTGCCCTACAAGCAGTCCTTTACAACCGAGT

*L. argentimaculatus* ATGGTATGGCCGAGCCGATGCAAACACAGCCGCCCTCCAGGCAGTCCTTTATAACCGAGT

*E. struhsakeri* ATGATACGGACGAGCGGACGCGAACACCGCCGCCCTGCAAGCAGTCCTATACAATCGGGT

*B. albus* ATGATACGGCCGAACAGACGCGAACACCGCCGCACTACAAGCAGTCCTTTACAACCGAGT

*C. auripes* ATGGTACGGACGAGCAGACGCAAACACCGCCGCCCTCCAGGCAGTCCTATACAACCGAGT

*C. melampygus* ATGATACGGCCGAGCAGACGCCAACACCGCAGCCCTACAAGCAGTGCTTTACAACCGAGT

*L. calcarifer* ATGGCACGGCCGAGCCGACGCAAACACCGCAGCTTTACAAGCAGTTCTCTACAATCGCCT

*S. maena* ATGATACGGCCGAGCAGACGCAAATACCGCCGCACTTCAAGCGGTACTTTATAATCGTGT

*P. auriga* ATGGTATGGACGAGCAGATGCCAACACCGCTGCCCTTCAAGCAGTTCTCTACAATCGTGT

*C. lucidus* ATGGTATGGCCGAGCAGACGCAAACACTGCAGCCCTCCAAGCTGTTCTCTATAACCGAGT

*S. sihama* ATGATACGGCCGAGCAGACGCAAACACAGCTGCCCTACAAGCAGTCCTTTACAACCGAGT

*C. loricula* ATGATACGGCCGAGCAGACGCAAACACCGCCGCTCTCCAAGCCGTCCTTTACAACCGGGT

*A. trutta* ATGATACGCACGATCAGACGCCAATACAGCCGCCCTACAAGCTGTCCTGTACAACCGGGT

*H. gemma* ATGATACGGACGAGCTGACGCAAATACCGCTGCCTTACAAGCAGTCGTCTATAATCGTGT

*P. flavescens* ATGATATGGGCGAGCAGATGCAAACACCGCTGCCCTACAAGCAGTTGTATACAACCGAGT

*P. macrolepida* ATGGTACGGACGGGCAGATGCAAACACTGCAGCCCTTCAAGCAGTTGTATACAACCGAGT

*S. canadensis* GTGGTACGGACGGGCAGATGCAAACACCGCAGCCCTTCAAGCGGTTGTATATAATCGCGT

*A. rogaa* AGGTGATATTGGACTAATTTTTTCCATGGCATGAATAGCAACCAATCTTAACTCCTGAGA

*C. argus* AGGTGACATCGGCCTAATCCTTTCTATAGCGTGGATAGCAACCAACCTCAACTCTTGAGA

*C. sonnerati* AGGAGACGTTGGACTAATCTTTGCAATAGCATGAATAGCAACCAACCTTAACTCTTGAGA

*E. fuscoguttatus* CGGGGATATTGGTTTAATCCTTGCAATAGCATGAATAGTTTCTCATCTTAACTCATGAGA

*E. coioides* CGGGGATATTGGTCTAATCTTTGCAATAGCATGAATGGTGTCTCACCTTAACTCATGAGA

*E. bruneus* GGGGGATATTGGTTTAATCTTCGCAATAGCATGAATAGTATCTCACCTTAATTCATGAGA

*E. moara* AGGGGACATTGGTTTAATCTTTGCAATAGCATGAATAGTGTCCCACCTTAACTCATGAGA

*E. lanceolatus*  AGGAGACATTGGATTAATCCTTGCAATAGCATGAATGGTATCTCACTTTAATTCATGAGA

*A. leucogrammicus*  GGGAGACATCGGCTTAATCCTTGCAATAGCATGAATGGTTTCCCACCTCAATTCATGGGA

*C. altivelis* GGGGGACATTGGCCTAATCTTTGCAATAGCATGAATGGTCTCCCACCTTAACTCCTGAGA

*E. epistictus* TGGTGACATCGGACTAATCTTTGCAATAGCATGAATAATGTCCCACCTTAACTCATGAGA

*E. octofasciatus* GGGAGATATTGGCCTAATCTTTGCAATGGCATGAATAGTCTCCCACCTCAACTCCTGAGA

*E. septemfasciatus* GGGGGACATTGGGCTAATCTTTGCAATGGCATGAATAGTATCCCACCTTAACTCCTGAGA

*T. dermopterus* CGGGGATATCGGCCTAATCTTTGCAATAGCATGAATAATGTCCCACCTCAACTCCTGAGA

*E. awoara* GGGGGACATTGGTCTGATCTTCGCAATAGCATGAATGGTATCTCACCTCAACTCATGAGA

*E. akaara* TGGGGACATCGGCCTAATCTTCGCAATAGCATGAATAGTATCCCACCTCAACTCATGAGA

*E. trimaculatus* TGGGGATATCGGACTAATTTTCGCAATAGCCTGAATAGTTTCCCATCTTAACTCGTGAGA

*E. areolatus* TGGTGATATCGGTCTAATCTTTGCAATAGCTTGAATAGTATCCCACCTTAACTCATGAGA

*V. albimarginata* CGGTGACGTGGGCTTAATCTTTGCCATAGCATGAATAGCAACCAACCTAAATTCTTGAGA

*V. louti* TGGTGATATCGGCTTAATCTTTACCATAGCATGAATAGCAACTAACCTTAATTCTTGAGA

*P. leopardus* CGGAGACGTAGGTTTAATTCTGGCTATAGCTTGAATAGCTACACATCTTAATTCATGAGA

*P. areolatus* AGGAGATGTAGGTTTAATCCTAGCAATAGCCTGAATAGCAACACATTTTAACTCATGAGA

*E. radiosum* CGGGGATGTCGGACTAATTTTTGCCATGGCATGAATGGCTACTAACCTAAATTCCTGGGA

*P. sieboldi* TGGTGACATTGGCCTCATCTTCGCCATAGCTTGAATGGCTATAAACCTCAACTCTTGAGA

*E. armatus* TGGTGACGTAGGCCTTATCTTCGCAATAGCATGAATAGCAACAAACCTCAACTCATGAGA

*R. oxyrhynchus* AGGAGATATCGGACTAATCCTTGCAATAGCTTGAATAGCAATAAACCTAAACTCCTGAGA

*K. cinerascens* AGGCGACATCGGCCTAATTTTTGCCATAGCATGAATAGCAATAAACCTCAACTCATGAGA

*T. chatareus* CGGAGATATTGGGCTAATTTTTGCTATAGCATGAATAGCAACCAACCTAAACTCATGAGA

*D. berycoides* GGGGGATATTGGACTAATCTTTACCTTAGCATGAATAGCAATAAACCTTAACTCCTGGGA

*H. typus* TGGAGATATCGGCTTAATTTTCGCTATAGCATGAATGGCAACAAACCTCAACTCCTGAGA

*M. argenteus* GGGGGATATCGGACTAATCTTCGCCATGGCATGAATAGCAACAAACCTCAACTCATGAGA

*S. chuatsi* CGGGGATGTTGGACTAATTTTCGCCATAGCATGGATAGCCACAAACTTTAACTCTTGGGA

*O. fasciatus* TGGAGATATCGGCTTAATCTTTGCCATAGCATGAATAGCAATAAACCTTAACTCATGAGA

*P. trilineatum* AGGTGATGTCGGACTAATCTTCGCCATAGCCTGAATAGCTATAAATCTTAACTCATGAGA

*M. salmoides* TGGAGATGTAGGCCTAATTTTTGCCATAGCTTGAATGGCCACAAACTTCAATTCTTGGGA

*P. tile* CGGAGACATCGGCCTGATCTTCGCTATAGCATGAATAGCAACAAACCTTAACTCCTGAGA

*L. argentimaculatus* CGGAGACATCGGCCTAATCTTCGCCATAGCATGAATAGCAACAAACCTCAACTCCTGAGA

*E. struhsakeri* CGGGGATATTGGACTAATTTTCGCCATAGCCTGAATAGCAACAAACCTCAACTCCTGAGA

*B. albus* AGGAGACATCGGACTAATCTTTGCTATAGCATGAATAGCTACCAACCTTAACTCCTGGGA

*C. auripes* CGGAGACATTGGGCTAATCCTAAGCATGGCCTGAATCGCAATAAACTTAAACTCATGAGA

*C. melampygus* CGGAGACATCGGACTAATCTTCGCAATAGCATGAATAGCTACAAACTTTAATTCGTGAGA

*L. calcarifer* AGGAGACATCGGACTGATCTTCACCATAGCATGAATCGCAATAAACCTCAACTCATGAGA

*S. maena* CGGGGACATTGGCTTAATCTTCGCTATAGCATGAATAGCAACAAACCTTAACTCATGAGA

*P. auriga* GGGTGACATCGGACTAATCTTCGCCATAGCTTGAATAGCAATAAACCTCAATTCATGAGA

*C. lucidus* CGGAGACATCGGCCTAATCTTCGCCATAGCCTGAATAGCAACCAACCTCAACTCATGAGA

*S. sihama* CGGAGACATCGGCCTGATCTTCGCTATAGCATGAATAGCAACAAACCTTAACTCCTGAGA

*C. loricula* CGGAGATGTCGGCCTACTCTTTGCCATAGCATGGATCGCAATAAATCTCAACTCCTGAGA

*A. trutta* TGGGGACATCGGACTTATCCTAGCCATAGCATGAATAGCAAGCAGCCTAAACTCATGGGA

*H. gemma* AGGTGACATTGGCCTGATTCTTGCCATGGCCTGAATTGCTTCGAACCTTAACTCCTGAGA

*P. flavescens* CGGAGATATCGGTTTAATTTTCGCTCTAGCATGAATAGCCACGAACCTTAACTCTTGAGA

*P. macrolepida* AGGGGACATCGGCCTAATTTTTGCTATAGCATGAATAGCCACAAACCTTAACTCCTGAGA

*S. canadensis* GGGTGATATCGGCCTTATCTTTGCTATAGCCTGGATGGCCACAAATCTAAACTCCTGGGA

*A. rogaa* AATACAGCAAATATTTGCCACTGCTAAAGACTTCGAC---CTCACCTTTCCACTACTTGG

*C. argus* AATACAACAAATTTTCTCTGCCGCAAAAGATTTTGAC---CTTACCTTTCCACTGCTTGG

*C. sonnerati* AATACAACAAATATTCATTACTGCCAAAGACTTCGAC---CTTACTTTTCCACTACTAGG

*E. fuscoguttatus* ACTACAACAAATCTTTGCAGTTGCCAAAGACTTTGAC---CTTACCTACCCACTTCTTGG

*E. coioides* ATTACAACAAATCTTTACCACCGCTAAAGACTTTGAC---CTCACCTACCCACTACTCGG

*E. bruneus* ACTACAACAAATTTTTGCAACTGCTAAAGACTTCGAC---CTCACCTACCCACTCCTAGG

*E. moara* ACTACAACAAATCTTTGCAACTGCTAAAGACTTTGAC---CTCACCTACCCACTCCTGGG

*E. lanceolatus*  ATTACAACAAATCTTTGCCACCGCTAAAGACTTTGAC---CTCACCTACCCCCTCCTCGG

*A. leucogrammicus*  ACTACAACAAATCTTTGCAACGGCTAAAGACTTTGAT---CTCACGTACCCACTCTTTGG

*C. altivelis* ACTACAACAAATCTTCACAACCGCTAAAGATTTTGAC---CTTACCTATCCACTCCTTGG

*E. epistictus* ACTACAACAAATCTTTGCAGCTGCTAAAGACTTTGAC---CTTACCTACCCACTCCTCGG

*E. octofasciatus* ATTACAACAAATTTTTACAACCGCTAAAGATTTCGAC---CTTACCTACCCACTTCTTGG

*E. septemfasciatus* ATTACAACAAATTTTTACAACAGCTAAAGATTTCGAC---CTCACCTACCCACTTCTTGG

*T. dermopterus* ACTACAACAAATCTTTTCAGCCGCCAAAGACTTCGAC---CTCACTTATCCCCTCCTTGC

*E. awoara* ACTACAACAAATCTTTGCAGCCGCTAAAAATTTTGAC---CTCACCTATCCCCTTCTTGG

*E. akaara* ACTGCAACAAATCTTTGCAGCCGCTAAAAACTTTGAC---CTTACCTACCCGCTCCTTGG

*E. trimaculatus* ATTACAACAAATCTTTGCAGTCGCCAAAAACTTTGAC---CTTACCTATCCACTCCTCGG

*E. areolatus* GCTACAACAAATCTTTGCAATCGCCAAAAACTTTGAC---CTCACCTACCCCCTTCTAGG

*V. albimarginata* AATACAACAGATCTTTGTAACCTCTAAAGACCTTGAC---CTTACTTTTCCCCTCCTTGG

*V. louti* AATCCAACAGATCTTTGTAACCTCTAAAGACCTTGAC---CTCACTTTTCCACTCCTTGG

*P. leopardus* AATTCAACAAATATTCGCTACCGCAAAAGATTTTGAC---TTAACTCTTCCTCTCCTTGG

*P. areolatus* AATTCAACAAATATTTGTTACTGGAAAAGATTTTGAC---CTGACTCTTCCCCTCCTGGG

*E. radiosum* AATACAACAAGTGTTTGTAGCCGCTAAAGGCTTTGAC---CTTACTTTCCCCCTCCTAGG

*P. sieboldi* ACTCCACCAGCTAGTCGCCTCATCCAAGAGCTATGAC---CTCACTTTCCCCCTCATCGG

*E. armatus* AATACAACAACTATTTGCAGCTGCCAAAGATATAGAC---CTTACTTTCCCACTTCTAGG

*R. oxyrhynchus* AATGCAACAAATATTTGCAGCCTCAAAAGGCTTAGAC---CTTACCCTCCCCCTACTAGG

*K. cinerascens* AATACAACAAATATTTGCAGCCGCAAAAGACCTCGAC---CTCACCTTCCCACTTCTAGG

*T. chatareus* GATACAACAAATATTTGCAGCTGCTAAAAACTTTGAC---CTCACTTTCCCCCTTCTAGG

*D. berycoides* AATACAACAGATCTTTGCAACTGCCAAAACATTTGAC---CTAACATTTCCCCTCTTAGG

*H. typus* ATTACAACAAATATTCGCAACTGCTAAAGACCTCGAC---CTTACTTTTCCTCTCCTGGG

*M. argenteus* AATGCAGCAAATATTCGCAGCCGCTAAAAATCTTGAC---CTCACTTTCCCACTCTTAGG

*S. chuatsi* AATGCAACAAATATTTGCAGCCGCCAAAGACTTTGAC---CTTACCTTCCCACTTCTAGG

*O. fasciatus* AATACAACAGATGTTCGCAGCCGCAAAAAACTTTGAC---CTTACCTTCCCGCTTCTAGG

*P. trilineatum* AATGCAACAAATATTTGCAGCCTCTAAAGACATGGAC---CTAACCTTTCCCCTTCTAGG

*M. salmoides* AATCCAACAAATATTCATTTCTGCTAATAATTTTGAT---CTCACTTTTCCCCTATTGGG

*P. tile* AATACAACAAATATTTGCAGCCGCCAAAGACTTTGAT---CTTACCTTTCCTCTTCTTGG

*L. argentimaculatus* AATACAACAAATATTCGCAGCTGCCAAAAACATAGAT---CTTACCTTTCCTCTCCTGGG

*E. struhsakeri* AATGCAACAAATATTTGCAGCCGCTAAAGACTTCGAC---CTTACCGTCCCTGTCTTAGG

*B. albus* AATACAACAGATATTTGTTGCTGCCAAAAACCTAGAC---CTTACCTTCCCCCTCCTGGG

*C. auripes* AATACAACAAATCTTTGCCGCTGCTAAAAACATAGAT---CTTACCCTACCCCTCCTTGG

*C. melampygus* AATACAACAAATATTTGCCGCAGCTAAAGACTTCGAC---CTTACTTACCCCCTTTTAGG

*L. calcarifer* AATACAACAAATATTTGCCTCTGCCAAAGACATAGAC---CTCACCCTCCCCCTACTAGG

*S. maena* AATACAACAGATAATAACCACTGCAAAGGATCTTGAC---CTAACCTTCCCCCTTATCGG

*P. auriga* GATACAACAAATATTTGCAGCCGCAAAAGACCTAAAC---TTAACCCCCCCCCTTCTTGG

*C. lucidus* AATACAACAAATATTTACAACAGCTAAAAATCAAGAC---CTTACCTTCCCCCTCTTAGG

*S. sihama* AATACAACAAATTTTCTCGACTGCTAAGGAATTCGAC---CTCACATACCCCCTGATCGG

*C. loricula* AATGCAACAAATTTTTGCAGCAGCTAAGGACACAGAC---ATAACCCTCCCCCTCCTCGG

*A. trutta* AATGCCGCAAGTCTTTACAGCCGCCCAAGCCCTTAATAGTCCCCTCCTTCCTCTTCTCGC

*H. gemma* AATACAACAGATCTTCACCTCAGCAAAAGGATTAGAT---CTTACCCTTCCCCTTCTCGG

*P. flavescens* GATACAACAAGTATTTACAGCCGCCAAAGACTTCGAT---CTTACTTTCCCCCTACTAGG

*P. macrolepida* AATACAACAGGTATTCACGGCCGCCAAAGACTTCGAT---CTCACTTTCCCCCTTCTAGG

*S. canadensis* AATACAACAAGTATTTACAACCGCCAAAGATTTTGAC---CTCACCTTCCCCCTACTAGG

*A. rogaa* GCTAATTGTAGCAGCCACGGGCAAATCTGCCCAATTCGGGCTACACCCGTGACTCCCCTC

*C. argus* ACTAATTTTAGCCGCCACGGGAAAATCCGCTCAATTCGGGTTACATCCATGACTTCCTTC

*C. sonnerati* GTTAATTGTGGCTGCCACAGGAAAGTCCGCCCAGTTCGGACTCCACCCATGACTCCCTTC

*E. fuscoguttatus* CCTAATTGTAGCCGCCACAGGTAAATCCGCTCAATTCGGACTACATCCTTGACTCCCTTC

*E. coioides* CTTAATTGTGGCCGCCACAGGTAAATCCGCCCAATTTGGGTTACATCCTTGACTCCCCGC

*E. bruneus* CCTAATCGTGGCCGCCACAGGTAAGTCCGCCCAATTCGGGTTACATCCATGACTTCCCTC

*E. moara* CCTAATCGTAGCCGCCACAGGTAAGTCCGCTCAATTCGGGTCACATCCATGACTTCCCTC

*E. lanceolatus*  TCTAATTGTAGCTGCTACAGGTAAATCGGCCCAATTCGGACTACACCCCTGACTCCCCTC

*A. leucogrammicus*  TTTAATTGTGGCCGCTACGGGTAAATCCGCCCAATTCGGATTACACCCGTGACTCCCTTC

*C. altivelis* CCTAATCGTAGCAGCAACAGGCAAATCTGCCCAATTCGGACTACACCCGTGACTACCCTC

*E. epistictus* ACTAATCGTGGCCGCCACAGGTAAATCTGCCCAATTTGGACTACACCCCTGACTTCCTTC

*E. octofasciatus* GCTAATCGTGGCCGCCACAGGTAAATCTGCCCAGTTCGGATTACACCCCTGACTTCCCTC

*E. septemfasciatus* ACTAATCGTGGCCGCCACAGGTAAATCTGCCCAATTCGGATTACACCCGTGACTTCCCTC

*T. dermopterus* ACTAATCGTAGCCGCCACGGGCAAGTCTGCCCAATTCGGACTACACCCCTGACTCCCCTC

*E. awoara* ATTAATCGTGGCTGCTACAGGAAAGTCCGCCCAATTTGGCCTACACCCCTGACTCCCTGC

*E. akaara* ATTAATCGTAGCCGCTACGGGTAAGTCCGCCCAATTCGGACTACACCCATGACTCCCTGC

*E. trimaculatus* ACTAATCGTAGCCGCTACAGGCAAATCCGCCCAGTTCGGATTACACCCATGACTCCCCGC

*E. areolatus* ATTAATCGTAGCTGCCACAGGCAAGTCTGCCCAATTCGGATTACACCCATGACTACCTGC

*V. albimarginata* CCTGATCGTAGCCGCTACTGGCAAATCTGCCCAATTTGGTCTCCATCCATGGCTTCCATC

*V. louti* CTTAATTGTAGCCGCTACTGGTAAGTCTGCCCAATTTGGCCTCCATCCATGGCTCCCGTC

*P. leopardus* ACTTATTCTCGCTGCCACCGGTAAATCCGCCCAATTTGGTCTTCACCCCTGACTTCCTTC

*P. areolatus* CCTCATTCTTGCTGCCACTGGTAAGTCCGCTCAATTTGGACTTCACCCCTGACTTCCTTC

*E. radiosum* ACTAATTGTTGCGGCCACAGGAAAGTCTGCCCAGTTCGGACTTCACCCTTGGCTTCCTTC

*P. sieboldi* CTTTATCGTTGCAGCCACCGGCAAGTCGGCACAGTTTGGCCTCCACCCCTGACTTCCTTC

*E. armatus* CCTAATTATTGCCGCTACCGGCAAATCGGCCCAATTTGGACTACACCCTTGACTTCCTTC

*R. oxyrhynchus* ACTAATTCTTGCTGCAACAGGAAAATCCGCCCAATTCGGACTACACCCATGACTCCCTTC

*K. cinerascens* ACTAATCCTTGCCGCAACCGGCAAGTCAGCCCAATTCGGACTACACCCATGACTTCCTTC

*T. chatareus* ACTTATCCTTGCCGCAACTGGTAAATCAGCCCAATTCGGCCTACACCCTTGACTTCCTTC

*D. berycoides* GCTTATTATTGCTGCCACTGGTAAATCTGCCCAATTTGGATTACACCCCTGGCTCCCCTC

*H. typus* ACTTATCATTGCCGCCACCGGTAAGTCAGCCCAATTTGGACTACACCCCTGGCTTCCCTC

*M. argenteus* TCTTATCGTCGCTGCCACTGGTAAATCGGCCCAGTTCGGCCTACATCCTTGGCTCCCCTC

*S. chuatsi* GCTGATTATTGCCGCTACCGGTAAATCAGCCCAATTTGGACTTCACCCATGGCTTCCCTC

*O. fasciatus* GCTAATTATTGCTGCCACTGGTAAATCAGCCCAATTCGGCCTACACCCCTGACTTCCCTC

*P. trilineatum* ACTTATCCTTGCTGCAACCGGAAAATCAGCCCAATTTGGTCTCCACCCCTGACTTCCCTC

*M. salmoides* GTTAATTATTGCCGCCACTGGTAAATCGGCCCAGTTTGGACTTCATCCTTGGCTTCCCTC

*P. tile* CCTAATCCTTGCAGCAACTGGAAAGTCAGCCCAATTCGGCCTTCACCCATGACTTCCTTC

*L. argentimaculatus* GCTCATCCTTGCAGCCACCGGAAAGTCAGCCCAATTTGGACTCCACCCATGACTTCCCTC

*E. struhsakeri* GTTGATTGTTGCCGCCACTGGTAAATCAGAGCAATTTGGAATACACCCGTGACTTCCCTG

*B. albus* ACTGATCCTCGCCGCCACTGGCAAATCAGCCCAATTTGGACTTCACCCCTGACTTCCTTC

*C. auripes* CCTAATCCTTGCCGCTACTGGTAAGTCCGCCCAATTTGGCCTCCACCCCTGACTTCCCGC

*C. melampygus* ATTAATTGTAGCTGCAACCGGAAAATCAGCTCAATTTGGACTTCACCCGTGACTTCCATC

*L. calcarifer* ACTCGTCATTGCTGCAACCGGTAAATCCGCTCAATTTGGACTCCATCCATGGCTCCCCTC

*S. maena* CTTAATTGTTGCTGCTACTGGCAAGTCCGCCCAATTTGGTCTCCACCCCTGACTCCCTTC

*P. auriga* CTTAATCGTCGCTGCCACTGGTAAATCTGCCCAATTTGGCCTTCACCCGTGGCTTCCCTC

*C. lucidus* CCTCATCCTTGCCGCAACTGGTAAATCAGCACAATTTGGACTTCACCCATGACTTCCCTC

*S. sihama* CCTCATTGTAGCTGCCACAGGAAAGTCAGCTCAGTTCGGGCTTCACCCGTGGCTTCCTGC

*C. loricula* TCTTATCCTTGCCGCCACTGGAAAATCAGCCCAGTTCGGCCTGCATCCATGGCTTCCCTC

*A. trutta* TGTGATCGCCGCCGCCACTGGTAAATCAGCCCAATTTGGACTCCACCCCTGACTTCCCTC

*H. gemma* ACTTATCCTCGCCGCAACTGGTAAATCAGCCCAATTTGGCCTTCACCCATGACTCCCTTC

*P. flavescens* ACTTATTGTTGCCGCCACCGGCAAGTCCGCCCAGTTCGGGCTTCATCCATGACTTCCCTC

*P. macrolepida* ATTAATTGTGGCAGCAACGGGCAAGTCTGCCCAATTTGGGCTCCATCCCTGGCTCCCCTC

*S. canadensis* ACTGATTGTTGCCGCCACCGGCAAATCAGCCCAATTTGGGCTTCATCCATGGCTTCCCTC

*A. rogaa* CGCCATGGAGGGCCCTACACCAGTTTCCGCCCTATTACACTCAAGTACAATAGTCGTTGC

*C. argus* CGCCATGGAAGGCCCCACACCAGTTTCTGCCCTATTACATTCAAGCACTATAGTTGTTGC

*C. sonnerati* TGCCATGGAAGGCCCCACACCAGTCTCTGCACTACTACACTCCAGCACTATGGTCGTCGC

*E. fuscoguttatus* TGCTATGGAAGGACCCACACCAGTATCTGCCTTACTCCATTCCAGCACTATAGTTGTTGC

*E. coioides* CGCTATAGAAGGACCCACACCAGTATCTGCCCTACTCCACTCCAGCACTATGGTCGTTGC

*E. bruneus* TGCTATAGAAGGCCCCACACCAGTATCTGCCCTACTTCACTCCAGCACCATGGTCGTTGC

*E. moara* TGCTATAGAAGGGCCCACACCGGTATCTGCCCTACTTCACTCCAGCACCATAGTCGTTGC

*E. lanceolatus*  TGCCATAGAAGGACCTACACCAGTATCCGCCCTACTCCACTCCAGCACTATGGTAGTTGC

*A. leucogrammicus*  TGCCATAGAAGGGCCTACACCAGTATCCGCCCTACTCCACTCCAGCACTATGGTTGTTGC

*C. altivelis* TGCCATAGAAGGACCCACACCAGTATCCGCCCTACTCCACTCTAGCACTATGGTTGTTGC

*E. epistictus* GGCCATAGAAGGCCCTACACCAGTTTCTGCCCTACTTCACTCCAGCACTATGGTTGTAGC

*E. octofasciatus* TGCCATAGAAGGCCCTACGCCGGTGTCTGCCTTACTCCACTCCAGCACTATGGTCGTTGC

*E. septemfasciatus* TGCCATAGAAGGCCCTACGCCGGTATCTGCCTTACTCCACTCCAGCACTATGGTTGTTGC

*T. dermopterus* CGCCATGGAGGGCCCTACACCAGTATCCGCTCTACTCCACTCCAGCACTATGGTTGTCGC

*E. awoara* TGCCATAGAAGGCCCTACACCAGTATCCGCACTACTCCACTCTAGCACCATAGTTGTTGC

*E. akaara* TGCCATGGAAGGTCCTACACCAGTATCTGCCCTACTTCACTCTAGCACTATAGTTGTTGC

*E. trimaculatus* TGCCATAGAAGGCCCTACACCAGTATCAGCCCTACTCCACTCCAGCACTATAGTTGTTGC

*E. areolatus* TGCCATAGAAGGTCCTACGCCAGTATCTGCCCTACTCCACTCCAGCACTATAGTTGTCGC

*V. albimarginata* CGCCATGGAAGGTCCTACGCCAGTATCTGCCCTACTTCACTCAAGCACGATGGTTGTCGC

*V. louti* CGCCATGGAGGGTCCTACGCCAGTATCTGCCCTACTCCACTCGAGCACTATGGTTGTCGC

*P. leopardus* TGCTATAGAAGGTCCTACACCGGTCTCTGCCCTACTACACTCCAGTACTATAGTCGTTGC

*P. areolatus* TGCTATAGAAGGCCCTACTCCGGTCTCCGCCCTACTACATTCCAGCACTATAGTCGTTGC

*E. radiosum* CGCTATGGAAGGTCCTACACCGGTCTCTGCCCTACTGCACTCCAGTACAATGGTTGTCGC

*P. sieboldi* CGCGATAGAGGGCCCGACACCAGTCTCTGCTCTCTTGCATTCAAGCACCATGGTTGTGGC

*E. armatus* CGCCATGGAAGGTCCTACACCGGTATCTGCCCTACTACATTCTAGCACCATGGTTGTTGC

*R. oxyrhynchus* CGCCATGGAAGGTCCCACACCGGTCTCTGCCCTACTTCACTCGAGCACAATGGTCGTAGC

*K. cinerascens* CGCTATGGAAGGCCCTACACCGGTCTCTGCCCTACTTCACTCCAGCACCATGGTCGTCGC

*T. chatareus* CGCCATGGAAGGCCCTACACCGGTCTCTGCCCTACTGCACTCCAGCACCATGGTCGTCGC

*D. berycoides* TGCCATAGAAGGCCCTACACCGGTCTCTGCCCTACTACACTCAAGCACAATAGTGGTCGC

*H. typus* TGCCATGGAAGGTCCTACGCCGGTCTCTGCCCTACTGCACTCGAGCACAATAGTGGTCGC

*M. argenteus* AGCCATAGAGGGTCCTACACCGGTCTCTGCCCTACTACATTCCAGCACCATGGTTGTTGC

*S. chuatsi* TGCCATAGAGGGTCCCACACCGGTCTCTGCCCTACTGCACTCCAGCACCATGGTTGTTGC

*O. fasciatus* TGCCATGGAGGGCCCTACACCGGTCTCTGCCCTACTGCATTCAAGCACCATGGTTGTTGC

*P. trilineatum* AGCCATGGAAGGTCCTACTCCAGTCTCTGCACTACTTCACTCAAGTACTATGGTAGTAGC

*M. salmoides* TGCCATAGAGGGTCCTACACCGGTCTCTGCCCTACTGCATTCCAGCACCATAGTCGTGGC

*P. tile* TGCCATGGAAGGCCCTACACCGGTCTCTGCCCTACTACACTCCAGCACAATGGTCGTCGC

*L. argentimaculatus* CGCCATGGAAGGCCCTACACCGGTCTCTGCCCTACTGCACTCCAGCACCATGGTCGTTGC

*E. struhsakeri* TGCCATAGAGGGTCCCACACCGGTCTATGCCATGCTGCACTCTAGCACTATGGATGTCGC

*B. albus* TGCCATAGAAGGTCCTACGCCGGTCTCTGCCCTACTGCACTCCAGCACAATAGTTGTCGC

*C. auripes* TGCAATAGAAGGCCCCACTCCGGTATCTGCCCTACTGCACTCCAGCACTATGGTCGTTGC

*C. melampygus* CGCAATGGAAGGCCCTACGCCGGTCTCTGCCCTACTACACTCCAGCACAATAGTTGTCGC

*L. calcarifer* CGCCATGGAGGGTCCCACACCAGTCTCTGCCCTACTACATTCAAGCACAATGGTTGTGGC

*S. maena* CGCAATGGAAGGCCCAACACCGGTCTCTGCCCTACTTCACTCCAGCACTATGGTCGTTGC

*P. auriga* TGCCATAGAGGGCCCTACACCGGTCTCTGCCCTACTTCACTCAAGTACTATAGTCGTAGC

*C. lucidus* TGCCATAGAGGGTCCCACACCGGTCTCCGCCCTACTGCACTCCAGCACTATGGTTGTTGC

*S. sihama* AGCCATGGAAGGCCCTACACCGGTCTCTGCCCTACTGCATTCCAGCACCATGGTTGTAGC

*C. loricula* TGCCATAGAGGGCCCTACACCGGTCTCTGCCCTACTACACTCTAGCACTATGGTAGTGGC

*A. trutta* CGCCATGGAAGGCCCTACACCAGTATCCGCTCTACTCCACTCCAGCACCATAGTAGTCGC

*H. gemma* TGCCATGGAAGGCCCTACGCCAGTTTCAGCTCTACTCCACTCCAGCACTATGGTAGTAGC

*P. flavescens* TGCTATAGAGGGTCCTACACCGGTCTCTGCCCTACTGCATTCCAGCACAATAGTAGTCGC

*P. macrolepida* GGCCATGGAGGGTCCTACGCCGGTATCTGCCCTACTACATTCCAGCACAATAGTTGTTGC

*S. canadensis* TGCCATAGAGGGTCCCACACCGGTCTCTGCCCTACTGCACTCCAGCACAATAGTTGTTGC

*A. rogaa* AGGAATCTTTCTTCTAGTACGTATAAGCCCCCTTTTAGAAAATAACCAAACCGCTTTAAC

*C. argus* AGGAATCTTTCTTCTAGTACGAATAAGCCCCCTCTTGGAAAATAACCAAACCGCCTTAAC

*C. sonnerati* AGGCATCTTCCTTTTAGTACGAATAAGCCCACTTTTAGAAAATAACCAAACTGCCCTAAC

*E. fuscoguttatus* AGGCATTTTTCTTCTAGTACGCATGAGCCCTCTCTTGGAAAACAATCCTACCGCCCTCAC

*E. coioides* AGGTATTTTCCTTCTAGTACGCATGAGCCCTCTCTTAGAAAACAACTCTACCGCCCTCAC

*E. bruneus* AGGTATCTTCCTCTTAGTACGCATGAGCCCCCTCTTAGAAAACAACCCTGCCGCCCTCAC

*E. moara* AGGTATCTTCCTCTTAGTACGCATGAGCCCCCTTTTAGAAAACAACCCTACCGCCCTCAC

*E. lanceolatus*  AGGCATCTTCCTCCTAGTACGTATAAGCCCCCTCTTAGAAAACAATCCTACCGCTCTCAC

*A. leucogrammicus*  AGGCATCTTCCTCCTAGTGCGCATGAGCCCCCTCTTAGAAAATAATACTACCGCCCTCAC

*C. altivelis* AGGCATCTTCCTTCTAGTACGCATGAGCCCCCTCTTAGAAAATAACCCCGCCGCCCTCAC

*E. epistictus* AGGCATCTTCCTACTAGTACGAATAAGCCCCCTTTTGGAAAACAACCCCACCGCCCTCAC

*E. octofasciatus* AGGTATCTTCCTCTTAGTACGAATGAGCCCCCTCTTAGAAAATAACCCAACTGCCCTCAC

*E. septemfasciatus* AGGTATCTTTCTCTTAGTACGAATAAGCCCCCTCTTAGAAAATAACCCAACTGCCCTCAC

*T. dermopterus* AGGCATCTTTCTCTTAGTTCGAATGAGCCCCCTATTAGAAAACAACCCAACCGCCCTTAC

*E. awoara* AGGAATTTTCCTCCTAGTCCGAATGAGCCCCCTTCTAGAGAATAATTCCACCGCTCTTAC

*E. akaara* AGGAATCTTCCTTCTAGTCCGAATGAGCCCCCTTCTAGAAAATAATTCCACCGCTCTTAC

*E. trimaculatus* AGGTATTTTTCTTCTAGTACGAATGAGCCCCCTTTTAGAAAATAATACAACCGCCCTCAC

*E. areolatus* AGGAATTTTCCTCTTGGTACGAATAAGCCCCCTCTTGGAAGACAATATTACTGCCCTAAC

*V. albimarginata* GGGAATCTTTCTTTTAGTACGAATAAGCCCCCTATTAGAAAACAACCCAACCGCCTTAAC

*V. louti* AGGAATCTTCCTTCTGGTACGTATAAGCCCCCTCCTAGAAAACAACCCAACCGCCTTAAC

*P. leopardus* AGGTATTTTTTTATTAGTGCGCCTGAGCCCTCTCTTAGAAAATAACCCTACCGCCCTTAC

*P. areolatus* AGGTATTTTTTTGTTAGTGCGCCTGAGCCCTCTTTTAGAAAACAACCCTACCGCCCTCAC

*E. radiosum* GGGCATCTTTCTACTAATTCGAATGAGCCCCCTGCTAGCAGAAAATCCCACTACCCTCAC

*P. sieboldi* AGGCATCTTCCTACTAGTCCGTATCAGCCCTATGATAGAAGACAACCAAACTGCTCTCAC

*E. armatus* CGGCATCTTCTTACTCATCCGCATGAGCCCCCTAATAGAAAACAACCAAACTGCCTTAAC

*R. oxyrhynchus* AGGAATCTTCCTCCTAATCCGAATGAGCCCCCTTTTAGAAACCAACCCTGCCGCTCTAAC

*K. cinerascens* AGGAATCTTCCTACTAGTCCGCATGAGCCCCCTCCTGGAAAACAACCCAACAGCCCTAAC

*T. chatareus* AGGAATCTTCTTACTCATCCGAATAAGCCCCCTAATAGAAAACAACCAAACCGCCCTAAC

*D. berycoides* AGGCATTTTCCTCCTAGTACGAATAAGCCCCTTAATAGAACATAACCAAACAGCTCTCAC

*H. typus* GGGCATTTTTCTTCTAGTCCGTATAAGCCCCCTAATAGAAAACAACCAGACCGCCCTAAC

*M. argenteus* AGGAATTTTCCTCTTAATCCGACTAAGTCCTCTGATAGAAAACAACCAAACTGCTTTAAC

*S. chuatsi* GGGAATTTTTCTCCTAGTCCGATTGAGCCCTCTAATGGAACACAACCAGACGGCTTTAAC

*O. fasciatus* AGGCATTTTCCTCCTAGTTCGTATGAGCCCCCTTTTAGAAAATAACCAAACAGCCCTCAC

*P. trilineatum* GGGTATTTTCCTCCTTGTTCGTATGAGCCCCCTCATAGAAAATAACCAAACCGCTCTAAC

*M. salmoides* AGGCATTTTTTTACTCATTCGATTAAGCCCTTTAATAGAGAATAACCAATTAGCTCTTAC

*P. tile* AGGAATTTTCCTCTTGGTCCGAATAAGCCCCCTTATAGAAAACAACCAAACTGCCCTAAC

*L. argentimaculatus* CGGAATCTTCCTCCTAGTCCGTATAAGCCCCCTCTTAGAAAATAACCAAACCGCCCTAAC

*E. struhsakeri* AGGTATTTTTCTCCTAGTCCGAATAAGCCCACTGATGGAAAACAATCAAACTGCTTTAAC

*B. albus* AGGCATTTTCCTCCTCATTCGAGTCAGCCCACTAATAGAAAATAATCAAACTGCACTAAC

*C. auripes* AGGGATCTTCCTTCTTATCCGAATAAGTCCTCTCTTAGAAAATAATCAAGTTGCCCTCAC

*C. melampygus* AGGAATCTTCCTCCTCGTTCGAATGAGCCCCCTCATAGAAAATAACCAAACCGCCCTAAC

*L. calcarifer* AGGTATCTTCCTCTTAGTCCGAATAAGTCCCTTAATAGAAAACAACCAGACTGCCCTCAC

*S. maena* GGGCATTTTTCTGCTTATTCGAATAAGCCCCCTTATAGAGAATAACCAAACTGCATTAAC

*P. auriga* AGGCATTTTTCTTCTGATCCGAATAAGCCCACTAATAGAAAACAACCAAACAGCCCTAAC

*C. lucidus* CGGAATTTTCCTTCTTGTCCGAATAAGTCCACTTCTAGAAAATAATCAAACAGCCCTTAC

*S. sihama* CGGAATTTTTCTGCTTGTTCGAATGAGCCCCCTTATCGAAACTAACCAGACTGCGCTTAC

*C. loricula* GGGCATTTTTCTGCTTATTCGAATAAGCCCCCTCTTGCAAGATAATCAACTAGCGCTAAC

*A. trutta* AGGCATTTTCCTTCTGATTCGAATAAACCCTCTTATAGAGACTAACCCTGTCGCCCTCAC

*H. gemma* AGGGATTTTCCTTCTCATTCGTCTCAGCCCACTATTTAAGGATAACCCCACAGCTTTAAC

*P. flavescens* AGGTATTTTTCTTTTGATCCGAATAAGCCCTTTATTAGCAGATAATCAAACTGCCCTTAC

*P. macrolepida* GGGAATTTTTTTGTTGATTCGAATGAGCCCCCTGCTAGCAGAAAACCCAACTGCCCTCAC

*S. canadensis* AGGTATCTTCCTTTTAATCCGAATAAGTCCTCTATTGGCAGAAAACCAAACCGCTCTTAC

*A. rogaa* CATCTGCCTTTGCCTCGGTGCCCTTACAACTTTATTTACAGCCACATGTGCCCTCACCCA

*C. argus* CATCTGCTTATGTCTCGGCGCTCTTACAACCCTGTTCACAGCCACATGTGCTCTAACCCA

*C. sonnerati* TATCTGCTTATGTCTTGGCGCTCTAACCACCTTATTCACAGCCACATGCGCCCTCACCCA

*E. fuscoguttatus* CACCTGCCTATGTCTTGGAGCCCTAACCACACTATTCACAGCCACATGCGCCCTAACCCA

*E. coioides* CACCTGTCTATGCCTTGGAGCCTTAACCACACTATTCACAGCCACATGTGCTTTAACCCA

*E. bruneus* CACCTGCCTATGTCTCGGAGCCCTAACCACACTATTCACAGCCACCTGCGCCTTAACCCA

*E. moara* CACCTGCCTATGTCTCGGAGCCCTAACCACACTGTTCACAGCCACATGCGCCTTAACCCA

*E. lanceolatus*  CACCTGCCTATGCCTCGGAGCCCTAACCACACTATTCACAGCTACCTGCGCCTTAACCCA

*A. leucogrammicus*  CACTTGCCTATGCCTTGGAGCCTTAACCACACTATTTACAGCCACATGTGCTCTCACCCA

*C. altivelis* CACCTGCCTATGCCTCGGAGCCCTAACTACGCTATTCACAGCCACATGTGCCCTAACCCA

*E. epistictus* CACCTGCTTATGCCTAGGTGCTTTAACAACATTATTTACAGCCACGTGCGCTCTAACCCA

*E. octofasciatus* TACCTGCCTATGTCTTGGTGCCCTAACCACCCTATTCACAGCTACCTGCGCTCTAACCCA

*E. septemfasciatus* TACCTGCCTATGCCTTGGTGCCCTAACCACACTATTCACAGCTACCTGCGCTCTAACCCA

*T. dermopterus* CACCTGCCTATGCCTCGGCGCCCTAACCACACTATTCACAGCCACATGCGCCCTAACCCA

*E. awoara* CACTTGCCTATGCCTTGGAGCCCTAACCACACTATTTACAGCCACATGTGCTTTGACCCA

*E. akaara* CACTTGCTTATGCCTTGGAGCCCTAACCACGCTATTTACAGCCACATGCGCTTTAACCCA

*E. trimaculatus* CACCTGCCTATGCCTCGGTGCCCTTACCACATTATTCACAGCCACATGTGCTTTAACCCA

*E. areolatus* CACCTGCCTATGCCTCGGAGCCCTAACCACTCTATTTACAGCCACATGCGCCTTAACCCA

*V. albimarginata* CATTTGCCTTTGCCTCGGTGCATTAACTACACTATTCACAGCCACCTGTGCCCTAACTCA

*V. louti* CATTTGCCTTTGCCTTGGTGCACTAACCACCCTATTCACAGCCACCTGTGCTCTAACTCA

*P. leopardus* CATCTGCCTTTGCCTAGGAGCCCTTACCACTATGTTTACAGCTACGTGTGCTCTTACCCA

*P. areolatus* CATCTGCCTTTGTCTTGGAGCCCTTACCACCATATTTACAGCTACATGCGCTCTTACCCA

*E. radiosum* AGTCTGTCTTTGCCTTGGTGCCTTAACAACTTTGTTTACAGCTATCTGCGCCCTAACACA

*P. sieboldi* GATCTGCCTTTGTTTAGGGGCCCTCACTACTATATTTACGGCCACTTGCGCCCTTACCCA

*E. armatus* TGTTTGCCTTTGCCTGGGCGCCTTAACCACCCTGTTTACTGCCACCTGTGCCCTCACCCA

*R. oxyrhynchus* CATTTGTTTATGCTTAGGAGCCTTAACAACCCTATTTACTGCCACCTGTGCTTTGACCCA

*K. cinerascens* CATCTGCCTATGCCTCGGTGCCCTAACAACTCTATTCACAGCCACCTGCGCCCTCACCCA

*T. chatareus* CGTCTGCCTATGCTTAGGTGCCCTAACCACCCTCTTCACTGCCACTTGCGCCCTTACCCA

*D. berycoides* CACCTGTCTTTGCCTAGGCGCCCTAACTACACTATTCACCGCTACCTGTGCTCTCACCCA

*H. typus* CACCTGCCTATGCCTAGGGGCTCTTACTACCCTATTTACTGCCACCTGTGCCCTCACCCA

*M. argenteus* TACTTGCTTATGTCTTGGTGCCCTAACCACCCTATTTACTGCCACATGTGCCCTTACCCA

*S. chuatsi* CGTATGTTTATGTTTAGGTGCCCTAACGACCCTATTCACCGCTACCTGTGCCCTCACTCA

*O. fasciatus* CATCTGCCTATGCCTTGGCGCCCTCACAACCCTCTTCACCGCCACTTGTGCCCTCACCCA

*P. trilineatum* TGCCTGCCTTTGCCTCGGAGCCCTTACCACACTATTTACTGCCACCTGCGCCCTCACCCA

*M. salmoides* CACCTGCCTATGTCTAGGCGCCCTCACCACCCTTTTTACCGCCACTTGTGCCCTCACCCA

*P. tile* AACCTGCCTCTGCCTGGGAGCCCTCACCACCCTATTTACAGCTACCTGTGCCCTCACCCA

*L. argentimaculatus* AACATGCTTATGCTTGGGAGCTCTAACAACCCTATTCACCGCTACTTGCGCACTTACCCA

*E. struhsakeri* CACCTGCCTATGCTTAGGAGCCCTAACCACACTATTTACCGCCACTTGTGCCCTCACTCA

*B. albus* CACCTGCCTCTGCTTAGGTGCTTTAACTACACTATTTACAGCCACCTGCGCCCTCACACA

*C. auripes* TACTTGCCTATGCTTAGGGGCCCTCACCACTCTATTTACTGCCACCTGTGCCCTCACCCA

*C. melampygus* AACCTGCCTCTGCTTAGGTGCCCTCACAACTTTCTTTACAGCCACCTGCGCCCTCACCCA

*L. calcarifer* CACCTGTCTCTGCCTAGGCGCCCTAACTACCCTCTTCACTGCCACCTGTGCCCTTCCCCA

*S. maena* AATTTGTCTCTGCCTTGGTGCCCTGACAACTGTTTTTACCGCCACCTGCGCCCTTACTCA

*P. auriga* AACCTGCCTCTGTCTCGGTGCCCTAACCACTGTATTTACCGCCACCTGTGCCCTCACACA

*C. lucidus* TCTCTGCTTATGCTTAGGGGCCCTAACCACTCTATTTACCGCCACTTGTGCCCTCACCCA

*S. sihama* CATTTGCCTTTGCCTTGGAGCCTTAACCACCTTATTCACCGCCACTTGCGCCCTCACCCA

*C. loricula* AACTTGCTTATGCTTGGGAGCCCTAACCACGCTATTCACCGCCACCTGCGCACTAACACA

*A. trutta* CATCTGCCTCTGCTTAGGTTCCCTAACAACCCTATTCACTGCCACATGTGCTCTTACTCA

*H. gemma* CATTTGCCTCTGCTTAGGAGCTTTAACAACACTATTTACCGCCACCTGTGCCCTCACCCA

*P. flavescens* CATCTGCCTTTGTCTAGGAGCCCTCACTACCCTATTTACCGCCACCTGTGCTTTAACCCA

*P. macrolepida* AGTTTGCCTTTGTCTCGGCGCACTCACAACTTTATTTACAGCTACATGTGCCCTAACCCA

*S. canadensis* CGTCTGTCTTTGCCTTGGCGCCCTCACCACCCTGTTCACAGCCACCTGCGCCTTAACCCA

*A. rogaa* AAATGACATCAAAAAAATCGTTGCATTTTCAACATCCAGTCAATTAGGACTAATAATAGT

*C. argus* AAATGATATTAAAAAAATTGTCGCATTTTCAACATCCAGTCAACTAGGCTTAATAATGGT

*C. sonnerati* AAATGACATTAAAAAGATCGTTGCATTCTCAACCTCAAGCCAACTAGGTCTGATAATAGT

*E. fuscoguttatus* AAACGATATTAAAAAAATTGTTGCATTTTCAACATCAAGCCAGCTAGGTTTAATAATAGT

*E. coioides* AAACGACATCAAAAAAATTGTTGCATTCTCAACATCAAGCCAACTAGGCCTGATAATAGT

*E. bruneus* GAACGACATTAAAAAAATTGTTGCATTCTCAACATCAAGTCAACTAGGCCTAATAATAGT

*E. moara* GAACGACATTAAAAAAATTGTTGCATTCTCAACATCAAGTCAACTAGGCCTAATAATAGT

*E. lanceolatus*  AAACGACATCAAAAAAATTGTTGCATTCTCAACATCAAGCCAACTAGGCCTAATAATAGT

*A. leucogrammicus*  AAACGATATTAAAAAAATCGTCGCATTCTCAACATCAAGCCAACTAGGCCTAATGATAGT

*C. altivelis* AAACGACATCAAAAAAATCGTTGCATTCTCAACATCAAGCCAACTAGGCTTAATAATAGT

*E. epistictus* AAATGACATTAAAAAAATTGTTGCATTCTCAACATCAAGTCAACTGGGCCTAATAATGGT

*E. octofasciatus* AAACGATATCAAAAAAATTGTTGCCTTCTCAACATCCAGTCAACTAGGTTTAATAATAGT

*E. septemfasciatus* AAACGACATCAAAAAAATTGTTGCCTTCTCAACATCCAGTCAACTAGGTTTGATAATAGT

*T. dermopterus* AAACGATATTAAAAAAATCGTTGCATTCTCAACATCCAGTCAATTAGGCCTAATAATAGT

*E. awoara* AAACGACATCAAAAAAATTGTTGCATTCTCAACATCCAGCCAATTGGGCTTAATAATAGT

*E. akaara* AAACGACATTAAAAAAATTGTTGCATTCTCAACATCCAGTCAGCTAGGCTTAATAATAGT

*E. trimaculatus* AAATGATATCAAAAAAATCGTTGCATTCTCAACATCCAGTCAACTAGGCTTAATAATAGT

*E. areolatus* AAATGATATCAAAAAAATTGTTGCATTCTCAACATCAAGCCAGCTAGGCTTAATAATAGT

*V. albimarginata* AAACGATATTAAAAAAATTGTTGCATTCTCTACATCTAGTCAACTAGGCCTAATGATAGT

*V. louti* AAATGACATCAAAAAAATCGTTGCATTCTCTACATCCAGCCAGCTAGGCCTAATGATAGT

*P. leopardus* AAACGACATTAAGAAAATTGTTGCCTTCTCAACATCCAGCCAACTCGGACTGATAATAGT

*P. areolatus* AAACGACATCAAGAAAATTGTTGCTTTTTCAACATCCAGCCAACTTGGCTTAATGATAGT

*E. radiosum* AAACGATATTAAGAAAATTGTTGCATTCTCAACATCTAGTCAGCTAGGACTAATAATAGT

*P. sieboldi* AAATGACATCAAGAAAATTGTAGCCTTCTCCACATCCAGTCAGCTGGGCTTAATGATGGT

*E. armatus* AAACGATATTAAAAAAATCGTCGCATTCTCAACATCCAGCCAACTAGGCCTCATAATAGT

*R. oxyrhynchus* AAATGATATCAAAAAAATCGTTGCATTTTCAACATCTAGCCAATTAGGCCTAATAATAGT

*K. cinerascens* GAATGACATTAAAAAAATCGTAGCATTCTCCACATCCAGCCAACTAGGACTGATAATAGT

*T. chatareus* AAACGATATTAAAAAAATCGTTGCATTTTCCACATCCAGCCAGCTTGGCCTCATGATAGT

*D. berycoides* GAATGATATTAAAAAAATCGTTGCGTTCTCTACATCAAGTCAACTAGGCCTAATAATAGT

*H. typus* AAACGACATCAAAAAAATCGTTGCATTCTCTACATCCAGCCAACTAGGTTTAATGATAGT

*M. argenteus* AAATGACATCAAAAAAATCGTTGCTTTCTCTACATCAAGCCAACTAGGCCTAATAATGGT

*S. chuatsi* AAATGACATCAAAAAAATCGTTGCATTCTCTACATCTAGCCAATTAGGCCTAATGATGGT

*O. fasciatus* AAACGACATCAAAAAAATTGTTGCATTCTCAACATCCAGCCAACTAGGCCTTATAATAGT

*P. trilineatum* AAATGACATTAAAAAAATTGTTGCTTTCTCTACATCTAGTCAACTAGGCCTAATAATAGT

*M. salmoides* GAATGATATCAAAAAAATCGTTGCATTCTCTACATCAAGCCAGCTGGGCCTAATGATAGT

*P. tile* AAACGACATCAAAAAAATCGTTGCCTTCTCCACATCAAGTCAGCTAGGATTAATAATGGT

*L. argentimaculatus* AAACGACATCAAAAAAATCGTCGCTTTCTCCACATCCAGCCAACTAGGCCTAATAATGGT

*E. struhsakeri* AAACGACATCAAAAAAATCGTTGCCTTCTCCACATCAAGCCAACTAGGCCTAATAATAGT

*B. albus* AAATGACATCAAAAAAATTGTCGCCTTCTCTACCTCCAGCCAACTAGGACTAATAATAGT

*C. auripes* AAATGACATCAAAAAAATCGTTGCCTTCTCAACCTCCAGCCAACTAGGTCTAATAATAGT

*C. melampygus* AAACGATATCAAAAAAATCGTTGCCTTCTCAACATCAAGTCAACTAGGCCTAATAATAGT

*L. calcarifer* AAATGATATCAAGAAAATCGTTGCCTTCTCCACCTCTAGCCAACTAGGTCTAATAATAGT

*S. maena* AAATGATATCAAAAAAATCGTTGCTTTCTCGACATCAAGCCAACTAGGCCTAATAATAGT

*P. auriga* AAATGACATCAAAAAAATTGTTGCTTTCTCAACCTCAAGCCAGCTAGGCTTGATAATAGT

*C. lucidus* AAATGATATTAAAAAAATCGTTGCTTTCTCTACATCAAGCCAACTCGGCCTTATGATAGT

*S. sihama* GAATGATATTAAGAAAATCGTTGCTTTTTCAACATCCAGCCAGCTAGGATTAATGATAGT

*C. loricula* AAACGACATCAAAAAAATCGTTGCTTTCTCTACATCTAGTCAGCTAGGCCTGATAATAGT

*A. trutta* AAACGACATCAAGAAAATCGTAGCATTCTCCACGTCAAGTCAGCTAGGACTGATAATAGT

*H. gemma* AAATGACATCAAAAAAATTGTTGCATTCTCCACATCAAGCCAGCTTGGCCTAATAATAGT

*P. flavescens* AAATGACATTAAAAAAATTGTTGCGTTCTCAACATCAAGTCAATTAGGACTAATAATGGT

*P. macrolepida* AAATGACATCAAAAAAATTGTTGCATTCTCAACATCAAGCCAGCTAGGGCTAATAATAGT

*S. canadensis* AAATGATATCAAAAAAATTGTTGCATTCTCAACATCAAGCCAATTAGGCTTAATAATAGT

*A. rogaa* TACTATTGGATTAAACCAACCCCAACTAGCCTTTCTTCACATCTGCACTCACGCTTTCTT

*C. argus* TACTATTGGCTTAAACCAACCCCAACTAGCCTTCCTCCACATCTGTACCCACGCCTTCTT

*C. sonnerati* AACAATTGGACTAAATCAACCCCAATTAGCCTTCCTCCACATTTGCACCCACGCCTTCTT

*E. fuscoguttatus* AACAATTGGACTAAACCAGCCCCAATTGGCATTCCTTCACATCTGTACCCACGCTTTCTT

*E. coioides* AACAATTGGATTAAACCAACCCCAACTGGCATTTCTCCATATCTGCACCCACGCCTTCTT

*E. bruneus* AACAATTGGATTAAACCAACCCCAACTGGCATTTCTTCACATCTGCACCCACGCTTTCTT

*E. moara* AACAATTGGATTGAACCAGCCCCAGCTGGCATTTCTTCACATCTGCACCCACGCTTTCTT

*E. lanceolatus*  AACAATTGGATTAAACCAACCCCAACTGGCATTCCTTCACATCTGCACCCACGCTTTCTT

*A. leucogrammicus*  AACAATTGGGTTAAACCAACCTCAACTAGCATTCCTCCACATCTGCACCCACGCCTTCTT

*C. altivelis* AACAATTGGTTTAAACCAACCCCAACTAGCATTCCTTCATATCTGTACCCACGCCTTCTT

*E. epistictus* AACAATTGGACTAAACCAACCCCAGCTGGCATTCCTTCACATCTGTACCCACGCCTTCTT

*E. octofasciatus* AACAATTGGCCTAAATCAACCCCAACTAGCATTCCTCCACATCTGCACTCACGCTTTTTT

*E. septemfasciatus* AACAATTGGCCTAAATCAACCCCAACTAGCATTCCTCCACATCTGCACCCATGCTTTCTT

*T. dermopterus* GACAATCGGCTTAAACCAGCCCCAACTAGCATTCCTTCACATCTGCACCCACGCATTCTT

*E. awoara* AACAATTGGGTTAAACCAACCCCAATTAGCATTACTTCACATCTGTACCCACGCCTTTTT

*E. akaara* AACAATCGGGTTAAACCAGCCCCAACTAGCATTTCTTCACATCTGTACTCACGCCTTCTT

*E. trimaculatus* AACAATTGGACTAAACCAACCCCAATTAGCATTTCTTCACATCTGCACTCACGCTTTCTT

*E. areolatus* AACAATTGGGCTAAACCAACCCCAACTAGCTTTTCTTCATATCTGTACCCACGCCTTCTT

*V. albimarginata* TACAATTGGTCTTAATCAACCCCAACTCGCCTTCCTTCACATCTGCACTCATGCCTTTTT

*V. louti* TACGATTGGACTCAACCAACCCCAACTCGCTTTCCTCCATATTTGTACCCACGCCTTTTT

*P. leopardus* AACTATTGGTCTCAACCAACCCCAACTAGCCTTCCTTCATATCTGCACCCATGCATTCTT

*P. areolatus* AACCATCGGTCTCAACCAACCCCAACTAGCTTTCCTCCATATCTGTACCCACGCCTTCTT

*E. radiosum* GACCCTCGGATTAAACCAGCCTCAATTAGCTTTCCTCCATATTTGTACCCACGCCTTCTT

*P. sieboldi* TACCATTGGCCTTAACCAACCCCAGCTCGCCTTCCTACACATCTGCACGCATGCCTTCTT

*E. armatus* AACTATTGGACTTAACCAACCTCAACTTGCCTTTCTTCACATCTGTACACATGCCTTCTT

*R. oxyrhynchus* CACAATTGGACTAAACCAACCACAACTCGCCTTCCTCCACATTTGCACCCACGCCTTCTT

*K. cinerascens* AACCATCGGTCTAAACCAACCCCAACTCGCATTCCTCCACATTTGCACCCACGCCTTCTT

*T. chatareus* TACAATCGGCCTCAATCAACCACAATTAGCCTTCCTCCACATTTGTACCCACGCCTTTTT

*D. berycoides* GACCATCGGCCTAAACCAGCCCCAACTTGCTTTCCTTCATATCTGTACCCACGCTTTCTT

*H. typus* AACTATTGGGCTTAATCAACCCCAACTTGCCTTCCTTCATATCTGCACCCACGCCTTCTT

*M. argenteus* TACCATCGGCCTAAACCAACCTCAACTAGCCTTCCTCCATATTTGCACCCATGCCTTCTT

*S. chuatsi* TACCATCGGATTAAACCAACCTCAACTTGCCTTCCTTCACATCTGTACGCACGCTTTCTT

*O. fasciatus* AACCATCGGACTAAACCAACCTCAACTTGCCTTCCTACACATTTGCACCCATGCTTTCTT

*P. trilineatum* AACCATTGGATTAAATCAACCCCAACTTGCCTTCCTTCACATCTGCACCCACGCATTCTT

*M. salmoides* GACAATCGGACTAAACCAACCCCAACTTGCCTTCCTCCATATCTGCACCCACGCATTCTT

*P. tile* CACCATCGGACTTAATCAACCCCAACTTGCCTTCCTGCACATCTGTACCCACGCCTTCTT

*L. argentimaculatus* CACAATTGGACTTAACCAACCTCAACTTGCCTTCCTCCACATCTGCACCCATGCTTTCTT

*E. struhsakeri* AACTATCGGGCTTAATCAACCCCAACTTGCATTCCTCCACATCTGCACCCACGCTTTCTT

*B. albus* GACCATCGGCCTAAACCAGCCCCAACTTGCCTTCCTCCATATCCGTACCCACGCATTCTT

*C. auripes* TACCATCGGCCTAAACCAACCTCAACTTGCATTCCTTCATATCTGCACCCACGCCTTCTT

*C. melampygus* AACTATTGGCCTTAACCAACCCCAACTTGCCTTCCTCCATATCTGTACACACGCATTCTT

*L. calcarifer* TACCATTGGACTTAACCAACCACAATTAGCCTTCCTACACATTTGTACTCATGCATTCTT

*S. maena* AACTATCGGGTTAAACCAGCCTCAACTTGCATTCCTCCACATTTGTACCCACGCCTTCTT

*P. auriga* TACTATTGGTCTTAACCAACCACAACTTGCTTTCCTCCATATCTGCACTCACGCCTTCTT

*C. lucidus* AACCATTGGACTAAATCAGCCCCAACTTGCCTTCCTTCACATCTGCACTCACGCATTTTT

*S. sihama* GACTATTGGGCTAAACCAACCCCAACTTGCATTCCTGCACATCTGTACTCACGCCTTCTT

*C. loricula* TGCCATTGGACTAAACCAACCTCAACTCGCCTTCATTCACATCTCCACTCACGCCTTTTT

*A. trutta* TACAATCGGATTAAACCAACCCCAACTCGCCTTCCTACACATCTGCACACACGCTTTCTT

*H. gemma* TACAATTGGCCTTAATCAACCTCAACTCGCTTTCCTTCATATCTGCACACACGCTTTCTT

*P. flavescens* GACTATTGGCCTAAACCAGCCACAGCTCGCCTTCCTCCATATTTGCACCCACGCCTTCTT

*P. macrolepida* TACACTAGGGCTAAATCAACCCCAACTAGCTTTCCTTCACATTTGTACCCATGCTTTCTT

*S. canadensis* AACCATTGGACTAAATCAACCCCAATTAGCCTTTCTACATATCTGCACACATGCCTTCTT

*A. rogaa* CAAAGCTATATTATTCCTATGCTCCGGCTCAATTATTCATAGCCTTAATGACGAACAAGA

*C. argus* TAAAGCTATGCTATTCCTATGCTCCGGCTCGATTATTCACAGCCTAAATGACGAACAAGA

*C. sonnerati* CAAAGCCATACTATTCCTCTGCTCTGGTTCAATTATCCACAGCCTTAATGACGAACAAGA

*E. fuscoguttatus* CAAAGCAATACTTTTCCTATGTTCCGGCTCCATCATCCACAGTCTTAACGACGAACAAGA

*E. coioides* TAAAGCAATACTTTTCCTATGCTCTGGCTCCATTATTCACAGCCTTAATGACGAACAAGA

*E. bruneus* TAAAGCAATACTTTTCCTATGCTCCGGCTCCATTATCCACAGCCTTAATGACGAACAAGA

*E. moara* TAAAGCAATACTTTTCCTATGTTCCGGCTCCATTATCCACAGCCTCAATGACGAACAAGA

*E. lanceolatus*  CAAAGCAATACTTTTCCTATGTTCTGGCTCCATTATTCACAGCCTCAACGACGAACAAGA

*A. leucogrammicus*  TAAAGCAATGCTTTTCCTATGTTCCGGCTCCATTATTCATAGCCTTAATGACGAACAAGA

*C. altivelis* TAAAGCAATACTTTTCCTATGCTCCGGCTCCATTATTCACAGCCTTAATGACGAACAAGA

*E. epistictus* TAAAGCAATACTCTTTCTATGTTCCGGCTCTATCATTCACAGCCTTAATGACGAACAAGA

*E. octofasciatus* CAAAGCAATGCTTTTCCTATGCTCCGGCTCTATTATCCATAGCCTTAACGACGAACAAGA

*E. septemfasciatus* CAAAGCAATGCTCTTCCTATGTTCCGGCTCTATTATTCATAGCCTCAACGACGAACAAGA

*T. dermopterus* TAAAGCAATACTCTTCCTATGCTCCGGCTCTATTATTCACAGCCTCAACGACGAACAGGA

*E. awoara* TAAAGCAATGCTCTTCCTATGTTCAGGCTCTATTATTCATAGCCTCAACGACGAACAAGA

*E. akaara* TAAAGCAATACTCTTCCTATGTTCAGGCTCTATTATTCATAGCCTTAATGACGAACAAGA

*E. trimaculatus* TAAAGCAATACTCTTCTTATGTTCCGGCTCCATTATTCACAGTCTCAATGATGAACAAGA

*E. areolatus* TAAAGCCATACTCTTCTTATGCTCAGGCTCTATCATTCACAGCCTTAACGACGAACAAGA

*V. albimarginata* CAAGGCTATACTCTTCCTATGCTCTGGCTCAATCATTCACAGCCTTAACGACGAGCAAGA

*V. louti* TAAAGCCATGCTCTTCCTCTGCTCGGGCTCAATCATTCACAGTCTCAACGACGAGCAAGA

*P. leopardus* TAAGGCTATACTCTTCCTTTGTTCTGGTTCAATTATCCACAGCCTAAATGACGAACAAGA

*P. areolatus* CAAAGCAATACTCTTCCTTTGTTCTGGTTCAATTATTCACAGCCTCAACGACGAGCAAGA

*E. radiosum* CAAGGCCATACTCTTTTTATGCTCGGGCTCAATTATTCACAGTCTGAATGATGAGCAAGA

*P. sieboldi* CAAGGCCATGCTATTCCTCTGCTCCGGGTCAATTATCCACAGCCTTAATGACGAGCAAGA

*E. armatus* TAAAGCTATACTCTTCCTTTGTTCAGGTTCGATCATTCATAGCTTAAATGATGAGCAAGA

*R. oxyrhynchus* TAAGGCAATACTCTTCCTCTGCTCCGGATCTATTATCCACAGTCTAAACGACGAACAAGA

*K. cinerascens* CAAAGCCATGCTTTTCCTATGCTCCGGTTCTATTATCCACAGCCTAAACGATGAACAAGA

*T. chatareus* CAAAGCAATGCTCTTCCTATGCTCCGGCTCAATTATCCATAGTCTAAACGACGAACAGGA

*D. berycoides* CAAAGCAATATTATTCCTCTGCTCCGGATCGATTATTCACAGCCTAAATGACGAACAAGA

*H. typus* TAAAGCAATACTCTTTCTCTGCTCCGGCTCCATTATTCACAGTCTAAACGATGAACAAGA

*M. argenteus* TAAAGCAATACTTTTCCTCTGCTCAGGCTCAATCATCCACAGCCTTAACGACGAACAAGA

*S. chuatsi* CAAAGCAATACTCTTTCTATGCTCAGGTTCAATCATTCACAGCTTAAATGACGAACAAGA

*O. fasciatus* CAAAGCAATGCTTTTCCTCTGCTCTGGCTCTATTATTCACAGCCTAAATGATGAACAAGA

*P. trilineatum* CAAAGCAATACTCTTCCTATGCTCCGGATCGATCATTCACAGTCTTAACGACGAACAGGA

*M. salmoides* TAAAGCCATATTATTCTTATGCTCCGGCTCAATTATCCATAGCCTAAACGACGAACAAGA

*P. tile* CAAAGCAATACTATTCCTATGCTCGGGCTCAATTATTCACAGCCTCAACGACGAACAAGA

*L. argentimaculatus* CAAGGCTATACTTTTCCTCTGCTCTGGCTCAATCATCCACAGCCTTAATGATGAACAAGA

*E. struhsakeri* CAAAGCTATACTCTTCCTATGTTCCGGCTCAATTATTCACAGCCTTAACGACGAGCAAGA

*B. albus* TAAAGCGATACTGTTTCTCTGCTCGGGGGCAATCATCCATAGCCTCAATGACGAGCAAGA

*C. auripes* CAAGGCAATACTCTTCCTATGCTCCGGCTCAATTATCCACAGCCTTAATGACGAGCAAGA

*C. melampygus* CAAAGCAATACTCTTCCTCTGCTCAGGCTCTATTATTCACAGCCTAAACGACGAACAAGA

*L. calcarifer* CAAAGCTATGCTCTTCCTCTGCTCAGGCTCTATTATCCACAGCTTGAACGACGAACAAGA

*S. maena* TAAAGCCATGCTATTTCTCTGCTCAGGTTCAATCATTCACAGCCTTAATGACGAACAAGA

*P. auriga* TAAAGCAATGCTCTTCCTCTGCTCAGGCTCAATTATTCACAGCCTAAACGATGAGCAAGA

*C. lucidus* CAAAGCAATACTTTTCCTCTGCTCAGGCTCAATTATTCACAGCCTTAACGACGAACAAGA

*S. sihama* TAAGGCCATACTCTTCCTTTGCTCAGGCTCAATCATCCACAGCCTAAATGATGAGCAAGA

*C. loricula* TAAAGCTTTACTGTTCTTATGCTCGGGCTCAATTATCCACAGCCTCGACGATGAACAGGA

*A. trutta* CAAAGCTATGCTTTTCCTCTGCTCTGGCTCTATTATCCACAATTTAAACGACGAACAGGA

*H. gemma* CAAAGCTATATTATTTCTGTGTTCAGGATCAATCATTCACAGCCTAAATGATGAACAAGA

*P. flavescens* TAAAGCAATACTTTTCCTCTGTTCTGGCTCTATTATTCATAGCCTCAACGACGAACAAGA

*P. macrolepida* CAAAGCAATACTTTTTCTATGCTCAGGTTCAATTATTCATAGCCTAAACGACGAGCAAGA

*S. canadensis* CAAAGCAATGCTTTTCCTCTGCTCTGGCGCTATTATTCACAGTCTCAATGATGAGCAAGA

*A. rogaa* CATCCGAAAAATAGGAGGCATACACCACCTCACCCCCTTCACCTCCACTTGCTTAACTAT

*C. argus* TATCCGAAAAATAGGAGGAATACATCATCTTGTTCCTTTTACCTCCACCTGCTTGACAAT

*C. sonnerati* CATCCGAAAAATGGGAGGAATACATCACCTTACCCCATTCACATCCACCTGCCTAACCAT

*E. fuscoguttatus* CATCCGAAAAATGGGCGGCATGCACCGCCTCACCCCCTTTACTTCCTCCTGTCTTACTAT

*E. coioides* CATCCGAAAAATAGGCGGCATACACCGCCTTACCCCCTTTACCTCTTCCTGCCTTACCAT

*E. bruneus* CATCCGAAAAATAGGTGGCATGCAGCGTCTTACCCCTTTTACCTCTTCTTGCCTTACCAT

*E. moara* CATCCGAAAAATAGGCGGCATACACCATCTTACCCCCTTTACCTCTTCTTGCCTTACCAT

*E. lanceolatus*  CATTCGAAAAATAGGTGGTATGCACCGTCTCACCCCCTTTACCTCTTCTTGCCTTACCAT

*A. leucogrammicus*  TATCCGAAAAATAGGAGGCATACACCGCCTTGTCCCCCTCACCTCTTCTTGCCTTACTAT

*C. altivelis* TATTCGAAAAATAGGCGGCATACACCGCCTCACCCCCTTTACCTCCTCTTGCCTCACCAT

*E. epistictus* TATCCGAAAAATAGGTGGTATACACCGTCTTGCCCCCTTTACCTCTACCTGCCTTACCAT

*E. octofasciatus* CATCCGAAAAATGGGGGGCATGCACCGTCTTACCCCCTTTACCTCTACTTGTCTTACCAT

*E. septemfasciatus* CATCCGAAAAATAGGGGGCATGCACCGTCTTGCCCCCTTTACCTCTACTTGTCTTACCAT

*T. dermopterus* TATCCGAAAAATAGGAGGCATGCACCGTCTTACCCCTTTCACCTCTACCTGTCTCACCAT

*E. awoara* CATTCGTAAAATAGGAGGCATGCACCGCCTTACCCACTTCACTTCTACCTGTCTAACCAT

*E. akaara* CATTCGCAAAATAGGAGGGATGCACCGCCTTACCCCCTTCACCTCTACCTGCCTCACCAT

*E. trimaculatus* CATCCGCAAAATAGGAGGTATGCACCGCCTCACCCCTTTCACCTCCACCTGTCTCACTAT

*E. areolatus* CATTCGCAAAATAGGAGGCATGCACCGTCTCACCCCCTTCACCTCTTCCTGCCTCACCAT

*V. albimarginata* CATTCGCAAAATAGGAGGTATACATCACCTCACTCCATTCACATCTACCTGCTTAACAAT

*V. louti* CATCCGCAAAATAGGAGGTATACATCACCTCACTCCATTCACATCTACCTGCCTAACTAT

*P. leopardus* TATTCGAAAAATGGGAGGAATACACCACCTTACCCCCTTCACCTCGACTTGCCTAACCAT

*P. areolatus* TATTCGCAAAATAGGAGGAATACAACACCTCACACCCCTCACCTCAACTTGCCTGACTAT

*E. radiosum* TATTCGCAAAATAGGGGGCATGCACCACCTTACCCCTTTTACCTCTTCTTGCCTAACAAT

*P. sieboldi* TATCCGGAAAATGGGTGGCATGCACCACTTGACCCCCTTCACCTCCTCTTGCTTTACCCT

*E. armatus* TATCCGAAAAATGGGGGGCATACATCATCTTACTCCTTTTACATCTTCCTGCCTCACTAT

*R. oxyrhynchus* TATCCGAAAAATGGGGGGTATGCACCACCTCACCCCCTTTACCTCCTCCTGCCTTACCAT

*K. cinerascens* CATCCGAAAAATAGGAGGAATACACCATCTCACTCCCTTCACATCCTCTTGCCTCACTAT

*T. chatareus* CATCCGAAAAATAGGGGGCATACACCATTTAACCCCCTTCACATCCTCCTGCCTTACTAT

*D. berycoides* CATTCGAAAAATAGGAGGAATACACCACCTTACTCCATTTACAGCATCCTGCTTTACTAT

*H. typus* CATCCGAAAAATAGGAGGAATACACCACCTCACCCCCTTTACATCCTCCTGCCTTACTAT

*M. argenteus* CATTCGAAAAATAGGAGGCATACATCATCTTACCCCTTTCACATCCTCTTGCCTCACCAT

*S. chuatsi* CATTCGCAAGATAGGAGGTATACATCACCTCACTCCTTTTACATCCTCCTGCCTTACCAT

*O. fasciatus* CATTCGAAAAATAGGAGGAATACATCACCTTACCCCCTTCACATCTTCCTGCCTCACTGT

*P. trilineatum* CATCCGAAAAATAGGAGGTATACATCACCTCACACCTTTTACATCCTCCTGCCTAACTAT

*M. salmoides* TATTCGAAAAATGGGAGGAATACACCATTTAACCCCCTTCACCTCCTCATGCCTCACCAT

*P. tile* CATCCGAAAAATGGGGGGAATACACCACCTCACCCCCTTTACATCTTCTTGTCTTACCCT

*L. argentimaculatus* CATCCGAAAAATAGGAGGAATGCACCACCTCACCCCCTTTACATCATCCTGCCTAACCCT

*E. struhsakeri* CATTCGGAAAATAGGAGGCATGCACCATCTCACCCCCTTCACATCCTCCTGCTTTACCAT

*B. albus* CATCCGTAAAATAGGAGGAATGCACCACCTTGCCCCATTTACATCCTCCTGCATAACTCT

*C. auripes* TATCCGAAAAATAGGAGGAATACACCACCTTGCACCATTCACCTCTTCCTGCATAACAAT

*C. melampygus* CATCCGCAAAATAGGAGGAATGCATCACCTCACCCCATTCACCTCCTCCTGCCTAACTAT

*L. calcarifer* CATCCGCAAAATGGGGGGAATACACAACCTCACCCCCTTCACTTCCTCTTGTTTAACTCT

*S. maena* TATCCGAAAAATAGGAGGAATACACCGCCTGACCCCTTTTACATCCTCCTGCATAACTAT

*P. auriga* CATTCGAAAAATAGGAGGAATACATCATCTAACCCCCTTTACATCCTCCTGCCTAACCAT

*C. lucidus* TATTCGAAAAATAGGGGGCATGCACCACCTTACACCCTTTACCTCTTCTTGTCTTACTAT

*S. sihama* TATCCGGAAAATAGGAGGCATGCAACACCTCACCCCCTTTACATCCTCCTGTCTTACCTT

*C. loricula* CATCCGAAAAATAGGGGGGATACATCACCTCACCCCTTTTACATCCTCCTGCCTCACAAT

*A. trutta* CATTCGCAAAATAGGGGGCATGCACAACCTCACCCCTATCACCTCCACTTGCTTAATCAT

*H. gemma* TATCCGAAAAATAGGAGGAATACATCATCTTACCCCCTTCACATCTTCTTGCCTTACCAT

*P. flavescens* CATCCGAAAAATAGGAGGTATACACCATCTCACCCCTTTTACATCCTCCTGCTTAACCAT

*P. macrolepida* TATCCGCAAAATAGGAGGCATGCACCACCTCACCCCTTTCACATCCTCCTGCCTGACTGT

*S. canadensis* CATTCGAAAAATAGGAGGTATACATCATCTCACCCCCTTTACATCCTCCTGCTTAACCAT

*A. rogaa* TGGAAGCCTAGCCCTCACGGGCACCCCTTTCCTAGCAGGATTCTTCTCCAAAGATGCTAT

*C. argus* TGGAAGCCTTGCCCTTACAGGCACTCCTTTCCTAGCAGGCTTTTTCTCTAAAGATGCAAT

*C. sonnerati* TGGAAGCCTAGCCCTCACAGGAACACCTTTCCTAGCAGGCTTCTTCTCCAAAGATGCTAT

*E. fuscoguttatus* CGGAAGCCTAGCCCTTACAGGTACCCCTTTCCTAGCAGGCTTCTTTTCTAAAGATGCCAT

*E. coioides* TGGAAGCCTAGCCCTCACAGGCACCCCCTTCCTAGCAGGCTTCTTCTCTAAAGACGCCAT

*E. bruneus* TGGAAGCCTGGCCCTCACAGGCACCCCCTTTCTAGCAGGGTTTTTCTCTAAAGATGCTAT

*E. moara* TGGAAGCCTAGCCCTCACAGGCACCCCTTTCCTAGCAGGATTTTTCTCTAAAGATGCTAT

*E. lanceolatus*  TGGAAGCCTAGCCCTCACAGGCACTCCCTTCCTGGCAGGTTTCTTCTCCAAAGATGCTAT

*A. leucogrammicus*  TGGAAGCCTAGCCCTCACAGGCACCCCCTTTCTGGCAGGCTTTTTCTCCAAAGATGCCAT

*C. altivelis* TGGTAGCCTAGCCCTTACAGGCACCCCCTTTCTAGCAGGCTTTTTCTCCAAAGATGCCAT

*E. epistictus* TGGAAGCCTGGCCCTCACAGGCACCCCCTTCCTAGCGGGCTTCTTCTCTAAAGATGCTAT

*E. octofasciatus* TGGAAGTCTAGCCCTCACTGGCACCCCTTTCCTAGCAGGCTTCTTCTCCAAAGATGCTAT

*E. septemfasciatus* TGGAAGCCTAGCCCTCACTGGCACCCCCTTCCTAGCGGGCTTCTTCTCTAAAGATGCTAT

*T. dermopterus* CGGGAGCCTAGCCCTCACTGGCACCCCCTTCCTAGCGGGCTTTTTCTCTAAAGATGCCAT

*E. awoara* TGGAAGCCTAGCCCTCACGGGCACCCCTTTCTTAGCCGGCTTTTTCTCCAAAGATGCTAT

*E. akaara* CGGAAGCCTAGCCCTCACGGGCACCCCTTTCCTAGCCGGCTTCTTCTCTAAAGATGCTAT

*E. trimaculatus* CGGAAGCCTTGCCCTCACAGGCACCCCTTTCCTAGCCGGCTTCTTCTCTAAAGACGCTAT

*E. areolatus* TGGAAGCCTTGCCCTTACAGGTACCCCCTTCCTAGCCGGCTTCTTCTCTAAAGATGCCAT

*V. albimarginata* TGGCAGTCTTGCCCTCACAGGCACCCCCTTCTTAGCCGGCTTCTTCTCCAAAGACGCCAT

*V. louti* TGGCAGTCTCGCCCTCACAGGTACCCCATTTTTGGCCGGCTTCTTCTCCAAAGACGCGAT

*P. leopardus* CGGAAGCCTAGCCCTGACAGGAACTCCTTTCCTAGCAGGGTTTTTCTCAAAAGATGCTAT

*P. areolatus* TGGAAGCCTGGCCCTAACAGGAACCCCTTTCTTAGCAGGGTTTTTTTCAAAAGATGCTAT

*E. radiosum* CGGAAGTCTCGCCCTTACAGGAACCCCCTTCTTAGCGGGCTTTTTTTCAAAAGACGCCAT

*P. sieboldi* CGGCAGCCTCGCCCTCACGGGCACCCCGTTCCTCGCGGGCTTCTTCTCTAAAGACGCAAT

*E. armatus* TGGGAGCCTTGCCCTCACGGGCACTCCCTTCCTAGCAGGCTTCTTTTCTAAAGATGCCAT

*R. oxyrhynchus* TGGCAGTTTAGCCCTCACAGGAACCCCTTTCCTTGCGGGCTTTTTCTCCAAAGACGCCAT

*K. cinerascens* CGGCAGCCTAGCCCTCACAGGCACCCCATTCCTTGCAGGCTTCTTCTCCAAAGACGCCAT

*T. chatareus* CGGCAGCCTAGCCCTTACAGGCACTCCCTTTCTAGCAGGCTTCTTCTCTAAAGACGCCAT

*D. berycoides* CGGCAGCCTAGCCCTAACGGGCACTCCTTTCCTGGCGGGATTCTTCTCCAAAGACGCCAT

*H. typus* TGGCAGCCTTGCCCTCACCGGCACTCCCTTCCTAGCAGGGTTCTTTTCTAAAGATGCCAT

*M. argenteus* CGGCAGCCTAGCTCTCACAGGCACTCCCTTTCTAGCAGGCTTTTTCTCTAAAGATGCCAT

*S. chuatsi* TGGCAGTCTAGCCCTAACAGGTACCCCCTTTTTAGCAGGCTTCTTCTCCAAAGACGCCAT

*O. fasciatus* TGGTAGCTTAGCCCTTACAGGCACCCCCTTCCTAGCAGGCTTTTTCTCTAAAGATGCCAT

*P. trilineatum* TGGCAGCCTTGCACTTACAGGTACCCCTTTCCTAGCAGGCTTCTTCTCTAAGGATGCCAT

*M. salmoides* CGGAAGCCTGGCCTTAACCGGCACCCCCTTCCTAGCAGGCTTCTTCTCTAAAGACGCAAT

*P. tile* TGGCAGTCTTGCCCTTCCACGCACCCCCTTCCTCGCAGGTTTCTTCTCCAAAGATGCAAT

*L. argentimaculatus* TGGCAGCCTCGCCCTCACAGGCACCCCCTTCTTAGCAGGCTTCTTCTCTAAAGACGCCAT

*E. struhsakeri* TGGTAGTTTAGCCCTCACAGGCACCCCCTTCCTAGCAGGATTCTTCTCCAAAGACGCCAT

*B. albus* TGGCAGCCTAGCCCTCACCGGCACCCCTTTCCTAGCAGGATTCTTCTCCAAAGATGCTAT

*C. auripes* CGGCAGTCTCGCCCTAACAGGCACTCCCTTCCTTGCCGGCTTCTTTTCTAAAGACGCCAT

*C. melampygus* TGGCAGCCTAGCCCTCACCGGTACCCCCTTCCTTGCAGGCTTCTTCTCAAAAGACGCCAT

*L. calcarifer* CGGCAGTCTCGCCCTTACCGGAACCCCCTTCCTTGCAGGCTTCTTCTCAAAAGATGCCAT

*S. maena* CGGAAGCTTAGCTCTTACAGGTACTCCCCTCCTCGCAGGCTTCTTTTCCAAAGATGCCAT

*P. auriga* TGGAAGCCTGGCCCTTACAGGTACGCCCTTCCTAGCAGGATTCTTCTCTAAAGATGCCAT

*C. lucidus* TGGAAGCCTAGCCCTCACTGGTACCCCTTTTTTAGCAGGCTTCTTCTCAAAAGACGCTAT

*S. sihama* GGGCAGCCTGGCCCTCACGGGAACCCCCTTCCTTGCCGGGTTCTTTTCGAAGGACGCCAT

*C. loricula* CGGCAGCCTCGCCCTCACAGGGACACCCTTTTTAGCTGGCTTCTTCTCAAAAGACGCCAT

*A. trutta* TGGAAGCCTTGCTCTTACGGGCACCCCCTTCCTCTCAGGCTTTTTCTCAAAAGATGCAAT

*H. gemma* TGGGAGTTTAGCCCTAACGGGCACTCCTTTCTTGGCCGGGTTCTTCTCCAAAGACGCTAT

*P. flavescens* TGGAAGCCTTGCCCTCACAGGCACCCCTTTCTTAGCAGGCTTTTTCTCTAAAGATGCTAT

*P. macrolepida* TGGAAGTCTCGCCCTAACAGGCACTCCATTTTTAGCAGGTTTCTTCTCTAAAGACGCAAT

*S. canadensis* TGGGAGCCTCGCCCTCACAGGCACCCCCTTCTTAGCAGGCTTCTTCTCTAAAGACGCCAT

*A. rogaa* TATTGAAGCCCTCAACACCTCGAACCTAAACGCCTGAGCCCTCACCCTCACTCTCCTTGC

*C. argus* TATTGAAGCCTTAAACACCTCGAACCTAAACGCCTGAGCCCTAACCCTCACTCTCCTTGC

*C. sonnerati* CATTGAAGCCCTCAACACCTCAAACCTAAACGCCTGAGCCCTTACCCTGACCCTCCTTGC

*E. fuscoguttatus* CATTGAAGCTCTCAACACCTCATACCTAAACGCCTGAGCCCTTACCTTGACCCTCCTAGC

*E. coioides* CATTGAAGCCCTCAACACCTCACACCTAAACGCCTGGGCCCTTACCTTGACTCTCCTAGC

*E. bruneus* TATTGAAGCCCTCAACACCTCATACCTAAACGCCTGAGCCCTTACCTTGACCCTCCTAGC

*E. moara* TATTGAAGCCCTCAACACCTCATACCTAAACGCCTGAGCCCTTACCTTGACCCTCCTAGC

*E. lanceolatus*  CATTGAAGCCCTCAACACCTCATACCTAAACGCCTGAGCCCTTACCTTAACTCTCCTAGC

*A. leucogrammicus*  CATCGAAGCCCTCAACACCTCATACCTAAACGCCTGAGCCCTTACCCTGACTCTCTTAGC

*C. altivelis* CATTGAAGCCCTTAATACCTCATATCTGAACGCCTGAGCCCTTACCCTAACTCTTCTAGC

*E. epistictus* TATTGAAGCCCTTAACACCTCATACCTAAACGCCTGAGCCCTTACCTTAACTCTCCTGGC

*E. octofasciatus* CATTGAAGCCCTTAACACCTCATACCTAAACGCCTGGGCCCTTACCTTGACTCTCCTAGC

*E. septemfasciatus* CATTGAAGCCCTTAACACATCCCACCTAAACGCCTGAGCCCTTACCTTGACTCTCCTAGC

*T. dermopterus* CATTGAAGCCCTTAATACCTCATACCTAAACGCCTGGGCCCTTACCTTGACTCTCCTAGC

*E. awoara* TATTGAAGCCCTCAACACCTCATATCTTAACGCCTGAGCCCTTACCTTGACCCTTCTAGC

*E. akaara* TATTGAAGCCCTCAACACCTCATATCTTAACGCCTGAGCCCTTACCTTAACCCTCCTAGC

*E. trimaculatus* CATTGAAGCCCTTAACACCTCATACCTCAACGCCTGGGCCCTTACCTTAACTCTCCTTGC

*E. areolatus* CATTGAAGCTCTCAACACCTCATACCTTAACGCCTGGGCCCTTACCTTAACCCTTCTAGC

*V. albimarginata* CATTGAAGCATTAAACACATCATACCTTAACGCCTGGGCCCTCATCCTCACTCTCCTCGC

*V. louti* CATCGAAGCACTAAACACATCATACCTTAACGCCTGGGCCCTCATCCTCACACTCCTCGC

*P. leopardus* TATTGAAGCATTAAACACTTCTTACCTAAACGCCTGGGCCCTAATCCTGACTTTATTAGC

*P. areolatus* TATTGAAGCACTAAACACTTCTTACCCTAACGCCTGAGCCCTAATCCTGACTTTATTAGC

*E. radiosum* TATTGAAGCATTAAACACATCCCATCTAAACGCCTGAGCCCTAGTACTAACCCTGCTGGC

*P. sieboldi* CATTGAAGCCCTCAACACCTCCTACCTAAACGCCTGAGCCCTCACCCTAACCCTTCTGGC

*E. armatus* CATTGAAGCACTAAACACATCCCACCTTAACGCCTGAGCCCTTACCTTAACCCTCTTAGC

*R. oxyrhynchus* TATTGAAGCACTAAATACATCTCACCTCAACGCCTGAGCCCTCACTCTGACTCTTATTGC

*K. cinerascens* CATTGAAGCACTAAACACATCCCATCTTAACGCCTGAGCCCTTACCCTCACCCTTCTAGC

*T. chatareus* CATCGAAGCACTAAACACATCTTACCTAAACGCCTGAGCCCTAACCCTTACACTCCTAGC

*D. berycoides* TATTGAAGCCCTCAACACATCCTACCTTAACGCCTGGGCCCTTACCTTAACCCTCCTAGC

*H. typus* TATTGAAGCCCTCAACACATCCCACCTTAACGCCTGAGCCCTTACCCTAACCCTTCTAGC

*M. argenteus* CATTGAAGCACTAAACACATCCCACCTTAACGCCTGAGCCCTAACTCTGACTCTCCTGGC

*S. chuatsi* CATTGAATCACTAAACACATCACACCTTAACGCCTGAGCCCTGACCTTAACCATTTTAGC

*O. fasciatus* TATTGAAGCACTAAACACATCCCATCTTAACGCCTGAGCCCTTGCCCTAACCCTCTTAGC

*P. trilineatum* CATTGAAGCACTCAACACATCCCATCTCAACGCCTGAGCCCTAACTCTCACCCTCCTAGC

*M. salmoides* TATCGAAGCACTTAACACATCACACCTAAACGCCTGAGCCCTTGCCCTAACCCTCCTGGC

*P. tile* TATCGAGGCACTCAACACATCCCATCTAAACGCCTGAGCCCTAACCCTTACACTCCTAGC

*L. argentimaculatus* CATCGAAGCCCTAAACACATCTTATCTCAACGCCTGAGCCCTCGCCCTCACACTTTTAGC

*E. struhsakeri* CATTGAAGCACTAAACACATCCCACCTTAACGCCTGAGCCCTAACCCTGACCCTTCTGGC

*B. albus* TATTGAAGCACTAAACAACTCCTACCTTAACGCCTGAGCCCTCTCCCTTACTTTACTAGC

*C. auripes* CATTGAGGCTTTAAACACATCTTACCTAAACGCCTGAGCCCTAGCCTTGACACTCGTAGC

*C. melampygus* TATCGAAGCACTAAACACCTCCCACCTTAACGCCTGAGCCCTAGTCCTGACCCTCCTAGC

*L. calcarifer* TATCGAAGCACTAAACACATCCCATCTTAACGCCTGAGCCCTGATCCTGACCCTCCTAGC

*S. maena* TATTGAAGCCCTAAACAACTCCTACCTAAACGCCTGAGCCCTAACCATGACCCTCCTAGC

*P. auriga* TATCGAAGCCCTAAATAACTCTTACCTAAACGCCTGAGCCCTAGCCATAACCCTCCTGGC

*C. lucidus* TATTGAAGCACTTAACACATCCCATCTCAACGCCTGAGCCCTTGCCCTCACTCTCCTAGC

*S. sihama* TATCGAAGCCCTCAATACCTCTCACCTTAACGCCTGAGCCGTGGCCTCTATACTACTAGT

*C. loricula* CATTGAGGCACTAAACACCTCACACCTTAACGCCTGAGCCCTTGTCCTGACCCTCTTAGC

*A. trutta* CATTGAAGCCCTTAATACATCAACTGTTAATGCCTGAGCCCTGGCACTCACCCTGCTTGC

*H. gemma* CATTGAAGCCCTAAACACCTCTTACCTAAACGCCTGAGCCCTAATCCTCACCTTACTAGC

*P. flavescens* TATTGAAGCATTAAACACATCCCATTTAAACGCCTGAGCCCTTGTCTTAACCCTTTTAGC

*P. macrolepida* CATCGAAGCACTAAACACATCCCACCTAAACGCCTGAGCCCTAGTCTTGACCCTTCTCGC

*S. canadensis* TATTGAAGCATTAAACACATCCCACCTAAACGCCTGGGCCCTAGTTCTGACCCTACTAGC

*A. rogaa* CACCTCTTTTACGGCTATTTACAGCTTACGTGTTATCTTCTTTGTCTCAATGGGACACCC

*C. argus* CACCTCCTTTACAGCTATCTACAGCTTGCGCGTAATCTTCTTTGTCTCAATAGGACACCC

*C. sonnerati* CACCTCTTTTACGGCCATCTACAGCTTACGCGTAGTCTTCTTCGTATCAATAGGACACCC

*E. fuscoguttatus* CACCTCCTTCACAGCTATTTACAGCTTACGCATTGTTTTTTTTGTCTCAATAGGACGCCC

*E. coioides* CACCTCCTTCACAGCTATTTACAGCTTACGCGTCATTTTCTTTGTCTCAATAGGACGCCC

*E. bruneus* CACCTCTTTCACAGCTATCTACAGCCTACGCATTATTTTCTTCGTCTCAATAGGACGCCC

*E. moara* CACCTCTTTCACGGCTATCTACAGCCTACGCATCATTTTCTTCGTCTCAATAGGACGCCC

*E. lanceolatus*  CACCTCCTTCACAGCCATTTATAGTTTACGCATTGTTTTCTTCGTCTCAATAGGACATCC

*A. leucogrammicus*  CACCTCTTTCACAGCCATTTATAGCTTACGTATTGTTTTCTTCGTCTCAATAGGACACCC

*C. altivelis* CACCTCCTTCACAGCTATTTACAGCTTACGCATCATTTTTTTTGTCTCTATAGGACACCC

*E. epistictus* CACTTCCTTCACAGCTATTTATAGCTTACGTGTTATTTACTTCGTTTCAATAGGACACCC

*E. octofasciatus* CACCTCCTTCACAGCTATTTATAGCCTACGTGTTATTTACTTCGTTTCAATAGGACACCC

*E. septemfasciatus* CACCTCCTTCACAGCTATTTATAGCCTACGTGTTATTTACTTCGTTTCAATAGGACACCC

*T. dermopterus* CACCTCCTTCACAGCTATCTACAGCTTACGTGTCATCTACTTCGTTTCAATAGGACACCC

*E. awoara* CACCTCCTTCACAGCAATCTACAGTCTACGCGTCATTTACTTCGTCTCAATGGGTAACCC

*E. akaara* CACCTCCTTCACAGCAATCTACAGCCTACGCGTTATTTATTTCGTCTCGATGGGTAACCC

*E. trimaculatus* TACCTCCTTCACAGCAATCTACAGCTTACGTATTGTCTTCTTTGTCTCAATAGGTCACCC

*E. areolatus* CACCTCCTTCACAGCAATTTACAGCTTGCGCATCGTCTACTTCGTCTCAATAGGCCGCCC

*V. albimarginata* CACCTCTTTTACAGCAATTTACAGCTTACGCGTAGTTTTCTTTGTCTCCATGGGCAACCC

*V. louti* CACCTCTTTCACAGCAATCTACAGCTTACGCGTAGTTTTCTTCGTTTCCATGGGCAACCC

*P. leopardus* CACCTCATTTACAGCTATTTACAGCTTGCGTGTTATCTTCTTTGTTTCAATAGGTCACCC

*P. areolatus* CACCTCATTTACAGCTATTTATAGCTTACGTATTGTCTTCTTTGTCTCAATGGGTTACCC

*E. radiosum* CACCTCCTTTACGGCCATCTACAGCCTCCGAGTTGTATATTTTGTATCAATGGGGCACCC

*P. sieboldi* CACGTCCTTTACAGCTGTCTACAGCCTACGCCTTGTTTATTTTGTCGCAATGGGCCACCC

*E. armatus* CACCTCCTTCACAGCCATTTATAGTCTCCGAGTTGTCTTCTTTGTTTCCATGGGGCACCC

*R. oxyrhynchus* CACCTCTTTCACAGCAATCTACAGCCTCCGAGTCGTCTTCTTTGTCTCCATAGGTTATCC

*K. cinerascens* CACCTCTTTCACAGCCATCTACAGCCTCCGAGTTGTCTTCTTCGTGTCAATAGGCCACCC

*T. chatareus* CACCTCTTTCACCGCCATTTACAGCCTCCGCGTTGTATACTTCGTAGCCATAGGATTCCC

*D. berycoides* TACCTCATTTACGGCCATCTATAGCCTCCGAATCATCTTCTTTGTATCCATAGGCCACCC

*H. typus* CACCTCATTTACGGCCATCTACAGCCTCCGAGCTGTATTCTTCGTAGCTATGGGCCACCC

*M. argenteus* CACCTCTTTCACAGCTATCTACAGCCTCCGAGTCGTCTTCTTTGTTTCCATAGGACACCC

*S. chuatsi* CACCTCTTTTACAGCCATTTACAGCCTCCGAGTGGTGTACTTCGTCTCCATGGGCCACCC

*O. fasciatus* CACCTCCTTCACAGCCATCTACAGCCTACGAGTTGTTTTCTTTGTGTCAATAGGCCACCC

*P. trilineatum* CACCTCTTTCACAGCTATTTACAGCCTCCGGGTTGTCTTCTTCGTTTCTATAGGCCACCC

*M. salmoides* CACCTCCTTCACTGCAATTTACAGCCTTCGCGTTGTATTCTTTGTGTCCATAGGACACCC

*P. tile* CACCTCATTTACAGCTATCTACAGCCTCCGAGTTGTTTTCTTCGTTGCTATAGGCCACCC

*L. argentimaculatus* CACCTCATTCACAGCTATCTACAGCCTCCGCGTAGTCTTCTTTGTCGCCATAGGCCACCC

*E. struhsakeri* CACCTCCTTCACAGCTATTTACAGCCTTCGTGTTATTTTCTTCGTAGCTATAGGCCACCC

*B. albus* CACTTCATTCACAGCCGTGTATAGCCTTCGTCTCGTATTTTTTGTTGTTATAGGCCACCC

*C. auripes* CACTTCTTTCACAGCCATTTACAGCCTCCGTGTCATCTTCTTTGTCTCCATAGGTCACCC

*C. melampygus* AACCTCATTCACCGCTATCTACAGCATACGAATCGTCTTCTTTGTAGTCATGGGTCACCC

*L. calcarifer* AACCTCTTTCACCGCTATTTACAGCATACGTGTTGTCTTCTTTGTAGTTATGGGGCACCC

*S. maena* CACCTCATTCACGGCTATCTACAGCTTACGAGTAGTTTTTTTCGTCTCTATGGGGCACCC

*P. auriga* CACCTCCTTCACTGCCATTTACAGCCTGCGGGTGGTTTTTTTTGTTTCCATAGGTCACCC

*C. lucidus* TACCTCCTTTACAGCCATTTATAGCCTCCGAGTCGTTTTCTTTGTATCCATAGGCCACCC

*S. sihama* CACATCCTTCACCGCGGTCTACAGCCTCCGAGTGGTCTTTTTCGTCTCCATAGGCCACCC

*C. loricula* CACTTCCTTCACGGCCGTCTACAGCTTACGCGTAATCTTCTTTGTATCAATAGGCCACCC

*A. trutta* CACTTCATTTACAGCTGTTTACAGCCTCCGACTCATTTACTTCGTGTCGATAGGCTTCCC

*H. gemma* TACATCATTTACAGCCATCTATAGCCTGCGGGTAATTTTCTTTGTATCTCTTGGACACCC

*P. flavescens* TACCTCCTTCACAGCCATCTACAGTCTCCGTGTTATTTATTTTGTTTCCATAGGCCACCC

*P. macrolepida* CACTTCTTTCACTGCTATTTACAGTCTCCGGGTCGTGTATTTTGTGTCTATAGGACACCC

*S. canadensis* TACCTCTTTTACCGCCATCTACAGTCTCCGTGTCATCTACTTTGTTACTATGGGCCACCC

*A. rogaa* TCGATTCAACGCACTCTCACCTATCAATGAAAACAACCGAGCAGTCATTAACCCTATTAA

*C. argus* CCGATTTAACGCACTTTCACCTATTAATGAAAACAACCAAGCAGTCATTAACCCTATCAA

*C. sonnerati* CCGATTTAATGCATTCTCACCCATCAACGAAAATAACCGCACAGTCATTAACCCCATCAA

*E. fuscoguttatus* TCGATTTAATGCACTTTCCCCTATTAACGAAAACAACCCTGCAGTTATTAACCCAATTAA

*E. coioides* CCGGTTTAATGCACTTTCTCCCATCAACGAAAACAACCCTGCAGTCATTAACCCTATCAA

*E. bruneus* CCGATTTAATGCACTCTCCCCCATCAACGAAAACAACCCCGCAGTAATCAACCCCCTTAA

*E. moara* CCGATTTAATGCACTCTCCCCTATCAACGAAAACAACCCCGCAGTTATCAACCCCCTTAA

*E. lanceolatus*  CCGGTTTAATGCACTCTCCCCCATTAACGAGAACAACCCCGCAGTCATTAACCCTATCAA

*A. leucogrammicus*  CCGATTCAACGCATTTTCCCCTATCAACGAAAACAACCCAGCAGTTATCAACCCTATTAA

*C. altivelis* CCGATTCAATACACTTTCTCCCATCAACGAAAACAACCCTGCAGTCATTAATCCTATTAA

*E. epistictus* CCGATTCAACGTACTCTCCCCCATCAACGAAAATAACCCTGCAGTCCTCAACCCTATCAA

*E. octofasciatus* CCGATTTAATGCACTCTCCCCCATCAACGAAAATAATCCTGCAGTCCTAAACCCCATTAA

*E. septemfasciatus* CCGATTTAATGCACTCTCCCCCATCAACGAAAATAACCCTGCAGTCCTAAACCCCATTAA

*T. dermopterus* CCGGTTTAATGCACTTTCCCCCATTAACGAAAACAACCCCGCAGTCCTCAACCCCATTAA

*E. awoara* CCGCTTTAATTCACTTTCCCCTATCAACGAAAACAACCCCGCAGTTCTTAACCCAATCAA

*E. akaara* CCGCTTTAATTCACTTTCCCCTATCAACGAAAACAACCCTGCAGTCCTTAATCCCATCAA

*E. trimaculatus* CCGCTTCAACACACTCTCTCCCATCAACGAAAATAATCCTGCAGTCCTAAACCCTATCAA

*E. areolatus* CCGCTTTAATGCACTCTCCCCAATCAACGAAAACAACCCCGCAGTTCTAAACCCTATCAA

*V. albimarginata* CCGATTTAACCCTCTTTCACCAATCAACGAAAACAACCCTGCAGTCATCAAACCCATTAA

*V. louti* CCGATTTAACCCTCTTTCACCCATCAACGAGAACAACCCTGCAGTCCTCAACCCCATTAA

*P. leopardus* CCGATTTAATTCTTTATTACCTATCAATGAAAACAACCCCGCAGTGATTAACCCTATTAA

*P. areolatus* CCGATTTAATCCTCTATCACCTATCAACGAAAATAACCCTGCAGTAATCAACCCTATCAA

*E. radiosum* TCGCTTCAACCCACTCTCTCCTATTAATGAAAACAACCCGGCGGTAATTAACCCAATCAA

*P. sieboldi* TCGGTTTAATGCCCTCTCTCCCATCAACGAGAATAACCCCGCTGTCATCAACCCTATTAA

*E. armatus* TCGATTCAACTCCCTTTCCCCAATTAATGAAAACAATCCAGCGGTGATTAACCCAATCAA

*R. oxyrhynchus* CCGATTCAACTCTCTTTCCCCCATCAACGAAAACAACCCAGCAGTAATTAACCCAATTAA

*K. cinerascens* TCGATTCAACTCACTCTCCCCCATCAACGAAAACAACCCAGCAGTTATTAACCCAATCAA

*T. chatareus* TCGATTCAACACACTCTCACCTATTAATGAAAACAATCCCACAGTAATTAACCCCATCAA

*D. berycoides* CCGATTTAATACCCTCTCCCCTATTAACGAAAACAACCCAGCAGTTATTAACCCAATCAA

*H. typus* CCGATTTAATTCGCTATCCCCTATCAACGAAAACAACCCAGCAGTCCTCCAGCCCATTAA

*M. argenteus* CCGATTTAACTCACTATCACCCATTAACGAAAACAACACAGCCGTTATTAATCCCATCAA

*S. chuatsi* CCGATTCAGCCCCATATCCCCTATTAACGAAAATAACCCAGCAGTAATCAACCCAATCAA

*O. fasciatus* CCGATTCAACCCTCTTTCCCCTATCAACGAAAACAACCCAGCCGTAATTAACCCAATCAA

*P. trilineatum* CCGATTTAACTCACTATCCCCTATTAATGAGAACAACCCAGCAGTTATTAACCCCATTAA

*M. salmoides* TCGATTTAATACACTCTCCCCCATCAATGAAAATAACCCAGCAGTAATTAACCCAATTAA

*P. tile* CCGATTCAACTCACTCTCCCCTATTAACGAAAACAACCCGGCAGTCATCAACCCCATCAA

*L. argentimaculatus* CCGATTCAACTCACTCTCCCCAATTAATGAAAACAACCCTGCAGTCATCAACCCCATTAA

*E. struhsakeri* CCGATTTAACTCACTATCCCCTATCAACGAAAACAACCCGGCAGTCATTAACCCTATCAA

*B. albus* TCGCTTTAACCCCCTGTCACCAATTAACGAAAACTCGCCCTCCGTCATCAATCCCCTAAA

*C. auripes* TCGATTCAACCCACTATCCCCCATTAACGAAAATGACCCAAAAGTGATCAACCCTATTAA

*C. melampygus* CCGATTTAATGCACTTTCCCCTATTAACGAAAACAATCCAGCAGTGATTAACCCTATTAA

*L. calcarifer* CCGATTTAACTCCCTCTCCCCAATTAACGAAAACAACCCAGCAGTAATCAATCCCATCAA

*S. maena* CCGATTCCCTTCACTTTCCCCCATTAATGAAAACAACCCGACAGTGATTAATCCGATCAA

*P. auriga* CCGATTCCAACCCCTTTCTCCCATTAATGAAAACAACCCGGCAGTAATCAACCCTATCAA

*C. lucidus* CCGATTCAACACACTCTCCCCCATCAATGAAAACAACCCAGCAGTCTTAAACCCCATCAA

*S. sihama* CCGCTTTAACCCTCTCTCACCCATCAACGAGAACAACCCCGCAGTAATTAACCCCATTAA

*C. loricula* CCGATTTAAGTCCCTTTCCCCCATTAACGAAAACGACCCCGCCGTTATTAACCCAATAAA

*A. trutta* ACGATTCAACCCTCTCTCCCCTATCAACGAAAATAACCCCCTAGTGATTAACCCCCTAAA

*H. gemma* CCGTTTTAACTCTCTTTCACCTATTAATGAGAATAACCCTTCAGTAATTAATCCTATTAA

*P. flavescens* CCGCTTTAATTCACTTCCTCCTATTAATGAAAACAACCCAGCAGTTATTAACCCCATTAA

*P. macrolepida* TCGTTTCAACCCCCTTTCCCCAATTAATGAAAACAACCCAGCAGTAATCAACCCAATCAA

*S. canadensis* CCGTTTTAATTCTCTATCCCCCATTAATGAAAATAACCCAGCAGTAATTAATTCCCTTAA

*A. rogaa* ACGACTAGCCTGAGGCAGCATTGTTGCCGGTCTCTTAATTACTACCAACCTCCTCCCG--

*C. argus* ACGACTAGCTTGAGGCAGCATTGTTGCCGGTCTATTAATTACCACTAACCTTCTCCCA--

*C. sonnerati* ACGACTAGCTTGAGGAAGCATTATCGCCGGTCTTCTAATCACAACCAACGTACTCCCA--

*E. fuscoguttatus* ACGACTAGCTTGAGGAAGCATTGTAGCTGGCCTTCTAATTACCTCCAACCTACTTCCA--

*E. coioides* ACGACTAGCCTGAGGAAGCATTGTAGCTGGCCTCCTAATCACCTCCAACCTACTTCCA--

*E. bruneus* ACGACTAGCTTGAGGAAGCATTGTAGCCGGCCTCCTAATTACTTCCAACCTACTCCCA--

*E. moara* ACGACTAGCTTGAGGAAGCATTGTAGCCGGCCTCCTAATTACCTCCAACCTACTCCCA--

*E. lanceolatus*  ACGATTAGCCTGAGGAAGCATTGTAGCCGGCCTATTAATTACCTCCAACCTACTTCCA--

*A. leucogrammicus*  ACGACTAGCCTGAGGAAGCATTGTAGCTGGCTTTCTAATCACTTCCAACCTAATTCCA--

*C. altivelis* ACGACTAGCCTGAGGAAGTATTGTAGCCGGCCTCCTGATCACTTCCAACCTACTTCCA--

*E. epistictus* ACGATTAGCTTGAGGGAGCATTGTAGCCGGCCTCCTACTTACCTCCAACCTTCTCCCA--

*E. octofasciatus* ACGACTAGCTTGGGGAAGCATTGTTGCCGGTCTCTTGCTCACTTCCAATCTTCTCCCA--

*E. septemfasciatus* ACGACTAGCTTGGGGAAGCATTGTTGCCGGCCTCTTACTCACTTCCAACCTTCTCCCA--

*T. dermopterus* ACGACTAGCTTGAGGGAGCATTGTAGCCGGCCTCTTGCTCACTTCCAACCTACTCCCA--

*E. awoara* GCGTCTAGCTTGAGGAAGCATTGTAGCTGGTCTTCTAATTACCTCTAACCTAATCCCA--

*E. akaara* GCGCCTAGCTTGAGGAAGCATTGTAGCTGGCCTTCTAATTACCTCTAACCTAGTCCCA--

*E. trimaculatus* ACGCCTAGCCTGAGGAAGCATTGTAGCCGGCCTCCTAATTACATCTAACTTAATCCCC--

*E. areolatus* ACGCCTAGCCTGAGGAAGCATTGTCGCCGGCCTCCTAATTACGTCAAACCTACTCCCA--

*V. albimarginata* ACGACTAGCATGAGGTAGCATCATCGCAGGCCTTGTAATTACATCAAACATCACTCCT--

*V. louti* GCGACTAGCATGAGGTAGCATCATCGCAGGCCTTGTAATCACCTCAAACATCACCCCT--

*P. leopardus* ACGACTAGCATGAGGCAGTATTATTGCAGGCCTCCTGATTACATCCAATATCTTGCCG--

*P. areolatus* ACGACTAGCGTGAGGCAGCATTATCGCAGGCCTCCTGATCACATCAAACATCTTGCCG--

*E. radiosum* GCGGCTAGCCTGGGGCAGTGTTATCGCCGGTCTTCTAATTACCTCAAATATTACGCCC--

*P. sieboldi* ACGGCTAGCCTGAGGCAGCATCGTCGCTGGGCTAATTCTAACCTCCAACATCCTCCCC--

*E. armatus* GCGACTGGCCTGAGGAAGCATCCTTGCCGGCCTTTTAATTACATCCAACATCACTCCT--

*R. oxyrhynchus* ACGACTGGCCTGAGGCAGCATTATCGCCGGGCTCCTGATTACTTCTCATATCTCCCCA--

*K. cinerascens* ACGACTAGCCTGAGGAAGCATTATCGCCGGCTTACTAATCACCTCTAACATCCTCCCC--

*T. chatareus* ACGACTAGCATGAGGAAGCATCATCGCCGGCCTCCTAATTACCTCAAACATTACGCCC--

*D. berycoides* ACGACTTGCCTGAGGAAGCATCATTGCCGGACTCCTAATCACCTCAAATATTATCCCC--

*H. typus* ACGACTGGCCTGAGGAAGTATCATTGCCGGCCTCCTAATTACCTCAAATATCATTCCT--

*M. argenteus* ACGACTAGCCTGAGGAAGCATTATCGCTGGCCTCCTAATTACTGCAAATATTATGCCT--

*S. chuatsi* ACGACTAGCCTGAGGTAGCATCGTCGCCGGTCTCCTAATTACCTCAAACATTATCCCT--

*O. fasciatus* ACGACTGGCCTGAGGCAGTATTGTCGCCGGCCTATTAATTACCTCTAACATCACCCTA--

*P. trilineatum* ACGCTTAGCCTGAGGAAGCATTATTGCCGGCCTCTTAATTACATCCAACATCTTGCCC--

*M. salmoides* ACGACTTGCCTGAGGCAGCATCATCGCCGGACTCATTATTACTTCCAATATTACTCCT--

*P. tile* ACGACTAGCCTGAGGAAGCATTGTCGCCGGACTCCTAATCACCTCAAACATTCTCCCT--

*L. argentimaculatus* ACGATTAGCTTGAGGAAGCATTGTCGCCGGATTACTTATCACCTCCAACATCCTCCCC--

*E. struhsakeri* ACGACTTGCCTGAGGAAGCATTATCGCCGGCCTCTTGATTACCTCAAATATTATTCCC--

*B. albus* ACGACTAGCCTGAGGAAGTATTATTGCAGGCCTCTTAATCACTTCAAACATTACTCCC--

*C. auripes* ACGACTGGCCTGAGGAAGCATTATCGCTGGCCTCTTAATTACTTCCCACATTGTCCCT--

*C. melampygus* ACGACTAGCCTGAGGGAGCATTATCGCTGGCCTTCTAATTACATCAAACATTCTTCCT--

*L. calcarifer* ACGACTTGCTTGAGGAAGCATTGTTGCTGGCCTTCTAATTACATCCAACATTATACCCAA

*S. maena* ACGACTTGCCTGAGGAAGTATCATTGCAGGCCTACTAATTACCTCAAACATTATTCCA--

*P. auriga* ACGCCTAGCCTGAGGAAGTATCATTGCAGGCCTCCTGATCACTTCCAATATTATTCCC--

*C. lucidus* ACGCCTAGCCTGAGGAAGCATTATTGCCGGCCTTTTAATTACCTCTAATATAACCCCC--

*S. sihama* GCGTTTGGCTTGAGGAAGTATTCTTGCAGGACTTATTCTCACCTCAAACATTACCCCC--

*C. loricula* ACGGCTGGTCGGAGGTAGCATCGTCGCCGGTTTCATTATTATCTCAAATACACTCCCC--

*A. trutta* GCGACTAGCCTGAGGGAGCATTATTGCTGGTCTCTTTATTACCCTAACCATTGCCCCC--

*H. gemma* ACGCCTAGCTTGAGGGAGTATTATTGCCGGCCTTATCATTACATCAAATCTTCTGCCC--

*P. flavescens* ACGACTAGCCTGAGGAAGCATTATTGCCGGTCTCCTAATTACTTCAAATATTTTTCCC--

*P. macrolepida* ACGGCTAGCCTGAGGCAGCATCTTCGCCGGTCTTCTAATTACCTCTAACCTCATTCCA--

*S. canadensis* ACGATTAGCCTGGGGAAGCGTAATTGCCGGCCTACTAATCACCTCTAATATTCTTCCC--

*A. rogaa* -CTA---------AAAACACCAATTATATCTATACCACCCCTCCTTAAACTTGCCGCCCT

*C. argus* -TTA---------AAAACACCAATTATATCTATACCGCCTCTCCTAAAACTAGCCGCCCT

*C. sonnerati* -CTA---------AAAACACCAGTTATATCAATACCTCCCCTACTAAAACTTGCCGCCCT

*E. fuscoguttatus* -CTA---------AAAACCCCCGTAATATCAATACCGCTAATGCTCAAACTAACCGCTCT

*E. coioides* -CTA---------AAAACACCAGTAATATCAATACCCCTTATGCTCAAACTAACCGCTTT

*E. bruneus* -CTA---------AAAACGCCAGTAATATCAATACCCCCCATACTTAAACTAACCGCTTT

*E. moara* -CTA---------AAAACGCCAGTAATATCAATACCCCTCATACTTAAACTAACCGCTTT

*E. lanceolatus*  -CTA---------AAAACACCTATCATATCTATACCCCCTATACTCAAACTAACCGCCTT

*A. leucogrammicus*  -CTA---------AAAACACCAGTAATATCAATACCCCTATTACTTAAACTAGCCGCTTT
[truncated: 329,739 more chars]
