# Supplementary material for: A Comprehensive Description and Evolutionary Analysis of 22 Grouper (Perciformes, Epinephelidae) Mitochondrial Genomes with Emphasis on Two Novel Genome Organizations
Source: PLoS One. 2013 Aug 9;8(8):e73561. doi: 10.1371/journal.pone.0073561 (PMC3739747; doi:10.1371/journal.pone.0073561)
Supplement: Table S1 — GenBank accession numbers for mitochondrial genome sequences of the Percoidei species used in phylogenetic analysis. (DOCX) [file pone.0073561.s003.docx]

**Table S1. GenBank accession numbers for mitochondrial genome sequences of the Percoidei species used in phylogenetic analysis.**

| Family | Species | Accession No. |
| --- | --- | --- |
| Acropomidae | *Doederleinia berycoides* | NC_009867 |
| Arripidae | *Arripis trutta* | NC_015787 |
| Caesionidae | *Pterocaesio tile* | NC_004408 |
| Carangidae | *Caranx melampygus* | NC_004406 |
| Centracanthidae | *Spicara maena* | NC_009854 |
| Centrarchidae | *Micropterus salmoides* | NC_014686 |
| Chaetodontidae | *Chaetodon auripes* | NC_009870 |
| Emmelichthyidae | *Emmelichthys struhsakeri* | NC_004407 |
| Enoplosidae | *Enoplosus armatus* | NC_013181 |
| Epinephelidae | *Aethaloperca rogaa* | KC593376* |
|  | *Anyperodon leucogrammicus* | GQ131336 |
|  | *Cephalopholis argus* | KC593377* |
|  | *Cephalopholis* *sonnerati* | KC593378* |
|  | *Cromileptes altivelis* | KC593375* |
|  | *Epinephelus akaara* | EU043377* |
|  | *Epinephelus areolatus* | KC593374* |
|  | *Epinephelus awoara* | JX109835* |
|  | *Epinephelus bruneus* | JQ518289 |
|  | *Epinephelus coioides* | EU043376* |
|  | *Epinephelus epistictus* | KC593373* |
|  | *Epinephelus fuscoguttatus* | JX119192* |
|  | *Epinephelus lanceolatus* | HQ660062 |
|  | *Epinephelus moara* | JQ518290 |
|  | *Epinephelus trimaculatus* | KC593372* |
|  | *Hyporthodus octofasciatus* | JX135579* |
|  | *Hyporthodus septemfasciatus* | FJ594966 |
|  | *Plectropomus areolatus* | KC262636* |
|  | *Plectropomus leopardus* | JQ420074* |
|  | *Triso dermopterus* | KC593371* |
|  | *Variola albimarginata* | KC593370* |
|  | *Variola louti* | KC593369* |
| Kyphosidae | *Kyphosus cinerascens* | NC_013138 |
| Latidae | *Lates calcarifer* | NC_007439 |
| Lutjanidae | *Lutjanus argentimaculatus* | NC_016661 |
| Malacanthidae | *Branchiostegus albus* | NC_012905 |
| Monodactylidae | *Monodactylus argenteus* | NC_009858 |
| Oplegnathidae | *Oplegnathus fasciatus* | NC_010968 |
| Pentacerotidae | *Histiopterus typus* | NC_015786 |
| Percidae | *Etheostoma radiosum* | NC_005254 |
| Pomacanthidae | *Centropyge loricula* | NC_009872 |
| Pomadasyidae | *Parapristipoma trilineatum* | NC_009857 |
| Sciaenidae | *Collichthys lucidus* | NC_014350 |
| Serranidae | *Hypoplectrus gemma* | NC_013832 |
| Sillaginidae | *Sillago sihama* | NC_016672 |
| Sinipercidae | *Siniperca chuatsi* | NC_015822 |
| Sparidae | *Pagrus auriga* | NC_005146 |
| Theraponidae | *Rhynchopelates oxyrhynchus* | NC_013141 |
| Toxotidae | *Toxotes chatareus* | NC_013151 |

*Sequenced in this study.
